# Rh-Catalyzed Atroposelective Single-Carbon Insertion

**DOI:** 10.1021/jacs.5c06139

**Published:** 2025-07-07

**Authors:** Bowen Li, Valero G. Alfonso, Alessio Puggioli, Albert Solé-Daura, Feliu Maseras, Marcos G. Suero

**Affiliations:** ‡ 202569 Institute of Chemical Research of Catalonia (ICIQ-CERCA), The Barcelona Institute of Science and Technology, Països Catalans 16, 43007 Tarragona, Spain; & ICREA, Pg Lluis Companys 23, 08010 Barcelona, Spain; † Departament de Química Analítica i Química Orgánica, Universitat Rovira i Virgili, Marcel·lí Domingo 1, 43007 Tarragona, Spain

## Abstract

Single-carbon insertion processes have gained considerable momentum over the past few years. Although innovative methods have emerged for converting indole or indene into quinoline or naphthalene cores, the enantioselective version of such ring-expansions to create (hetero)­biaryl atropisomers has not been developed. Herein, we report the first enantioselective single-carbon insertion that converts 3-aryl indoles to atropochiral quinolines. Key in the process was the generation of a chiral Rh-carbynoid that mediated in the creation of the stereogenic C­(*sp* ^2^)–C­(*sp* ^2^) axis.

## Graphical abstract

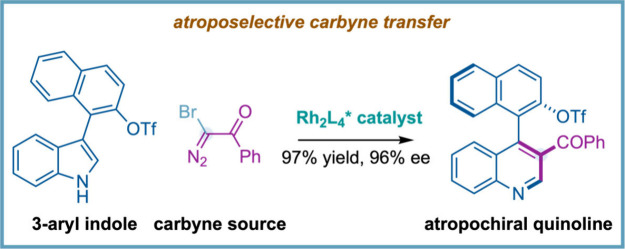

Atropisomers, defined as molecular entities containing a rotationally restricted single bond, are present in a wide range of natural products, materials, chiral ligands, pharmaceuticals, and agrochemicals (Figure 1A).^1^ Among them, (hetero)­biaryl atropisomers represent the prototypical and most extensively studied systems. Early synthesis of atropisomers often relied on stoichiometric amounts of chiral (auxiliary) reagents. However, the challenge of creating a stereogenic axis through enantioselective catalysis has attracted huge attention and resulted in a wealth of methodologies based on desymmetrizations, (dynamic) kinetic resolutions, cross-couplings, or *de novo* ring construction.^2^ Despite these developments, catalytic strategies involving atroposelective ring-expansions remain unexplored.

**Figure 1:**
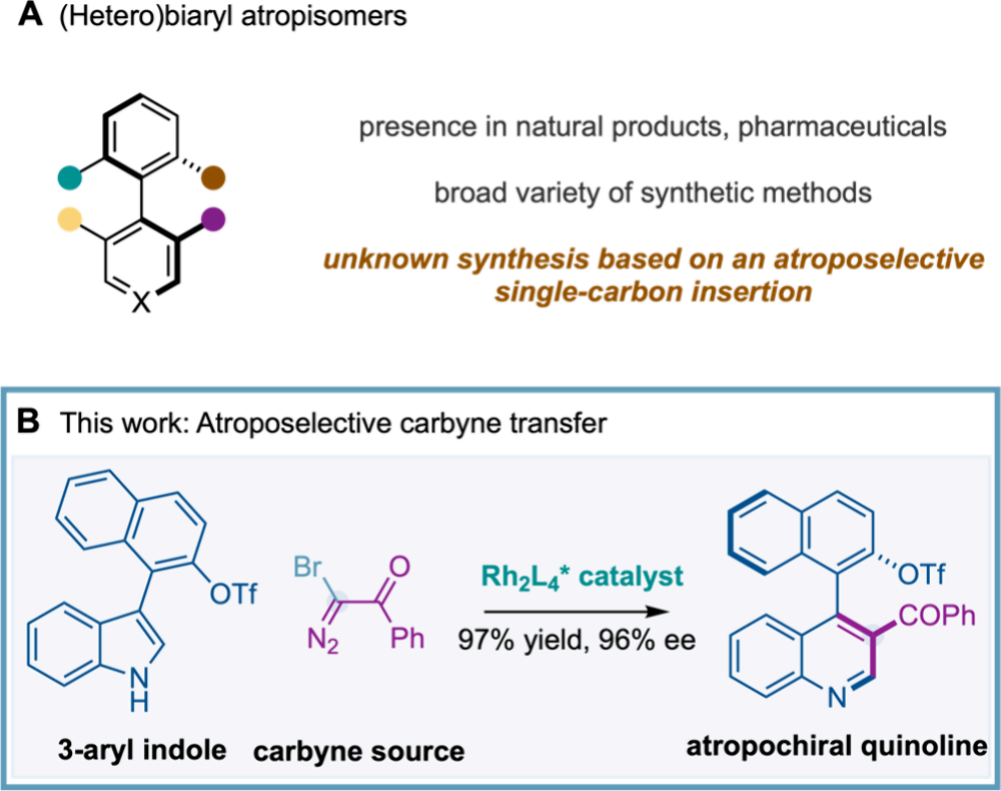
Atroposelective single-carbon insertion.

Two related examples of this class were reported by the groups of Yeung^3^ and Song,^4^ that involved a dynamic kinetic resolution semipinacol rearrangement and a Pd-catalyzed ring-expansion of benzosilacyclobutenes with alkynes to form axially chiral silacyclohexenyl arenes, respectively. An unexplored yet unknown strategy for the development of an atroposelective ring-expansion could involve a single-carbon insertion into the unsaturated C–C bonds of (hetero)­aromatic rings, enabling the creation of a stereogenic axis during the enantiodetermining step. Such a type of transformation, categorized as skeletal editing, has received considerable attention.^5^ However, catalytic enantioselective single-carbon insertions remain unexplored and limited to the creation of chiral centers^6^ or planes.^7^

Herein, we report the first enantioselective single-carbon insertion of 3-aryl indoles to access atropochiral quinolines (Figure 1B). Key to the success of this atroposelective Ciamician–Dennstedt-type transformation was the identification of suitable catalytically generated chiral Rh-carbynoid, as a cationic carbyne source (:^+^C–R), which efficiently forged the chiral axis with excellent enantiocontrol.

Our group has pioneered the catalytic generation of chiral Rh­(II)-carbynoids^8,5l,5p^ —a class of Rh­(II)-carbenes bearing an aryl–I^(III)^ leaving group generated from diazo hypervalent iodine reagents^9^ and enantiopure dirhodium carboxylate catalyst^10^—and their application in (i) the enantioselective single-carbon insertion into alkenes through the generation of an enantioenriched cyclopropane–I^(III)^ intermediate^6b^ and (ii) the enantioselective aryl C–H bond cyclopropylation through the generation of a chiral donor/acceptor Rh­(II)-carbene (Figure 2A).^11^ Recently, we questioned whether chiral Rh­(II)-carbynoid (***int-I***) could be a suitable species to promote an atroposelective Ciamician–Dennstedt-type reaction. While this single-carbon insertion into the C­(*sp* ^2^)–C­(*sp* ^2^) double bond of the pyrrolic core of indoles has recently attracted considerable attention, no enantioselective variant has been reported. We hypothesized that ***int-I*** could cyclopropanate 3-(*ortho*-substituted-aryl) indoles and provide access to cyclopropyl intermediates ***int-II*** with excellent enantiocontrol (Figure 2B). We anticipated that during the creation of the three chiral centers of the cyclopropyl ring a chiral C­(*sp* ^3^)–C­(*sp* ^2^) axis could be forged. Finally, we hypothesized that the concerted ring-opening of ***int-II*** and departure of the leaving group would be faster than possible erosion of the enantioselectivity through the rotation of the chiral axis.

**Figure 2:**
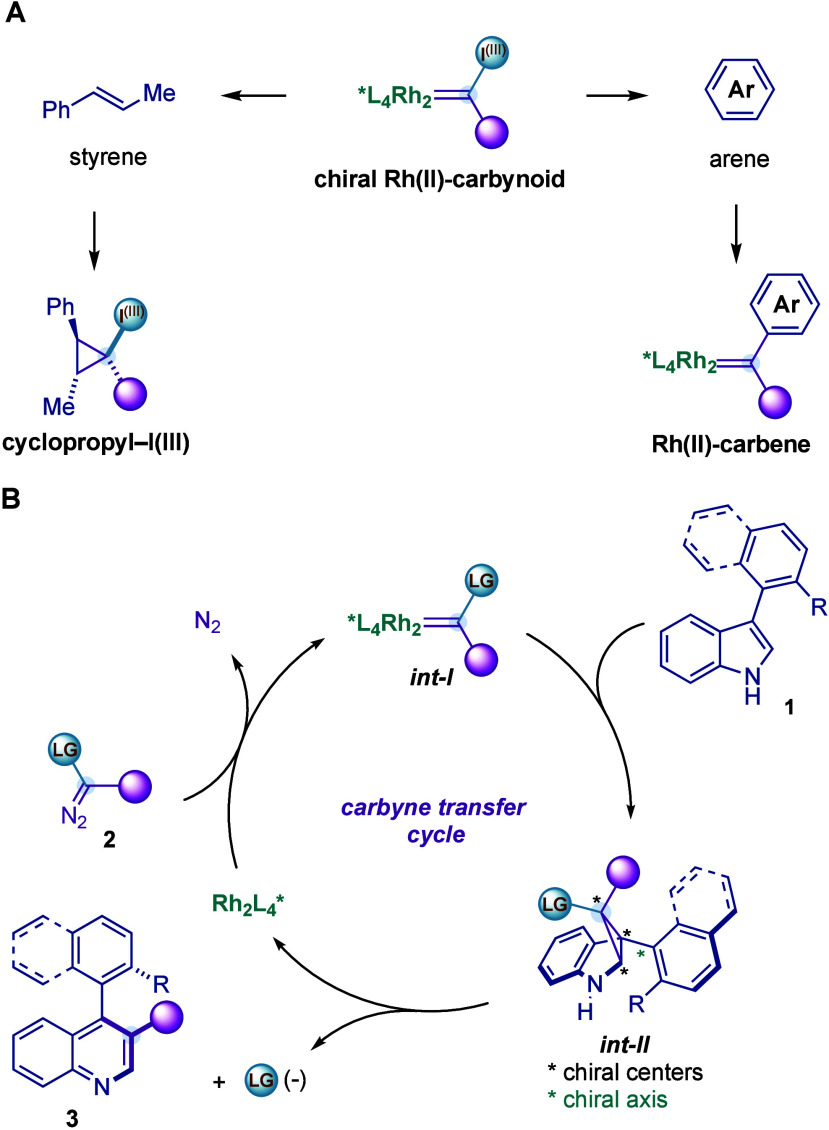
Mechanistic hypothesis.

We tested our hypothesis with indole **1a**, pseudocyclic hypervalent iodine compound **2a**, Rh_2_(esp)_2_ (esp = α,α,α′,α′-tetramethy-1,3-benzenedipropanoate)^12^ as catalyst in CH_2_Cl_2_ at −50 °C. Although we observed full conversion of **2a** we have not detected formation of the desired quinoline derivative **3a** and instead a complex mixture of unidentified products (Table 1, entry 1). Then we wondered whether decreasing the electrophilicity and steric bulkiness of the Rh­(II)-carbynoid by replacing the leaving group Ar–I^(III)^PF_6_ with a halide atom would be beneficial for a selective single-carbon insertion into the indole. Gratifyingly, the reaction carried out with **1a** and α-diazo bromoester **2b** under the previous reaction conditions led to the formation of quinoline **3a** in moderate yield (entry 2). Supercritical fluid chromatography mass spectrometry (SFC-MS) analysis on a chiral stationary phase confirmed that **3a** was obtained as a mixture of atropisomers.

**Table 1:**
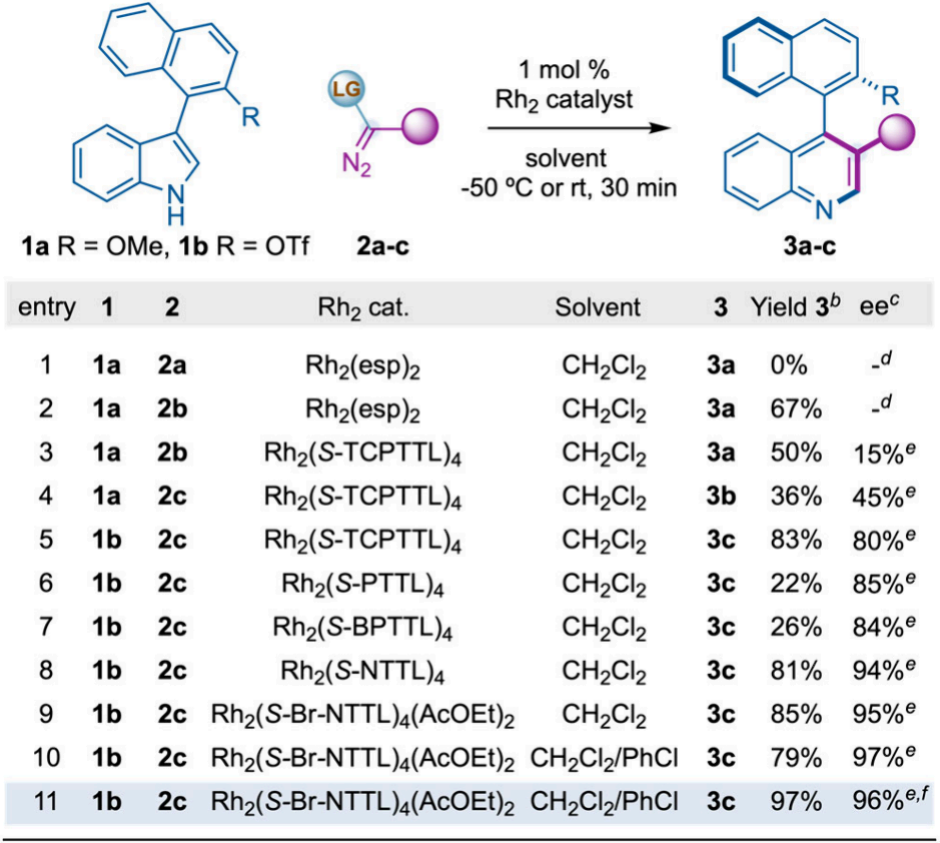
Optimization Studies^a^

a Performed with **1** (0.1 mmol, 1 equiv), **2a**–**c** (0.2 mmol, 2 equiv), and Rh_2_ catalyst (0.001 mmol, 1 mol %) in solvent (0.05 M). **2b**–**c** were synthesized with the corresponding terminal diazo compound (0.2 mmol, 2 equiv), 1,8-diazabicyclo[5.4.0]­undec-7-ene (DBU) (0.15 mmol, 1.5 equiv), and *N*-bromosuccinimide (NBS) (0.24 mmol, 2.4 equiv) in CH_2_Cl_2_ at 0 °C for 10 min.

b Isolated yield of pure product **3a**–**c**.

c Enantiomeric excess (ee) was determined by supercritical fluid chromatography mass spectrometry (SFC-MS) analysis on a chiral stationary phase.

d Reaction carried out at −50 °C.

e Reaction carried out at room temperature (rt).

f *N*,*N*-Diisopropylethylamine (DIPEA) (0.15 mmol, 1.5 equiv) was used as base, and catalytic amounts of DBU (0.02 mmol, 10 mol %) were used for the formation of **2c**. esp = α,α,α′,α′-tetramethy-1,3-benzenedipropanoate. Enantiomeric excess (ee) was determined by SFC-MS analysis on a chiral stationary phase of the isolated pure product by using flash column chromatography.
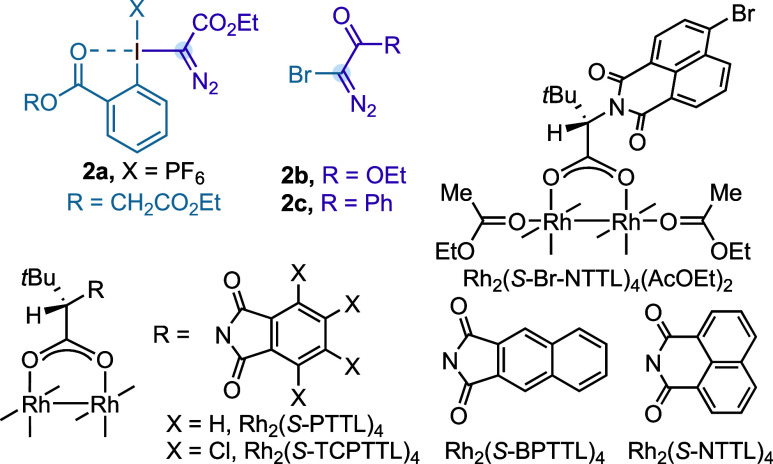

After this, we turned our attention to the development of the atroposelective single-carbon insertion by using chiral C_4_-symmetric bowl-shape dirhodium tetracarboxylate complexes as the catalyst.^13^ Initial experiments showed that 1 mol % Rh_2_(*S*-TCPTTL)_4_ (*S*-TCPTTL = *N*-tetrachlorophthaloyl-(*S*)-*tert*-leucinate) provided **3a** albeit with poor enantiocontrol (entry 3). Then, we observed that α-diazo bromoketone **2c** led to a significant increase in enantioselectivity (entry 4), probably due to a higher steric bulk of the phenyl ring of **2c** versus the ethoxy group in **2b**. Based on this result, we questioned whether replacing the methoxy substituent on the naphthalene moiety with a bulkier group could further improve the enantiocontrol. Rather than introducing *iso*-propoxy or *tert*-butoxy groups, we considered triflate to be a more suitable option due to its utility in cross-coupling reactions. We were glad to see that triflate derivative **1b** in combination with α-diazo bromoketone **2c** provided **3c** in 83% yield with 80% ee (entry 5). While *tert*-leucinate-based dirhodium catalysts substituted with (benzo)­phthaloyl groups led to similar enantioselectivities (entries 6 and 7), naphthaloyl substituents provided excellent values (entries 8 and 9, 94–95% ee). This slight increment in enantioselectivity may be due to naphthaloyl-based catalysts having a wider bowl framework able to accommodate the bounded carbynoid and bulky substrate **1**.^14,15^

Finally, the use of chlorobenzene as cosolvent (entry 10) and *i*Pr_2_EtN as base provided **3a** in excellent yield and enantioselectivity (entry 11, 97% yield, 96% ee). Control experiments carried out with the corresponding α-diazo chloro- and iodoketone under the optimized reaction conditions gave **3c** in 96% ee but in significantly lower yields (6–20%), likely due to the lower stability of the corresponding α-diazo compounds under the reaction conditions.^16,17^

After the optimization studies, we explored the scope and limitations of our Rh-catalyzed atroposelective synthesis of 4-aryl-quinoline **3** (Table 2). We observed that substitution at the C4 (**3d**,**e**), C5 (**3f**–**h**) and C6 positions (**3i**–**l**) of the indole core was well tolerated with alkyl, halide and phenyl substituents (Table 2A). While enantioselectivities were generally excellent (92–97% ee), C4-substituted indoles exhibited a notable drop in yield (54–61% yield). In contrast, indoles substituted at C7 with a fluorine atom or methyl group could not be converted to the desired quinolines **3m**,**n**.

**Table 2:**
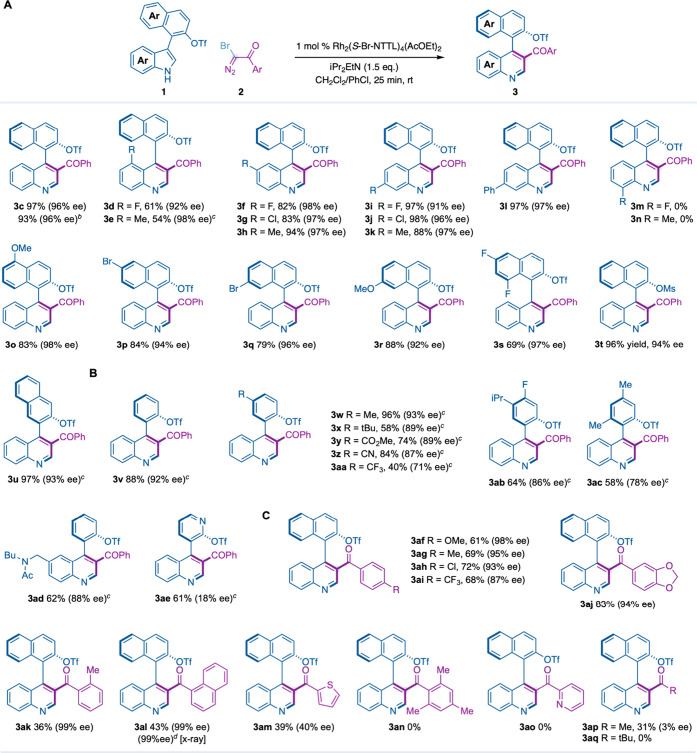
Scope of the Rh-Catalyzed Atroposelective Single-Carbon Insertion^a^

a Performed with **1** (0.1 mmol, 1 equiv), **2** (0.2 mmol, 2 equiv), iPr_2_EtN (0.15 mmol, 1.5 equiv) and Rh_2_(*S*-Br-NTTL)_4_(AcOEt)_2_ (0.001 mmol, 1 mol %) in CH_2_Cl_2_/PhCl (1:1). Yields are reported based on isolated pure product. Enantiomeric excess (ee) was determined using SFC-MS analysis on a chiral stationary phase.

b Reaction performed using 1.17 g of **1b**.

c Reaction performed at −20 °C.

d Enantiomeric excess determined after keeping **3al** at room temperature for 8 months. The absolute configuration of quinolines **3** was assigned by analogy to that of **3al**, which was determined by single-crystal X-ray diffraction analysis.

Different indole derivatives substituted with decorated naphthyl rings afforded quinolines **3o**–**s** with high efficiency (79–86% yield) and excellent enantioselectivity (93–98% ee). Notably, replacement of the triflate for a mesylate group or placing the indole ring at C3 of the naphthalene moiety did not compromise the yield or enantioselectivity (**3t**,**u**). Moreover, we observed that 3-phenyl-indole derivatives substituted with alkyl groups (**3w**,**x**,**ac**), ester (**3y**), cyano (**3z**), trifluoromethyl (**3aa**), fluoro (**3ab**) and amide (**3ad**) were tolerated (Table 2B). However, we noticed that bulky alkyl substituents (*t*Bu, *i*Pr), strong electron-withdrawing groups (CF_3_) or disubstitution on the phenyl rings decreased both yield and enantioselectivities, probably due to steric clashes or repulsive interactions with the naphthaloyl group of the catalyst. Furthermore, a pyridyl-indole provided the expected quinoline in moderate yield, albeit without enantiocontrol (**3ae**).

After the evaluation of the indole scope, we successfully explored a range of α-diazo bromoketones **2** bearing aryl substituents at the *para* (**3af**–**ai**), *meta* (**3aj**) and *ortho* (**3ak**) positions (Table 2C), as well as 1-naphthyl (**3al**) and 1-thienyl group (**3am**). α-Diazo bromoketones substituted at the two *ortho* positions of the aryl ring (**3an**) or with 2-pyridyl (**3ao**) failed to provide the corresponding atropochiral quinoline. α-Diazo derivatives substituted with methyl (**3ap**) or *t*Bu (**3aq**), instead of an (hetero)­aryl ring, led to poor yields and no enantiocontrol or no reaction, respectively. Importantly, **3c** was prepared in >1 g without compromising the efficiency of the process and it was observed that the resulting atropisomers were configurationally stable, as exemplified by compound **3al**, which retained its enantiomeric excess for over 8 months at room temperature.

To further highlight the importance of a triflate substituent in **1**, we performed control experiments using 3-aryl-indoles bearing alkyl (Me, *i*Pr) or phenyl groups (Figure 3A). Surprisingly, the expected quinoline derivatives **4**–**6** were not formed. Interestingly, aldehyde or cyano groups led to quinolines **7** and **8** with low efficiency and enantiocontrol. Moreover, we were unable to convert 2-aryl-indole to 2-aryl-quinoline under the optimized reaction conditions (**9**). The latter result is aligned with a known limitation for this type of Ciamician–Dennstedt-type transformation.^5i^

**Figure 3:**
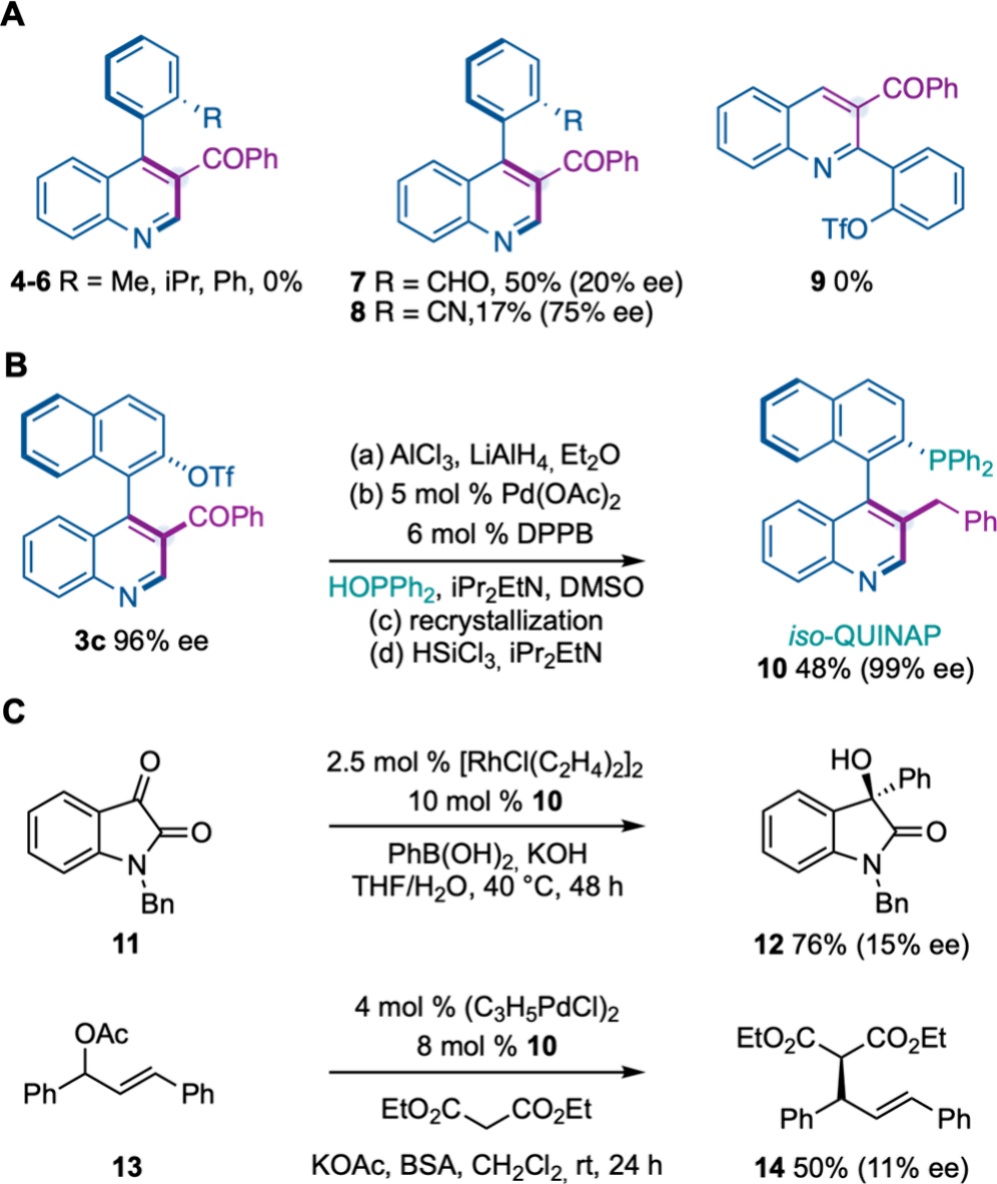
Replacing triflate for alternative groups and, synthesis/applications of chiral phosphine ligand **10**. See the SI for the experimental details.

On the other hand, considering that axially chiral biaryl motifs can be found in privileged ligands for asymmetric catalysis, such as BINAP, BINOL, and their phosphoric acid derivatives,^1^ we sought to make a chiral phosphine ligand by functionalizing the triflate group (Figure 3B). The synthesis required reduction of the ketone group (step 1) for an efficient Pd-catalyzed phosphorylation of the aryl–triflate moiety (step 2). Recrystallization and reduction of the P=O bond with a silane hydride provided phosphine ligand *iso*-QUINAP **10** in 48% yield (4 steps) and 99% ee. Finally, **10** was successfully tested in a Rh-catalyzed arylation and Pd-catalyzed asymmetric allylic alkylation and provided **12** and **14** in moderate to good yields albeit in poor enantioselectivities (Figure 3C).

Density Functional Theory (DFT) calculations were carried out to further understand the origin of the experimentally observed atroposelectivity. Calculations were performed at the B3LYP-D3BJ level using a polarized double-ζ-quality basis set and implicit solvation to account for the solvent effects of chlorobenzene (see the SI for further details).^18^ Characterization of the reaction mechanism using a model system [Rh_2_(OAc)_4_ as the catalyst, 3-(2-methylnaphthalen-1-yl)-1*H*-indole as the model substrate] for the sake of computational efficiency revealed that the product enantioselectivity is determined at the early stages of the reaction. The free energy profile for the full catalytic cycle is reported in the Supporting Information (Figures S2 and S3). The configuration of the stereogenic C­(*sp* ^2^)–C­(*sp* ^2^) axis is governed by the structure of the transition state **TS1**, which is associated with the attack of the indole from the C2 position to the carbynoid carbon atom of the corresponding Rh-carbynoid (***int-I***) to form the tetrahedral intermediate **B**. From this point, the reaction proceeds via straightforward cyclopropanation and ring-opening steps to yield the product. Notably, the computed free-energy barriers for evolving from **B** to the product are lower than the reverse barrier, leading back to the reactants. This rules out a possible Curtin–Hammett scenario in which atroposelectivity would be determined at a later stage of the mechanism.^19^

To gain deeper insight into the factors that govern enantioselectivity, we next characterized the **TS1** leading to *S* and *R* enantiomers on a real experimentally tested case, that is, using a nonsimplified model of the Rh-carbynoid and substrate **1b**. We carried out a conformational analysis on this transition state (see the SI), and the two most stable conformations, one going to the *S* product and the other to the *R* product, are shown in Figure 4. The TS yielding the *S* isomer (**TS1**_**S**_) is 2.9 kcal mol^–1^ more stable than that giving access to the *R* isomer, in good agreement with experimental findings. Visual inspection of the calculated TS structures suggested that the less stable character of **TS1**_**R**_ compared to **TS1**_**S**_ is due to steric repulsion between the triflate substituent of the substrate and the benzoyl group of the chiral Rh-carbynoid. Indeed, this conclusion was supported by additional calculations, whereby the triflate group was replaced by a less sterically demanding H atom, which led to very similar energies between **TS1**_**R**_ and **TS1**_**S**_, thus confirming that the observed selectivity arises from steric effects. It is noteworthy that the optimized structures of both TSs revealed the presence of a hydrogen bond between the NH group of the indole and an oxygen atom of one of the chiral carboxylate ligands, which might contribute to stabilizing the TSs.

**Figure 4:**
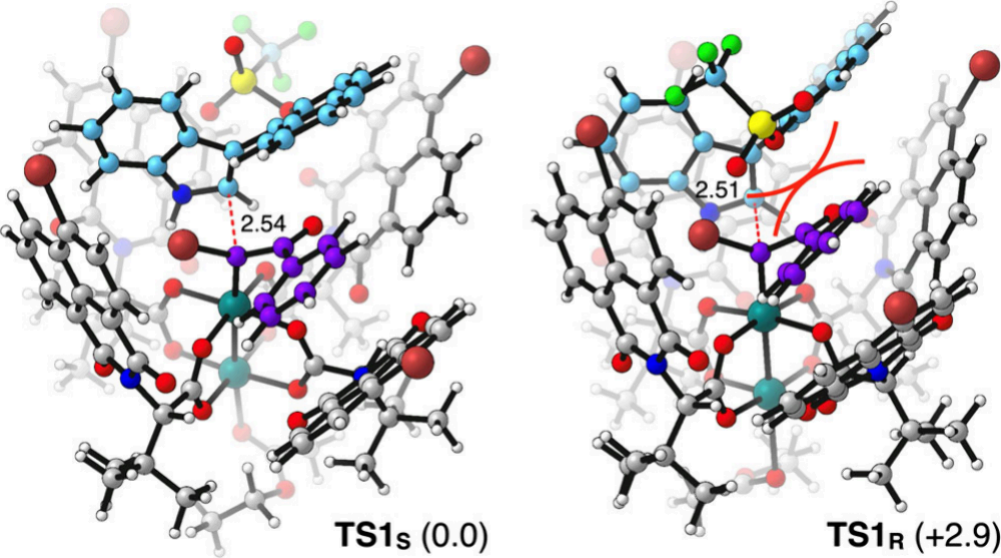
Comparison of the transition states for the enantio-determining step leading to *S* (left) and *R* (right) enantiomers of compound **3c**. Relative Gibbs free energies (kcal mol^–1^) are shown in parentheses and key distances are given in Å.

In conclusion, we have disclosed a novel atroposelective single-carbon insertion using chiral Rh-carbynoids derived from α-diazo bromoketones and an enantiopure dirhodium carboxylate catalyst. With this catalytic system, we were able to transform 3-aryl indoles in atropochiral quinolines with excellent efficiencies and enantiocontrol. It is worth highlighting that this work represents a unique example of a highly enantioselective process using α-diazo halocarbonyl compounds. DFT calculations confirmed our initial hypothesis of the origin of the atroposelectivity and key features of the Rh­(II)-carbynoid intermediates.

## Supplementary Material



## Notes

Experimental procedures and spectral data (PDF)
