# Supplementary material for: Rh-Catalyzed Atroposelective Single-Carbon Insertion
Source: J Am Chem Soc. 2025 Jul 7;147(28):24206–12. doi: 10.1021/jacs.5c06139 (PMC12272680; doi:10.1021/jacs.5c06139)
Supplement: Supplementary file 1 [file ja5c06139_si_001.pdf]

Rh-Catalyzed Atroposelective Single-Carbon Insertion

Bowen Li‡, Valero G. Alfonso‡,^, Alessio Puggioli‡,^, Albert Solé-Daura‡, Feliu Maseras‡* and Marcos G. Suero‡,&,*

‡Institute of Chemical Research of Catalonia (ICIQ-CERCA), The Barcelona Institute of Science and Technology, Països Catalans 16, 43007 Tarragona, Spain. &ICREA, Pg. Lluis Companys 23, 08010 Barcelona, Spain. ^Departament de Química Analítica i Química Orgánica, Universitat Rovira i Virgili, Calle Marcel·lí Domingo, 1, Tarragona, 43007, Spain

*Correspondence to mgsuero@iciq.es

Supporting Information

Table of Contents

1. General information. **S1**

2. Synthesis of starting materials **S2**

3. Rh-catalyzed atroposelective single-carbon insertion: reaction optimization and scope. **S27**

4. -OTf Control experiments **S92**

5. Synthesis of *iso*-QUINAP ligand 4 and applications **S99**

6. X-Ray crystal data of 3al **S108**

7. Computational studies **S110**

7. References **S210**

8. Copies of NMR spectra. **S214**

1. General information.

All reagents were used as purchased without further purification. Anhydrous solvents were dried by passing through an activated alumina column on a PureSolvTM solvent purification system (Innovative Technologies, Inc., MA). Analytical thin layer chromatography (TLC) was carried out using aluminum sheets with 0.2 mm of silica gel (Merck GF234). Visualization of the developed chromatogram was performed by irradiation with UV light. Flash column chromatography was performed on silica gel (Aldrich, 230-400 mesh) or neutral silica gel (Material Harvest Ltd., 230-400 mesh). Organic solutions were concentrated under reduced pressure on a Büchi rotatory evaporator. Yields refer to purified compounds unless otherwise noted. NMR spectra were recorded at 298 K on Bruker Avance 300, Bruker Avance 400 Ultrashield or Bruker Avance 500 Ultrashield apparatuses. Coupling constants (J) are quoted in hertz (Hz). Multiplicity is reported with the following abbreviations: s = singlet, brs = broad singlet, d = doublet, t = triplet, q = quartet, dt = doublet of triplets, td = triplet of doublets, tt = triplet of triplets, m = multiplet, app = apparent. Melting points were measured using open glass capillaries in a Büchi B540 apparatus. Mass spectra were recorded on a Waters LCT Premier spectrometer. Liquid chromatography-mass spectrometry (LC-MS) and gas chromatographymass spectrometry (GC-MS) analysis were carried out in Agilent 1260 Infinity – 6130 Quadrupole and Agilent 7890B - 5977A MSD, respectively. The enantiomeric excess was determined by SFC-MS analysis on a chiral stationary phase performed on Agilent 1260 Infinity II SFC system on Daicel Immobilized type chiral columns unless otherwise stated.

2. Synthesis of starting materials.

2.1 Synthesis of Rh_2(*S*-Br-NTTL)$_4$(AcOEt)$_2$.

Rh_2(*S*-Br-NTTL)$_4$ [1](900.5 mg, 0.5 mmol) was stirred at room temperature in ethyl acetate (5mL) in a 25 mL round bottom flask over an hour and solvent was removed under rotary evaporation and then dried under high vacuum to obtain a green powder quantitatively (981.3 mg, 99 %).

^{1}H NMR (400 MHz, $CDCl_3$) δ 8.84 – 8.75 (m, 1H), 8.61 – 8.47 (m, 2H), 8.39 – 8.25 (m, 3H), 8.15 – 8.06 (m, 1H), 7.95 – 7.77 (m, 2H), 7.72 – 7.59 (m, 1H), 5.84 – 5.73 (m, 2H), 4.11 (q, *J* = 7.1 Hz, 2H), 2.04 (s, 3H), 1.30 – 1.25 (m, 18H), 1.22 (t, *J* = 7.1 Hz, 3H).
^{13}C NMR (101 MHz, $CDCl_3$) δ 187.4, 171.8, 164.3, 162.7, 133.3, 132.9, 132.6, 132.4, 132.0, 131.7, 131.1, 130.7, 130.3, 129.8, 129.5, 128.92, 128.87, 128.8, 127.8, 123.3, 123.23, 123.20, 122.5, 122.4, 62.2, 60.7, 36.3, 28.9, 21.2, 14.3.

2.2 Synthesis of substrates

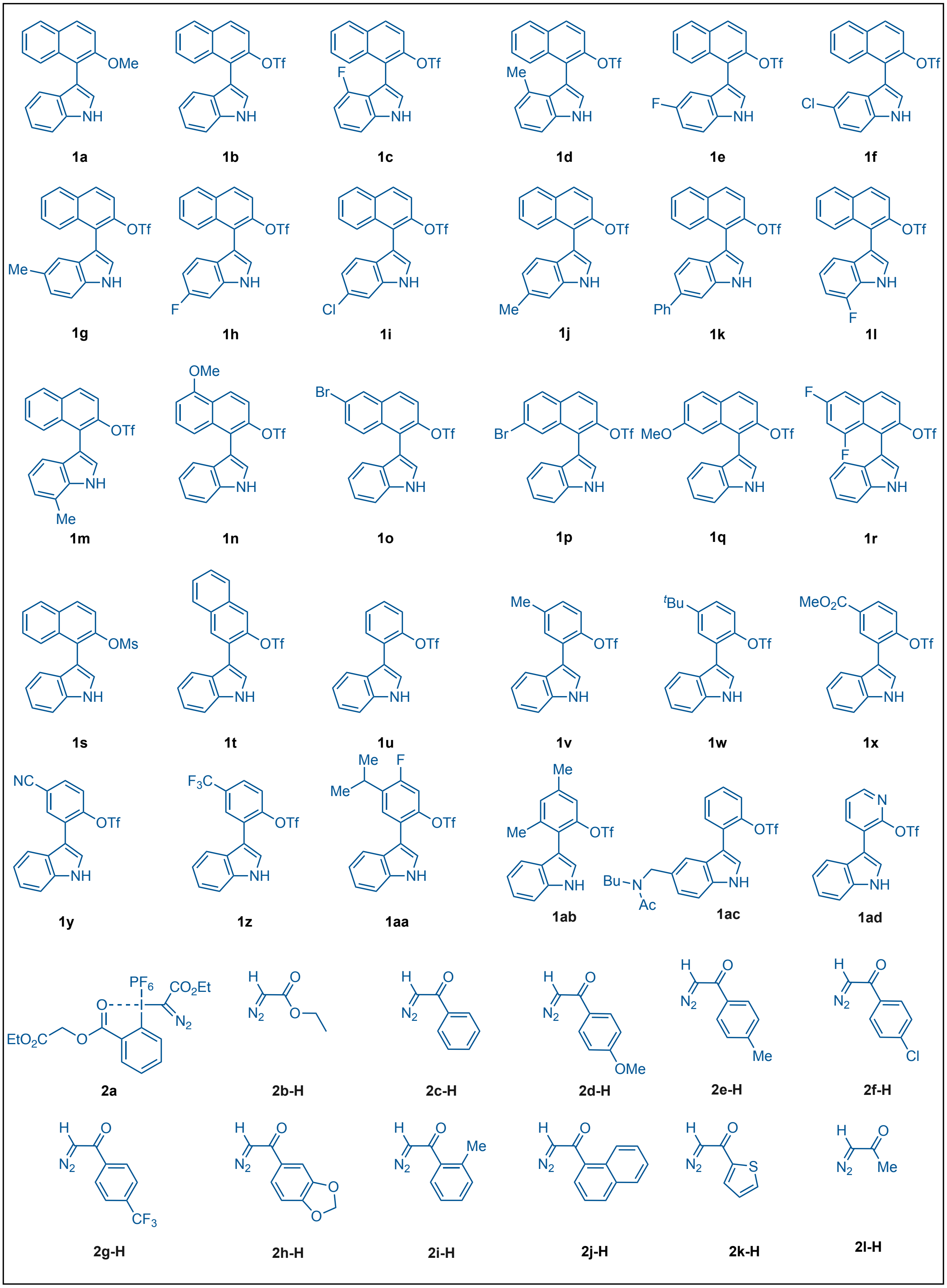
1a 1b 1c 1d 1e 1f
1g 1h 1i 1j 1k 1l
1m 1n 1o 1p 1q 1r
1s 1t 1u 1v 1w 1x
1y 1z 1aa 1ab 1ac 1ad
2a 2b-H 2c-H 2d-H 2e-H 2f-H
2g-H 2h-H 2i-H 2j-H 2k-H 2l-H

2b-H was commercially available and used directly without further purification. Substrates **1a-b**,[2] **1e-i**,[2] **1n-o**,[3] **1q**,[3] **2a**,[4] **2c-f-H**,[5] **2g-H**,[6] **2h-H**,[5] **2i-H**[7] and **2j-l-H**[5] are known compounds. Below are the general procedures used for the synthesis of 3-aryl indoles:

General procedure A:[2]

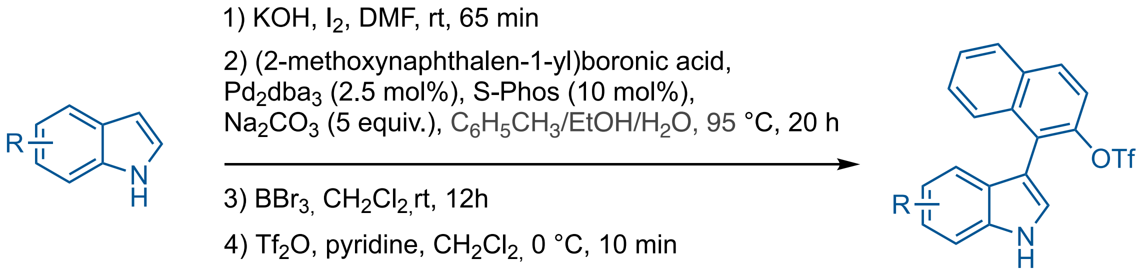

1) To a 100 mL round-bottom flask equipped with a stirring bar were added the corresponding indole (10 mmol, 1.0 equiv.), dimethylformamide (15 mL) and potassium hydroxide (1.40 g, 25 mmol, 2.5 equiv.). The reaction was stirred at room temperature for 20 minutes and a solution of iodine (2.79 g, 11 mmol, 1.1 equiv.) in dimethylformamide (5 mL) was added. The resulting mixture was stirred for an additional 45 minutes. Then, the reaction mixture was poured into cold aqueous solution of Na_2SO_3 (300 mL, 0.1 M) and the precipitate was collected by filtration, washed with water, and directly used for the next step.
2) To a 250 mL two-neck round-bottom flask equipped with a stirring bar were added 3-iodoindole derivatives (10 mmol, 1.0 equiv.), (2-methoxynaphthalen-1-yl)boronic acid (2.02g, 11 mmol, 1.1 equiv.), Pd_2dba_3 (229 mg, 2.5 mol%), *S*-Phos (411 mg, 10 mol%) and Na_2CO_3 (5.30 g, 50 mmol, 5 equiv.). The flask was sealed before being evacuated and backfilled with argon three times. Then, toluene (60 mL), ethanol (20 mL) and water (20 mL) were added and the reaction mixture was stirred at 95 °C for 20 hours under argon. After completion monitored by TLC, the reaction mixture was cooled to room temperature, quenched with water (150 mL) and extracted with ethyl acetate (3 x 50 mL). The combined organic layer was washed with brine and dried over anhydrous Na_2SO_4. The solvent was removed under *vacuum* and the crude was purified by flash column chromatography on silica gel to afford the corresponding 3-(2-methoxynaphthalen-1-yl)-1*H*-indole derivatives.
3) To a 50 mL oven-dried round-bottom flask equipped with a stirring bar was added 3-(2-methoxynaphthalen-1-yl)-1*H*-indole derivatives (3-7 mmol, 1.0 equiv.). The flask was

sealed before being evacuated and backfilled with argon three times. The flask was sealed before being evacuated and backfilled with argon three times. Then, anhydrous dichloromethane (10-20 mL) was added followed by slow addition of BBr_3 (9-21 mmol, 3.0 equiv.). The reaction mixture was stirred at room temperature for 12 hours. The reaction was quenched with ice water (100 mL) and extracted with dichloromethane (3 x 20 mL). The combined organic layer was dried over anhydrous Na_2SO_4 and the solvent was removed under *vacuum*. The crude compound was used directly for the next step without purification.

4) To a 50 mL oven-dried round-bottom flask equipped with a stirring bar was added 1-(1*H*-indol-3-yl)naphthalen-2-ol derivatives (2-6 mmol, 1.0 equiv.). The flask was sealed before being evacuated and backfilled with argon three times. Pyridine (4-12 mmol, 2.0 equiv.) and anhydrous dichloromethane (10-20 mL) were added. The reaction mixture was cooled to 0 °C and Tf_2O (3-9 mmol, 1.5 equiv.) was added. Stirring was continued for 10 minutes at 0 °C. Then, the reaction mixture was quenched with water (50 mL) and extracted with dichloromethane (3 x 10 mL). The combined organic layer was dried over anhydrous Na_2SO_4 and the solvent was removed under *vacuum*. The crude was purified by flash column chromatography on silica gel to afford the corresponding 3-aryl indoles.

General procedure B:[3]

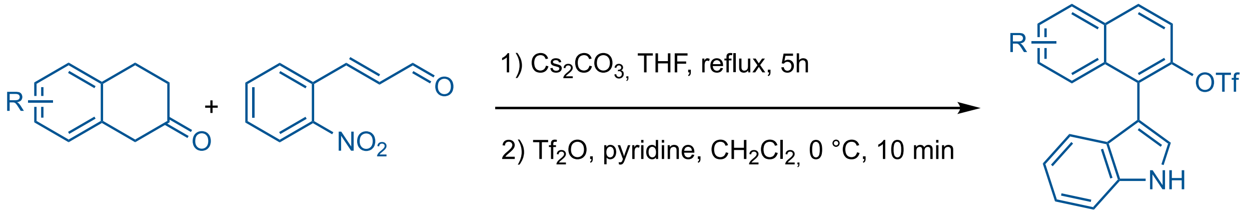

1) To a 100 mL two-neck round-bottom flask equipped with a stirring bar was added nitrocinnamaldehyde (886 mg, 5 mmol, 1.0 equiv.), the corresponding *β*-tetralone (5 mmol, 1.0 equiv.), Cs_2CO_3 (1.63 g, 5 mmol, 1.0 equiv.) and tetrahydrofuran (25 mL). Then, the reaction mixture was heated at reflux for 5 hours in the open air until the completion of the reaction as indicated by TLC. The solvent was removed under *vacuum* and the crude was purified by flash column chromatography on silica gel to afford the corresponding 1-(1*H*-indol-3-yl)naphthalen-2-ol derivates.

2) To a 50 mL oven-dried round-bottom flask equipped with a stirring bar was added 1-(1*H*-indol-3-yl)naphthalen-2-ol derivatives (2 mmol, 1.0 equiv.). The flask was sealed before being evacuated and backfilled with argon three times. Pyridine (317 mg, 4 mmol, 2.0 equiv.) and anhydrous dichloromethane (10 mL) were added. The reaction mixture was cooled to 0 °C and Tf_2O (3 mmol, 1.5 equiv.) was added. Stirring was continued for 10 minutes at 0 °C. Then, the reaction mixture was quenched with water (50 mL) and extracted with dichloromethane (3 x 15 mL). The combined organic layer was dried over anhydrous Na_2SO_4 and the solvent was removed under *vacuum*. The crude was purified by flash column chromatography on silica gel to afford the corresponding 3-aryl indoles.

General procedure C:[2]

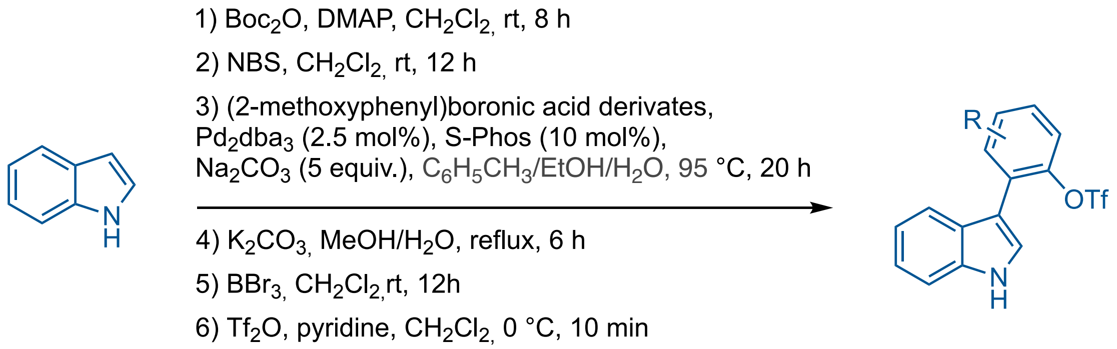

1) To a 100 mL round-bottom flask equipped with a stirring bar were added indole (10 mmol, 1.0 equiv.), Boc_2O (2.40 g, 11 mmol, 1.1 equiv.), 4-dimethylaminopyridine (122 mg, 1 mmol, 0.1 equiv.) and dichloromethane (20 mL). The reaction was stirred at room temperature for 8 hours. The reaction was quenched with water (50 mL) and extracted with dichloromethane (3 x 15 mL). The combined organic layer was dried over anhydrous Na_2SO_4 and the solvent was removed under *vacuum*. The crude compound was used directly for the next step without purification.
2) To a 100 mL round-bottom flask equipped with a stirring bar were added the corresponding *tert*-butyl 1*H*-indole-1-carboxylate (10 mmol, 1 equiv.), dichloromethane (20 mL) and *N*-bromosuccinimide (NBS, 1.96 g, 11 mmol, 1.1 equiv,). The reaction mixture was stirred at room temperature for 12 hours. Then, the reaction was quenched with water (50 mL) and the mixture was extracted with dichloromethane (3 x 15 mL). The combined organic layer

was dried over anhydrous Na_2SO_4 and the solvent was removed under *vacuum*. The crude compound was used directly for the next step without purification.

3) To a 250 mL two-neck round-bottom flask equipped with a stirring bar were added the mixture of *tert*-butyl 3-bromo-1*H*-indole-1-carboxylate (2.96 g, 10 mmol, 1.0 equiv.), (2-methoxyphenyl)boronic acid derivates (11 mmol, 1.1 equiv.), Pd_2dba_3 (229 mg, 2.5 mol%), *S*-Phos (411 mg, 10 mol%) and Na_2CO_3 (5.30 g, 50 mmol, 5.0 equiv.). The flask was sealed before being evacuated and backfilled with argon three times. A mix of toluene (60 mL), ethanol (20 mL) and water (20 mL) was added and the reaction mixture was stirred at 95 °C for 20 hours under argon. After completion monitored by TLC, the reaction mixture was cooled to room temperature, quenched with water (150 mL) and extracted with ethyl acetate (3 x 50 mL). The combined organic layer was washed with brine and dried over anhydrous Na_2SO_4. The solvent was removed under *vacuum* and the crude was purified by flash column chromatography on silica gel to afford the corresponding *tert*-butyl 3-(2-methoxyphenyl)-1*H*-indole-1-carboxylate derivates.
4) To a 100 mL round-bottom flask equipped with a stirring bar were added the corresponding 3-(2-methoxyphenyl)-1*H*-indole derivatives (8 mmol, 1.0 equiv.), K_2CO_3 (3.32g, 24 mmol, 3.0 equiv.), methanol (40 mL) and water (8 mL). The reaction was heated at reflux for 6 hours. Then, the reaction mixture was cooled to room temperature, diluted with water (50 mL) and extracted with dichloromethane (3 x 20 mL). The combined organic layer was dried over anhydrous Na_2SO_4 and the solvent was removed under *vacuum.* The crude was purified by flash column chromatography on silica gel to afford the compound 3-(2-methoxyphenyl)-1*H*-indole derivatives.
5) To a 50 mL oven-dried round-bottom flask equipped with a stirring bar was added 3-(2-methoxyphenyl)-1*H*-indole derivatives (3-7 mmol, 1.0 equiv.). The flask was sealed before being evacuated and backfilled with argon three times. Then, anhydrous dichloromethane (10-20 mL) was added followed by slow addition of BBr_3 (9-21 mmol, 3.0 equiv.). The reaction mixture was stirred at room temperature for 12 hours. The reaction was quenched with ice water (100 mL) and extracted with dichloromethane (3 x 20 mL). The combined organic layer was dried over anhydrous Na_2SO_4 and the solvent was removed under *vacuum*. The crude compound was used directly for the next step without purification.

6) To a 50 mL oven-dried round-bottom flask equipped with a stirring bar was added 2-(1*H*-indol-3-yl)phenol derivatives (2 mmol, 1.0 equiv.). The flask was sealed before being evacuated and backfilled with argon three times. Pyridine (317 mg, 4 mmol, 2.0 equiv.) and anhydrous dichloromethane (10 mL) were added. The reaction mixture was cooled to 0 °C and Tf_2O (3 mmol, 1.5 equiv.) was added. Stirring was continued for 10 minutes at 0 °C. Then, the reaction mixture was quenched with water (50 mL) and extracted with dichloromethane (3 x 15 mL). The combined organic layer was dried over anhydrous Na_2SO_4 and the solvent was removed under *vacuum*. The crude was purified by flash column chromatography on silica gel to afford the corresponding 3-aryl indoles.

General procedure C*:

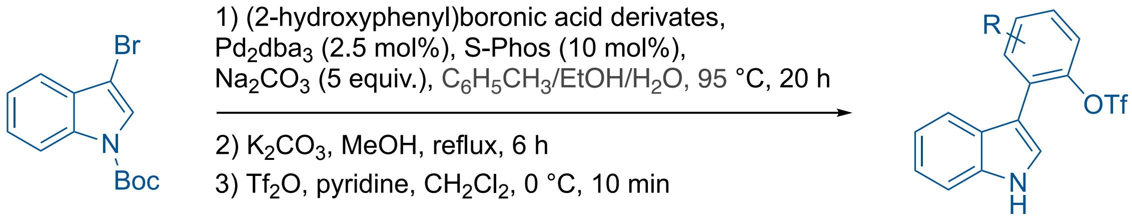

1) To a 250 mL two-neck round-bottom flask equipped with a stirring bar were added the mixture of *tert*-butyl 3-bromo-1*H*-indole-1-carboxylate (1.48 g, 5 mmol, 1.0 equiv.), (2-hydroxyphenyl)boronic acid derivates (5 mmol, 1.0 equiv.), Pd_2dba_3 (114 mg, 2.5 mol%), *S*-Phos (206 mg, 10 mol%) and Na_2CO_3 (2.65 g, 50 mmol, 5.0 equiv.). The flask was sealed before being evacuated and backfilled with argon three times. A mix of toluene (30 mL), ethanol (10 mL) and water (10 mL) were added and the reaction mixture was stirred at 95 °C for 20 hours under argon. After completion monitored by TLC, the reaction mixture was cooled to room temperature, quenched with water (80 mL) and extracted with ethyl acetate (3 x 25 mL). The combined organic layer was washed with brine and dried over anhydrous Na_2SO_4. The solvent was removed under *vacuum* and the crude was purified by flash column chromatography on silica gel to afford the corresponding *tert*-butyl 3-(2-hydroxyphenyl)-1*H*-indole-1-carboxylate derivates.
2) To a 100 mL round-bottom flask equipped with a stirring bar were added the corresponding 3-(2-hydroxyphenyl)-1*H*-indole derivatives (4 mmol, 1.0 equiv.), K_2CO_3 (1.66g, 12 mmol, 3.0 equiv.) and methanol (20 mL). The reaction was heated at reflux for 6 hours. Then, the

reaction mixture was cooled to room temperature, diluted with water (25 mL) and extracted with dichloromethane (3 x 10 mL). The combined organic layer was dried over anhydrous Na_2SO_4 and the solvent was removed under *vacuum.* The crude was used directly for the next step without purification.

3) To a 50 mL oven-dried round-bottom flask equipped with a stirring bar was added 2-(1*H*-indol-3-yl)phenol derivatives (2-4 mmol, 1.0 equiv.). The flask was sealed before being evacuated and backfilled with argon three times. Pyridine (2.0 equiv.) and anhydrous dichloromethane (15 mL) were added. The reaction mixture was cooled to 0 °C and Tf_2O (1.5 equiv.) was added. Stirring was continued for 10 minutes at 0 °C. Then, the reaction mixture was quenched with water (25 mL) and extracted with dichloromethane (3 x 10 mL). The combined organic layer was dried over anhydrous Na_2SO_4 and the solvent was removed under *vacuum*. The crude was purified by flash column chromatography on silica gel to afford the corresponding 3-aryl indoles.

1-(4-Fluoro-1*H*-indol-3-yl)naphthalen-2-yl trifluoromethanesulfonate (1c)

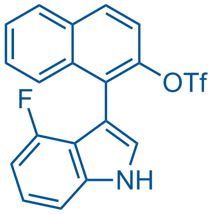

This compound was synthesized based on the general procedure **A** using 4-fluoro-1*H*-indole (1.35 g, 10.0 mmol). Purification by flash column chromatography (hexane/ethyl acetate = 15/1) provided the title compound as a white solid (687 mg, 17% yield).

^{1}H NMR (400 MHz, $CDCl_3$) δ 8.57 (brs, 1H), 7.99 – 7.90 (m, 2H), 7.86 (d, *J* = 8.4 Hz, 1H), 7.56 (ddd, *J* = 8.1, 6.8, 1.3 Hz, 1H), 7.52 – 7.43 (m, 2H), 7.32 (d, *J* = 2.5 Hz, 1H), 7.28 (d, *J* = 8.2 Hz, 1H), 7.19 (td, *J* = 8.0, 4.9 Hz, 1H), 6.77 (ddd, *J* = 10.8, 7.8, 0.8 Hz, 1H).

^{13}C NMR (101 MHz, $CDCl_3$) δ 156.8 (d, *J* = 248.9 Hz), 145.9, 138.7 (d, *J* = 10.3 Hz), 134.6, 132.6, 130.0, 128.2, 127.4, 127.1, 126.9, 126.3, 125.6, 123.4 (d, *J* = 7.8 Hz), 119.4, 118.6 (q, *J* = 320.3 Hz), 117.0 (d, *J* = 19.3 Hz), 107.6 (d, *J* = 3.9 Hz), 105.9 (d, *J* = 19.2 Hz), 105.9 (d, *J* = 1.7 Hz).

^{19}**F NMR** (376 MHz, $CDCl_3$) δ -74.54, -122.74.

HRMS (ESI): calculated for $C_{19}H_{11}F_4NNaO_3S$ $[M+Na]^+$ m/z: 432.0288, found: 432.0294.

m.p. 137-138 °C

1-(4-Methyl-1*H*-indol-3-yl)naphthalen-2-yl trifluoromethanesulfonate (1d)

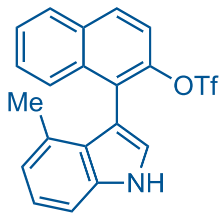

This compound was synthesized based on the general procedure **A** using 4-methyl-1*H*-indole (1.31 g, 10.0 mmol). Purification by flash column chromatography (hexane/ethyl acetate = 15/1) provided the title compound as a white solid (981 mg, 24% yield).

^{1}**H NMR** (300 MHz, $CDCl_3$) δ 8.46 (brs, 1H), 8.04 – 7.87 (m, 2H), 7.73 (ddt, *J* = 8.5, 1.5, 0.8 Hz, 1H), 7.56 (ddd, *J* = 8.2, 6.9, 1.3 Hz, 1H), 7.53 – 7.38 (m, 2H), 7.36 (dt, *J* = 8.2, 0.9 Hz, 1H), 7.29 (d, *J* = 2.6 Hz, 1H), 7.24 – 7.12 (m, 1H), 6.86 (dt, *J* = 7.1, 0.9 Hz, 1H), 1.83 (s, 3H).

^{13}**C NMR** (75 MHz, $CDCl_3$) δ 146.1, 136.4, 135.7, 132.3, 131.1, 130.0, 128.5, 128.1, 127.7, 127.5, 127.0, 126.8, 125.1, 122.9, 121.8, 119.3, 118.6 (q, *J* = 320.2 Hz), 109.3, 108.0, 18.9.

^{19}**F NMR** (282 MHz, $CDCl_3$) δ -74.49.

HRMS (ESI): calculated for $C_{20}H_{15}F_3NO_3S$ $[M+H]^+$ m/z: 406.0719, found: 406.0719.

m.p. 141-142 °C

1-(6-Methyl-1*H*-indol-3-yl)naphthalen-2-yl trifluoromethanesulfonate (1j)

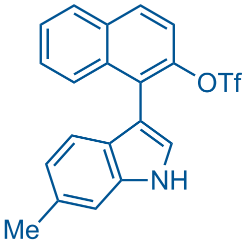

This compound was synthesized based on the general procedure **A** using 6-methyl-1*H*-indole (1.31 g, 10.0 mmol). Purification by flash column chromatography (hexane/ethyl acetate = 15/1) provided the title compound as a pale yellow foam (2.14 g, 53% yield).

^{1}H NMR (400 MHz, $CDCl_3$) δ 8.38 (brs, 1H), 8.07 – 7.88 (m, 3H), 7.61 (dd, *J* = 8.5, 6.4 Hz, 2H), 7.48 (ddd, *J* = 8.3, 6.8, 1.3 Hz, 1H), 7.36 (d, *J* = 2.4 Hz, 1H), 7.31 (s, 1H), 7.22 (d, *J* = 8.1 Hz, 1H), 7.04 (d, *J* = 8.2 Hz, 1H), 2.57 (s, 3H).
^{13}C NMR (101 MHz, $CDCl_3$) δ 145.7, 136.5, 134.1, 132.8, 132.5, 129.6, 128.2, 127.6, 127.2, 127.0, 126.6, 125.8, 125.0, 122.3, 119.8, 119.6, 118.6 (q, *J* = 320.3 Hz), 111.5, 107.8, 21.8.
^{19}F NMR (376 MHz, $CDCl_3$) δ -74.40.

HRMS (ESI): calculated for $C_{20}H_{14}F_3NNaO_3S$ $[M+Na]^+$ m/z: 428.0539, found: 428.0540.

1-(6-Phenyl-1*H*-indol-3-yl)naphthalen-2-yl trifluoromethanesulfonate (1k)

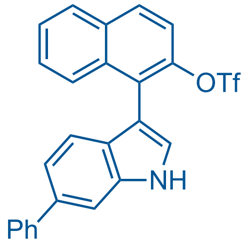

This compound was synthesized based on the general procedure **A** using 6-phenyl-1*H*-indole (0.97 g, 5.0 mmol). Purification by flash column chromatography (hexane/ethyl acetate = 15/1) provided the title compound as a white solid (1.10 g, 47% yield).

^{1}H NMR (400 MHz, $CDCl_3$) δ 8.54 (brs, 1H), 7.98 (dd, *J* = 8.7, 5.0 Hz, 2H), 7.90 (d, *J* = 8.5 Hz, 1H), 7.73 – 7.65 (m, 3H), 7.58 (ddd, *J* = 8.1, 6.8, 1.2 Hz, 1H), 7.54 (d, *J* = 9.1 Hz, 1H), 7.51 – 7.42 (m, 4H), 7.41 – 7.32 (m, 2H), 7.28 (d, *J* = 8.4 Hz, 1H).

^{13}**C NMR** (101 MHz, $CDCl_3$) δ 145.8, 142.1, 136.7, 136.3, 134.1, 132.8, 129.8, 128.9, 128.3, 127.58, 127.56, 127.4, 127.3, 127.1, 126.9, 126.3, 126.1, 120.5, 120.4, 119.7, 118.6 (q, J = 320.4 Hz), 109.9, 108.2.

^{19}**F NMR** (376 MHz, $CDCl_3$) δ -74.41.

HRMS (ESI): calculated for $C_{25}H_{17}F_3NO_3S$ $[M+H]^+$ m/z: 468.0876, found: 468.0875.

m.p. 150-151 °C

1-(7-Fluoro-1*H*-indol-3-yl)naphthalen-2-yl trifluoromethanesulfonate (1l)

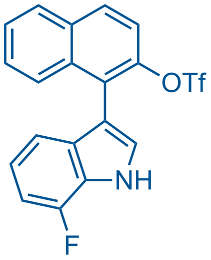

This compound was synthesized based on the general procedure **A** using 7-fluoro-1*H*-indole (1.35 g, 10.0 mmol). Purification by flash column chromatography (hexane/ethyl acetate = 15/1) provided the title compound as a white solid (1.47 g, 36% yield).

^{1}**H NMR** (400 MHz, $CDCl_3$) δ 8.71 (brs, 1H), 7.98 (t, J = 8.5 Hz, 2H), 7.82 (dd, J = 8.5, 1.2 Hz, 1H), 7.62 – 7.42 (m, 4H), 7.08 – 6.95 (m, 3H).

^{13}**C NMR** (101 MHz, $CDCl_3$) δ 149.7 (d, J = 244.2 Hz), 145.7, 134.0, 132.8, 131.4 (d, J = 4.8 Hz), 130.1, 128.3, 127.5, 127.3, 127.1, 126.1, 125.6, 124.6 (d, J = 13.9 Hz), 120.8 (d, J = 6.1 Hz), 119.7, 118.5 (q, J = 320.3 Hz), 116.0 (d, J = 3.6 Hz), 109.1 (d, J = 2.7 Hz), 107.6 (d, J = 16.0 Hz).

^{19}**F NMR** (376 MHz, $CDCl_3$) δ -74.47, -135.32.

HRMS (APCI): calculated for $C_{19}H_{11}F_4NNaO_3S$ $[M+Na]^+$ m/z: 432.0288, found: 432.0292.

m.p. 114-115 °C

1-(7-Methyl-1*H*-indol-3-yl)naphthalen-2-yl trifluoromethanesulfonate (1m)

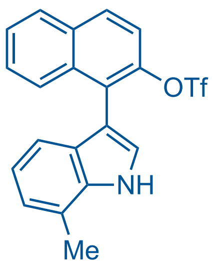

This compound was synthesized based on the general procedure **A** using 7-methyl-1*H*-indole (1.31 g, 10.0 mmol). Purification by flash column chromatography (hexane/ethyl acetate = 15/1) provided the title compound as a white solid (1.09 g, 27% yield).

^{1}H NMR (400 MHz, $CDCl_3$) δ 8.48 (brs, 1H), 8.04 – 7.88 (m, 3H), 7.64 – 7.55 (m, 2H), 7.51 – 7.40 (m, 2H), 7.19 – 7.06 (m, 3H), 2.58 (s, 3H).
^{13}C NMR (101 MHz, $CDCl_3$) δ 145.7, 135.7, 134.1, 132.8, 129.7, 128.2, 127.6, 127.5, 127.2, 127.0, 126.6, 125.3, 123.2, 120.7, 120.6, 119.6, 118.6 (q, *J* = 320.4 Hz), 117.9, 108.5, 16.6.
^{19}F NMR (376 MHz, $CDCl_3$) δ -74.37.

HRMS (ESI): calculated for $C_{20}H_{15}F_3NO_3S$ $[M+H]^+$ m/z: 406.0719, found: 406.0717.

m.p. 57-58 °C

7-Bromo-1-(1*H*-indol-3-yl)naphthalen-2-yl trifluoromethanesulfonate (1p)

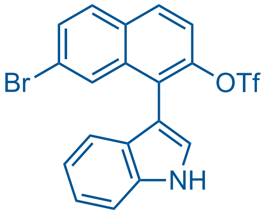

This compound was synthesized based on the general procedure **B** using 7-bromo-3,4-dihydronaphthalen-2(1*H*)-one (1.12 g, 5.0 mmol). Purification by flash column chromatography (hexane/ethyl acetate = 15/1) provided the title compound as a white solid (516 mg, 22% yield).

^{1}H NMR (400 MHz, $CDCl_3$) δ 8.56 (brs, 1H), 8.07 (d, *J* = 1.9 Hz, 1H), 7.93 (d, *J* = 9.0 Hz, 1H), 7.82 (d, *J* = 8.7 Hz, 1H), 7.65 (dd, *J* = 8.7, 2.0 Hz, 1H), 7.60 – 7.47 (m, 2H), 7.41 (d, *J* = 2.6 Hz, 1H), 7.35 – 7.25 (m, 2H), 7.16 (ddd, *J* = 8.0, 7.0, 1.0 Hz, 1H).

^{13}**C NMR** (101 MHz, $CDCl_3$) δ 146.3, 136.0, 135.4, 131.3, 130.6, 129.9, 129.7, 129.6, 127.5, 125.9, 125.7, 122.9, 122.1, 120.8, 120.2, 119.9, 118.5 (q, J = 320.5 Hz), 111.6, 107.4.
^{19}**F NMR** (376 MHz, $CDCl_3$) δ -74.45.

HRMS (ESI): calculated for $C_{19}H_{11}BrF_3NNaO_3S$ $[M+Na]^+$ m/z: 491.9487, found: 491.9490.

m.p. 124-125 °C

6,8-Difluoro-1-(1*H*-indol-3-yl)naphthalen-2-yl trifluoromethanesulfonate (1r)

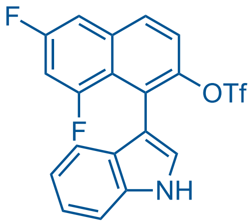

This compound was synthesized based on the general procedure **B** using 7-bromo-3,4-dihydronaphthalen-2(1*H*)-one (910 mg, 5.0 mmol). Purification by flash column chromatography (hexane/ethyl acetate = 15/1) provided the title compound as a white solid (363 mg, 17% yield).

^{1}**H NMR** (400 MHz, $CDCl_3$) δ 8.39 (s, 1H), 7.91 (dd, J = 9.1, 1.5 Hz, 1H), 7.57 – 7.37 (m, 3H), 7.33 (d, J = 2.5 Hz, 1H), 7.28 – 7.22 (m, 1H), 7.21 – 7.14 (m, 1H), 7.14 – 7.05 (m, 1H), 6.95 (ddd, J = 12.2, 8.7, 2.5 Hz, 1H).
^{13}**C NMR** (101 MHz, $CDCl_3$) δ 161.7 (dd, J = 18.1, 12.4 Hz), 159.2 (dd, J = 12.4, 4.9 Hz), 146.3 (d, J = 2.9 Hz), 135.7, 134.9 (dd, J = 10.7, 3.7 Hz), 129.4 (dd, J = 5.6, 3.1 Hz), 128.4 (d, J = 3.7 Hz), 124.4 (d, J = 3.0 Hz), 124.19, 124.17, 124.15, 122.6, 121.8, 120.4, 119.3, 118.5 (d, J = 320.2 Hz), 111.4, 109.3 (d, J = 4.4 Hz), 108.2 (d, J = 5.0 Hz), 108.0 (d, J = 5.0 Hz), 104.9, 104.7 (d, J = 2.6 Hz), 104.4.
^{19}**F NMR** (376 MHz, $CDCl_3$) δ -74.55, -104.00 (d, J = 8.8 Hz), -110.69 (d, J = 8.7 Hz).
HRMS (ESI): calculated for $C_{19}H_{11}F_5NO_3S$ $[M+H]^+$ m/z: 428.0374, found: 428.0378.

m.p. 136-137 °C

3-(1*H*-Indol-3-yl)naphthalen-2-yl trifluoromethanesulfonate (1s)

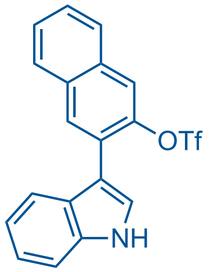

This compound was synthesized based on the general procedure **C** using (3-methoxynaphthalen-2-yl)boronic acid (2.22 g, 11.0 mmol). Purification by flash column chromatography (hexane/ethyl acetate = 15/1) provided the title compound as a white solid (2.00 g, 51% yield).

^{1}H NMR (400 MHz, $CDCl_3$) δ 8.41 (brs, 1H), 8.19 (s, 1H), 7.91 (s, 3H), 7.81 (dt, *J* = 8.0, 1.0 Hz, 1H), 7.63 – 7.44 (m, 4H), 7.35 – 7.20 (m, 2H).
^{13}C NMR (101 MHz, $CDCl_3$) δ 146.1, 136.2, 132.8, 132.0, 130.9, 127.9, 127.8, 127.5, 127.3, 127.1, 126.6, 124.8, 122.9, 120.8, 120.1, 119.7, 118.6 (q, *J* = 320.6 Hz), 111.6, 111.2.
^{19}F NMR (376 MHz, $CDCl_3$) δ -74.01.

HRMS (ESI): calculated for $C_{19}H_{13}F_3NO_3S$ $[M+H]^+$ m/z: 392.0563, found: 392.0573.

m.p. 126-127 °C

1-(1*H*-Indol-3-yl)naphthalen-2-yl methanesulfonate (1t)

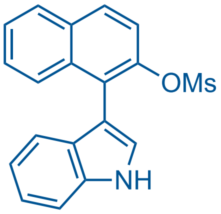

This compound was synthesized based on the general procedure **A** using 1*H*-indole (1.17 g, 10.0 mmol) and Ms_2O. Purification by flash column chromatography (hexane/ethyl acetate = 10/1) provided the title compound as a white solid (2.00 g, 59% yield).

^{1}H NMR (300 MHz, $CDCl_3$) δ 8.57 (brs, 1H), 7.99 – 7.81 (m, 3H), 7.65 (d, *J* = 9.0 Hz, 1H), 7.58 – 7.35 (m, 4H), 7.32 – 7.21 (m, 2H), 7.11 (ddd, *J* = 7.8, 6.9, 1.0 Hz, 1H), 2.35 (s, 3H).

^{13}C NMR (75 MHz, $CDCl_3$) δ 145.6, 135.9, 133.9, 132.6, 129.3, 128.3, 127.7, 127.2, 126.9, 126.4, 125.5, 124.8, 122.8, 122.2, 120.7, 120.4, 111.6, 109.2, 37.8.
HRMS (ESI): calculated for $C_{19}H_{16}NO_3S$ $[M+H]^+$ m/z: 338.0845, found: 338.0847.

m.p. 170-171 °C

2-(1*H*-Indol-3-yl)phenyl trifluoromethanesulfonate (1u)

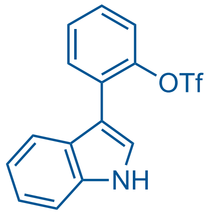

This compound was synthesized based on the general procedure **C** using (2-methoxyphenyl)boronic acid (1.67 g, 11.0 mmol). Purification by flash column chromatography (hexane/ethyl acetate = 20/1) provided the title compound as a white solid (1.63 g, 48% yield).

^{1}H NMR (400 MHz, $CDCl_3$) δ 8.35 (brs, 1H), 7.76 (dd, J = 7.7, 1.8 Hz, 1H), 7.75 – 7.70 (m, 1H), 7.51 – 7.34 (m, 5H), 7.29 (ddd, J = 8.2, 7.0, 1.3 Hz, 1H), 7.23 (ddd, J = 8.1, 7.0, 1.1 Hz, 1H).
^{13}C NMR (101 MHz, $CDCl_3$) δ 147.4, 136.2, 132.1, 129.3, 128.6, 127.8, 126.3, 124.6, 122.8, 122.2, 120.7, 119.6, 118.6 (q, J = 320.5 Hz), 111.6, 111.0.
^{19}F NMR (376 MHz, $CDCl_3$) δ -74.21.

HRMS (ESI): calculated for $C_{15}H_{11}F_3NO_3S$ $[M+H]^+$ m/z: 342.0406, found: 342.0402.

m.p. 78-79 °C

2-(1*H*-Indol-3-yl)-4-methylphenyl trifluoromethanesulfonate (1v)

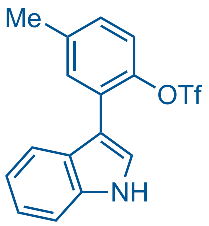

This compound was synthesized based on the general procedure **C** using (2-methoxy-5-methylphenyl)boronic acid (1.83 g, 11.0 mmol). Purification by flash column chromatography (hexane/ethyl acetate = 20/1) provided the title compound as a white solid (1.59 g, 45% yield).

^{1}H NMR (400 MHz, $CDCl_3$) δ 8.31 (brs, 1H), 7.81 – 7.74 (m, 1H), 7.61 – 7.55 (m, 1H), 7.46 – 7.38 (m, 2H), 7.37 – 7.14 (m, 4H), 2.47 (s, 3H).
^{13}C NMR (101 MHz, $CDCl_3$) δ 145.4, 138.6, 136.2, 132.4, 128.9, 128.4, 126.3, 124.5, 122.7, 121.8, 120.6, 119.6, 118.6 (q, J = 320.6 Hz), 111.6, 111.0, 21.0.
^{19}F NMR (376 MHz, $CDCl_3$) δ -74.17.

HRMS (ESI): calculated for $C_{16}H_{13}F_3NO_3S$ $[M+H]^+$ m/z: 356.0563, found: 356.0562.

m.p. 77-78 °C

4-(*tert*-Butyl)-2-(1*H*-indol-3-yl)phenyl trifluoromethanesulfonate (1w)

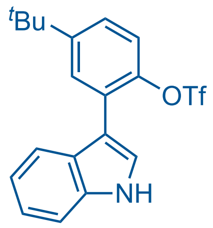

This compound was synthesized based on the general procedure **C** using (5-(*tert*-butyl)-2-methoxyphenyl)boronic acid (2.29 g, 11 mmol),. Purification by flash column chromatography (hexane/ethyl acetate = 20/1) provided the title compound as a white solid (0.95 g, 24% yield).

^{1}H NMR (400 MHz, $CDCl_3$) δ 8.38 (brs, 1H), 7.79 – 7.75 (m, 1H), 7.75 – 7.67 (m, 1H), 7.50 – 7.42 (m, 2H), 7.41 – 7.19 (m, 4H), 1.39 (s, 9H).
^{13}C NMR (101 MHz, $CDCl_3$) δ 151.7, 145.2, 136.2, 129.2, 128.4, 126.5, 124.8, 124.4, 122.8, 121.6, 120.8, 119.6, 118.6 (q, J = 320.7 Hz), 111.6, 111.5, 34.9, 31.5.
^{19}F NMR (376 MHz, $CDCl_3$) δ -74.27.

HRMS (ESI): calculated for $C_{19}H_{19}F_3NO_3S$ $[M+H]^+$ m/z: 398.1032, found: 398.1024.

m.p. 123-124 °C

Methyl 3-(1*H*-indol-3-yl)-4-(((trifluoromethyl)sulfonyl)oxy)benzoate (1x)

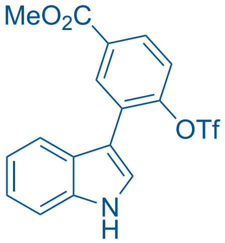

This compound was synthesized based on the general procedure **C*** using (2-hydroxy-5-(methoxycarbonyl)phenyl)boronic acid (0.98 g, 5.0 mmol). Purification by flash column chromatography (hexane/ethyl acetate = 5/1) provided the title compound as a white solid (1.54 g, 77% yield).

^{1}H NMR (500 MHz, $CDCl_3$) δ 8.55 (s, 1H), 8.13 (dd, *J* = 8.1, 1.7 Hz, 1H), 8.08 (d, *J* = 1.6 Hz, 1H), 7.86 (d, *J* = 8.1 Hz, 1H), 7.75 (d, *J* = 8.0 Hz, 1H), 7.56 (dd, *J* = 2.8, 1.1 Hz, 1H), 7.46 (d, *J* = 8.1 Hz, 1H), 7.30 (t, *J* = 7.6 Hz, 1H), 7.26 – 7.21 (m, 1H), 3.98 (s, 3H).

^{13}C NMR (126 MHz, $CDCl_3$) δ 165.6, 146.9, 136.3, 134.3, 131.5, 129.6, 129.4, 125.9, 125.5, 123.5, 123.2, 121.2, 119.5, 118.5 (q, *J* = 321.3 Hz), 111.8, 110.3, 52.7.

^{19}F NMR (471 MHz, $CDCl_3$) δ -73.93.

HRMS: (ESI) calculated for $C_{17}H_{13}F_3NO_5S$ $[M+H]^+$ m/z: 400.0461, found: 400.0455.

m.p. 143-144 °C

4-cyano-2-(1*H*-indol-3-yl)phenyl trifluoromethanesulfonate (1y)

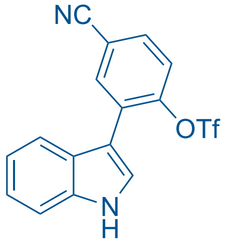

This compound was synthesized based on the general procedure **C*** using (5-cyano-2-hydroxyphenyl)boronic acid (0.82 g, 5.0 mmol). Purification by flash column chromatography (hexane/ethyl acetate = 10/1) provided the title compound as a white solid (1.25 g, 68% yield).

^{1}H NMR (500 MHz, $CDCl_3$) δ 8.59 (s, 1H), 7.92 (d, *J* = 8.1 Hz, 1H), 7.77 – 7.67 (m, 3H), 7.59 (d, *J* = 2.8 Hz, 1H), 7.49 (dt, *J* = 8.2, 0.9 Hz, 1H), 7.32 (ddd, *J* = 8.2, 7.1, 1.2 Hz, 1H), 7.26 (ddd, *J* = 8.1, 7.1, 1.1 Hz, 1H).

^{13}C NMR (126 MHz, $CDCl_3$) δ 146.6, 136.3, 135.0, 132.2, 132.1, 126.1, 125.9, 125.6, 123.5, 121.6, 119.3, 118.5 (q, *J* = 321.3 Hz), 117.5, 112.0, 110.7, 109.6.

^{19}F NMR (471 MHz, $CDCl_3$) δ -73.76.

HRMS: (ESI) calculated for $C_{16}H_9F_3N_2NaO_3S$ $[M+Na]^+$ m/z: 389.0178, found: 389.0182.

m.p. 152-153 °C

2-(1*H*-indol-3-yl)-4-(trifluoromethyl)phenyl trifluoromethanesulfonate (1z)

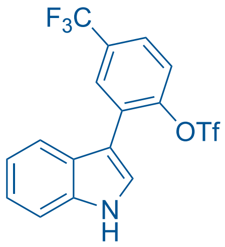

This compound was synthesized based on the general procedure **C*** using (2-hydroxy-5-(trifluoromethyl)phenyl)boronic acid (1.03 g, 5.0 mmol). Purification by flash column chromatography (hexane/ethyl acetate = 15/1) provided the title compound as a white solid (1.39 g, 68% yield).

^{1}H NMR (500 MHz, DMSO) δ 8.01 (d, *J* = 7.9 Hz, 1H), 7.96 – 7.88 (m, 2H), 7.78 (d, *J* = 2.7 Hz, 1H), 7.64 (d, *J* = 8.0 Hz, 1H), 7.51 (dt, *J* = 8.1, 0.9 Hz, 1H), 7.21 (ddd, *J* = 8.2, 7.0, 1.2 Hz, 1H), 7.13 (ddd, *J* = 8.1, 7.0, 1.1 Hz, 1H).

^{13}C NMR (101 MHz, DMSO) δ 146.1, 136.4, 133.8, 132.4, 127.4 (q, *J* = 33.3 Hz), 127.0, 125.9, 125.2, 123.3 (q, *J* = 272.7 Hz), 122.1, 120.3, 119.8, 118.7, 117.8 (q, *J* = 322.2 Hz), 112.2, 107.8.

^{19}F NMR (471 MHz, DMSO) δ -60.93, -74.07.

HRMS: (ESI) calculated for $C_{16}H_9F_6NNaO_3S$ [M+Na]$^+$ m/z: 432.0100, found: 432.0104.

m.p. 160-161 °C

5-Fluoro-2-(1*H*-indol-3-yl)-4-isopropylphenyl trifluoromethanesulfonate (1aa)

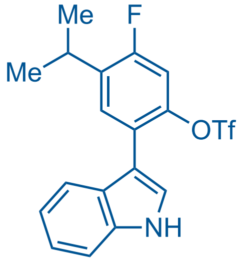

This compound was synthesized based on the general procedure **C** using (4-fluoro-5-isopropyl-2-methoxyphenyl)boronic acid (2.33 g, 11.0 mmol). Purification by flash column chromatography (hexane/ethyl acetate = 20/1) provided the title compound as a white solid (640 mg, 16% yield).

^{1}H NMR (400 MHz, $CDCl_3$) δ 8.37 (brs, 1H), 7.66 – 7.62 (m, 1H), 7.60 (d, *J* = 8.2 Hz, 1H), 7.48 – 7.44 (m, 1H), 7.42 (d, *J* = 2.6 Hz, 1H), 7.33 – 7.25 (m, 1H), 7.25 – 7.17 (m, 1H), 7.17 – 7.08 (m, 1H), 3.31 (hept, *J* = 7.0 Hz, 1H), 1.32 (d, *J* = 6.6 Hz, 6H).

^{13}C NMR (101 MHz, $CDCl_3$) δ 158.6 (d, *J* = 248.9 Hz), 144.8 (d, *J* = 10.8 Hz), 136.2, 136.1 (d, *J* = 14.9 Hz), 130.2 (d, *J* = 6.2 Hz), 126.4, 125.1 (d, *J* = 4.0 Hz), 124.3, 122.9, 120.8, 119.4, 118.5 (d, *J* = 320.6 Hz), 111.6, 110.6, 110.0 (d, *J* = 27.7 Hz), 27.3 (d, *J* = 1.6 Hz), 22.7.

^{19}F NMR (376 MHz, $CDCl_3$) δ -74.15, -117.44.

HRMS (ESI): calculated for $C_{18}H_{16}F_4NO_3S$ [M+H]$^+$ m/z: 402.0782, found: 402.0769.

m.p. 90-91 °C

2-(1*H*-Indol-3-yl)-3,5-dimethylphenyl trifluoromethanesulfonate (1ab)

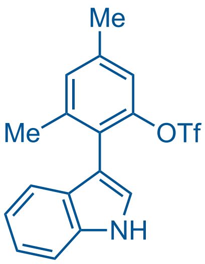

This compound was synthesized based on the general procedure **C** using (2-methoxy-4,6-dimethylphenyl)boronic acid (1.98 g, 11.0 mmol). Purification by flash column chromatography (hexane/ethyl acetate = 20/1) provided the title compound as a yellow oil (920 mg, 25% yield).

^{1}H NMR (400 MHz, $CDCl_3$) δ 8.32 (s, 1H), 7.44 (dt, *J* = 8.1, 0.9 Hz, 1H), 7.32 – 7.23 (m, 2H), 7.22 – 7.18 (m, 2H), 7.15 (ddd, *J* = 8.0, 7.0, 1.0 Hz, 1H), 7.06 (s, 1H), 2.44 (s, 3H), 2.20 (s, 3H).
^{13}C NMR (101 MHz, $CDCl_3$) δ 148.9, 141.4, 138.9, 136.0, 130.8, 127.2, 125.7, 124.4, 122.4, 120.2, 119.8, 119.3, 118.5 (q, *J* = 320.3 Hz), 111.4, 109.5, 21.2, 20.7.
^{19}F NMR (376 MHz, $CDCl_3$) δ -74.61.

HRMS (ESI): calculated for $C_{17}H_{15}F_3NO_3S$ $[M+H]^+$ m/z: 370.0719, found: 370.0713.

2-(5-((*N*-butylacetamido)methyl)-1*H*-indol-3-yl)phenyl trifluoromethanesulfonate (1ac)

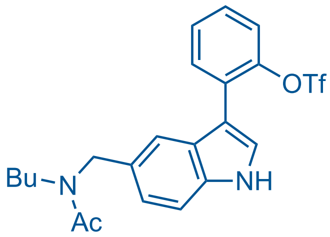

This compound was synthetized following the synthetic route described above:

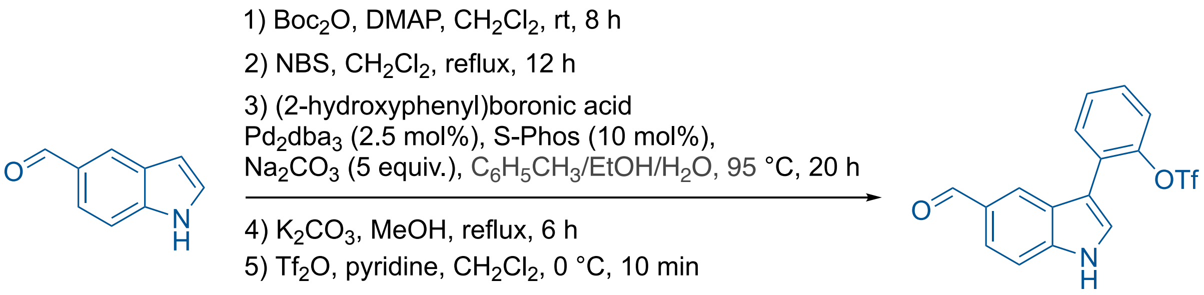

1) To a 100 mL round-bottom flask equipped with a stirring bar were added 1*H*-indole-5-carbaldehyde (1.45 g, 10 mmol, 1.0 equiv.), Boc_2O (2.40 g, 11 mmol, 1.1 equiv.), 4-dimethylaminopyridine (122 mg, 1 mmol, 0.1 equiv.) and dichloromethane (30 mL). The reaction was stirred at room temperature for 8 hours. The reaction was quenched with water (50 mL) and extracted with dichloromethane (3 x 15 mL). The combined organic layer was dried over anhydrous Na_2SO_4 and the solvent was removed under *vacuum*. The crude compound was used directly for the next step without purification.
2) To a 100 mL round-bottom flask equipped with a stirring bar were added the corresponding *tert*-butyl 5-formyl-1*H*-indole-1-carboxylate (10 mmol, 1 equiv.), dichloromethane (30 mL) and *N*-bromosuccinimide (NBS, 1.96 g, 11 mmol, 1.1 equiv,). The reaction mixture was stirred at reflux for 12 hours. Then, the reaction was quenched with water (50 mL) and the mixture was extracted with dichloromethane (3 x 15 mL). The combined organic layer was dried over anhydrous Na_2SO_4 and the solvent was removed under *vacuum*. The crude compound was purified by flash column chromatography on silica gel to afford the corresponding *tert*-butyl 3-bromo-5-formyl-1*H*-indole-1-carboxylate.
3) To a 250 mL two-neck round-bottom flask equipped with a stirring bar were added the mixture of *tert*-butyl 3-bromo-5-formyl-1*H*-indole-1-carboxylate (2.92 g, 9 mmol, 1.0 equiv.), (2-hydroxyphenyl)boronic acid (1.24 g, 9.9 mmol, 1.1 equiv.), Pd_2dba_3 (206 mg, 2.5 mol%), *S*-Phos (370 mg, 10 mol%) and Na_2CO_3 (4.77 g, 50 mmol, 5.0 equiv.). The flask was sealed before being evacuated and backfilled with argon three times. A mix of toluene (60 mL), ethanol (20 mL) and water (20 mL) were added, and the reaction mixture was stirred at 95 ℃ for 20 hours under argon. After completion monitored by TLC, the reaction mixture was cooled to room temperature, quenched with water (150 mL) and extracted with ethyl acetate (3 x 50 mL). The combined organic layer was washed with brine and dried over anhydrous Na_2SO_4. The solvent was removed under *vacuum* and the crude was purified by flash column chromatography on silica gel to afford the corresponding *tert*-butyl 5-formyl-3-(2-hydroxyphenyl)-1*H*-indole-1-carboxylate.
4) To a 100 mL round-bottom flask equipped with a stirring bar were added *tert*-butyl 5-formyl-3-(2-hydroxyphenyl)-1*H*-indole-1-carboxylate (2.64 g, 7.83 mmol, 1.0 equiv.), K_2CO_3 (3.24 g, 23.5 mmol, 3.0 equiv.) and methanol (40 mL). The reaction was heated at reflux for 6 hours. Then, the reaction mixture was cooled to room temperature, diluted with

water (50 mL) and extracted with dichloromethane (3 x 20 mL). The combined organic layer was dried over anhydrous Na_2SO_4 and the solvent was removed under *vacuum.* The crude was used directly for the next step without purification.

5) To a 100 mL oven-dried round-bottom flask equipped with a stirring bar was added 3-(2-hydroxyphenyl)-1*H*-indole-5-carbaldehyde (1.0 equiv.). The flask was sealed before being evacuated and backfilled with argon three times. Pyridine (2.0 equiv.) and anhydrous dichloromethane (30 mL) were added. The reaction mixture was cooled to 0 °C and Tf_2O (1.5 equiv.) was added. Stirring was continued for 10 minutes at 0 °C. Then, the reaction mixture was quenched with water (50 mL) and extracted with dichloromethane (3 x 20 mL). The combined organic layer was dried over anhydrous Na_2SO_4 and the solvent was removed under *vacuum*. The crude was purified by flash column chromatography on silica gel (hexane/ethyl acetate = 8/1) to afford the corresponding 2-(5-formyl-1*H*-indol-3-yl)phenyl trifluoromethanesulfonate as a white solid (2.77 g, 75% yield).

^{1}H NMR (500 MHz, $CDCl_3$) δ 10.05 (s, 1H), 9.00 (s, 1H), 8.21 (dd, *J* = 1.5, 0.7 Hz, 1H), 7.84 (dd, *J* = 8.5, 1.5 Hz, 1H), 7.75 – 7.68 (m, 1H), 7.58 – 7.47 (m, 3H), 7.46 – 7.41 (m, 2H).

^{13}C NMR (126 MHz, $CDCl_3$) δ 192.7, 147.4, 139.7, 132.1, 130.5, 128.9, 128.7, 128.3, 126.4, 126.3, 124.9, 123.3, 122.4, 118.5 (q, *J* = 321.3 Hz), 112.9, 112.4.

^{19}F NMR (471 MHz, $CDCl_3$) δ -74.05.

HRMS: (ESI) calculated for $C_{16}H_{10}F_3NNaO_4S$ $[M+Na]^+$ m/z: 392.0175, found: 392.0186.

m.p. 116-117 °C

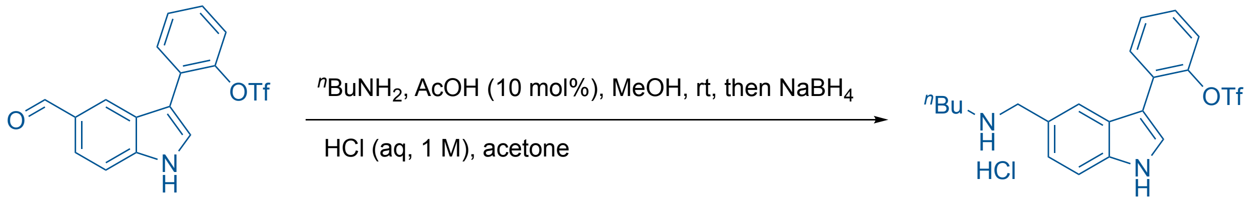

To a 100 mL round-bottom flask equipped with a stirring bar were added 2-(5-formyl-1*H*-indol-3-yl)phenyl trifluoromethanesulfonate (1.85 g, 5 mmol, 1.0 equiv.), butan-1-amine (1.10 g, 15 mmol, 3.0 equiv.), acetic acid (30 mg, 2 drops) and methanol (20 mL). The reaction was stirred at room temperature for 10 hours. After the reaction has been completed, $NaBH_4$ (378 mg, 10 mmol, 2

equiv.) was added with portions to the reaction mixture. Then, the reaction mixture was diluted with water (50 mL) and extracted with dichloromethane (3 x 20 mL). The combined organic layer was dried over anhydrous Na_2SO_4 and the solvent was removed under *vacuum.* The crude was dissolved in acetone (10 mL) and 25 mL HCl (1 M) aqueous solution was added to the solution. The obtained white solid was filtered and dried to the corresponding compound as a white solid (1.99 g, 86% yield).

^{1}H NMR (500 MHz, DMSO) δ 11.86 (s, 1H), 9.29 (s, 2H), 7.91 – 7.79 (m, 2H), 7.69 (d, J = 2.6 Hz, 1H), 7.62 – 7.45 (m, 4H), 7.40 (d, J = 8.5 Hz, 1H), 4.18 (s, 2H), 2.90 – 2.74 (m, 2H), 1.63 (p, J = 7.7 Hz, 2H), 1.28 (h, J = 7.4 Hz, 2H), 0.84 (t, J = 7.4 Hz, 3H).

^{13}C NMR (126 MHz, DMSO) δ 146.7, 136.3, 131.8, 129.2, 128.9, 127.9, 126.8, 125.5, 123.8, 123.1, 122.2, 121.2, 117.9 (q, J = 321.3 Hz), 112.0, 108.9, 50.5, 45.6, 27.3, 19.4, 13.4.

^{19}F NMR (471 MHz, DMSO) δ -74.14.

HRMS: (ESI) calculated for $C_{20}H_{22}F_3N_2O_3S$ $[M-Cl]^+$ m/z: 427.1298, found: 427.1311.

m.p. > 200 °C

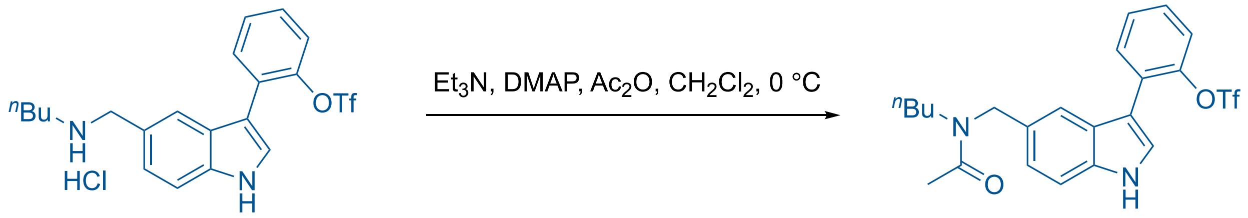

To a 50 mL round-bottom flask equipped with a stirring bar was added 2-(5-((butylamino)methyl)-1*H*-indol-3-yl)phenyl trifluoromethanesulfonate hydrochloride (926 mg, 2 mmol, 1.0 equiv.), triethylamine (607 g, 6 mmol, 3.0 equiv.), 4-dimethylaminopyridine (24 mg, 10 mol%) and CH_2Cl_2 (15 mL). The reaction mixture was cooled to 0 °C and acetic acid anhydride (245 mg, 2.4 mmol, 1.2 equiv.) was added. Stirring was continued for 10 minutes at 0 °C. Then, the reaction mixture was quenched with water (25 mL) and extracted with dichloromethane (3 x 10 mL). The combined organic layer was dried over anhydrous Na_2SO_4 and the solvent was removed under *vacuum*. The crude was purified by flash column chromatography on silica gel (hexane/ethyl acetate = 3/1) to afford the corresponding 2-(5-((*N*-butylacetamido)methyl)-1*H*-indol-3-yl)phenyl trifluoromethanesulfonate as a white foam (880 mg, 95% yield).

^{1}**H NMR** (500 MHz, $CDCl_3$) δ 9.32 – 8.91 (m, 1H), 7.70 – 7.61 (m, 1H), 7.55 – 7.30 (m, 6H), 7.22 – 6.99 (m, 1H), 4.73 (s, 1H), 4.63 (s, 1H), 3.47 – 3.32 (m, 1H), 3.23 – 3.11 (m, 1H), 2.18 (d, *J* = 10.7 Hz, 3H), 1.61 – 1.47 (m, 2H), 1.33 – 1.22 (m, 2H), 0.93 – 0.83 (m, 3H). Messy due to amide effect.

^{13}**C NMR** (126 MHz, $CDCl_3$) δ 171.3, 170.8, 147.5, 147.5, 135.8, 135.7, 132.0, 131.9, 129.9, 129.4, 129.2, 128.9, 128.7, 128.6, 128.0, 127.8, 126.5, 126.2, 125.5, 125.1, 123.2, 122.3, 122.2, 121.1, 119.3, 118.5 (q, *J* = 321.3 Hz), 117.4, 112.2, 111.9, 111.0, 110.9, 52.6, 48.5, 47.3, 45.8, 30.5, 29.8, 22.0, 21.6, 20.3, 20.1, 13.9, 13.8. Messy due to amide effect.

^{19}**F NMR** (471 MHz, $CDCl_3$) δ -74.21.

HRMS: (ESI) calculated for $C_{30}H_{27}F_3N_2NaO_5S$ $[M+Na]^+$ m/z: 607.1485, found: 607.1493.

m.p. 138-139 °C

3-(1*H*-Indol-3-yl)pyridin-2-yl trifluoromethanesulfonate (1ad)

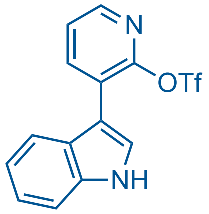

This compound was synthesized based on the general procedure **C** using (2-methoxypyridin-3-yl)boronic acid (1.68 g, 11.0 mmol). Purification by flash column chromatography (hexane/ethyl acetate = 5/1) provided the title compound as a white solid (624 mg, 18% yield).

^{1}**H NMR** (400 MHz, $CDCl_3$) δ 8.52 (brs, 1H), 8.28 (dd, *J* = 4.7, 1.9 Hz, 1H), 8.20 (dd, *J* = 7.6, 1.9 Hz, 1H), 7.77 – 7.70 (m, 1H), 7.55 (d, *J* = 2.7 Hz, 1H), 7.51 – 7.42 (m, 2H), 7.34 – 7.27 (m, 1H), 7.27 – 7.20 (m, 1H).

^{13}**C NMR** (101 MHz, $CDCl_3$) δ 153.3, 145.1, 140.9, 136.3, 125.8, 125.1, 124.3, 123.4, 123.2, 121.2, 119.1, 118.6 (d, *J* = 320.5 Hz), 111.9, 108.8.

^{19}**F NMR** (376 MHz, $CDCl_3$) *δ* -73.50.

HRMS (ESI): calculated for $C_{14}H_9F_3N_2NaO_3S$ $[M+Na]^+$ m/z: 365.0178, found: 365.0166.

m.p. 85-86 ℃

3. Rh-catalyzed atroposelective single-carbon insertion: reaction optimization and scope.

Procedure for reaction optimization:

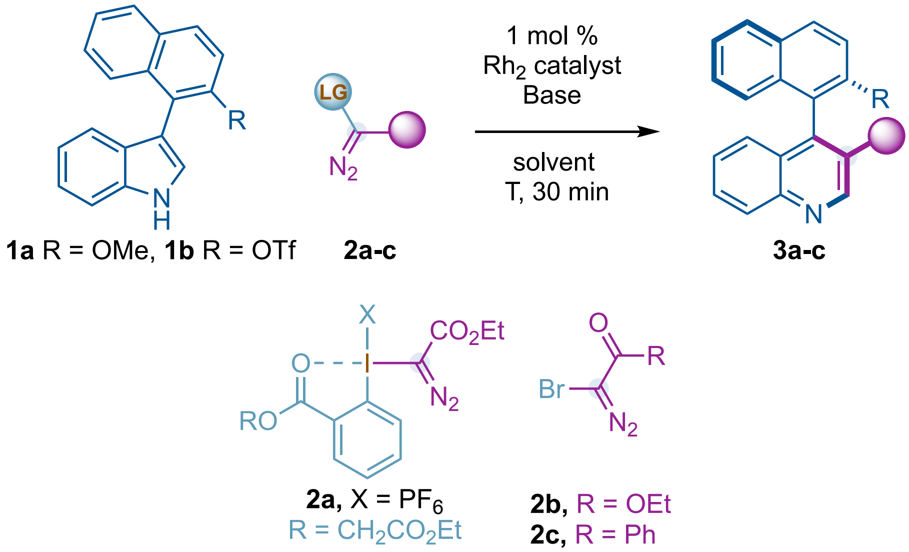

To a 10 mL round-bottom flask tube equipped with a stirring bar were added the corresponding Rh_2 catalyst (0.001 mmol, 1 mol%), **1** (0.1 mmol, 1.0 equiv.), base (0.15 mmol, 1.5 equiv.) and solvent (1 mL). The reaction mixture was placed in the corresponding temperature bath and **2a-c** (0.2 mmol, 2.0 equiv.), dissolved in dichloromethane (1 mL), was added dropwise during 5 minutes using a syringe pump. The reaction was stirred for 25 minutes and the solvent was removed under *vacuum*. The crude was purified by flash column chromatography on silica gel to afford the corresponding compounds **3**.

Note 1: *Compounds* ***2b-c*** *were synthetize using the following protocol and used without further purification:*[8] *To a 10 mL oven-dried round-bottom flask equipped with a stirring bar were added* ***2b-c-H*** *(0.2 mmol, 1.0 equiv.), dichloromethane (1 mL) and 1,8-diazabicyclo[5.4.0]undec-7-ene 23 mg, 0.15 mmol, 0.75 equiv.). The reaction mixture was cooled to 0 ℃ and N-bromosuccinimide (42.7 mg, 0.24 mmol, 1.2 equiv.) was added to the reaction solution in one portion. After 10 minutes stirred at 0 ℃, the* α-diazo bromoketone *was formed as a red brown solution. The solution was used for the next reaction within 10 minutes due to its instability.*

Table S1. Full optimization studies.

Entry	**1**	**2**	Rh catalyst	Solvent	Base	T (°C)	Yield (%)[a]	Ee (%)[b]
1	**1a**	**2a**	$Rh_2(esp)_2$	CH_2Cl_2	-	-50	0	-
2	**1a**	**2b**	$Rh_2(esp)_2$	CH_2Cl_2	-	-50	67	-
3	**1a**	**2b**	Rh_2(*S*-TCPTTL)$_4$	CH_2Cl_2	-	rt	50	15
4	**1a**	**2c**	Rh_2(*S*-TCPTTL)$_4$	CH_2Cl_2	-	rt	36	45
5	**1b**	**2c**	Rh_2(*S*-TCPTTL)$_4$	CH_2Cl_2	-	rt	83	80
6	**1b**	**2c**	Rh_2(*S*-PTTL)$_4$	CH_2Cl_2	-	rt	22	85
7	**1b**	**2c**	Rh_2(*S*-BPTTL)$_4$	CH_2Cl_2	-	rt	26	84
8	**1b**	**2c**	Rh_2(*S*-PTPG)$_4$	CH_2Cl_2	-	rt	10	0
9	**1b**	**2c**	Rh_2(*S*-NTTL)$_4$	CH_2Cl_2	-	rt	81	94
10	**1b**	**2c**	Rh_2(*S*-Br-NTTL)$_4$(AcOEt)$_2$	CH_2Cl_2	-	rt	85	95
11	**1b**	**2c**	Rh_2(*S*-BHTL)$_4$	CH_2Cl_2	-	rt	22	76
12	**1b**	**2c**	Rh_2(*R*-DOSP)$_4$	CH_2Cl_2	-	rt	63	49
13	**1b**	**2c**	Rh_2(*S*-Br-NTTL)$_4$(AcOEt)$_2$	$CH_2Cl_2/ClCH_2CH_2Cl$	-	rt	65	94
14	**1b**	**2c**	Rh_2(*S*-Br-NTTL)$_4$(AcOEt)$_2$	$CH_2Cl_2/CHCl_3$	-	rt	87	91
15	**1b**	**2c**	Rh_2(*S*-Br-NTTL)$_4$(AcOEt)$_2$	CH_2Cl_2/EtOAc	-	rt	53	94
16	**1b**	**2c**	Rh_2(*S*-Br-NTTL)$_4$(AcOEt)$_2$	CH_2Cl_2/PhMe	-	rt	77	95
17	**1b**	**2c**	Rh_2(*S*-Br-NTTL)$_4$(AcOEt)$_2$	CH_2Cl_2/PhCl	-	rt	79	97
18	**1b**	**2c**	Rh_2(*S*-Br-NTTL)$_4$(AcOEt)$_2$	CH_2Cl_2/MeOH	-	rt	0	-
19	**1b**	**2c**	Rh_2(*S*-Br-NTTL)$_4$(AcOEt)$_2$	CH_2Cl_2/THF	-	rt	0	-
20[c]	**1b**	**2c**	Rh_2(*S*-Br-NTTL)$_4$(AcOEt)$_2$	CH_2Cl_2/PhCl	-	rt	45	80

21[c]	**1b**	**2c**	Rh_2(*S*-Br-NTTL)$_4$(AcOEt)$_2$	CH_2Cl_2/PhCl	DIPEA	rt	97	96
22[c]	**1b**	**2c**	Rh_2(*S*-Br-NTTL)$_4$(AcOEt)$_2$	CH_2Cl_2/PhCl	2,6-di-^{t}Bu-pyridine	rt	90	96
23[c]	**1b**	**2c**	Rh_2(*S*-Br-NTTL)$_4$(AcOEt)$_2$	CH_2Cl_2/PhCl	pyridine	rt	32	98
24[c]	**1b**	**2c**	Rh_2(*S*-Br-NTTL)$_4$(AcOEt)$_2$	CH_2Cl_2/PhCl	tetramethyl urea	rt	71	99
25[c]	**1b**	**2c**	Rh_2(*S*-Br-NTTL)$_4$	CH_2Cl_2/PhCl	DIPEA	rt	97	96

[a]Isolated yield. [b] ee values were determined by SFC-MS analysis on a chiral stationary phase. [c] Catalytic amount of 1,8-diazabicyclo[5.4.0]undec-7-ene (3 mg, 0.02 mmol, 10 mol%) was used for the formation of **2b-c**.

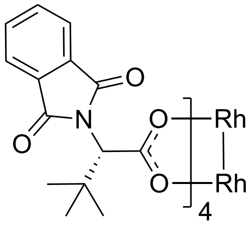

Rh_2(*S*-PTTL)$_4$

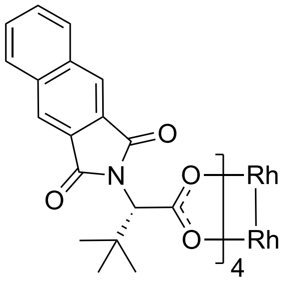

Rh_2(*S*-BPTTL)$_4$

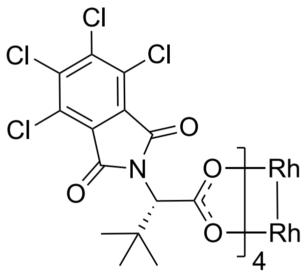

Rh_2(*S*-TCPTTL)$_4$

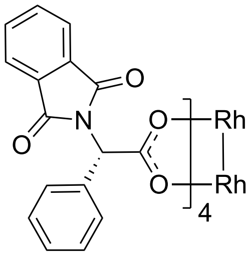

Rh_2(*S*-PTPG)$_4$

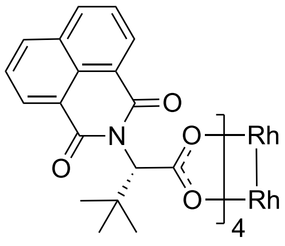

Rh_2(*S*-NTTL)$_4$

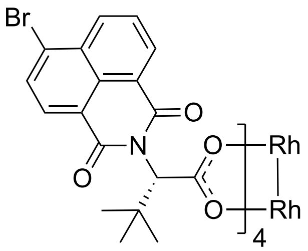

Rh_2(*S*-Br-NTTL)$_4$

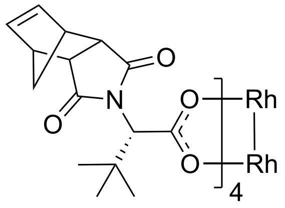

Rh_2(*S*-BHTL)$_4$

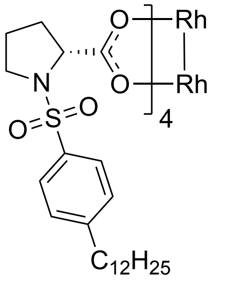

Rh_2(*R*-DOSP)$_4$

Ethyl (*S*)-4-(2-methoxynaphthalen-1-yl)quinoline-3-carboxylate (3a)

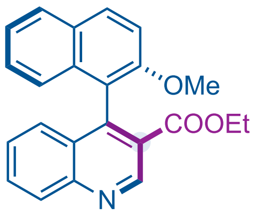

This compound was synthesized following the optimization procedure using **1a** (27.3 mg, 0.1 mmol), **2b-H** (22.8 mg, 0.2 mmol) and Rh_2(*S*-TCPTTL)$_4$ (1.2 mg, 0.001 mmol). Purification by flash column chromatography (hexane/ethyl acetate: 4/1) provided the title compound as colorless oil (17.9 mg, 50% yield, 15% ee). Enantiomeric excess was determined by SFC analysis on a chiral stationary phase (CHIRALPAK IG-3, 1 mL/min, 40% methanol, λ = 230 nm, t_r(major) = 2.380 min, t_r(minor) = 1.804 min).

$[\alpha]_D^{23}$ = -0.8 (c = 0.19, $CHCl_3$).

^{1}H NMR (400 MHz, $CDCl_3$) δ 9.54 (s, 1H), 8.24 (d, *J* = 8.4 Hz, 1H), 8.02 (d, *J* = 8.8 Hz, 1H), 7.87 (d, *J* = 8.1 Hz, 1H), 7.77 (ddd, *J* = 8.3, 6.1, 2.0 Hz, 1H), 7.42 (d, *J* = 9.1 Hz, 1H), 7.39 – 7.29 (m, 3H), 7.22 (ddd, *J* = 8.3, 6.8, 1.3 Hz, 1H), 6.91 (d, *J* = 8.5 Hz, 1H), 3.94 (qd, *J* = 7.1, 4.1 Hz, 2H), 3.74 (s, 3H), 0.71 (t, *J* = 7.1 Hz, 3H).

^{13}C NMR (101 MHz, $CDCl_3$) δ 166.2, 153.8, 150.9, 149.5, 146.4, 133.2, 131.3, 130.3, 129.8, 128.9, 128.1, 127.7, 127.5, 127.4, 127.0, 124.6, 124.5, 123.9, 119.6, 113.3, 60.9, 56.7, 13.5.

HRMS: (ESI) calculated for $C_{23}H_{20}NO_3$ $[M+H]^+$ m/z: 358.1438, found: 358.1442.

3a

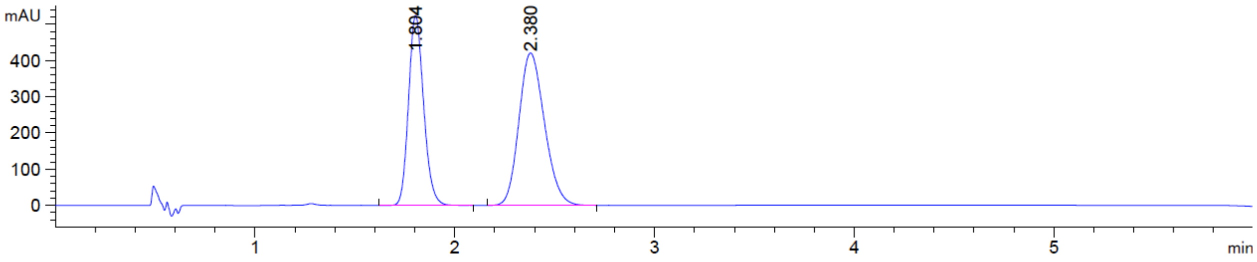

Peak #	RetTime [min]	Type	Width [min]	Area [mAU*s]	Height [mAU]	Area %
1	1.804	VV R	0.0808	1425.45154	271.33752	42.3980
2	2.380	BV R	0.1378	1936.62476	217.56531	57.6020

***Rac*-3a**

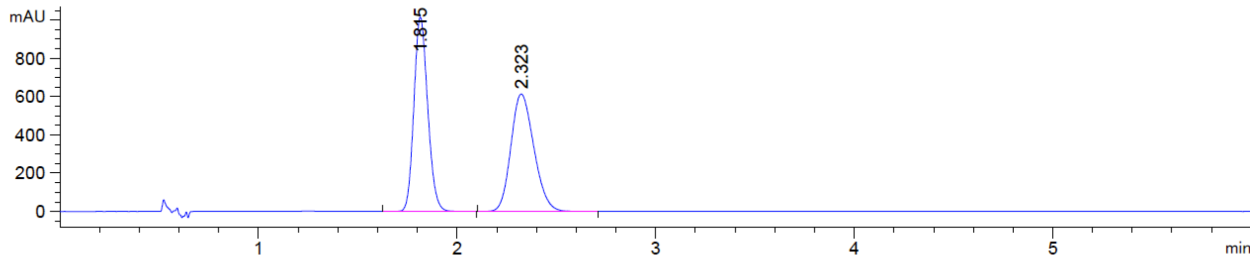

Peak #	RetTime [min]	Type	Width [min]	Area [mAU*s]	Height [mAU]	Area %
1	1.815	VV R	0.0754	4906.84326	1014.56049	49.9168
2	2.323	BV R	0.1247	4923.19727	613.26605	50.0832

(*S*)-(4-(2-Methoxynaphthalen-1-yl)quinolin-3-yl)(phenyl)methanone (3b)

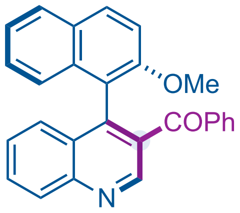

This compound was synthesized following the optimization procedure using **1a** (27.3 mg, 0.1 mmol), **2c-H** (29.2 mg, 0.2 mmol) and Rh_2(*S*-TCPTTL)$_4$ (1.9 mg, 0.001 mmol). Purification by flash column chromatography (hexane/ethyl acetate: 4/1) provided the title compound as colorless oil (14.0 mg, 36% yield, 45% ee). Enantiomeric excess was determined by SFC analysis on a chiral stationary phase (CHIRALPAK ID-3, 1 mL/min, 30% methanol, λ = 210 nm, t_r(major) = 1.800 min, t_r(minor) = 2.031 min).

$[\alpha]_D^{23}$ = +7.2 (c = 0.09, $CHCl_3$).

^{1}H NMR (400 MHz, $CDCl_3$) δ 9.13 (s, 1H), 8.28 (d, *J* = 8.5 Hz, 1H), 7.85 – 7.76 (m, 2H), 7.74 (dd, *J* = 7.0, 2.5 Hz, 1H), 7.50 (dd, *J* = 8.4, 1.3 Hz, 2H), 7.44 – 7.37 (m, 2H), 7.37 – 7.31 (m, 1H), 7.31 – 7.23 (m, 2H), 7.19 – 7.10 (m, 3H), 7.09 – 7.03 (m, 1H), 3.63 (s, 3H).

^{13}C NMR (101 MHz, $CDCl_3$) δ 196.4, 153.9, 149.3, 148.9, 143.8, 137.5, 133.4, 133.3, 132.8, 131.1, 130.8, 129.8, 129.3, 128.6, 128.1, 127.9, 127.7, 127.5, 127.3, 127.2, 125.0, 123.9, 117.3, 112.3, 55.9.

HRMS (ESI): calculated for $C_{27}H_{20}NO_2$ $[M+H]^+$ m/z: 390.1489, found: 390.1492.

3b

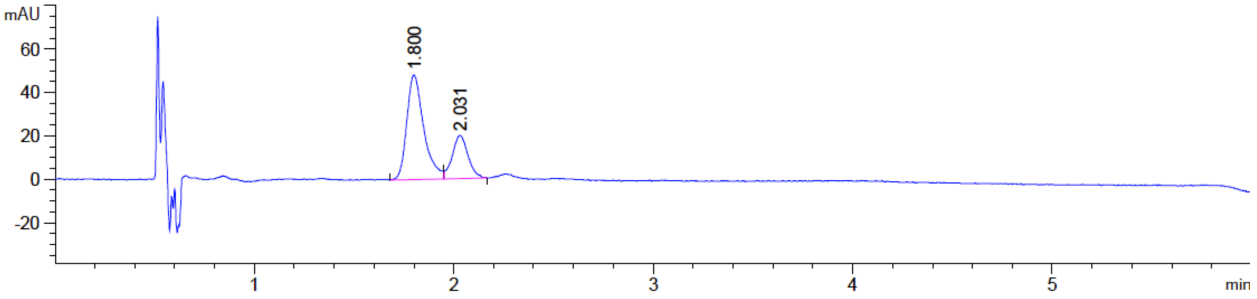

Peak #	RetTime [min]	Type	Width [min]	Area [mAU*s]	Height [mAU]	Area %
1	1.800	BV	0.0881	285.13010	48.16565	72.4072
2	2.031	VB	0.0817	108.65688	19.88856	27.5928

***Rac*-3b**

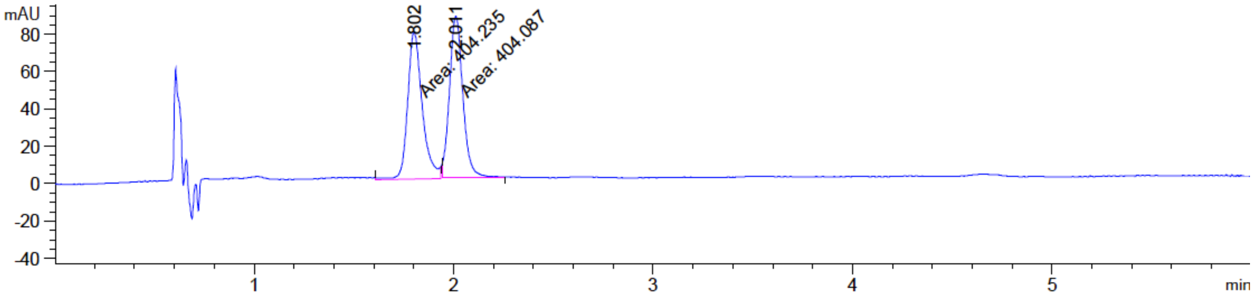

Peak #	RetTime [min]	Type	Width [min]	Area [mAU*s]	Height [mAU]	Area %
1	1.802	MM	0.0856	404.23456	78.68412	50.0091
2	2.011	MM	0.0779	404.08722	86.43556	49.9909

General procedure D: Rh-catalyzed atroposelective single-carbon insertion for the synthesis of 3c-3ah

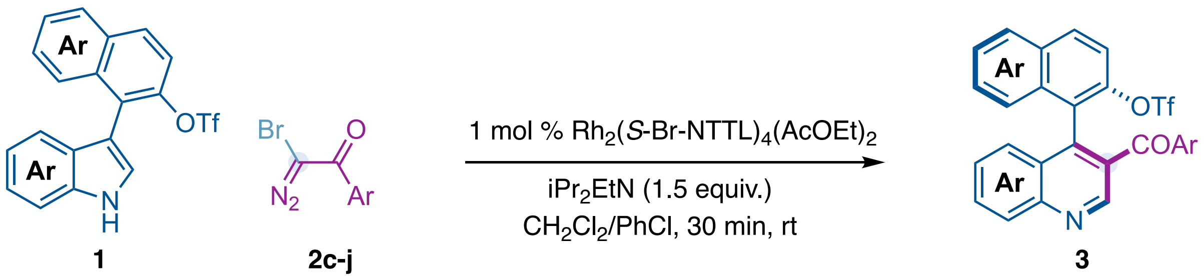

To a 10 mL round-bottom flask tube equipped with a stirring bar were added the corresponding enantiopure dirhodium catalyst Rh_2(*S*-Br-NTTL)$_4$(AcOEt)$_2$ (1.9 mg, 0.001 mmol, 1 mol%), **1** (0.1 mmol, 1.0 equiv.), chlorobenzene (1 mL) and DIPEA (19.4 mg, 0.15 mmol, 1.5 equiv.). The reaction mixture was placed in a room temperature water bath (otherwise noted) and **2c-j** (0.2 mmol, 2.0 equiv.) was added dropwise during 5 minutes using a syringe pump. The reaction was stirred for 25 minutes and the solvent was removed under *vacuum*. The crude was purified by flash column chromatography on silica gel to afford the corresponding chiral compounds **3**. Racemic compounds were prepared using Rh_2(esp)$_2$ as catalyst unless otherwise stated.

Note 1: *Compounds **2c-j** were synthetize using the following protocol and used without further purification: To a 10 mL oven-dried round-bottom flask equipped with a stirring bar were added **2c-j-H** (0.2 mmol, 1.0 equiv.), dichloromethane (1 mL) and 1,8-diazabicyclo[5.4.0]undec-7-ene (3 mg, 0.02 mmol, 10 mol%). The reaction mixture was cooled to 0 °C and N-bromosuccinimide (42.7 mg, 0.24 mmol, 1.2 equiv.) was added to the reaction solution in one portion. After 10 minutes stirred at 0 °C, the* α-diazo bromoketone *was formed as a red brown solution. The solution was used for the next reaction within 10 minutes due to its instability.*

(*S*)-1-(3-Benzoylquinolin-4-yl)naphthalen-2-yl trifluoromethanesulfonate (3c)

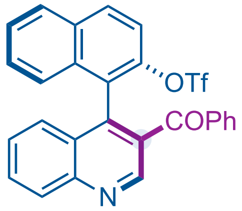

This compound was synthesized following the general procedure **D** using **1b** (39.1 mg, 0.1 mmol) and **2c-H** (29.2 mg, 0.2 mmol). Purification by flash column chromatography (hexane/ethyl acetate: 6/1) provided the title compound as pale yellow foam (49.3 mg, 97% yield, 96% ee). Enantiomeric excess was determined by SFC analysis on a chiral stationary phase (CHIRALPAK IG-3, 1 mL/min, 10% methanol, λ = 210 nm, t_r(major) = 2.700 min, t_r(minor) = 3.394 min).

$[\alpha]_D^{23}$ = +50.7 (c = 0.15, $CHCl_3$).

^{1}H NMR (400 MHz, $CDCl_3$) δ 9.20 (s, 1H), 8.33 (d, *J* = 8.0 Hz, 1H), 7.98 (d, *J* = 9.1 Hz, 1H), 7.92 (d, *J* = 8.3 Hz, 1H), 7.84 (ddd, *J* = 8.4, 6.8, 1.4 Hz, 1H), 7.75 – 7.64 (m, 2H), 7.54 (ddd, *J* = 8.2, 6.8, 1.2 Hz, 1H), 7.52 – 7.38 (m, 4H), 7.38 – 7.30 (m, 3H), 7.27 (d, *J* = 9.2 Hz, 1H).

^{13}C NMR (101 MHz, $CDCl_3$) δ 194.9, 149.4, 148.9, 144.2, 140.6, 136.8, 133.5, 133.2, 132.5, 132.0, 131.7, 131.5, 130.0 (2C), 128.5, 128.4, 128.2, 128.1, 127.4, 127.3, 127.2, 126.7, 126.1, 119.1, 118.1 (q, *J* = 321.2 Hz).

^{19}F NMR (376 MHz, $CDCl_3$) δ -74.63.

HRMS: (ESI) calculated for $C_{27}H_{17}F_3NO_4S$ $[M+H]^+$ m/z: 508.0825, found: 508.0835.

^{1}H-^{1}H COSY, ^{1}H-^{13}C HSQC and ^{1}H-^{13}C HMBC spectra were measured.

3c

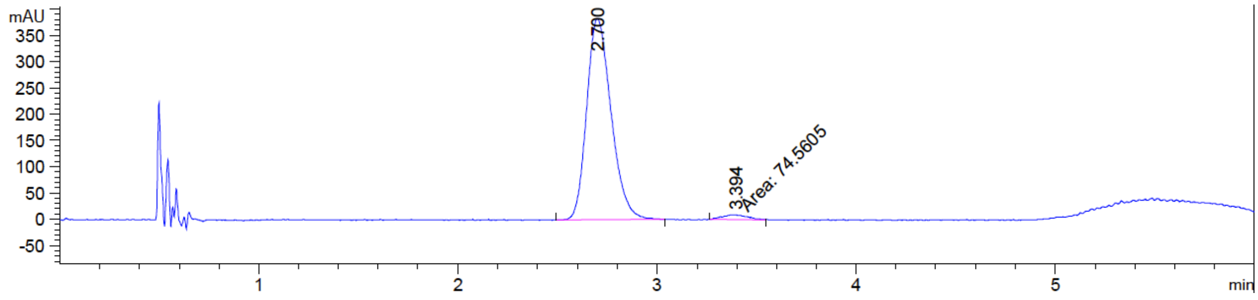

Peak #	RetTime [min]	Type	Width [min]	Area [mAU*s]	Height [mAU]	Area %
1	2.700	VV R	0.1311	3273.28882	383.44690	97.7729
2	3.394	MM	0.1379	74.56048	9.01020	2.2271

***Rac*-3c**

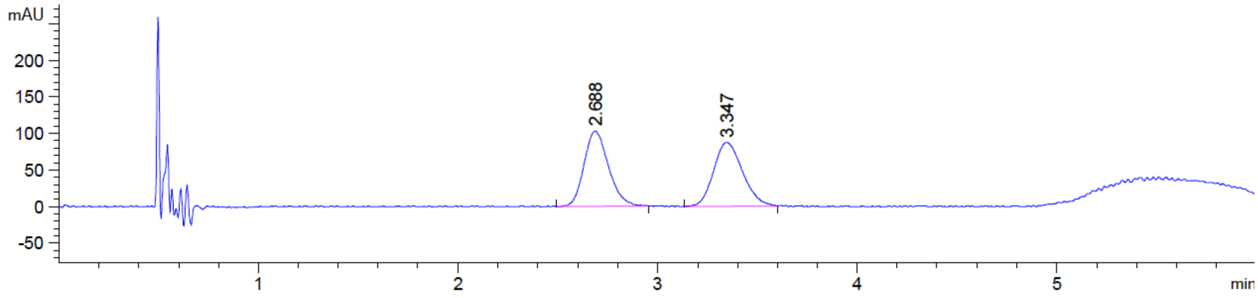

Peak #	RetTime [min]	Type	Width [min]	Area [mAU*s]	Height [mAU]	Area %
1	2.688	VV R	0.1176	886.50787	103.31069	50.1040
2	3.347	VV R	0.1303	882.82758	87.02979	49.8960

Procedure for a 3 mmol-scale reaction with 1b:

To a 100 mL oven-dried round-bottom flask equipped with a stirring bar was charged with **2c-H** (877 mg, 6 mmol, 2.0 equiv.), dichloromethane (20 mL) and 1,8-diazabicyclo[5.4.0]undec-7-ene (91 mg, 0.6 mmol, 20 mol%). The reaction flask was transferred to an ice-water bath and stirred for 5 min. Then *N*-bromosuccinimide (1.28 g, 7.2 mmol, 2.4 equiv.) was added to the reaction solution in one portion. After 10 min stirring at 0 °C, the resulting red brown solution was added dropwise during 20 minutes to a 100 mL oven-dried round-bottom flask which was equipped with Rh_2(*S*-BrNTTL)$_4$(AcOEt)$_2$ (29 mg, 0.015 mmol, 0.5 mol%), **1b** (1.17 g, 3 mmol), DIPEA (582 mg, 4.5 mmol, 0.15 equiv.) and PhCl (10 mL) in a room temperature water bath. The reaction was stirred for 25 min and solvent was removed under *vacuum*. The crude residue was purified by flash column chromatography (hexane/ethyl acetate: 6/1) provided the title compound as pale yellow foam (1.42 g, 93% yield, 96% ee).

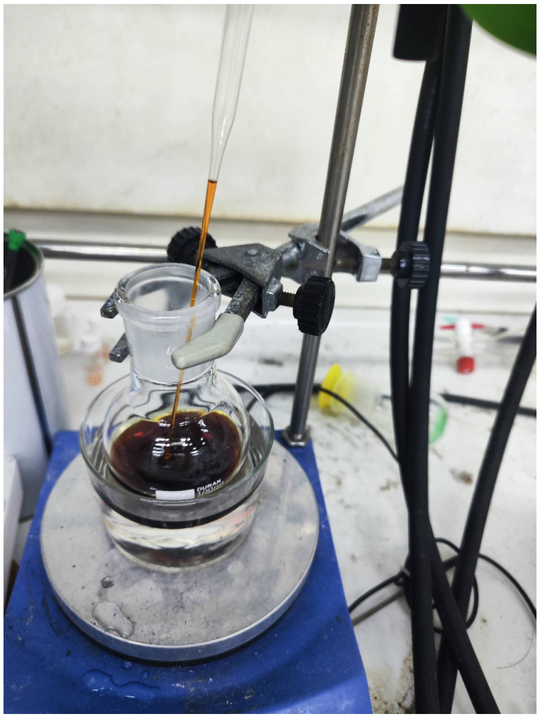

Figure S1. Set-up of the gram-scale reaction.

3c-gram scale

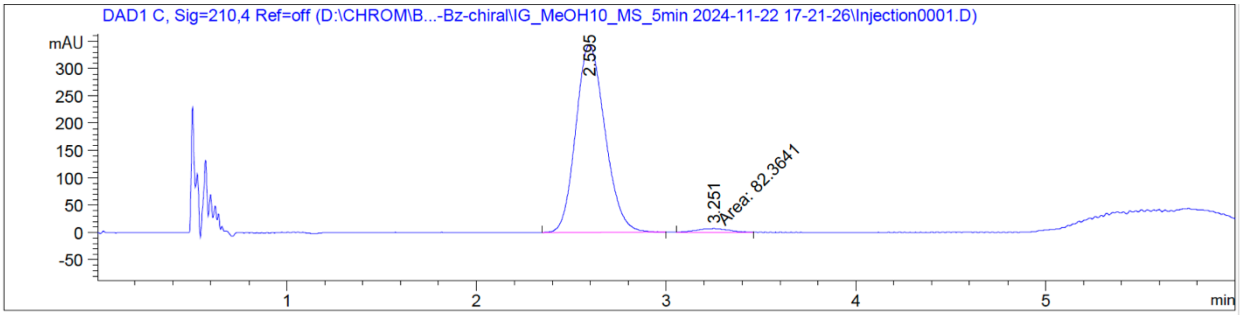

Peak #	RetTime [min]	Type	Width [min]	Area [mAU*s]	Height [mAU]	Area %
1	2.595	BV R	0.1653	3682.86060	342.97891	97.8125
2	3.251	MM	0.1996	82.36405	6.87807	2.1875

***Rac*-3c**

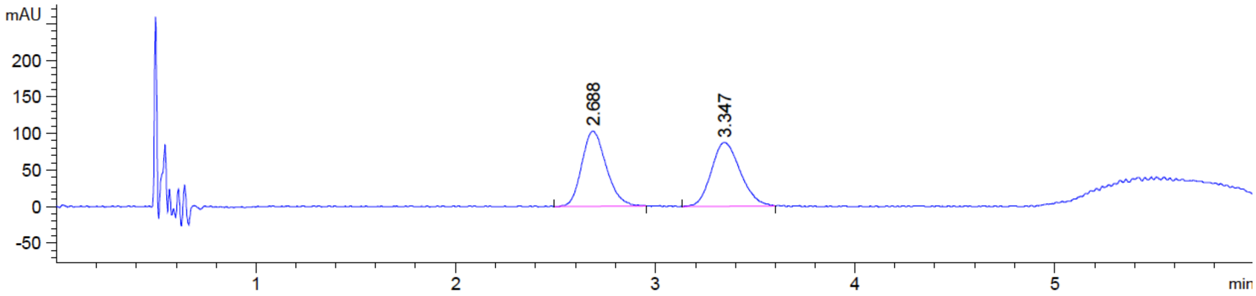

Peak #	RetTime [min]	Type	Width [min]	Area [mAU*s]	Height [mAU]	Area %
1	2.688	VV R	0.1176	886.50787	103.31069	50.1040
2	3.347	VV R	0.1303	882.82758	87.02979	49.8960

Note 1: Control experiments carried out with the corresponding α-diazo chloro- and iodoketone under the optimized reaction conditions gave ***3c*** *in 96% ee albeit in poor yields 6-20% yields.*

Note 2: Using Rh_2(R-Br-NTTL)$_4$ under the optimized reaction conditions gave ***(R)-1-(3-Benzoylquinolin-4-yl)naphthalen-2-yl trifluoromethanesulfonate (ent-3c)*** *in 95% ee and 73% yield.*

$[\alpha]_D^{23}$ = -46.7 (c = 0.125, $CHCl_3$).

Ent-3c

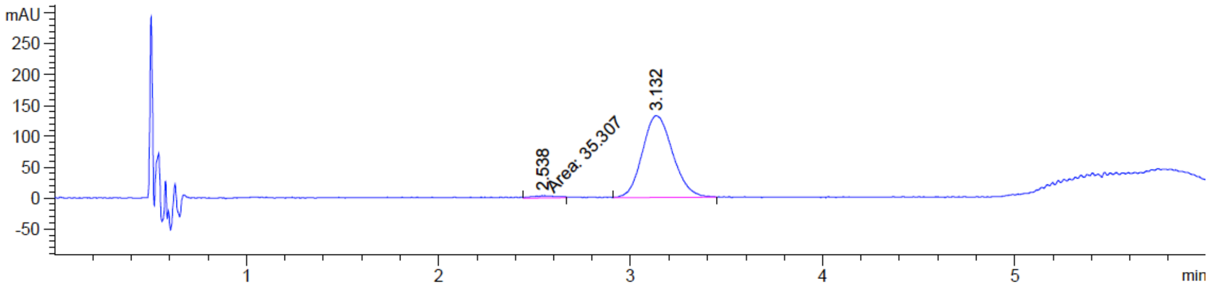

Peak #	RetTime [min]	Type	Width [min]	Area [mAU*s]	Height [mAU]	Area %
1	2.538	MM	0.1563	35.30703	3.76381	2.3605
2	3.132	VV R	0.1477	1460.40894	132.85341	97.6395

(*R*)-1-(3-Benzoyl-5-fluoroquinolin-4-yl)naphthalen-2-yl trifluoromethanesulfonate (3d)

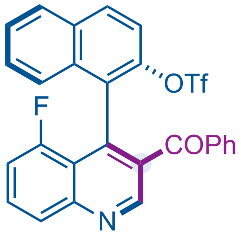

This compound was synthesized following the general procedure **D** using **1c** (40.9 mg, 0.1 mmol) and **2c-H** (29.2 mg, 0.2 mmol). Purification by flash column chromatography (hexane/ethyl acetate: 6/1) provided the title compound as pale yellow oil (32.1 mg, 61% yield, 92% ee). Enantiomeric excess was determined by SFC analysis on a chiral stationary phase (CHIRALPAK ID-3, 1 mL/min, 15% ethanol, λ = 210 nm, t_r(major) = 1.333 min, t_r(minor) = 1.508 min).

$[\alpha]_D^{23}$ = +7.7 (c = 0.14, $CHCl_3$).

^{1}H NMR (400 MHz, $CDCl_3$) δ 9.13 (s, 1H), 8.18 (d, *J* = 8.6 Hz, 1H), 7.88 (t, *J* = 8.3 Hz, 2H), 7.79 (s, 1H), 7.59 (d, *J* = 7.7 Hz, 2H), 7.53 (ddd, *J* = 8.2, 6.8, 1.2 Hz, 1H), 7.50 – 7.39 (m, 2H), 7.37 – 7.26 (m, 4H), 7.15 (dd, *J* = 11.7, 7.9 Hz, 1H).

^{13}C NMR (101 MHz, $CDCl_3$) δ 194.5, 158.7 (d, *J* = 261.2 Hz), 149.8, 149.7, 143.5 (d, *J* = 2.5 Hz), 137.1, 136.5, 134.1, 133.7, 133.3 (d, *J* = 3.9 Hz), 131.7, 131.4, 131.3, 129.8, 128.4, 128.0, 127.6

(d, J = 2.6 Hz), 127.1, 126.5 (d, J = 4.2 Hz), 126.1, 118.6, 118.4 (d, J = 9.7 Hz), 118.3 (q, J = 321.2 Hz), 113.4 (d, J = 21.6 Hz).

^{19}F NMR (376 MHz, CDCl$_3$) δ -74.66, -108.83.

HRMS: (ESI) calculated for $C_{27}H_{15}F_4NNaO_4S$ $[M+Na]^+$ m/z: 548.0550, found: 548.0541.

3d

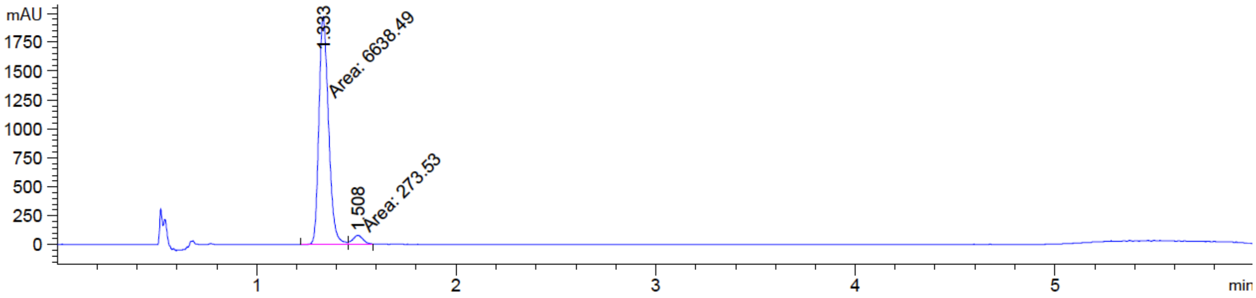

Peak #	RetTime [min]	Type	Width [min]	Area [mAU*s]	Height [mAU]	Area %
1	1.333	MM	0.0561	6638.49023	1970.46704	96.0427
2	1.508	MM	0.0607	273.52972	75.10049	3.9573

***Rac*-3d**

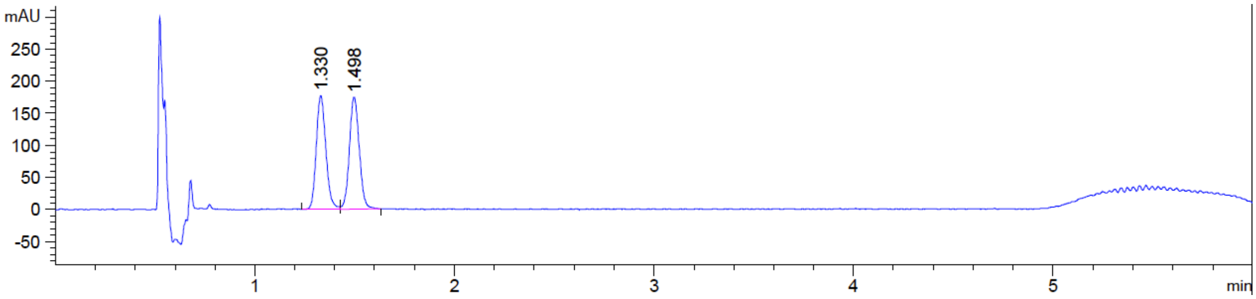

Peak #	RetTime [min]	Type	Width [min]	Area [mAU*s]	Height [mAU]	Area %
1	1.330	BV	0.0516	592.96924	176.75461	49.7060
2	1.498	VV R	0.0536	599.98438	174.53468	50.2940

(*S*)-1-(3-Benzoyl-5-methylquinolin-4-yl)naphthalen-2-yl trifluoromethanesulfonate (3e)

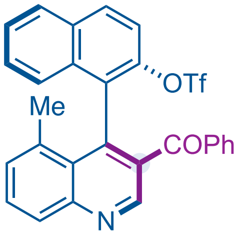

This compound was synthesized following the general procedure **D** at -20 ℃ using **1d** (40.5 mg, 0.1 mmol) and **2c-H** (29.2 mg, 0.2 mmol). Purification by flash column chromatography (hexane/ethyl acetate: 6/1) provided the title compound as pale yellow oil (28.2 mg, 54% yield, 98% ee). Enantiomeric excess was determined by SFC analysis on a chiral stationary phase (CHIRALPAK ID-3, 1 mL/min, 10% methanol, λ = 210 nm, t_r(major) = 2.755 min, t_r(minor) = 3.408 min).

$[\boldsymbol{\alpha}]_{\boldsymbol{D}}^{\mathbf{23}}$ = -4.1 (c = 0.12, $CHCl_3$).

^{1}H NMR (400 MHz, $CDCl_3$) δ 9.04 (s, 1H), 8.23 (d, J = 8.5 Hz, 1H), 7.92 (d, J = 9.1 Hz, 1H), 7.87 (d, J = 8.2 Hz, 1H), 7.73 (t, J = 8.0 Hz, 1H), 7.63 – 7.50 (m, 3H), 7.49 – 7.40 (m, 2H), 7.39 – 7.24 (m, 5H), 1.73 (s, 3H).

^{13}C NMR (101 MHz, $CDCl_3$) δ 195.1, 150.1, 148.0, 144.4, 139.3, 136.8, 136.5, 134.2, 133.7, 133.5, 131.9, 131.6, 131.4, 131.0, 129.8, 129.5, 128.5, 128.41, 128.36, 128.3, 127.4, 126.7, 126.6, 118.8, 118.1 (q, J = 321.2 Hz), 22.8.

^{19}F NMR (376 MHz, $CHCl_3$) δ -74.87.

HRMS: (ESI) calculated for $C_{28}H_{19}F_3NO_4S$ $[M+H]^+$ m/z: 522.0981, found: 522.0993.

3e

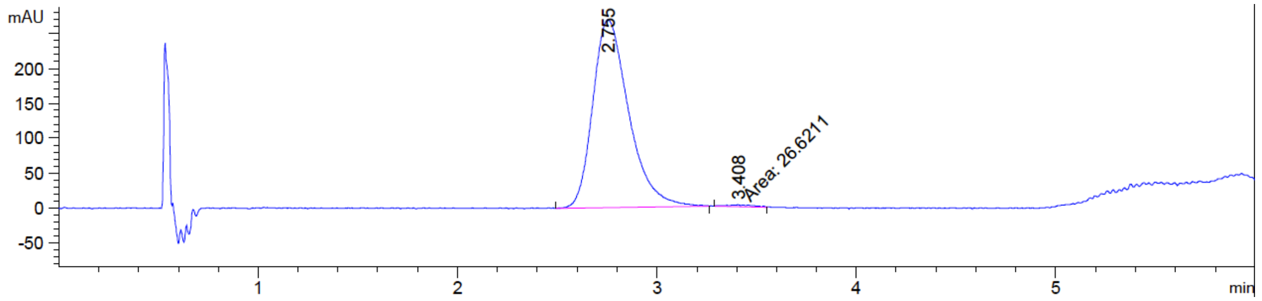

Peak #	RetTime [min]	Type	Width [min]	Area [mAU*s]	Height [mAU]	Area %
1	2.755	VV R	0.1797	3380.58105	269.78323	99.2187
2	3.408	MM	0.1529	26.62114	2.90225	0.7813

***Rac*-3e**

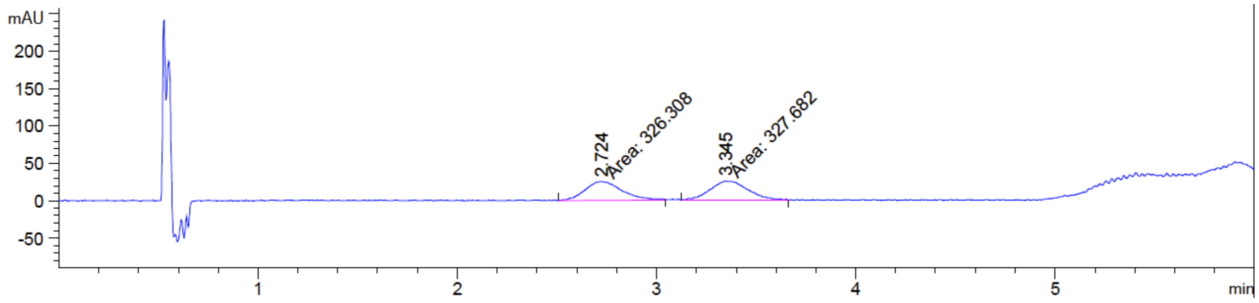

Peak #	RetTime [min]	Type	Width [min]	Area [mAU*s]	Height [mAU]	Area %
1	2.724	MM	0.2142	326.30792	25.38573	49.8949
2	3.345	MM	0.2180	327.68243	25.05690	50.1051

(*S*)-1-(3-Benzoyl-6-fluoroquinolin-4-yl)naphthalen-2-yl trifluoromethanesulfonate (3f)

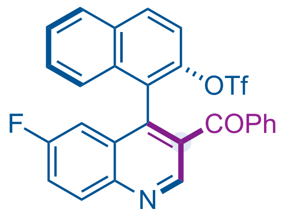

This compound was synthesized following the general procedure **D** using **1e** (40.9 mg, 0.1 mmol) and **2c-H** (29.2 mg, 0.2 mmol). Purification by flash column chromatography (hexane/ethyl acetate: 6/1) provided the title compound as pale yellow oil (43.1 mg, 82% yield, 98% ee). Enantiomeric excess was determined by SFC analysis on a chiral stationary phase (CHIRALPAK IG-3, 1 mL/min, 10% methanol, λ = 210 nm, t_r(major) = 1.727 min, t_r(minor) = 2.001 min).

$[\alpha]_D^{23}$ = +40.4 (c = 0.18, $CHCl_3$).

^{1}H NMR (400 MHz, $CDCl_3$) δ 9.13 (s, 1H), 8.32 (dd, J = 9.3, 5.4 Hz, 1H), 8.00 (d, J = 9.1 Hz, 1H), 7.94 (d, J = 8.5 Hz, 1H), 7.72 – 7.65 (m, 2H), 7.65 – 7.53 (m, 2H), 7.53 – 7.47 (m, 1H), 7.45

(ddd, $J = 8.3, 6.9, 1.3$ Hz, 1H), 7.40 (d, $J = 9.1$ Hz, 1H), 7.37 – 7.29 (m, 2H), 7.23 (dd, $J = 8.5, 1.1$ Hz, 1H), 6.91 (dd, $J = 9.5, 2.8$ Hz, 1H).

^{13}C NMR (101 MHz, $CDCl_3$) δ 194.7, 161.4 (d, $J = 251.0$ Hz), 148.7 (d, $J = 2.8$ Hz), 146.0, 144.2, 140.0 (d, $J = 5.9$ Hz), 136.6, 133.7, 133.2, 132.9, 132.6 (d, $J = 9.3$ Hz), 132.1, 132.0, 130.0, 128.6, 128.51, 128.45, 128.4, 127.5, 126.5, 125.6, 122.0 (d, $J = 26.0$ Hz), 119.1, 118.2 (q, $J = 322.2$ Hz), 110.6 (d, $J = 23.4$ Hz).

^{19}F NMR (376 MHz, $CDCl_3$) δ -74.57, -109.70.

HRMS: (ESI) calculated for $C_{27}H_{16}F_4NO_4S$ $[M+H]^+$ m/z: 526.0731, found: 526.0738.

3f

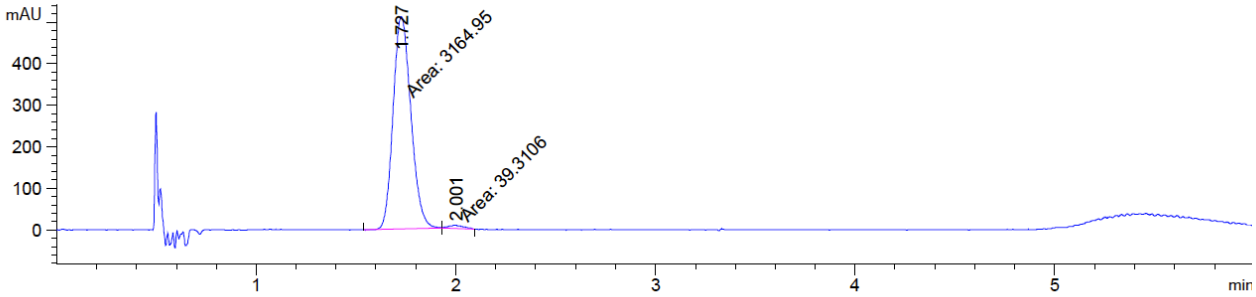

Peak #	RetTime [min]	Type	Width [min]	Area [mAU*s]	Height [mAU]	Area %
1	1.727	MM	0.1031	3164.95190	511.49829	98.7732
2	2.001	MM	0.0883	39.31059	7.42039	1.2268

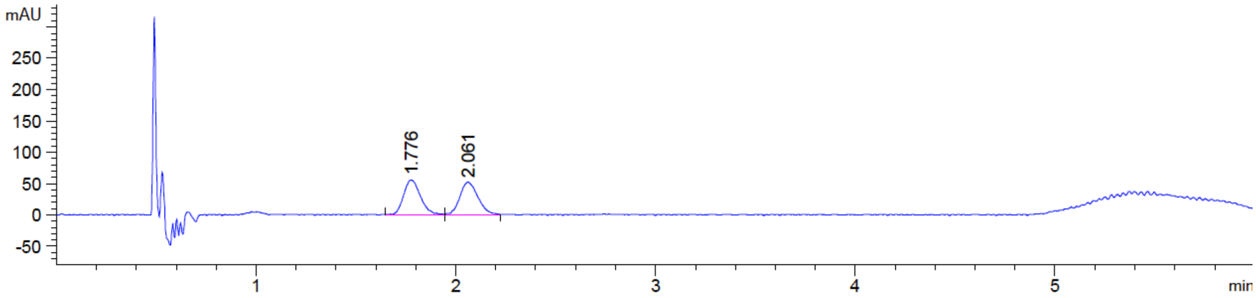

Peak #	RetTime [min]	Type	Width [min]	Area [mAU*s]	Height [mAU]	Area %
1	1.776	VV R	0.0856	317.12079	55.10775	49.7391
2	2.061	BB	0.0763	320.44711	51.70843	50.2609

(*S*)-1-(3-Benzoyl-6-chloroquinolin-4-yl)naphthalen-2-yl trifluoromethanesulfonate (3g)

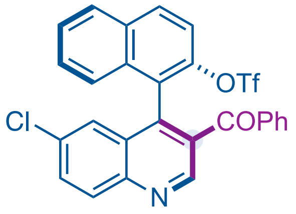

This compound was synthesized following the general procedure **D** using **1f** (42.6 mg, 0.1 mmol) and **2c-H** (29.2 mg, 0.2 mmol). Purification by flash column chromatography (hexane/ethyl acetate: 6/1) provided the title compound as pale yellow oil (45.0 mg, 83% yield, 97% ee). Enantiomeric excess was determined by SFC analysis on a chiral stationary phase (CHIRALPAK ID-3, 1 mL/min, 10% ethanol, λ = 210 nm, t_r(major) = 1.960 min, t_r(minor) = 2.255 min).

$[\alpha]_D^{23}$ = +81.3 (c = 0.13, $CHCl_3$).

^{1}H NMR (400 MHz, $CDCl_3$) δ 9.15 (s, 1H), 8.26 (d, *J* = 9.0 Hz, 1H), 8.00 (d, *J* = 9.1 Hz, 1H), 7.94 (d, *J* = 8.2 Hz, 1H), 7.78 (dd, *J* = 9.1, 2.3 Hz, 1H), 7.71 – 7.62 (m, 2H), 7.57 (ddd, *J* = 8.2, 6.9, 1.2 Hz, 1H), 7.53 – 7.42 (m, 2H), 7.40 (d, *J* = 9.1 Hz, 1H), 7.37 – 7.30 (m, 2H), 7.29 (d, *J* = 2.3 Hz, 1H), 7.23 (d, *J* = 8.5 Hz, 1H).

^{13}C NMR (101 MHz, $CDCl_3$) δ 194.5, 149.5, 147.2, 144.3, 139.8, 136.5, 134.4, 133.7, 133.4, 133.0, 132.6, 132.2, 132.1, 131.6, 130.0, 128.6, 128.5 (2C), 128.1, 127.6, 126.4, 125.9, 125.3, 119.1, 118.2 (q, *J* = 321.2 Hz).

^{19}F NMR (376 MHz, $CHCl_3$) δ -74.54.

HRMS: (ESI) calculated for $C_{27}H_{15}ClF_3NNaO_4S$ $[M+Na]^+$ m/z: 564.0255, found: 564.0257.

3g

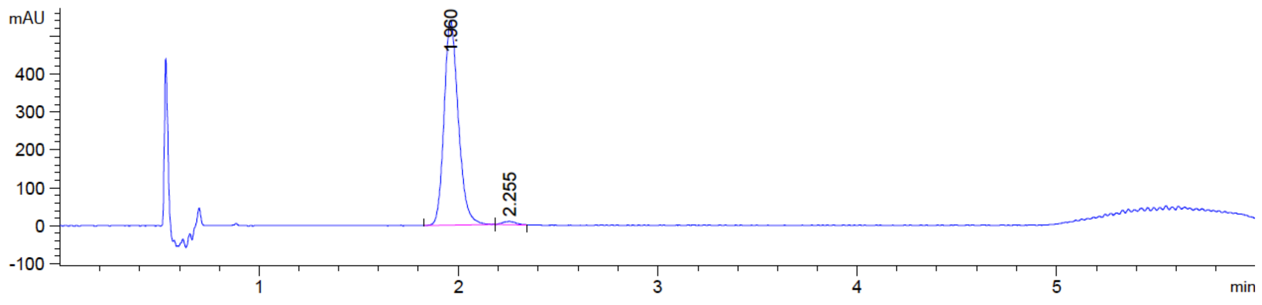

Peak #	RetTime [min]	Type	Width [min]	Area [mAU*s]	Height [mAU]	Area %
1	1.960	BV R	0.0764	2678.91040	539.93036	98.4427
2	2.255	BV R	0.0564	42.37873	9.26009	1.5573

***Rac*-3g**

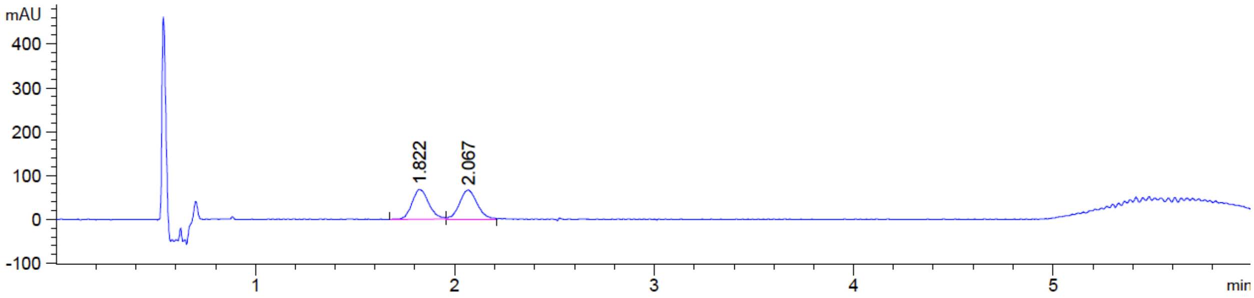

Peak #	RetTime [min]	Type	Width [min]	Area [mAU*s]	Height [mAU]	Area %
1	1.822	VV R	0.0908	404.32889	68.06693	50.0037
2	2.067	VB	0.0896	404.26929	65.91132	49.9963

(*S*)-1-(3-Benzoyl-6-methylquinolin-4-yl)naphthalen-2-yl trifluoromethanesulfonate (3h)

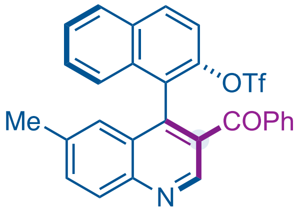

This compound was synthesized following the general procedure **D** using **1g** (40.5 mg, 0.1 mmol) and **2c-H** (29.2 mg, 0.2 mmol). Purification by flash column chromatography (hexane/ethyl acetate: 6/1) provided the title compound as pale yellow oil (48.9 mg, 94% yield, 97% ee). Enantiomeric excess was determined by SFC analysis on a chiral stationary phase (CHIRALPAK ID-3, 1 mL/min, 10% ethanol, λ = 210 nm, t_r(major) = 2.390 min, t_r(minor) = 2.829 min).

$[\alpha]_D^{23}$ = +43.6 (c = 0.12, $CHCl_3$).

^{1}H NMR (400 MHz, $CDCl_3$) δ 9.11 (s, 1H), 8.22 (d, *J* = 8.6 Hz, 1H), 7.98 (d, *J* = 9.1 Hz, 1H), 7.93 (d, *J* = 8.2 Hz, 1H), 7.75 – 7.60 (m, 3H), 7.55 (ddd, *J* = 8.2, 6.8, 1.2 Hz, 1H), 7.51 – 7.45 (m, 1H), 7.45 – 7.37 (m, 2H), 7.36 – 7.23 (m, 3H), 7.08 (s, 1H), 2.33 (s, 3H).

^{13}C NMR (101 MHz, $CDCl_3$) δ 195.1, 148.5, 147.6, 144.3, 139.8, 138.4, 136.9, 134.0, 133.4, 133.2, 132.5, 132.0, 131.6, 129.9, 129.6, 128.5, 128.4, 128.2, 127.4, 127.2, 126.7, 126.2, 125.8, 119.0, 118.2 (q, *J* = 321.2 Hz), 21.8.

^{19}F NMR (376 MHz, $CDCl_3$) δ -74.70.

HRMS: (ESI) calculated for $C_{28}H_{18}F_3NNaO_4S$ $[M+Na]^+$ m/z: 544.0801, found: 544.0787.

3h

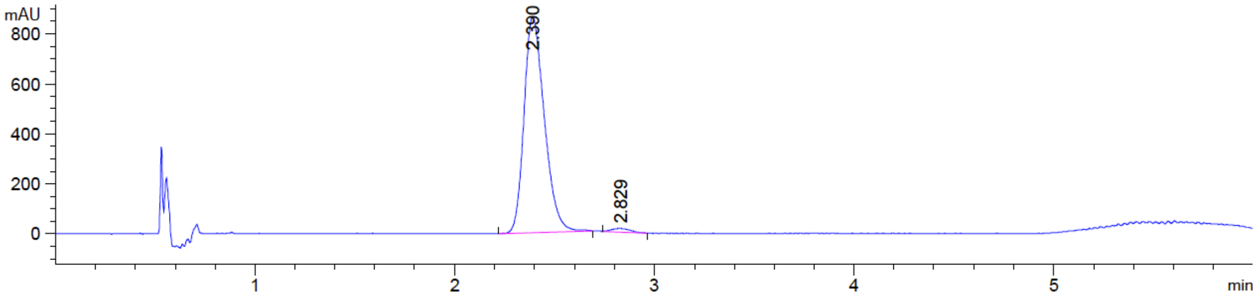

Peak #	RetTime [min]	Type	Width [min]	Area [mAU*s]	Height [mAU]	Area %
1	2.390	VV R	0.1084	6105.37012	863.42096	98.5120
2	2.829	BV R	0.0759	92.21756	14.76810	1.4880

***Rac*-3h**

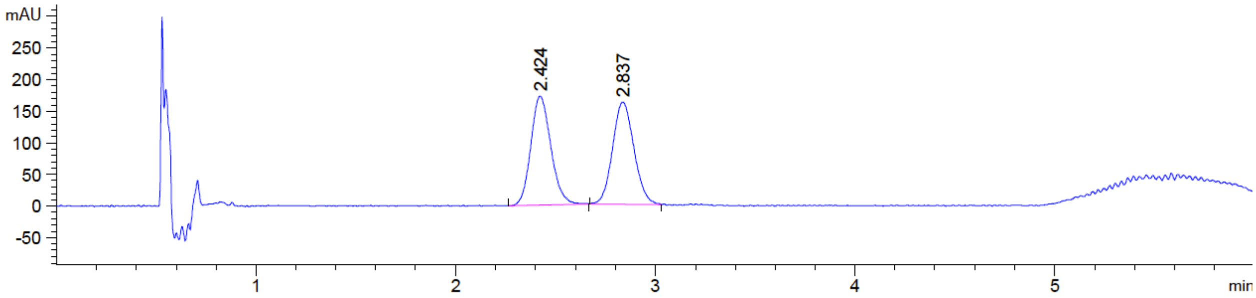

Peak #	RetTime [min]	Type	Width [min]	Area [mAU*s]	Height [mAU]	Area %
1	2.424	VV R	0.1064	1203.62085	172.47556	50.0155
2	2.837	BV R	0.1143	1202.87366	161.60008	49.9845

(*S*)-1-(3-Benzoyl-7-fluoroquinolin-4-yl)naphthalen-2-yl trifluoromethanesulfonate (3i)

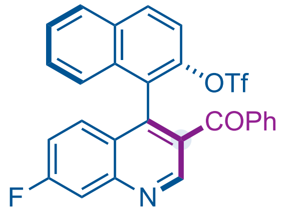

This compound was synthesized following the general procedure **D** using **1h** (40.9 mg, 0.1 mmol) and **2c-H** (29.2 mg, 0.2 mmol). Purification by flash column chromatography (hexane/ethyl acetate: 6/1) provided the title compound as white foam (51.0 mg, 97% yield, 91% ee). Enantiomeric excess was determined by SFC analysis on a chiral stationary phase (CHIRALPAK IG-3, 1 mL/min, 10% methanol, λ = 210 nm, t_r(major) = 1.965 min, t_r(minor) = 2.510 min).

$[\alpha]_D^{23}$ = +48.6 (c = 0.12, $CHCl_3$).

^{1}H NMR (400 MHz, $CDCl_3$) δ 9.18 (s, 1H), 8.00 (d, *J* = 9.1 Hz, 1H), 7.97 – 7.90 (m, 2H), 7.71 – 7.63 (m, 2H), 7.57 (ddd, *J* = 8.2, 6.9, 1.2 Hz, 1H), 7.53 – 7.47 (m, 1H), 7.44 (ddd, *J* = 8.3, 6.9, 1.3 Hz, 1H), 7.40 (d, *J* = 9.1 Hz, 1H), 7.38 – 7.30 (m, 3H), 7.29 – 7.19 (m, 2H).

^{13}C NMR (101 MHz, $CDCl_3$) δ 194.6, 164.2 (d, *J* = 254.5 Hz), 150.7, 150.2 (d, *J* = 13.0 Hz), 144.2, 140.9, 136.7, 133.6, 133.1, 132.1, 132.0 (d, *J* = 2.7 Hz), 131.9, 130.0, 129.9 (d, *J* = 10.0 Hz), 128.6, 128.5, 128.4, 127.5, 126.5, 125.9, 124.4, 119.2, 118.8 (d, *J* = 25.4 Hz), 118.2 (q, *J* = 321.2 Hz), 113.7 (d, *J* = 20.6 Hz).

^{19}F NMR (376 MHz, $CDCl_3$) δ -74.58, -105.96.

HRMS: (ESI) calculated for $C_{27}H_{16}F_4NO_4S$ $[M+H]^+$ m/z: 526.0731, found: 526.0744.

3i

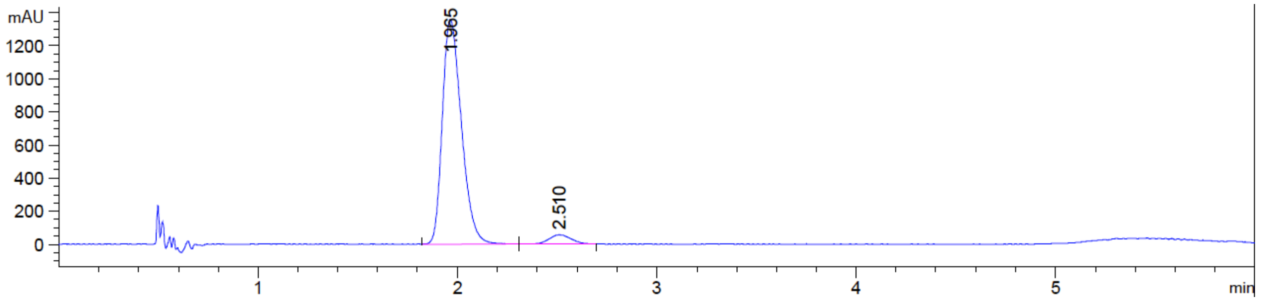

Peak #	RetTime [min]	Type	Width [min]	Area [mAU*s]	Height [mAU]	Area %
1	1.965	VV R	0.1036	9041.33008	1359.14526	95.3859
2	2.510	VV R	0.1000	437.36008	56.06297	4.6141

***Rac*-3i**

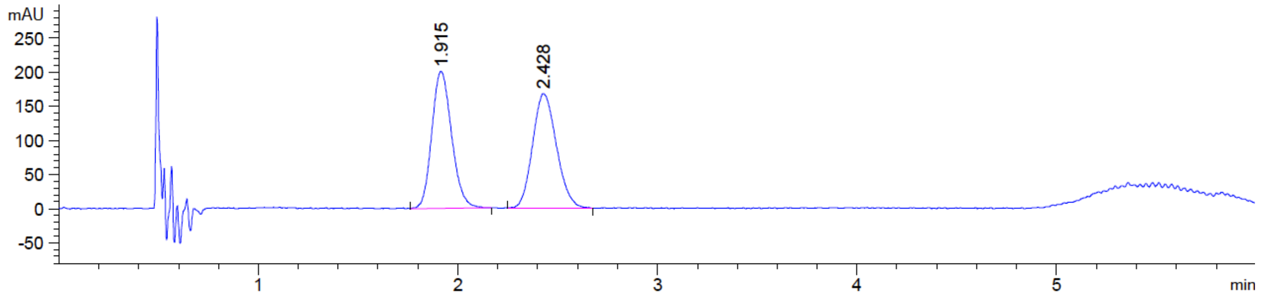

Peak #	RetTime [min]	Type	Width [min]	Area [mAU*s]	Height [mAU]	Area %
1	1.915	BV R	0.1035	1386.51294	200.90332	49.8019
2	2.428	VV R	0.1265	1397.54199	168.01009	50.1981

(*S*)-1-(3-Benzoyl-7-chloroquinolin-4-yl)naphthalen-2-yl trifluoromethanesulfonate (3j)

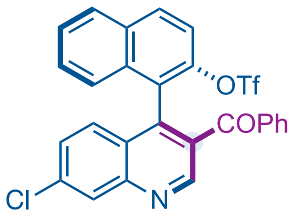

This compound was synthesized following the general procedure **D** using **1i** (42.6 mg, 0.1 mmol) and **2c-H** (29.2 mg, 0.2 mmol). Purification by flash column chromatography (hexane/ethyl acetate: 6/1) provided the title compound as white foam (53.1 mg, 98% yield, 96% ee). Enantiomeric excess was determined by SFC analysis on a chiral stationary phase (CHIRALPAK IG-3, 1 mL/min, 20% methanol, λ = 210 nm, t_r(major) = 1.891 min, t_r(minor) = 2.500 min).

$[\alpha]_D^{23}$ = +71.7 (c = 0.12, $CHCl_3$).

^{1}H NMR (400 MHz, $CDCl_3$) δ 9.18 (s, 1H), 8.32 (d, *J* = 2.1 Hz, 1H), 7.99 (d, *J* = 9.1 Hz, 1H), 7.94 (dt, *J* = 8.3, 0.9 Hz, 1H), 7.73 – 7.64 (m, 2H), 7.57 (ddd, *J* = 8.2, 6.9, 1.2 Hz, 1H), 7.53 – 7.47

(m, 1H), 7.47 – 7.37 (m, 3H), 7.37 – 7.30 (m, 2H), 7.28 (d, J = 9.0 Hz, 1H), 7.22 (d, J = 8.5 Hz, 1H).

^{13}C NMR (101 MHz, $CDCl_3$) δ 194.5, 150.5, 149.2, 144.1, 140.8, 137.8, 136.6, 133.6, 133.1, 132.7, 132.1, 132.0, 130.0, 129.2, 129.0, 128.7, 128.6, 128.5, 128.4, 127.5, 126.5, 125.8, 125.7, 119.1, 118.2 (q, J = 321.2 Hz).

^{19}F NMR (376 MHz, $CDCl_3$) δ -74.52.

HRMS: (ESI) calculated for $C_{27}H_{15}ClF_3NNaO_4S$ $[M+Na]^+$ m/z: 564.0255, found: 564.0259.

3j

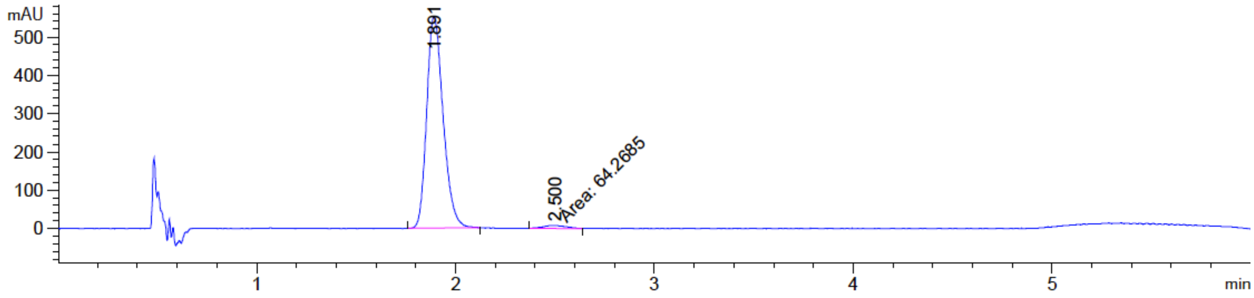

Peak #	RetTime [min]	Type	Width [min]	Area [mAU*s]	Height [mAU]	Area %
1	1.891	BV R	0.0881	3180.67627	552.73822	98.0194
2	2.500	MM	0.1366	64.26848	7.84138	1.9806

***Rac*-3j**

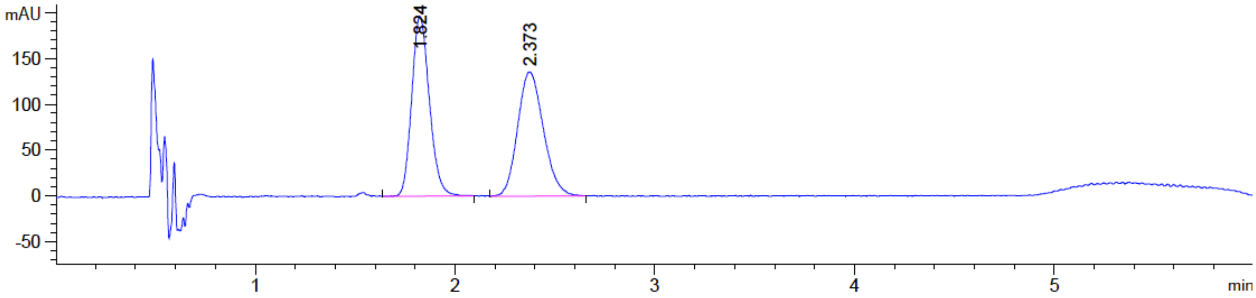

Peak #	RetTime [min]	Type	Width [min]	Area [mAU*s]	Height [mAU]	Area %
1	1.824	VV R	0.0936	1214.77869	196.53532	50.0022
2	2.373	VV R	0.1265	1214.67236	135.65475	49.9978

(*S*)-1-(3-Benzoyl-7-methylquinolin-4-yl)naphthalen-2-yl trifluoromethanesulfonate (3k)

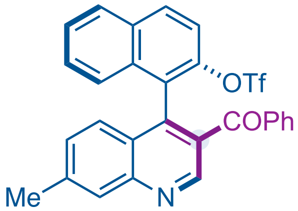

This compound was synthesized following the general procedure **D** using **1j** (40.5 mg, 0.1 mmol) and **2c-H** (29.2 mg, 0.2 mmol). Purification by flash column chromatography (hexane/ethyl acetate: 6/1) provided the title compound as pale yellow foam (45.9 mg, 88% yield, 97% ee). Enantiomeric excess was determined by SFC analysis on a chiral stationary phase (CHIRALPAK IC-3, 1 mL/min, 10% methanol, λ = 210 nm, t_r(major) = 1.995 min, t_r(minor) = 1.763 min).

$[\alpha]_D^{23}$ = +84.2 (c = 0.12, $CHCl_3$).

^{1}H NMR (400 MHz, $CDCl_3$) δ 9.15 (s, 1H), 8.10 (s, 1H), 7.97 (d, *J* = 9.1 Hz, 1H), 7.92 (d, *J* = 8.2 Hz, 1H), 7.75 – 7.65 (m, 2H), 7.54 (ddd, *J* = 8.2, 6.8, 1.2 Hz, 1H), 7.51 – 7.45 (m, 1H), 7.45 – 7.37 (m, 2H), 7.37 – 7.19 (m, 5H), 2.60 (s, 3H).

^{13}C NMR (101 MHz, $CDCl_3$) δ 195.0, 149.6, 149.2, 144.1, 142.4, 140.5, 136.9, 133.3, 133.2, 132.0, 131.7, 131.6, 130.4, 130.0, 128.9, 128.5, 128.4, 128.2, 127.3, 127.0, 126.7, 126.4, 125.4, 119.1, 118.2 (q, *J* = 321.2 Hz), 22.0.

^{19}F NMR (376 MHz, $CDCl_3$) δ -74.61.

HRMS: (ESI) calculated for $C_{28}H_{19}F_3NO_4S$ $[M+H]^+$ m/z: 522.0981, found: 522.0990.

3k

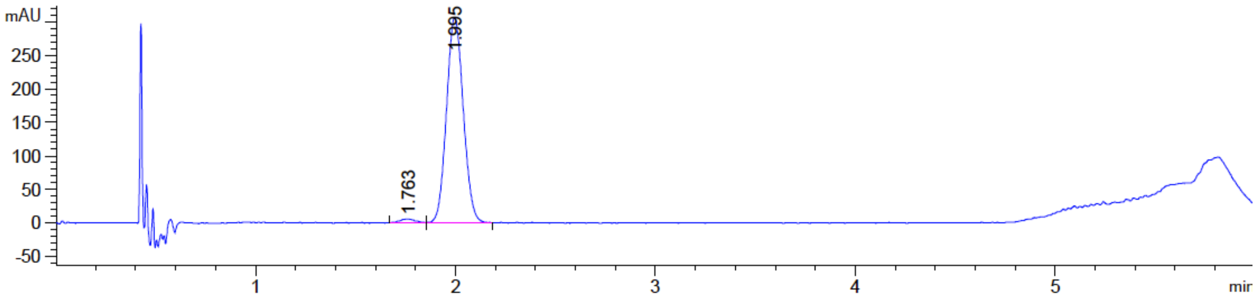

Peak #	RetTime [min]	Type	Width [min]	Area [mAU*s]	Height [mAU]	Area %
1	1.763	VB R	0.0599	28.76267	5.94991	1.5800
2	1.995	BV R	0.0897	1791.61218	306.39172	98.4200

***Rac*-3k**

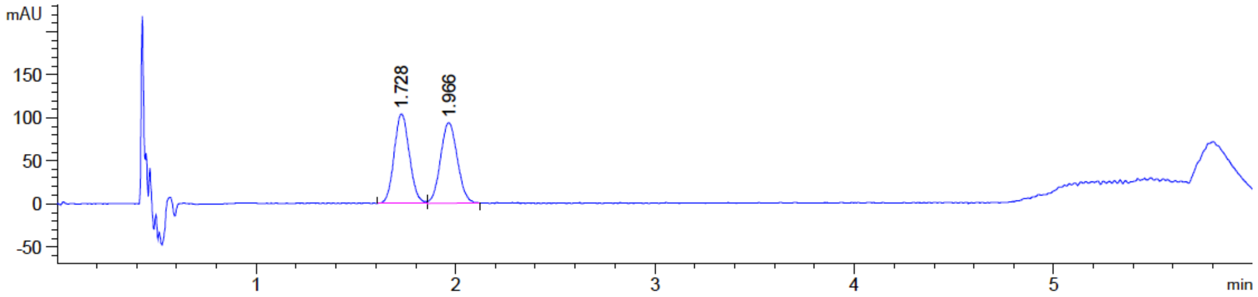

Peak #	RetTime [min]	Type	Width [min]	Area [mAU*s]	Height [mAU]	Area %
1	1.728	VV R	0.0826	552.11353	103.69892	50.0936
2	1.966	VV R	0.0946	550.05060	93.58289	49.9064

(*S*)-1-(3-Benzoyl-7-phenylquinolin-4-yl)naphthalen-2-yl trifluoromethanesulfonate (3l)

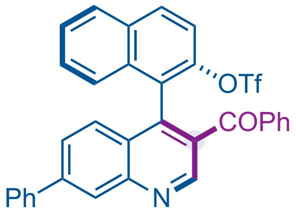

This compound was synthesized following the general procedure **D** using **1k** (46.7 mg, 0.1 mmol) and **2c-H** (29.2 mg, 0.2 mmol). Purification by flash column chromatography (hexane/ethyl acetate: 6/1) provided the title compound as pale yellow foam (56.6 mg, 97% yield, 97% ee). Enantiomeric excess was determined by SFC analysis on a chiral stationary phase (CHIRALPAK IC-3, 1 mL/min, 15% methanol, λ = 210 nm, t_r(major) = 2.596 min, t_r(minor) = 2.203 min).

$[\alpha]_D^{23}$ = +92.4 (c = 0.18, $CHCl_3$).

^{1}H NMR (400 MHz, $CDCl_3$) δ 9.21 (s, 1H), 8.56 (d, *J* = 1.9 Hz, 1H), 8.01 (d, *J* = 9.1 Hz, 1H), 7.95 (d, *J* = 8.3 Hz, 1H), 7.83 – 7.77 (m, 2H), 7.77 – 7.68 (m, 3H), 7.57 (ddd, *J* = 8.2, 6.8, 1.2 Hz, 1H), 7.54 – 7.48 (m, 3H), 7.48 – 7.28 (m, 7H).

^{13}C NMR (101 MHz, $CDCl_3$) δ 194.9, 150.0, 149.3, 144.2, 140.7, 139.5, 136.8, 133.5, 133.2, 132.3, 132.1, 131.7, 130.0, 129.2, 128.6, 128.53, 128.46, 128.3, 127.8, 127.74, 127.66, 127.4, 127.3, 126.7, 126.4, 126.2, 119.1, 118.2 (q, *J* = 321.2 Hz). (1 missing signal due to aromatic overlap)

^{19}F NMR (376 MHz, $CDCl_3$) δ -74.53.

HRMS: (ESI) calculated for $C_{33}H_{20}F_3NNaO_4S$ $[M+Na]^+$ m/z: 606.0957, found: 606.0955.

3l

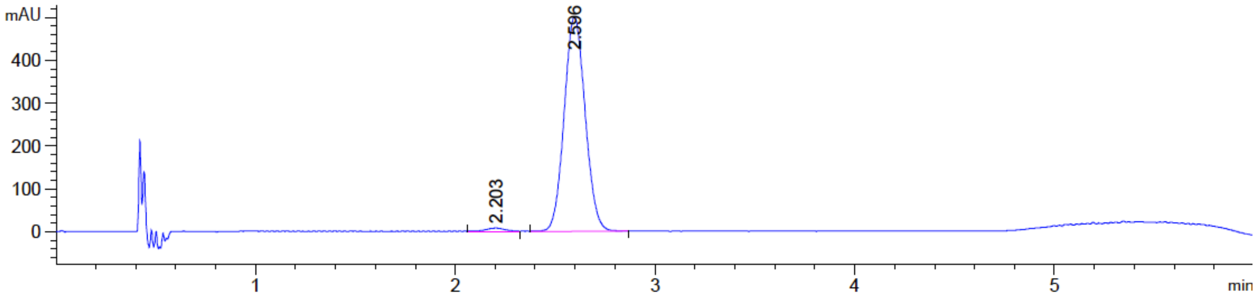

Peak #	RetTime [min]	Type	Width [min]	Area [mAU*s]	Height [mAU]	Area %
1	2.203	VB R	0.0792	52.61128	8.27581	1.4152
2	2.596	VV R	0.1153	3664.92578	501.36920	98.5848

***Rac*-3l**

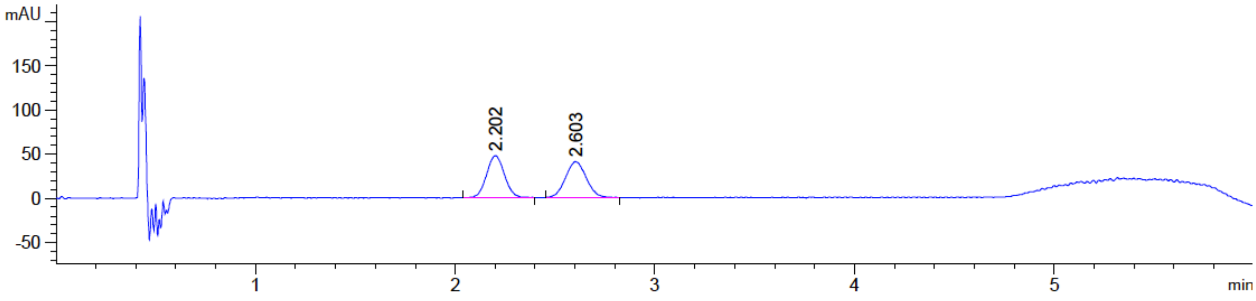

Peak #	RetTime [min]	Type	Width [min]	Area [mAU*s]	Height [mAU]	Area %
1	2.202	VV R	0.0951	299.49530	47.78160	50.2005
2	2.603	BV R	0.1050	297.10330	40.77110	49.7995

(*R*)-1-(3-Benzoyl-2,3-dihydroquinolin-4-yl)-5-methoxynaphthalen-2-yl trifluoromethanesulfonate (3o)

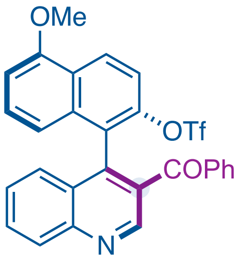

This compound was synthesized following the general procedure **D** using **1n** (42.1 mg, 0.1 mmol) and **2c-H** (29.2 mg, 0.2 mmol). Purification by flash column chromatography (hexane/ethyl acetate: 4/1) provided the title compound as colorless oil (44.4 mg, 83% yield, 98% ee). Enantiomeric excess was determined by SFC analysis on a chiral stationary phase (CHIRALPAK IA-3, 1 mL/min, 10% methanol, λ = 210 nm, t_r(major) = 3.139 min, t_r(minor) = 3.933 min).

$[\alpha]_D^{23}$ = +37.7 (c = 0.055, $CHCl_3$).

^{1}H NMR (400 MHz, $CDCl_3$) δ 9.15 (s, 1H), 8.44 (dd, *J* = 9.4, 0.8 Hz, 1H), 8.30 (dd, *J* = 8.6, 1.0 Hz, 1H), 7.84 (ddd, *J* = 8.4, 6.8, 1.4 Hz, 1H), 7.75 – 7.65 (m, 2H), 7.55 – 7.29 (m, 8H), 6.90 – 6.84 (m, 1H), 6.81 – 6.73 (m, 1H), 4.03 (s, 3H).

^{13}C NMR (101 MHz, $CDCl_3$) δ 194.9, 155.8, 149.5, 148.9, 145.0, 141.0, 136.9, 134.5, 133.5, 132.5, 131.5, 130.1, 130.0, 128.8, 128.5, 128.0, 127.5, 127.3, 126.3, 125.4, 124.5, 118.8, 118.2 (q, *J* = 319.1 Hz), 118.1, 105.3, 56.0.

^{19}F NMR (376 MHz, $CDCl_3$) δ -74.67.

HRMS (ESI): calculated for $C_{28}H_{19}F_3NO_5S^+$ $[M+H]^+$ m/z: 538.0931, found 538.0952.

3o

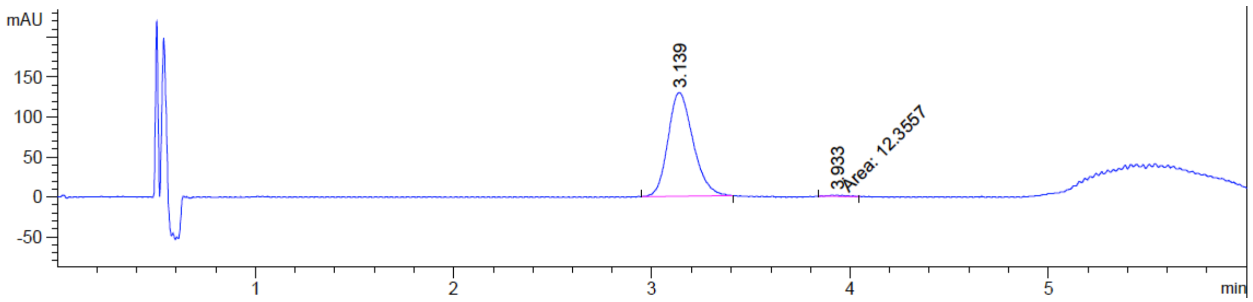

Peak #	RetTime [min]	Type	Width [min]	Area [mAU*s]	Height [mAU]	Area %
1	3.139	VV R	0.1259	1149.94226	129.79755	98.9370
2	3.933	MM	0.1176	12.35569	1.75162	1.0630

***Rac*-3o**

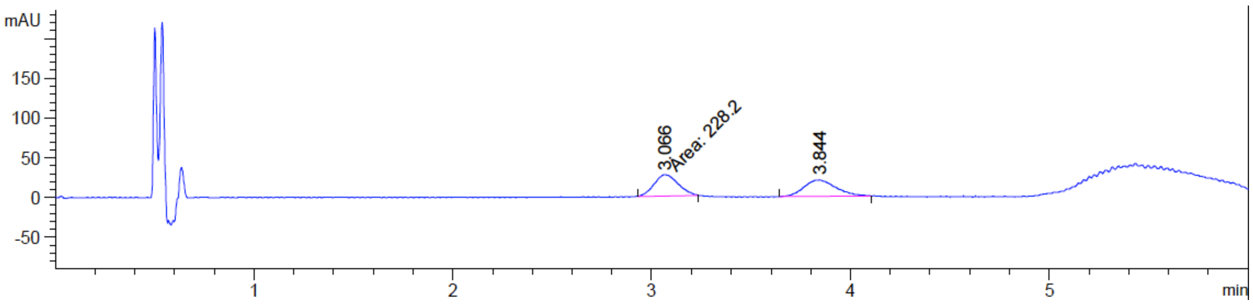

Peak #	RetTime [min]	Type	Width [min]	Area [mAU*s]	Height [mAU]	Area %
1	3.066	MM	0.1421	228.19989	26.75813	49.3572
2	3.844	VV R	0.1372	234.14366	20.74224	50.6428

(*R*)-1-(3-Benzoyl-2,3-dihydroquinolin-4-yl)-6-bromonaphthalen-2-yl trifluoromethanesulfonate (3p)

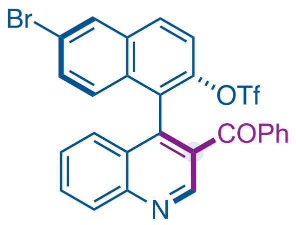

This compound was synthesized following the general procedure **D** using **1o** (47.0 mg, 0.1 mmol) and **2c-H** (29.2 mg, 0.2 mmol). Purification by flash column chromatography (hexane/ethyl acetate: 5/1) provided the title compound pale yellow foam (49.1 mg, 84% yield, 94% ee).

Enantiomeric excess was determined by SFC analysis on a chiral stationary phase (CHIRALPAK IG-3, 1 mL/min, 20% methanol, λ = 210 nm, t_r(major) = 1.760 min, t_r(minor) = 2.613 min).

$[\alpha]_D^{23}$ = +15.4 (c = 0.09, $CHCl_3$).

^{1}H NMR (500 MHz, $CDCl_3$) δ 9.18 (s, 1H), 8.32 (d, *J* = 8.5 Hz, 1H), 8.12 (d, *J* = 2.0 Hz, 1H), 7.91 (d, *J* = 9.1 Hz, 1H), 7.86 (t, *J* = 7.7 Hz, 1H), 7.70 (d, *J* = 7.7 Hz, 2H), 7.59 – 7.45 (m, 3H), 7.43 (d, *J* = 9.2 Hz, 1H), 7.38 (t, *J* = 7.6 Hz, 2H), 7.27 (d, *J* = 8.4 Hz, 1H), 7.12 (d, *J* = 9.0 Hz, 1H).

^{13}C NMR (126 MHz, $CDCl_3$) δ 194.7, 149.5, 149.1, 144.9, 140.0, 136.7, 134.4, 133.7, 132.5, 131.8, 131.6, 131.1, 130.5, 130.2, 130.11, 130.06, 128.8, 128.6, 128.4, 127.1, 127.0, 125.6, 123.0, 119.7, 118.1 (d, *J* = 320.3 Hz).
^{19}F NMR (471 MHz, $CDCl_3$) δ -74.46.

HRMS (ESI): calculated for $C_{27}H_{16}BrF_3NO_4S^+$ $[M+H]^+$ m/z: 585.9930, found 585.9931.

3p

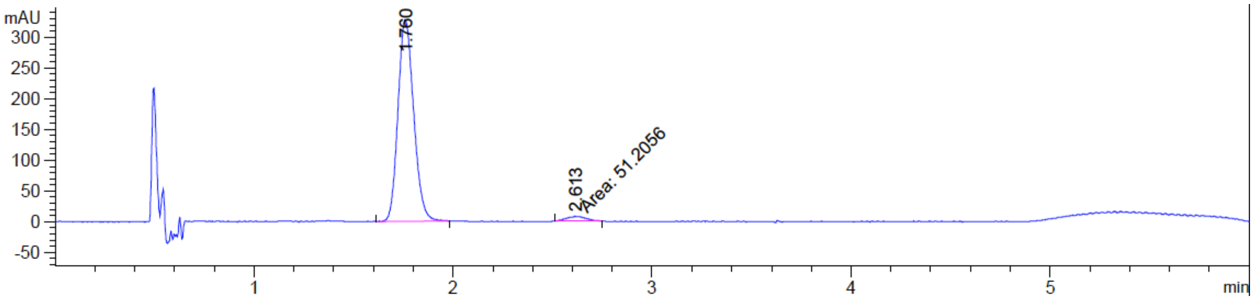

Peak #	RetTime [min]	Type	Width [min]	Area [mAU*s]	Height [mAU]	Area %
1	1.760	VV R	0.0859	1799.03333	328.26166	97.2325
2	2.613	MM	0.1105	51.20563	7.72483	2.7675

***Rac*-3p**

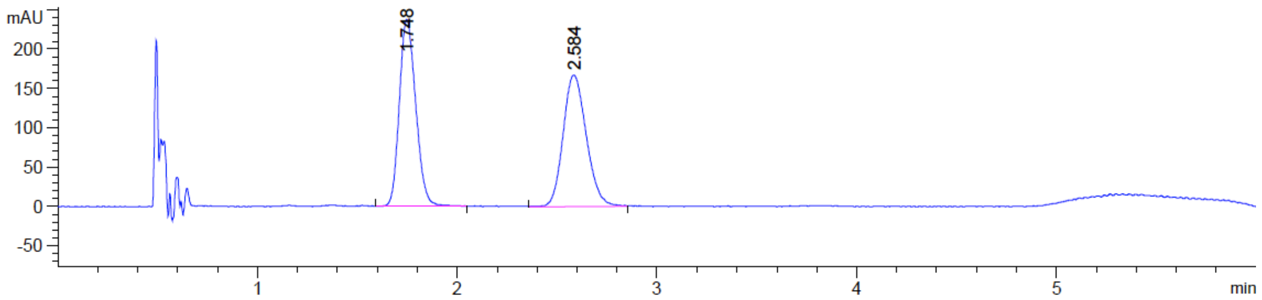

Peak #	RetTime [min]	Type	Width [min]	Area [mAU*s]	Height [mAU]	Area %
1	1.748	BV R	0.0897	1359.03418	237.53429	50.2432
2	2.584	VV R	0.1246	1345.87805	166.94963	49.7568

(*R*)-1-(3-Benzoyl-2,3-dihydroquinolin-4-yl)-7-bromonaphthalen-2-yl trifluoromethanesulfonate (3q)

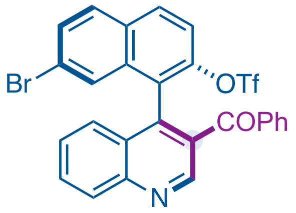

This compound was synthesized following the general procedure **D** using **1p** (47.0 mg, 0.1 mmol) and **2c-H** (29.2 mg, 0.2 mmol). Purification by flash column chromatography (hexane/ethyl acetate: 5/1) provided the title compound as white foam (46.4 mg, 79% yield, 96% ee). Enantiomeric excess was determined by SFC analysis on a chiral stationary phase (CHIRALPAK IG-3, 1 mL/min, 20% methanol, λ = 210 nm, t_r(major) = 1.763 min, t_r(minor) = 1.460 min).

$[\alpha]_D^{23}$ = +40.5 (c = 0.13, $CHCl_3$).

^{1}H NMR (500 MHz, $CDCl_3$) δ 9.18 (s, 1H), 8.33 (d, *J* = 8.3 Hz, 1H), 7.96 (d, *J* = 9.1 Hz, 1H), 7.88 (ddd, *J* = 8.4, 6.9, 1.4 Hz, 1H), 7.80 (d, *J* = 8.7 Hz, 1H), 7.72 – 7.66 (m, 2H), 7.63 (dd, *J* = 8.8, 1.9 Hz, 1H), 7.56 – 7.47 (m, 2H), 7.44 (d, *J* = 9.1 Hz, 1H), 7.41 – 7.35 (m, 3H), 7.29 (ddd, *J* = 8.5, 1.4, 0.6 Hz, 1H).

^{13}C NMR (126 MHz, $CDCl_3$) δ 194.7, 149.5, 149.1, 144.9, 140.0, 136.7, 134.4, 133.7, 132.5, 131.8, 131.6, 131.1, 130.5, 130.2, 130.11, 130.06, 128.8, 128.6, 128.4, 127.1, 127.0, 125.6, 123.0, 119.7, 118.2 (q, J = 320.3 Hz).

^{19}F NMR (471 MHz, $CDCl_3$) δ -74.49.

HRMS (ESI): calculated for $C_{27}H_{16}BrF_3NO_4S^+$ $[M+H]^+$ m/z: 585.9930, found 585.9912.

3q

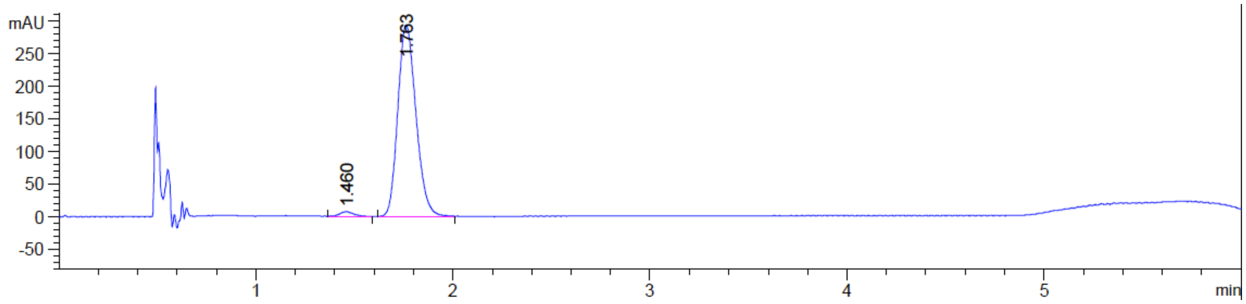

Peak #	RetTime [min]	Type	Width [min]	Area [mAU*s]	Height [mAU]	Area %
1	1.460	VV R	0.0632	35.94529	7.32932	1.8820
2	1.763	BV R	0.0990	1874.05554	293.67081	98.1180

***Rac*-3q**

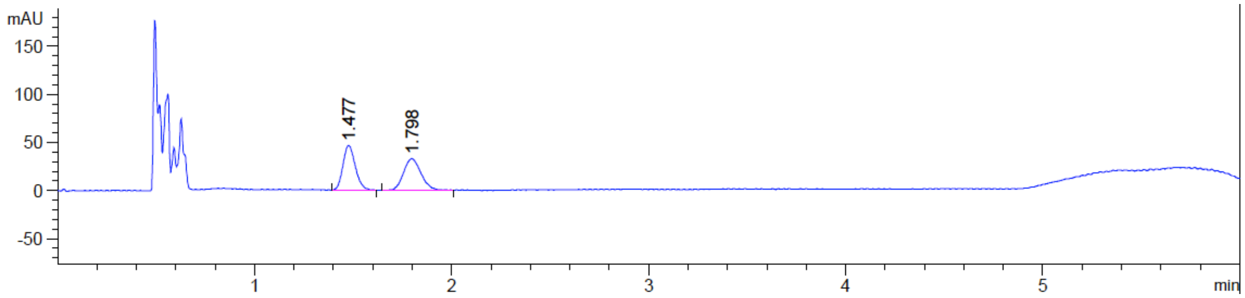

Peak #	RetTime [min]	Type	Width [min]	Area [mAU*s]	Height [mAU]	Area %
1	1.477	BV R	0.0664	204.86928	46.51034	49.9810
2	1.798	VV R	0.0944	205.02531	33.04647	50.0190

(*R*)-1-(3-Benzoyl-2,3-dihydroquinolin-4-yl)-7-methoxynaphthalen-2-yl trifluoromethanesulfonate (3r)

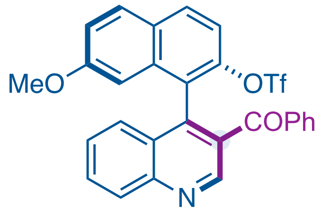

This compound was synthesized following the general procedure **D** using **1q** (42.1 mg, 0.1 mmol) and **2c-H** (29.2 mg, 0.2 mmol). Purification by flash column chromatography (hexane/ethyl acetate: 4/1) provided the title compound as white foam (47.5 mg, 88% yield, 92% ee). Enantiomeric excess was determined by SFC analysis on a chiral stationary phase (CHIRALPAK IC-3, 1.2 mL/min, 2.5% methanol, λ = 210 nm, t_r(major) = 7.089 min, t_r(minor) = 6.259 min).

$[\alpha]_D^{23}$ = +40.7 (c = 0.07, $CHCl_3$).

^{1}H NMR (500 MHz, $CDCl_3$) δ 9.18 (s, 1H), 8.31 (d, *J* = 8.5 Hz, 1H), 7.91 – 7.79 (m, 3H), 7.72 – 7.62 (m, 2H), 7.54 – 7.45 (m, 2H), 7.40 – 7.29 (m, 3H), 7.24 (d, *J* = 9.0 Hz, 1H), 7.20 (dd, *J* = 9.0, 2.5 Hz, 1H), 6.45 (d, *J* = 2.5 Hz, 1H), 3.52 (s, 3H).

^{13}C NMR (126 MHz, $CDCl_3$) δ 195.0, 159.4, 149.6, 149.0, 145.0, 140.8, 136.8, 134.7, 133.5, 132.6, 131.6, 131.3, 130.1, 130.04, 129.97, 128.5, 128.1, 127.6, 127.3, 127.0, 124.5, 120.1, 118.2 (d, *J* = 320.2 Hz), 116.5, 105.2, 55.4.

^{19}F NMR (471 MHz, $CDCl_3$) δ -74.60.

HRMS (ESI): calculated for $C_{28}H_{18}F_3NNaO_5S^+$ $[M+Na]^+$ m/z: 560.0750, found 560.0759.

3r

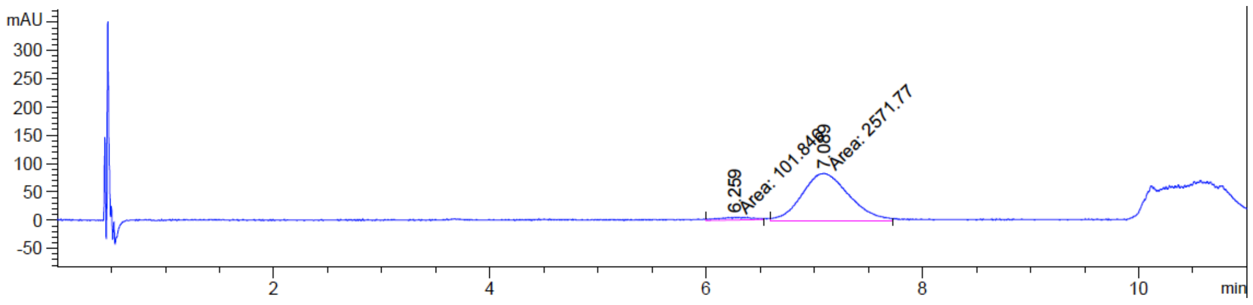

Peak #	RetTime [min]	Type	Width [min]	Area [mAU*s]	Height [mAU]	Area %
1	6.259	MM	0.3443	101.84607	4.92989	3.8093
2	7.089	MM	0.5084	2571.76904	84.30804	96.1907

***Rac*-3r**

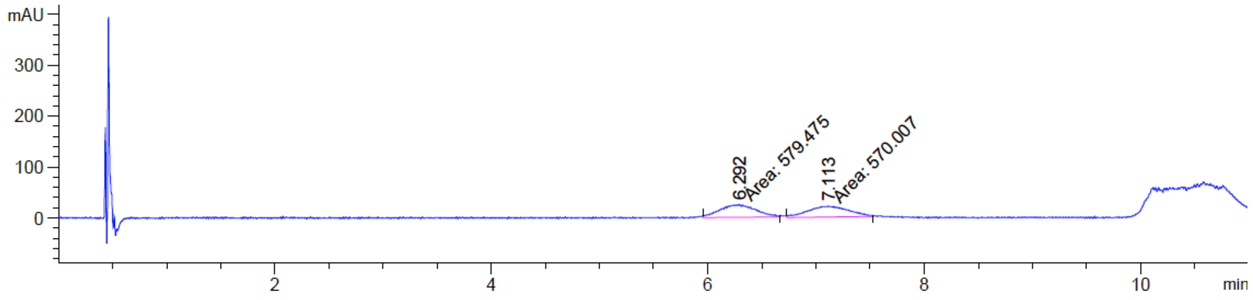

Peak #	RetTime [min]	Type	Width [min]	Area [mAU*s]	Height [mAU]	Area %
1	6.292	MM	0.3888	579.47485	24.84067	50.4118
2	7.113	MM	0.4419	570.00659	21.49938	49.5882

(*R*)-1-(3-Benzoyl-2,3-dihydroquinolin-4-yl)-6,8-difluoronaphthalen-2-yl trifluoromethanesulfonate (3s)

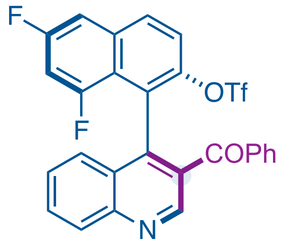

This compound was synthesized following the general procedure **D** using **1r** (42.7 mg, 0.1 mmol) and **2c-H** (29.2 mg, 0.2 mmol). Purification by flash column chromatography (hexane/ethyl acetate: 5/1) provided the title compound as colorless oil (37.6 mg, 69% yield, 97% ee). Enantiomeric excess was determined by SFC analysis on a chiral stationary phase (CHIRALPAK IG-3, 1 mL/min, 10% methanol, λ = 210 nm, t_r(major) = 3.023 min, t_r(minor) = 2.540 min).

$[\alpha]_D^{23}$ = +14.2 (c = 0.05, $CHCl_3$).

^{1}H NMR (400 MHz, $CDCl_3$) δ 9.12 (s, 1H), 8.32 – 8.25 (m, 1H), 7.96 (dd, J = 9.2, 1.5 Hz, 1H), 7.85 (ddd, J = 8.4, 6.8, 1.4 Hz, 1H), 7.79 – 7.70 (m, 2H), 7.59 – 7.36 (m, 7H), 7.36 – 7.27 (m, 1H), 6.95 (ddd, J = 12.0, 8.6, 2.5 Hz, 1H).

^{13}C NMR (101 MHz, $CDCl_3$) δ 195.0, 161.4 (dd, J = 92.2, 12.5 Hz), 158.9 (dd, J = 101.3, 12.4 Hz), 149.5, 148.7, 144.1 (d, J = 3.0 Hz), 142.6 (d, J = 2.6 Hz), 137.1, 133.9 (dd, J = 10.7, 4.1 Hz), 133.5, 131.5, 130.8 (dd, J = 5.7, 3.2 Hz), 130.2, 130.0, 128.6, 128.0, 127.3 (d, J = 3.9 Hz), 126.4, 123.9, 121.4, 121.2 (dd, J = 10.8, 2.0 Hz), 119.7, 116.5, 108.4 (dd, J = 20.9, 4.7 Hz), 105.0 (dd, J = 28.9, 25.2 Hz).

^{19}F NMR (376 MHz, $CDCl_3$) δ -74.67, -105.38 (d, J = 8.6 Hz), -109.27 (d, J = 8.6 Hz).

HRMS (ESI): calculated for $C_{27}H_{15}F_5NO_4S^+$ $[M+H]^+$ m/z: 544.0636, found 544.0650.

3s

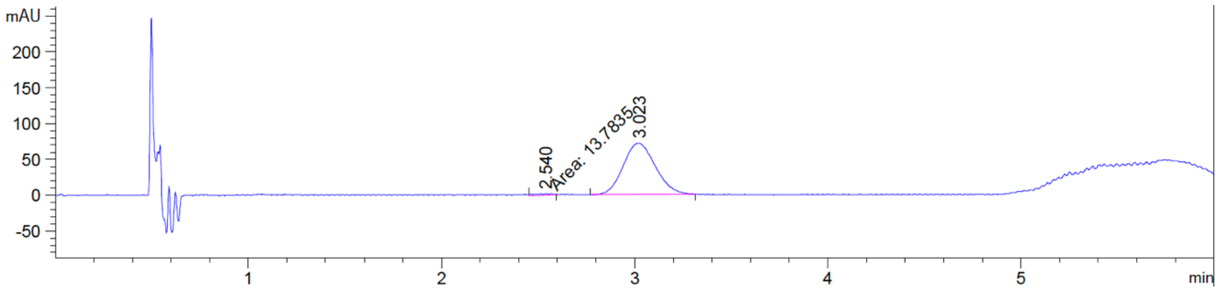

Peak #	RetTime [min]	Type	Width [min]	Area [mAU*s]	Height [mAU]	Area %
1	2.540	MM	0.1173	13.78352	1.95825	1.6266
2	3.023	VV R	0.1420	833.62164	71.77446	98.3734

*Note: **Rac-3s** was not able to synthetize using $Rh_2(esp)_2$, $Rh_2(OAc)_4$ or $Rh_2(pivalate)_4$. Instead, we synthetize **3s'**using $Rh_2(R\text{-}Br\text{-}NTTL)_4$.*

3s'

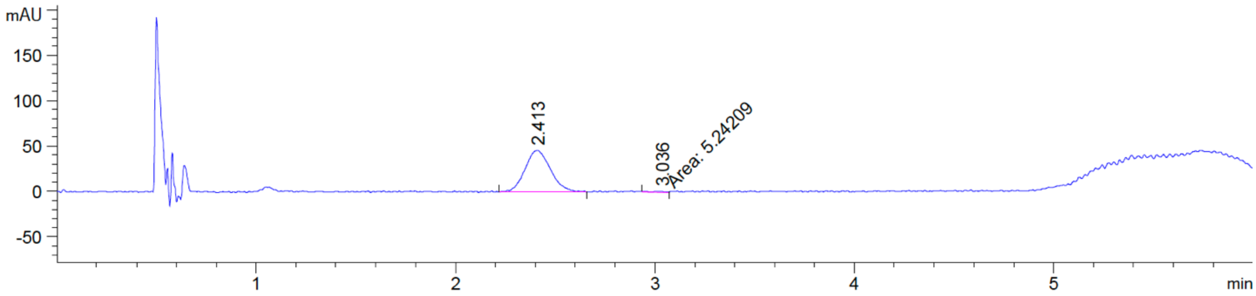

Peak #	RetTime [min]	Type	Width [min]	Area [mAU*s]	Height [mAU]	Area %
1	2.413	VV R	0.1082	412.31796	45.95076	98.7446
2	3.036	MM	0.0859	5.24209	1.01711	1.2554

(*S*)-1-(3-Benzoylquinolin-4-yl)naphthalen-2-yl methanesulfonate (3t)

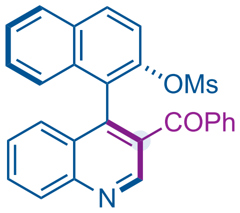

This compound was synthesized following the general procedure **D** using **1s** (33.7 mg, 0.1 mmol) and **2c-H** (29.2 mg, 0.2 mmol). Purification by flash column chromatography (hexane/ethyl acetate: 6/1) provided the title compound as pale yellow foam (43.5 mg, 96% yield, 94% ee). Enantiomeric excess was determined by SFC analysis on a chiral stationary phase (CHIRALPAK IB-3, 1 mL/min, 20% methanol, λ = 210 nm, t_r(major) = 1.852 min, t_r(minor) = 2.115 min).

$[\alpha]_D^{23}$ = +80.6 (c = 0.14, $CHCl_3$).

^{1}H NMR (400 MHz, $CDCl_3$) δ 9.19 (s, 1H), 8.30 (d, *J* = 8.5 Hz, 1H), 7.91 – 7.76 (m, 3H), 7.62 – 7.56 (m, 2H), 7.53 (d, *J* = 9.1 Hz, 1H), 7.48 – 7.37 (m, 3H), 7.37 – 7.29 (m, 2H), 7.23 – 7.12 (m, 3H), 2.61 (s, 3H).

^{13}C NMR (101 MHz, $CDCl_3$) δ 195.6, 149.6, 148.9, 144.8, 141.5, 136.8, 133.2, 132.9, 132.8, 131.5, 131.4, 131.3, 130.0, 129.5, 128.4, 128.2, 128.1, 127.7, 127.6, 127.2, 126.5, 126.3, 124.3, 120.4, 38.8.

HRMS: (ESI) calculated for $C_{27}H_{20}NO_4S$ $[M+H]^+$ m/z: 454.1108, found: 454.1099.

3t

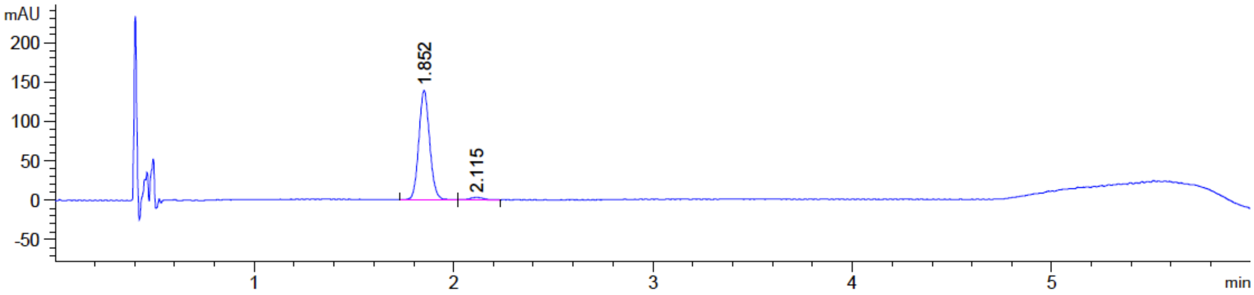

Peak #	RetTime [min]	Type	Width [min]	Area [mAU*s]	Height [mAU]	Area %
1	1.852	VV R	0.0586	525.44263	138.86620	97.2473
2	2.115	VV R	0.0599	14.87322	3.18720	2.7527

***Rac*-3t**

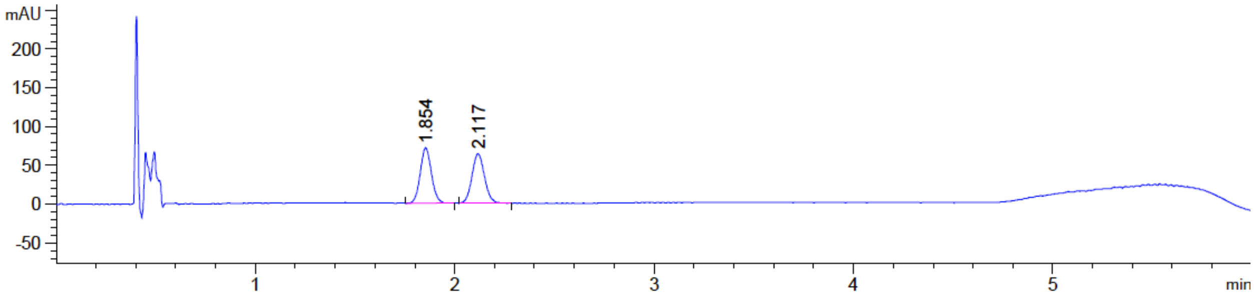

Peak #	RetTime [min]	Type	Width [min]	Area [mAU*s]	Height [mAU]	Area %
1	1.854	BV R	0.0608	279.45294	71.21361	50.0501
2	2.117	BV R	0.0691	278.89313	63.67460	49.9499

(*S*)-3-(3-Benzoylquinolin-4-yl)naphthalen-2-yl trifluoromethanesulfonate (3u)

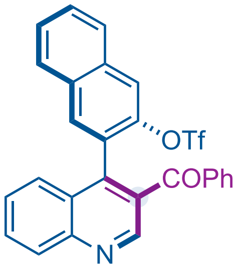

This compound was synthesized following the general procedure **D** at -20 ℃ using **1t** (39.1 mg, 0.1 mmol) and **2c-H** (29.2 mg, 0.2 mmol). Purification by flash column chromatography (hexane/ethyl acetate: 6/1) provided the title compound as pale yellow oil (49.2 mg, 97% yield, 93% ee). Enantiomeric excess was determined by SFC analysis on a chiral stationary phase (CHIRALPAK IA-3, 1 mL/min, 15% methanol, λ = 210 nm, t_r(major) = 1.892 min, t_r(minor) = 2.500 min).

$[\alpha]_D^{23}$ = +21.0 (c = 0.18, $CHCl_3$).

^{1}H NMR (400 MHz, $CDCl_3$) δ 9.12 (s, 1H), 8.30 (d, *J* = 8.5 Hz, 1H), 7.93 (s, 1H), 7.91 – 7.83 (m, 3H), 7.81 (s, 1H), 7.76 (d, *J* = 7.9 Hz, 2H), 7.71 (d, *J* = 8.5 Hz, 1H), 7.65 – 7.49 (m, 4H), 7.39 (t, *J* = 7.8 Hz, 2H).

^{13}C NMR (101 MHz, $CDCl_3$) δ 195.2, 149.1, 149.0, 144.7, 142.4, 136.9, 133.6, 133.4, 132.8, 131.7, 131.6, 131.4, 130.2, 129.9, 128.6, 128.28, 128.25, 128.1, 128.0, 127.9, 127.1, 127.0, 126.9, 119.4, 118.2 (q, *J* = 321.2 Hz).

^{19}F NMR (376 MHz, $CDCl_3$) δ -74.23.

HRMS: (ESI) calculated for $C_{27}H_{16}F_3NNaO_4S$ $[M+Na]^+$ m/z: 530.0644, found: 530.0632.

3u

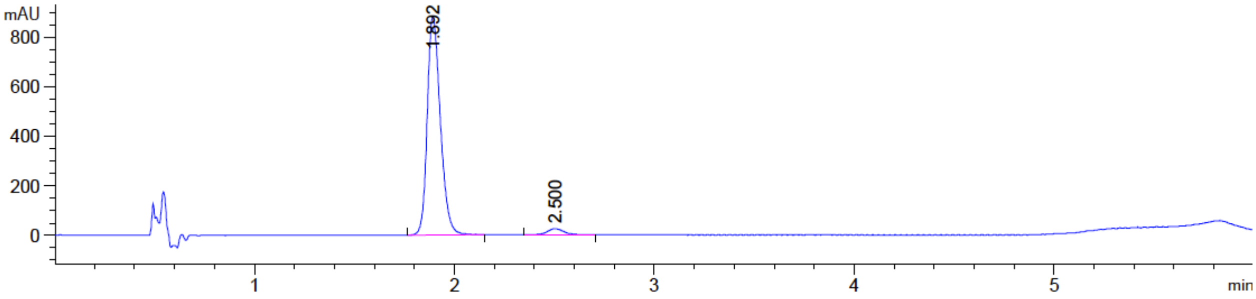

Peak #	RetTime [min]	Type	Width [min]	Area [mAU*s]	Height [mAU]	Area %
1	1.892	BV R	0.0696	4025.51733	884.78040	96.5181
2	2.500	VV R	0.0753	145.21983	24.80307	3.4819

***Rac*-3u**

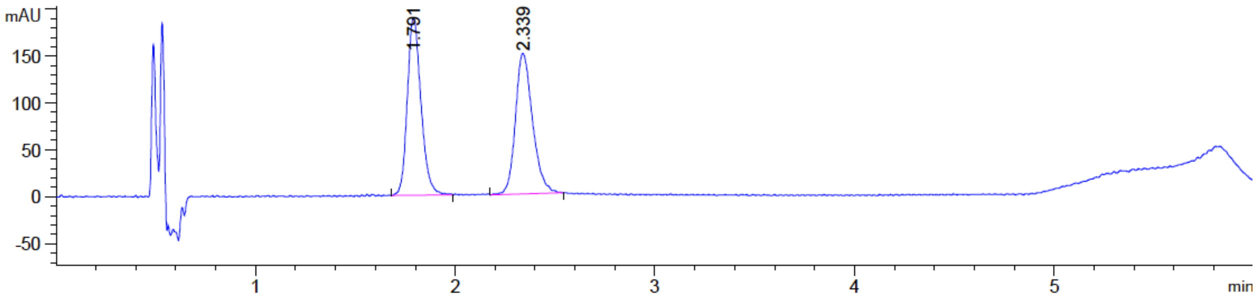

Peak #	RetTime [min]	Type	Width [min]	Area [mAU*s]	Height [mAU]	Area %
1	1.791	BV R	0.0726	900.46411	189.00209	50.1226
2	2.339	VV R	0.0907	896.05908	150.04230	49.8774

(*S*)-2-(3-Benzoylquinolin-4-yl)phenyl trifluoromethanesulfonate (3v)

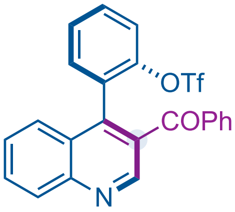

This compound was synthesized following the general procedure **D** at -20 °C using **1u** (34.1 mg, 0.1 mmol) and **2c-H** (29.2 mg, 0.2 mmol). Purification by flash column chromatography (hexane/ethyl acetate: 8/1) provided the title compound as pale yellow oil (40.3 mg, 88% yield, 92% ee). Enantiomeric excess was determined by SFC analysis on a chiral stationary phase (CHIRALPAK IG-3, 1 mL/min, 10% methanol, λ = 210 nm, t_r(major) = 1.880 min, t_r(minor) = 2.215 min).

$[\alpha]_D^{24}$ = +86.9 (c = 0.11, $CHCl_3$).

^{1}H NMR (400 MHz, $CDCl_3$) δ 9.07 (s, 1H), 8.27 (d, *J* = 8.3 Hz, 1H), 7.85 (ddd, *J* = 8.4, 6.4, 1.8 Hz, 1H), 7.80 – 7.71 (m, 2H), 7.66 – 7.53 (m, 3H), 7.53 – 7.45 (m, 3H), 7.45 – 7.37 (m, 2H), 7.33 (d, *J* = 7.6 Hz, 1H).

^{13}C NMR (101 MHz, $CDCl_3$) δ 195.1, 149.03, 148.98, 146.7, 142.0, 136.9, 133.7, 133.0, 131.5, 131.4, 130.9, 130.2, 129.9, 129.1, 128.6, 128.1, 128.0, 126.8, 126.7, 121.6, 118.2 (q, *J* = 321.2 Hz).

^{19}**F NMR** (376 MHz, $CDCl_3$) δ -74.39.

HRMS: (ESI) calculated for $C_{23}H_{15}F_3NO_4S$ $[M+H]^+$ m/z: 458.0668, found: 458.0674.

3v

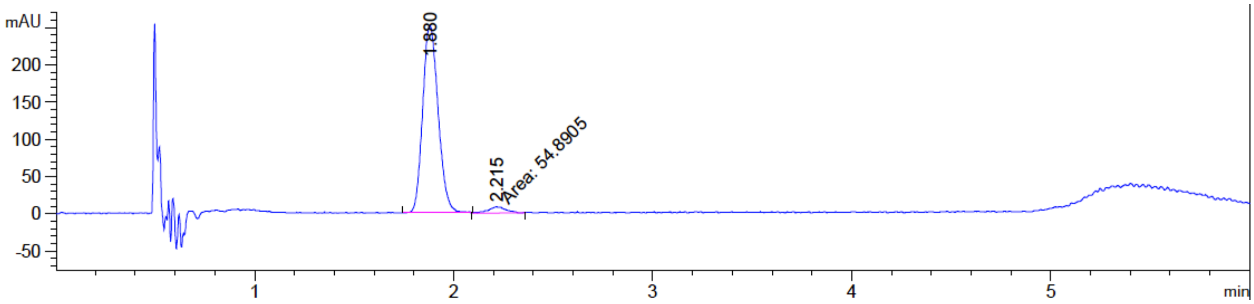

Peak #	RetTime [min]	Type	Width [min]	Area [mAU*s]	Height [mAU]	Area %
1	1.880	VV R	0.0855	1362.96667	252.31694	96.1286
2	2.215	MM	0.1095	54.89046	8.35267	3.8714

***Rac*-3v**

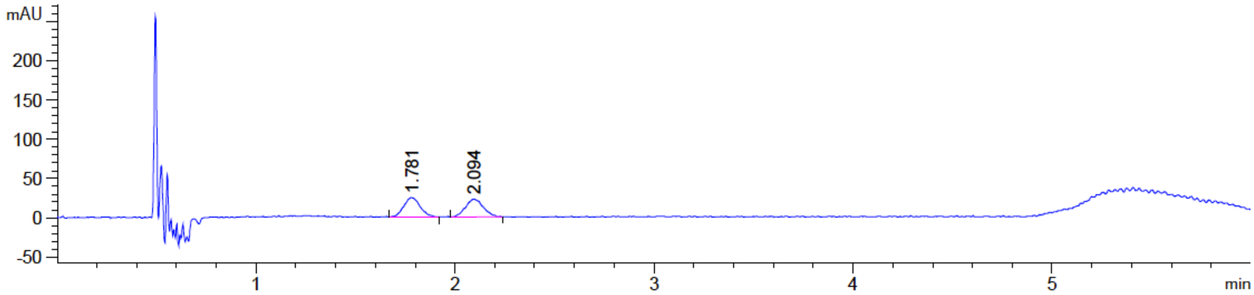

Peak #	RetTime [min]	Type	Width [min]	Area [mAU*s]	Height [mAU]	Area %
1	1.781	BV R	0.0810	144.41563	24.76741	50.0442
2	2.094	BV R	0.0804	144.16031	22.90788	49.9558

(*S*)-2-(3-Benzoylquinolin-4-yl)-4-methylphenyl trifluoromethanesulfonate (3w)

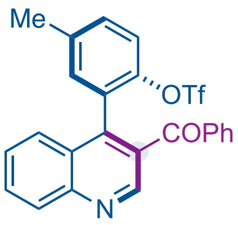

This compound was synthesized following the general procedure **D** at -20 °C using **1v** (35.5 mg, 0.1 mmol) and **2c-H** (29.2 mg, 0.2 mmol). Purification by flash column chromatography (hexane/ethyl acetate: 8/1) provided the title compound as pale brown oil (45.3 mg, 96% yield, 93% ee). Enantiomeric excess was determined by SFC analysis on a chiral stationary phase (CHIRALPAK ID-3, 1 mL/min, 10% methanol, λ = 210 nm, t_r(major) = 1.561 min, t_r(minor) = 2.219 min).

$[\alpha]_D^{23}$ = +51.9 (c = 0.21, $CHCl_3$).

^{1}H NMR (400 MHz, $CDCl_3$) δ 9.05 (s, 1H), 8.26 (d, *J* = 8.5 Hz, 1H), 7.85 (t, *J* = 7.6 Hz, 1H), 7.75 (d, *J* = 7.0 Hz, 2H), 7.66 (d, *J* = 7.1 Hz, 1H), 7.62 – 7.52 (m, 2H), 7.42 (t, *J* = 7.8 Hz, 2H), 7.30 – 7.22 (m, 2H), 7.19 (d, *J* = 8.4 Hz, 1H), 2.40 (s, 3H).

^{13}C NMR (101 MHz, $CDCl_3$) δ 195.2, 149.04, 149.98, 144.7, 142.2, 138.2, 136.9, 133.6, 133.3, 131.5, 131.43, 131.37, 130.3, 129.9, 128.7, 128.6, 128.0, 127.0, 126.8, 121.3, 118.2 (q, *J* = 321.2 Hz), 21.0.

^{19}F NMR (376 MHz, $CDCl_3$) δ -74.39.

HRMS: (APCI) calculated for $C_{24}H_{17}F_3NO_4S$ $[M+H]^+$ m/z: 472.0825, found: 472.0826.

3w

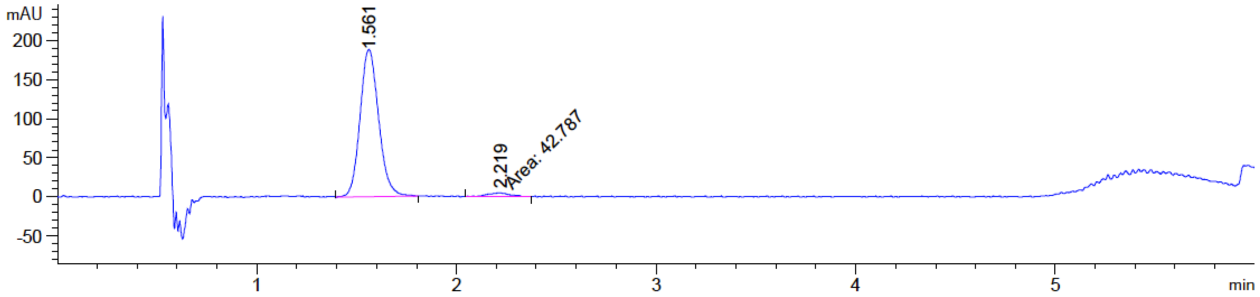

Peak #	RetTime [min]	Type	Width [min]	Area [mAU*s]	Height [mAU]	Area %
1	1.561	VV R	0.0995	1229.16931	188.72195	96.6361
2	2.219	MM	0.1407	42.78701	5.06853	3.3639

***Rac*-3w**

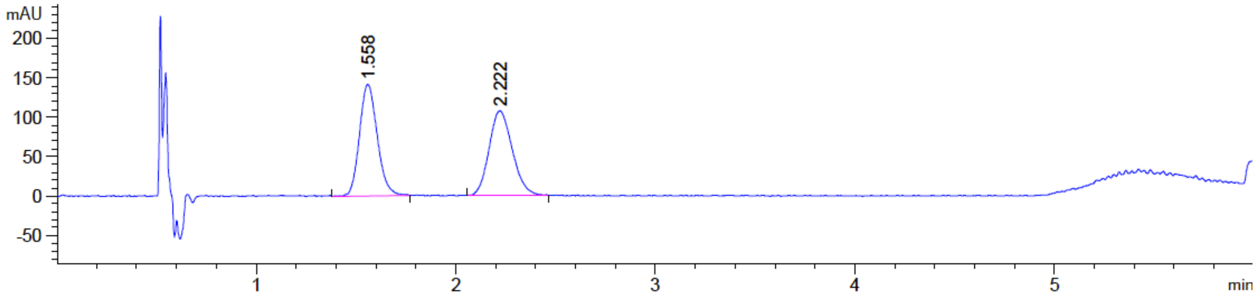

Peak #	RetTime [min]	Type	Width [min]	Area [mAU*s]	Height [mAU]	Area %
1	1.558	VV R	0.0946	883.25079	141.91640	50.1879
2	2.222	VV R	0.1133	876.63788	107.90697	49.8121

(*S*)-2-(3-Benzoylquinolin-4-yl)-4-(*tert*-butyl)phenyl trifluoromethanesulfonate (3x)

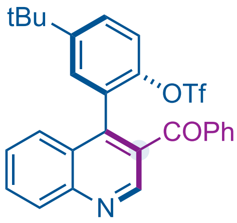

This compound was synthesized following the general procedure **D** at -20 °C using **1w** (39.7 mg, 0.1 mmol) and **2c-H** (29.2 mg, 0.2 mmol). Purification by flash column chromatography (hexane/ethyl acetate: 8/1) provided the title compound as pale yellow oil (30.0 mg, 58% yield, 89% ee). Enantiomeric excess was determined by SFC analysis on a chiral stationary phase (CHIRALPAK IG-3, 1 mL/min, 10% methanol, λ = 210 nm, t_r(major) = 1.218 min, t_r(minor) = 1.529 min).

$[\alpha]_D^{23}$ = +29.1 (c = 0.08, $CHCl_3$).

^{1}H NMR (400 MHz, $CDCl_3$) δ 9.07 (s, 1H), 8.27 (d, J = 8.4 Hz, 1H), 7.86 (ddd, J = 8.4, 6.4, 1.8 Hz, 1H), 7.72 – 7.66 (m, 2H), 7.66 – 7.57 (m, 2H), 7.55 – 7.48 (m, 1H), 7.43 (dd, J = 8.7, 2.5 Hz, 1H), 7.40 – 7.32 (m, 3H), 7.20 (d, J = 8.7 Hz, 1H), 1.28 (s, 9H).

^{13}C NMR (101 MHz, $CDCl_3$) δ 195.6, 151.2, 149.0, 148.9, 144.6, 142.0, 137.2, 133.5, 131.9, 131.2, 130.3, 130.01, 129.98, 128.5, 128.1, 128.0, 127.9, 126.8, 126.6, 120.9, 118.2 (q, J = 320.2 Hz), 34.9, 31.2.

^{19}F NMR (376 MHz, $CDCl_3$) δ -74.47.

HRMS: (ESI) calculated for $C_{27}H_{23}F_3NO_4S$ $[M+H]^+$ m/z: 514.1294, found: 514.1282.

3x

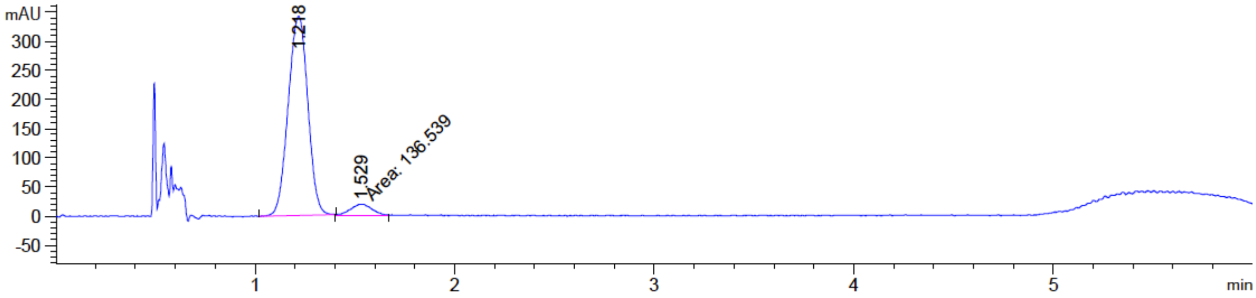

Peak #	RetTime [min]	Type	Width [min]	Area [mAU*s]	Height [mAU]	Area %
1	1.218	VV R	0.1078	2337.76270	341.78723	94.4817
2	1.529	MM	0.1219	136.53879	18.66590	5.5183

***Rac*-3x**

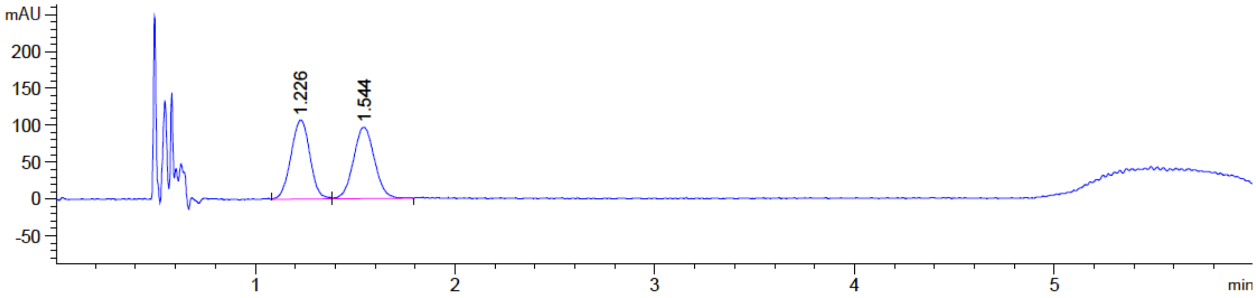

Peak #	RetTime [min]	Type	Width [min]	Area [mAU*s]	Height [mAU]	Area %
1	1.226	BV R	0.1020	701.96362	106.98853	49.9448
2	1.544	VV R	0.1132	703.51447	96.31600	50.0552

Methyl (*S*)-3-(3-benzoylquinolin-4-yl)-4-(((trifluoromethyl)sulfonyl)oxy)benzoate (3y)

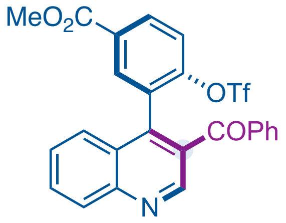

This compound was synthesized following the general procedure **D** at -20 °C using **1x** (40.0 mg, 0.1 mmol) and **2c-H** (29.2 mg, 0.2 mmol). Purification by flash column chromatography (hexane/ethyl acetate: 4/1) provided the title compound as pale yellow oil (38.1 mg, 74% yield, 89% ee). Enantiomeric ratio was determined by SFC analysis on a chiral stationary phase (CHIRALPAK IA-3, 1 mL/min, 10% methanol, λ = 210 nm, t_r(major) = 1.577 min, t_r(minor) = 1.988 min.

$[\alpha]_D^{28}$ = +25.3 (c = 1.03, $CHCl_3$).

^{1}H NMR (500 MHz, $CDCl_3$) δ 9.08 (s, 1H), 8.28 (d, *J* = 8.3 Hz, 1H), 8.16 (dd, *J* = 7.9, 1.5 Hz, 1H), 7.98 (d, *J* = 1.5 Hz, 1H), 7.88 (ddd, *J* = 8.4, 6.7, 1.5 Hz, 1H), 7.80 – 7.72 (m, 2H), 7.65 – 7.53 (m, 4H), 7.49 – 7.40 (m, 2H), 3.97 (s, 3H).

^{13}C NMR (126 MHz, $CDCl_3$) δ 194.8, 164.9, 149.1, 149.1, 146.6, 141.4, 136.6, 134.1, 133.9, 133.2, 133.0, 131.7, 131.1, 130.4, 130.1, 129.0, 128.8, 128.4, 126.5, 126.4, 122.9, 118.2 (q, *J* = 320.0 Hz), 52.9.

^{19}F NMR (471 MHz, $CDCl_3$) δ -74.06.

HRMS: (ESI) calculated for $C_{25}H_{16}F_3NNaO_6S$ $[M+Na]^+$ m/z: 538.0543, found: 538.0538.

3y

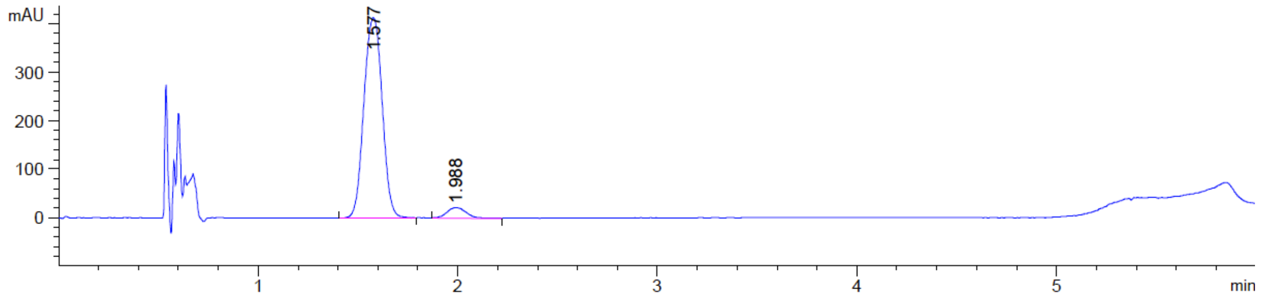

Peak #	RetTime [min]	Type	Width [min]	Area [mAU*s]	Height [mAU]	Area %
1	1.577	BV R	0.1034	2639.06372	413.96280	94.6849
2	1.988	BV R	0.0916	148.14372	21.84041	5.3151

***Rac*-3y**

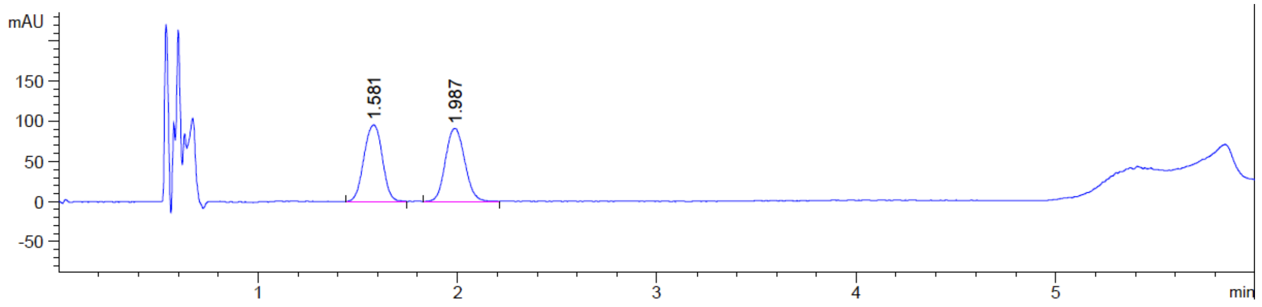

Peak #	RetTime [min]	Type	Width [min]	Area [mAU*s]	Height [mAU]	Area %
1	1.581	BV R	0.1039	612.79523	95.50219	49.9165
2	1.987	BV R	0.1066	614.84619	91.23788	50.0835

(*S*)-2-(3-Benzoylquinolin-4-yl)-4-cyanophenyl trifluoromethanesulfonate (3z)

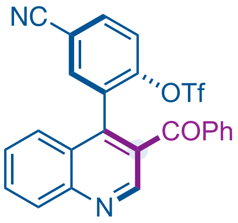

This compound was synthesized following the general procedure **D** at -20 °C using **1y** (36.7 mg, 0.1 mmol) and **2c-H** (29.2 mg, 0.2 mmol). Purification by flash column chromatography (hexane/ethyl acetate: 8/1) provided the title compound as pale yellow oil (40.3 mg, 84% yield,

87% ee). Enantiomeric ratio was determined by SFC analysis on a chiral stationary phase (CHIRALPAK IA-3, 1 mL/min, 10% methanol, λ = 210 nm, t_r(major) = 1.538 min, t_r(minor) = 2.180 min.

$[\alpha]_D^{28}$ = +31.0 (c = 1.24, $CHCl_3$).

^{1}H NMR (500 MHz, $CDCl_3$) δ 9.11 (s, 1H), 8.30 (d, J = 8.5 Hz, 1H), 7.88 (s, 1H), 7.83 – 7.70 (m, 3H), 7.70 – 7.56 (m, 4H), 7.48 (q, J = 9.1, 7.7 Hz, 3H).

^{13}C NMR (126 MHz, $CDCl_3$) δ 194.6, 149.3, 149.1, 146.5, 140.5, 136.5, 135.0, 134.1, 134.0, 131.9, 131.6, 130.9, 130.4, 130.3, 128.8, 128.6, 126.1, 126.0, 125.4, 118.1 (q, J = 321.3 Hz), 116.6, 114.8.

^{19}F NMR (471 MHz, $CDCl_3$) δ -73.86.

HRMS: (ESI) calculated for $C_{24}H_{14}F_3N_2O_4S$ $[M+H]^+$ m/z: 483.0621, found: 483.0626.

3z

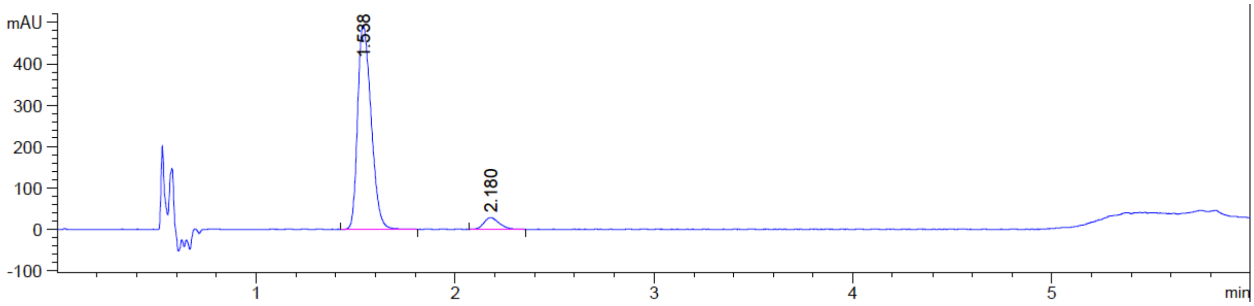

Peak #	RetTime [min]	Type	Width [min]	Area [mAU*s]	Height [mAU]	Area %
1	1.538	BV R	0.0750	2323.87646	493.05130	93.7234
2	2.180	BV R	0.0765	155.62784	28.36987	6.2766

***Rac*-3z**

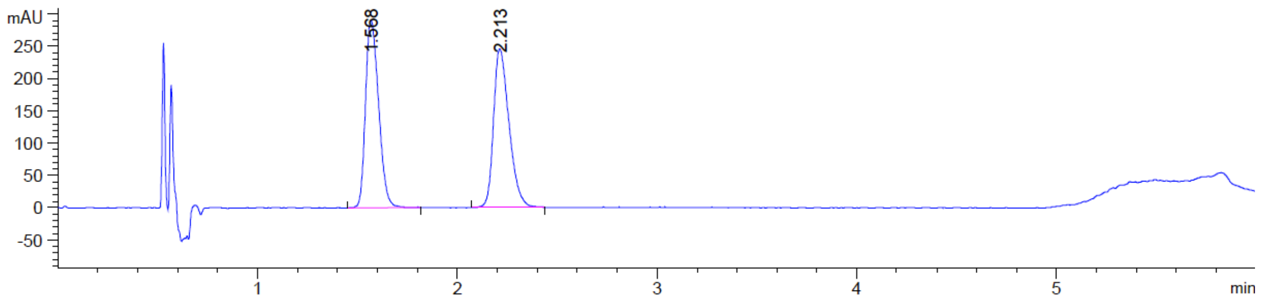

Peak #	RetTime [min]	Type	Width [min]	Area [mAU*s]	Height [mAU]	Area %
1	1.568	VV R	0.0724	1330.38062	290.90891	50.1393
2	2.213	BV R	0.0818	1322.98987	245.88881	49.8607

(*S*)-2-(3-Benzoylquinolin-4-yl)-4-(trifluoromethyl)phenyl trifluoromethanesulfonate (3aa)

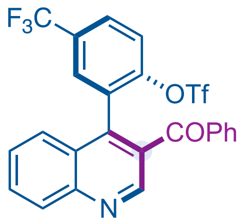

This compound was synthesized following the general procedure **D** at -20 °C using **1z** (41.0 mg, 0.1 mmol) and **2c-H** (29.2 mg, 0.2 mmol). Purification by flash column chromatography (hexane/ethyl acetate: 8/1) provided the title compound as pale yellow oil (21.0 mg, 40% yield, 71% ee). Enantiomeric ratio was determined by SFC analysis on a chiral stationary phase (CHIRALPAK IA-3, 1 mL/min, 5% isopropanol, λ = 210 nm, t_r(major) = 1.594 min, t_r(minor) = 2.542 min.

$[\alpha]_D^{28}$ = +42.6 (c = 0.99, $CHCl_3$).

^{1}H NMR (300 MHz, $CDCl_3$) δ 9.10 (s, 1H), 8.29 (d, *J* = 8.3 Hz, 1H), 7.89 (ddd, *J* = 8.4, 6.7, 1.5 Hz, 1H), 7.83 – 7.72 (m, 3H), 7.70 – 7.51 (m, 5H), 7.46 (dd, *J* = 8.4, 7.1 Hz, 2H).

^{13}C NMR (126 MHz, $CDCl_3$) δ 194.8, 149.2, 149.1, 146.5, 141.0, 136.6, 134.0, 133.8, 133.5, 133.2 (q, *J* = 34.0 Hz), 131.8, 131.1, 130.4, 130.2, 128.8, 128.5, 126.4, 126.4, 125.0 (q, *J* = 3.8 Hz), 122.8 (q, *J* = 273.4 Hz), 119.2, 118.2 (q, *J* = 321.3 Hz).

^{19}F NMR (282 MHz, $CDCl_3$) δ -62.86, -74.00.

HRMS: (ESI) calculated for $C_{24}H_{14}F_6NO_4S$ $[M+H]^+$ m/z: 526.0542, found: 526.0550.

3aa

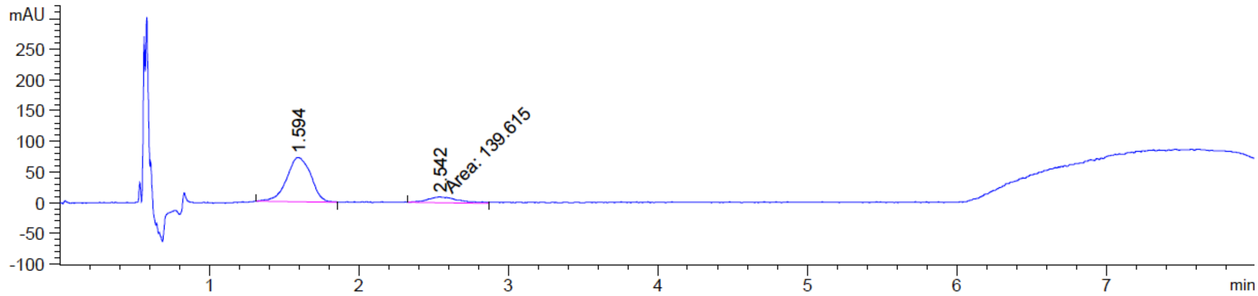

Peak #	RetTime [min]	Type	Width [min]	Area [mAU*s]	Height [mAU]	Area %
1	1.594	VV R	0.1406	818.17389	72.52707	85.4232
2	2.542	MM	0.2383	139.61516	9.76599	14.5768

***Rac*-3aa**

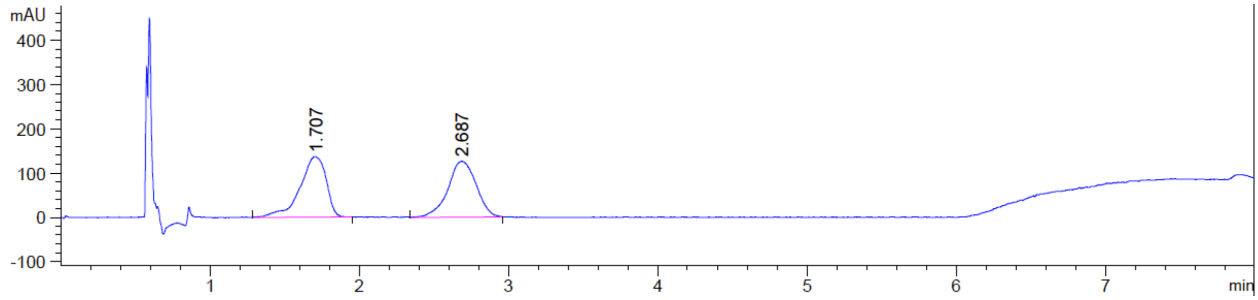

Peak #	RetTime [min]	Type	Width [min]	Area [mAU*s]	Height [mAU]	Area %
1	1.707	VV R	0.1644	1679.79688	136.61928	50.2583
2	2.687	VV R	0.1633	1662.52966	126.11505	49.7417

(*S*)-2-(3-Benzoylquinolin-4-yl)-5-fluoro-4-isopropylphenyl trifluoromethanesulfonate (3ab)

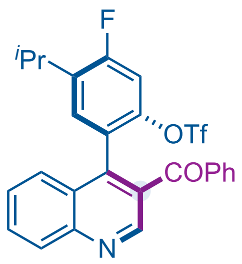

This compound was synthesized following the general procedure **D** at -20 °C using **1aa** (40.1 mg, 0.1 mmol) and **2c-H** (29.2 mg, 0.2 mmol). Purification by flash column chromatography (hexane/ethyl acetate: 8/1) provided the title compound as pale yellow oil (33.1 mg, 64% yield, 86% ee). Enantiomeric excess was determined by SFC analysis on a chiral stationary phase (CHIRALPAK IG-3, 1 mL/min, 5% methanol, λ = 210 nm, t_r(major) = 1.427 min, t_r(minor) = 1.912 min).

$[\alpha]_D^{23}$ = +41.5 (c = 0.14, $CHCl_3$).

^{1}H NMR (400 MHz, $CDCl_3$) δ 9.06 (s, 1H), 8.27 (dt, *J* = 8.5, 1.0 Hz, 1H), 7.86 (ddd, *J* = 8.4, 5.3, 3.0 Hz, 1H), 7.71 (dd, *J* = 8.3, 1.4 Hz, 2H), 7.65 – 7.58 (m, 2H), 7.58 – 7.51 (m, 1H), 7.44 – 7.35 (m, 2H), 7.29 (d, *J* = 7.9 Hz, 1H), 7.00 (d, *J* = 9.6 Hz, 1H), 3.24 (hept, *J* = 6.9 Hz, 1H), 1.25 (d, *J* = 6.9 Hz, 3H), 1.19 (d, *J* = 6.9 Hz, 3H).

^{13}C NMR (101 MHz, $CDCl_3$) δ 195.4, 160.4 (d, *J* = 252.3 Hz), 149.1, 149.0, 144.4 (d, *J* = 11.2 Hz), 141.2, 137.0, 136.0 (d, *J* = 15.3 Hz), 133.7, 131.9, 131.3 (d, *J* = 7.1 Hz), 131.3, 130.1, 130.0, 128.6, 128.2, 126.7, 126.5, 124.6 (d, *J* = 4.2 Hz), 118.2 (q, *J* = 320.6 Hz), 109.5 (d, *J* = 28.1 Hz), 27.2 (d, *J* = 1.6 Hz), 22.5, 22.4.

^{19}F NMR (376 MHz, $CDCl_3$) δ -74.28, -112.57.

HRMS: (ESI) calculated for $C_{26}H_{20}F_4NO_4S$ $[M+H]^+$ m/z: 518.1044, found: 518.1027.

3ab

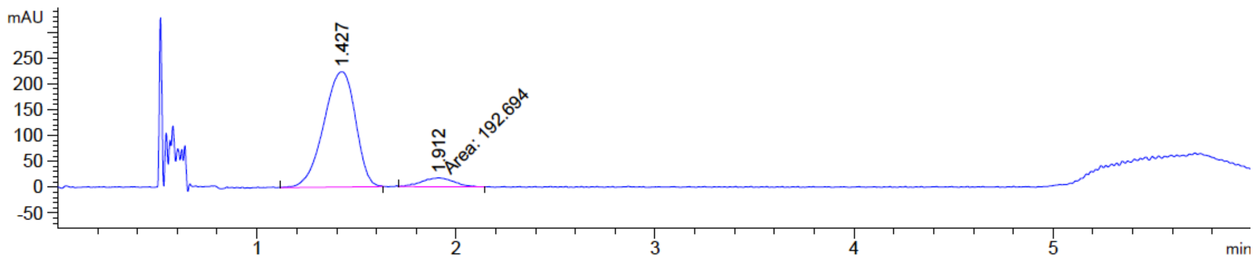

Peak #	RetTime [min]	Type	Width [min]	Area [mAU*s]	Height [mAU]	Area %
1	1.427	VV R	0.1516	2476.68677	222.87184	92.7813
2	1.912	MM	0.1874	192.69359	17.13779	7.2187

***Rac*-3ab**

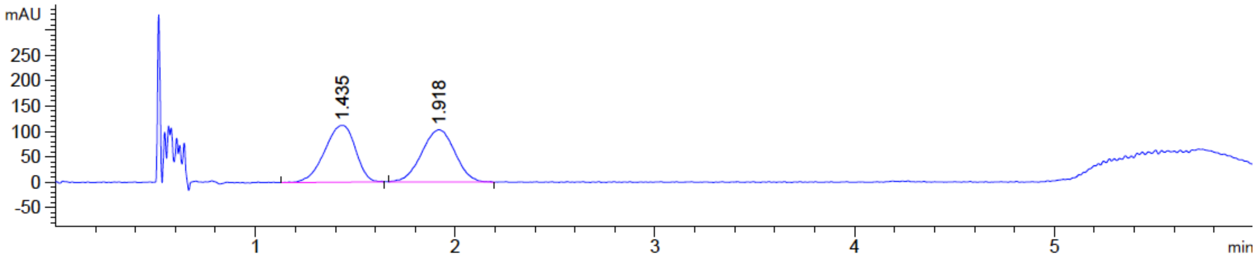

Peak #	RetTime [min]	Type	Width [min]	Area [mAU*s]	Height [mAU]	Area %
1	1.435	VV R	0.1533	1203.39722	112.01317	50.2783
2	1.918	VV R	0.1430	1190.07666	102.85667	49.7217

(*S*)-2-(3-Benzoylquinolin-4-yl)-3,5-dimethylphenyl trifluoromethanesulfonate (3ac)

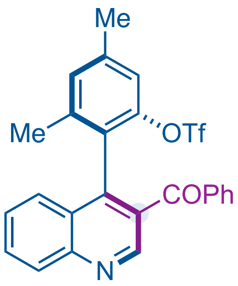

This compound was synthesized following the general procedure **D** at -20 °C using **1ab** (36.9 mg, 0.1 mmol) and **2c-H** (29.2 mg, 0.2 mmol). Purification by flash column chromatography (hexane/ethyl acetate: 8/1) provided the title compound as pale yellow oil (28.2 mg, 58% yield, 78% ee). Enantiomeric excess was determined by SFC analysis on a chiral stationary phase (CHIRALPAK IC-3, 1 mL/min, 5% methanol, λ = 210 nm, t_r(major) = 2.147 min, t_r(minor) = 1.541 min).

$[\alpha]_D^{23}$ = +4.1 (c = 0.15, $CHCl_3$).

^{1}H NMR (400 MHz, $CDCl_3$) δ 9.05 (s, 1H), 8.26 (d, J = 8.4 Hz, 1H), 7.84 (ddd, J = 8.4, 6.8, 1.5 Hz, 1H), 7.78 (dd, J = 8.3, 1.3 Hz, 2H), 7.65 – 7.54 (m, 2H), 7.50 (dd, J = 8.5, 0.9 Hz, 1H), 7.48 – 7.41 (m, 2H), 7.17 (s, 1H), 6.95 (s, 1H), 2.40 (s, 3H), 2.03 (s, 3H).

^{13}C NMR (101 MHz, $CDCl_3$) δ 195.1, 149.2, 148.9, 146.5, 142.1, 140.9, 140.6, 136.8, 133.7, 131.7, 131.4, 130.6, 130.4, 130.1, 128.6, 128.1, 126.8, 126.7, 125.7, 119.1, 118.1 (q, J = 321.2 Hz), 21.4, 20.4.

^{19}F NMR (376 MHz, $CDCl_3$) δ -74.82.

HRMS: (ESI) calculated for $C_{25}H_{19}F_3NO_4S$ $[M+H]^+$ m/z: 486.0981, found: 486.0980.

3ac

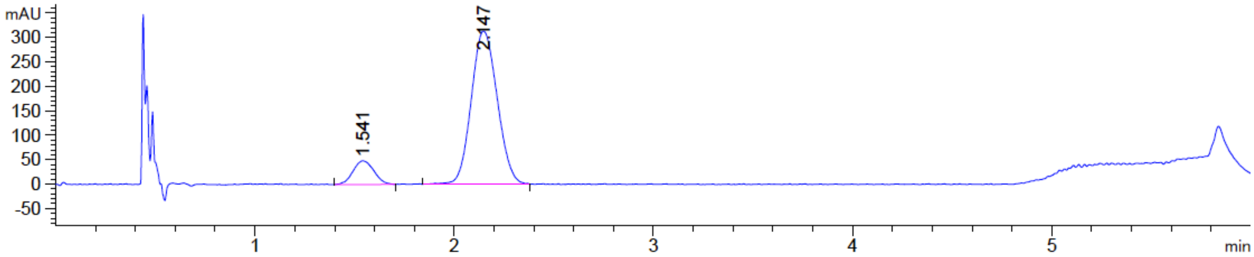

Peak #	RetTime [min]	Type	Width [min]	Area [mAU*s]	Height [mAU]	Area %
1	1.541	BB	0.1003	354.56573	48.48587	11.0890
2	2.147	VV R	0.1414	2842.89355	313.18491	88.9110

***Rac*-3ac**

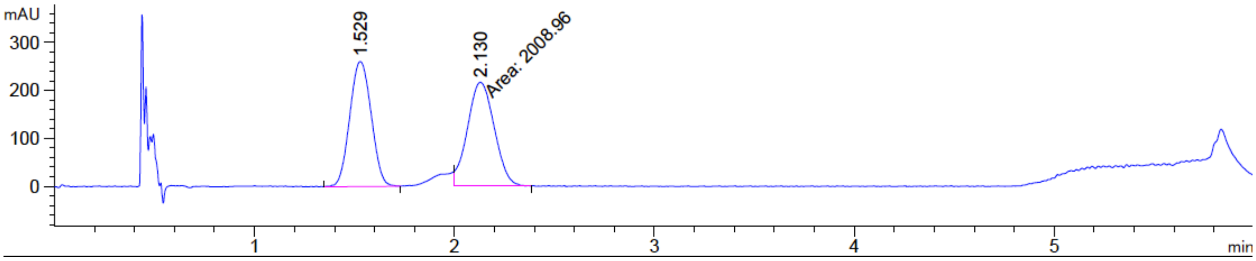

Peak #	RetTime [min]	Type	Width [min]	Area [mAU*s]	Height [mAU]	Area %
1	1.529	VB	0.1146	1943.67542	260.43912	49.1742
2	2.130	MM	0.1551	2008.95825	215.87030	50.8258

(*S*)-2-(3-benzoyl-6-((*N*-butylacetamido)methyl)quinolin-4-yl)phenyl trifluoromethanesulfonate (3ad)

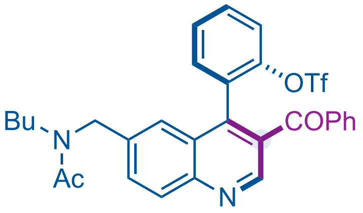

This compound was synthesized following the general procedure **D** at -20 °C using **1ac** (46.9 mg, 0.1 mmol) and **2c-H** (29.2 mg, 0.2 mmol). Purification by flash column chromatography (hexane/ethyl acetate: 1/2) provided the title compound as pale yellow oil (36.2 mg, 62% yield, 88% ee). Enantiomeric ratio was determined by SFC analysis on a chiral stationary phase (CHIRALPAK IB-3, 1 mL/min, 10% methanol, λ = 210 nm, t_r(major) = 1.975 min, t_r(minor) = 2.392 min.

$[\alpha]_D^{28}$ = +27.1 (c = 0.99, $CHCl_3$).

^{1}H NMR (300 MHz, $CDCl_3$) δ 9.11 – 8.95 (m, 1H), 8.31 – 8.12 (m, 1H), 7.82 – 7.63 (m, 3H), 7.62 – 7.37 (m, 6H), 7.36 – 7.27 (m, 2H), 4.94 – 4.47 (m, 2H), 3.38 – 2.97 (m, 2H), 2.17 – 1.98 (m, 3H), 1.44 (dtd, *J* = 13.9, 7.0, 3.1 Hz, 2H), 1.21 (h, *J* = 7.3 Hz, 2H), 0.84 (td, *J* = 7.3, 2.7 Hz, 3H). Messy due to amide effect.

^{13}C NMR (75 MHz, $CDCl_3$) δ 195.1, 170.8, 148.7, 146.6, 138.3, 136.9, 136.8, 133.8, 133.7, 133.0, 132.8, 131.6, 131.3, 131.0, 130.8, 130.3, 130.2, 129.6, 129.2, 128.6, 128.2, 126.6, 125.0, 123.9, 121.6, 116.1, 52.3, 48.3, 48.0, 30.6, 29.8, 21.8, 21.3, 20.2, 20.0, 13.9, 13.7. Messy due to amide effect.

^{19}F NMR (282 MHz, $CDCl_3$) δ -74.35, -74.40.

HRMS: (ESI) calculated for $C_{30}H_{27}F_3N_2NaO_5S$ $[M+Na]^+$ m/z: 607.1485, found: 607.1493.

3ad

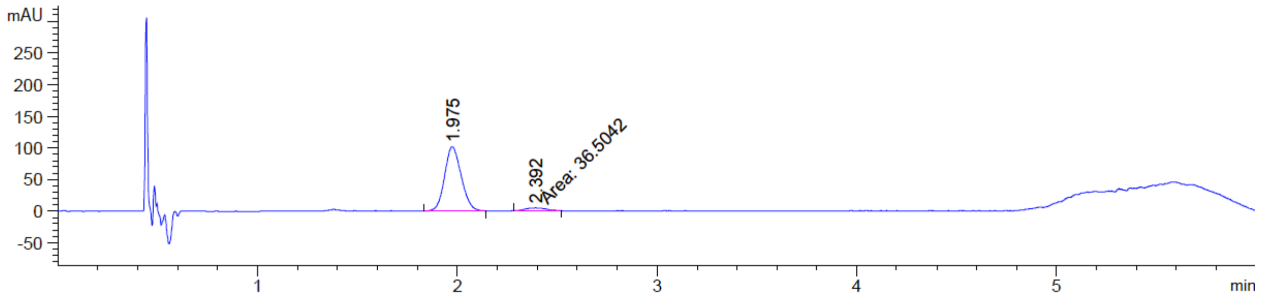

Peak #	RetTime [min]	Type	Width [min]	Area [mAU*s]	Height [mAU]	Area %
1	1.975	BB	0.0881	588.45563	101.52404	94.1589
2	2.392	MM	0.1286	36.50423	4.72922	5.8411

***Rac*-3ad**

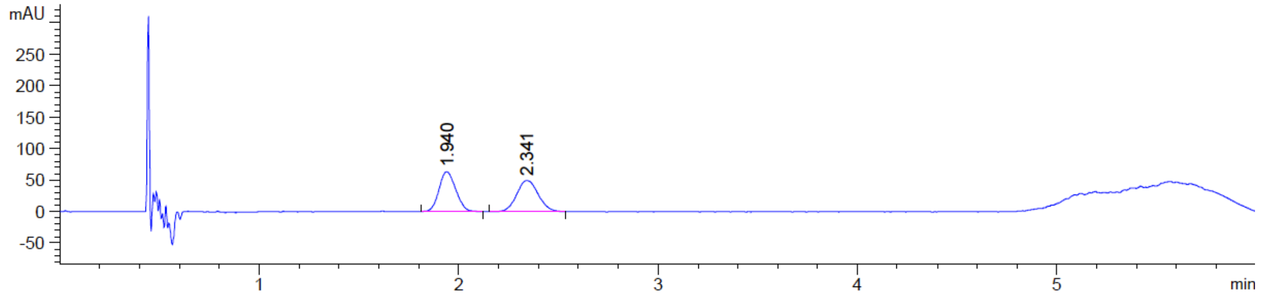

Peak #	RetTime [min]	Type	Width [min]	Area [mAU*s]	Height [mAU]	Area %
1	1.940	BV R	0.0873	376.99121	63.50263	50.0124
2	2.341	VV R	0.1107	376.80499	49.85580	49.9876

(*S*)-3-(3-Benzoylquinolin-4-yl)pyridin-2-yl trifluoromethanesulfonate (3ae)

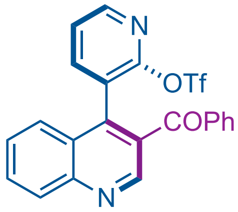

This compound was synthesized following the general procedure **D** at -20 °C using **1ad** (34.2 mg, 0.1 mmol) and **2c-H** (29.2 mg, 0.2 mmol). Purification by flash column chromatography (hexane/ethyl acetate: 6/1) provided the title compound as pale yellow oil (27.9 mg, 61% yield,

18% ee). Enantiomeric excess was determined by SFC analysis on a chiral stationary phase (CHIRALPAK IB-3, 1 mL/min, 10% methanol, λ = 210 nm, t_r(major) = 1.364 min, t_r(minor) = 1.562 min).

$[\alpha]_D^{23}$ = -7.7 (c = 0.13, $CHCl_3$).

^{1}H NMR (400 MHz, $CDCl_3$) δ 9.09 (s, 1H), 8.40 (dd, *J* = 4.9, 1.9 Hz, 1H), 8.29 (d, *J* = 8.5 Hz, 1H), 7.96 – 7.80 (m, 2H), 7.70 (d, *J* = 7.7 Hz, 2H), 7.66 – 7.54 (m, 2H), 7.53 – 7.34 (m, 4H).

^{13}C NMR (101 MHz, $CDCl_3$) δ 195.1, 153.0, 149.0, 148.8, 143.0, 139.6, 136.7, 134.1, 131.7, 131.6, 130.3, 130.0, 128.8 (2C), 128.6, 126.0, 125.9, 123.5, 122.4, 118.2 (q, *J* = 322.2 Hz).

^{19}F NMR (376 MHz, $CDCl_3$) δ -73.49.

HRMS: (ESI) calculated for $C_{22}H_{14}F_3N_2O_4S$ $[M+H]^+$ m/z: 459.0621, found: 459.0619.

3ae

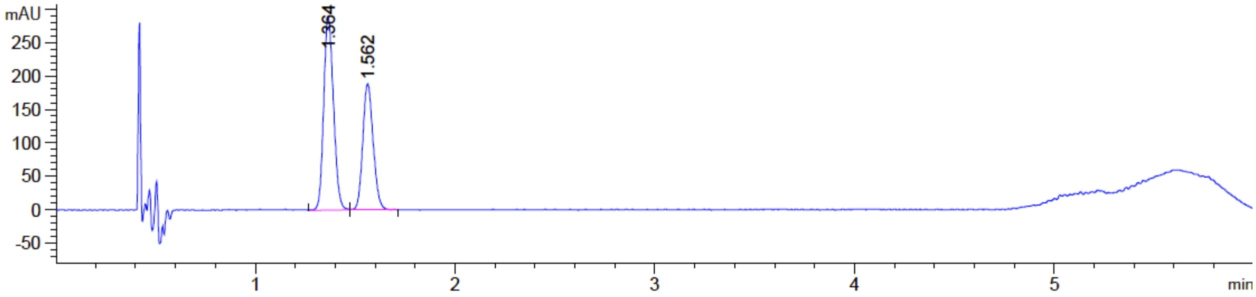

Peak #	RetTime [min]	Type	Width [min]	Area [mAU*s]	Height [mAU]	Area %
1	1.364	VV R	0.0533	990.70410	290.57950	59.0371
2	1.562	VV R	0.0559	687.40015	188.92717	40.9629

***Rac*-3ae**

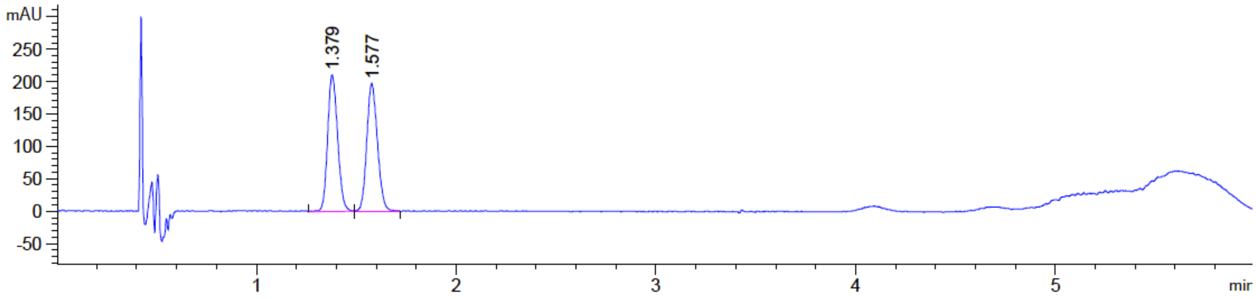

Peak #	RetTime [min]	Type	Width [min]	Area [mAU*s]	Height [mAU]	Area %
1	1.379	VB R	0.0545	731.00397	210.33284	49.9425
2	1.577	BV R	0.0584	732.68604	197.13857	50.0575

(*R*)-1-(3-(4-Methoxybenzoyl)-2,3-dihydroquinolin-4-yl)naphthalen-2-yl trifluoromethanesulfonate (3af)

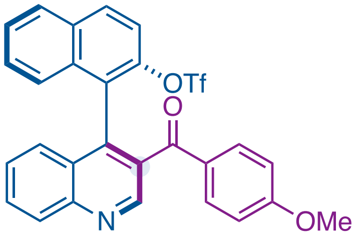

This compound was synthesized following the general procedure **D** using **1b** (39.1 mg, 0.1 mmol) and **2d-H** (35.2 mg, 0.2 mmol). Purification by flash column chromatography (hexane/ethyl acetate: 5/1) provided the title compound as white foam (33.0 mg, 61% yield, 98% ee). Enantiomeric excess was determined by SFC analysis on a chiral stationary phase (CHIRALPAK IG-3, 1 mL/min, 20% methanol, λ = 210 nm, t_r(major) = 2.011 min, t_r(minor) = 2.482 min).

$[\alpha]_D^{23}$ = -14.1 (c = 0.075, $CHCl_3$).

^{1}H NMR (400 MHz, $CDCl_3$) δ 9.01 (s, 1H), 8.17 (d, *J* = 8.2 Hz, 1H), 7.86 (d, *J* = 9.1 Hz, 1H), 7.81 (d, *J* = 8.5 Hz, 1H), 7.70 (ddd, *J* = 8.4, 6.8, 1.4 Hz, 1H), 7.60 – 7.55 (m, 2H), 7.42 (ddd, *J* = 8.2, 6.8, 1.2 Hz, 1H), 7.36 – 7.23 (m, 3H), 7.20 – 7.05 (m, 2H), 6.74 – 6.66 (m, 2H), 3.70 (s, 3H).

^{13}C NMR (101 MHz, $CDCl_3$) δ 193.3, 164.1, 149.3, 148.8, 144.3, 140.3, 133.4, 133.1, 132.7, 132.1, 131.7, 131.3, 130.0, 129.6, 128.9, 128.5, 128.2, 128.0, 127.4, 127.3, 126.9, 126.3, 119.1, 118.2 (q, *J* = 320.1 Hz), 113.8, 55.7.

^{19}F NMR (376 MHz, $CDCl_3$) δ -74.64.

HRMS (ESI): calculated for $C_{28}H_{19}F_3NO_5S^+$ $[M+H]^+$ m/z: 538.0931, found 538.0920.

3af

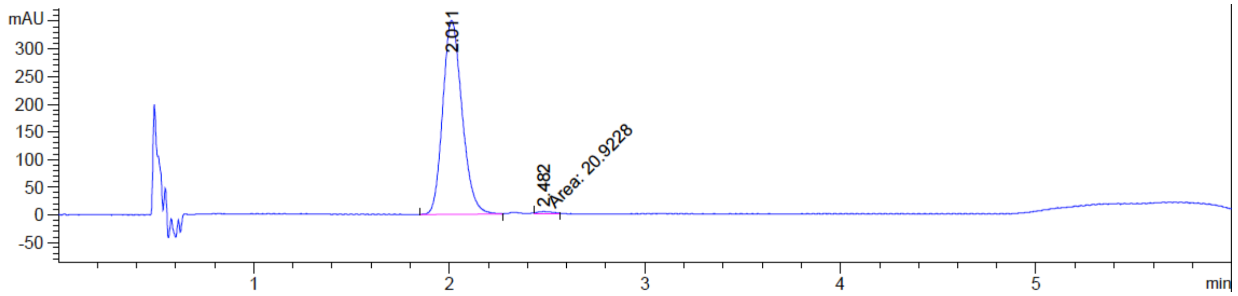

Peak #	RetTime [min]	Type	Width [min]	Area [mAU*s]	Height [mAU]	Area %
1	2.011	BV R	0.1053	2413.38330	350.77115	99.1405
2	2.482	MM	0.0906	20.92283	3.84907	0.8595

***Rac*-3af**

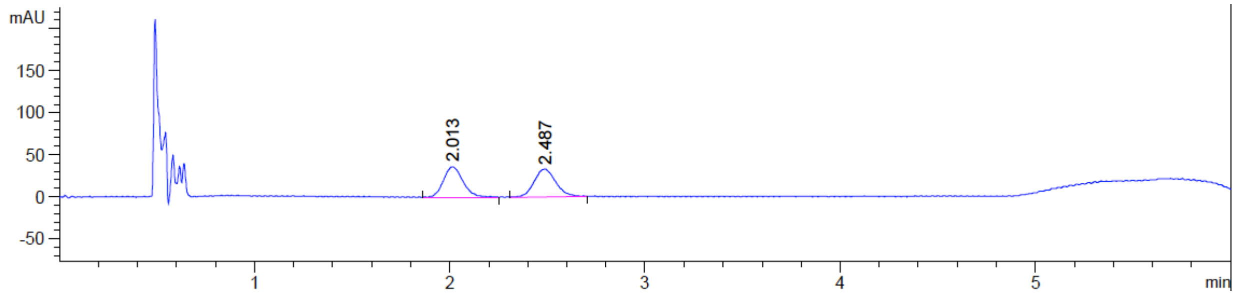

Peak #	RetTime [min]	Type	Width [min]	Area [mAU*s]	Height [mAU]	Area %
1	2.013	VV R	0.0963	254.39415	36.49666	49.7397
2	2.487	VV R	0.0970	257.05640	33.30662	50.2603

(*R*)-1-(3-(4-Methylbenzoyl)-2,3-dihydroquinolin-4-yl)naphthalen-2-yl trifluoromethanesulfonate (3ag)

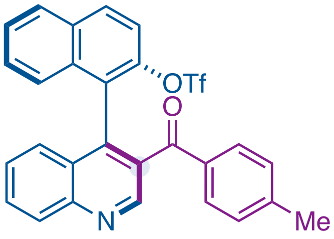

This compound was synthesized following the general procedure **D** using **1b** (39.1 mg, 0.1 mmol) and **2e-H** (32.0 mg, 0.2 mmol). Purification by flash column chromatography (hexane/ethyl

acetate: 5/1) provided the title compound as white foam (35.8 mg, 69% yield, 95% ee). Enantiomeric excess was determined by SFC analysis on a chiral stationary phase (CHIRALPAK IA-3, 1 mL/min, 15% methanol, λ = 210 nm, t_r(major) = 1.716 min, t_r(minor) = 1.990 min).

$[\alpha]_D^{23}$ = +22.0 (c = 0.09, $CHCl_3$).

^{1}H NMR (400 MHz, $CDCl_3$) δ 9.16 (s, 1H), 8.31 (d, *J* = 8.3 Hz, 1H), 8.00 (d, *J* = 9.1 Hz, 1H), 7.94 (d, *J* = 8.3 Hz, 1H), 7.84 (t, *J* = 7.7 Hz, 1H), 7.64 – 7.52 (m, 3H), 7.50 – 7.37 (m, 3H), 7.35 – 7.21 (m, 2H), 7.16 (d, *J* = 8.0 Hz, 2H), 2.37 (s, 3H).

^{13}C NMR (126 MHz, $CDCl_3$) δ 194.4, 149.4, 148.8, 144.7, 144.2, 140.6, 134.2, 133.3, 132.8, 132.0, 131.7, 131.5, 130.4, 130.0, 129.2 (2C), 128.5, 128.2, 128.1, 127.4, 127.3, 126.8, 126.2, 119.1, 118.2 (q, *J* = 320.2 Hz), 21.8.
^{19}F NMR (376 MHz, $CDCl_3$) δ -74.54.

HRMS (ESI): calculated for $C_{28}H_{18}F_3NNaO_4S^+$ $[M+Na]^+$ m/z: 544.0801, found 544.0801.

3ag

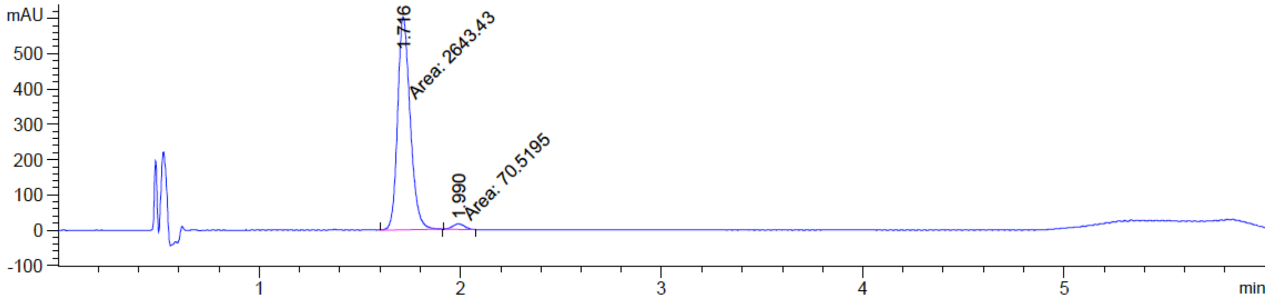

Peak #	RetTime [min]	Type	Width [min]	Area [mAU*s]	Height [mAU]	Area %
1	1.716	MM	0.0728	2643.43140	605.04675	97.4016
2	1.990	MM	0.0729	70.51954	16.12853	2.5984

***Rac*-3ag**

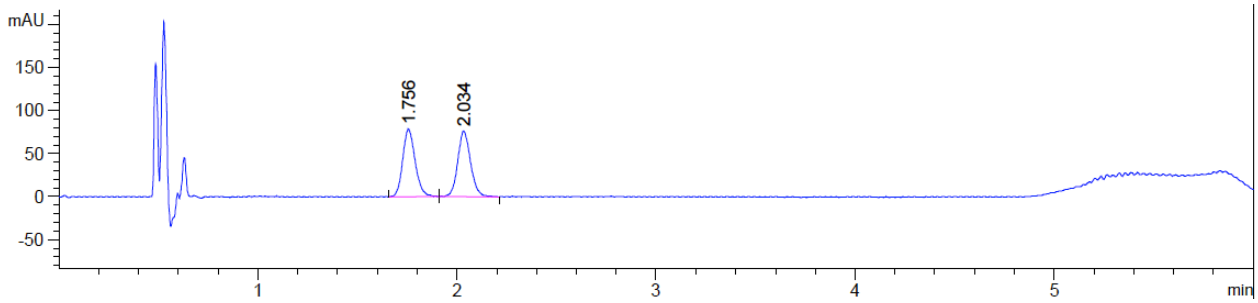

Peak #	RetTime [min]	Type	Width [min]	Area [mAU*s]	Height [mAU]	Area %
1	1.756	BV R	0.0672	342.25186	78.69613	49.8643
2	2.034	VV R	0.0711	344.11533	76.26438	50.1357

(*R*)-1-(3-(4-Chlorobenzoyl)-2,3-dihydroquinolin-4-yl)naphthalen-2-yl trifluoromethanesulfonate (3ah)

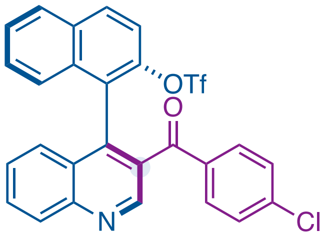

This compound was synthesized following the general procedure **D** using **1b** (39.1 mg, 0.1 mmol) and **2f-H** (36.1 mg, 0.2 mmol). Purification by flash column chromatography (hexane/ethyl acetate: 5/1) provided the title compound as white foam (39.2 mg, 72% yield, 93% ee). Enantiomeric excess was determined by SFC analysis on a chiral stationary phase (CHIRALPAK IA-3, 1 mL/min, 15% methanol, λ = 210 nm, t_r(major) = 1.840 min, t_r(minor) = 2.081 min).

$[\alpha]_D^{23}$ = +20.8 (c = 0.065, $CHCl_3$).

^{1}H NMR (500 MHz, $CDCl_3$) δ 9.15 (s, 1H), 8.31 (d, *J* = 8.1 Hz, 1H), 7.86 (ddd, *J* = 8.4, 6.8, 1.4 Hz, 1H), 7.64 – 7.61 (m, 2H), 7.57 (ddd, *J* = 8.2, 6.9, 1.2 Hz, 1H), 7.47 (ddd, *J* = 8.3, 6.8, 1.2 Hz, 1H), 7.47 (ddd, *J* = 8.3, 6.8, 1.3 Hz, 1H), 7.43 (ddd, *J* = 8.3, 6.8, 1.3 Hz, 1H), 7.41 (d, *J* = 9.1 Hz, 1H), 7.37 – 7.27 (m, 3H), 7.22 (d, *J* = 8.5 Hz, 1H).

^{13}C NMR (126 MHz, $CDCl_3$) δ 193.8, 149.2, 149.1, 144.2, 140.8, 140.1, 135.1, 133.2, 132.11, 132.06, 131.9, 131.7, 131.3, 130.1, 128.8, 128.6, 128.4, 128.3, 127.5, 127.4, 127.2, 126.7, 126.0, 119.1, 116.9 (q, J = 319.5 Hz).

^{19}F NMR (471 MHz, $CDCl_3$) δ -74.48.

HRMS (ESI): calculated for $C_{27}H_{15}ClF_3NNaO_4S^+$ $[M+Na]^+$ m/z: 564.0255, found 564.0266.

3ah

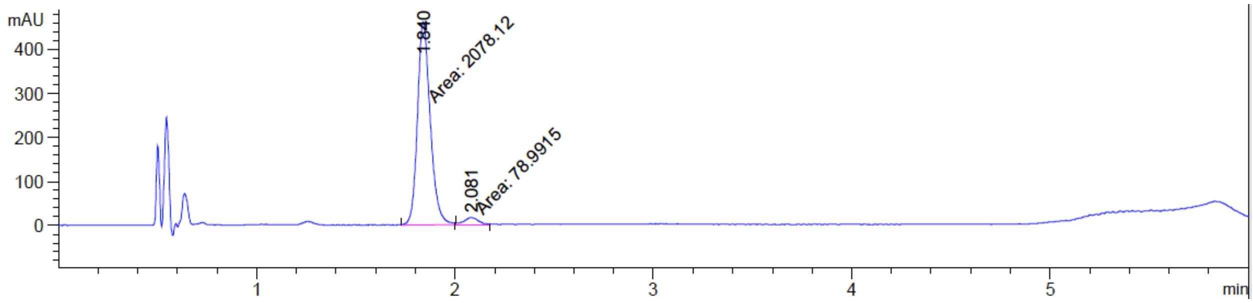

Peak #	RetTime [min]	Type	Width [min]	Area [mAU*s]	Height [mAU]	Area %
1	1.840	MM	0.0750	2078.11523	461.87903	96.3381
2	2.081	MM	0.0844	78.99150	15.60477	3.6619

***Rac*-3ah**

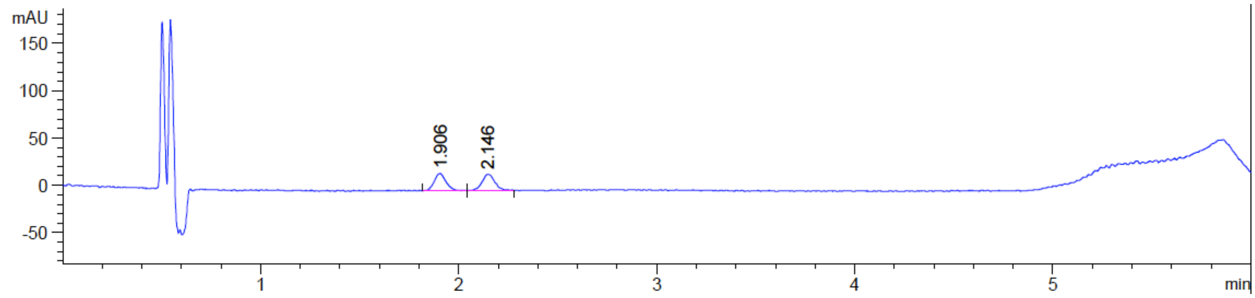

Peak #	RetTime [min]	Type	Width [min]	Area [mAU*s]	Height [mAU]	Area %
1	1.906	VV R	0.0643	76.92419	18.19674	49.5931
2	2.146	VV R	0.0602	78.18642	16.98368	50.4069

(*R*)-1-(3-(4-(Trifluoromethyl)benzoyl)-2,3-dihydroquinolin-4-yl)naphthalen-2-yl trifluoromethanesulfonate (3ai)

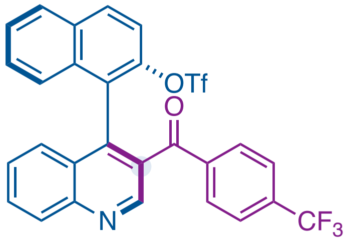

This compound was synthesized following the general procedure **D** using **1b** (39.1 mg, 0.1 mmol) and **2g-H** (42.8 mg, 0.2 mmol). Purification by flash column chromatography (hexane/ethyl acetate: 5/1) provided the title compound as white foam (38.9 mg, 68% yield, 87% ee). Enantiomeric excess was determined by SFC analysis on a chiral stationary phase (CHIRALPAK IA-3, 1 mL/min, 10% methanol, λ = 210 nm, t_r(major) = 1.206 min, t_r(minor) = 1.435 min).

$[\alpha]_D^{23}$ = +24.6 (c = 0.12, $CHCl_3$).

^{1}H NMR (400 MHz, $CDCl_3$) δ 9.18 (s, 1H), 8.33 (d, *J* = 8.3 Hz, 1H), 7.98 (d, *J* = 9.2 Hz, 1H), 7.93 (d, *J* = 8.3 Hz, 1H), 7.87 (ddd, *J* = 8.4, 6.8, 1.4 Hz, 1H), 7.73 – 7.66 (m, 2H), 7.57 (ddd, *J* = 8.2, 6.9, 1.2 Hz, 1H), 7.54 – 7.32 (m, 6H), 7.24 – 7.19 (m, 1H).

^{13}C NMR (101 MHz, $CDCl_3$) δ 194.3, 149.23, 149.20, 144.3, 141.0, 139.8, 134.4 (d, *J* = 32.9 Hz), 133.1, 132.1, 132.02, 131.98, 131.8, 130.1, 129.8, 128.6, 128.44, 128.39, 127.6, 127.4, 127.1, 126.6, 125.9, 125.4 (d, *J* = 3.8 Hz), 123.5 (q, *J* = 272.8 Hz), 119.8 (q, *J* = 320.8 Hz), 119.1.

^{19}F NMR (376 MHz, $CDCl_3$) δ -63.38, -74.55.

HRMS (ESI): calculated for $C_{28}H_{18}F_3NNaO_4S^+$ $[M+Na]^+$ m/z: 544.0801, found 544.0792.

3ai

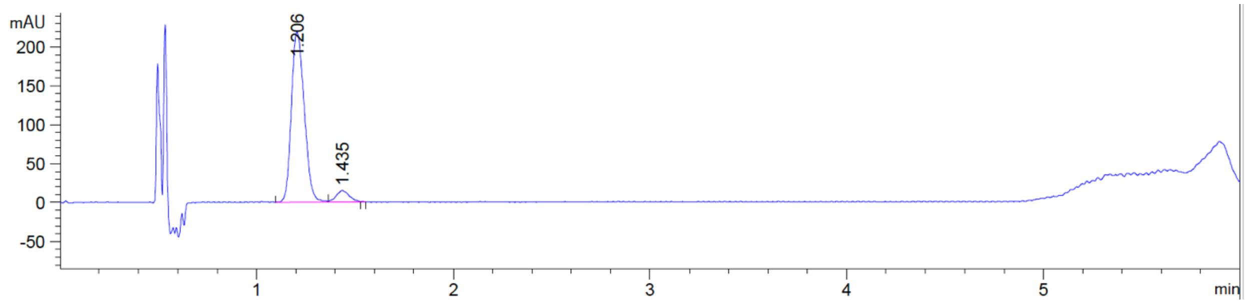

Peak #	RetTime [min]	Type	Width [min]	Area [mAU*s]	Height [mAU]	Area %
1	1.206	BV R	0.0732	998.15906	218.97560	93.5903
2	1.435	VV E	0.0611	68.36052	14.74771	6.4097

***Rac*-3ai**

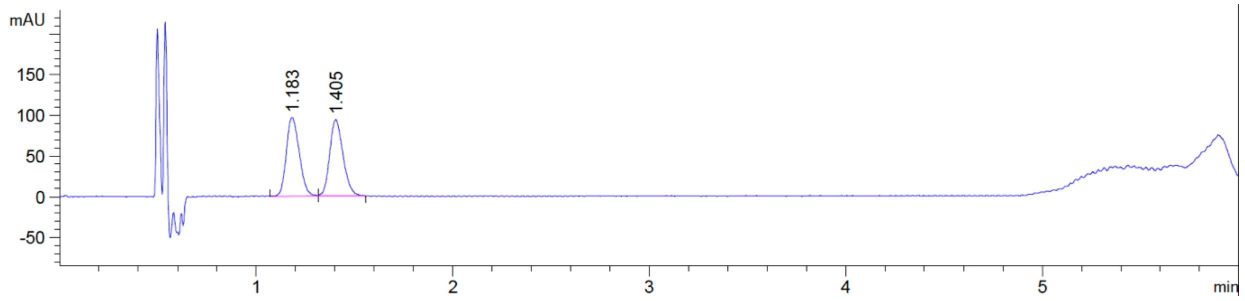

Peak #	RetTime [min]	Type	Width [min]	Area [mAU*s]	Height [mAU]	Area %
1	1.183	VV R	0.0735	443.88971	96.73754	49.8992
2	1.405	VV R	0.0742	445.68228	94.11736	50.1008

(*R*)-1-(3-(Benzo[*d*][1,3]dioxole-5-carbonyl)-2,3-dihydroquinolin-4-yl)naphthalen-2-yl trifluoromethanesulfonate (3aj)

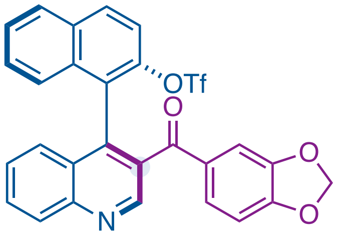

This compound was synthesized following the general procedure **D** using **1b** (39.1 mg, 0.1 mmol) and **2h-H** (38.0 mg, 0.2 mmol). Purification by flash column chromatography (hexane/ethyl acetate: 5/1) provided the title compound as white foam (45.9 mg, 83% yield, 94% ee). Enantiomeric excess was determined by SFC analysis on a chiral stationary phase (CHIRALPAK IG-3, 1 mL/min, 20% methanol, λ = 210 nm, t_r(major) = 2.091 min, t_r(minor) = 2.529 min).

$[\alpha]_D^{23}$ = -8.9 (c = 0.065, $CHCl_3$).

^{1}H NMR (400 MHz, $CDCl_3$) δ 9.14 (s, 1H), 8.30 (d, *J* = 8.5 Hz, 1H), 8.01 (d, *J* = 9.1 Hz, 1H), 7.95 (d, *J* = 8.3 Hz, 1H), 7.83 (ddd, *J* = 8.4, 6.8, 1.4 Hz, 1H), 7.56 (ddd, *J* = 8.2, 6.9, 1.2 Hz, 1H), 7.48 – 7.37 (m, 3H), 7.33 – 7.17 (m, 4H), 6.73 (d, *J* = 8.1 Hz, 1H), 6.01 (dd, *J* = 6.6, 1.2 Hz, 2H).

^{13}C NMR (101 MHz, $CDCl_3$) δ 192.9, 152.5, 149.1, 148.7, 148.3, 144.3, 140.4, 133.3, 133.0, 132.1, 131.7, 131.44, 131.42, 130.0, 128.5, 128.3, 128.1, 127.8, 127.43, 127.36, 127.3, 126.8, 126.1, 119.1, 118.2 (q, *J* = 320.2 Hz), 109.2, 107.8, 102.1.

^{19}F NMR (376 MHz, $CDCl_3$) δ -74.64.

HRMS (ESI): calculated for $C_{28}H_{17}F_3NO_6S^+$ $[M+H]^+$ m/z: 552.0723, found 552.0739.

3aj

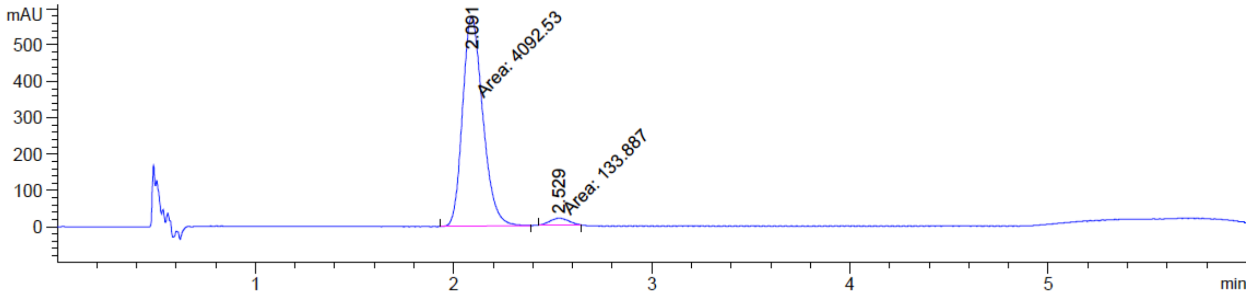

Peak #	RetTime [min]	Type	Width [min]	Area [mAU*s]	Height [mAU]	Area %
1	2.091	MM	0.1183	4092.53320	576.67609	96.8321
2	2.529	MM	0.1150	133.88693	19.40493	3.1679

***Rac*-3aj**

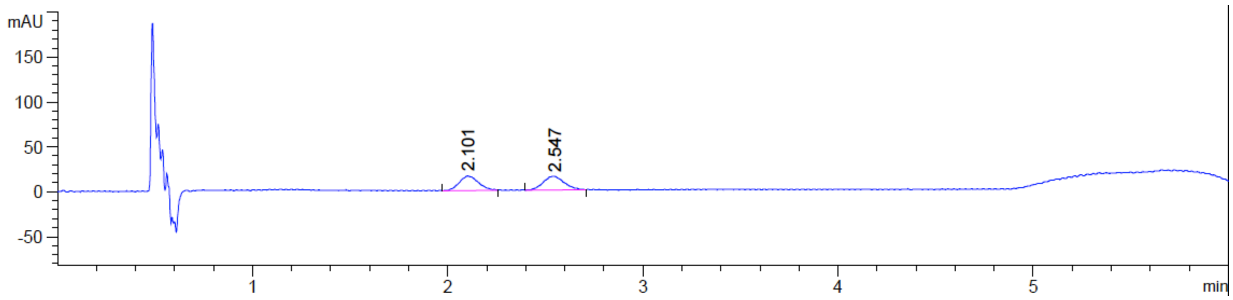

Peak #	RetTime [min]	Type	Width [min]	Area [mAU*s]	Height [mAU]	Area %
1	2.101	VV R	0.0864	110.35993	16.12281	49.2460
2	2.547	VV R	0.0943	113.73916	15.11539	50.7540

(*R*)-1-(3-(2-Methylbenzoyl)-2,3-dihydroquinolin-4-yl)naphthalen-2-yl trifluoromethanesulfonate (3ak)

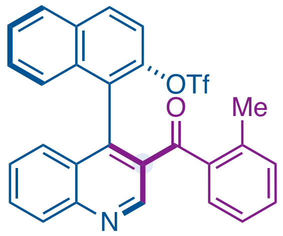

This compound was synthesized following the general procedure **D** using **1b** (39.1 mg, 0.1 mmol) and **2i-H** (32.0 mg, 0.2 mmol). Purification by flash column chromatography (hexane/ethyl acetate: 5/1) provided the title compound as white foam (18.9 mg, 36% yield, 99% ee). Enantiomeric excess was determined by SFC analysis on a chiral stationary phase (CHIRALPAK IA-3, 1 mL/min, 15% methanol, λ = 210 nm, t_r(major) = 1.541 min, t_r(minor) = 1.700 min).

$[\alpha]_D^{23}$ = +18.6 (c = 0.11, $CHCl_3$).

^{1}H NMR (400 MHz, $CDCl_3$) δ 9.20 (s, 1H), 8.30 (dt, *J* = 8.5, 1.0 Hz, 1H), 7.95 – 7.87 (m, 2H), 7.84 (ddd, *J* = 8.4, 6.8, 1.4 Hz, 1H), 7.54 (ddd, *J* = 8.2, 6.9, 1.2 Hz, 1H), 7.46 (ddd, *J* = 8.2, 6.8, 1.3 Hz, 1H), 7.40 (ddd, *J* = 8.3, 6.8, 1.3 Hz, 1H), 7.38 – 7.29 (m, 2H), 7.22 – 7.09 (m, 3H), 6.98 (d, *J* = 7.6 Hz, 1H), 6.88 (t, *J* = 7.5 Hz, 1H) 2.09 (s, 3H).

^{13}C NMR (101 MHz, $CDCl_3$) δ 197.3, 150.2, 149.1, 144.3, 140.2, 138.1, 137.3, 133.3, 132.8, 132.0, 131.7, 131.6, 131.3, 130.0, 128.5, 128.21, 128.16, 127.4, 127.3, 126.9, 126.7, 125.9, 125.1, 118.9, 118.2 (q, *J* = 320.1 Hz), 20.0. (1 missing signal due to aromatic overlap)

^{19}F NMR (376 MHz, $CDCl_3$) δ -74.73.

HRMS (ESI): calculated for $C_{28}H_{18}F_3NNaO_4S^+$ $[M+Na]^+$ m/z: 544.0801, found 544.0792.

3ak

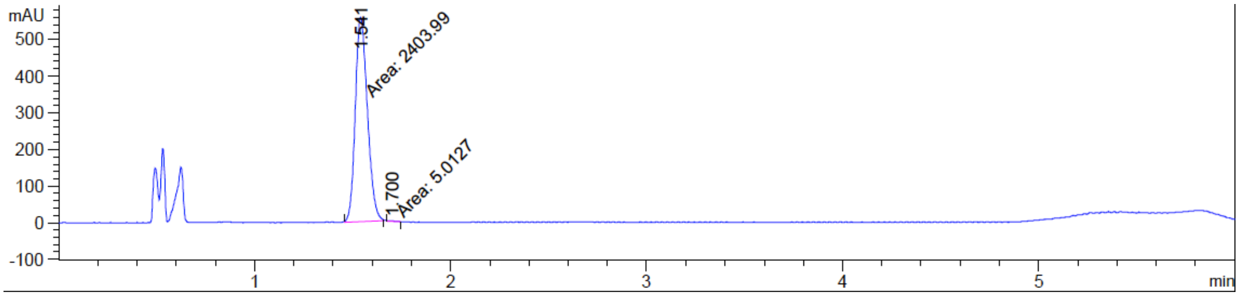

Peak #	RetTime [min]	Type	Width [min]	Area [mAU*s]	Height [mAU]	Area %
1	1.541	MM	0.0718	2403.99414	557.65247	99.7919
2	1.700	MM	0.0478	5.01270	1.74926	0.2081

***Rac*-3ak**

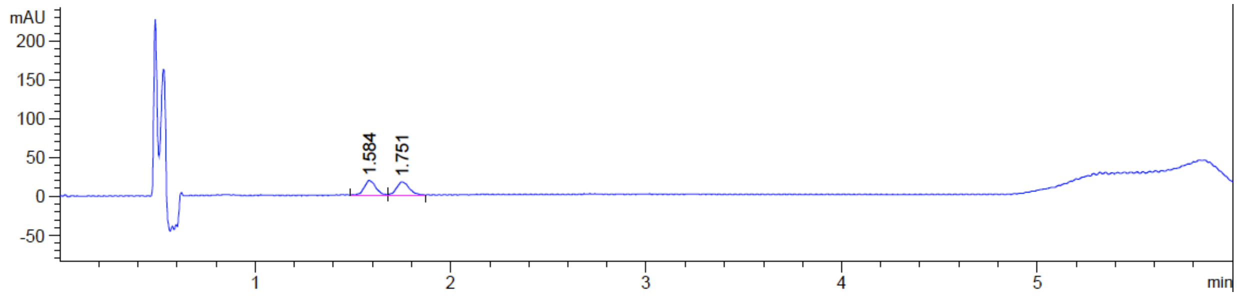

Peak #	RetTime [min]	Type	Width [min]	Area [mAU*s]	Height [mAU]	Area %
1	1.584	VV R	0.0562	80.19260	19.21757	50.3655
2	1.751	VV R	0.0603	79.02866	17.29107	49.6345

(*S*)-1-(3-(2-naphthoyl)quinolin-4-yl)naphthalen-2-yl trifluoromethanesulfonate (3al)

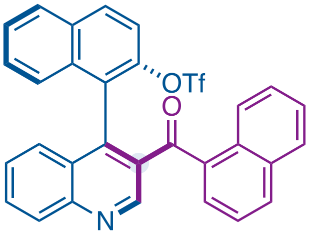

This compound was synthesized following the general procedure **D** using **1b** (39.1 mg, 0.1 mmol) and **2j-H** (39.2 mg, 0.2 mmol). Purification by flash column chromatography (hexane/ethyl acetate: 5/1) provided the title compound as white solid (23.9 mg, 43% yield, 99% ee).

Enantiomeric excess was determined by SFC analysis on a chiral stationary phase (CHIRALPAK IA-3, 1 mL/min, 15% methanol, λ = 210 nm, t_r(major) = 2.597 min, t_r(minor) = 2.875 min).

$[\alpha]_D^{23}$ = +8.6 (c = 0.06, $CHCl_3$).

^{1}H NMR (400 MHz, $CDCl_3$) δ 9.34 (s, 1H), 8.32 (dd, *J* = 8.3, 0.9 Hz, 1H), 8.01 (dd, *J* = 8.6, 1.1 Hz, 1H), 7.85 (ddd, *J* = 8.4, 6.8, 1.4 Hz, 1H), 7.76 – 7.63 (m, 3H), 7.58 (d, *J* = 9.1 Hz, 1H), 7.55 – 7.28 (m, 7H), 7.16 (dd, *J* = 8.5, 1.1 Hz, 1H), 7.08 – 6.99 (m, 2H).

^{13}C NMR (101 MHz, $CDCl_3$) δ 197.1, 150.1, 149.2, 144.2, 140.6, 135.1, 134.0, 133.4, 132.7, 132.3, 131.8, 131.8, 131.1, 130.0, 130.0, 129.2, 128.4, 128.2, 128.2, 127.8, 127.4, 127.3, 126.9, 126.6, 126.5, 125.7, 125.1, 123.8, 118.8, 118.2 (q, *J* = 319.9 Hz). (1 missing signal due to aromatic overlap)

^{19}F NMR (376 MHz, $CDCl_3$) δ -74.68.

HRMS (ESI): calculated for $C_{31}H_{18}F_3NNaO_4S^+$ $[M+Na]^+$ m/z: 580.0801, found 580.0789.

m.p. 181-182 °C.

3al

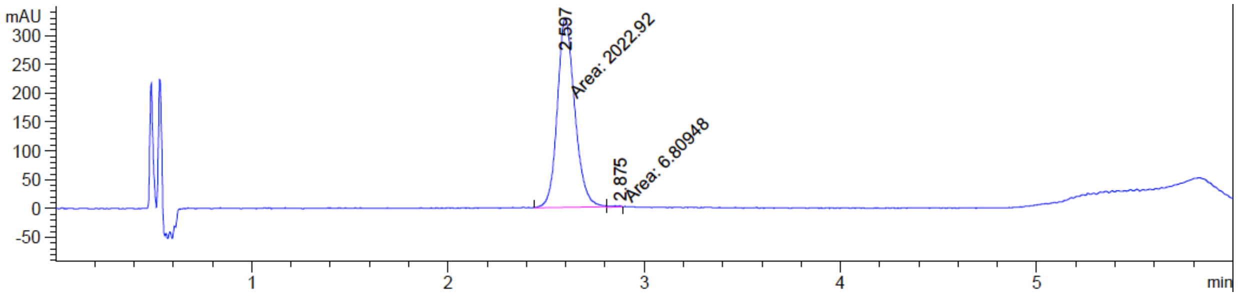

Peak #	RetTime [min]	Type	Width [min]	Area [mAU*s]	Height [mAU]	Area %
1	2.597	MM	0.1029	2022.91626	327.62097	99.6645
2	2.875	MM	0.0632	6.80948	1.79641	0.3355

***Rac*-3al**

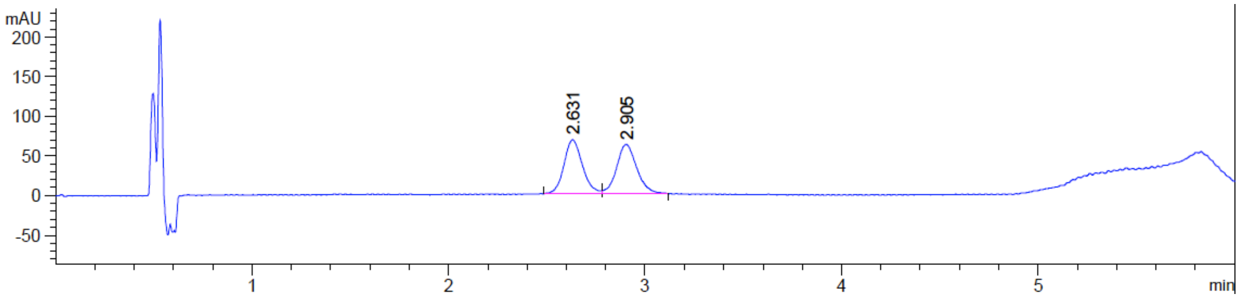

Peak #	RetTime [min]	Type	Width [min]	Area [mAU*s]	Height [mAU]	Area %
1	2.631	BV R	0.0978	445.99921	68.15250	49.9943
2	2.905	VV R	0.0995	446.10150	62.32846	50.0057

*Note 1: After 8 months of storage under air at room temperature, compound **3al** retained an enantiomeric excess of 99%.*

*Note 2: The absolute configuration of quinolines **3** was assigned by analogy to that of **3al**, which was determined by single-crystal X-ray diffraction analysis (See section 5).*

(*S*)-1-(3-(thiophene-2-carbonyl)quinolin-4-yl)naphthalen-2-yl trifluoromethanesulfonate (3am)

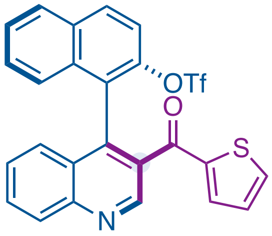

This compound was synthesized following the general procedure **D** using **1b** (39.1 mg, 0.1 mmol) and **2k-H** (30.4 mg, 0.2 mmol). Purification by flash column chromatography (hexane/ethyl acetate: 5/1) provided the title compound as white foam (20.0 mg, 39% yield, 40% ee). Enantiomeric excess was determined by SFC analysis on a chiral stationary phase (CHIRALPAK ID-3, 1 mL/min, 10% methanol, λ = 210 nm, t_r(major) = 3.129 min, t_r(minor) = 4.032 min).

$[\alpha]_D^{23}$ = -4.3 (c = 0.1, $CHCl_3$).

^{1}H NMR (500 MHz, $CDCl_3$) δ 9.31 (s, 1H), 8.32 (d, *J* = 8.5 Hz, 1H), 8.04 (d, *J* = 9.1 Hz, 1H), 7.99 – 7.93 (m, 1H), 7.84 (ddd, *J* = 8.5, 6.8, 1.4 Hz, 1H), 7.69 (dd, *J* = 4.9, 1.4 Hz, 1H), 7.59 –

7.52 (m, 2H), 7.49 – 7.37 (m, 3H), 7.32 – 7.26 (m, 1H), 7.23 (d, *J* = 8.5 Hz, 1H), 7.11 (dd, *J* = 4.9, 3.9 Hz, 1H).

^{13}C NMR (126 MHz, $CDCl_3$) δ 186.2, 148.9, 148.7, 144.1, 143.5, 140.3, 136.0, 135.5, 133.2, 132.4, 132.0, 131.7, 131.4, 129.9, 128.4, 128.3, 128.1, 128.0, 127.3,127.23, 127.21, 126.7, 125.8, 119.0, 116.8 (q, *J* = 320.1 Hz).

^{19}F NMR (471 MHz, $CDCl_3$) δ -74.59.

HRMS (ESI): calculated for $C_{25}H_{15}F_3NO_4S_2^+$ [M+H]$^+$ m/z: 514.0389, found 514.0395.

3am

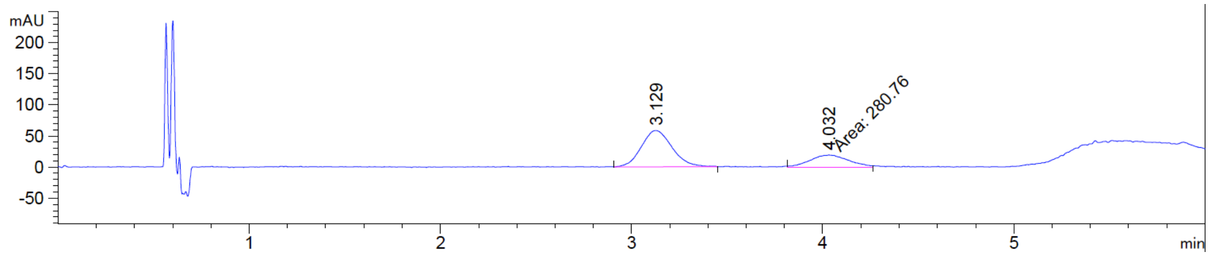

Peak #	RetTime [min]	Type	Width [min]	Area [mAU*s]	Height [mAU]	Area %
1	3.129	BV R	0.1346	656.23480	58.64022	70.0361
2	4.032	MM	0.2372	280.76038	19.73029	29.9639

***Rac*-3am**

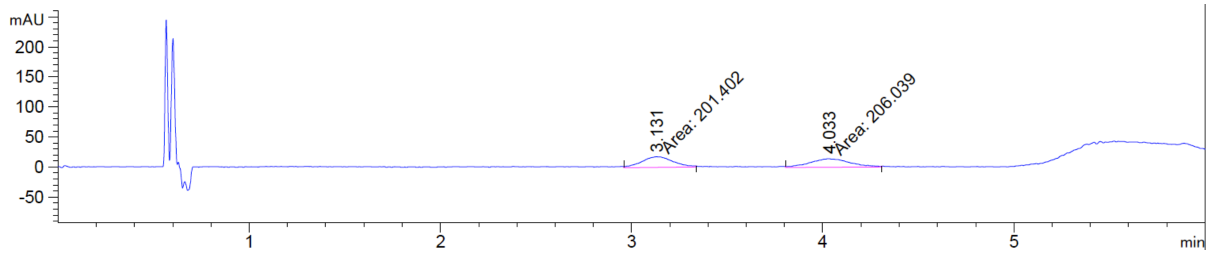

Peak #	RetTime [min]	Type	Width [min]	Area [mAU*s]	Height [mAU]	Area %
1	3.131	MM	0.1908	201.40201	17.59356	49.4310
2	4.033	MM	0.2437	206.03874	14.08847	50.5690

(*S*)-1-(3-acetylquinolin-4-yl)naphthalen-2-yl trifluoromethanesulfonate (3ap)

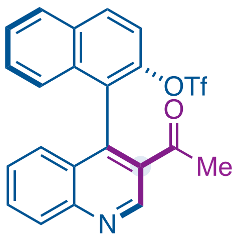

This compound was synthesized following the general procedure **D** using **1b** (39.1 mg, 0.1 mmol), **2l-H** (16.8 mg, 0.2 mmol). Purification by flash column chromatography (hexane/ethyl acetate: 4/1) provided the title compound as pale yellow oil (14.0 mg, 31% yield, 3% ee). Enantiomeric ratio was determined by SFC analysis on a chiral stationary phase (CHIRALPAK IG-3, 1 mL/min, 10% methanol, λ = 210 nm, t_r(major) = 2.935 min, t_r(minor) = 1.934 min.

$[\alpha]_D^{28}$ = +16.5 (c = 0.47, $CHCl_3$).

^{1}H NMR (300 MHz, $CDCl_3$) δ 9.45 (s, 1H), 8.28 (d, *J* = 8.4 Hz, 1H), 8.12 (d, *J* = 9.1 Hz, 1H), 8.02 (d, *J* = 7.9 Hz, 1H), 7.82 (ddd, *J* = 8.4, 6.8, 1.4 Hz, 1H), 7.65 – 7.52 (m, 2H), 7.48 – 7.34 (m, 2H), 7.22 (d, *J* = 8.5 Hz, 1H), 7.11 (d, *J* = 8.5 Hz, 1H), 2.34 (s, 3H).

^{13}C NMR (75 MHz, $CDCl_3$) δ 198.2, 149.6, 149.3, 144.0, 140.0, 133.0, 132.3, 131.9, 131.6, 131.3, 129.9, 128.7, 128.5, 128.2, 127.6, 127.4, 127.0, 126.8, 126.1, 119.5, 118.1 (q, *J* = 318.8 Hz), 29.4.

^{19}F NMR (282 MHz, $CDCl_3$) δ -74.59.

HRMS: (ESI) calculated for $C_{22}H_{15}F_3NO_4S$ $[M+H]^+$ m/z: 446.0668, found: 446.0664.

3ap

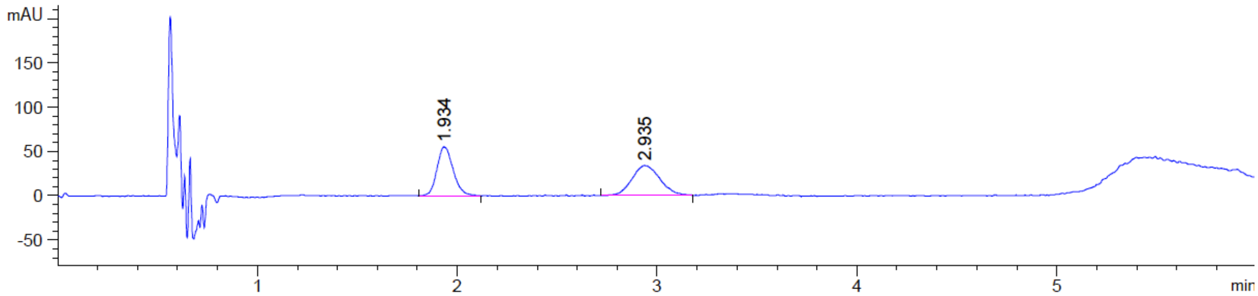

Peak #	RetTime [min]	Type	Width [min]	Area [mAU*s]	Height [mAU]	Area %
1	1.934	VV R	0.0864	312.37463	55.27186	48.4091
2	2.935	VV R	0.1225	332.90607	33.92661	51.5909

***Rac*-3ap**

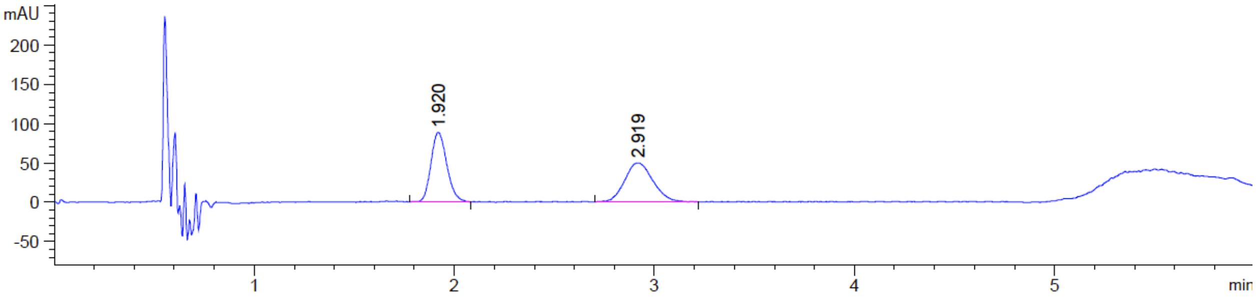

Peak #	RetTime [min]	Type	Width [min]	Area [mAU*s]	Height [mAU]	Area %
1	1.920	VB R	0.0851	491.62415	88.82359	49.7881
2	2.919	VV R	0.1229	495.80838	49.91874	50.2119

*Note: following the general procedure **D** using **1b** and the corresponding diazocompounds, the following products were not obtained.*

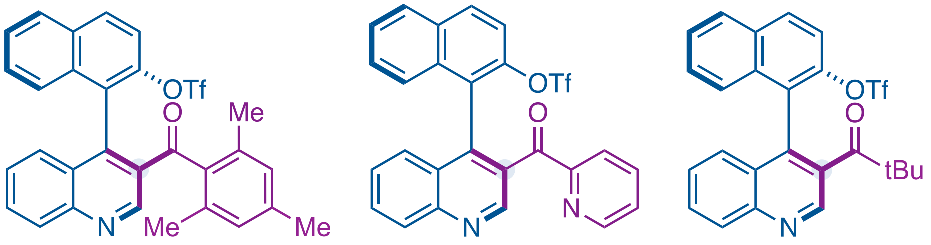

3an 0% **3ao** 0% **3aq** 0%

4. -OTf Control experiments

Below is the procedure used for the synthesis of 3-aryl indoles:

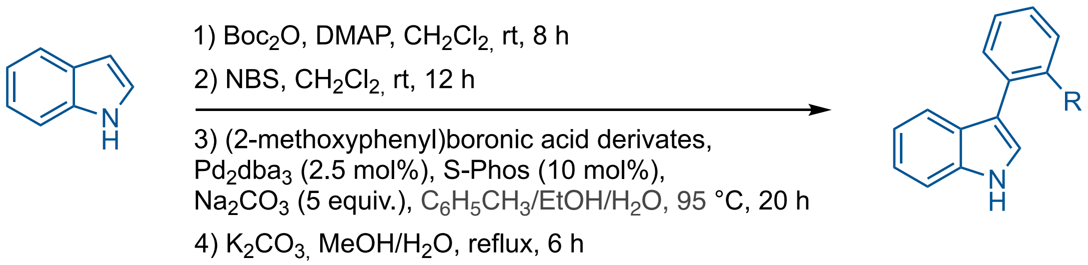

1) To a 100 mL round-bottom flask equipped with a stirring bar were added indole (10 mmol, 1.0 equiv.), Boc_2O (2.40 g, 11 mmol, 1.1 equiv.), 4-dimethylaminopyridine (122 mg, 1 mmol, 0.1 equiv.) and dichloromethane (20 mL). The reaction was stirred at room temperature for 8 hours. The reaction was quenched with water (50 mL) and extracted with dichloromethane (3 x 15 mL). The combined organic layer was dried over anhydrous Na_2SO_4 and the solvent was removed under *vacuum*. The crude compound was used directly for the next step without purification.
2) To a 100 mL round-bottom flask equipped with a stirring bar were added the corresponding *tert*-butyl 1*H*-indole-1-carboxylate (10 mmol, 1 equiv.), dichloromethane (20 mL) and *N*-bromosuccinimide (NBS, 1.96 g, 11 mmol, 1.1 equiv,). The reaction mixture was stirred at room temperature for 12 hours. Then, the reaction was quenched with water (50 mL) and the mixture was extracted with dichloromethane (3 x 15 mL). The combined organic layer was dried over anhydrous Na_2SO_4 and the solvent was removed under *vacuum*. The crude compound was used directly for the next step without purification.
3) To a 250 mL two-neck round-bottom flask equipped with a stirring bar were added the mixture of *tert*-butyl 3-bromo-1*H*-indole-1-carboxylate (2.96 g, 10 mmol, 1.0 equiv.), (2-methoxyphenyl)boronic acid derivates (11 mmol, 1.1 equiv.), Pd_2dba_3 (229 mg, 2.5 mol%), *S*-Phos (411 mg, 10 mol%) and Na_2CO_3 (5.30 g, 50 mmol, 5.0 equiv.). The flask was sealed before being evacuated and backfilled with argon three times. A mix of toluene (60 mL), ethanol (20 mL) and water (20 mL) was added and the reaction mixture was stirred at 95 °C for 20 hours under argon. After completion monitored by TLC, the reaction mixture was cooled to room temperature, quenched with water (150 mL) and extracted with ethyl acetate (3 x 50 mL). The combined organic layer was washed with brine and dried over anhydrous Na_2SO_4. The solvent was removed under *vacuum* and the crude was purified by flash column chromatography on silica gel to afford the corresponding *tert*-butyl 3-(2-methoxyphenyl)-1*H*-indole-1-carboxylate derivates.
4) To a 100 mL round-bottom flask equipped with a stirring bar were added the corresponding 3-(2-methoxyphenyl)-1*H*-indole derivatives (8 mmol, 1.0 equiv.), K_2CO_3 (3.32g, 24 mmol, 3.0 equiv.), methanol (40 mL) and water (8 mL). The reaction was heated at reflux for 6 hours. Then, the reaction mixture was cooled to room temperature, diluted with water (50 mL) and extracted with dichloromethane (3 x 20 mL). The combined organic layer was

dried over anhydrous Na_2SO_4 and the solvent was removed under *vacuum.* The crude was purified by flash column chromatography on silica gel to afford the compound 3-(aryl)-1*H*-indole derivatives

3-(*o*-tolyl)-1*H*-indole (S4)

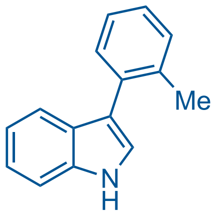

This compound was synthesized based on the general procedure **D** using (*o*-tolylboronic acid (1.50 g, 11.0 mmol). Purification by flash column chromatography (hexane/ethyl acetate = 20/1) provided the title compound as a colorless oil (1.66 g, 73% yield).

^{1}H NMR (400 MHz, $CDCl_3$) δ 8.19 (brs, 1H), 7.60 – 7.52 (m, 1H), 7.49 – 7.43 (m, 2H), 7.36 (ddt, J = 6.1, 4.3, 2.5 Hz, 1H), 7.32 – 7.24 (m, 3H), 7.22 – 7.12 (m, 2H), 2.36 (s, 1H).
^{13}C NMR (101 MHz, $CDCl_3$) δ 137.0, 136.0, 134.6, 131.0, 130.5, 127.3, 126.9, 125.8, 122.9, 122.3, 120.3, 120.1, 117.7, 111.3, 20.8.
These data are in accordance with the literature.[9]

3-(2-isopropylphenyl)-1*H*-indole (S5)

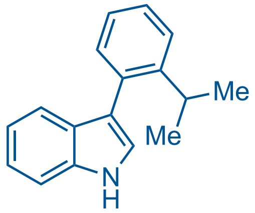

This compound was synthesized using (2-isopropylphenyl)boronic acid (1.80 g, 11.0 mmol). Purification by flash column chromatography (hexane/ethyl acetate = 20/1) provided the title compound as a colorless oil (2.10 g, 81% yield).

^{1}H NMR (400 MHz, $CDCl_3$) δ 8.20 (brs, 1H), 7.50 – 7.42 (m, 3H), 7.40 – 7.32 (m, 2H), 7.30 – 7.19 (m, 2H), 7.19 – 7.11 (m, 2H), 3.22 (hept, J = 6.9 Hz, 1H), 1.18 (d, J = 6.9 Hz, 6H).

^{13}C NMR (101 MHz, $CDCl_3$) δ 148.4, 135.9, 133.2, 131.6, 128.1, 127.6, 125.6, 125.5, 122.7, 122.3, 120.04, 119.99, 117.6, 111.2, 29.8, 24.5.

HRMS: (ESI) calculated for $C_{17}H_{18}N$ $[M+H]^+$ m/z: 236.1434, found: 236.1431.

3-([1,1'-biphenyl]-2-yl)-1*H*-indole (S6)

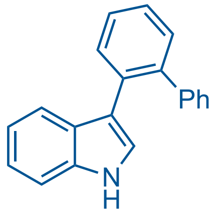

This compound was synthesized using [1,1'-biphenyl]-2-ylboronic acid (2.18 g, 11.0 mmol). Purification by flash column chromatography (hexane/ethyl acetate = 20/1) provided the title compound as a orange oil (1.43 g, 53% yield).

^{1}H NMR (400 MHz, $CDCl_3$) δ 7.94 (s, 1H), 7.76 – 7.58 (m, 2H), 7.54 – 7.38 (m, 3H), 7.34 (dt, J = 8.1, 1.0 Hz, 1H), 7.30 – 7.16 (m, 6H), 7.12 (ddd, J = 8.0, 7.0, 1.1 Hz, 1H), 6.64 (d, J = 2.5 Hz, 1H).

^{13}C NMR (101 MHz, $CDCl_3$) δ 142.5, 141.4, 135.9, 133.5, 131.0, 130.8, 129.7, 127.9, 127.5, 127.0, 126.8, 126.5, 124.1, 122.1, 120.0, 120.0, 116.8, 111.1.

These data are in accordance with the literature.[10]

2-(1*H*-indol-3-yl)benzaldehyde (S7)

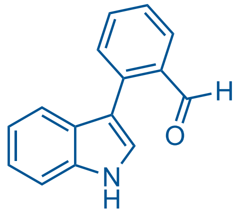

This compound was synthesized using (2-formylphenyl)boronic acid (1.65 g, 11.0 mmol). Purification by flash column chromatography (hexane/ethyl acetate = 20/1) provided the title compound as a orange oil (1.47 g, 60% yield).

^{1}H NMR (400 MHz, $CDCl_3$) δ 10.13 (s, 1H), 8.76 (brs, 1H), 8.13 – 8.06 (m, 1H), 7.73 – 7.61 (m, 3H), 7.52 – 7.42 (m, 2H), 7.34 – 7.17 (m, 3H).

^{13}C NMR (101 MHz, $CDCl_3$) δ 193.6, 139.1, 136.3, 134.4, 134.0, 131.5, 127.8, 127.4, 127.1, 125.3, 123.1, 121.0, 119.3, 113.7, 111.7.

These data are in accordance with the literature.[11]

2-(1*H*-indol-3-yl)benzonitrile (S8)

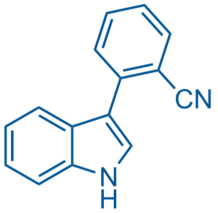

This compound was synthesized using (2-cyanophenyl)boronic acid (1.62 g, 11.0 mmol). Purification by flash column chromatography (hexane/ethyl acetate = 5/1) provided the title compound as a yellowish solid (489 mg, 20% yield).

^{1}H NMR (500 MHz, $CDCl_3$) δ 8.74 (brs, 1H), 7.84 – 7.78 (m, 3H), 7.70 – 7.62 (m, 2H), 7.45 (dd, *J* = 7.8, 1.1 Hz, 1H), 7.37 (td, *J* = 7.8, 1.1 Hz, 1H), 7.32 – 7.21 (m, 3H).

^{13}C NMR (126 MHz, $CDCl_3$) δ 139.2, 136.4, 134.0, 132.9, 130.0, 126.2, 125.8, 124.7, 122.8, 120.8, 119.9, 119.2, 113.7, 111.9, 110.7.

HRMS: (ESI) calculated for $C_{15}H_{10}N_2Na$ $[M+Na]^+$ m/z: 241.0736, found: 241.0733.

m.p. 142 – 143 °C

2-(1*H*-indol-2-yl)phenyl trifluoromethanesulfonate (S9)

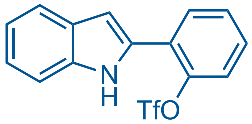

This compound was synthesized following the reported procedure.[12]

(*S*)-2-(3-benzoylquinolin-4-yl)benzaldehyde (7)

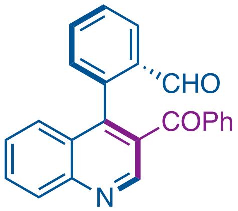

This compound was synthesized following the general procedure **D** at -20 °C using **S7** (22.1 mg, 0.1 mmol) and **2c-H** (29.2 mg, 0.2 mmol). Purification by flash column chromatography (hexane/ethyl acetate: 5/1) provided the title compound as orange oil (16.7 mg, 50% yield, 20% ee). Enantiomeric excess was determined by SFC analysis on a chiral stationary phase (CHIRALPAK IA-3, 1 mL/min, 20% methanol, λ = 210 nm, t_r(major) = 2.329 min, t_r(minor) = 3.574 min).

$[\alpha]_D^{23}$ = +1.5 (c = 0.2, $CHCl_3$).

^{1}H NMR (500 MHz, DMSO) δ 9.67 (s, 1H), 9.02 (s, 1H), 8.22 (d, *J* = 8.4 Hz, 1H), 7.96 – 7.87 (m, 2H), 7.70 – 7.52 (m, 6H), 7.45 – 7.27 (m, 4H).

^{13}C NMR (126 MHz, DMSO) δ 195.7, 191.4, 148.2, 147.6, 144.3, 136.9, 136.4, 134.4, 133.83, 133.76, 131.6, 131.1, 131.0, 129.6, 129.54, 129.51, 129.47, 128.6, 128.2, 126.6, 126.2.

HRMS: (ESI) calculated for $C_{23}H_{16}NO_2$ $[M+H]^+$ m/z: 338.1176, found: 338.1173.

Note: ***Rac-7*** *was not able to synthetize using $Rh_2(esp)_2$, $Rh_2(OAc)_4$ or $Rh_2(pivalate)_4$. Instead, we synthetize 7'using $Rh_2(R\text{-}Br\text{-}NTTL)_4$.*

7

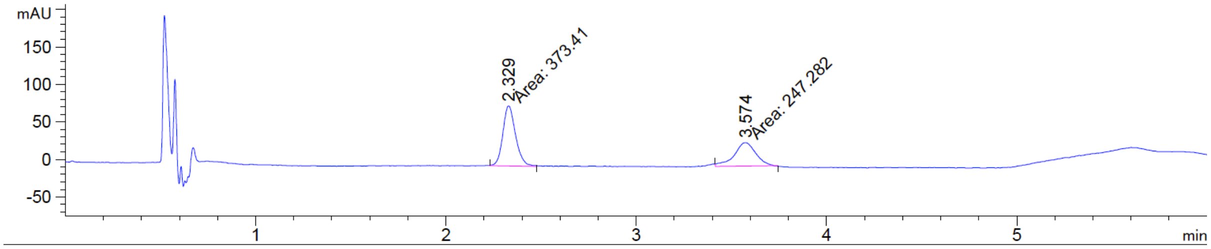

Peak #	RetTime [min]	Type	Width [min]	Area [mAU*s]	Height [mAU]	Area %
1	2.329	MM	0.0778	373.41037	80.02140	60.1603
2	3.574	MM	0.1321	247.28185	31.20930	39.8397

7'

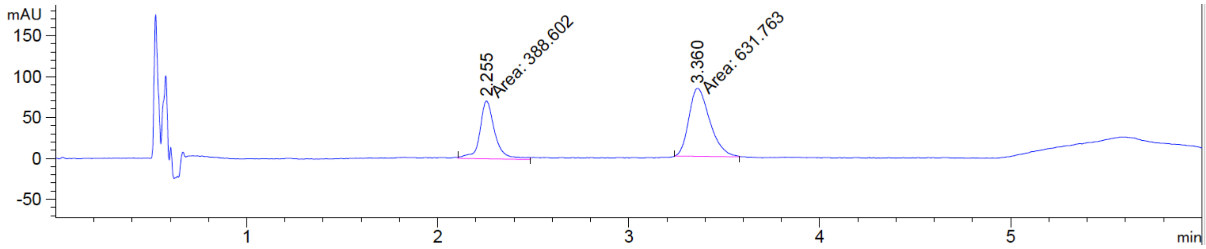

Peak #	RetTime [min]	Type	Width [min]	Area [mAU*s]	Height [mAU]	Area %
1	2.255	MM	0.0916	388.60159	70.69994	38.0846
2	3.360	MM	0.1268	631.76270	83.02669	61.9154

(*S*)-2-(3-benzoylquinolin-4-yl)benzonitrile (8)

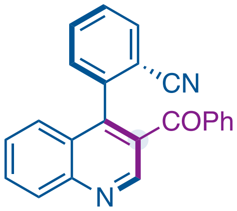

This compound was synthesized following the general procedure **D** at -20 °C using **S8** (21.8 mg, 0.1 mmol) and **2c-H** (29.2 mg, 0.2 mmol). Purification by flash column chromatography (hexane/ethyl acetate: 4/1) provided the title compound as orange oil (5.9 mg, 18% yield, 75% ee). Enantiomeric excess was determined by SFC analysis on a chiral stationary phase (CHIRALPAK IA-3, 1 mL/min, 30% methanol, λ = 210 nm, t_r(major) = 1.380 min, t_r(minor) = 1.614 min).

$[\alpha]_D^{23}$ = -54.4 (c = 0.12, $CHCl_3$).

^{1}H NMR (400 MHz, $CDCl_3$) δ 9.10 (s, 1H), 8.31 (d, *J* = 8.4 Hz, 1H), 7.88 (ddd, *J* = 8.4, 6.9, 1.4 Hz, 1H), 7.79 – 7.73 (m, 2H), 7.73 – 7.38 (m, 9H).

^{13}C NMR (101 MHz, $CDCl_3$) δ 195.7, 149.0, 148.96, 144.1, 139.4, 137.2, 133.8, 132.9, 132.6, 131.7, 131.39, 131.37, 130.3, 130.2, 129.1, 128.7, 128.4, 126.3, 126.2, 117.4, 113.3.

HRMS: (ESI) calculated for $C_{23}H_{15}N_2O$ $[M+H]^+$ m/z: 335.1179, found: 335.1178.

8

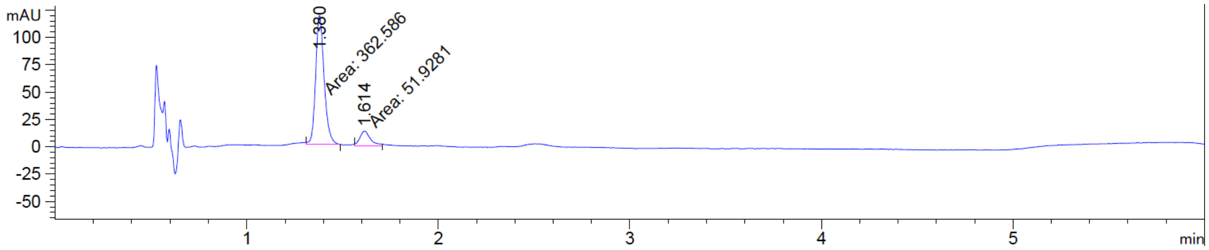

Peak #	RetTime [min]	Type	Width [min]	Area [mAU*s]	Height [mAU]	Area %
1	1.380	MM	0.0512	362.58603	117.93687	87.4725
2	1.614	MM	0.0643	51.92814	13.45780	12.5275

***Rac*-8**

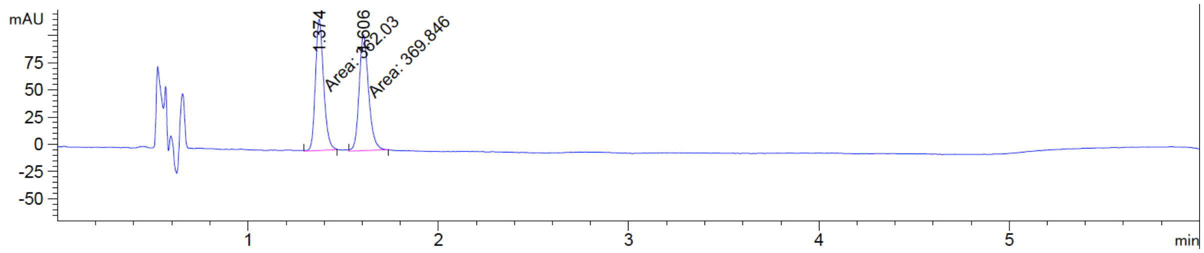

Peak #	RetTime [min]	Type	Width [min]	Area [mAU*s]	Height [mAU]	Area %
1	1.374	MM	0.0500	362.03018	120.63313	49.4661
2	1.606	MM	0.0578	369.84552	106.58469	50.5339

5. Synthesis of *iso*-QUINAP ligand 4 and applications

1-(3-Benzylquinolin-4-yl)naphthalen-2-yl trifluoromethanesulfonate (int-a)

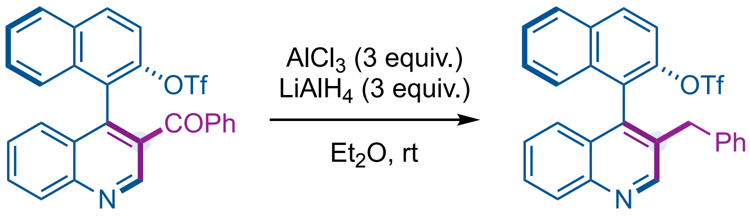

To a 10 mL round-bottom flask equipped with a stirring bar were added $LiAlH_4$ (22.8 mg, 0.6 mmol, 3 equiv.) and Et_2O (1 mL). Then $AlCl_3$ (80.0 mg, 0.6 mmol, 3 equiv.) dissolved in Et_2O (1 mL) was slowly added to the reaction mixture at room temperature. After gas releasing ceased about 10 minutes, **3c** (101.5 mg, 0.2 mmol, 1 equiv.) dissolved in Et_2O (1 mL) was added to the

reaction solution. The reaction mixture was stirred at room temperature for 1 hour, then poured into ice water (10 mL), extracted with dichloromethane (3 x 5 mL). The combined organic layer was washed with brine and dried over anhydrous Na_2SO_4. The solvent was removed under *vacuum* and the crude was purified by flash column chromatography on silica gel to afford the title compound as colourless oil (80.0 mg, 79% yield, 95% ee). Enantiomeric excess was determined by SFC analysis on a chiral stationary phase (CHIRALPAK IG-3, 1 mL/min, 10% ethanol, λ = 210 nm, t_r(major) = 2.868 min, t_r(minor) = 2.488 min.

$[\alpha]_D^{23}$ = +35.2 (c = 0.18, $CHCl_3$).

^{1}H NMR (400 MHz, $CDCl_3$) δ 8.97 (s, 1H), 8.20 (d, J = 9.0 Hz, 1H), 8.11 (d, J = 9.1 Hz, 1H), 7.98 (d, J = 8.3 Hz, 1H), 7.68 (ddd, J = 8.4, 6.8, 1.4 Hz, 1H), 7.62 – 7.49 (m, 2H), 7.40 – 7.27 (m, 2H), 7.14 (dd, J = 8.5, 1.3 Hz, 1H), 7.08 – 6.97 (m, 4H), 6.81 (dd, J = 6.9, 2.6 Hz, 2H), 3.90 (d, J = 15.6 Hz, 1H), 3.77 (d, J = 15.6 Hz, 1H).

^{13}C NMR (101 MHz, $CDCl_3$) δ 152.4, 147.1, 144.6, 138.7, 138.1, 133.4, 132.7, 132.4, 131.5, 129.7, 129.3, 129.0, 128.5, 128.4, 128.1, 127.51, 127.47, 127.3, 126.5, 126.4, 125.9, 119.5, 118.2 (q, J = 321.2 Hz), 37.6. (1 missing signal due to aromatic overlap)

^{19}F NMR (376 MHz, $CDCl_3$) δ -74.67.

HRMS: (ESI) calculated for $C_{27}H_{19}F_3NO_3S$ $[M+H]^+$ m/z: 494.1032, found: 494.1042.

Int-a

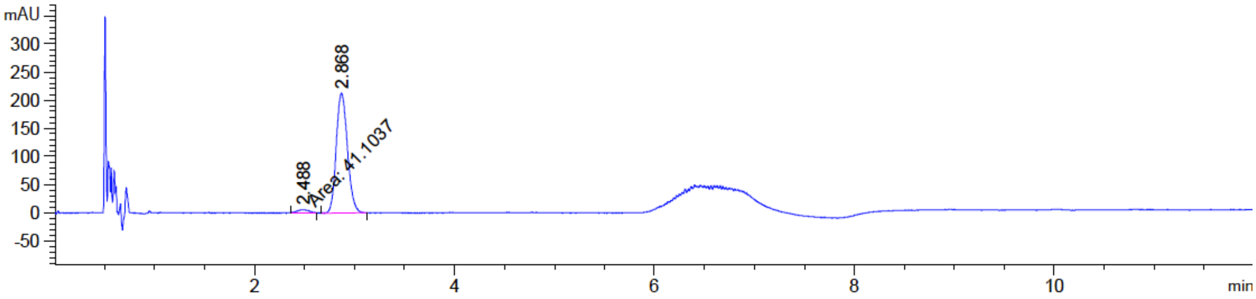

Peak #	RetTime [min]	Type	Width [min]	Area [mAU*s]	Height [mAU]	Area %
1	2.488	MM	0.1288	41.10368	5.31939	2.4150
2	2.868	VV R	0.1260	1660.89417	212.95723	97.5850

Rac-Int-a

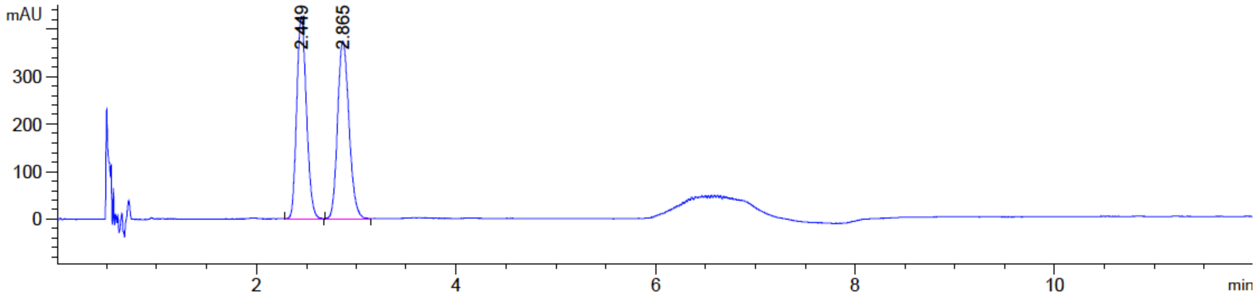

Peak #	RetTime [min]	Type	Width [min]	Area [mAU*s]	Height [mAU]	Area %
1	2.449	VB R	0.1065	2876.74829	425.01080	49.2111
2	2.865	BV R	0.1246	2968.97998	373.89725	50.7889

(1-(3-Benzylquinolin-4-yl)naphthalen-2-yl)diphenylphosphine oxide (int-b)

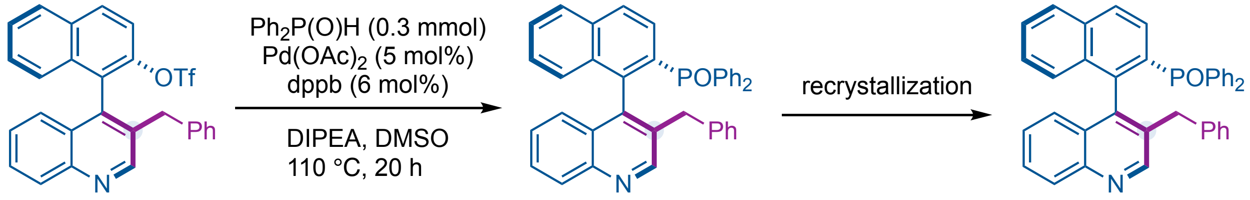

To a 10 mL oven-dried round-bottom flask equipped with a stirring bar were added **int-a** (98.7 mg, 0.2 mmol, 1 equiv.), diphenylphosphine oxide (60.7 mg, 0.3 mmol, 1.5 equiv.), $Pd(OAc)_2$ (2.2 mg, 0.01 mmol, 5 mol%) and dppp (5.2 mg, 0.012 mmol, 6 mol%). The flask was sealed before being evacuated and backfilled with argon three times. DIPEA (129.2 mg, 1 mmol, 5 equiv.) and anhydrous dimethyl sulfoxide (1.5 mL) were added and the reaction mixture was stirred at 110 °C for 20 hours. Then, the reaction was quenched with water (10 mL) and the mixture was extracted with ethyl acetate (3 ×10 mL). The combined organic layer was washed with brine and dried over anhydrous Na_2SO_4. The solvent was removed under *vacuum* and the crude was purified by flash column chromatography (Hexane/Ethyl acetate = 1:2) and recrystallized to provide the title compound as whith solid (86.8 mg, 94% yield, 99% ee). Enantiomeric excess was determined by SFC analysis on a chiral stationary phase (CHIRALPAK IA-3, 1 mL/min, 30% methanol, λ = 210 nm, t_r(major) = 2.972 min, t_r(minor) = 2.555 min).

$[\alpha]_D^{23}$ = +96.1 (c = 0.19, $CHCl_3$).

^{1}H NMR (400 MHz, $CDCl_3$) δ 8.73 (s, 1H), 7.98 (dd, *J* = 8.7, 2.0 Hz, 1H), 7.90 (d, *J* = 8.5 Hz, 2H), 7.80 – 7.63 (m, 3H), 7.55 – 7.47 (m, 2H), 7.47 – 7.38 (m, 3H), 7.30 – 7.19 (m, 2H), 7.16 – 7.06 (m, 2H), 7.04 – 6.90 (m, 6H), 6.88 (d, *J* = 8.7 Hz, 1H), 6.79 (dd, *J* = 6.7, 2.9 Hz, 2H), 6.74 (d, *J* = 8.5 Hz, 1H), 4.09 (d, *J* = 15.7 Hz, 1H), 3.78 (d, *J* = 15.7 Hz, 1H).

^{13}C NMR (101 MHz, $CDCl_3$) δ 152.1, 146.1, 140.5 (d, *J* = 8.1 Hz), 139.4, 134.7, 134.6 (d, *J* = 2.3 Hz), 133.8, 132.8, 132.4 (d, *J* = 11.4 Hz), 132.1 (d, *J* = 9.2 Hz), 132.0, 131.8 (d, *J* = 2.8 Hz), 131.1 (d, *J* = 2.9 Hz), 131.0 (d, *J* = 9.4 Hz), 130.3, 129.3, 128.9 (d, *J* = 12.3 Hz), 128.5 (d, *J* = 12.1 Hz), 128.4 (d, *J* = 12.1 Hz), 128.4, 128.2, 128.1 (d, *J* = 6.4 Hz), 127.7 (d, *J* = 12.1 Hz), 127.7, 127.2 (d, *J* = 17.9 Hz), 126.3 (d, *J* = 18.1 Hz), 126.0, 38.3.

^{31}P NMR (162 MHz, $CDCl_3$) δ 29.33.

HRMS: (ESI) calculated for $C_{38}H_{29}NOP$ $[M+H]^+$ m/z: 546.1981, found: 546.1989.

m.p. 244-245 °C

Int-b

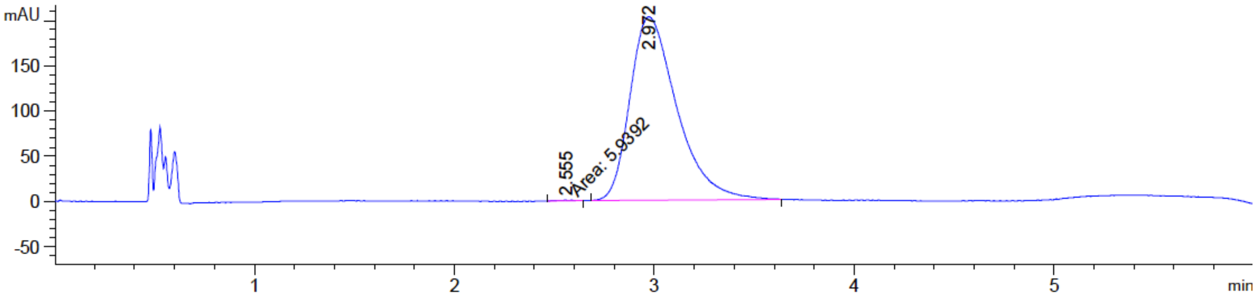

Peak #	RetTime [min]	Type	Width [min]	Area [mAU*s]	Height [mAU]	Area %
1	2.555	MM	0.1212	5.93920	8.16717e-1	0.1796
2	2.972	BV R	0.2373	3301.57007	202.42331	99.8204

Rac-Int-b

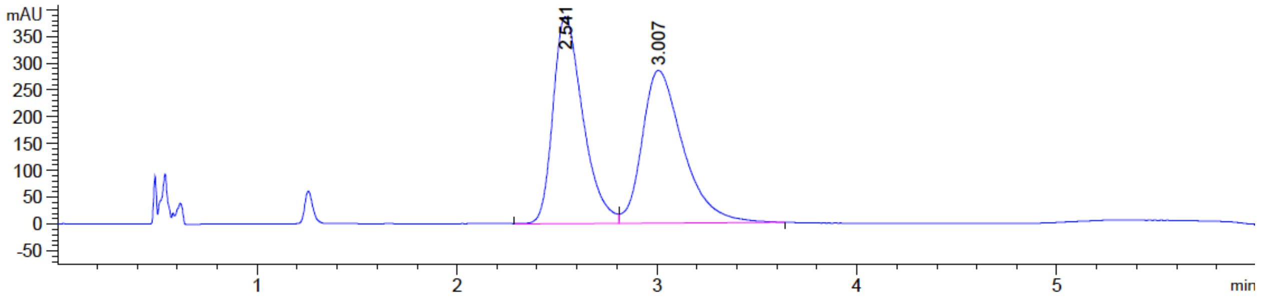

Peak #	RetTime [min]	Type	Width [min]	Area [mAU*s]	Height [mAU]	Area %
1	2.541	BV	0.1584	3997.54077	385.67932	49.6118
2	3.007	VV R	0.2104	4060.09399	284.71844	50.3882

3-Benzyl-4-(2-(diphenylphosphaneyl)naphthalen-1-yl)quinoline (10)

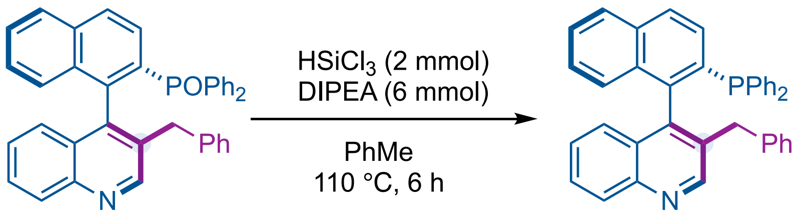

To a 10 mL oven-dried round-bottom flask flask equipped with a stirring bar was added **int-b** (109.1 mg, 0.2 mmol, 1 equiv.). The flask was sealed before being evacuated and backfilled with argon three times. Then, toluene (1.5 mL), DIPEA (517.0 mg, 4 mmol) and $HSiCl_3$ (270.9 mg, 2 mmol) were added and the reaction mixture was stirred at 120 °C for 6 hours under argon. After the reaction was completed, the solvent was removed under *vacuum* and the crude was purified by flash column chromatography on silica gel (Hexane/Ethyl acetate = 5:1) to afford the title compound as white solid (79.4 mg, 76% yield, 99% ee). Enantiomeric excess was determined by SFC analysis on a chiral stationary phase (CHIRALPAK IB-3, 1 mL/min, 20% methanol, λ = 210 nm, t_r(major) = 3.746 min).

$[\alpha]_D^{23}$ = -19.8 (c = 0.2, $CHCl_3$).

^{1}H NMR (400 MHz, $CDCl_3$) δ 8.82 (s, 1H), 8.14 (d, *J* = 8.5 Hz, 1H), 7.92 (dd, *J* = 17.5, 8.2 Hz, 2H), 7.59 – 7.44 (m, 3H), 7.36 – 7.30 (m, 3H), 7.28 – 7.19 (m, 4H), 7.18 – 7.12 (m, 2H), 7.09 – 6.97 (m, 7H), 6.87 – 6.76 (m, 3H), 3.63 (d, *J* = 2.9 Hz, 2H).

^{13}C NMR (101 MHz, $CDCl_3$) δ 152.2, 146.6, 144.4 (d, J = 8.3 Hz), 141.3 (d, J = 34.2 Hz), 139.0, 137.0 (d, J = 12.3 Hz), 136.6 (d, J = 12.4 Hz), 135.2 (d, J = 13.4 Hz), 133.9 (d, J = 20.6 Hz), 133.6 (d, J = 20.2 Hz), 133.6, 133.0 (d, J = 2.6 Hz), 132.2 (d, J = 7.4 Hz), 130.2 (d, J = 1.7 Hz), 129.3, 129.0 (d, J = 48.3 Hz), 128.7, 128.7, 128.4 (d, J = 7.1 Hz), 128.4, 128.2, 128.1, 127.2, 126.9, 126.5 (d, J = 3.0 Hz), 126.3 (d, J = 2.5 Hz), 126.2, 37.5 (d, J = 2.4 Hz).

^{31}P NMR (162 MHz, $CDCl_3$) δ -11.54.

m.p. 196-197 °C

HRMS: (ESI) calculated for $C_{38}H_{29}NP$ $[M+H]^+$ m/z: 530.2032, found: 530.2035.

4

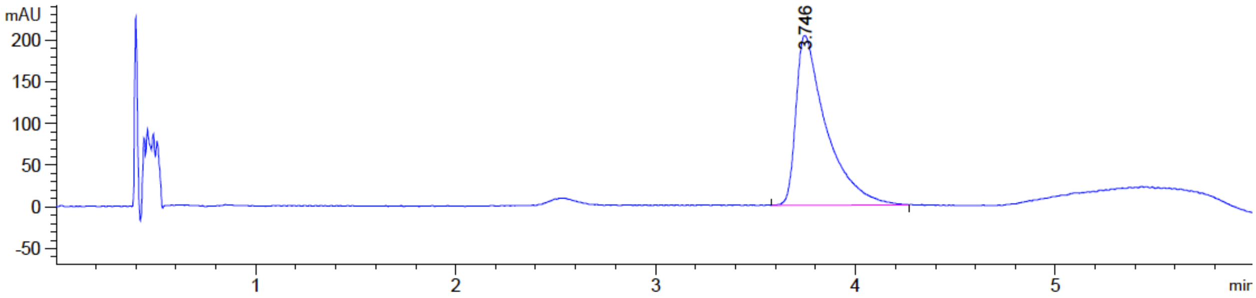

Peak #	RetTime [min]	Type	Width [min]	Area [mAU*s]	Height [mAU]	Area %
1	3.747	BV R	0.1466	2332.50415	221.06023	100.0000

***Rac*-4**

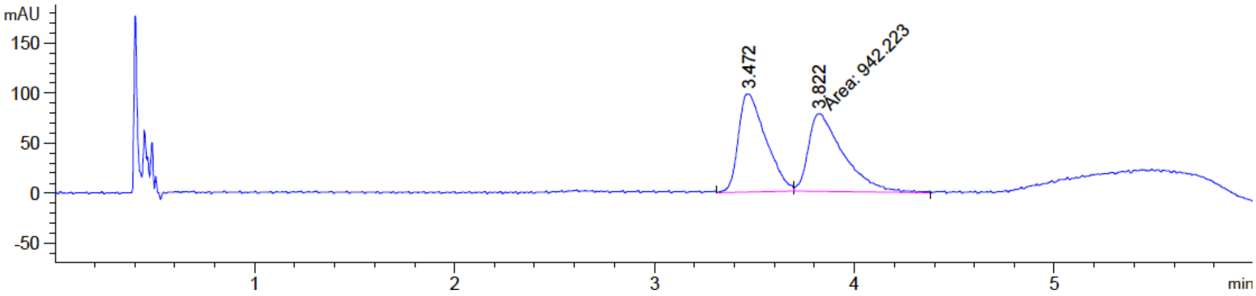

Peak #	RetTime [min]	Type	Width [min]	Area [mAU*s]	Height [mAU]	Area %
1	3.472	BV	0.1258	939.10638	97.95964	49.9172
2	3.822	MM	0.2022	942.22278	77.67889	50.0828

(R)-1-benzyl-3-hydroxy-3-phenylindolin-2-one (12)

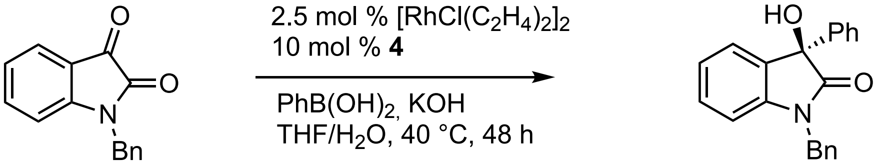

To a 10 mL oven-dried round-bottom flask flask equipped with a stirring bar was added $[RhCl(C_2H_4)_2]_2$ (1.9 mg, 0.005 mmol, 2.5 mol%) and **10** (10.6 mg, 0.02 mmol, 10 mol%). The flask was sealed before being evacuated and backfilled with argon three times. Tetrahydrofuran (1 mL) was added followed by addition of KOH (0.1 mL, 0.03 mmol; 0.3 M aqueous), isatin **11** (47.4 mg, 0.2 mmol), and $PhB(OH)_2$ (48.8 mg, 0.4 mmol). The resulting mixture was stirred for 48 h at 40 °C. The reaction was quenched with saturated aqueous NH_4Cl (5 mL) and extracted with ethyl acetate (3 x 3 mL). The organic phase was washed with saturated aqueous $NaHCO_3$ (10 mL), brine (10 mL) and dried over anhydrous Na_2SO_4. The solvent was removed under *vacuum* and the crude was purified by flash column chromatography on silica gel (hexane/ethyl acetate = 5:1) to afford compound **6** as a white solid (47.9 mg, 76% yield, 15% ee). Enantiomeric excess was determined by SFC analysis on a chiral stationary phase (CHIRALPAK IA-3, 1 mL/min, 20% methanol, λ = 210 nm, t_r(major) = 3.313 min, t_r(minor) = 4.017).

$[\boldsymbol{\alpha}]_D^{23}$ = +8.8 (c = 0.32, $CHCl_3$).

^{1}H NMR (400 MHz, $CDCl_3$) δ 7.46 – 7.39 (m, 2H), 7.39 – 7.26 (m, 9H), 7.23 (td, *J* = 7.8, 1.3 Hz, 1H), 7.04 (td, *J* = 7.6, 1.0 Hz, 1H), 6.79 (d, *J* = 7.9 Hz, 1H), 5.04 (d, *J* = 15.7 Hz, 1H), 4.82 (d, *J* = 15.7 Hz, 1H), 3.68 (s, 1H).

^{13}C NMR (101 MHz, $CDCl_3$) δ 177.8, 142.8, 140.3, 135.6, 131.8, 129.9, 129.0, 128.8, 128.4, 127.9, 127.4, 125.5, 125.1, 123.7, 109.9, 44.2.

These data are in accordance with the literature.[13]

12

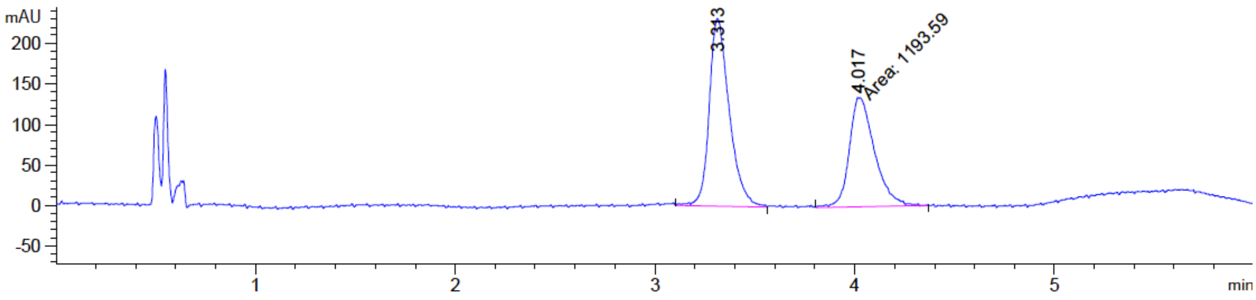

Peak #	RetTime [min]	Type	Width [min]	Area [mAU*s]	Height [mAU]	Area %
1	3.313	VV R	0.0979	1615.97461	231.50938	57.5170
2	4.017	MM	0.1474	1193.58618	134.93788	42.4830

Rac-12

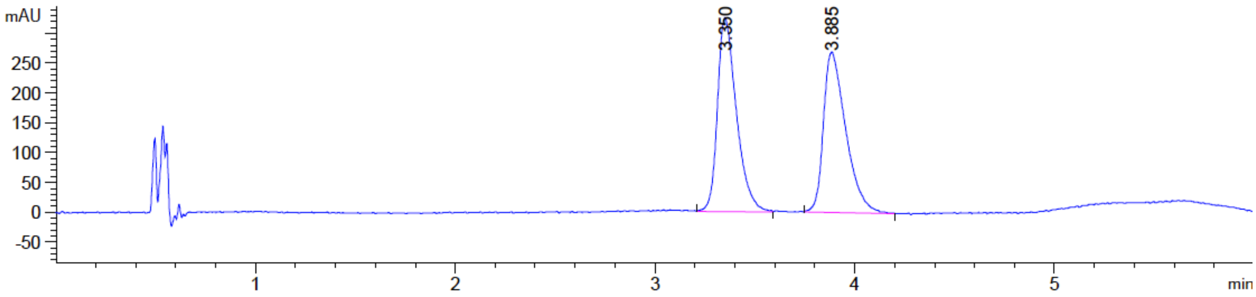

Peak #	RetTime [min]	Type	Width [min]	Area [mAU*s]	Height [mAU]	Area %
1	3.350	BV R	0.0995	2147.66211	325.53159	50.5592
2	3.885	BV R	0.1065	2100.15234	269.23892	49.4408

Diethyl (*R*,*E*)-2-(1,3-diphenylallyl)malonate (14)

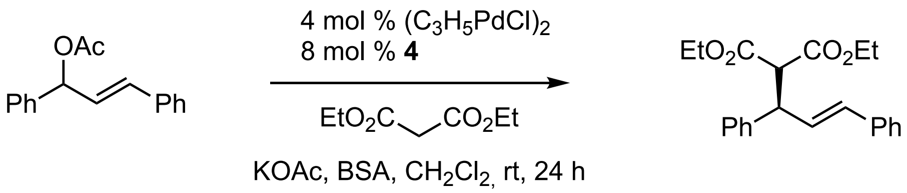

To a 10 mL oven-dried round-bottom flask flask equipped with a stirring bar was added $Pd(C_3H_5)Cl]_2$ (2.9 mg, 0.008 mmol, 4 mol%) and **10** (8.5 mg, 0.016 mmol, 8 mol%). The flask was sealed before being evacuated and backfilled with argon three times. Dichloromethane (1 mL) was added and the reaction was stirred for 30 min. Then, 1,3-diphenylprop-2-en-1-yl acetate (50.4 mg, 0.2 mmol), dimethyl malonate (96.1 mg, 0.6 mmol), KOAc (1.6 mg, 0.016 mmol) and *N,O*-

bis(trimethylsilyl)acetamide (BSA, 122.1 mg, 0.6 mmol) were added. The reaction mixture was stirred at room temperature for 24 h.Then, quenched with saturated aqueous NH_4Cl (5 mL) and extracted with dichloromethane (3 x 3 mL). The organic phase was washed with saturated aqueous $NaHCO_3$ (10 mL), brine (10 mL) and dried over anhydrous Na_2SO_4. The solvent was removed under *vacuum* and the crude was purified by flash column chromatography on silica gel (hexane/ethyl acetate = 25:1) to afford compound **14** as a colorless oil (35.2 mg, 50% yield, 11% ee). Enantiomeric excess was determined by SFC analysis on a chiral stationary phase (CHIRALPAK IG-3, 1 mL/min, 20% methanol, λ = 210 nm, t_r(major) = 3.344 min, t_r(minor) = 1.615).

$[\alpha]_D^{23}$ = +2.7 (c = 0.12, $CHCl_3$).

^{1}H NMR (400 MHz, $CDCl_3$) δ 7.39 – 7.12 (m, 10H), 6.48 (d, *J* = 15.8 Hz, 1H), 6.34 (dd, *J* = 15.8, 8.5 Hz, 1H), 4.27 (dd, *J* = 11.0, 8.5 Hz, 1H), 4.18 (q, *J* = 7.1 Hz, 2H), 4.04 – 3.87 (m, 3H), 1.21 (t, *J* = 7.1 Hz, 3H), 1.01 (t, *J* = 7.1 Hz, 3H).

^{13}C NMR (101 MHz, $CDCl_3$) δ 168.0, 167.6, 140.5, 137.0, 131.8, 129.5, 128.8, 128.6, 128.1, 127.6, 127.2, 126.5, 61.7, 61.5, 57.9, 49.3, 14.3, 13.9.

These data are in accordance with the literature.[14]

14

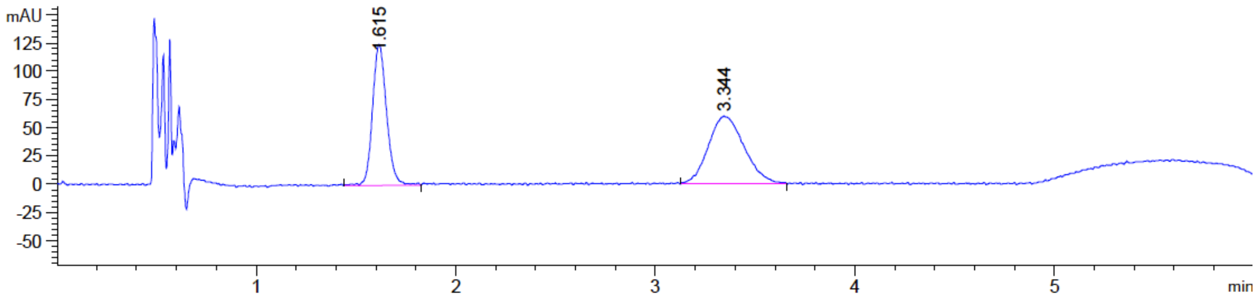

Peak #	RetTime [min]	Type	Width [min]	Area [mAU*s]	Height [mAU]	Area %
1	1.615	VV R	0.0740	609.60577	124.76763	44.6602
2	3.344	BV R	0.1489	755.38208	60.20923	55.3398

Rac-14

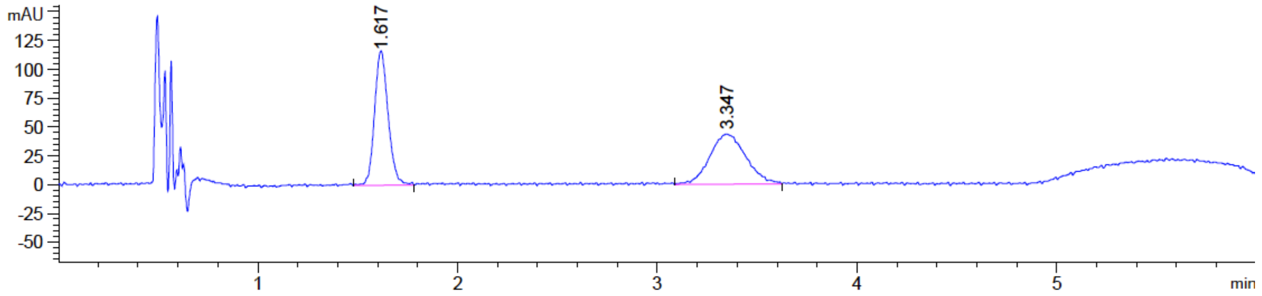

Peak #	RetTime [min]	Type	Width [min]	Area [mAU*s]	Height [mAU]	Area %
1	1.617	VV R	0.0721	557.24261	116.93989	50.4001
2	3.347	VV R	0.1513	548.39624	43.61324	49.5999

6. X-Ray crystal data of 3al.

Single-crystal X-Ray difraction analysis of 3al: The crystal of **3al** used for the single-crystal X-ray diffraction experiment was grown by slow evaporation of a solution of **3al** in dichloromethane and hexane at room temperature. An ORTEP diagram of the crystal structure of **3al** is shown below (CCDC No: 2437760):

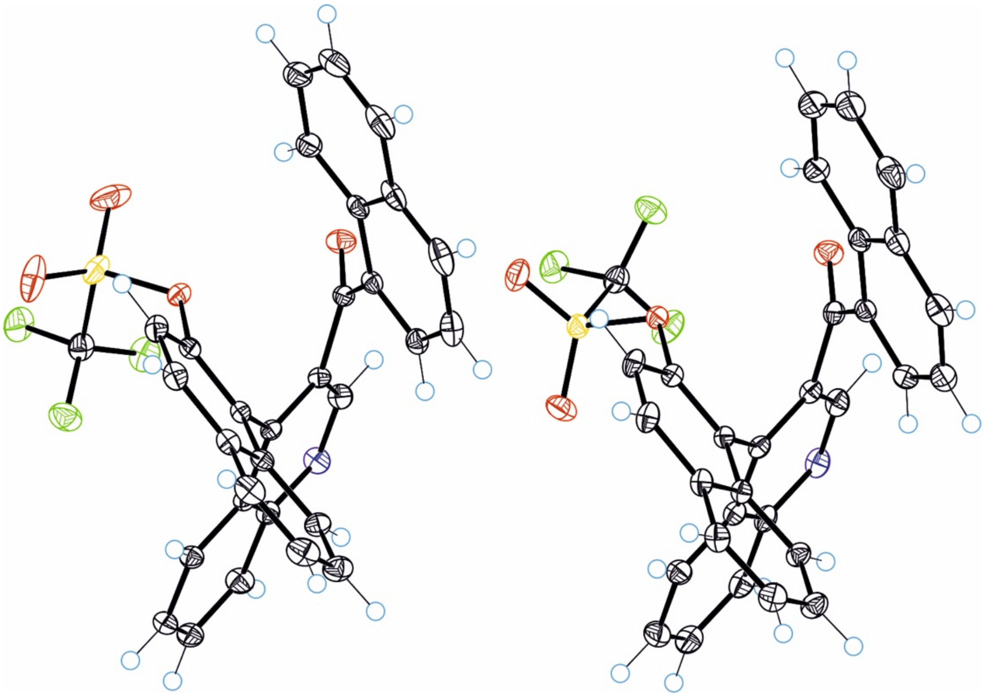

Table S2. Crystal data and structure refinement for mo_VGAII45b_0m_a.

Identification code	mo_VGAII45b_0m_a
Empirical formula	$C_{31}H_{18}F_3NO_4S$
Formula weight	557.52
Temperature/K	99.72
Crystal system	monoclinic
Space group	$P2_1$
a/Å	11.0608(4)
b/Å	16.2688(6)
c/Å	14.1181(5)
α/°	90
β/°	98.0560(10)

γ/°	90
Volume/Å^3	2515.42(16)
Z	4
ρ_{calc}g/cm^3	1.472
μ/mm^{-1}	0.191
F(000)	1144.0
Crystal size/mm^3	0.1 × 0.1 × 0.05
Radiation	MoKα (λ = 0.71073)
2Θ range for data collection/°	3.718 to 63.282
Index ranges	-16 ≤ h ≤ 16, -21 ≤ k ≤ 23, -14 ≤ l ≤ 20
Reflections collected	32382
Independent reflections	15140 [R_{int} = 0.0215, R_{sigma} = 0.0306]
Data/restraints/parameters	15140/1/721
Goodness-of-fit on F^2	1.028
Final R indexes [I>=2σ (I)]	R_1 = 0.0351, wR_2 = 0.0892
Final R indexes [all data]	R_1 = 0.0391, wR_2 = 0.0919
Largest diff. peak/hole / e Å^{-3}	0.54/-0.23

7. Computational studies.

Density Functional Theory (DFT) calculations were carried out at the B3LYP-D3BJ level of theory[15–19] using the Gaussian 09 (rev. A.02) package.[20] Rhodium was described by the LANL2DZ

basis set and associated pseudopotentials,[21] and supplemented with a shell of f-type polarization functions, using the exponents provided by Frenking and coworkers.[22] The remaining atoms were described by the Dunning's cc-pVDZ basis set.[23,24] Solvent effects of chlorobenzene were included in both geometry optimizations and energy calculations by means of the IEF-PCM solvent model[25] as implemented in Gaussian 09. A conformational analysis of the possible conformers involved in the enantio-determining step was conducted using CREST.[26] A dataset collection of the optimized structures is available in the ioChem-BD repository.[27]

a) Characterization of the reaction mechanism using a model system.

First, we characterized the whole reaction mechanism to further support the reactivity of the Rh-carbene toward indole substrates and to identify the selectivity-determining step, aiming to rationalize the experimentally observed enantioselectivity. Owing to the large size of the catalyst, this was done using simplified model systems of both the Rh catalyst and the substrate to reduce the computational cost of the mechanistic study. Specifically, the Rh catalyst was modeled with four acetate chelating ligands ($Rh_2(OAc)_4$) rather than with the sophisticated ligands used in the real system, while the triflate group bound to the aryl moiety of the substrate was replaced by a smaller methyl group.

Figure S2 shows the calculated free-energy profile for the cyclopropanation of the indole promoted by the Rh-carbene (species **A**) followed by electrocyclic-ring opening of the cyclopropane to give the product. Such a class of dimeric Rh compounds are well-known to spontaneously yield Rh-carbenes upon reacting with diazo compounds, releasing N_2.[28] Thus, we did not study this process in detail but focused on the reaction between the Rh-carbene and the indole substrates. As shown in Figure S2, the reaction begins with a stepwise cyclopropanation of the indole, which initiates via a nucleophilic attack of the double bond of the indole to the carbene, overcoming a low free-energy barrier of only 0.8 kcal mol^{-1}. This takes place through **TS1**, which involves a C-C bond formation between the carbon atom of the carbene and the C2 (adjacent to the N atom) of the indole, most likely due to steric hinderance induced by the arene group, which might hamper the attack from the more nucleophilic C3.[29–31] This yields the zwitterionic intermediate **B**, which lies 16 kcal mol^{-1} below the reactants in terms of Gibbs free energy.

Intermediate **B** can then evolve towards the slightly less stable intermediate **C** through **TS2**, overcoming a moderate free-energy barrier of 14.2 kcal mol^{-1} (Figure S2). In this process, a second C-C bond is formed between the carbon atom of the carbene and C3 of the indole, leading to the cyclopropane intermediate. Also, in the way from **TS2** to intermediate **C**, the cyclopropane intermediate suffers a rearrangement to coordinate to the Rh center of the catalyst through the oxygen atom of the ketone group. At this stage, the decoordination of the cyclopropane intermediate from the Rh catalyst happens with a free-energy release of 12.5 kcal mol^{-1} (Figure S3). Hence, the final electrocyclic-ring opening from **C'** to deliver **D** can take place via **TS3** with an also smooth barrier of 12.0 kcal mol^{-1}, releasing the bromide anion, which forms an ion pair with the positively-charged protonated quinoline ring. This step was predicted to be strongly exergonic, providing the thermodynamic driving force for the reaction and making it irreversible, with a reverse barrier of more than 70 kcal mol^{-1}. The release of product **E** is finally achieved by deprotonation by DIPEA, that is also coordinating the bromide anion.

We introduced a final refinement in the model by introducing an additional AcOEt group coordinating to the second rhodium center, that without the carbene. The resulting free energy profile is reported in Figure S4. As expected, the energies are similar to those in Figure S2 in the absence of the AcOEt group. Intermediate B″ converts to C″ via transition state TS2" with a barrier of 9.1 kcal·mol^{-1}, marginally lower than in the absence of AcOEt. Finally, cyclopropane C' is released by decoordination of the rhodium catalyst with a free-energy release of 11.9 kcal mol^{-1}. An unpleasant feature of Figure S4 is that TS1" has the same energy as the preceding intermediate A". This is a numerical error associated to the fact that this is a bimolecular step, and that the optimizations are carried out in potential energy rather than free energy. It is not unprecedented, and it has little chemical meaning, as the barrier was also very small (0.8 kcal mol^{-1}) in the absence of the extra AcOEt group.

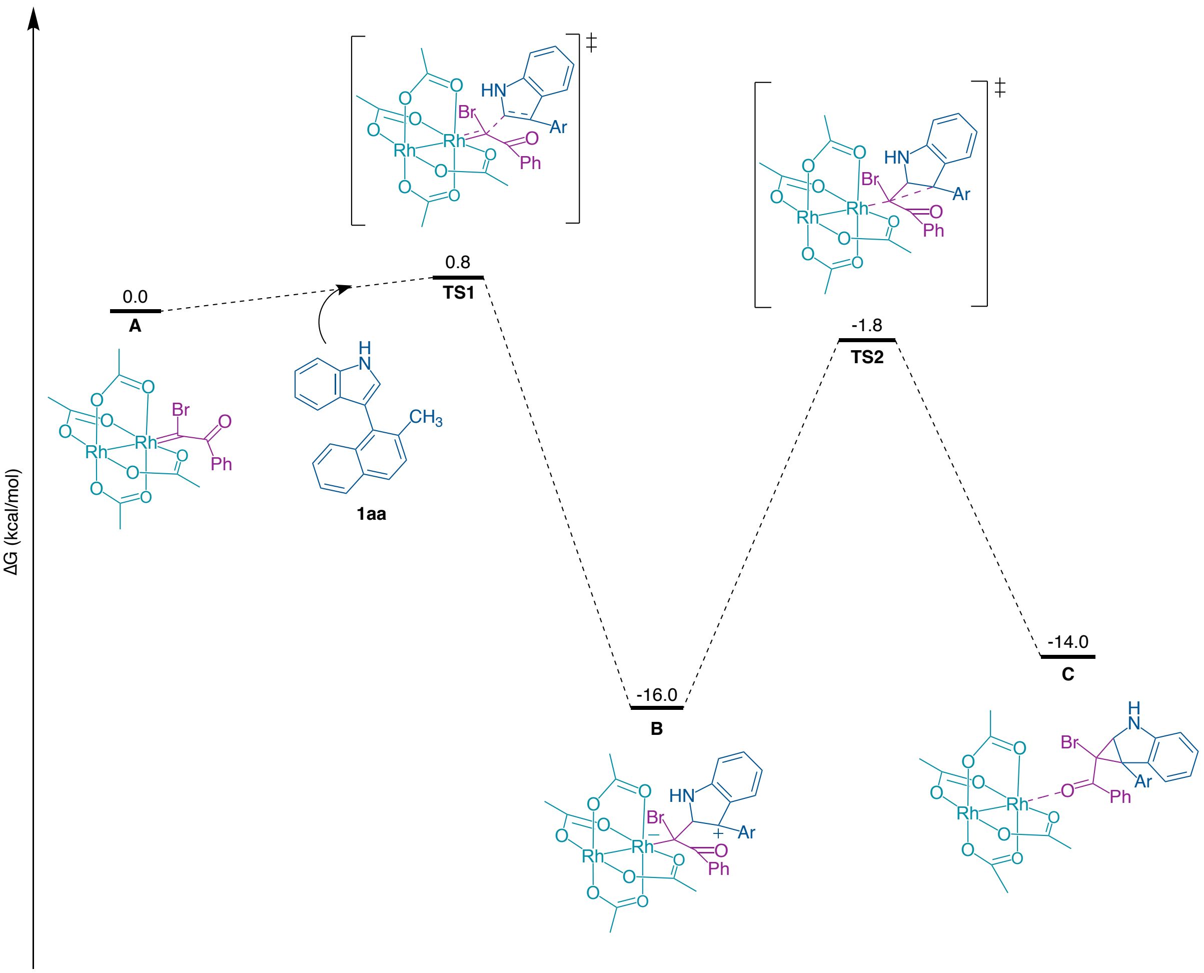

Figure S2. Calculated free-energy profile (kcal mol^{-1}) for the stepwise cyclopropanation.

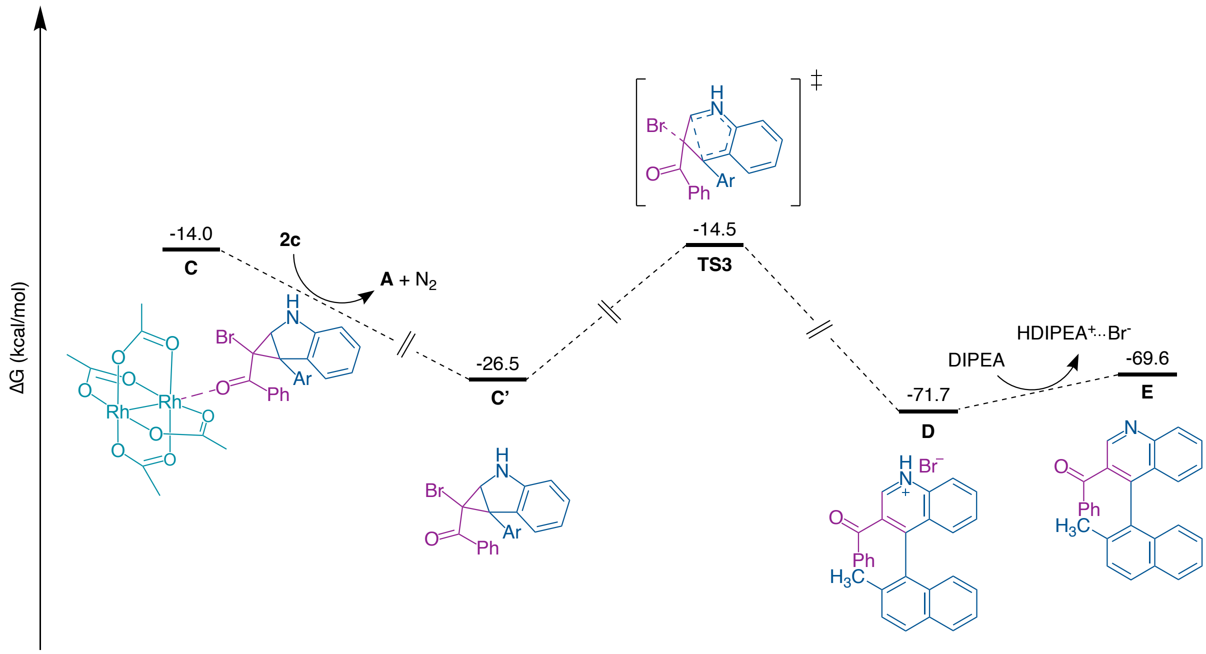

Figure S3. Calculated free-energy profile (kcal mol^{-1}) for Rh decoordination and electrocyclic ring opening.

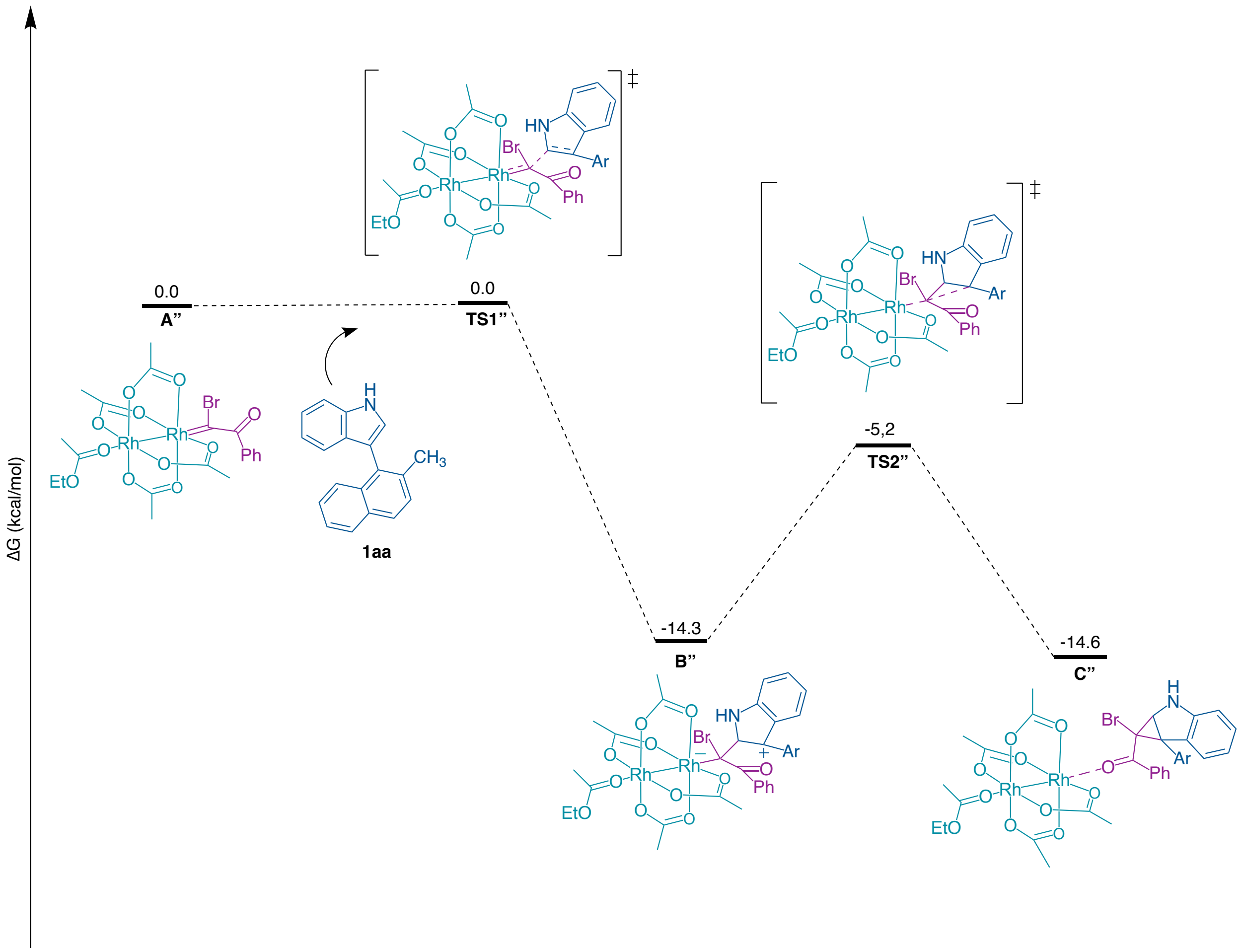

Figure S4. Calculated free-energy profile (kcal mol^{-1}) for the stepwise cyclopropanation, including an extra AcOEt group in the system.

b) Rotational barriers of B, C and 3c

To support that the process from **A** to **TS1** corresponds to the selectivity-determining step, we explore possible interconversion between pro-*R* and pro-*S* configurations on intermediates **B** and **C**. To do so, we performed a scan of the dihedral angle around the chiral axis of the intermediate **B** (Figure S5) and found that the rotation is blocked at room temperature ($\Delta G^{\ddagger}$ >30 kcal mol^{-1}). Similarly, the scan of the dihedral angle around the chiral axis of the intermediate **C** proved that the rotation is blocked (Figure S6, $\Delta G^{\ddagger}$ >24 kcal mol^{-}1). Accordingly, the enantio-determining step is the first one involving **TS1**. We also performed a scan of the dihedral angle around the chiral

axis in **3c** (Figure S7) to prove that the interchange among the two atropoisomeric products is not feasible at room temperature.

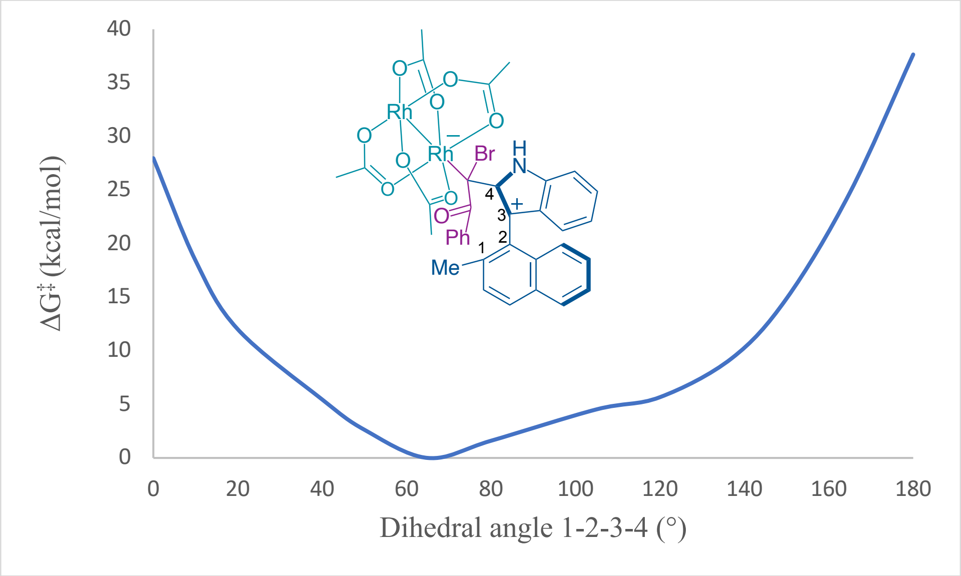

Figure S5. Scan of the dihedral angle in intermediate **B**.

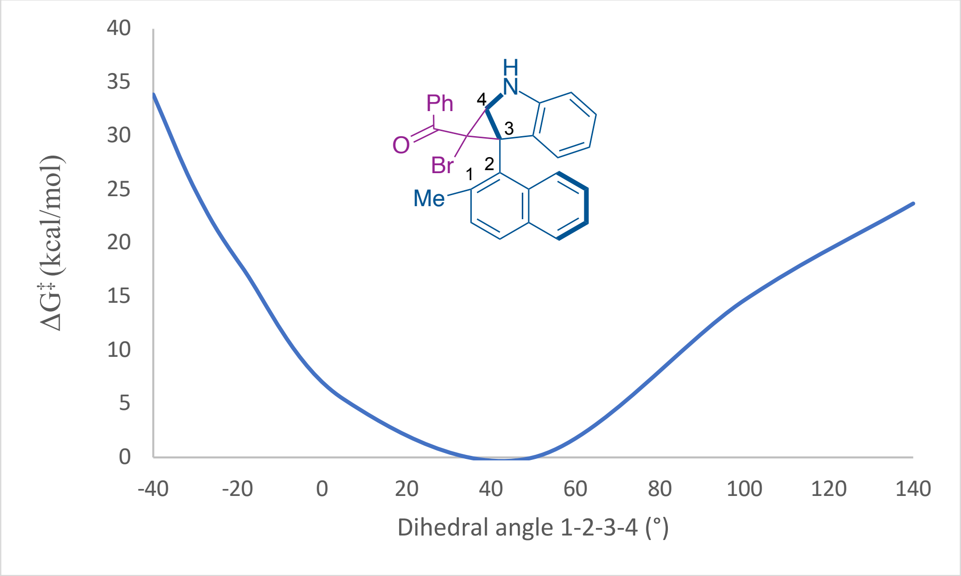

Figure S6. Scan of the dihedral angle in intermediate **C**.

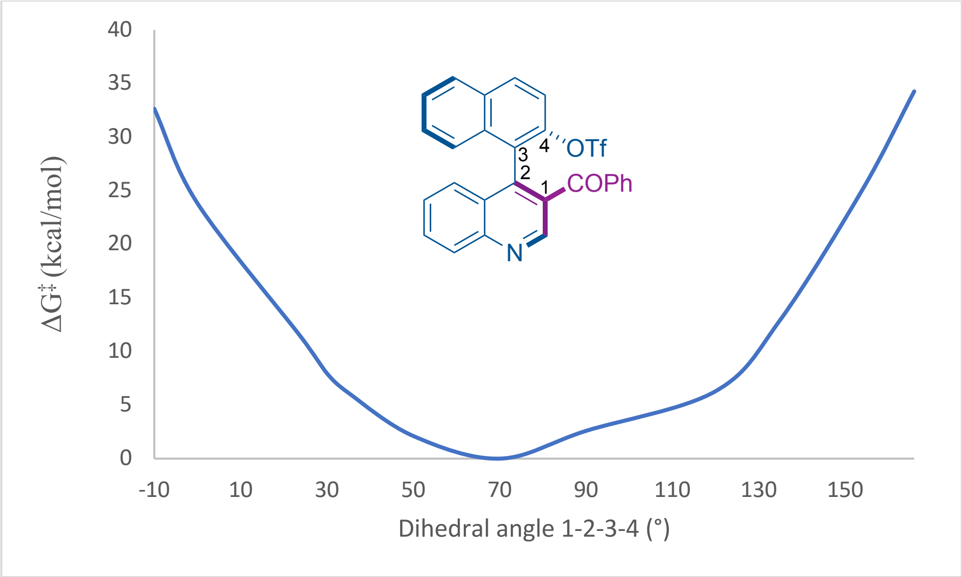

Figure S7. Scan of the dihedral angle in species **3c.**

c) Conformational analysis of TS1 in the real system using CREST

Since the enantio-determining step is the first step, a conformational analysis was conducted on the possible conformers involved in the cyclopropanation step in the real system using CREST. We individuated a total number of 16 possible conformers for **TS1**, but only the 8 involving a *syn* attack were considered (Figure S8).

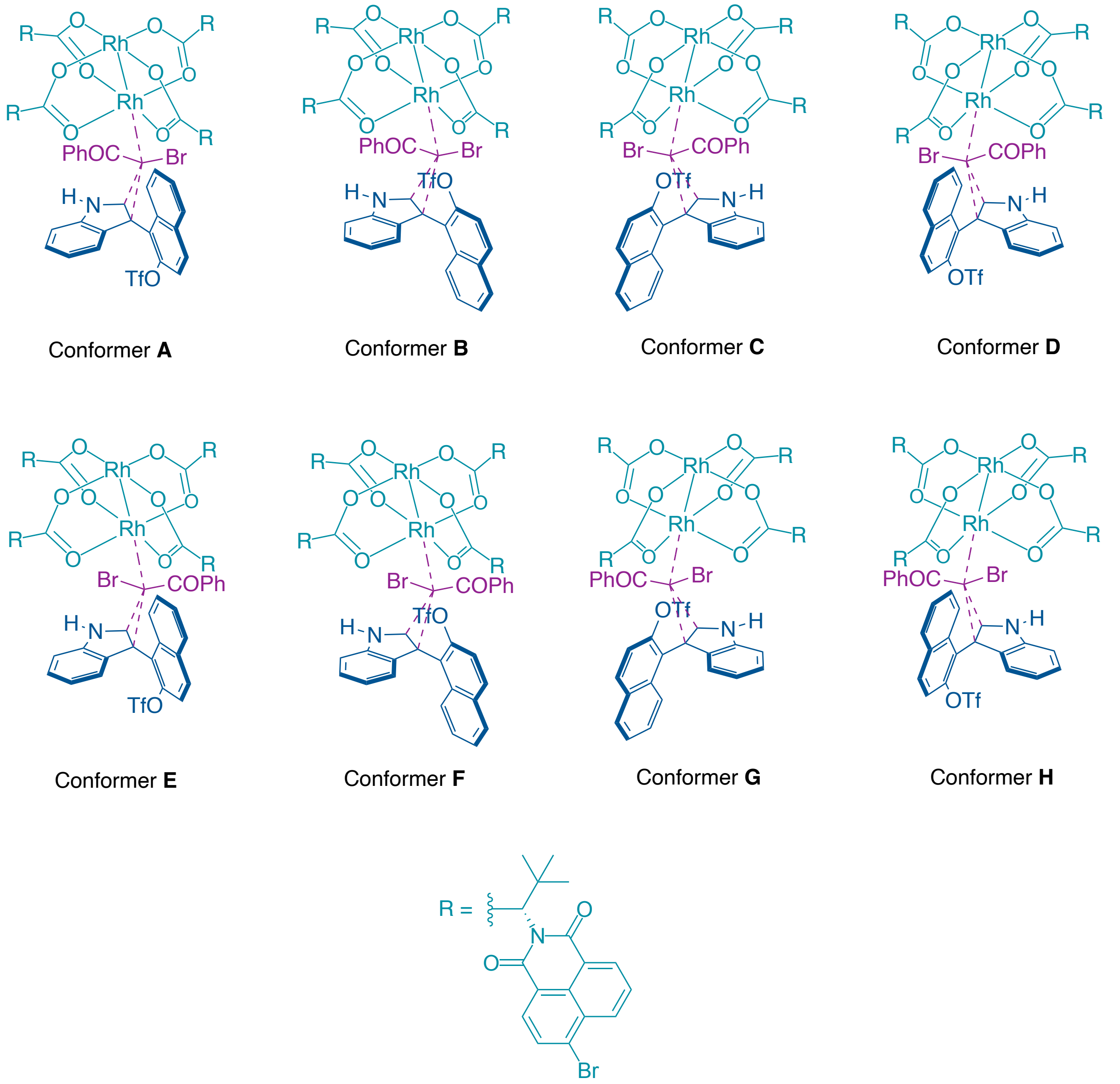

Figure S8. Possible conformers of **TS1** considered in the conformational analysis.

The results for the conformational analysis are summarized in Table S3.

Table S3. Selectivity and relative energies of conformers of **TS1**

Conformer label	Diastereoselectivity	Atroposelectivity	Relative energy (kcal mol^{-1})
**A**	*RRR*	*R*	0
**B**	*RRR*	*S*	+2,7
**C**	*SSS*	*R*	+4,2
**D**	*SSS*	*S*	-3,7
**E**	*SRR*	*R*	-3,9
**F**	*SRR*	*S*	+0,7
**G**	*RSS*	*R*	-1,4
**H**	*RSS*	*S*	-8,6

As inferred from the relative energies of the different conformers, the approach of the indole to the Rh-carbene leading to the cyclopropane *RSS* in a pro-*S* fashion is the most favourable (Table S3, conformer H). This qualitatively aligns with the experimental selectivity, although to understand its origin, conformer H should be compared with the most likely conformer of TS1 leading to the R enantiomer. According to CREST energies, the latter corresponds to conformer E (see Table S3). Still, revisiting the structures predicted by CREST as the most stables by means of DFT (B3LYP-D3BJ) calculations revealed that conformer G is the most stable leading to the *R* enantiomer. As such, we surmised that the origin of the atroposelectivity of the process could be figured out by looking with DFT calculations to the structures of **TS1** of conformer G and H. Indeed, **TS1*S***, arising from conformer H resulted 2.9 kcal mol^{-1} lower than **TS1*R*** (conformer G), in qualitative, if not quantitative agreement with the experimental enantiomeric excess of 99.

As discussed in the main text, we postulated that the preference for **TS1*S*** is due to the presence of steric clash between the triflate of the substrate and the phenyl ring of the carbene in **TS1*R***, absent in **TS1*S***. To evaluate this hypothesis, we performed additional calculations replacing the triflate moiety by a hydrogen atom in the TS structure. This replacement makes **TS1*R*-H** (conformer G) more stable than **TS1*S*-H** (conformer H) by 0.6 kcal mol^{-1}, supporting that the triflate is key to the atroposelectivity of the reaction.

It is noteworthy that a hydrogen bond is observed in both TSs, leading to a stabilization effect (Figure S9).

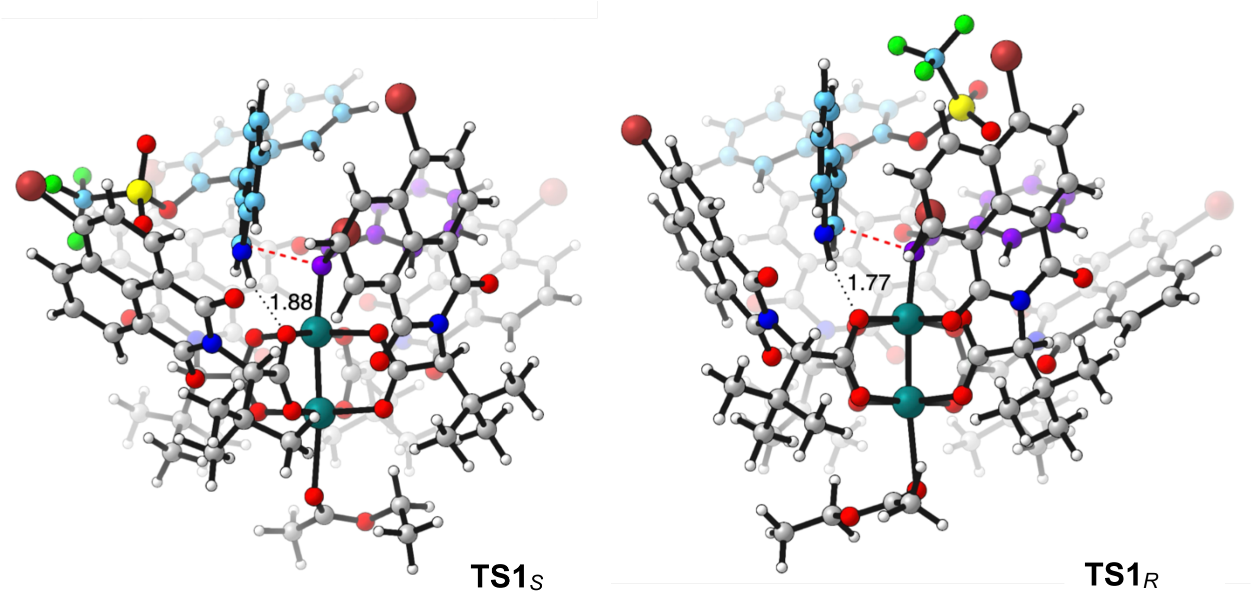

Figure S9. Hydrogen bonding in **TS1*S*** and **TS1*R***.

d) **Cartesian coordinates (Å) for the most representative species optimized at the B3LYP-D3BJ level.**

A

C	5.05658600	6.67558200	-0.06931900
Rh	3.70466600	5.28735600	0.14116200

Rh	2.14268000	3.41737200	0.30278900
O	5.20702300	3.87375100	0.38107800
O	3.75663900	2.14020900	0.51295000
O	3.70599000	4.98921300	-1.90151400
O	2.29444900	3.22170900	-1.75800400
O	2.06192800	6.51434900	-0.06377600
O	0.61100200	4.78000700	0.07664100
O	3.57547300	5.48074600	2.18901900
O	2.12490700	3.74325700	2.34695900
C	2.83256500	4.66934200	2.84436300
C	2.83363500	4.83275800	4.34253700
H	3.74784900	4.36572700	4.74336800
H	2.85549400	5.89824500	4.60762800
H	1.95883200	4.33868000	4.78217600
C	0.88537800	6.01010500	-0.04821000
C	3.01853400	4.03387800	-2.40534600
C	4.92216900	2.62878600	0.49341800
C	6.09697700	1.69100900	0.58970500
H	6.58528300	1.63729900	-0.39648100
H	6.83228600	2.08379900	1.30557300
H	5.76482800	0.68902000	0.88573700
C	3.09942500	3.89428800	-3.90333900
H	2.54424600	3.01164700	-4.24122700

H	2.68301700	4.80034400	-4.36963700
H	4.15501900	3.82449500	-4.20479300
C	-0.25348300	6.98880500	-0.16783700
H	-0.36104800	7.52080100	0.79097800
H	-0.02676900	7.73539300	-0.94115500
H	-1.18808900	6.46286400	-0.39615400
Br	6.18158400	7.24636700	1.30840600
C	5.36342000	7.33748400	-1.38236800
O	4.78097300	8.40044500	-1.56296600
C	6.26774800	6.68051800	-2.34208300
C	6.58327000	7.34549100	-3.53829600
C	6.79860500	5.40717400	-2.07668700
C	7.42939900	6.74042000	-4.46423100
H	6.15526600	8.33183300	-3.72188300
C	7.64353400	4.80605700	-3.00908100
H	6.53163300	4.87644200	-1.16118900
C	7.95906600	5.47040400	-4.19991400
H	7.67833900	7.25378900	-5.39464600
H	8.05390100	3.81443100	-2.81038100
H	8.62063600	4.99599300	-4.92768900

1aa

C	1.29081700	-3.49956400	-1.79661300

C	2.20896200	-4.37922800	-1.15124700
C	1.57304500	-4.84663900	0.06193700
C	0.32971900	-4.24849700	0.09888000
N	0.15947700	-3.44294900	-1.00940100
H	-0.66288600	-2.88784400	-1.20290800
C	2.14136700	-5.79276000	1.04917800
C	3.30430000	-5.41070400	1.80351900
C	1.57643500	-7.05444000	1.23432700
C	3.88348000	-6.33614200	2.73359200
C	2.16071800	-7.94974100	2.17305700
C	3.27943200	-7.61069200	2.89766800
H	1.70875100	-8.93600900	2.30376000
H	3.71863700	-8.31824100	3.60503600
C	0.36473800	-7.51024900	0.45748000
H	-0.56931800	-7.13525500	0.90933200
H	0.38722300	-7.14158900	-0.57794700
H	0.30257200	-8.60843600	0.44157700
H	-0.45444300	-4.33307900	0.84610300
C	1.59328000	-2.86503100	-3.00923600
C	3.45799400	-4.62526900	-1.75154000
H	4.17407700	-5.29529000	-1.27195600
H	0.87919200	-2.19297700	-3.48968100
C	3.76110100	-3.99683700	-2.95541400

C	2.83833700	-3.12479200	-3.57717100
H	3.10629500	-2.64563500	-4.52110600
H	4.72741900	-4.17719700	-3.43112300
C	3.90475800	-4.12491600	1.67567700
C	5.03897000	-5.95600700	3.47133700
H	5.47035500	-6.67533500	4.17188200
H	3.46375600	-3.40563000	0.98654600
C	5.60254200	-4.70832300	3.31224100
C	5.02323300	-3.78354400	2.40751900
H	6.48933700	-4.42724800	3.88412300
H	5.46500900	-2.79148000	2.29178200

TS1

C	-0.84356300	-4.28659300	-2.59110300
C	-0.00671400	-5.28737000	-2.01134900
C	0.33535900	-4.83443200	-0.68803700
C	-0.30817400	-3.59963500	-0.52238800
N	-1.04212800	-3.30538000	-1.65022900
H	-1.31676000	-2.34092300	-1.83998800
C	1.68722000	-1.88454900	-0.49679000
Rh	0.63335800	-0.20124100	-0.22255700
C	2.35789000	-2.15455500	-1.82394300
Br	2.56353800	-2.65040800	0.98538200

Rh	-0.42062100	1.97365600	0.20040200
C	3.76403300	-1.69382900	-2.01453500
O	1.72217900	-2.68266700	-2.72510700
C	4.36538600	-0.72698900	-1.19160100
C	4.47863500	-2.23179300	-3.09754800
C	5.67095300	-0.31014800	-1.45440700
H	3.81136800	-0.28276800	-0.36545100
C	5.78873800	-1.82558700	-3.34360800
H	3.98974600	-2.97157600	-3.73206300
C	6.38627500	-0.86182700	-2.52264400
H	6.13319100	0.44953400	-0.82112200
H	6.34601100	-2.25738500	-4.17713800
H	7.41120000	-0.53913300	-2.71739300
O	-1.91377700	1.45208700	-1.12700200
C	-1.88275100	0.32051400	-1.68317400
O	-0.97527500	-0.57091800	-1.50123600
C	-2.99020700	-0.04844700	-2.63579700
H	-2.55751500	-0.37350800	-3.59344100
H	-3.55621400	-0.89663300	-2.21931600
H	-3.66420400	0.80161300	-2.79150000
O	1.12273400	2.41102200	1.49732100
C	2.05608100	1.56907100	1.63863000
O	2.11717700	0.42161400	1.07367700

C	3.22038800	1.93028600	2.52348500
H	3.55197600	1.04975000	3.08971000
H	4.05649800	2.25559500	1.88323000
H	2.94904500	2.75075700	3.19863800
O	1.62705200	0.70219300	-1.79128700
C	1.46240600	1.95044400	-2.01796700
O	0.70054000	2.72941200	-1.37063700
C	2.25364700	2.51460000	-3.16993200
H	2.16062700	3.60628600	-3.20598000
H	3.30781700	2.21834500	-3.06940600
H	1.87646300	2.07913500	-4.10833800
O	-0.45607600	-0.95680800	1.35669200
C	-1.25749800	-0.18366200	1.99266000
O	-1.45304100	1.04227300	1.74533800
C	-2.00713800	-0.81462400	3.13749100
H	-1.28963900	-1.07108300	3.93255700
H	-2.76320700	-0.12563800	3.53154000
H	-2.47675900	-1.75033400	2.80118400
C	1.18768100	-5.51622900	0.29741300
C	2.53318400	-5.88574300	-0.07130400
C	0.72637900	-5.77372700	1.59207200
C	3.36867200	-6.55455100	0.88135800
C	1.57670800	-6.44099300	2.51403700

C	2.85142300	-6.83046000	2.17429900
H	1.19446400	-6.65209900	3.51546700
H	3.48553800	-7.34594200	2.89939200
C	-0.65283600	-5.37346800	2.05691400
H	-0.66303800	-4.32897300	2.41318500
H	-1.39607000	-5.44774500	1.25139600
H	-0.97746900	-6.00649600	2.89574200
H	-0.41878900	-2.98786400	0.36558300
C	-1.33733900	-4.39693900	-3.89764900
C	0.29917000	-6.44448700	-2.75765700
C	3.10480200	-5.53476500	-1.32747700
C	4.70559700	-6.89127100	0.53030800
H	-1.96340700	-3.61512600	-4.33131500
H	0.92323500	-7.22830200	-2.32602700
H	2.50487600	-4.98488600	-2.04799600
H	5.32274600	-7.41025500	1.26777900
C	-1.00265000	-5.54062100	-4.61575500
C	-0.19920500	-6.55992300	-4.04840400
C	4.41146000	-5.85558800	-1.63254300
C	5.22090900	-6.55202300	-0.70192000
H	0.03059400	-7.44796400	-4.64049400
H	-1.37167400	-5.65898900	-5.63667600
H	6.25197700	-6.80464100	-0.95740800

H	4.82717500	-5.55854100	-2.59739600

B

C	-0.49512800	-4.18480300	-2.28582400
C	0.34855300	-5.23513300	-1.74862100
C	1.04174300	-4.71506000	-0.67451400
C	0.62120500	-3.27337300	-0.49773500
N	-0.34844100	-3.08619400	-1.56819600
H	-0.70124300	-2.14402400	-1.80920400
C	1.71971400	-2.16485700	-0.46524500
Rh	0.73190800	-0.16409100	-0.33838000
C	2.59417500	-2.18962500	-1.67017800
Br	2.72645400	-2.49422100	1.22992000
Rh	-0.40386400	1.99561700	-0.01501800
C	3.95568800	-1.56368800	-1.69968700
O	2.16138000	-2.72123300	-2.70116700
C	4.38143900	-0.54473700	-0.83173300
C	4.83308300	-2.01920800	-2.69793400
C	5.65889100	0.00054700	-0.96719800
H	3.71342600	-0.16031500	-0.06600300
C	6.11589600	-1.48519500	-2.82009300
H	4.48911200	-2.79979700	-3.37614000
C	6.53241500	-0.46917100	-1.95414500

H	5.97466800	0.80083000	-0.29411600
H	6.79045300	-1.85946900	-3.59311500
H	7.53412300	-0.04371300	-2.04833500
O	-1.73889000	1.47092900	-1.51600400
C	-1.62299500	0.35302800	-2.08366700
O	-0.70513600	-0.51024600	-1.82894900
C	-2.61824100	-0.03382600	-3.14712700
H	-2.08159300	-0.29743100	-4.07064300
H	-3.17053500	-0.92799800	-2.81690500
H	-3.32185700	0.78478200	-3.33732100
O	0.97380700	2.43280000	1.45416500
C	1.89514600	1.59538900	1.69504200
O	2.03518000	0.45711600	1.13652000
C	2.94115600	1.96552400	2.71660400
H	3.16857000	1.09769400	3.35079200
H	3.86644700	2.24470000	2.18696300
H	2.60603000	2.81471900	3.32440100
O	1.90054700	0.77963300	-1.75694700
C	1.74929100	2.02661200	-1.97552400
O	0.89294700	2.78601000	-1.42407200
C	2.68979900	2.63446300	-2.98674900
H	2.51894600	3.71331100	-3.08112600
H	3.72692200	2.43657200	-2.67636500

H	2.54087300	2.14337300	-3.96040000
O	-0.55878100	-0.96755200	1.06796300
C	-1.42513500	-0.20885900	1.62408800
O	-1.60121000	1.02311800	1.38219900
C	-2.29065100	-0.84885400	2.68178500
H	-1.71967400	-0.88581800	3.62385200
H	-3.20170200	-0.25987100	2.84351000
H	-2.53915500	-1.88059500	2.39836800
C	1.97451700	-5.47420800	0.17703400
C	3.24900300	-5.85655300	-0.36191300
C	1.60534800	-5.83095600	1.47205800
C	4.15290200	-6.60108400	0.46435200
C	2.51517600	-6.58330800	2.25927000
C	3.75100300	-6.95225900	1.77949200
H	2.21635500	-6.86814900	3.27039200
H	4.43856000	-7.52452300	2.40642700
C	0.28367700	-5.42113200	2.07048400
H	0.34763500	-4.39935700	2.48186300
H	-0.52966200	-5.43092600	1.32993400
H	0.00236800	-6.09158400	2.89483300
H	0.08379100	-3.16415800	0.46053400
C	-1.31488700	-4.41978300	-3.42385100
C	0.34603800	-6.53380400	-2.35111500

C	3.67156400	-5.49149100	-1.67180800
C	5.42795900	-6.96425900	-0.05013200
H	-1.93977200	-3.62725700	-3.83531100
H	0.97516200	-7.32278100	-1.93880300
H	3.00960700	-4.90186600	-2.30367100
H	6.10571800	-7.53294100	0.59068000
C	-1.27105700	-5.67711500	-3.97445900
C	-0.45036400	-6.73723500	-3.44417000
C	4.91753400	-5.85490300	-2.13818100
C	5.80591700	-6.60082700	-1.32450500
H	-0.47675400	-7.71079600	-3.93478500
H	-1.88383500	-5.89222800	-4.85233200
H	6.78958900	-6.87932900	-1.70748300
H	5.22502000	-5.55658500	-3.14227300

TS2

C	-0.33526000	-4.10799200	-2.29186200
C	0.76411400	-4.93682600	-1.95399500
C	1.20570100	-4.55887000	-0.62411300
C	0.39689900	-3.36487600	-0.25064200
N	-0.62331000	-3.24708700	-1.25417600
H	-0.97295300	-2.30976300	-1.46740600
C	1.79150300	-2.72946300	-0.49375000

Rh	0.67563900	-0.07160400	-0.31689700
C	2.33608200	-2.26363800	-1.77318600
Br	2.82469000	-2.62924800	1.13945900
Rh	-0.39720400	2.03441800	0.08114500
C	3.74836100	-1.76101500	-1.91045700
O	1.65081600	-2.37201400	-2.79877200
C	4.33771400	-0.79039900	-1.08710300
C	4.47028500	-2.25249000	-3.01109900
C	5.63466600	-0.34305700	-1.35201700
H	3.78039600	-0.35994300	-0.25879500
C	5.77469700	-1.82357200	-3.25774700
H	3.99266300	-2.97897100	-3.67010400
C	6.36228800	-0.86475900	-2.42585400
H	6.07860800	0.42273100	-0.71194400
H	6.32961500	-2.23079400	-4.10563400
H	7.38022300	-0.51942000	-2.61936200
O	-1.87030900	1.49517100	-1.25820600
C	-1.81599700	0.35339000	-1.80566100
O	-0.88299100	-0.50227600	-1.61678800
C	-2.92547300	-0.05137100	-2.73746300
H	-2.49767500	-0.48737500	-3.65128400
H	-3.53137900	-0.82970900	-2.24626800
H	-3.56323400	0.80670700	-2.97929500

O	1.11028300	2.50473700	1.39582400
C	2.06259400	1.67505900	1.55338200
O	2.14181900	0.53278400	0.99497300
C	3.20350900	2.07625500	2.44903300
H	3.59365200	1.19723800	2.97846600
H	4.01179100	2.48257400	1.81920800
H	2.88491000	2.85162300	3.15643500
O	1.70197300	0.82819900	-1.85202700
C	1.51741700	2.06473100	-2.09189700
O	0.71580800	2.82854200	-1.45952400
C	2.30440400	2.65690400	-3.23026600
H	2.27262900	3.75240200	-3.19676000
H	3.34031100	2.29304000	-3.18925800
H	1.86555500	2.30868200	-4.17884200
O	-0.42877500	-0.85964200	1.23739600
C	-1.24017500	-0.10238300	1.86706100
O	-1.44672100	1.12701100	1.61295200
C	-1.99315700	-0.72206600	3.01416900
H	-1.29091400	-0.88373600	3.84742000
H	-2.80610200	-0.06568300	3.34599700
H	-2.38561300	-1.70370800	2.71357100
C	1.94233700	-5.47038100	0.27505700
C	3.30304300	-5.81974500	-0.02796600

C	1.29832100	-6.02666800	1.37985800
C	4.00414800	-6.72204500	0.83545000
C	2.01386100	-6.93059800	2.21109500
C	3.32348700	-7.26440200	1.95735100
H	1.49824800	-7.36641600	3.06971900
H	3.85506400	-7.95679100	2.61421800
C	-0.13801100	-5.71997800	1.72788500
H	-0.21251800	-4.79521400	2.32498600
H	-0.76592200	-5.58928000	0.83596600
H	-0.56738700	-6.53178900	2.33202300
H	0.05840600	-3.17529800	0.76699000
C	-0.99992300	-4.25463200	-3.51479700
C	1.18246100	-5.95231600	-2.82570100
C	4.00518300	-5.27457400	-1.13763000
C	5.36119900	-7.04500900	0.55838500
H	-1.83417200	-3.60409400	-3.78116700
H	2.01260000	-6.60496800	-2.55482600
H	3.49454600	-4.58698900	-1.80490400
H	5.88006200	-7.73473700	1.22822700
C	-0.55906200	-5.25933200	-4.37557700
C	0.51383200	-6.10953100	-4.03782500
C	5.32340200	-5.59884300	-1.37873200
C	6.01246700	-6.49467800	-0.52466600

H	0.82137700	-6.89250600	-4.73251000
H	-1.06333200	-5.39502300	-5.33494300
H	7.05655000	-6.74315800	-0.72473700
H	5.83922800	-5.15163700	-2.23051300

C

C	1.24049000	-3.87321100	-1.91976700
C	2.30409700	-4.40177200	-1.15324900
C	1.96023300	-4.27300900	0.30379000
C	0.56668100	-3.62777300	0.26648900
N	0.16380700	-3.54385200	-1.10674100
H	-0.50474400	-2.82350900	-1.38624600
C	1.73703500	-2.80464400	0.73308500
Rh	-0.86923300	-0.17887800	-0.06598300
C	2.15027600	-1.54775500	-0.00943100
Br	1.76470500	-2.38687900	2.63954200
Rh	-3.06244800	0.71748400	0.29209600
C	3.56514100	-1.10235800	-0.09820400
O	1.28394300	-0.78263700	-0.43498900
C	3.82459400	0.23689700	0.24875600
C	4.61721600	-1.92477900	-0.52651400
C	5.12734100	0.72914400	0.20991900
H	2.99657900	0.86910600	0.57192000

C	5.91635800	-1.41775000	-0.58882400
H	4.41649200	-2.94159300	-0.85244100
C	6.17612600	-0.09758700	-0.20959700
H	5.32627800	1.76115900	0.50456400
H	6.72743600	-2.05882400	-0.93850800
H	7.19587400	0.29074200	-0.24679000
O	-3.74283700	-0.53450600	-1.21276400
C	-2.92789200	-1.30531200	-1.80123300
O	-1.66989000	-1.36162500	-1.56433000
C	-3.45198000	-2.23184500	-2.86548300
H	-2.95342800	-2.00347400	-3.81971500
H	-3.19637100	-3.26880400	-2.59920000
H	-4.53744300	-2.12659500	-2.97428200
O	-2.26302600	1.89717000	1.77945400
C	-1.01833400	1.81515300	2.03296900
O	-0.19257300	1.04332000	1.44366100
C	-0.46361100	2.72391600	3.09850700
H	0.36825600	2.23326400	3.62024300
H	-0.07579900	3.63429400	2.61290500
H	-1.25227700	3.01312200	3.80415700
O	-0.54062900	1.33133900	-1.43207700
C	-1.46864800	2.17705200	-1.64899200
O	-2.61696100	2.17225600	-1.09795900

C	-1.17212700	3.29302900	-2.61693300
H	-2.09180800	3.61053300	-3.12500500
H	-0.77897500	4.15141700	-2.04792200
H	-0.41146200	2.97972300	-3.34285500
O	-1.33884800	-1.64434100	1.30720200
C	-2.47952000	-1.63596300	1.87473200
O	-3.39765500	-0.77894100	1.67298500
C	-2.75321100	-2.75456400	2.84607400
H	-2.96160400	-3.67293400	2.27333800
H	-1.86042300	-2.93772900	3.45962300
H	-3.61989700	-2.52141400	3.47603600
C	2.33182700	-5.39053100	1.23698500
C	3.57246200	-5.37658200	1.95782800
C	1.49063700	-6.50030100	1.31803000
C	3.91211900	-6.48460300	2.80414200
C	1.84700200	-7.58164000	2.17043800
C	3.01169300	-7.57667600	2.89960100
H	1.17149700	-8.43795000	2.23176400
H	3.26646100	-8.41993500	3.54575900
C	0.20972600	-6.62128300	0.52705300
H	-0.61862300	-6.08126000	1.01500100
H	0.30223700	-6.21741600	-0.48958300
H	-0.09006700	-7.67614200	0.45072900

H	-0.20551300	-3.84590900	0.99793700
C	1.34010400	-3.80134000	-3.31385500
C	3.41851400	-4.96065600	-1.76910300
H	4.21307400	-5.40684300	-1.16714700
H	0.53491400	-3.36821500	-3.90961300
C	3.51290000	-4.91556100	-3.16793600
C	2.48882100	-4.32383500	-3.92093800
H	2.57904900	-4.27985800	-5.00855500
H	4.38802500	-5.33210600	-3.66901900
C	4.50724500	-4.30897500	1.86359600
C	5.14520700	-6.47559900	3.51386200
H	5.38132800	-7.32776600	4.15565600
H	4.27493100	-3.45366200	1.24063700
C	6.02719700	-5.42395300	3.39220500
C	5.70091000	-4.33073600	2.55220800
H	6.97234900	-5.42968300	3.93876700
H	6.39474000	-3.49351000	2.45120100

A”

C	4.90632400	6.73309200	-0.06956400
Rh	3.65434300	5.27983500	0.34346200
Rh	2.27529100	3.29822000	0.71581300
O	5.28149000	4.01047200	0.58006700

O	3.99513100	2.19372000	0.98928700
O	3.54153200	4.84462600	-1.67188100
O	2.39843300	2.92128700	-1.32003600
O	1.90282500	6.35900800	0.17840300
O	0.61774200	4.50957500	0.39901800
O	3.63545400	5.60618400	2.37711200
O	2.27560900	3.82299900	2.72309100
C	2.93508700	4.82989700	3.11683300
C	2.87824900	5.18137600	4.58136700
H	3.85738900	5.54147800	4.92420000
H	2.15130500	5.99909100	4.71477500
H	2.55100800	4.31550200	5.16924500
C	0.77877900	5.75620600	0.23131200
C	2.99059200	3.75816800	-2.06279400
C	5.11121500	2.76795700	0.84425900
C	6.36637600	1.94464800	0.96941200
H	6.81900100	1.83995800	-0.02952200
H	7.09097700	2.46450900	1.61169000
H	6.13598400	0.95120100	1.37164300
C	3.08691900	3.47372500	-3.53910700
H	2.51290500	2.57742400	-3.80128300
H	2.72326700	4.34357500	-4.10500400
H	4.14731600	3.33207300	-3.80051800

C	-0.45759300	6.60929100	0.11524600
H	-0.87585600	6.75197300	1.12484500
H	-0.21881100	7.58941000	-0.31391600
H	-1.21145700	6.09196500	-0.49364900
Br	6.14740600	7.41453200	1.15539500
C	5.08646700	7.29947100	-1.44779000
O	4.46430000	8.33662100	-1.65190700
C	5.90300400	6.58406100	-2.44555300
C	6.01609300	7.12324500	-3.73724300
C	6.53569400	5.37126300	-2.12646100
C	6.75783600	6.45175000	-4.70619200
H	5.51024100	8.06324300	-3.96134000
C	7.27736400	4.70396300	-3.10069500
H	6.42515700	4.93454000	-1.13265800
C	7.38791300	5.24139000	-4.38834900
H	6.84568000	6.86606400	-5.71215600
H	7.76600100	3.75854000	-2.85840800
H	7.96691700	4.71423900	-5.14948700
O	0.63355600	1.15748500	0.96360000
C	-0.39543000	1.80683000	1.07250100
C	-1.40076400	1.94570700	-0.03976100
H	-1.18760200	1.18872400	-0.80284400
H	-2.43498800	1.84723700	0.31900400

H	-1.28222700	2.94539100	-0.48674800
C	-1.64101300	3.45550900	2.35887500
H	-1.70386200	4.02233200	1.42157600
H	-2.61055800	2.96850800	2.55568200
C	-1.22176300	4.36146700	3.49810600
H	-1.14854500	3.80089000	4.44250400
H	-0.23819800	4.80135200	3.27835600
H	-1.96065400	5.16789300	3.62663100
O	-0.62166700	2.43582400	2.23651300

TS1”

C	-0.87759200	-4.14339300	-2.50700500
C	-0.04735800	-5.17414300	-1.97150700
C	0.33461100	-4.76059000	-0.64752200
C	-0.27618000	-3.51330800	-0.43775000
N	-1.03530100	-3.18148900	-1.54016200
H	-1.29282900	-2.20672700	-1.70552600
C	1.70098300	-1.88366600	-0.43027800
Rh	0.68840400	-0.14755600	-0.12445600
C	2.36176700	-2.13188700	-1.76381500
Br	2.61098500	-2.66417200	1.03527100
Rh	-0.30699500	2.04847700	0.32333900
C	3.77442500	-1.68657800	-1.95800100

O	1.71301300	-2.62024900	-2.68000200
C	4.39710100	-0.73974300	-1.12775700
C	4.47237600	-2.21821500	-3.05476100
C	5.70595600	-0.33709600	-1.39701000
H	3.85685400	-0.29914500	-0.29072700
C	5.78620400	-1.82753100	-3.30712900
H	3.96721200	-2.94119300	-3.69588800
C	6.40461700	-0.88358200	-2.47884200
H	6.18382700	0.40777600	-0.75764900
H	6.32972100	-2.25582000	-4.15158600
H	7.43220700	-0.57236100	-2.67838900
O	-1.82357300	1.57917800	-1.00557700
C	-1.82257100	0.45408300	-1.57590600
O	-0.93539800	-0.45744000	-1.40658100
C	-2.94412700	0.12855200	-2.52744700
H	-2.52601900	-0.20567200	-3.48835400
H	-3.53784800	-0.70231400	-2.11447900
H	-3.58858300	1.00278800	-2.67499900
O	1.24688300	2.43162600	1.62352200
C	2.16166400	1.56611900	1.74812700
O	2.19289300	0.42486700	1.17040800
C	3.34007000	1.89236200	2.62847800
H	3.66439800	0.99576600	3.17322400

H	4.17394500	2.21872200	1.98574900
H	3.08858100	2.70334400	3.32258100
O	1.69385000	0.74644900	-1.69180800
C	1.56230100	1.99858200	-1.90617900
O	0.83603400	2.79424400	-1.23672900
C	2.34057500	2.55299300	-3.07152800
H	2.30634600	3.64874700	-3.07334800
H	3.37937100	2.19638500	-3.02382200
H	1.90103600	2.16936200	-4.00571900
O	-0.41881900	-0.89277700	1.44953000
C	-1.20526000	-0.11192600	2.08977200
O	-1.37067600	1.12243600	1.85537700
C	-1.97595700	-0.73534600	3.22477800
H	-1.27037400	-0.99349000	4.03007600
H	-2.73168300	-0.04063400	3.60946300
H	-2.44679400	-1.66912400	2.88526700
C	1.18447100	-5.49282800	0.30291200
C	2.51053900	-5.89219100	-0.10145600
C	0.73709000	-5.77683500	1.59672600
C	3.33976500	-6.61912000	0.81322800
C	1.58078600	-6.50097700	2.48113000
C	2.83587700	-6.92037000	2.10575100
H	1.20910100	-6.73222000	3.48208800

H	3.46473900	-7.47992800	2.80218000
C	-0.62001400	-5.34755100	2.09908600
H	-0.58972100	-4.31467500	2.48666300
H	-1.37983000	-5.37348500	1.30610100
H	-0.95014900	-5.99534300	2.92436600
H	-0.37091200	-2.93243000	0.47224600
C	-1.40391600	-4.21302600	-3.80397200
C	0.21730900	-6.31942400	-2.75169400
C	3.07047800	-5.52048200	-1.35685500
C	4.65761000	-6.98942700	0.42585100
H	-2.02428600	-3.40905600	-4.20406800
H	0.83532700	-7.12533700	-2.35351400
H	2.47626000	-4.92889100	-2.04849900
H	5.26999200	-7.55237300	1.13448000
C	-1.10954700	-5.34565200	-4.55587500
C	-0.31383300	-6.39438000	-4.03194900
C	4.35917800	-5.87590400	-1.69749800
C	5.16118300	-6.62892300	-0.80522400
H	-0.11671400	-7.27247900	-4.65001100
H	-1.50501500	-5.43268800	-5.57004800
H	6.17786400	-6.90831000	-1.08892700
H	4.76680600	-5.56280900	-2.66071700
O	-1.55098400	4.27081800	0.49184400

C	-2.72310000	4.14079200	0.16345100
C	-3.74585000	3.36965900	0.94943700
O	-3.24534700	4.70124000	-0.93510400
H	-3.26112600	2.50985300	1.42825600
H	-4.57764600	3.04935400	0.30973900
H	-4.14551900	4.02813600	1.73844600
C	-2.34769900	5.46146900	-1.79117900
C	-1.69676400	4.56508600	-2.82902100
H	-1.59830600	5.96088100	-1.16241700
H	-2.99911900	6.21456100	-2.25490100
H	-1.11524700	5.18648500	-3.52983600
H	-2.46055000	4.01916200	-3.40508200
H	-1.01615600	3.84308000	-2.35865500

B”

C	-0.49512700	-4.18172300	-2.33954600
C	0.33064300	-5.20899300	-1.73378400
C	0.97874600	-4.64850200	-0.65188800
C	0.55002500	-3.20352700	-0.54356300
N	-0.37814300	-3.05746300	-1.65705000
H	-0.72413100	-2.12618800	-1.94521600
C	1.64689600	-2.09659300	-0.51205400
Rh	0.62426600	-0.06910600	-0.50459700

C	2.55704700	-2.14897000	-1.67853200
Br	2.57904400	-2.34036800	1.23720100
Rh	-0.54348500	2.08148900	-0.29318000
C	3.91260300	-1.50646000	-1.68080500
O	2.16707200	-2.71057200	-2.71382900
C	4.29325300	-0.44960300	-0.83790900
C	4.83238800	-1.98706000	-2.62757200
C	5.56850500	0.10728300	-0.94610800
H	3.59099000	-0.04550600	-0.11419200
C	6.11272800	-1.44109300	-2.72215600
H	4.52261500	-2.79639500	-3.28844000
C	6.48451000	-0.38768700	-1.88089900
H	5.84879800	0.93708600	-0.29317400
H	6.82028400	-1.83524000	-3.45487500
H	7.48392400	0.04730500	-1.95411000
O	-1.80632900	1.49705800	-1.83555700
C	-1.65995700	0.35904800	-2.35471800
O	-0.75099100	-0.48843200	-2.02687500
C	-2.60493000	-0.07348900	-3.44608500
H	-2.02596700	-0.37407600	-4.33190400
H	-3.17203800	-0.95401700	-3.10464900
H	-3.29937600	0.73486300	-3.70230400
O	0.76577900	2.58430700	1.21811600

C	1.68898300	1.76887800	1.52270000
O	1.86961500	0.62176900	0.99990400
C	2.67509700	2.18892100	2.58345900
H	2.88900100	1.34342500	3.25157900
H	3.61972900	2.47593100	2.09371100
H	2.28958500	3.04573300	3.14944800
O	1.83496800	0.84025700	-1.91281800
C	1.68841100	2.07789100	-2.17049000
O	0.80543100	2.84619700	-1.67209000
C	2.65999900	2.66643400	-3.16340200
H	2.51569100	3.74902200	-3.26006900
H	3.68728900	2.44134700	-2.84025400
H	2.51104000	2.18265800	-4.14100400
O	-0.69993700	-0.83974700	0.88904100
C	-1.59294900	-0.07637300	1.38975800
O	-1.78684500	1.14009300	1.08671300
C	-2.47018300	-0.67870100	2.45949200
H	-1.92907400	-0.63070000	3.41872900
H	-3.40500100	-0.11291200	2.55493700
H	-2.67407100	-1.73517800	2.23933700
C	1.88139600	-5.37003700	0.26236600
C	3.18119700	-5.75504500	-0.20917000
C	1.46167200	-5.68705200	1.55215800

C	4.05626900	-6.46346700	0.67761800
C	2.34367700	-6.40552100	2.40044100
C	3.60173500	-6.77779300	1.98486200
H	2.00503600	-6.66038200	3.40705000
H	4.26694400	-7.32309100	2.65817300
C	0.11340500	-5.26737300	2.08076900
H	0.14938400	-4.22694300	2.44661100
H	-0.66999600	-5.32066200	1.31027800
H	-0.19250700	-5.90329600	2.92350700
H	-0.02257700	-3.06014600	0.38937800
C	-1.27062000	-4.46173200	-3.49846700
C	0.35690300	-6.52762100	-2.29004100
C	3.65590200	-5.42352200	-1.51005000
C	5.35628600	-6.82685500	0.22966500
H	-1.88361800	-3.68870200	-3.96169800
H	0.97307100	-7.29808800	-1.82622200
H	3.01604900	-4.85906400	-2.18629200
H	6.01193700	-7.36786600	0.91594700
C	-1.20065600	-5.73744800	-4.00232500
C	-0.39633800	-6.77387600	-3.40478300
C	4.92512300	-5.78493900	-1.91068000
C	5.78514700	-6.49624800	-1.03752100
H	-0.40009400	-7.76422200	-3.86132500

H	-1.77923600	-5.98651900	-4.89436100
H	6.78755800	-6.77377600	-1.36927500
H	5.27338100	-5.51178000	-2.90860300
O	-1.58382600	4.37569300	0.29275200
C	-0.73773200	5.06462800	0.84665700
C	0.45874100	5.65479700	0.15468600
O	-0.79158700	5.39825000	2.14396200
H	0.88906800	4.89895000	-0.51561200
H	1.20313600	6.01156700	0.87676400
H	0.12073200	6.50443000	-0.46092500
C	-1.89186900	4.86251400	2.92946400
C	-1.50540800	3.54519100	3.57735900
H	-2.76832000	4.74938000	2.27749100
H	-2.08911500	5.64143500	3.67891000
H	-2.31883900	3.22037400	4.24714800
H	-0.58994800	3.66125300	4.17874600
H	-1.34208500	2.76508500	2.82217400

TS2”

C	-0.33839400	-4.07742500	-2.23158300
C	0.71702900	-4.97399100	-1.91820700
C	1.20648000	-4.62850900	-0.60372100
C	0.49093900	-3.37765900	-0.20999000

N	-0.55179700	-3.20488800	-1.18904500
H	-0.86560500	-2.25250000	-1.39205200
C	1.88113200	-2.76076900	-0.48140000
Rh	0.76108600	-0.03811000	-0.26604700
C	2.39754300	-2.31233000	-1.76708200
Br	2.96849200	-2.70783400	1.11348000
Rh	-0.30629000	2.07944200	0.14373100
C	3.80635000	-1.80421600	-1.92871700
O	1.69483500	-2.41461800	-2.78586200
C	4.39103800	-0.81167300	-1.12836200
C	4.52359600	-2.30442600	-3.02779500
C	5.67885300	-0.35097800	-1.41438800
H	3.83659900	-0.37598600	-0.30044500
C	5.81915200	-1.86127700	-3.29657100
H	4.04853400	-3.04705100	-3.67060000
C	6.40213200	-0.88027500	-2.48774300
H	6.11896200	0.43156500	-0.79197500
H	6.37063500	-2.27505500	-4.14364600
H	7.41282200	-0.52376200	-2.69863800
O	-1.77445400	1.55630200	-1.20999300
C	-1.72260700	0.41553000	-1.76070000
O	-0.79442900	-0.44450300	-1.57786200
C	-2.83704200	0.02051700	-2.69238700

H	-2.41410000	-0.41928600	-3.60659400
H	-3.44900900	-0.75261000	-2.20038900
H	-3.46831300	0.88352300	-2.93395100
O	1.20872300	2.51258200	1.48028900
C	2.15709900	1.67351700	1.62770100
O	2.22891700	0.53774400	1.06231600
C	3.29680400	2.07519900	2.52670900
H	3.86106200	1.19188900	2.84943200
H	3.96886300	2.73963700	1.95934600
H	2.91848500	2.63532900	3.39226700
O	1.81137200	0.87331800	-1.78143100
C	1.63644900	2.11008500	-2.01352100
O	0.83044300	2.87563600	-1.38684100
C	2.43215100	2.71293900	-3.14154700
H	2.57803100	3.78842500	-2.97957300
H	3.39468300	2.19449600	-3.23893800
H	1.86969300	2.57326200	-4.07904400
O	-0.36624200	-0.82261400	1.27976300
C	-1.17139100	-0.06303400	1.91079400
O	-1.37571500	1.16865400	1.66130100
C	-1.92200100	-0.67062200	3.06735100
H	-1.27496100	-0.63235200	3.95892100
H	-2.83830300	-0.10409100	3.27411200

H	-2.15203300	-1.72379200	2.85892500
C	1.97227100	-5.54142300	0.26320200
C	3.30583600	-5.92520300	-0.11387100
C	1.38693000	-6.06089400	1.41801000
C	4.03472400	-6.83575900	0.71723000
C	2.12909200	-6.97187300	2.21712600
C	3.41007700	-7.34666000	1.88561200
H	1.65791900	-7.38053300	3.11384000
H	3.96277200	-8.04565900	2.51765200
C	-0.01367800	-5.70347900	1.85174400
H	-0.01830500	-4.76981200	2.43980600
H	-0.69146000	-5.56042700	0.99912400
H	-0.43098000	-6.49326300	2.49256000
H	0.17408700	-3.18886300	0.81577700
C	-1.03542300	-4.18171400	-3.44161600
C	1.05372100	-6.01175700	-2.80181500
C	3.95687200	-5.40152000	-1.26462700
C	5.36483500	-7.19514600	0.36476100
H	-1.83286400	-3.48020000	-3.69050500
H	1.84858000	-6.71383300	-2.54909000
H	3.42544700	-4.70263300	-1.90388100
H	5.90508200	-7.89188200	1.01003600
C	-0.67485000	-5.20932600	-4.31186200

C	0.35126800	-6.12546100	-3.99847500
C	5.25102600	-5.75999100	-1.57751900
C	5.96540400	-6.66879100	-0.75875600
H	0.59467500	-6.92417100	-4.70064900
H	-1.20624400	-5.31123000	-5.26081300
H	6.98979700	-6.94451700	-1.01670900
H	5.72954300	-5.32880100	-2.45881800
O	-1.39465500	4.00213900	0.66366200
C	-0.85990200	5.05873800	1.00178200
C	0.51287500	5.49349100	0.59260400
O	-1.47616300	5.92151500	1.80324300
H	0.68409700	5.20991700	-0.45363500
H	1.23382200	4.92357600	1.19956200
H	0.65355500	6.56859100	0.75326100
C	-2.78275300	5.52595400	2.32394500
C	-2.65321600	4.51553000	3.44972600
H	-3.38365000	5.13316300	1.49223900
H	-3.21364500	6.47302500	2.67217400
H	-3.64927900	4.33070100	3.88300200
H	-1.99841900	4.90167300	4.24650000
H	-2.25338600	3.55950400	3.08197000

C”

C	1.22158400	-3.86649600	-1.90827800
C	2.29086100	-4.39387900	-1.14824300
C	1.96144400	-4.25344900	0.31108500
C	0.57169000	-3.59904200	0.28229600
N	0.15545000	-3.52521300	-1.08757900
H	-0.51677200	-2.80783400	-1.36664100
C	1.75219100	-2.78054300	0.73033600
Rh	-0.91928100	-0.17116400	-0.06396500
C	2.16576400	-1.53082500	-0.02719500
Br	1.79914700	-2.34894200	2.63414600
Rh	-3.12785300	0.69709000	0.30193300
C	3.58430200	-1.08957500	-0.11180700
O	1.29820100	-0.77799900	-0.46405300
C	3.85159000	0.24371600	0.24918000
C	4.63030600	-1.91199900	-0.55384800
C	5.15683300	0.73026300	0.21100500
H	3.02751300	0.87698500	0.58072300
C	5.93210400	-1.41081100	-0.61534700
H	4.42292100	-2.92467500	-0.88951600
C	6.20010300	-0.09660700	-0.22167800
H	5.36189700	1.75809400	0.51613700
H	6.73867400	-2.05193900	-0.97542900
H	7.22164800	0.28711800	-0.25819800

O	-3.80414500	-0.58342200	-1.18032300
C	-2.97961400	-1.34388400	-1.76904600
O	-1.71942000	-1.37689000	-1.54404900
C	-3.49787500	-2.28340900	-2.82568500
H	-3.06801600	-1.99984900	-3.79882900
H	-3.16046800	-3.30615900	-2.60032900
H	-4.59203600	-2.24653400	-2.87822900
O	-2.32473600	1.91167700	1.77049400
C	-1.07957800	1.83082700	2.03152900
O	-0.24840300	1.07538000	1.43553200
C	-0.56123300	2.68590300	3.15886600
H	0.52654600	2.80516000	3.08625500
H	-1.06232100	3.66319100	3.15191900
H	-0.80137200	2.18808100	4.11246100
O	-0.62531200	1.33743200	-1.44512100
C	-1.56715300	2.16384900	-1.66706600
O	-2.71532000	2.14420700	-1.11475100
C	-1.29756800	3.27867600	-2.64531800
H	-2.21190300	3.52363100	-3.20184000
H	-0.99304500	4.17371000	-2.07851300
H	-0.48582900	3.00358700	-3.32984000
O	-1.36418100	-1.62903700	1.33037500
C	-2.49738800	-1.62523300	1.91038300

O	-3.42812600	-0.78093400	1.71067800
C	-2.75166500	-2.73527300	2.89795000
H	-2.99638000	-3.65254200	2.33776100
H	-1.84067800	-2.92955700	3.48029900
H	-3.59230100	-2.48722800	3.55716500
C	2.33529800	-5.36656700	1.24834000
C	3.58443400	-5.35573000	1.95426800
C	1.48715100	-6.46940400	1.34884600
C	3.92654200	-6.45894700	2.80579700
C	1.84598500	-7.54604900	2.20619100
C	3.01945800	-7.54359700	2.92127000
H	1.16509700	-8.39685800	2.28299000
H	3.27586600	-8.38331300	3.57140900
C	0.19617700	-6.58730500	0.57396800
H	-0.62242100	-6.03729800	1.06715000
H	0.27974800	-6.19155300	-0.44659200
H	-0.11209300	-7.64053100	0.50929500
H	-0.19524000	-3.80491100	1.02274100
C	1.30799900	-3.80576800	-3.30389100
C	3.39757600	-4.96037900	-1.77083100
H	4.19661600	-5.40443200	-1.17320800
H	0.49879400	-3.37350700	-3.89486000
C	3.47855000	-4.92618300	-3.17096100

C	2.44880200	-4.33707000	-3.91823700
H	2.52842600	-4.30154000	-5.00703300
H	4.34758400	-5.34901600	-3.67740800
C	4.52514600	-4.29542400	1.83929600
C	5.16832300	-6.45252200	3.50027700
H	5.40654100	-7.30088900	4.14635800
H	4.29075600	-3.44424200	1.21138300
C	6.05590200	-5.40802500	3.35886200
C	5.72697000	-4.31965900	2.51349600
H	7.00765600	-5.41579200	3.89385400
H	6.42532500	-3.48825800	2.39663800
O	-5.27239200	1.68238400	0.54692600
C	-5.44155400	2.57424200	1.37491700
C	-5.35236300	2.37816400	2.85838600
O	-5.68745300	3.83652200	1.02663000
H	-5.76899300	1.39829200	3.12509700
H	-5.85569600	3.18532700	3.40410800
H	-4.28008800	2.37218200	3.11343400
C	-5.64089300	4.14576900	-0.40166900
C	-6.95078400	3.80329300	-1.08554100
H	-4.79133800	3.60641700	-0.84273200
H	-5.44536100	5.22585300	-0.42119100
H	-6.91245300	4.13780400	-2.13463400

H	-7.79608600	4.30826900	-0.59244800
H	-7.12512900	2.71789400	-1.07559500

2c

C	4.09124600	-2.14232300	-0.13291900
Br	2.19133200	-2.15843200	-0.08882800
C	4.86337900	-3.24358800	0.46125200
O	4.28906000	-4.12542500	1.08760000
C	6.35114900	-3.23169500	0.30223300
C	7.12258200	-3.71080700	1.37384800
C	6.98718000	-2.82780400	-0.88210600
C	8.51234400	-3.74968100	1.27563100
H	6.61503700	-4.04607200	2.27904100
C	8.37891700	-2.88396400	-0.98331800
H	6.40181000	-2.49914900	-1.74193300
C	9.14304100	-3.33473500	0.09681300
H	9.10706100	-4.10924500	2.11744700
H	8.86652600	-2.58038400	-1.91141000
H	10.23138100	-3.37052100	0.01776900
N	4.63560600	-1.02927600	-0.55932500
N	5.09172700	-0.05554400	-0.93202500

N_2

N	3.46626700	-2.10977100	-5.68200800
N	2.36201200	-2.10977100	-5.68200800

C'

C	1.27430800	-3.84623600	-1.87938200
C	2.32075800	-4.40266700	-1.11740100
C	1.98043500	-4.27984800	0.34091500
C	0.59849400	-3.61107000	0.31427700
N	0.18617200	-3.49574000	-1.06441600
H	-0.29083200	-2.62759200	-1.29808500
C	1.78379600	-2.81158800	0.77598700
C	2.13229900	-1.52578400	0.02178800
Br	1.81467100	-2.40354000	2.68717200
C	3.53825400	-1.04745100	-0.10188200
O	1.20387600	-0.83671000	-0.37145700
C	3.77029300	0.32791400	0.07563300
C	4.61014600	-1.88904800	-0.43209200
C	5.06069200	0.84318900	-0.02915800
H	2.92659600	0.97718200	0.31235000
C	5.89862300	-1.36631400	-0.56003600
H	4.43449800	-2.94129400	-0.63708400
C	6.12889600	-0.00438000	-0.34644800
H	5.23592900	1.90836000	0.13330500

H	6.72354000	-2.02723500	-0.83227000
H	7.13950500	0.39955000	-0.43405000
C	2.33467200	-5.41810300	1.25667300
C	3.57097600	-5.43038100	1.98548500
C	1.48573600	-6.52430300	1.30448300
C	3.89846800	-6.56344800	2.80337400
C	1.82896700	-7.62873200	2.13215500
C	2.98988700	-7.65087400	2.86668400
H	1.14691000	-8.48125200	2.16797300
H	3.23562400	-8.51226300	3.49206400
C	0.21100200	-6.62042600	0.50031300
H	-0.61855300	-6.08420100	0.99011800
H	0.31556600	-6.20028800	-0.50854600
H	-0.09559700	-7.67143600	0.40187500
H	-0.17390300	-3.85415900	1.04243400
C	1.37416200	-3.76438600	-3.27035100
C	3.43157500	-4.96822100	-1.73450700
H	4.21846400	-5.43057200	-1.13506300
H	0.57549800	-3.31585600	-3.86370800
C	3.52993700	-4.90807400	-3.13207500
C	2.51648400	-4.29665500	-3.88236000
H	2.60867600	-4.24483100	-4.96928700
H	4.40082700	-5.33111900	-3.63522600

C	4.51453800	-4.36745600	1.92626800
C	5.12850700	-6.58423300	3.51806300
H	5.35449900	-7.45569900	4.13715800
H	4.29035600	-3.49108500	1.33007100
C	6.01968100	-5.53728300	3.42891500
C	5.70494100	-4.41825900	2.61920400
H	6.96260300	-5.56591800	3.97855700
H	6.40527200	-3.58360200	2.54600400

TS3

C	1.22227900	-3.81034800	-2.03632400
C	2.26734800	-4.36916500	-1.23937400
C	2.14921500	-4.22976100	0.18744500
C	0.25121500	-3.36412000	0.05619100
N	0.11369900	-3.42409400	-1.30193000
H	-0.81074400	-3.55210700	-1.69868500
C	1.54728300	-2.90123900	0.58259600
C	2.06527700	-1.56474800	-0.01663700
Br	1.44473300	-2.59885800	2.53551700
C	3.54447700	-1.31170500	-0.05804300
O	1.25955900	-0.75812200	-0.43325600
C	4.16147100	-0.61965400	0.99384900
C	4.30751200	-1.75057900	-1.14882100

C	5.53976800	-0.39614900	0.96713600
H	3.56497200	-0.27624700	1.83985600
C	5.68535100	-1.52249300	-1.17001200
H	3.82863200	-2.28029800	-1.97160200
C	6.30491300	-0.85380200	-0.11018400
H	6.01738000	0.13364100	1.79366900
H	6.27645100	-1.87484000	-2.01760000
H	7.38360600	-0.68577100	-0.12497200
C	2.60864700	-5.30401800	1.10660600
C	3.80937300	-5.15976200	1.88530800
C	1.87743700	-6.49398300	1.18032300
C	4.20592500	-6.20418100	2.78489200
C	2.30027100	-7.52091700	2.06796400
C	3.41628800	-7.38067800	2.85920000
H	1.70850700	-8.43755700	2.12301400
H	3.71598300	-8.17871200	3.54274200
C	0.61697600	-6.71363400	0.38181600
H	-0.20705000	-6.09265300	0.77052400
H	0.74506300	-6.44149500	-0.67525800
H	0.30217700	-7.76559000	0.43615000
H	-0.61222300	-3.55582200	0.68847300
C	1.29139500	-3.80514700	-3.44164200
C	3.31552500	-5.04425300	-1.90959500

H	4.10479000	-5.50745200	-1.31522100
H	0.49400700	-3.34088100	-4.02527500
C	3.36941600	-5.05980400	-3.29456200
C	2.36622000	-4.42470600	-4.06258800
H	2.42920500	-4.43280200	-5.15222600
H	4.20455900	-5.55058700	-3.79818100
C	4.65343400	-4.02192400	1.77449900
C	5.38754900	-6.04864400	3.56204200
H	5.66847400	-6.85123700	4.24844600
H	4.38984700	-3.24132100	1.06921400
C	6.16783300	-4.91828200	3.44683900
C	5.79810000	-3.89983700	2.53359600
H	7.07367300	-4.81030400	4.04704600
H	6.42217600	-3.01106100	2.42322700

D

C	0.93771100	-4.90687800	-2.31039500
C	2.19506200	-4.74352700	-1.65783400
C	2.22904800	-4.19208000	-0.33696100
C	-0.16943300	-3.90496800	-0.48344800
N	-0.19697900	-4.47938200	-1.67235900
H	-1.15053400	-4.59995600	-2.17242400
C	1.02914300	-3.76151400	0.23951300

C	0.92388500	-2.99102100	1.53618800
C	1.62414400	-3.46726000	2.75382000
O	0.24145100	-1.97535000	1.53340600
C	1.85718000	-4.83163200	2.97950300
C	2.01965800	-2.52220300	3.71385200
C	2.48436400	-5.24797400	4.15349500
H	1.53346100	-5.56791400	2.24322300
C	2.67090700	-2.93798900	4.87284000
H	1.82915800	-1.46594500	3.52188700
C	2.90166400	-4.30143900	5.09459000
H	2.65533000	-6.31117500	4.33065800
H	2.99994000	-2.20107300	5.60762500
H	3.40870100	-4.62688200	6.00514400
C	3.52469000	-4.04812300	0.38184300
C	4.01236900	-2.72744100	0.66743800
C	4.20444500	-5.17535200	0.83209500
C	5.18795900	-2.58337900	1.47242100
C	5.38489400	-5.00030100	1.60563300
C	5.85576800	-3.75172500	1.92811400
H	5.90267300	-5.88962100	1.97110200
H	6.74977000	-3.64026500	2.54553500
C	3.72279100	-6.58500100	0.59736100
H	2.68688000	-6.63238000	0.24044800

H	4.35346600	-7.09439900	-0.15021900
H	3.79153200	-7.16713900	1.52908300
H	-1.12318100	-3.56123000	-0.08345800
C	0.84299300	-5.47590000	-3.59900200
C	3.36431300	-5.13078400	-2.36661100
H	4.33595300	-4.98344000	-1.89771800
H	-0.14326800	-5.58553300	-4.05229100
C	3.26617500	-5.67752500	-3.62862700
C	2.00212900	-5.85923900	-4.24281400
H	1.94546100	-6.29624800	-5.24079200
H	4.17036500	-5.96978800	-4.16428100
C	3.37533100	-1.54814500	0.19034500
C	5.65507100	-1.28321900	1.80136500
H	6.54864700	-1.19210900	2.42285400
H	2.49731900	-1.62773800	-0.44949800
C	4.99791100	-0.15941900	1.34678200
C	3.85231400	-0.29753800	0.52703200
H	5.36215000	0.83567000	1.60843900
H	3.34258700	0.59244700	0.15367800
Br	-2.91960200	-4.83939000	-3.12005200

DIPEA

N	4.61598900	-1.37175500	0.05252000

C	4.59183700	-0.65357000	1.34219100
H	4.55548100	-1.42933400	2.11961100
C	5.64592000	-2.42072800	-0.07477300
H	6.64552900	-1.96301900	-0.26302400
C	4.56858000	-0.48033600	-1.11914700
H	4.64396400	0.57139200	-0.79935400
H	5.45365300	-0.64855700	-1.75854100
C	3.29277600	-0.65012900	-1.94533200
H	3.29558500	0.02701300	-2.81686500
H	3.19004500	-1.68302300	-2.31147100
H	2.40348500	-0.42289700	-1.33680400
C	5.76447700	-3.28762800	1.18230200
H	6.43488100	-4.13607300	0.97682300
H	6.17923400	-2.74445600	2.04291300
H	4.77927000	-3.69300900	1.46741500
C	5.32689300	-3.33049800	-1.26847900
H	4.35534400	-3.82830000	-1.11400500
H	5.27935600	-2.77897000	-2.21786900
H	6.10473300	-4.10320800	-1.37404500
C	3.29780400	0.15139700	1.49315300
H	3.24842700	0.99999000	0.79211700
H	2.42349200	-0.49247500	1.31314200
H	3.22628500	0.56333400	2.51227600

C	5.83075800	0.21619200	1.61707800
H	5.77976000	0.65240100	2.62799000
H	6.75866800	-0.37375700	1.55277000
H	5.91197600	1.04989500	0.90043600

HDIPEA^{+}•••Br^{-}

N	4.51389400	-1.37063900	0.12931000
C	4.45512500	-0.66343500	1.48593400
H	4.43653100	-1.48267700	2.21365600
C	5.63879200	-2.41185600	0.01100400
H	6.56293600	-1.81824800	-0.07368700
C	4.51847700	-0.39074000	-1.04083400
H	4.40334900	0.61171700	-0.61799300
H	5.52451300	-0.43243200	-1.48337900
C	3.39909600	-0.67080100	-2.02800300
H	3.45011000	0.07350100	-2.83661400
H	3.48217800	-1.66670200	-2.48528500
H	2.40769800	-0.58128100	-1.55503700
C	5.67328600	-3.33185200	1.22573700
H	6.41124700	-4.12245500	1.02884100
H	5.98668900	-2.82213000	2.14612700
H	4.70117000	-3.82365100	1.40053700
C	5.46089600	-3.20465300	-1.27894500

H	4.49674900	-3.73981700	-1.30363900
H	5.55585700	-2.57409100	-2.17155800
H	6.26365400	-3.95343600	-1.33085600
C	3.14025000	0.09843400	1.61669200
H	3.08560900	0.97474600	0.95456900
H	2.27425200	-0.55192000	1.41474600
H	3.05062100	0.45946900	2.65137000
C	5.68961100	0.19461400	1.72202200
H	5.64566800	0.58414600	2.74965500
H	6.62193700	-0.37324800	1.60585100
H	5.73697900	1.05243700	1.03624200
H	3.63448500	-1.89953300	0.07326600
Br	8.17675100	-0.30555100	-1.43019800

E

C	0.33797900	-4.83246400	-1.91128000
C	1.70107800	-4.73398900	-1.48269200
C	1.97703300	-4.10176100	-0.22838800
C	-0.40887600	-3.68231500	-0.07575300
N	-0.70369600	-4.30834600	-1.19336100
C	0.90177100	-3.56348500	0.47153500
C	1.04390700	-2.72811900	1.71184100
C	1.88619500	-3.20407500	2.84393800

O	0.44926600	-1.65738400	1.77057400
C	2.01705200	-4.56929400	3.13409700
C	2.52706300	-2.25545300	3.65538600
C	2.78250400	-4.98317300	4.22444700
H	1.51100300	-5.30573100	2.50905200
C	3.31528000	-2.67109300	4.72684500
H	2.41406900	-1.19773900	3.41520300
C	3.44118800	-4.03568600	5.01451400
H	2.87341700	-6.04677900	4.45270200
H	3.83334500	-1.93251200	5.34154800
H	4.05523900	-4.36074300	5.85695100
C	3.37864300	-3.98280900	0.27015000
C	3.98427400	-2.68173300	0.33289500
C	4.06671000	-5.10540800	0.71687500
C	5.28998800	-2.54601000	0.90733300
C	5.37070000	-4.94535900	1.26270000
C	5.96161900	-3.71036800	1.36757000
H	5.89423500	-5.83244600	1.62651700
H	6.95517800	-3.60644100	1.80958000
C	3.48020900	-6.49429400	0.69541600
H	2.40259400	-6.49707500	0.49243900
H	3.96399900	-7.11083500	-0.08075200
H	3.65195200	-6.99508100	1.66106900

H	-1.23958700	-3.23534000	0.47871900
C	0.04145300	-5.47994600	-3.13958700
C	2.72263600	-5.25387000	-2.32463900
H	3.76277200	-5.15393300	-2.01485100
H	-1.00677900	-5.54516300	-3.43462000
C	2.40503000	-5.87008200	-3.51684500
C	1.05297200	-5.99173700	-3.92490600
H	0.81612700	-6.48483600	-4.86967800
H	3.19900800	-6.26420500	-4.15378600
C	3.33786100	-1.50826700	-0.14850400
C	5.87940700	-1.25724900	1.00881000
H	6.87224800	-1.17201000	1.45668900
H	2.35430100	-1.58904800	-0.60934100
C	5.21650400	-0.13760800	0.55267300
C	3.93616800	-0.26971900	-0.03764100
H	5.67677200	0.84865700	0.63818900
H	3.41797100	0.61569700	-0.41093200

TS1*S*

C	-1.93797000	-0.70563400	-2.28113800
C	-1.92820200	-0.24004900	-0.95789300
C	0.54772300	0.27135300	-0.70528800

H	-1.77654700	-0.78197100	-0.03058400
C	-2.29695300	0.40976900	-3.10540200
N	-2.30930900	1.08552500	-0.93982600
C	-2.48473400	1.52766700	-2.23274800
H	-2.06907000	1.68535100	-0.14636000
C	-1.76435300	-2.09654200	-2.72681000
C	-0.76762100	-2.47822500	-3.68546400
C	-2.62717500	-3.07824900	-2.27127700
C	0.14283600	-1.53754100	-4.23936600
C	-0.67267500	-3.84987800	-4.09716000
C	-2.57759400	-4.41684700	-2.70115500
C	1.09338700	-1.92814700	-5.15768100
H	0.09080400	-0.49815200	-3.92165300
C	0.33341600	-4.22255900	-5.03086800
C	-1.59437700	-4.80228000	-3.58207700
H	-3.32266700	-5.11353500	-2.32623500
C	1.19545400	-3.28325900	-5.55597600
H	1.78818900	-1.19041800	-5.56225800
H	0.40306500	-5.27053900	-5.33118200
H	-1.52756700	-5.84200500	-3.90757000
H	1.96072000	-3.58153400	-6.27523800
O	-3.62810300	-2.74806100	-1.33528500
S	-4.90304300	-1.81510700	-1.89689500

O	-4.97900000	-0.58520000	-1.10471700
O	-4.94019500	-1.83370300	-3.36634800
C	-6.26824600	-2.96843000	-1.28471400
F	-7.43730600	-2.46741300	-1.68403800
F	-6.24528600	-3.04165800	0.04242200
F	-6.09422200	-4.18183800	-1.80730400
C	-2.77719500	2.80828600	-2.72732600
C	-2.46564500	0.57877800	-4.49696700
C	-2.91223100	2.94567900	-4.10189700
C	-2.76873200	1.84161000	-4.98049400
Rh	0.58453000	0.77447100	1.24633600
Rh	0.94218800	1.38424400	3.59553800
O	-0.52110500	0.01266500	4.05939500
O	2.43968600	2.69727800	2.96915400
O	-0.47149400	2.86728500	3.31841100
C	-1.06436400	-0.66221400	3.14655200
C	2.62420600	2.80760000	1.72972700
C	-1.11319700	2.92993700	2.24310000
O	-0.89185100	-0.50410900	1.89145900
C	-2.08907100	-1.72947600	3.52599800
O	2.06578700	2.11269600	0.81634200
C	3.62812900	3.80614600	1.14637100
O	-0.86456900	2.26180700	1.17430000

C	-2.26055700	3.93030700	2.09650500
N	-1.93941100	-2.88183300	2.61119000
H	-3.05134400	-1.28847800	3.23797600
C	-2.20575800	-2.10493600	5.03851500
N	2.99967000	4.35779300	-0.07953700
H	4.42992200	3.17170200	0.74726300
C	4.33034200	4.82262200	2.09009600
N	-3.43389200	3.23444000	1.51640600
H	-1.93214300	4.59959300	1.29298100
C	-2.59950900	4.83735400	3.31736400
C	-3.10984700	-3.38048300	2.01571800
C	-0.64864000	-3.35258500	2.32196300
C	-3.24790600	-3.22825200	5.19265500
C	-0.88047700	-2.57707400	5.65729800
C	-2.72604500	-0.86935400	5.80173300
C	3.59962600	4.00498900	-1.29946500
C	1.68506700	4.84193400	0.03012000
C	3.38662200	5.63796400	2.98782700
C	5.29877700	4.00434100	2.96820600
C	5.15630700	5.78407800	1.21751000
C	-4.01636100	3.80274000	0.37230100
C	-3.84104100	2.00907100	2.07777500
C	-3.73052200	5.79799900	2.90334500

C	-3.04584300	4.05886200	4.56506200
C	-1.35171700	5.68258200	3.64424200
O	-4.19251500	-2.83597500	2.19558000
C	-2.97030300	-4.61093400	1.20442800
O	0.32348700	-2.88497000	2.90072500
C	-0.52646400	-4.42496000	1.31159000
H	-3.37208000	-3.46355800	6.26110400
H	-2.93003100	-4.15182000	4.68470600
H	-4.22581700	-2.93394900	4.78575900
H	-1.02779500	-2.75079000	6.73612100
H	-0.09113800	-1.82868300	5.52742500
H	-0.53538200	-3.51800900	5.20692800
H	-1.99264100	-0.05442000	5.79654400
H	-2.94028200	-1.14843400	6.84587100
H	-3.66028900	-0.49164200	5.35497000
O	4.70436500	3.47099500	-1.32694100
C	2.83175700	4.27314900	-2.53581400
O	1.16343000	4.96238200	1.13068400
C	0.95905100	5.15506300	-1.21779400
H	2.72883300	6.28793500	2.39682700
H	3.99088100	6.27442200	3.65546900
H	2.75515400	4.98462800	3.60218700
H	4.76537900	3.23392200	3.53865500

H	5.81791400	4.66824200	3.67697500
H	6.06303300	3.50328500	2.35162800
H	4.50646100	6.42411000	0.60141800
H	5.83180200	5.23517600	0.54296400
H	5.76641700	6.43852600	1.85941300
O	-3.52689100	4.80127300	-0.15118100
C	-5.21950500	3.14065000	-0.16945200
O	-3.18060300	1.49719800	2.97188100
C	-5.07116700	1.39036300	1.54381800
H	-3.94589400	6.48841300	3.73341100
H	-4.66133900	5.25686000	2.67360200
H	-3.45529500	6.39266300	2.01981200
H	-2.30213600	3.30930500	4.85949800
H	-4.00479300	3.54631500	4.39996900
H	-3.18748100	4.76698800	5.39794400
H	-1.61812500	6.45651300	4.38130000
H	-0.96424900	6.18664800	2.74436500
H	-0.54539400	5.06616300	4.05871600
C	-4.10524900	-5.27846100	0.77887500
C	-1.68742000	-5.07953400	0.82850500
C	0.72980300	-4.81201500	0.87838700
C	3.36637600	3.90010600	-3.75777200
C	1.53099000	4.83380600	-2.47401600

C	-0.32326200	5.67223500	-1.14039600
C	-5.83859600	3.67409500	-1.28901900
C	-5.73780000	1.96910200	0.43536900
C	-5.55079000	0.22681900	2.12281500
H	-5.08251600	-4.90059400	1.07132800
C	-3.99901800	-6.42050700	-0.03745600
C	-1.55126300	-6.20280400	-0.04985400
C	0.86179700	-5.88076900	-0.03192200
H	1.61508600	-4.29049900	1.23405100
H	4.36208800	3.45902100	-3.78732800
C	2.62446500	4.06000600	-4.94405800
C	0.75779800	5.01740400	-3.66573400
C	-1.07218600	5.88936900	-2.31332300
H	-0.74687700	5.89255900	-0.16245600
H	-5.42731600	4.57375600	-1.74474600
C	-6.97842900	3.05968700	-1.83791100
C	-6.91153400	1.33796500	-0.08690500
C	-6.72010900	-0.38100500	1.62714500
H	-5.01040100	-0.21219100	2.95859900
H	-4.89731100	-6.94017300	-0.36808200
C	-2.75285600	-6.85515500	-0.44993300
C	-0.24736600	-6.57520600	-0.47253900
H	1.85698500	-6.16288500	-0.37747900

H	3.04713900	3.74558700	-5.89728800
C	1.35244100	4.59947300	-4.89300300
C	-0.54779400	5.56394200	-3.54865200
H	-2.08436800	6.28599500	-2.23443700
H	-7.44718500	3.47577700	-2.72846000
C	-7.49665000	1.92181900	-1.24650300
C	-7.38876400	0.16160200	0.54882600
H	-7.08046000	-1.30104200	2.08625200
Br	-2.67906500	-8.34588400	-1.64989600
H	-0.13474100	-7.41499900	-1.15712000
Br	0.37103800	4.74339700	-6.52851600
H	-1.13969400	5.70901600	-4.45084500
Br	-9.02632800	1.10911800	-2.05889500
H	-8.27579100	-0.32936100	0.15117700
C	5.67288900	-2.99493600	-0.20136100
C	4.59954200	-3.04354200	0.81434300
C	5.90050900	-4.10409700	-0.99689600
C	6.45895300	-1.82897800	-0.36738900
O	3.87350000	-4.02289100	0.94420400
N	4.47048100	-1.92857800	1.65509300
H	5.28152900	-4.98970700	-0.85800000
C	6.90905300	-4.08603000	-1.97867100
C	7.48853500	-1.78047500	-1.36068000

C	6.19765500	-0.68279200	0.42351800
C	3.49709700	-2.02178500	2.76207100
C	5.12370000	-0.70774200	1.43939500
H	7.07680200	-4.96131800	-2.60479700
C	7.67728700	-2.95045300	-2.15295100
C	8.22298500	-0.57462200	-1.51471900
C	6.92733000	0.47905200	0.24232200
C	2.54426100	-0.83082500	2.68512100
H	2.85942900	-2.87153200	2.49668800
C	4.14408900	-2.36552300	4.13754600
O	4.81573500	0.28467200	2.08711800
Br	9.00059900	-2.96807300	-3.53574300
H	9.00749400	-0.52691000	-2.26900600
C	7.94596600	0.52887200	-0.73096800
H	6.69332400	1.34713300	0.85732800
O	2.02333800	-0.66790900	1.53315200
O	2.28844600	-0.16693900	3.72558400
C	5.03163100	-3.61148100	3.95839200
C	4.99633000	-1.23033000	4.72466200
C	3.00777800	-2.72554000	5.11369000
H	8.51569400	1.44862400	-0.86860600
H	5.88526600	-3.41052600	3.29250700
H	4.46446900	-4.45647700	3.54076900

H	5.43860100	-3.91529500	4.93539200
H	5.88252100	-1.02591100	4.10636900
H	5.34919100	-1.52341100	5.72708300
H	4.41581700	-0.30687800	4.81270900
H	2.37247700	-3.52651200	4.70565300
H	2.36727900	-1.85857800	5.31023900
H	3.43609800	-3.07290200	6.06723100
O	1.12245100	1.74730200	6.10169300
C	1.82316700	1.03687700	6.80753500
C	1.30052500	-0.05653600	7.69717600
O	3.15763600	1.12994000	6.86683300
H	0.20868300	0.00704500	7.76171400
H	1.75708700	0.00478700	8.69519700
H	1.57966300	-1.02832400	7.26155400
C	3.80115700	2.11804000	6.00898100
C	3.62158300	3.53426400	6.52376400
H	3.41634000	2.00453900	4.98820100
H	4.85640000	1.81675900	6.02626400
H	4.21931800	4.22036500	5.90404600
H	3.96458800	3.62105400	7.56683400
H	2.56998400	3.84558400	6.46160800
H	-2.35321800	-0.27163600	-5.17004000
H	-2.89332800	1.99625500	-6.05383000

H	-3.13425200	3.92989600	-4.51849000
H	-2.87404600	3.66155000	-2.05935500
C	1.19524200	-1.04401500	-1.08672900
Br	0.62190600	1.63798400	-2.01059500
C	2.56352800	-1.01594400	-1.68705600
C	3.41893000	0.09696400	-1.59747300
C	2.95192500	-2.12813900	-2.44997200
C	4.60204400	0.12803700	-2.33337600
H	3.16706000	0.93506600	-0.94969300
C	4.13639400	-2.09347100	-3.18386500
H	2.28640300	-2.98979900	-2.49398200
C	4.94953900	-0.95578200	-3.14699100
H	5.24848700	1.00318100	-2.26064300
H	4.41964700	-2.94898500	-3.79923700
H	5.86601900	-0.92237700	-3.73852600
O	0.59586500	-2.08751800	-0.86900000

TS1*R*

C	-2.28347600	-2.17916300	-0.88264200
C	-2.30153000	-0.88383800	-0.34919700
C	0.08339000	-0.11731700	-0.41047300
H	-2.21290800	-0.55753800	0.68250600
C	-2.59999700	-2.05754100	-2.27062500

N	-2.68418700	0.00603700	-1.34590600
C	-2.78927800	-0.66518400	-2.53843700
H	-2.47086300	1.00637700	-1.26754500
C	-2.03143300	-3.46992600	-0.20774100
C	-3.06899000	-4.10214500	0.55510800
C	-0.84385500	-4.16168300	-0.38817700
C	-4.33775500	-3.48707500	0.74823400
C	-2.85954400	-5.41660200	1.08682500
C	-0.58882700	-5.41578100	0.20921700
C	-5.35292500	-4.15751400	1.39760200
H	-4.50393900	-2.48219200	0.36328600
C	-3.92982700	-6.08222000	1.74296700
C	-1.59216100	-6.03134900	0.92148600
H	0.38021000	-5.89019600	0.08771400
C	-5.15648400	-5.47220900	1.88694400
H	-6.32082300	-3.67362000	1.53195700
H	-3.75617500	-7.08609900	2.13542100
H	-1.41614400	-7.01169000	1.36625100
H	-5.97459400	-5.99238600	2.38892000
O	0.12743000	-3.48327900	-1.16664100
S	1.33885400	-4.19547000	-2.04556300
O	2.11963800	-3.08440200	-2.58837200
O	1.95593100	-5.32605400	-1.33433600

C	0.34749100	-4.92990300	-3.48453600
F	-0.06903100	-3.95207000	-4.27586200
F	-0.69708500	-5.61664600	-3.01449300
F	1.15285800	-5.74854800	-4.15528300
C	-3.02912400	-0.19301800	-3.84095200
C	-2.74005400	-2.99424200	-3.31658000
C	-3.13629200	-1.13799000	-4.85074000
C	-3.00844100	-2.52784000	-4.59241300
Rh	-0.01671100	1.74571800	0.37012700
Rh	0.10032700	4.01407000	1.30523700
O	-1.24988800	3.33980500	2.74484800
O	1.48725500	4.48151700	-0.18037600
O	-1.47298900	4.55450000	0.06659500
C	-1.62482200	2.13694600	2.73564400
C	1.85210800	3.55429300	-0.95216900
C	-2.04158500	3.66738000	-0.61479100
O	-1.39230300	1.28293300	1.81553400
C	-2.50093100	1.59142100	3.86741300
O	1.40020600	2.36152600	-0.96601200
C	3.01546600	3.75233700	-1.92676400
O	-1.59989600	2.47583100	-0.79673100
C	-3.32450800	4.00148300	-1.38578300
N	-2.08539300	0.19541600	4.13264600

H	-3.48621800	1.47805500	3.40116500
C	-2.72637900	2.48980300	5.12342700
N	2.77313000	2.86481600	-3.08963900
H	3.84405300	3.25824900	-1.40274000
C	3.51995500	5.19106200	-2.23495900
N	-4.27782600	2.87842600	-1.23157400
H	-3.03472500	3.94191900	-2.44345700
C	-3.94362400	5.42013800	-1.18693100
C	-3.07961700	-0.79086300	4.08493700
C	-0.71282100	-0.07772100	4.25544400
C	-3.47234400	1.66921400	6.19123300
C	-1.43813700	3.05100200	5.74788300
C	-3.64385200	3.64589100	4.67931100
C	3.65660900	1.78488100	-3.24352300
C	1.52318700	2.94396400	-3.72253700
C	4.14197600	5.74525600	-0.93651200
C	4.62902000	5.08084200	-3.29653700
C	2.43692700	6.16051000	-2.73143700
C	-4.79719000	2.31863500	-2.41166100
C	-4.51876700	2.36878800	0.05478000
C	-5.25586100	5.50080300	-1.98865600
C	-4.23878300	5.78019900	0.27814900
C	-2.95451700	6.43956300	-1.78460400

O	-4.25937800	-0.49096600	3.91992600
C	-2.64647100	-2.19383500	4.25177600
O	0.08933700	0.84600400	4.30762100
C	-0.29695900	-1.49323600	4.29566100
H	-3.72121000	2.31951400	7.04441500
H	-2.85215700	0.84243500	6.57045500
H	-4.40519400	1.24280500	5.79535100
H	-0.78557200	2.24727400	6.11273900
H	-1.70646000	3.69205000	6.60401100
H	-0.86665600	3.64919700	5.02839300
H	-3.81794000	4.33372800	5.52165200
H	-4.62230600	3.26384500	4.34578500
H	-3.20244400	4.21142800	3.85051300
O	4.70775600	1.73616100	-2.61171500
C	3.25091400	0.69494000	-4.15668500
O	0.74521500	3.84594600	-3.44178000
C	1.17407500	1.88333000	-4.69506400
H	4.91917900	5.06793800	-0.54803700
H	3.38301800	5.86905000	-0.15392800
H	4.60759100	6.72311500	-1.13741700
H	5.07465400	6.07210600	-3.47377300
H	4.23391500	4.71120100	-4.25512400
H	5.42692800	4.39552800	-2.97167300

H	2.05081300	5.87091000	-3.71709700
H	2.86794700	7.17225300	-2.81146600
H	1.58930800	6.19876700	-2.03529700
O	-4.48711200	2.77495200	-3.50808400
C	-5.68287600	1.14665700	-2.27157400
O	-3.97457900	2.87819000	1.02591600
C	-5.40831100	1.19386300	0.16704600
H	-5.66347500	6.52097900	-1.91622400
H	-6.01615900	4.81039400	-1.59192400
H	-5.09995100	5.26376900	-3.05098800
H	-4.60332700	6.81950600	0.32228100
H	-3.33979000	5.70064800	0.89775200
H	-5.01436700	5.13177800	0.70801900
H	-1.99993900	6.43349500	-1.24682400
H	-3.38187600	7.45257900	-1.72111900
H	-2.75666400	6.22248500	-2.84721800
C	-3.60218100	-3.19307300	4.32777000
C	-1.26978300	-2.52232600	4.30598700
C	1.05106800	-1.80303600	4.30030800
C	4.05339100	-0.42799800	-4.26636900
C	2.02060200	0.75719400	-4.85666400
C	-0.04213900	1.94729600	-5.35409400
C	-6.19783700	0.54983800	-3.41076500

C	-5.95537400	0.59563300	-0.99638400
C	-5.66067800	0.65433500	1.41673600
H	-4.65631800	-2.92511200	4.27943500
C	-3.21738100	-4.53901500	4.46544200
C	-0.85026400	-3.89004400	4.37239700
C	1.46770000	-3.14881000	4.31767800
H	1.79428100	-1.01181500	4.27524900
H	4.99381900	-0.46300700	-3.71892300
C	3.65161200	-1.52270200	-5.05397400
C	1.59178300	-0.33800500	-5.67563900
C	-0.45157000	0.88664500	-6.18639000
H	-0.68190000	2.81400400	-5.19091600
H	-5.97133900	0.98385500	-4.38344400
C	-6.98115700	-0.61510400	-3.31796700
C	-6.76056900	-0.58100000	-0.87141500
C	-6.46442800	-0.49573200	1.54546700
H	-5.22439400	1.11329500	2.29911300
H	-3.97418600	-5.31458000	4.55011500
C	-1.87712300	-4.87267800	4.46485600
C	0.54194700	-4.17234500	4.35981300
H	2.53499400	-3.37096500	4.29857100
H	4.27794300	-2.41134500	-5.11059000
C	2.45241300	-1.47462300	-5.73731500

C	0.33898500	-0.23653100	-6.33613600
H	-1.41281500	0.94332100	-6.69733800
H	-7.36534300	-1.08776700	-4.22066100
C	-7.24719300	-1.16513600	-2.07791600
C	-7.00792700	-1.09748400	0.42811700
H	-6.63387700	-0.91455100	2.53671600
Br	-1.42158700	-6.72833900	4.60197100
H	0.86882100	-5.21119300	4.38824000
Br	1.95458100	-3.00218100	-6.77415100
H	-0.00076400	-1.06611200	-6.95470800
Br	-8.28776700	-2.76836300	-2.01103300
H	-7.62896000	-1.98642300	0.52797600
C	5.81507000	-0.81317100	2.43848400
C	4.60742500	-0.23516800	3.06313400
C	6.29362400	-2.02921300	2.89441400
C	6.46936000	-0.14463100	1.37661000
O	4.00849800	-0.81155000	3.96427500
N	4.19423300	1.02614900	2.60840700
H	5.77240500	-2.52998300	3.70936500
C	7.42999400	-2.61687400	2.30805800
C	7.62075300	-0.72367100	0.75378600
C	5.94707700	1.07707800	0.88210100
C	3.12051600	1.70372700	3.36493200

C	4.72534800	1.65993800	1.47734900
H	7.79794600	-3.57570000	2.67064500
C	8.07051600	-1.97721000	1.26313400
C	8.21608300	-0.03705400	-0.33848000
C	6.55198000	1.71908600	-0.18404400
C	2.01655600	2.17005000	2.41557000
H	2.64260500	0.90753400	3.94352800
C	3.66418600	2.73892500	4.40155300
O	4.19245700	2.66554300	1.02405600
Br	9.58852600	-2.84142600	0.48079500
H	9.09098800	-0.47140300	-0.82085600
C	7.69305300	1.15758900	-0.79251400
H	6.12082000	2.64440900	-0.55755600
O	1.54370900	1.26663900	1.64743600
O	1.60961400	3.35750300	2.50988700
C	4.72621300	2.04191700	5.27420300
C	4.29932700	3.98473300	3.76203300
C	2.49979100	3.15318900	5.32361700
H	8.15649300	1.66738100	-1.63775700
H	5.61012200	1.74680200	4.68783700
H	4.32431200	1.14221500	5.76282700
H	5.06785900	2.73763500	6.05613100
H	5.21305800	3.73167100	3.20437400

H	4.58401400	4.69126000	4.55906500
H	3.60424300	4.48296100	3.07658900
H	1.73930600	3.72638500	4.78122100
H	2.88961800	3.77447300	6.14568500
H	2.01022300	2.26995100	5.76272500
O	0.43679500	6.41193400	2.25577700
C	-0.32593800	7.14405600	1.63936800
C	0.00418600	7.77759100	0.31816700
O	-1.55266400	7.44805000	2.07879100
H	0.21024200	6.97096900	-0.40043900
H	0.92686500	8.36670900	0.42415100
H	-0.81079400	8.41124200	-0.04938600
C	-1.93959600	6.83216000	3.34017100
C	-3.33845300	7.30282900	3.66946400
H	-1.20989700	7.13158800	4.10759900
H	-1.87838200	5.74146600	3.22162600
H	-3.64593500	6.88086000	4.63837900
H	-4.05838700	6.97143700	2.90688100
H	-3.37958100	8.40078800	3.74059300
H	-2.63100100	-4.05974600	-3.11575600
H	-3.11178200	-3.23420900	-5.41816300
H	-3.32747700	-0.80420600	-5.87250700
H	-3.13296500	0.87121200	-4.04769800

C	0.67317000	-1.19847000	0.46585300
Br	0.32637500	-0.31747900	-2.26074200
C	2.11677100	-1.53503000	0.29156200
C	3.01280400	-0.73214600	-0.43204100
C	2.55023200	-2.76395200	0.81361000
C	4.29852400	-1.19644000	-0.70079200
H	2.71112900	0.25387100	-0.78084000
C	3.83738000	-3.22361900	0.54157600
H	1.84643900	-3.36031400	1.39413900
C	4.70509900	-2.45032400	-0.23531300
H	4.98051700	-0.56609500	-1.27081700
H	4.15971800	-4.19453100	0.92149700
H	5.70891000	-2.81596900	-0.45896200
O	-0.02292800	-1.74219600	1.31253100

TS1$_{S\text{-H}}$

C	-1.93797000	-0.70563400	-2.28113800
C	-1.92820200	-0.24004900	-0.95789300
C	0.54772300	0.27135300	-0.70528800
H	-1.77654700	-0.78197100	-0.03058400
C	-2.29695300	0.40976900	-3.10540200
N	-2.30930900	1.08552500	-0.93982600
C	-2.48473400	1.52766700	-2.23274800

H	-2.06907000	1.68535100	-0.14636000
C	-1.76435300	-2.09654200	-2.72681000
C	-0.76762100	-2.47822500	-3.68546400
C	-2.62717500	-3.07824900	-2.27127700
C	0.14283600	-1.53754100	-4.23936600
C	-0.67267500	-3.84987800	-4.09716000
C	-2.57759400	-4.41684700	-2.70115500
C	1.09338700	-1.92814700	-5.15768100
H	0.09080400	-0.49815200	-3.92165300
C	0.33341600	-4.22255900	-5.03086800
C	-1.59437700	-4.80228000	-3.58207700
H	-3.32266700	-5.11353500	-2.32623500
C	1.19545400	-3.28325900	-5.55597600
H	1.78818900	-1.19041800	-5.56225800
H	0.40306500	-5.27053900	-5.33118200
H	-1.52756700	-5.84200500	-3.90757000
H	1.96072000	-3.58153400	-6.27523800
C	-2.77719500	2.80828600	-2.72732600
C	-2.46564500	0.57877800	-4.49696700
C	-2.91223100	2.94567900	-4.10189700
C	-2.76873200	1.84161000	-4.98049400
Rh	0.58453000	0.77447100	1.24633600
Rh	0.94218800	1.38424400	3.59553800

O	-0.52110500	0.01266500	4.05939500
O	2.43968600	2.69727800	2.96915400
O	-0.47149400	2.86728500	3.31841100
C	-1.06436400	-0.66221400	3.14655200
C	2.62420600	2.80760000	1.72972700
C	-1.11319700	2.92993700	2.24310000
O	-0.89185100	-0.50410900	1.89145900
C	-2.08907100	-1.72947600	3.52599800
O	2.06578700	2.11269600	0.81634200
C	3.62812900	3.80614600	1.14637100
O	-0.86456900	2.26180700	1.17430000
C	-2.26055700	3.93030700	2.09650500
N	-1.93941100	-2.88183300	2.61119000
H	-3.05134400	-1.28847800	3.23797600
C	-2.20575800	-2.10493600	5.03851500
N	2.99967000	4.35779300	-0.07953700
H	4.42992200	3.17170200	0.74726300
C	4.33034200	4.82262200	2.09009600
N	-3.43389200	3.23444000	1.51640600
H	-1.93214300	4.59959300	1.29298100
C	-2.59950900	4.83735400	3.31736400
C	-3.10984700	-3.38048300	2.01571800
C	-0.64864000	-3.35258500	2.32196300

C	-3.24790600	-3.22825200	5.19265500
C	-0.88047700	-2.57707400	5.65729800
C	-2.72604500	-0.86935400	5.80173300
C	3.59962600	4.00498900	-1.29946500
C	1.68506700	4.84193400	0.03012000
C	3.38662200	5.63796400	2.98782700
C	5.29877700	4.00434100	2.96820600
C	5.15630700	5.78407800	1.21751000
C	-4.01636100	3.80274000	0.37230100
C	-3.84104100	2.00907100	2.07777500
C	-3.73052200	5.79799900	2.90334500
C	-3.04584300	4.05886200	4.56506200
C	-1.35171700	5.68258200	3.64424200
O	-4.19251500	-2.83597500	2.19558000
C	-2.97030300	-4.61093400	1.20442800
O	0.32348700	-2.88497000	2.90072500
C	-0.52646400	-4.42496000	1.31159000
H	-3.37208000	-3.46355800	6.26110400
H	-2.93003100	-4.15182000	4.68470600
H	-4.22581700	-2.93394900	4.78575900
H	-1.02779500	-2.75079000	6.73612100
H	-0.09113800	-1.82868300	5.52742500
H	-0.53538200	-3.51800900	5.20692800

H	-1.99264100	-0.05442000	5.79654400
H	-2.94028200	-1.14843400	6.84587100
H	-3.66028900	-0.49164200	5.35497000
O	4.70436500	3.47099500	-1.32694100
C	2.83175700	4.27314900	-2.53581400
O	1.16343000	4.96238200	1.13068400
C	0.95905100	5.15506300	-1.21779400
H	2.72883300	6.28793500	2.39682700
H	3.99088100	6.27442200	3.65546900
H	2.75515400	4.98462800	3.60218700
H	4.76537900	3.23392200	3.53865500
H	5.81791400	4.66824200	3.67697500
H	6.06303300	3.50328500	2.35162800
H	4.50646100	6.42411000	0.60141800
H	5.83180200	5.23517600	0.54296400
H	5.76641700	6.43852600	1.85941300
O	-3.52689100	4.80127300	-0.15118100
C	-5.21950500	3.14065000	-0.16945200
O	-3.18060300	1.49719800	2.97188100
C	-5.07116700	1.39036300	1.54381800
H	-3.94589400	6.48841300	3.73341100
H	-4.66133900	5.25686000	2.67360200
H	-3.45529500	6.39266300	2.01981200

H	-2.30213600	3.30930500	4.85949800
H	-4.00479300	3.54631500	4.39996900
H	-3.18748100	4.76698800	5.39794400
H	-1.61812500	6.45651300	4.38130000
H	-0.96424900	6.18664800	2.74436500
H	-0.54539400	5.06616300	4.05871600
C	-4.10524900	-5.27846100	0.77887500
C	-1.68742000	-5.07953400	0.82850500
C	0.72980300	-4.81201500	0.87838700
C	3.36637600	3.90010600	-3.75777200
C	1.53099000	4.83380600	-2.47401600
C	-0.32326200	5.67223500	-1.14039600
C	-5.83859600	3.67409500	-1.28901900
C	-5.73780000	1.96910200	0.43536900
C	-5.55079000	0.22681900	2.12281500
H	-5.08251600	-4.90059400	1.07132800
C	-3.99901800	-6.42050700	-0.03745600
C	-1.55126300	-6.20280400	-0.04985400
C	0.86179700	-5.88076900	-0.03192200
H	1.61508600	-4.29049900	1.23405100
H	4.36208800	3.45902100	-3.78732800
C	2.62446500	4.06000600	-4.94405800
C	0.75779800	5.01740400	-3.66573400

C	-1.07218600	5.88936900	-2.31332300
H	-0.74687700	5.89255900	-0.16245600
H	-5.42731600	4.57375600	-1.74474600
C	-6.97842900	3.05968700	-1.83791100
C	-6.91153400	1.33796500	-0.08690500
C	-6.72010900	-0.38100500	1.62714500
H	-5.01040100	-0.21219100	2.95859900
H	-4.89731100	-6.94017300	-0.36808200
C	-2.75285600	-6.85515500	-0.44993300
C	-0.24736600	-6.57520600	-0.47253900
H	1.85698500	-6.16288500	-0.37747900
H	3.04713900	3.74558700	-5.89728800
C	1.35244100	4.59947300	-4.89300300
C	-0.54779400	5.56394200	-3.54865200
H	-2.08436800	6.28599500	-2.23443700
H	-7.44718500	3.47577700	-2.72846000
C	-7.49665000	1.92181900	-1.24650300
C	-7.38876400	0.16160200	0.54882600
H	-7.08046000	-1.30104200	2.08625200
Br	-2.67906500	-8.34588400	-1.64989600
H	-0.13474100	-7.41499900	-1.15712000
Br	0.37103800	4.74339700	-6.52851600
H	-1.13969400	5.70901600	-4.45084500

Br	-9.02632800	1.10911800	-2.05889500
H	-8.27579100	-0.32936100	0.15117700
C	5.67288900	-2.99493600	-0.20136100
C	4.59954200	-3.04354200	0.81434300
C	5.90050900	-4.10409700	-0.99689600
C	6.45895300	-1.82897800	-0.36738900
O	3.87350000	-4.02289100	0.94420400
N	4.47048100	-1.92857800	1.65509300
H	5.28152900	-4.98970700	-0.85800000
C	6.90905300	-4.08603000	-1.97867100
C	7.48853500	-1.78047500	-1.36068000
C	6.19765500	-0.68279200	0.42351800
C	3.49709700	-2.02178500	2.76207100
C	5.12370000	-0.70774200	1.43939500
H	7.07680200	-4.96131800	-2.60479700
C	7.67728700	-2.95045300	-2.15295100
C	8.22298500	-0.57462200	-1.51471900
C	6.92733000	0.47905200	0.24232200
C	2.54426100	-0.83082500	2.68512100
H	2.85942900	-2.87153200	2.49668800
C	4.14408900	-2.36552300	4.13754600
O	4.81573500	0.28467200	2.08711800
Br	9.00059900	-2.96807300	-3.53574300

H	9.00749400	-0.52691000	-2.26900600
C	7.94596600	0.52887200	-0.73096800
H	6.69332400	1.34713300	0.85732800
O	2.02333800	-0.66790900	1.53315200
O	2.28844600	-0.16693900	3.72558400
C	5.03163100	-3.61148100	3.95839200
C	4.99633000	-1.23033000	4.72466200
C	3.00777800	-2.72554000	5.11369000
H	8.51569400	1.44862400	-0.86860600
H	5.88526600	-3.41052600	3.29250700
H	4.46446900	-4.45647700	3.54076900
H	5.43860100	-3.91529500	4.93539200
H	5.88252100	-1.02591100	4.10636900
H	5.34919100	-1.52341100	5.72708300
H	4.41581700	-0.30687800	4.81270900
H	2.37247700	-3.52651200	4.70565300
H	2.36727900	-1.85857800	5.31023900
H	3.43609800	-3.07290200	6.06723100
O	1.12245100	1.74730200	6.10169300
C	1.82316700	1.03687700	6.80753500
C	1.30052500	-0.05653600	7.69717600
O	3.15763600	1.12994000	6.86683300
H	0.20868300	0.00704500	7.76171400

H	1.75708700	0.00478700	8.69519700
H	1.57966300	-1.02832400	7.26155400
C	3.80115700	2.11804000	6.00898100
C	3.62158300	3.53426400	6.52376400
H	3.41634000	2.00453900	4.98820100
H	4.85640000	1.81675900	6.02626400
H	4.21931800	4.22036500	5.90404600
H	3.96458800	3.62105400	7.56683400
H	2.56998400	3.84558400	6.46160800
H	-2.35321800	-0.27163600	-5.17004000
H	-2.89332800	1.99625500	-6.05383000
H	-3.13425200	3.92989600	-4.51849000
H	-2.87404600	3.66155000	-2.05935500
C	1.19524200	-1.04401500	-1.08672900
Br	0.62190600	1.63798400	-2.01059500
C	2.56352800	-1.01594400	-1.68705600
C	3.41893000	0.09696400	-1.59747300
C	2.95192500	-2.12813900	-2.44997200
C	4.60204400	0.12803700	-2.33337600
H	3.16706000	0.93506600	-0.94969300
C	4.13639400	-2.09347100	-3.18386500
H	2.28640300	-2.98979900	-2.49398200
C	4.94953900	-0.95578200	-3.14699100

H	5.24848700	1.00318100	-2.26064300
H	4.41964700	-2.94898500	-3.79923700
H	5.86601900	-0.92237700	-3.73852600
O	0.59586500	-2.08751800	-0.86900000
H	-3.37197300	-2.80431500	-1.55354700

TS1$_{R\text{-H}}$

C	-2.28347600	-2.17916300	-0.88264200
C	-2.30153000	-0.88383800	-0.34919700
C	0.08339000	-0.11731700	-0.41047300
H	-2.21290800	-0.55753800	0.68250600
C	-2.59999700	-2.05754100	-2.27062500
N	-2.68418700	0.00603700	-1.34590600
C	-2.78927800	-0.66518400	-2.53843700
H	-2.47086300	1.00637700	-1.26754500
C	-2.03143300	-3.46992600	-0.20774100
C	-3.06899000	-4.10214500	0.55510800
C	-0.84385500	-4.16168300	-0.38817700
C	-4.33775500	-3.48707500	0.74823400
C	-2.85954400	-5.41660200	1.08682500
C	-0.58882700	-5.41578100	0.20921700
C	-5.35292500	-4.15751400	1.39760200
H	-4.50393900	-2.48219200	0.36328600

C	-3.92982700	-6.08222000	1.74296700
C	-1.59216100	-6.03134900	0.92148600
H	0.38021000	-5.89019600	0.08771400
C	-5.15648400	-5.47220900	1.88694400
H	-6.32082300	-3.67362000	1.53195700
H	-3.75617500	-7.08609900	2.13542100
H	-1.41614400	-7.01169000	1.36625100
H	-5.97459400	-5.99238600	2.38892000
C	-3.02912400	-0.19301800	-3.84095200
C	-2.74005400	-2.99424200	-3.31658000
C	-3.13629200	-1.13799000	-4.85074000
C	-3.00844100	-2.52784000	-4.59241300
Rh	-0.01671100	1.74571800	0.37012700
Rh	0.10032700	4.01407000	1.30523700
O	-1.24988800	3.33980500	2.74484800
O	1.48725500	4.48151700	-0.18037600
O	-1.47298900	4.55450000	0.06659500
C	-1.62482200	2.13694600	2.73564400
C	1.85210800	3.55429300	-0.95216900
C	-2.04158500	3.66738000	-0.61479100
O	-1.39230300	1.28293300	1.81553400
C	-2.50093100	1.59142100	3.86741300
O	1.40020600	2.36152600	-0.96601200

C	3.01546600	3.75233700	-1.92676400
O	-1.59989600	2.47583100	-0.79673100
C	-3.32450800	4.00148300	-1.38578300
N	-2.08539300	0.19541600	4.13264600
H	-3.48621800	1.47805500	3.40116500
C	-2.72637900	2.48980300	5.12342700
N	2.77313000	2.86481600	-3.08963900
H	3.84405300	3.25824900	-1.40274000
C	3.51995500	5.19106200	-2.23495900
N	-4.27782600	2.87842600	-1.23157400
H	-3.03472500	3.94191900	-2.44345700
C	-3.94362400	5.42013800	-1.18693100
C	-3.07961700	-0.79086300	4.08493700
C	-0.71282100	-0.07772100	4.25544400
C	-3.47234400	1.66921400	6.19123300
C	-1.43813700	3.05100200	5.74788300
C	-3.64385200	3.64589100	4.67931100
C	3.65660900	1.78488100	-3.24352300
C	1.52318700	2.94396400	-3.72253700
C	4.14197600	5.74525600	-0.93651200
C	4.62902000	5.08084200	-3.29653700
C	2.43692700	6.16051000	-2.73143700
C	-4.79719000	2.31863500	-2.41166100

C	-4.51876700	2.36878800	0.05478000
C	-5.25586100	5.50080300	-1.98865600
C	-4.23878300	5.78019900	0.27814900
C	-2.95451700	6.43956300	-1.78460400
O	-4.25937800	-0.49096600	3.91992600
C	-2.64647100	-2.19383500	4.25177600
O	0.08933700	0.84600400	4.30762100
C	-0.29695900	-1.49323600	4.29566100
H	-3.72121000	2.31951400	7.04441500
H	-2.85215700	0.84243500	6.57045500
H	-4.40519400	1.24280500	5.79535100
H	-0.78557200	2.24727400	6.11273900
H	-1.70646000	3.69205000	6.60401100
H	-0.86665600	3.64919700	5.02839300
H	-3.81794000	4.33372800	5.52165200
H	-4.62230600	3.26384500	4.34578500
H	-3.20244400	4.21142800	3.85051300
O	4.70775600	1.73616100	-2.61171500
C	3.25091400	0.69494000	-4.15668500
O	0.74521500	3.84594600	-3.44178000
C	1.17407500	1.88333000	-4.69506400
H	4.91917900	5.06793800	-0.54803700
H	3.38301800	5.86905000	-0.15392800

H	4.60759100	6.72311500	-1.13741700
H	5.07465400	6.07210600	-3.47377300
H	4.23391500	4.71120100	-4.25512400
H	5.42692800	4.39552800	-2.97167300
H	2.05081300	5.87091000	-3.71709700
H	2.86794700	7.17225300	-2.81146600
H	1.58930800	6.19876700	-2.03529700
O	-4.48711200	2.77495200	-3.50808400
C	-5.68287600	1.14665700	-2.27157400
O	-3.97457900	2.87819000	1.02591600
C	-5.40831100	1.19386300	0.16704600
H	-5.66347500	6.52097900	-1.91622400
H	-6.01615900	4.81039400	-1.59192400
H	-5.09995100	5.26376900	-3.05098800
H	-4.60332700	6.81950600	0.32228100
H	-3.33979000	5.70064800	0.89775200
H	-5.01436700	5.13177800	0.70801900
H	-1.99993900	6.43349500	-1.24682400
H	-3.38187600	7.45257900	-1.72111900
H	-2.75666400	6.22248500	-2.84721800
C	-3.60218100	-3.19307300	4.32777000
C	-1.26978300	-2.52232600	4.30598700
C	1.05106800	-1.80303600	4.30030800

C	4.05339100	-0.42799800	-4.26636900
C	2.02060200	0.75719400	-4.85666400
C	-0.04213900	1.94729600	-5.35409400
C	-6.19783700	0.54983800	-3.41076500
C	-5.95537400	0.59563300	-0.99638400
C	-5.66067800	0.65433500	1.41673600
H	-4.65631800	-2.92511200	4.27943500
C	-3.21738100	-4.53901500	4.46544200
C	-0.85026400	-3.89004400	4.37239700
C	1.46770000	-3.14881000	4.31767800
H	1.79428100	-1.01181500	4.27524900
H	4.99381900	-0.46300700	-3.71892300
C	3.65161200	-1.52270200	-5.05397400
C	1.59178300	-0.33800500	-5.67563900
C	-0.45157000	0.88664500	-6.18639000
H	-0.68190000	2.81400400	-5.19091600
H	-5.97133900	0.98385500	-4.38344400
C	-6.98115700	-0.61510400	-3.31796700
C	-6.76056900	-0.58100000	-0.87141500
C	-6.46442800	-0.49573200	1.54546700
H	-5.22439400	1.11329500	2.29911300
H	-3.97418600	-5.31458000	4.55011500
C	-1.87712300	-4.87267800	4.46485600

C	0.54194700	-4.17234500	4.35981300
H	2.53499400	-3.37096500	4.29857100
H	4.27794300	-2.41134500	-5.11059000
C	2.45241300	-1.47462300	-5.73731500
C	0.33898500	-0.23653100	-6.33613600
H	-1.41281500	0.94332100	-6.69733800
H	-7.36534300	-1.08776700	-4.22066100
C	-7.24719300	-1.16513600	-2.07791600
C	-7.00792700	-1.09748400	0.42811700
H	-6.63387700	-0.91455100	2.53671600
Br	-1.42158700	-6.72833900	4.60197100
H	0.86882100	-5.21119300	4.38824000
Br	1.95458100	-3.00218100	-6.77415100
H	-0.00076400	-1.06611200	-6.95470800
Br	-8.28776700	-2.76836300	-2.01103300
H	-7.62896000	-1.98642300	0.52797600
C	5.81507000	-0.81317100	2.43848400
C	4.60742500	-0.23516800	3.06313400
C	6.29362400	-2.02921300	2.89441400
C	6.46936000	-0.14463100	1.37661000
O	4.00849800	-0.81155000	3.96427500
N	4.19423300	1.02614900	2.60840700
H	5.77240500	-2.52998300	3.70936500

C	7.42999400	-2.61687400	2.30805800
C	7.62075300	-0.72367100	0.75378600
C	5.94707700	1.07707800	0.88210100
C	3.12051600	1.70372700	3.36493200
C	4.72534800	1.65993800	1.47734900
H	7.79794600	-3.57570000	2.67064500
C	8.07051600	-1.97721000	1.26313400
C	8.21608300	-0.03705400	-0.33848000
C	6.55198000	1.71908600	-0.18404400
C	2.01655600	2.17005000	2.41557000
H	2.64260500	0.90753400	3.94352800
C	3.66418600	2.73892500	4.40155300
O	4.19245700	2.66554300	1.02405600
Br	9.58852600	-2.84142600	0.48079500
H	9.09098800	-0.47140300	-0.82085600
C	7.69305300	1.15758900	-0.79251400
H	6.12082000	2.64440900	-0.55755600
O	1.54370900	1.26663900	1.64743600
O	1.60961400	3.35750300	2.50988700
C	4.72621300	2.04191700	5.27420300
C	4.29932700	3.98473300	3.76203300
C	2.49979100	3.15318900	5.32361700
H	8.15649300	1.66738100	-1.63775700

H	5.61012200	1.74680200	4.68783700
H	4.32431200	1.14221500	5.76282700
H	5.06785900	2.73763500	6.05613100
H	5.21305800	3.73167100	3.20437400
H	4.58401400	4.69126000	4.55906500
H	3.60424300	4.48296100	3.07658900
H	1.73930600	3.72638500	4.78122100
H	2.88961800	3.77447300	6.14568500
H	2.01022300	2.26995100	5.76272500
O	0.43679500	6.41193400	2.25577700
C	-0.32593800	7.14405600	1.63936800
C	0.00418600	7.77759100	0.31816700
O	-1.55266400	7.44805000	2.07879100
H	0.21024200	6.97096900	-0.40043900
H	0.92686500	8.36670900	0.42415100
H	-0.81079400	8.41124200	-0.04938600
C	-1.93959600	6.83216000	3.34017100
C	-3.33845300	7.30282900	3.66946400
H	-1.20989700	7.13158800	4.10759900
H	-1.87838200	5.74146600	3.22162600
H	-3.64593500	6.88086000	4.63837900
H	-4.05838700	6.97143700	2.90688100
H	-3.37958100	8.40078800	3.74059300

H	-2.63100100	-4.05974600	-3.11575600
H	-3.11178200	-3.23420900	-5.41816300
H	-3.32747700	-0.80420600	-5.87250700
H	-3.13296500	0.87121200	-4.04769800
C	0.67317000	-1.19847000	0.46585300
Br	0.32637500	-0.31747900	-2.26074200
C	2.11677100	-1.53503000	0.29156200
C	3.01280400	-0.73214600	-0.43204100
C	2.55023200	-2.76395200	0.81361000
C	4.29852400	-1.19644000	-0.70079200
H	2.71112900	0.25387100	-0.78084000
C	3.83738000	-3.22361900	0.54157600
H	1.84643900	-3.36031400	1.39413900
C	4.70509900	-2.45032400	-0.23531300
H	4.98051700	-0.56609500	-1.27081700
H	4.15971800	-4.19453100	0.92149700
H	5.70891000	-2.81596900	-0.45896200
O	-0.02292800	-1.74219600	1.31253100
H	-0.08726300	-3.72651100	-1.00712200

7. References.

(1) Lebel, H.; Piras, H.; Bartholoméüs, J. Dirhodium (II) Tetrakis[N-4-Bromo-1,8-Naphthoyl-(S)-Tert-Leucinate]. *Org. Synth.* **2017**, *94*, 136–152.

(2) Jiang, F.; Chen, K.; Wu, P.; Zhang, Y.; Jiao, Y.; Shi, F. A Strategy for Synthesizing Axially Chiral Naphthyl-Indoles: Catalytic Asymmetric Addition Reactions of Racemic Substrates. *Angew Chem Int Ed* **2019**, *58*, 15104–15110.

(3) Poudel, T. N.; Lee, Y. R. Mild Base-Promoted Indole Annulation–Oxidative Cross- Coupling of 2-Nitrocinnamaldehydes with β-Tetralones for 3-Naphthylindole and 3-Naphthylbenzo[*g*]Indole Fluorophores. *Adv Synth Catal* **2017**, *359* (9), 1552–1562.

(4) Wang, Z.; Herraiz, A. G.; Del Hoyo, A. M.; Suero, M. G. Generating Carbyne Equivalents with Photoredox Catalysis. *Nature* **2018**, *554*, 86–91.

(5) Shu, W.-M.; Ma, J.-R.; Zheng, K.-L.; Sun, H.-Y.; Wang, M.; Yang, Y.; Wu, A.-X. One-Pot Synthesis of Polyfunctional Pyrazoles: An Easy Access to α-Diazoketones from Arylglyoxal Monohydrates and Tosylhydrazine. *Tetrahedron* **2014**, *70*, 9321–9329.

(6) Musio, B.; Mariani, F.; Śliwiński, E.; Kabeshov, M.; Odajima, H.; Ley, S. Combination of Enabling Technologies to Improve and Describe the Stereoselectivity of Wolff–Staudinger Cascade Reaction. *Synthesis* **2016**, *48*, 3515–3526.

(7) Zhang, J.; Chen, W.; Huang, D.; Zeng, X.; Wang, X.; Hu, Y. Tandem Synthesis of α-Diazoketones from 1,3-Diketones. *J. Org. Chem.* **2017**, *82*, 9171–9174.

(8) Mortén, M.; Hennum, M.; Bonge-Hansen, T. Synthesis of Quinoline-3-Carboxylates by a Rh(II)-Catalyzed Cyclopropanation-Ring Expansion Reaction of Indoles with Halodiazoacetates. *Beilstein J. Org. Chem.* **2015**, *11*, 1944–1949.

(9) Yamaguchi, M.; Suzuki, K.; Sato, Y.; Manabe, K. Palladium-Catalyzed Direct C3-Selective Arylation of N-Unsubstituted Indoles with Aryl Chlorides and Triflates. *Org. Lett.* **2017**, *19*, 5388–5391.

(10) Ye, A.; Li, Z.; Ding, T.; Ke, H.; Chen, Z. Phosphoric Acid Catalyzed Electrophilic Thiocyanation of Indoles: Access to SCN-Containing Aryl-Indole Compounds. *Chemistry An Asian Journal* **2022**, *17*, e202200256.

(11) Huang, R. Y.; Franke, P. T.; Nicolaus, N.; Lautens, M. Domino C–H Functionalization Reactions of Gem-Dibromoolefins: Synthesis of N-Fused Benzo[c]Carbazoles. *Tetrahedron* **2013**, *69*, 4395–4402.

(12) Xu, X.-H.; Taniguchi, M.; Azuma, A.; Liu, G. K.; Tokunaga, E.; Shibata, N. Remote Anionic Fries Rearrangement of Sulfonates: Regioselective Synthesis of Indole Triflones. *Org. Lett.* **2013**, *15*, 686–689.

(13) Lu, S.; Poh, S. B.; Siau, W.; Zhao, Y. Kinetic Resolution of Tertiary Alcohols: Highly Enantioselective Access to 3-Hydroxy-3-Substituted Oxindoles. *Angew Chem Int Ed* **2013**, *52*, 1731–1734.

(14) Li, Z.; Chen, Y.; Wang, C.; Xu, G.; Shao, Y.; Zhang, X.; Tang, S.; Sun, J. Construction of C−C Axial Chirality via Asymmetric Carbene Insertion into Arene C−H Bonds. *Angew Chem Int Ed* **2021**, *60*, 25714–25718.

(15) Lee, C.; Yang, W.; Parr, R. G. Development of the Colle-Salvetti Correlation-Energy Formula into a Functional of the Electron Density. *Phys. Rev. B* **1988**, *37*, 785–789.

(16) Becke, A. D. Density-Functional Thermochemistry. III. The Role of Exact Exchange. *The Journal of Chemical Physics* **1993**, *98*, 5648–5652.

(17) Stephens, P. J.; Devlin, F. J.; Chabalowski, C. F.; Frisch, M. J. Ab Initio Calculation of Vibrational Absorption and Circular Dichroism Spectra Using Density Functional Force Fields. *J. Phys. Chem.* **1994**, *98*, 11623–11627.

(18) Grimme, S.; Antony, J.; Ehrlich, S.; Krieg, H. A Consistent and Accurate *Ab Initio* Parametrization of Density Functional Dispersion Correction (DFT-D) for the 94 Elements H-Pu. *The Journal of Chemical Physics* **2010**, *132*, 154104.

(19) Grimme, S.; Ehrlich, S.; Goerigk, L. Effect of the Damping Function in Dispersion Corrected Density Functional Theory. *J Comput Chem* **2011**, *32*, 1456–1465.

(20) Gaussian 09, Revision A.02, Frisch, M. J.; Trucks, G. W.; Schlegel, H. B.; Scuseria, G. E.; Robb, M. A.; Cheeseman, J. R.; Scalmani, G.; Barone, V.; Mennucci, B.; Petersson, G. A.; Nakatsuji, H.; Caricato, M.; Li, X.; Hratchian, H. P.; Izmaylov, A. F.; Bloino, J.; Zheng, G.; Sonnenberg, J. L.; Hada, M.; Ehara, M.; Toyota, K.; Fukuda, R.; Hasegawa, J.; Ishida, M.; Nakajima, T.; Honda, Y.; Kitao, O.; Nakai, H.; Vreven, T.; Montgomery Jr., J. A.; Peralta, J. E.; Ogliaro, F.; Bearpark, M.; Heyd, J. J.; Brothers, E.; Kudin, K. N.; Staroverov, V. N.; Kobayashi, R.; Normand, K. Raghavachari, A. Rendell, J. C. Burant, S. S. Iyengar, J. Tomasi,

M. Cossi, N. Rega, J; Millam, J. M.; Klene, M.; Knox, J. E.; Cross, J. B.; Bakken, V.; Adamo, C.; Jaramillo, J; Gomperts, R.; Stratmann, R. E.; Yazyev, O.; Austin, A. J.; Cammi, R.; Pomelli, C.; Ochterski, J. W.; Martin, R. L.; Morokuma, K.; Zakrzewski, V. G.; Voth, G. A.; Salvador, P.; Dannenberg, J. J.; Dapprich, S.; Daniels, A. D.; Farkas, Ö.; Foresman, J. B.; Ortiz, J. V.; Cioslowski, J.; Fox, D. J. Gaussian, Inc., Wallingford CT, **2009** .

(21) Hay, P. J.; Wadt, W. R. *Ab Initio* Effective Core Potentials for Molecular Calculations. Potentials for the Transition Metal Atoms Sc to Hg. *The Journal of Chemical Physics* **1985**, *82*, 270–283.

(22) Ehlers, A. W.; Böhme, M.; Dapprich, S.; Gobbi, A.; Höllwarth, A.; Jonas, V.; Köhler, K. F.; Stegmann, R.; Veldkamp, A.; Frenking, G. A Set of F-Polarization Functions for Pseudo-Potential Basis Sets of the Transition Metals Sc□Cu, Y□Ag and La□Au. *Chemical Physics Letters* **1993**, *208*, 111–114.

(23) Dunning, T. H. Gaussian Basis Sets for Use in Correlated Molecular Calculations. I. The Atoms Boron through Neon and Hydrogen. *The Journal of Chemical Physics* **1989**, *90*, 1007–1023.

(24) Kendall, R. A.; Dunning, T. H.; Harrison, R. J. Electron Affinities of the First-Row Atoms Revisited. Systematic Basis Sets and Wave Functions. *The Journal of Chemical Physics* **1992**, *96*, 6796–6806.

(25) Cancès, E.; Mennucci, B.; Tomasi, J. A New Integral Equation Formalism for the Polarizable Continuum Model: Theoretical Background and Applications to Isotropic and Anisotropic Dielectrics. *The Journal of Chemical Physics* **1997**, *107*, 3032–3041.

(26) Pracht, P.; Bohle, F.; Grimme, S. Automated Exploration of the Low-Energy Chemical Space with Fast Quantum Chemical Methods. *Phys. Chem. Chem. Phys.* **2020**, *22*, 7169–7192.

(27) Álvarez-Moreno, M.; De Graaf, C.; López, N.; Maseras, F.; Poblet, J. M.; Bo, C. Managing the Computational Chemistry Big Data Problem: The **ioChem-BD** Platform. *J. Chem. Inf. Model.* **2015**, *55*, 95–103.

(28) Bonge, H. T.; Hansen, T. Halodiazoacetates as Useful Tools for Selective Halo-Functionalization. *Pure and Applied Chemistry* **2011**, *83*, 565–575.

(29) Diego Pizarro, J.; Morán-González, L.; González-Fernández, I.; Maseras, F.; Fructos, M. R.; Pérez, P. J. Selective C−H Bond Functionalization of Unprotected Indoles by Donor-Acceptor Carbene Insertion. *Adv Synth Catal* **2024**, *366*, 844–851.

(30) Liu, S.; Yang, Y.; Song, Q.; Liu, Z.; Lu, Y.; Wang, Z.; Sivaguru, P.; Bi, X. Tunable Molecular Editing of Indoles with Fluoroalkyl Carbenes. *Nat. Chem.* **2024**, *16*, 988–997.

(31) Zhang, X.; Song, Q.; Liu, S.; Sivaguru, P.; Liu, Z.; Yang, Y.; Ning, Y.; Anderson, E. A.; De Ruiter, G.; Bi, X. Asymmetric Dearomative Single-Atom Skeletal Editing of Indoles and Pyrroles. *Nat. Chem.* **2025**, *17*, 215–225.

8. Copies of NMR spectra.

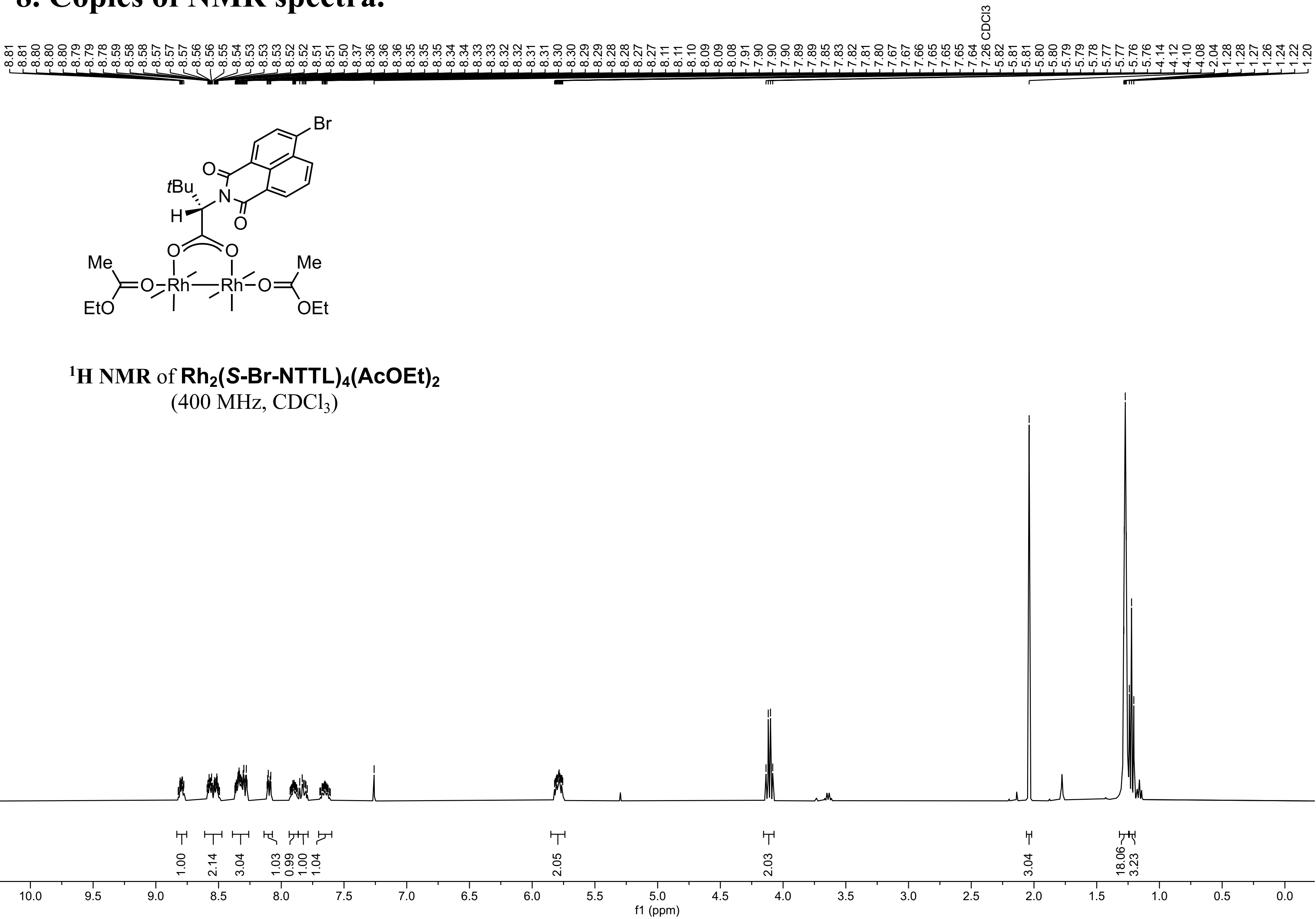

^{1}H NMR of **Rh_2(*S*-Br-NTTL)$_4$(AcOEt)$_2$**
(400 MHz, $CDCl_3$)

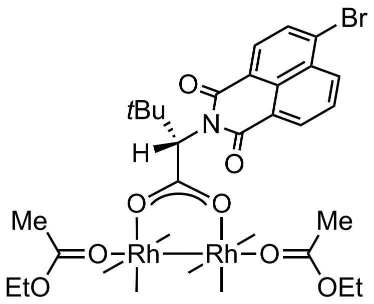

^{13}C NMR of **Rh_2(*S*-Br-NTTL)$_4$(AcOEt)$_2$**
(101 MHz, $CDCl_3$)

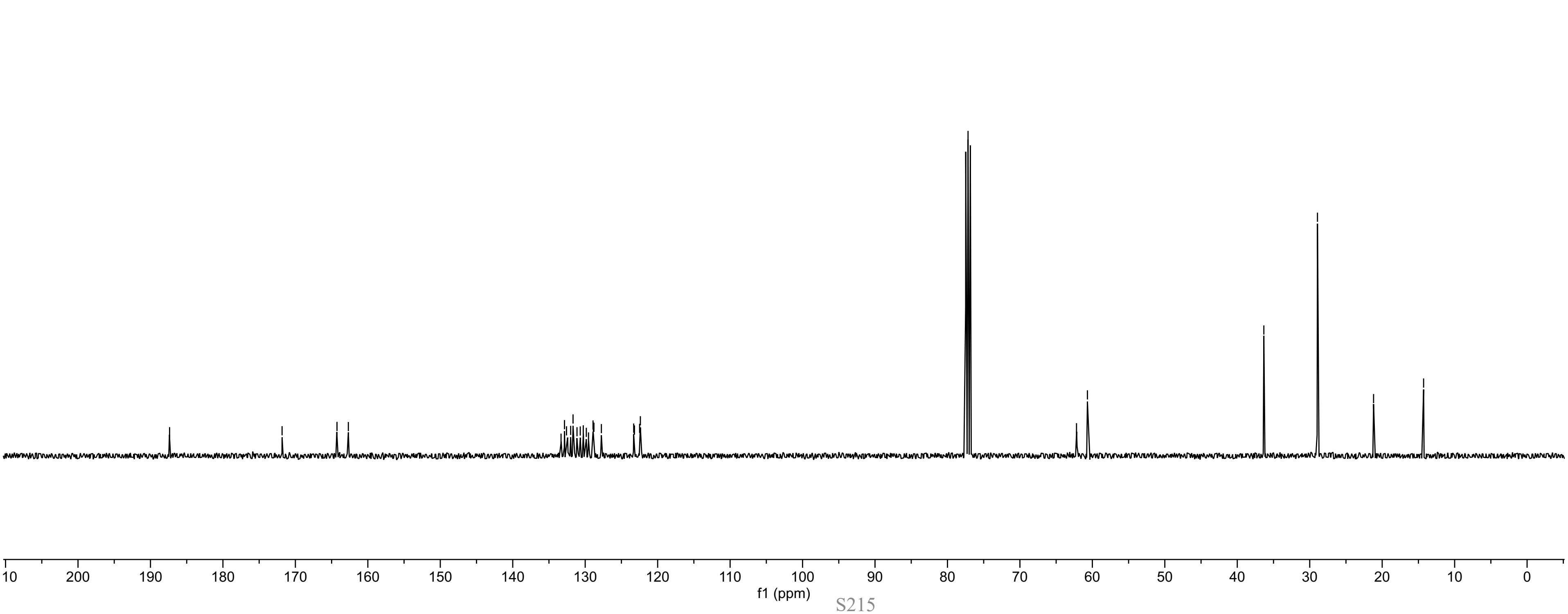

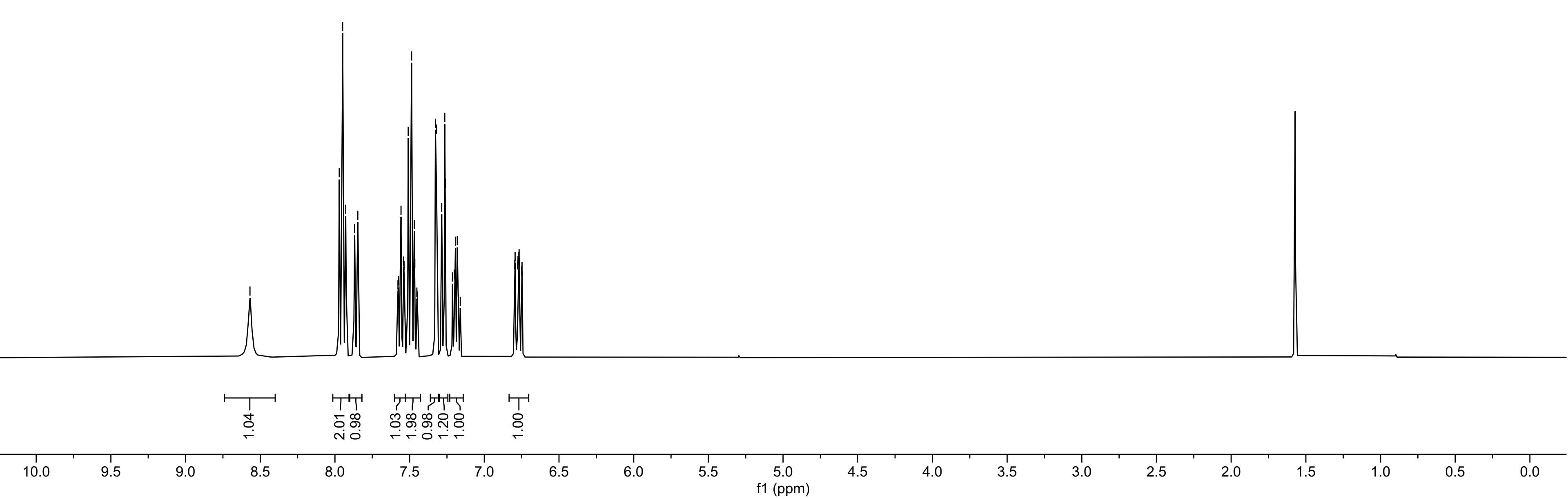

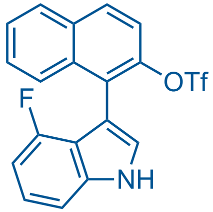

^{1}H NMR of **1c** (400 MHz, $CDCl_3$)

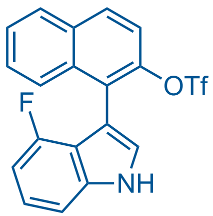

^{13}C NMR of **1c** (101 MHz, $CDCl_3$)

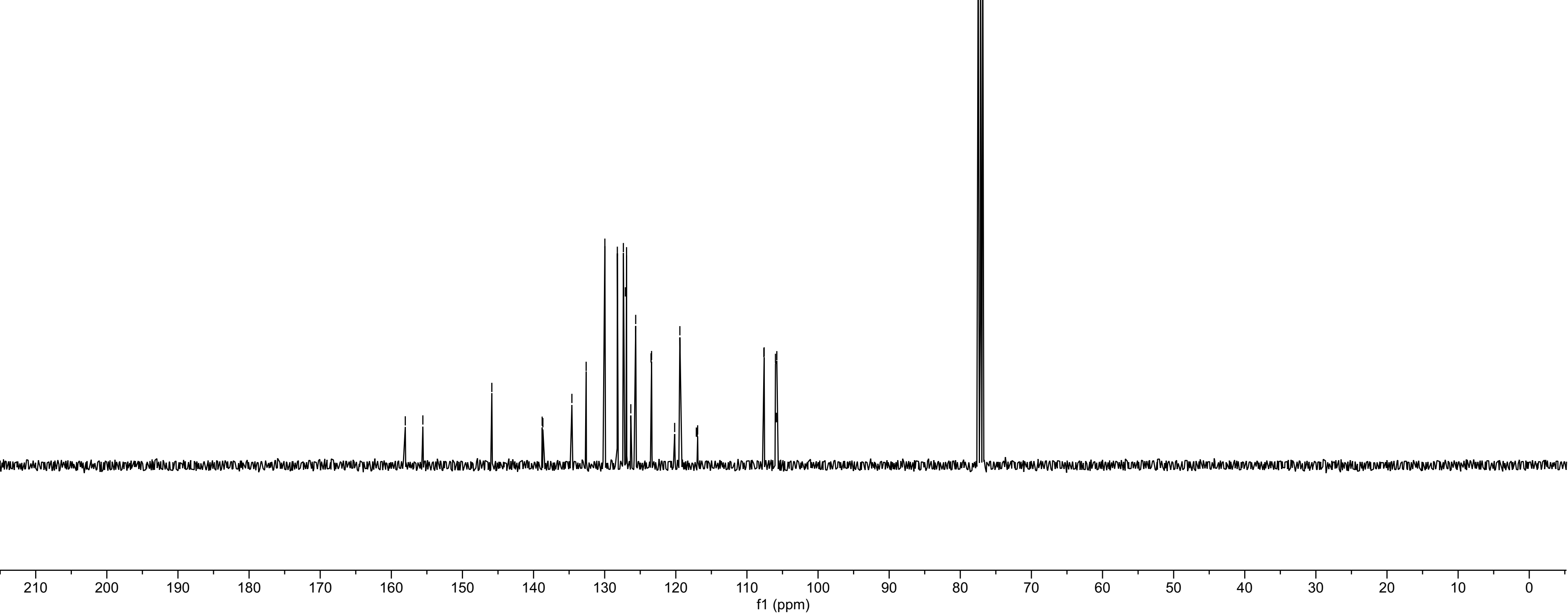

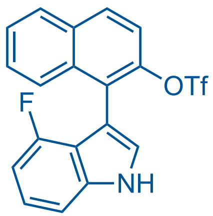

19F NMR of **1c** (376 MHz, $CDCl_3$)

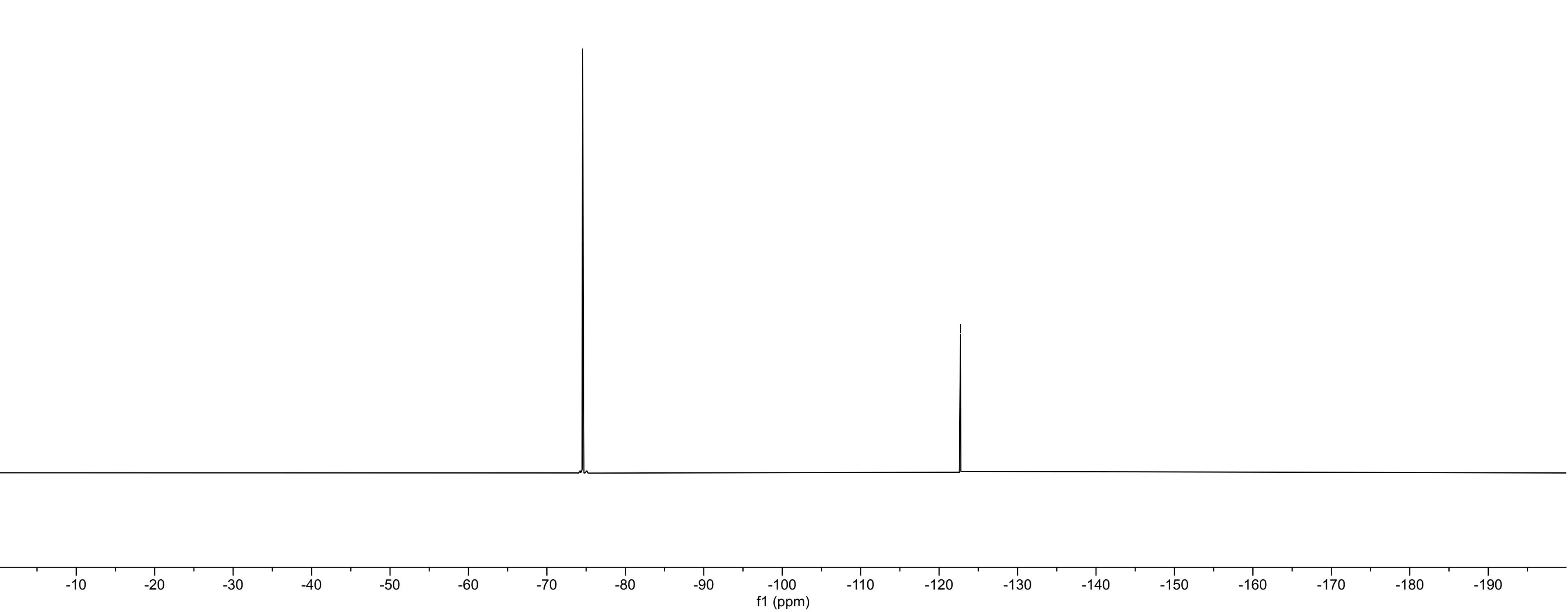

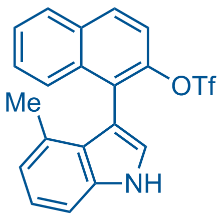

^{1}H NMR of **1d** (300 MHz, $CDCl_3$)

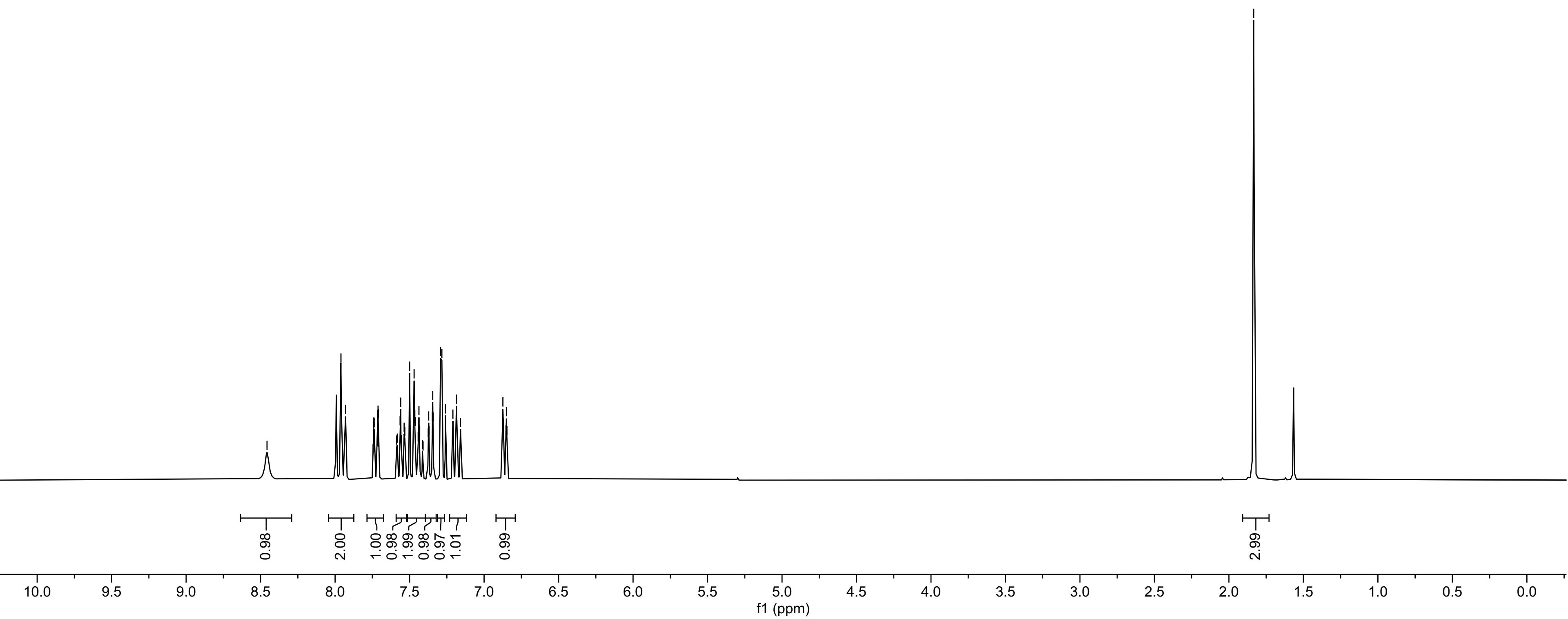

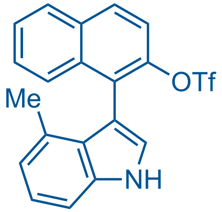

^{13}**C NMR** of **1d** (75 MHz, $CDCl_3$)

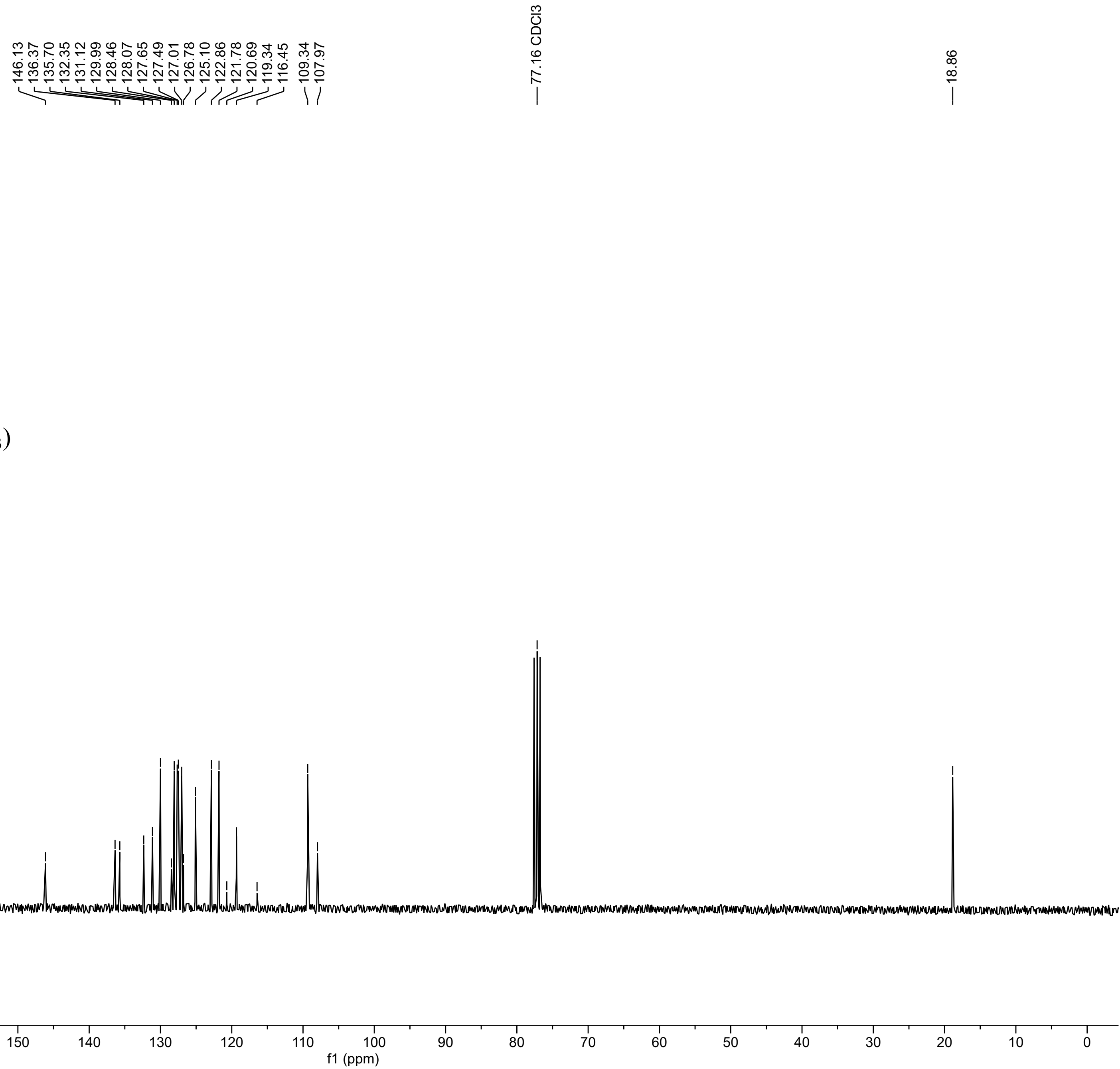

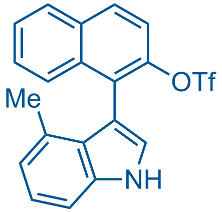

^{19}F NMR of **1d** (282 MHz, $CDCl_3$)

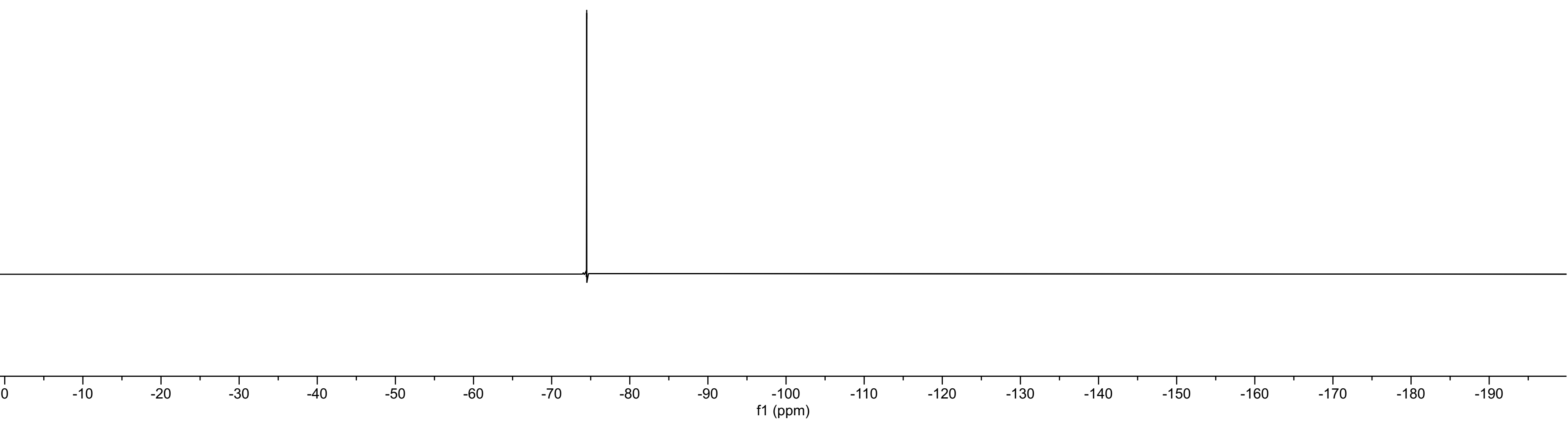

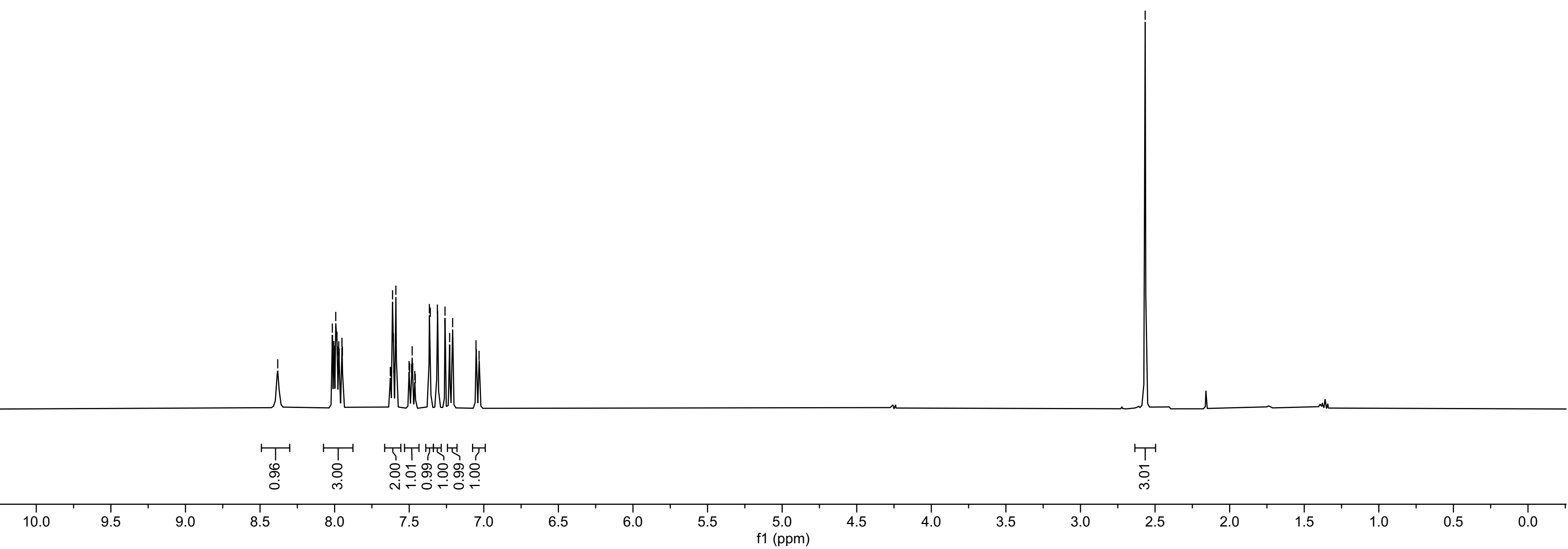

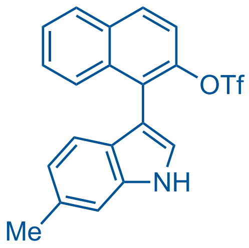

^{1}H NMR of **1j** (400 MHz, $CDCl_3$)

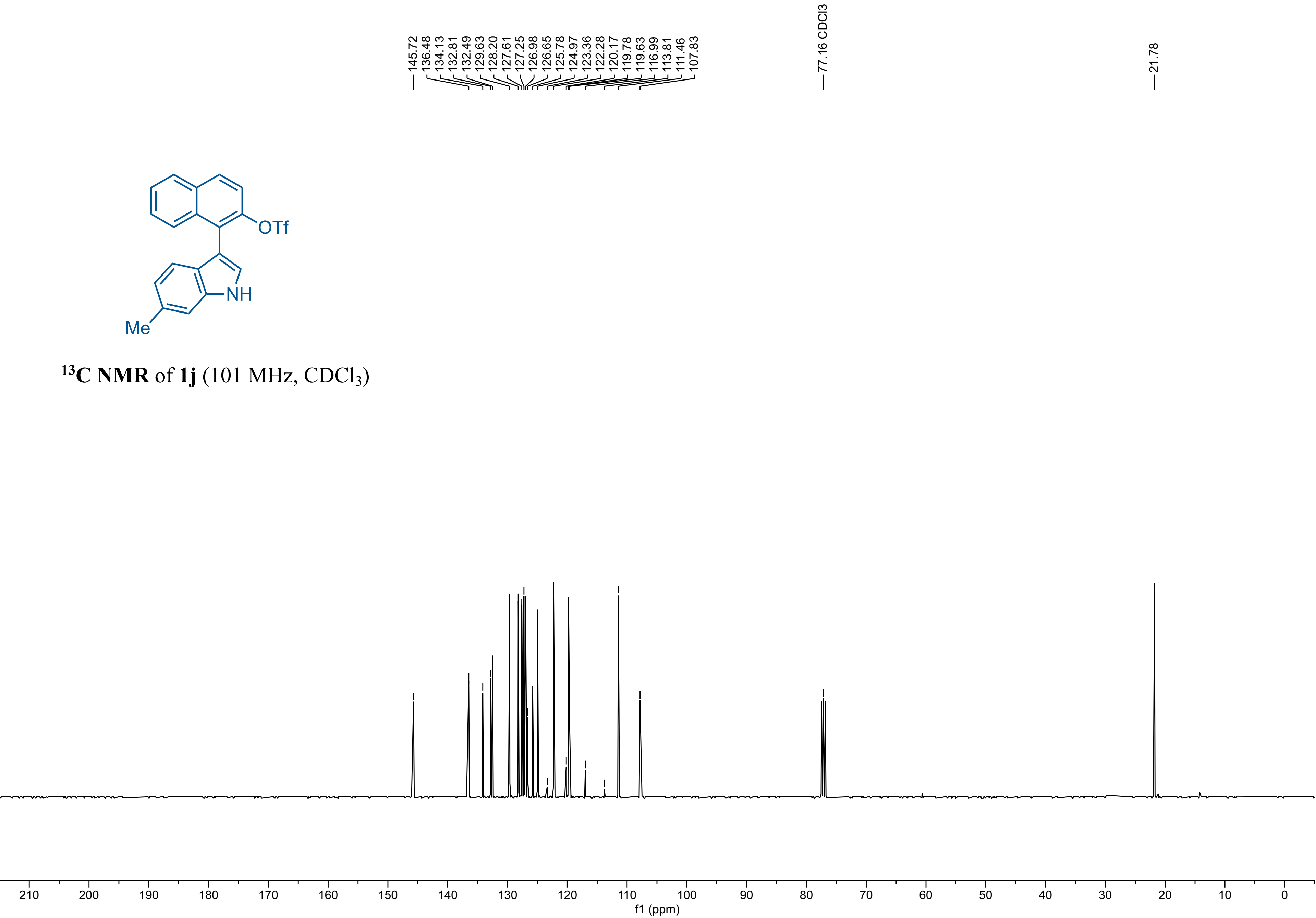

^{13}C NMR of **1j** (101 MHz, $CDCl_3$)

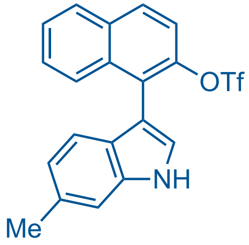

^{19}F NMR of **1j** (376 MHz, $CDCl_3$)

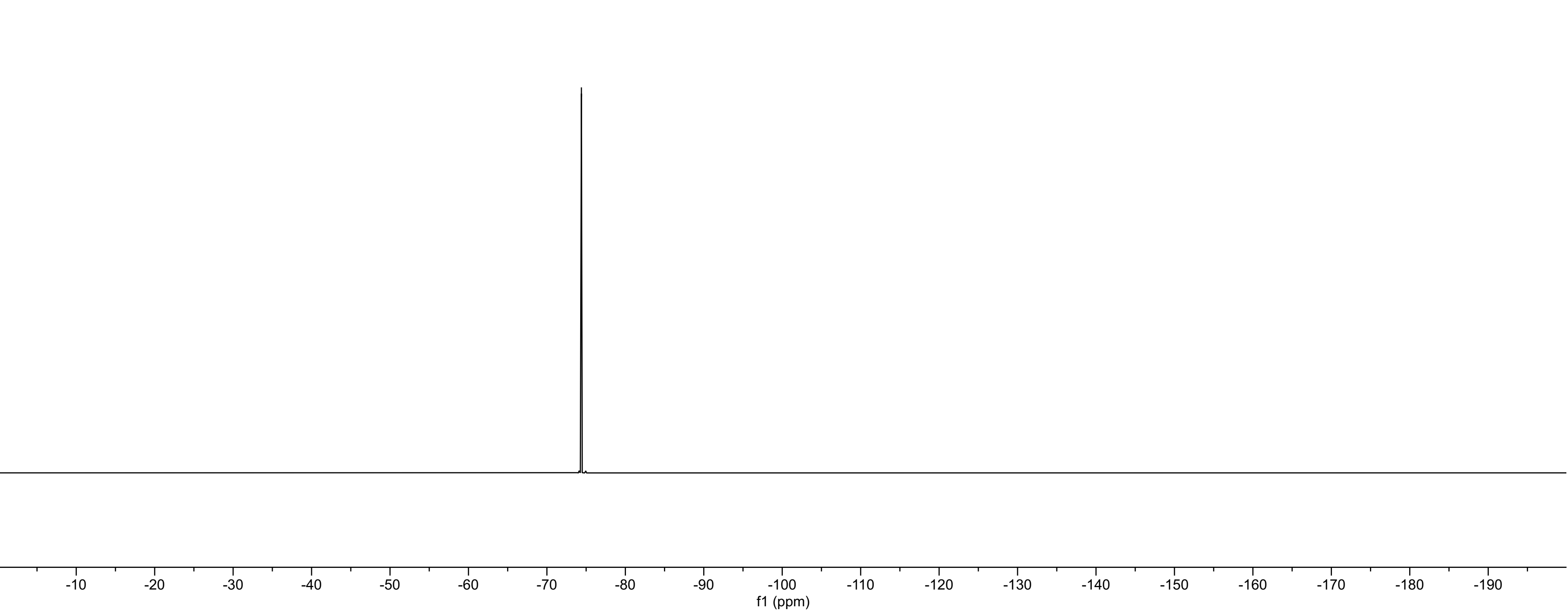

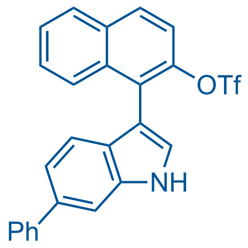

^{1}H NMR of **1k** (400 MHz, $CDCl_3$)

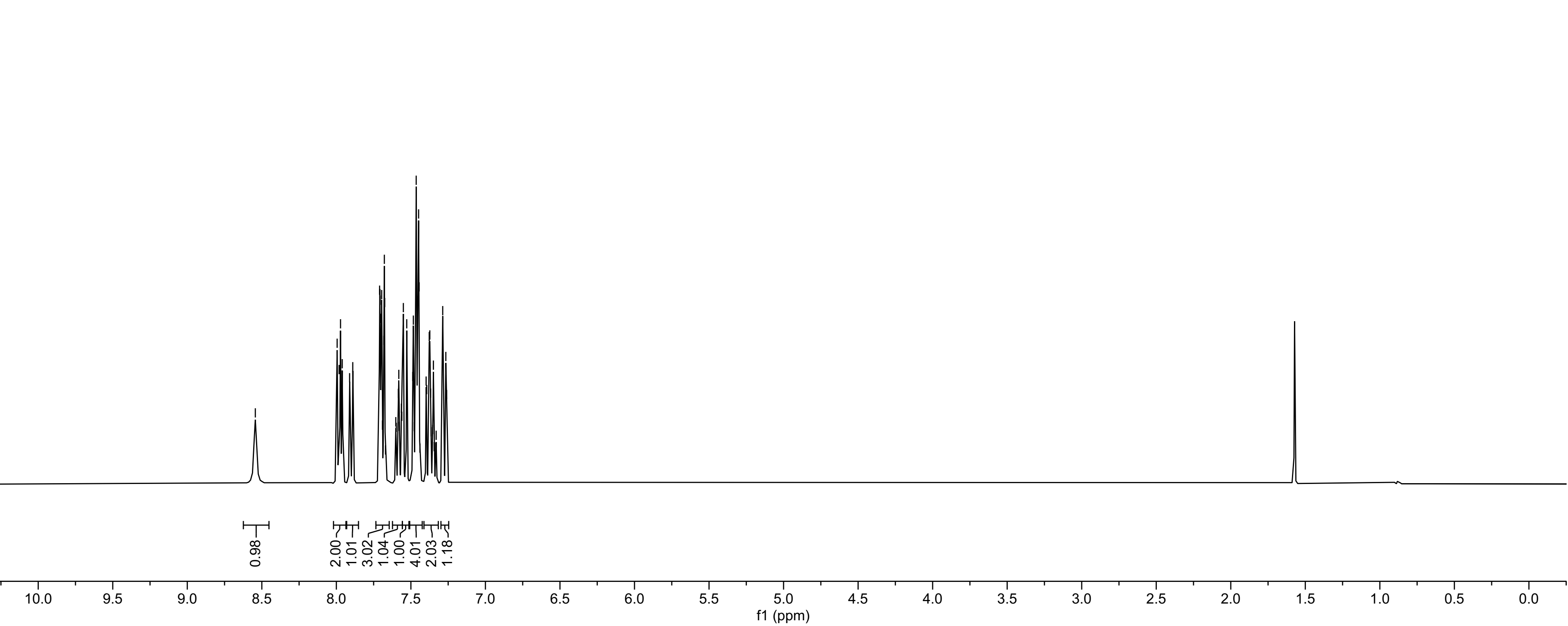

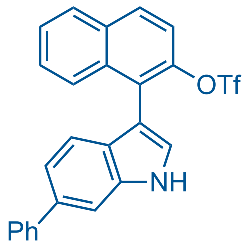

^{13}C NMR of **1k** (101 MHz, $CDCl_3$)

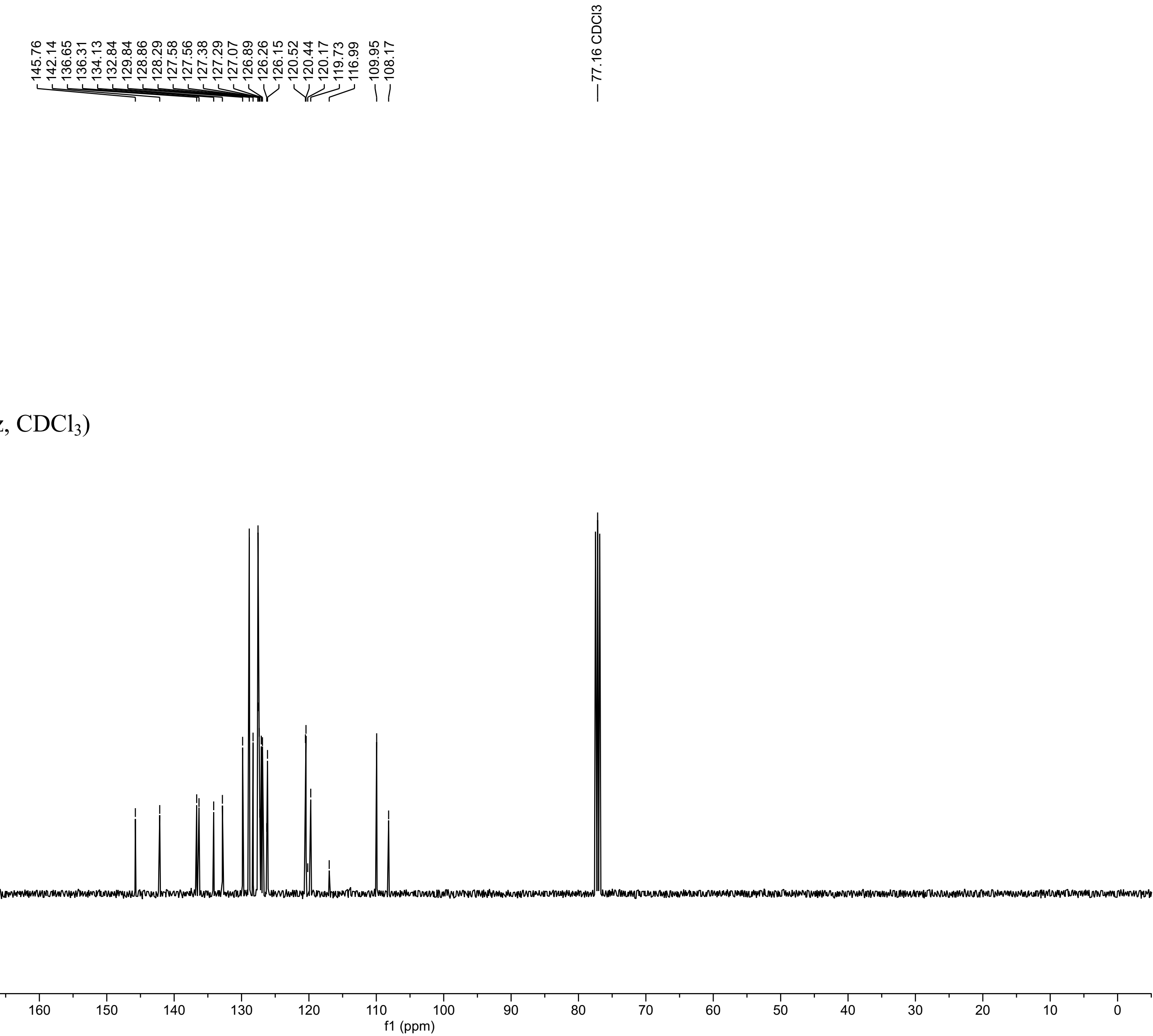

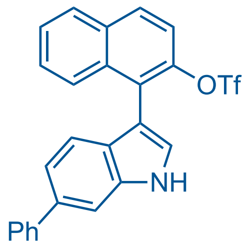

^{19}F NMR of **1k** (376 MHz, $CDCl_3$)

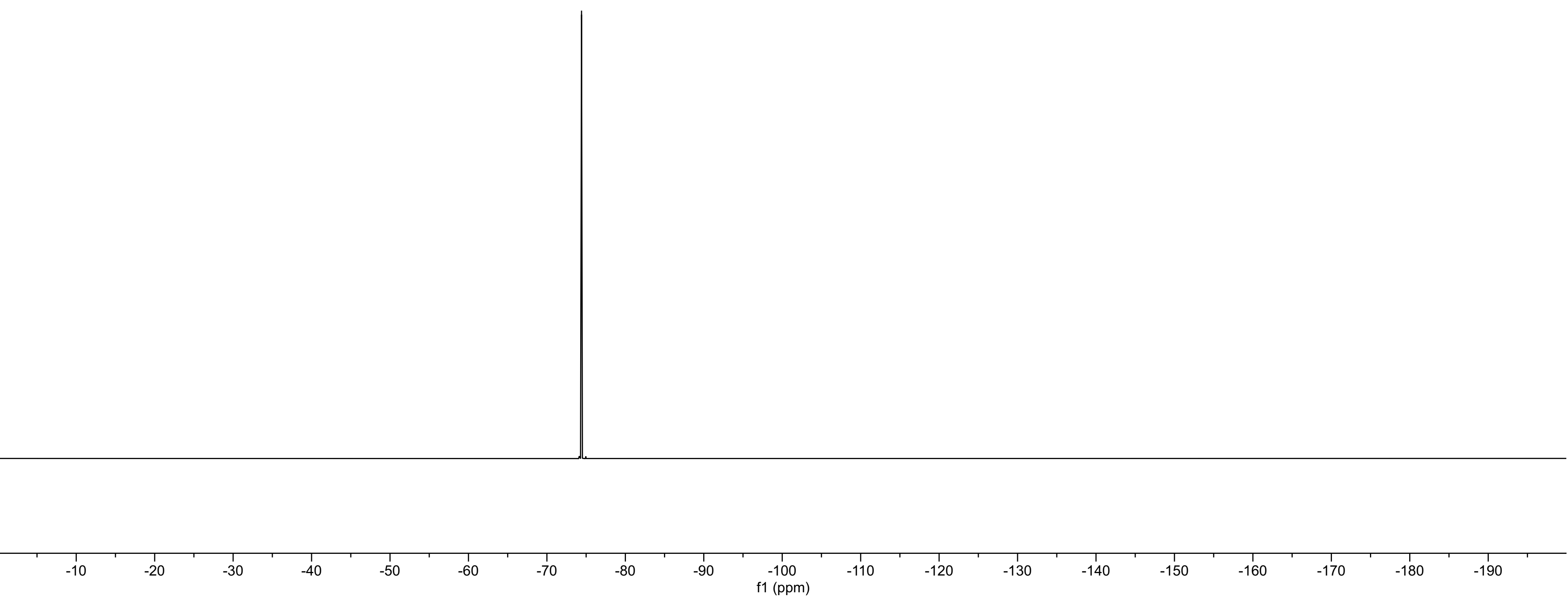

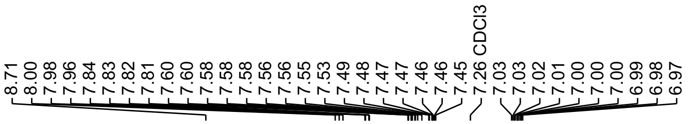

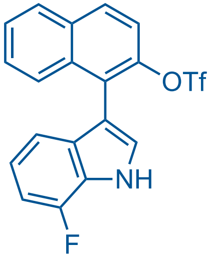

^{1}H NMR of **1l** (400 MHz, $CDCl_3$)

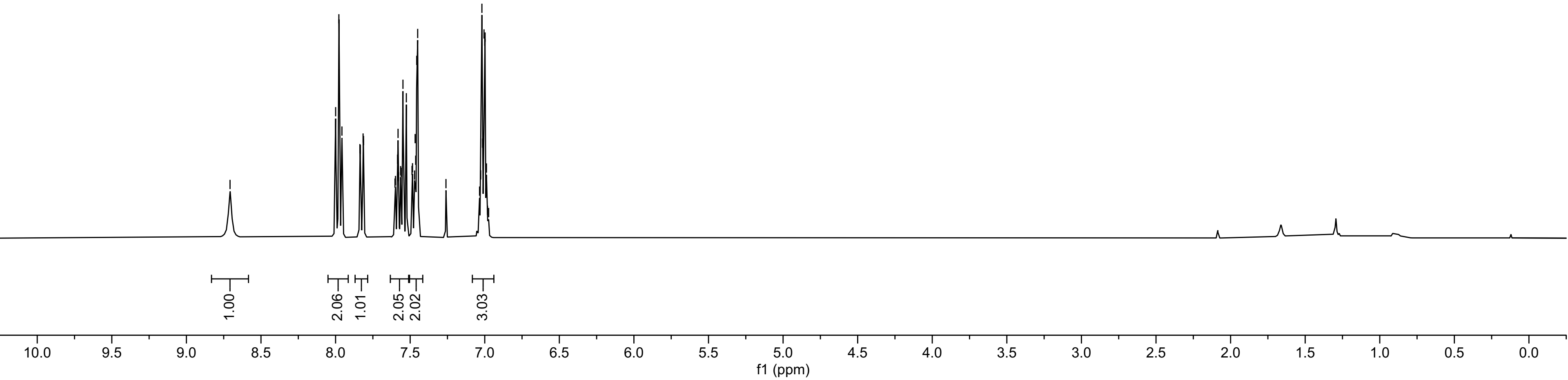

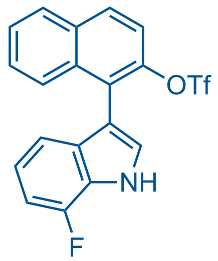

^{13}C NMR of **1l** (101 MHz, $CDCl_3$)

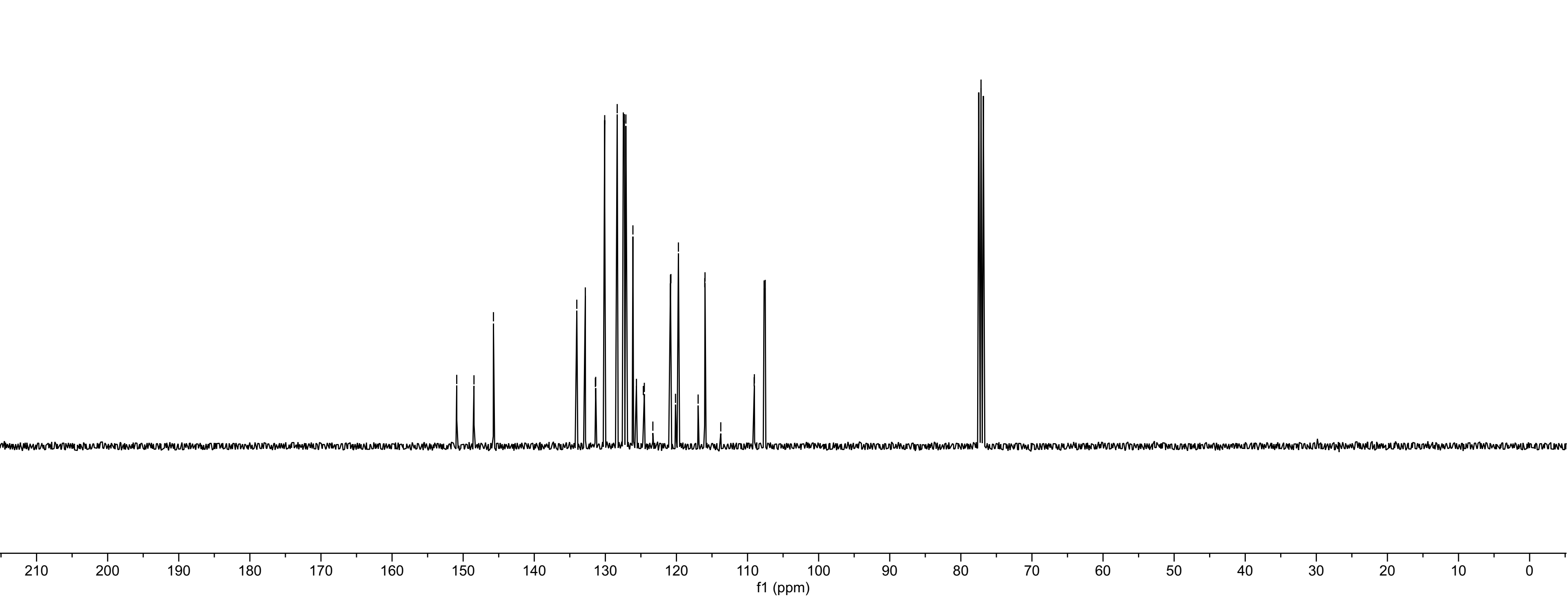

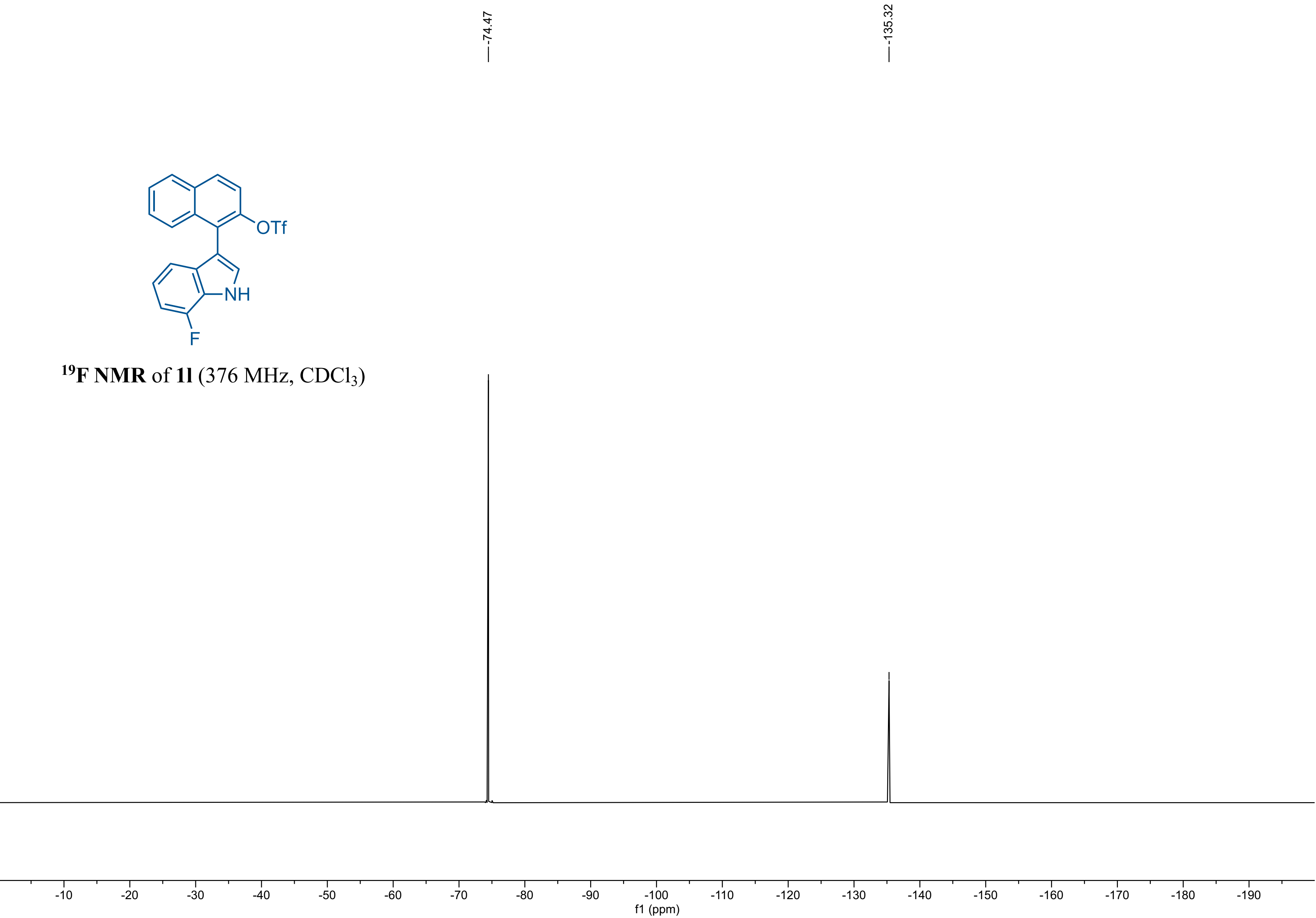

^{19}**F NMR** of **1l** (376 MHz, $CDCl_3$)

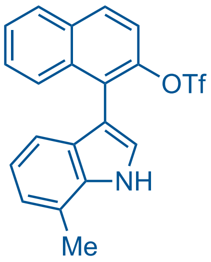

^{1}H NMR of **1m** (400 MHz, $CDCl_3$)

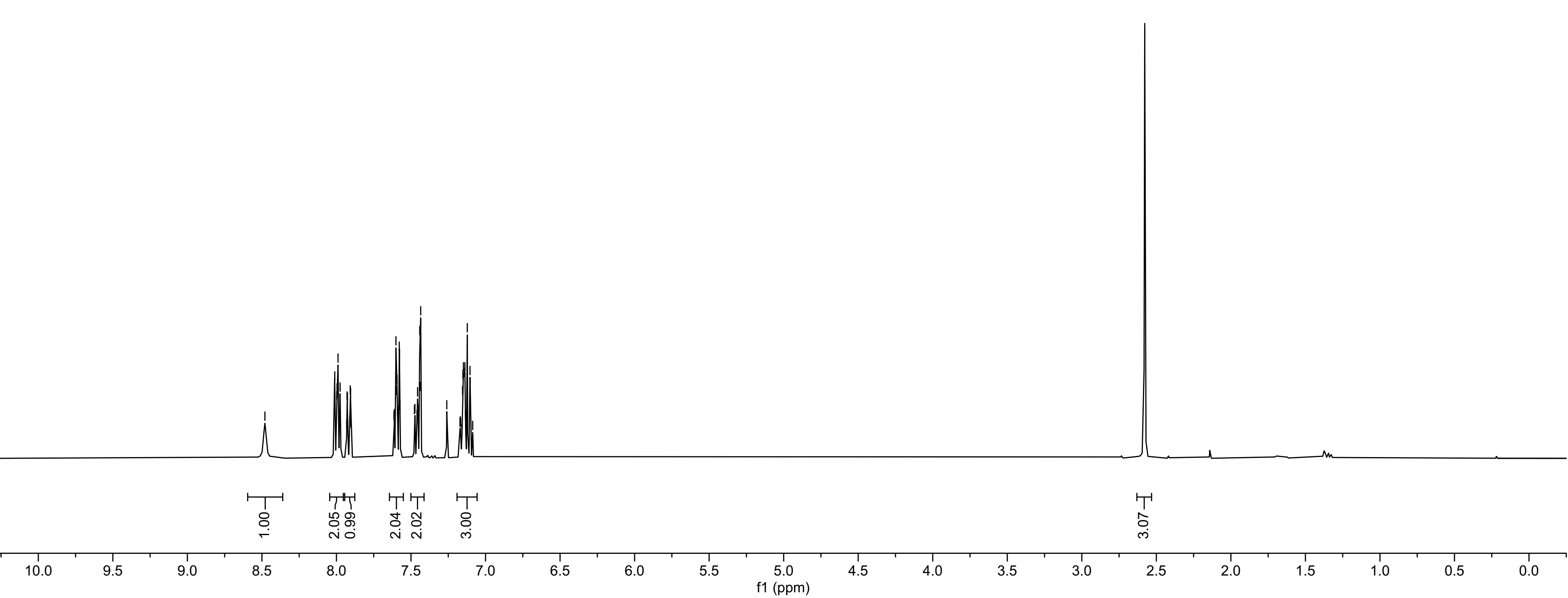

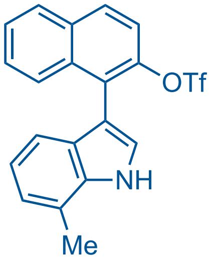

^{13}C NMR of **1m** (101 MHz, $CDCl_3$)

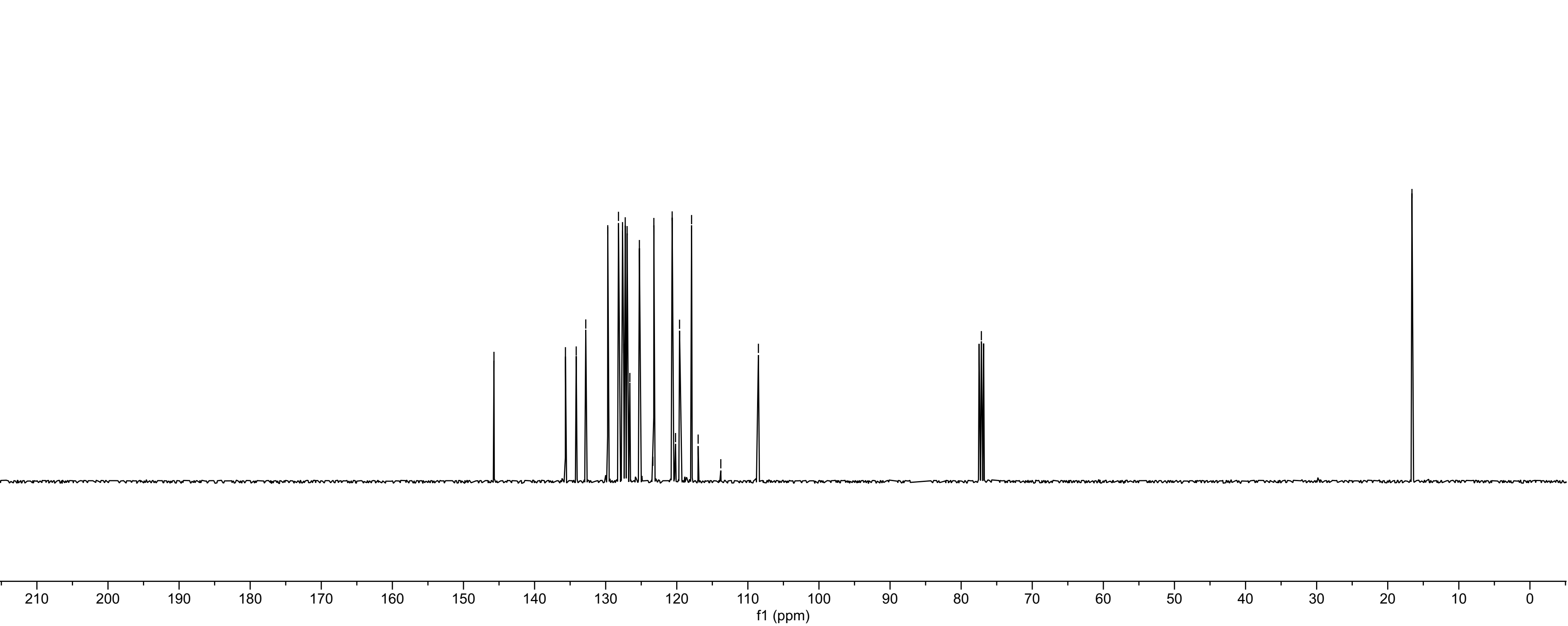

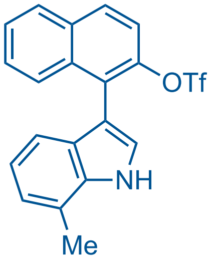

^{19}F NMR of **1m** (376 MHz, $CDCl_3$)

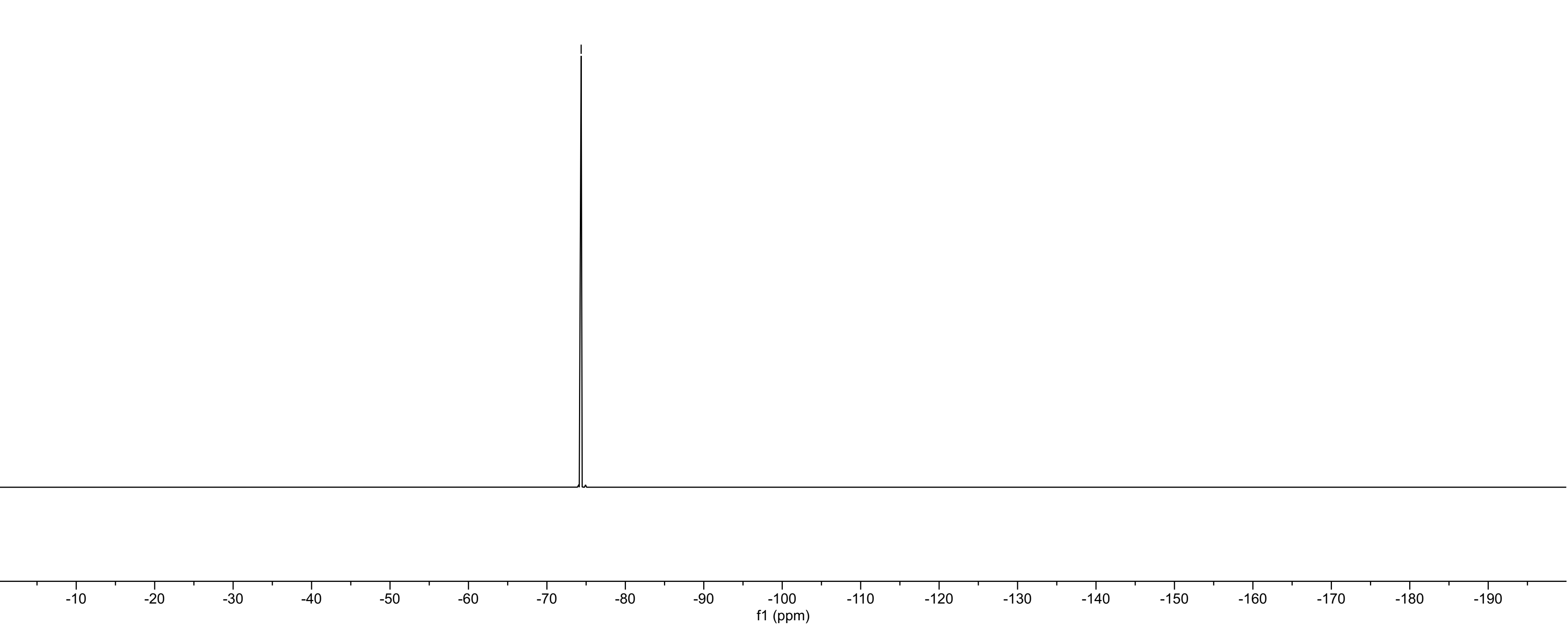

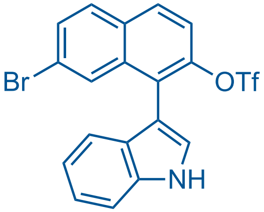

^{1}H NMR of **1p** (400 MHz, $CDCl_3$)

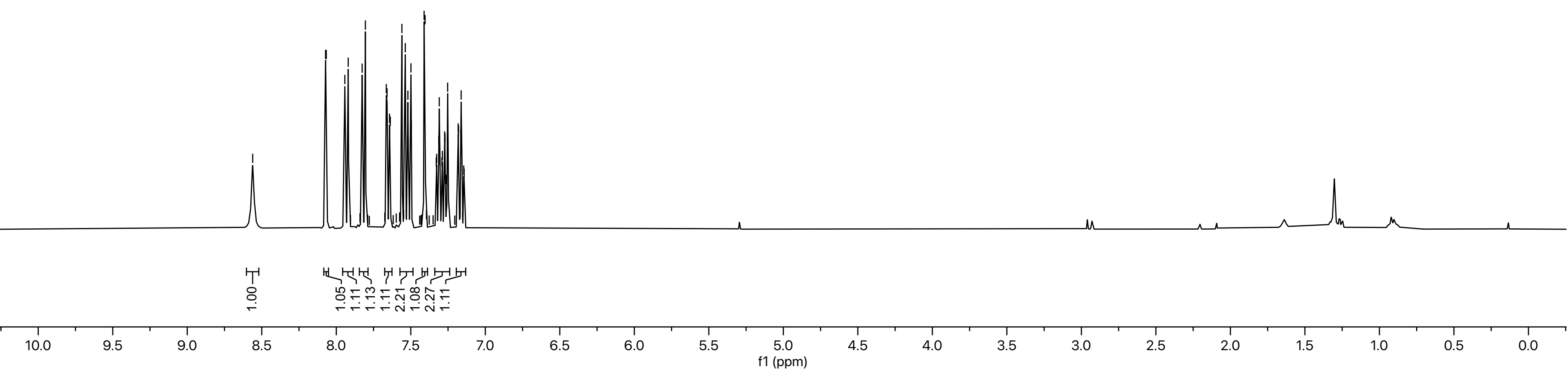

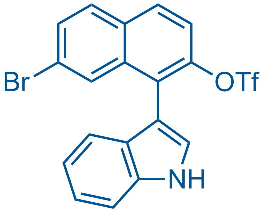

^{13}C NMR of **1p** (101 MHz, $CDCl_3$)

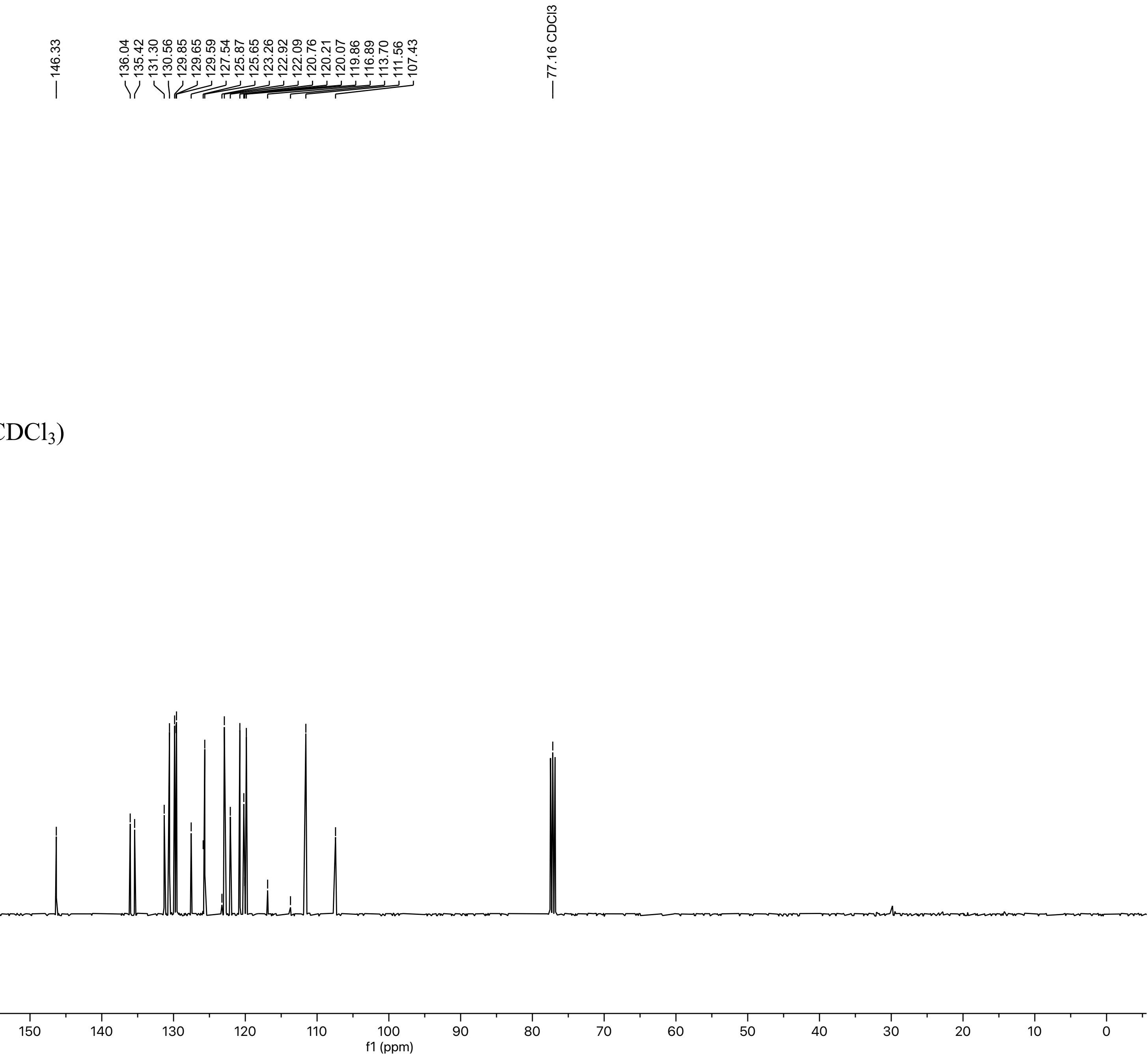

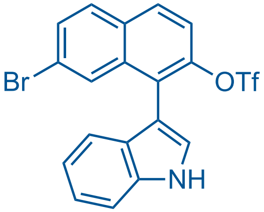

^{19}F NMR of 1p (376 MHz, $CDCl_3$)

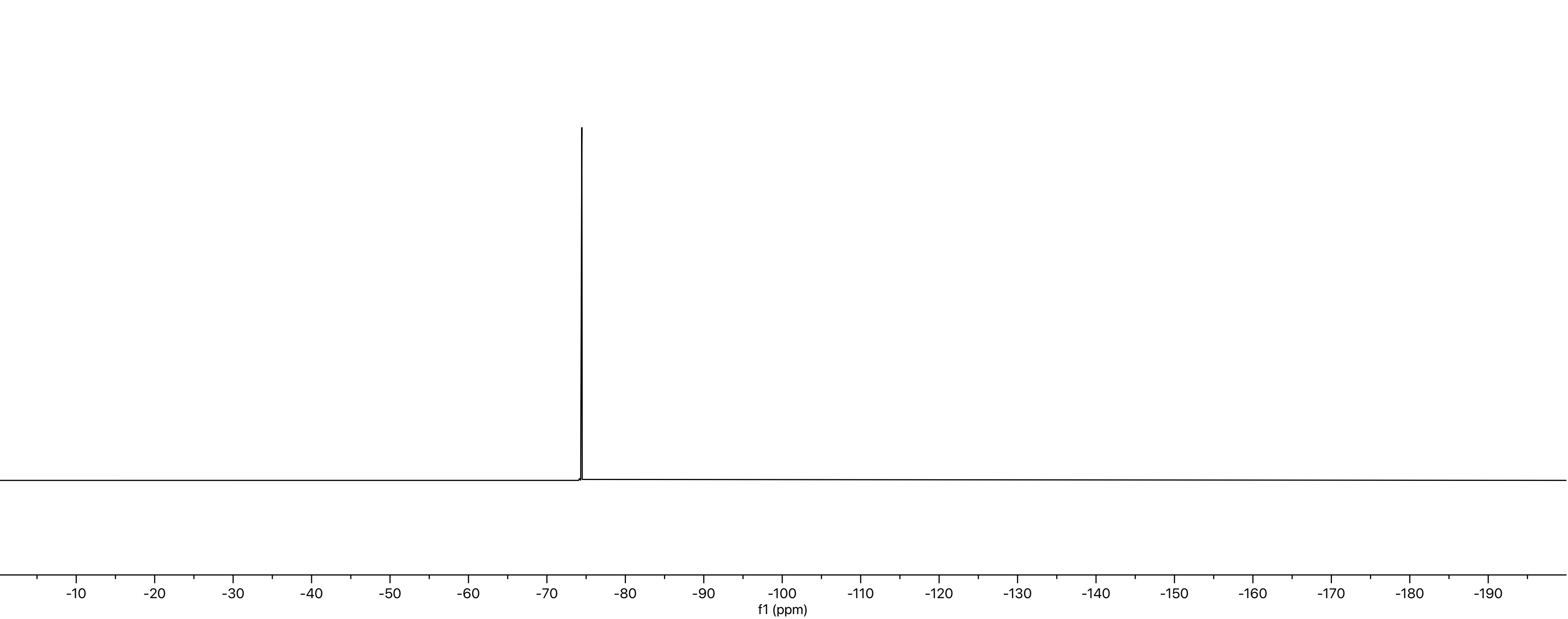

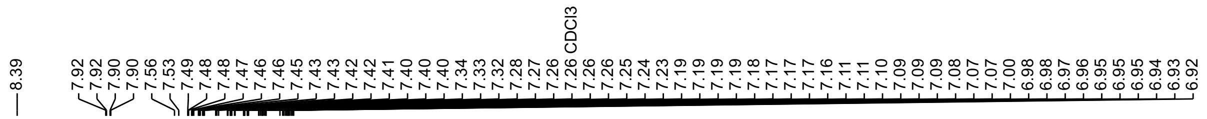

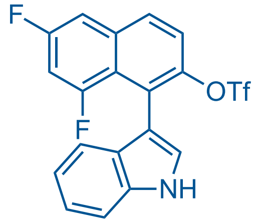

^{1}H NMR of **1r** (400 MHz, $CDCl_3$)

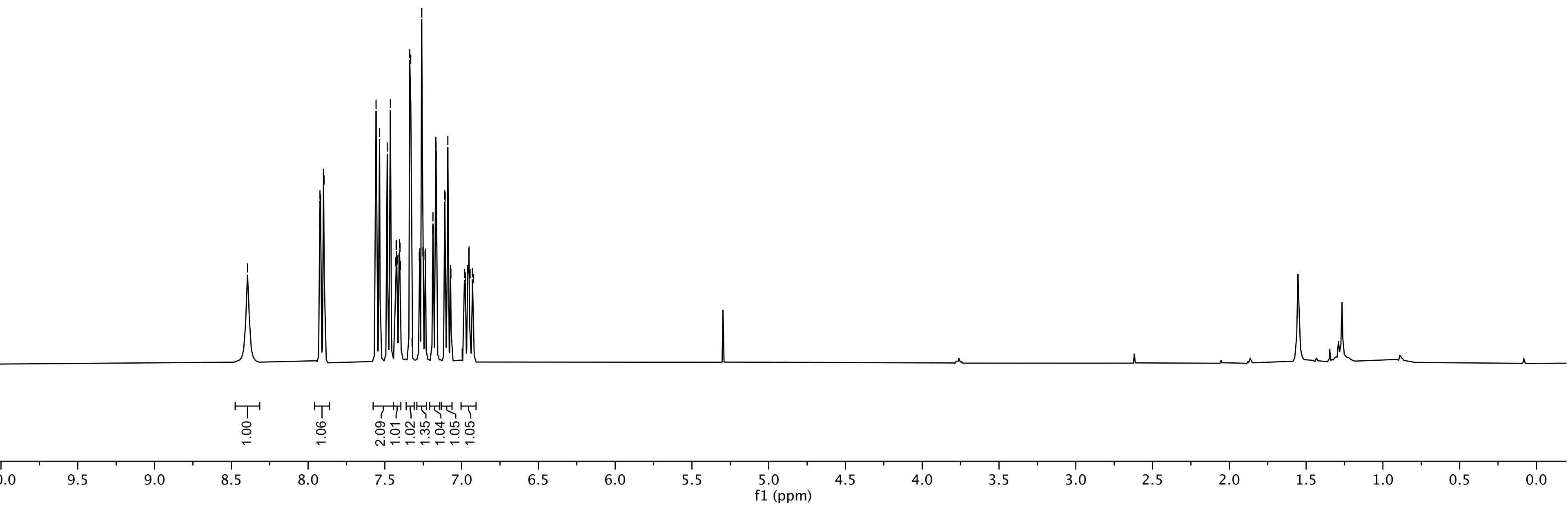

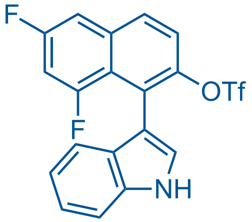

^{13}C NMR of **1r** (101 MHz, $CDCl_3$)

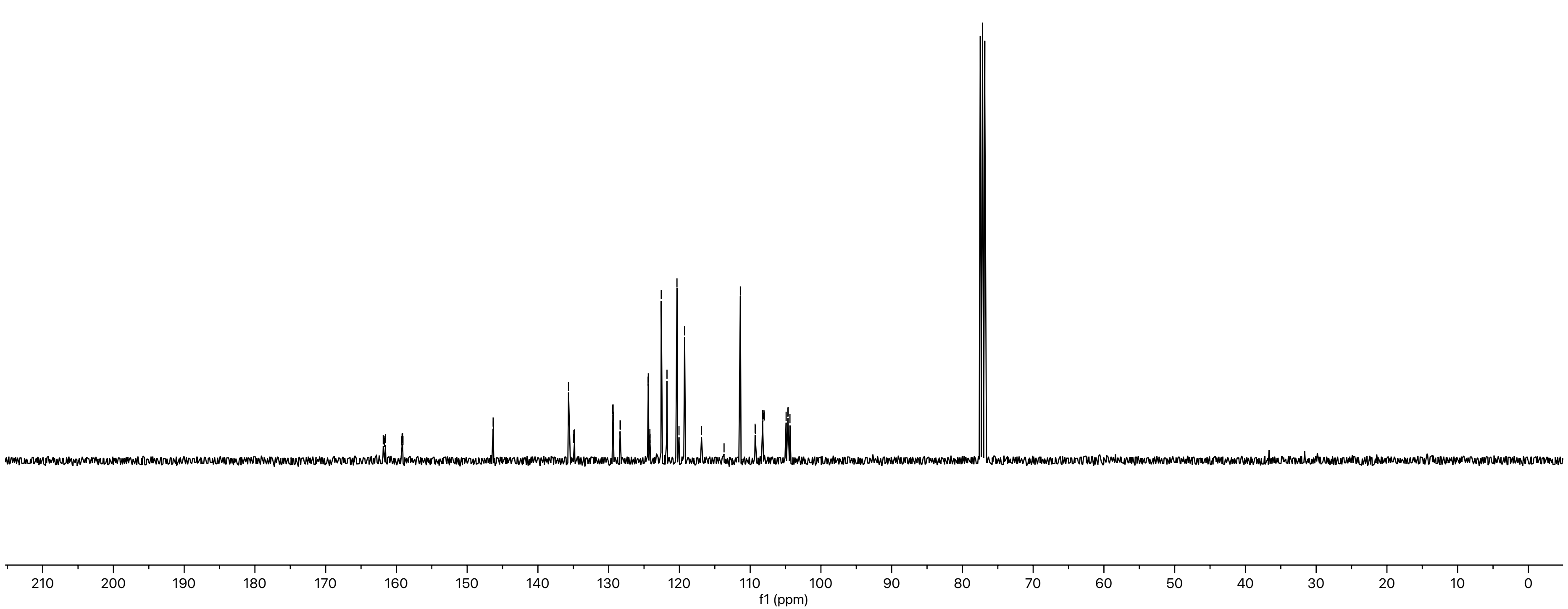

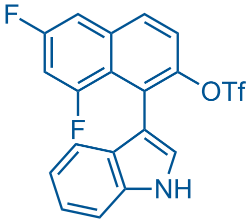

^{19}F NMR of **1r** (376 MHz, $CDCl_3$)

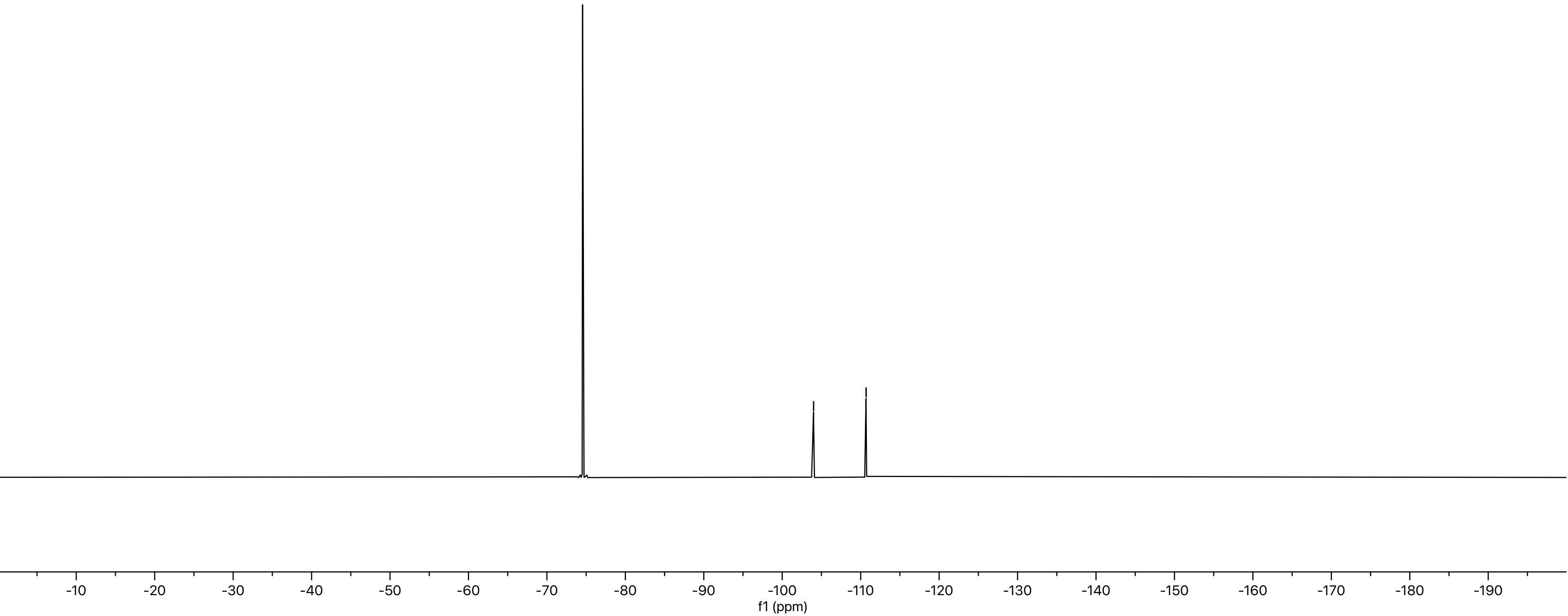

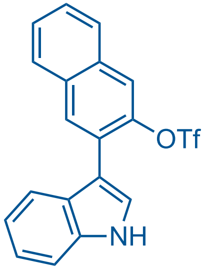

^{1}H NMR of **1s** (400 MHz, $CDCl_3$)

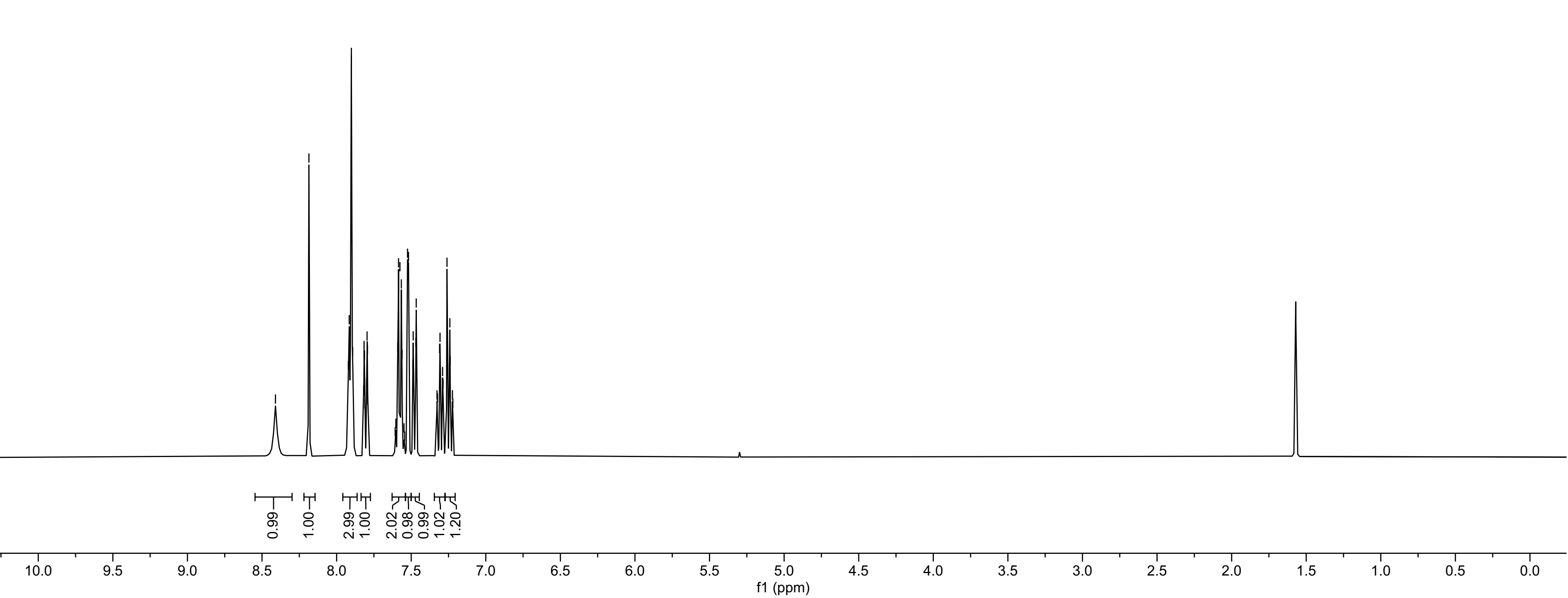

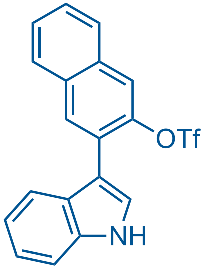

^{13}**C NMR** of **1s** (101 MHz, $CDCl_3$)

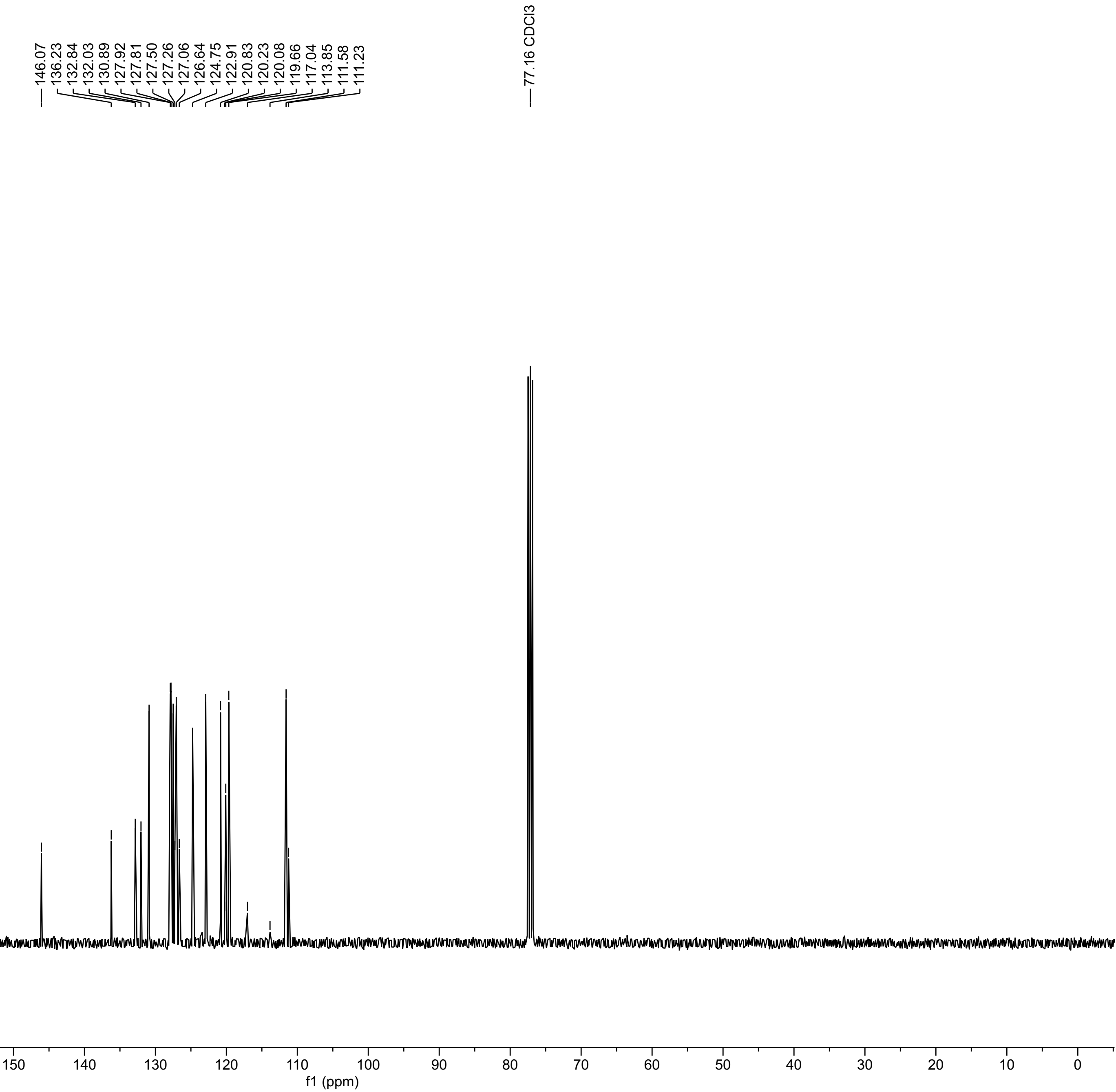

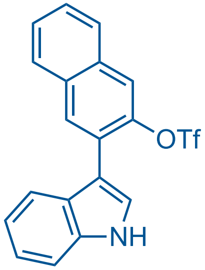

^{19}F NMR of **1s** (376 MHz, $CDCl_3$)

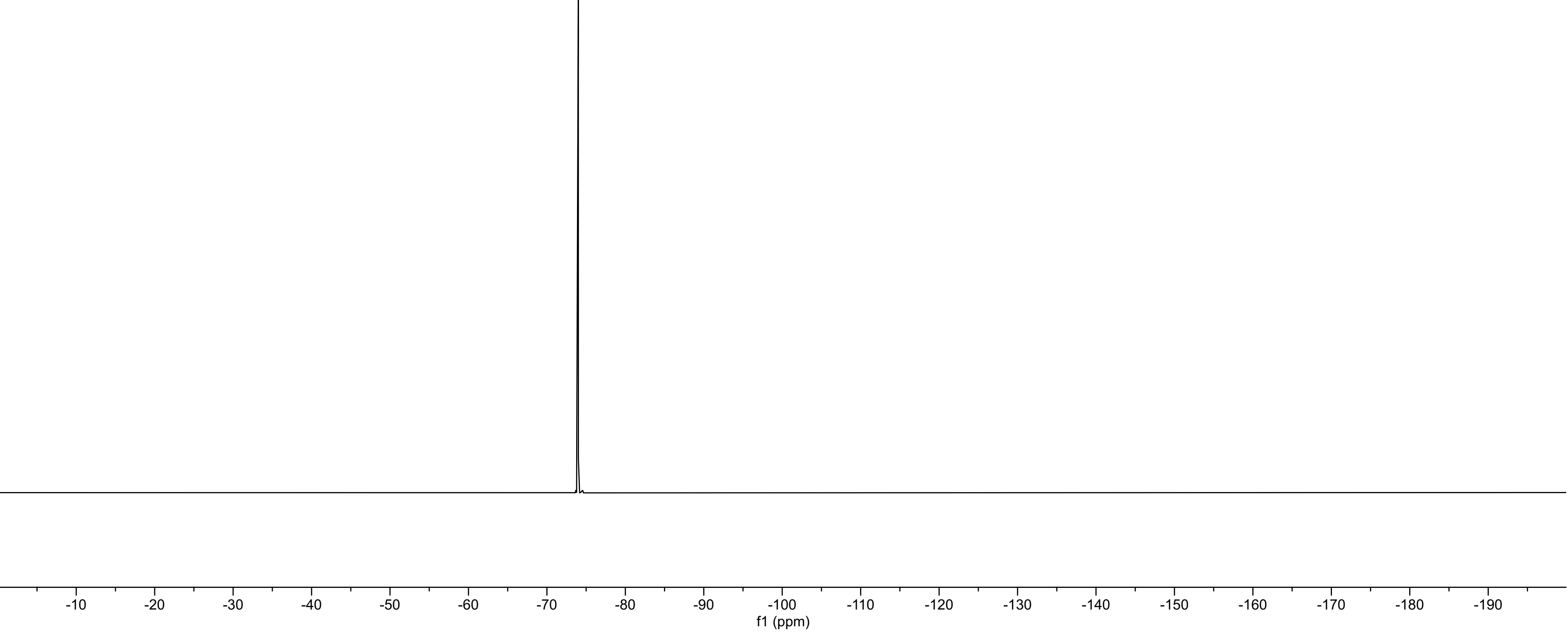

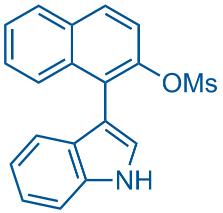

^{1}H NMR of **1t** (300 MHz, $CDCl_3$)

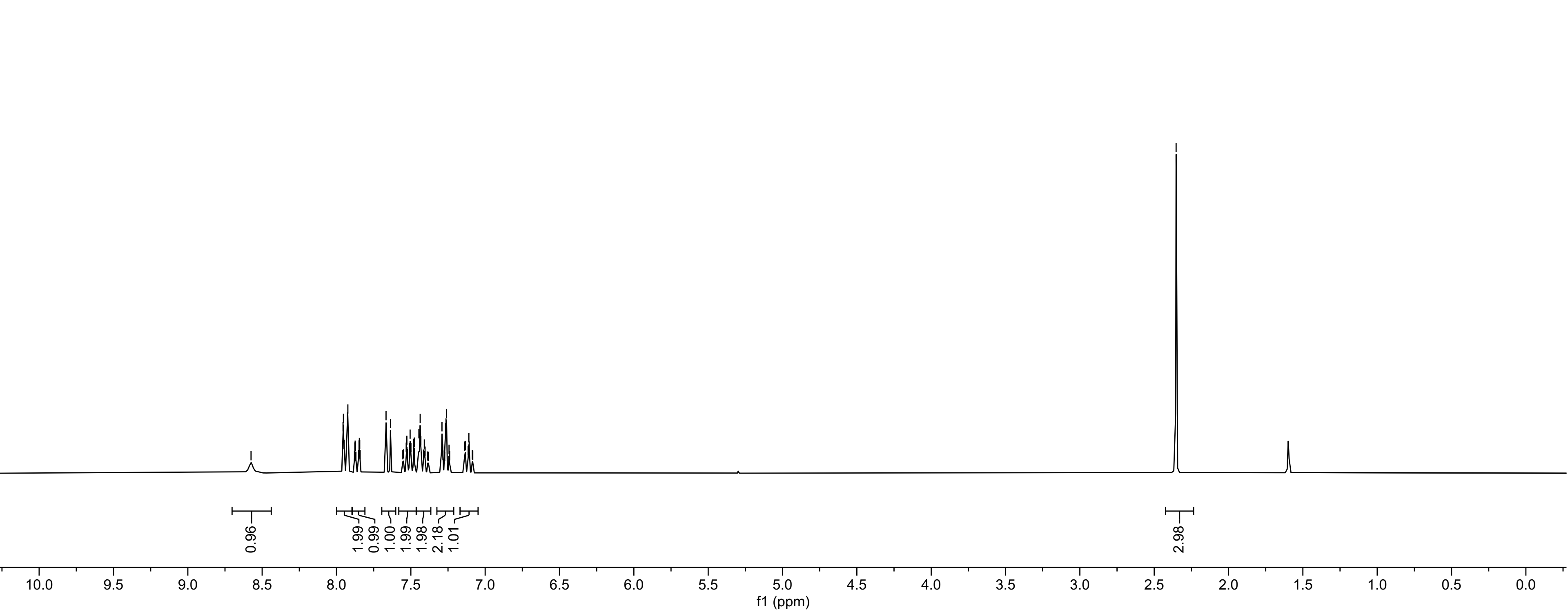

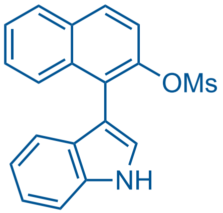

^{13}C NMR of **1t** (75 MHz, $CDCl_3$)

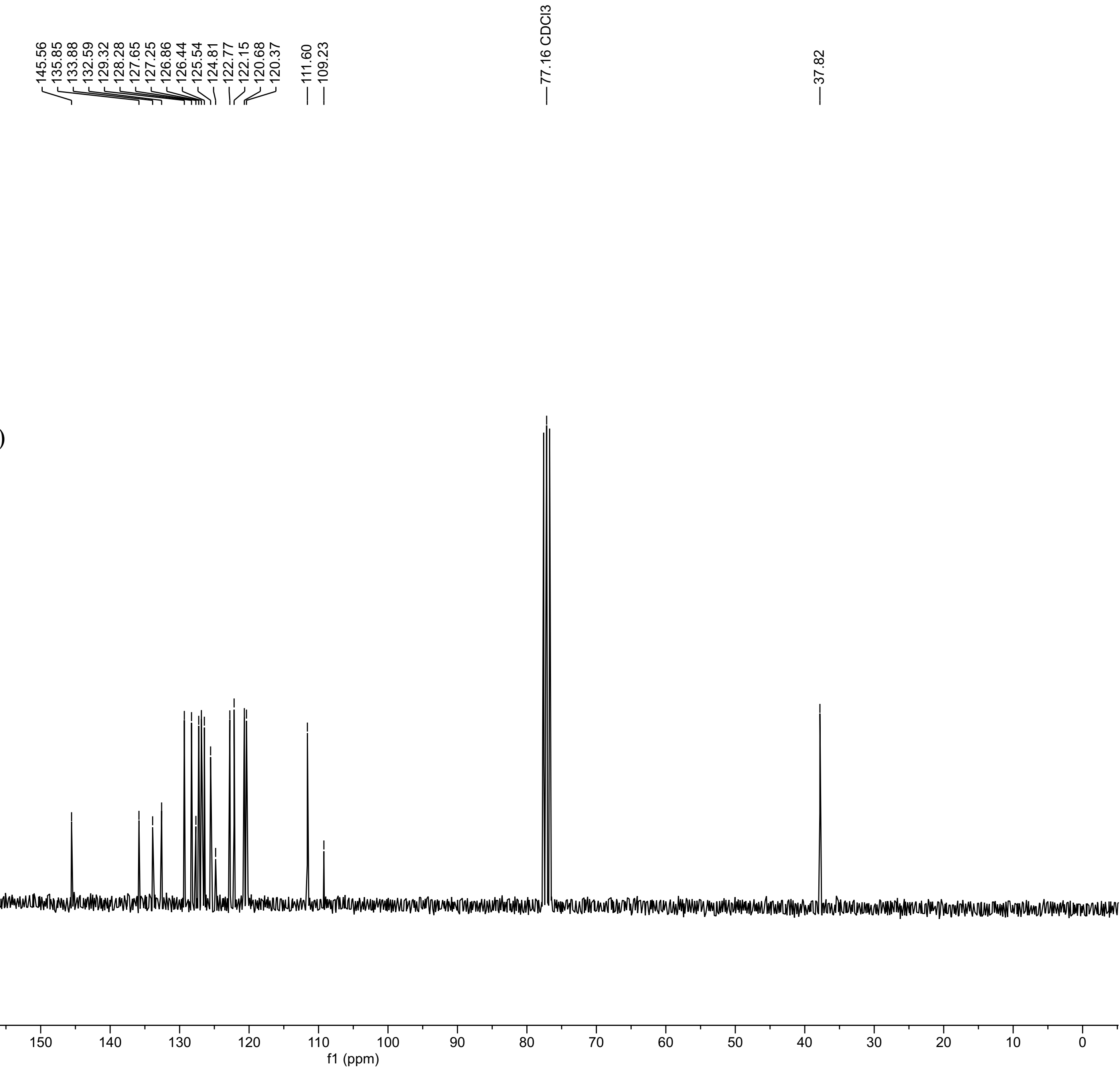

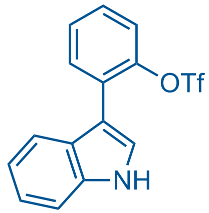

^{1}H NMR of **1u** (400 MHz, $CDCl_3$)

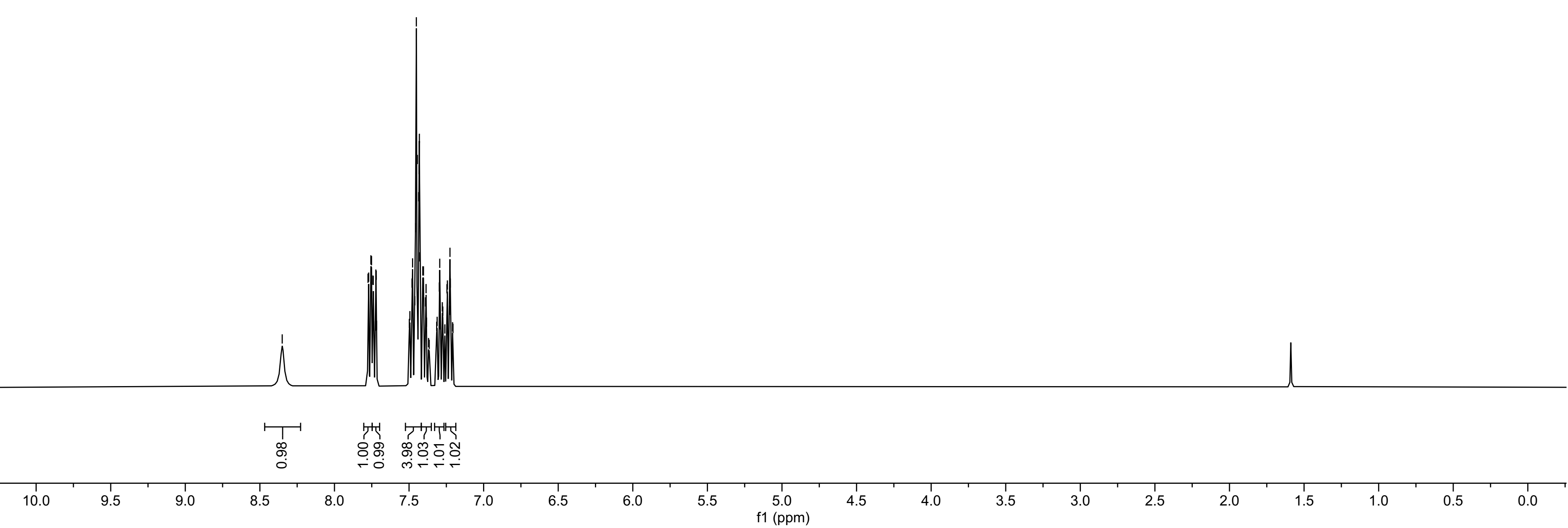

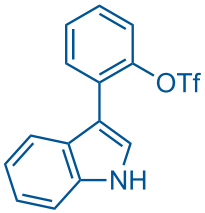

^{13}C NMR of **1u** (101 MHz, $CDCl_3$)

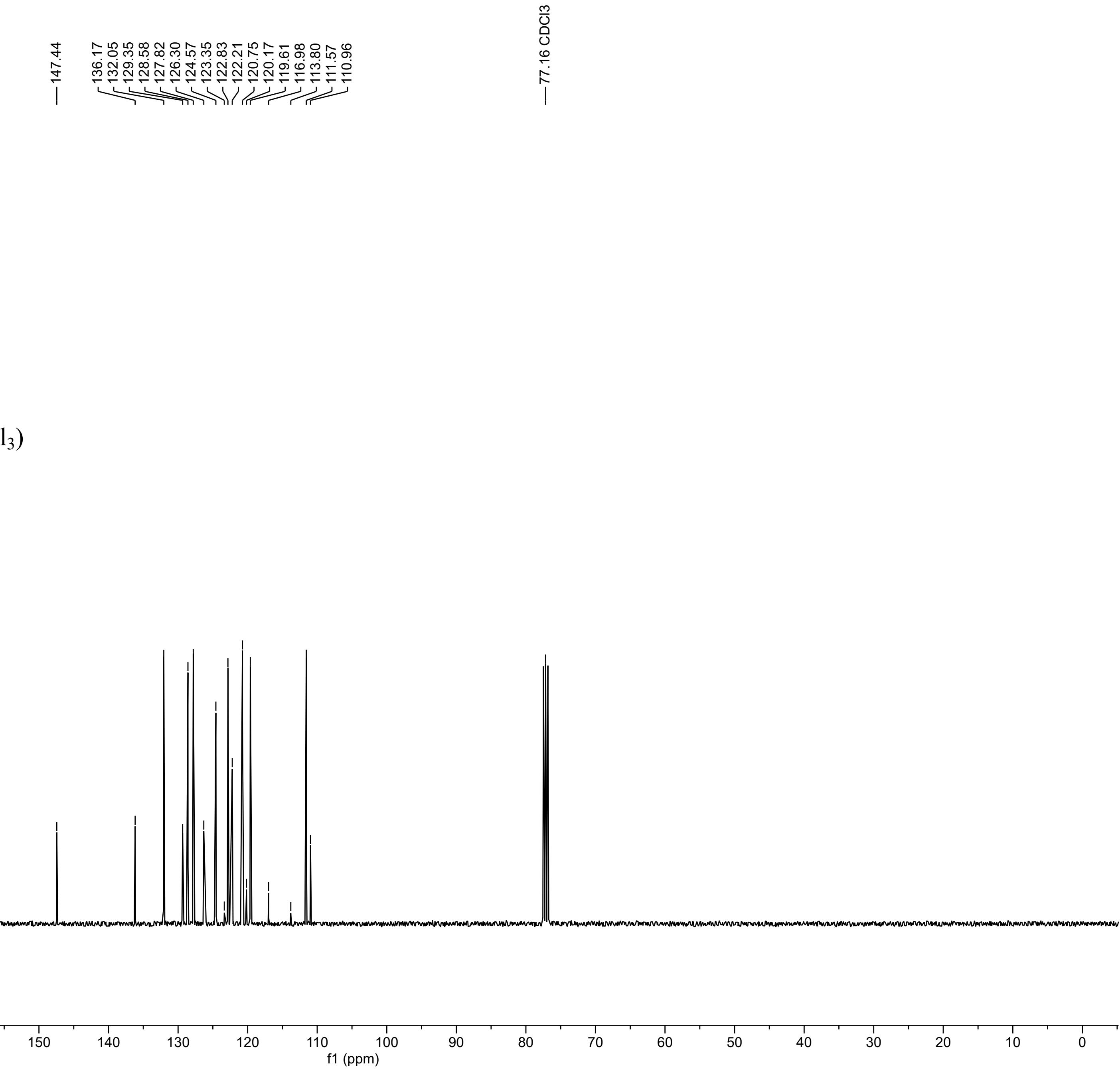

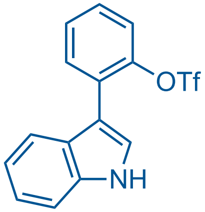

^{19}F NMR of **1u** (376 MHz, $CDCl_3$)

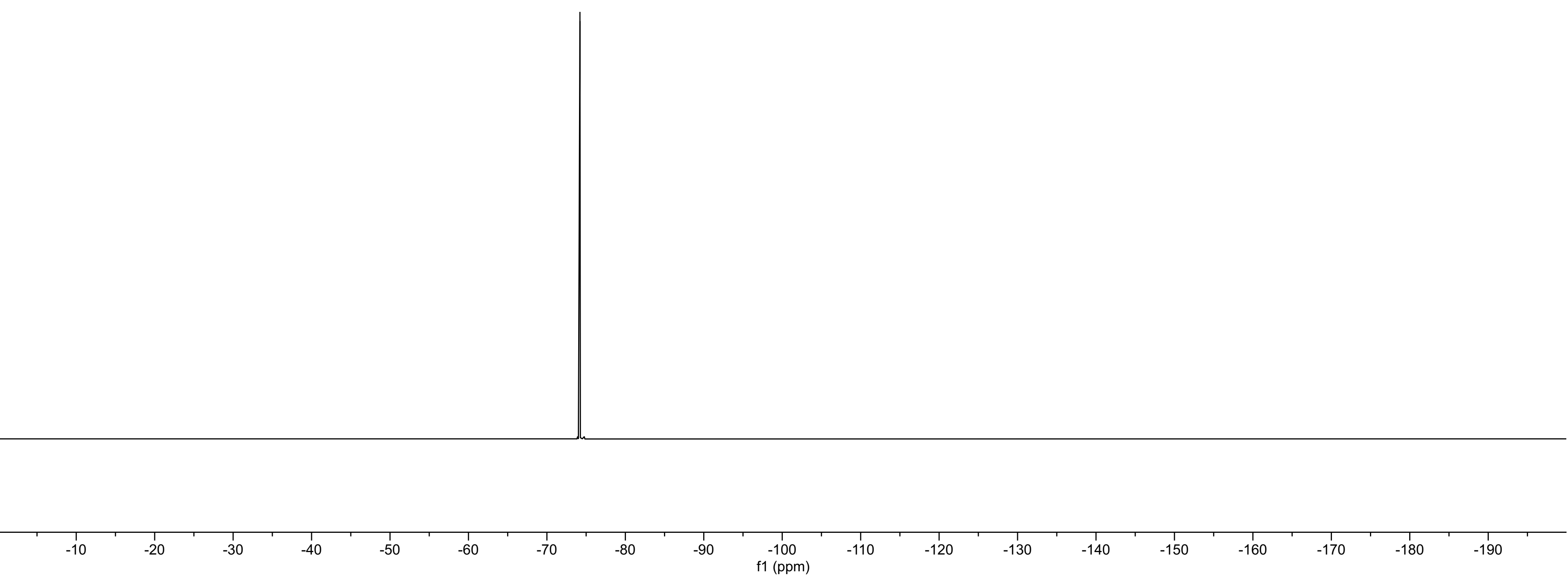

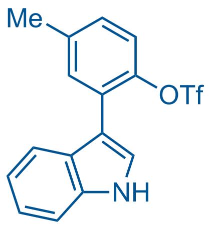

^{1}H NMR of **1v** (400 MHz, $CDCl_3$)

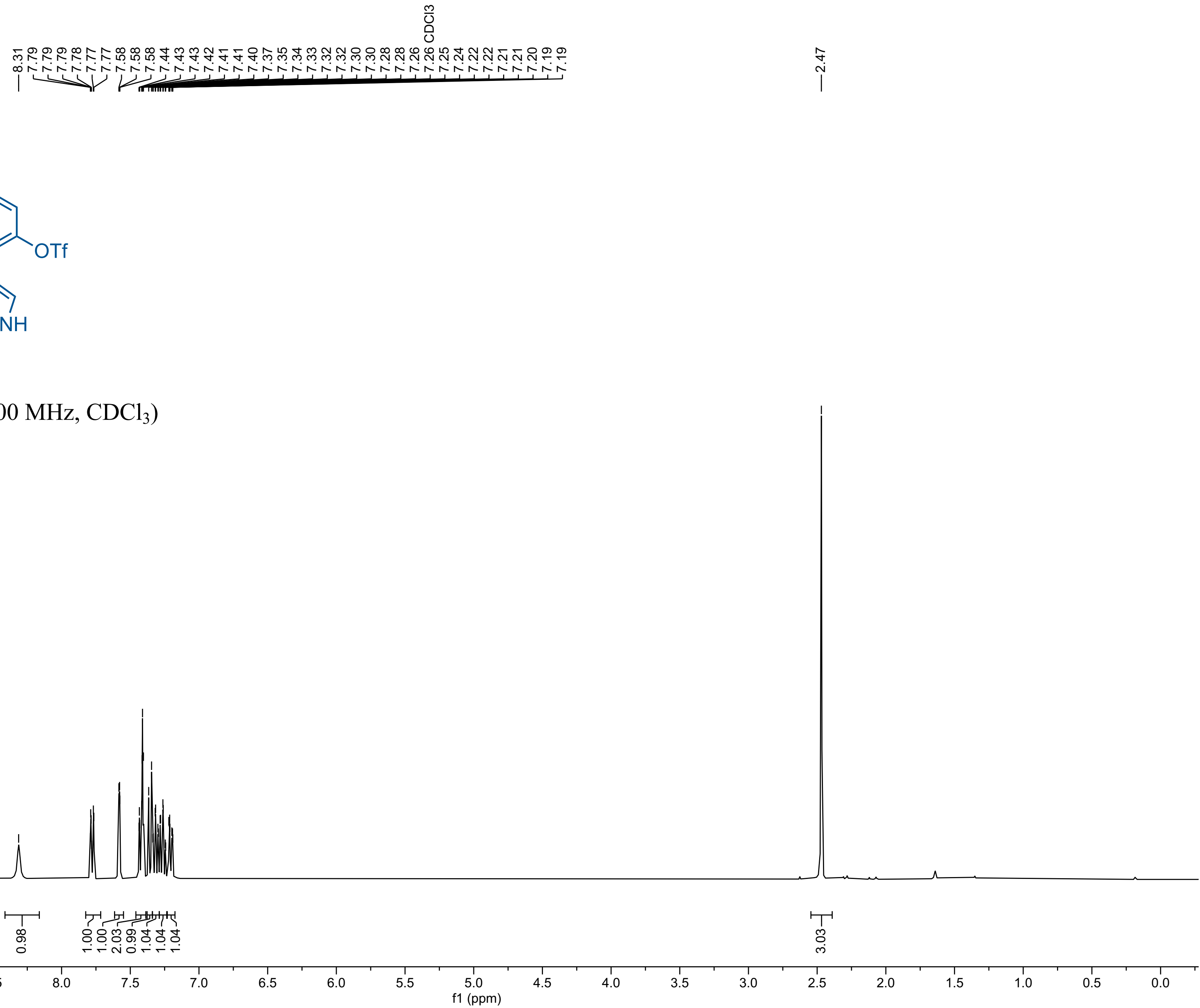

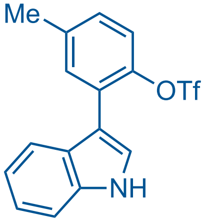

^{13}C NMR of **1v** (101 MHz, $CDCl_3$)

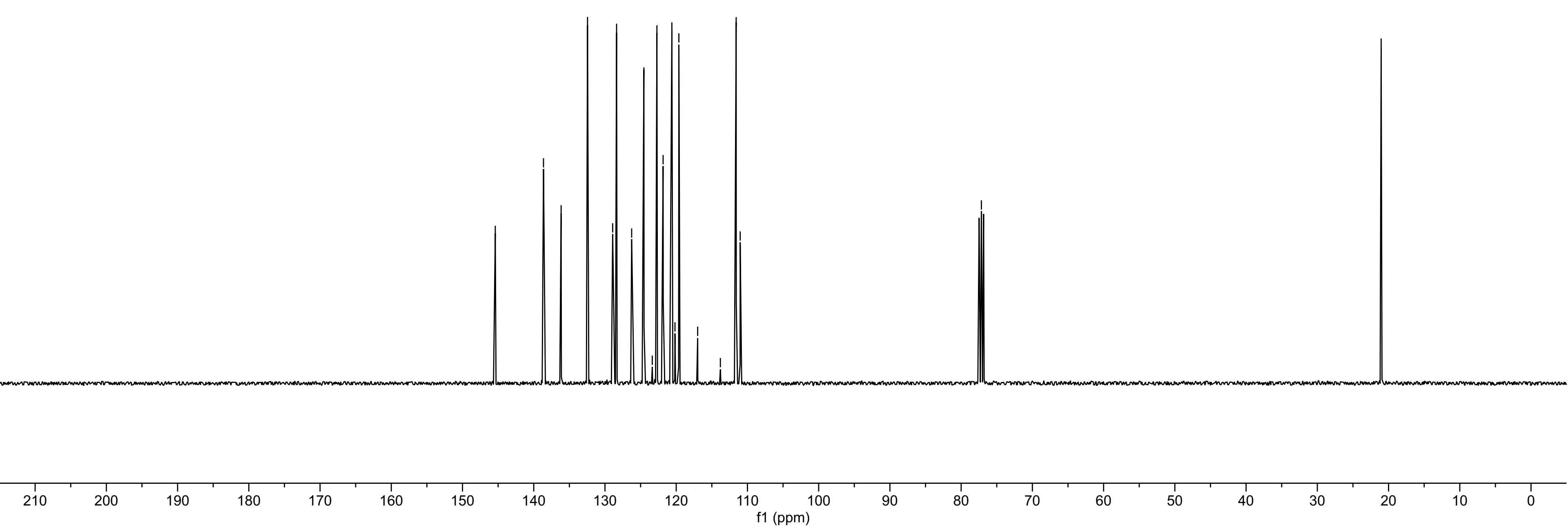

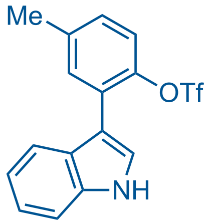

^{19}F NMR of 1v (376 MHz, $CDCl_3$)

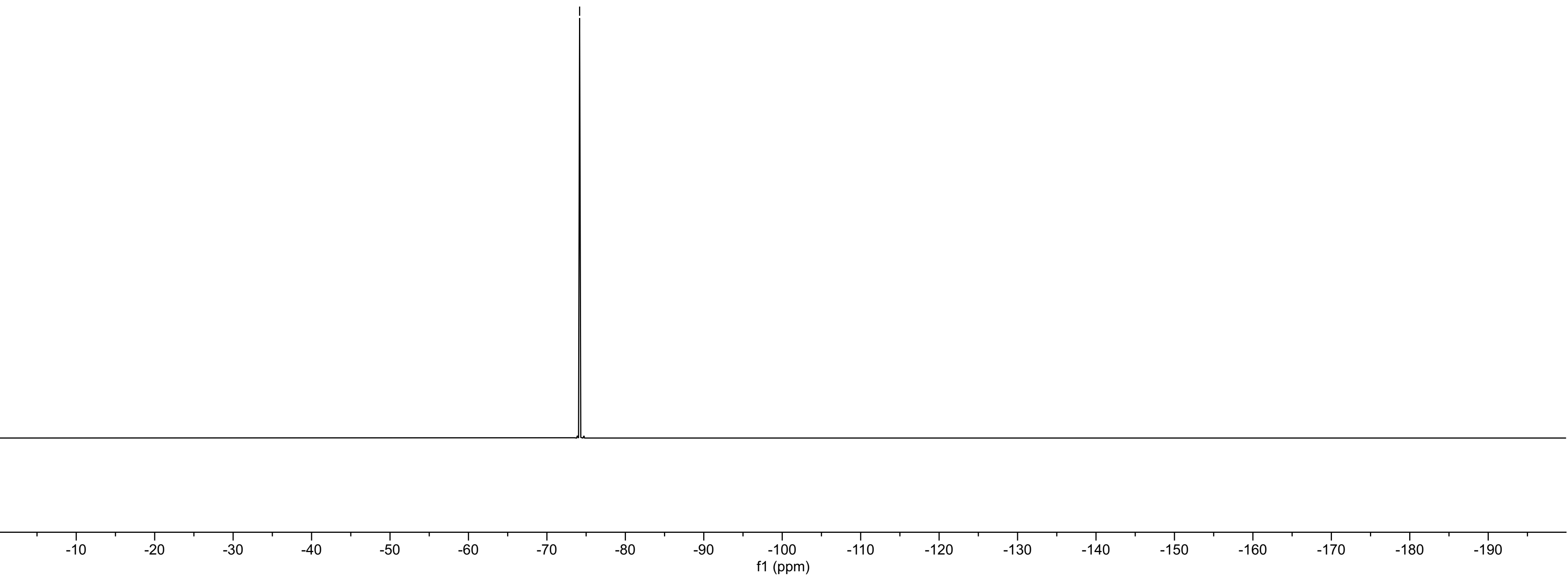

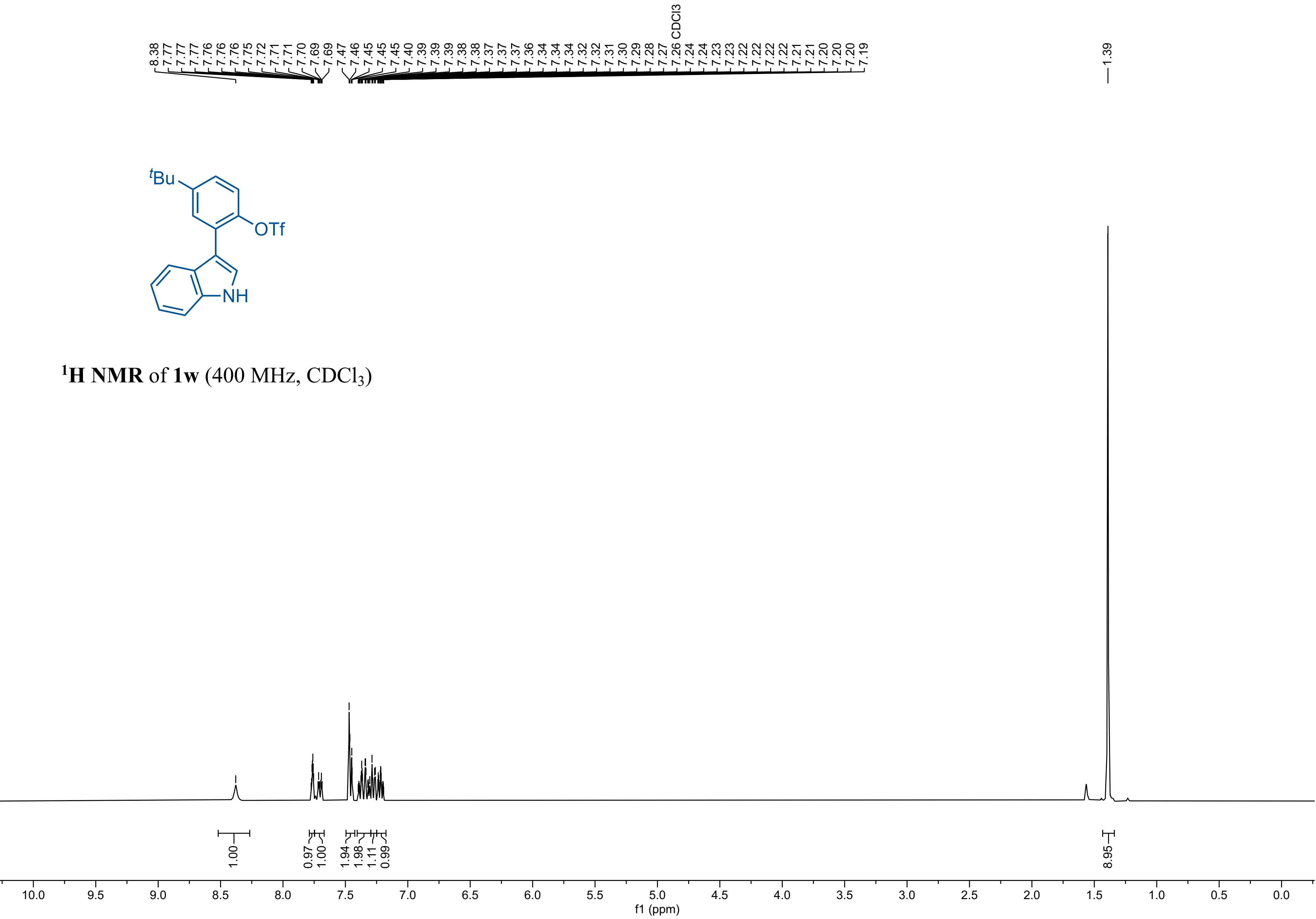

^{1}H NMR of **1w** (400 MHz, $CDCl_3$)

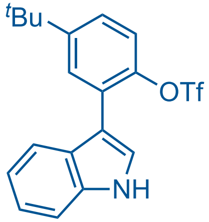

^{13}C NMR of **1w** (101 MHz, $CDCl_3$)

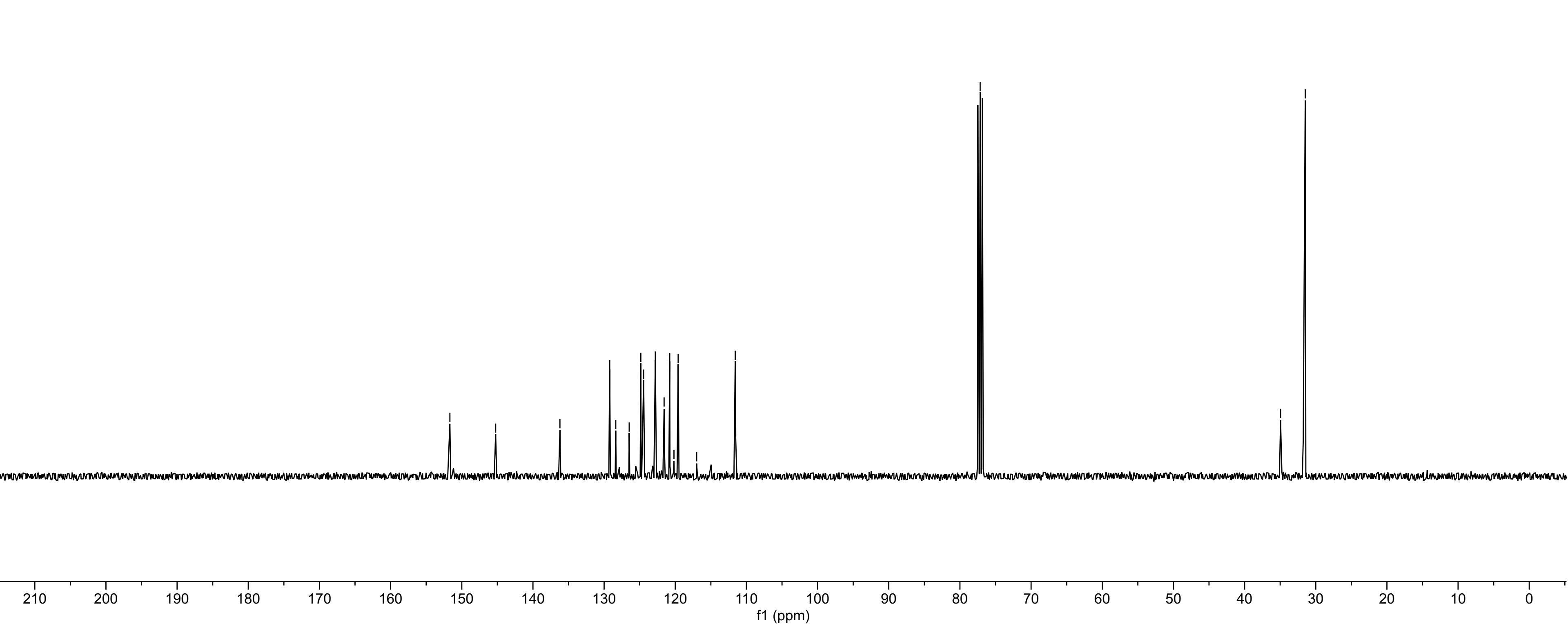

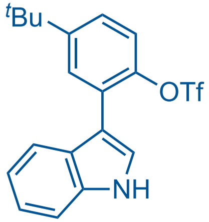

^{19}F NMR of **1w** (376 MHz, $CDCl_3$)

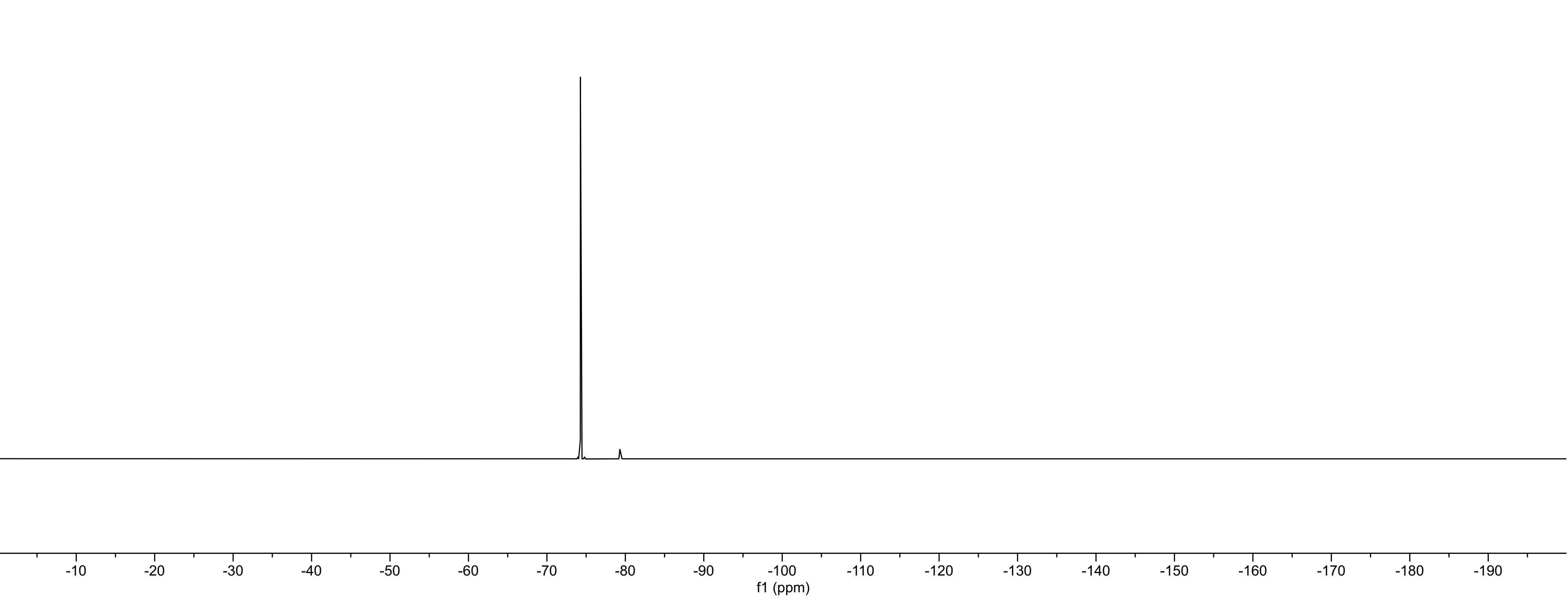

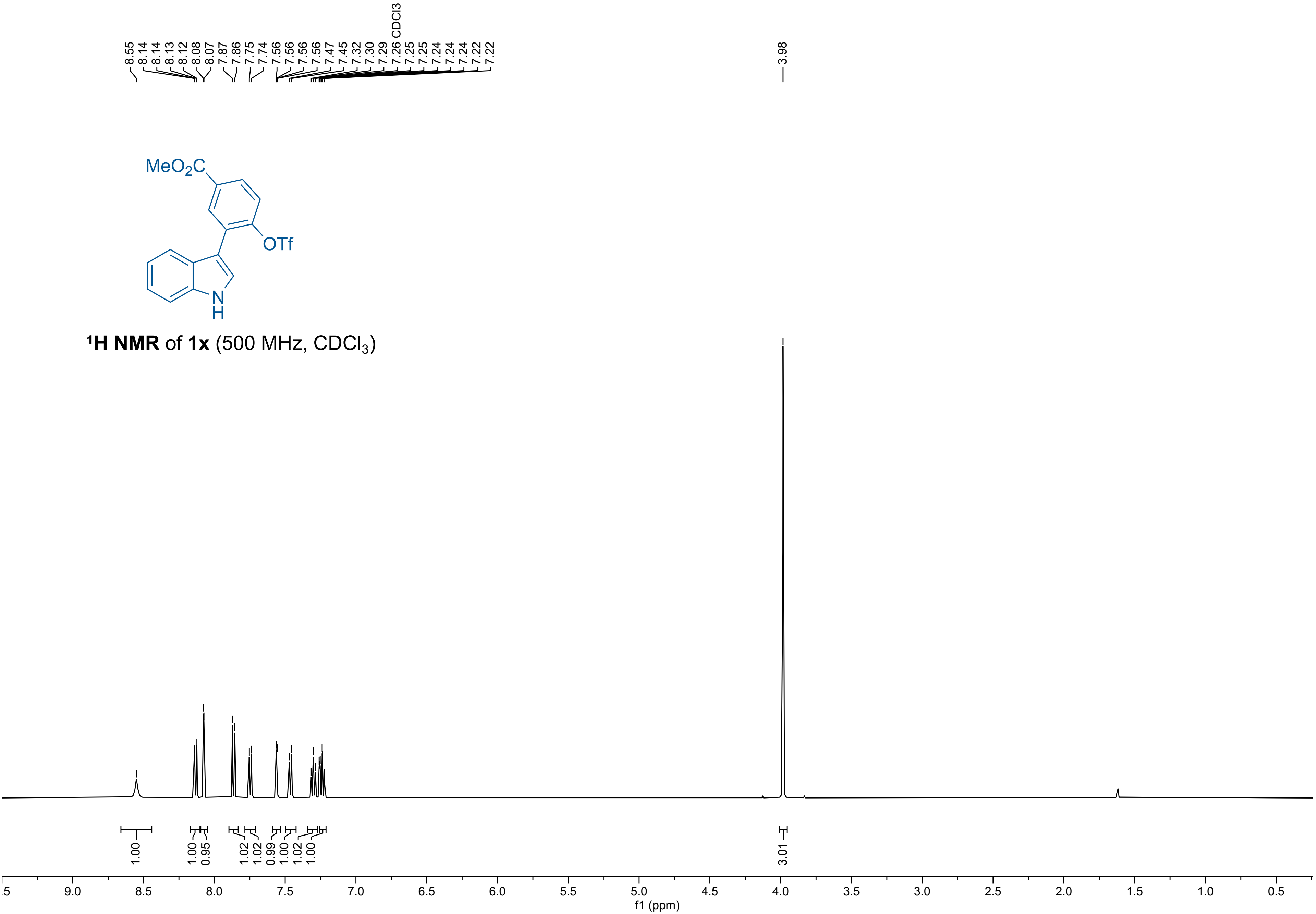

^{1}**H NMR** of **1x** (500 MHz, $CDCl_3$)

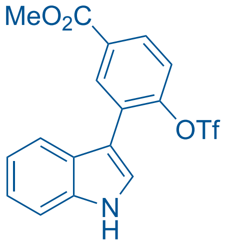

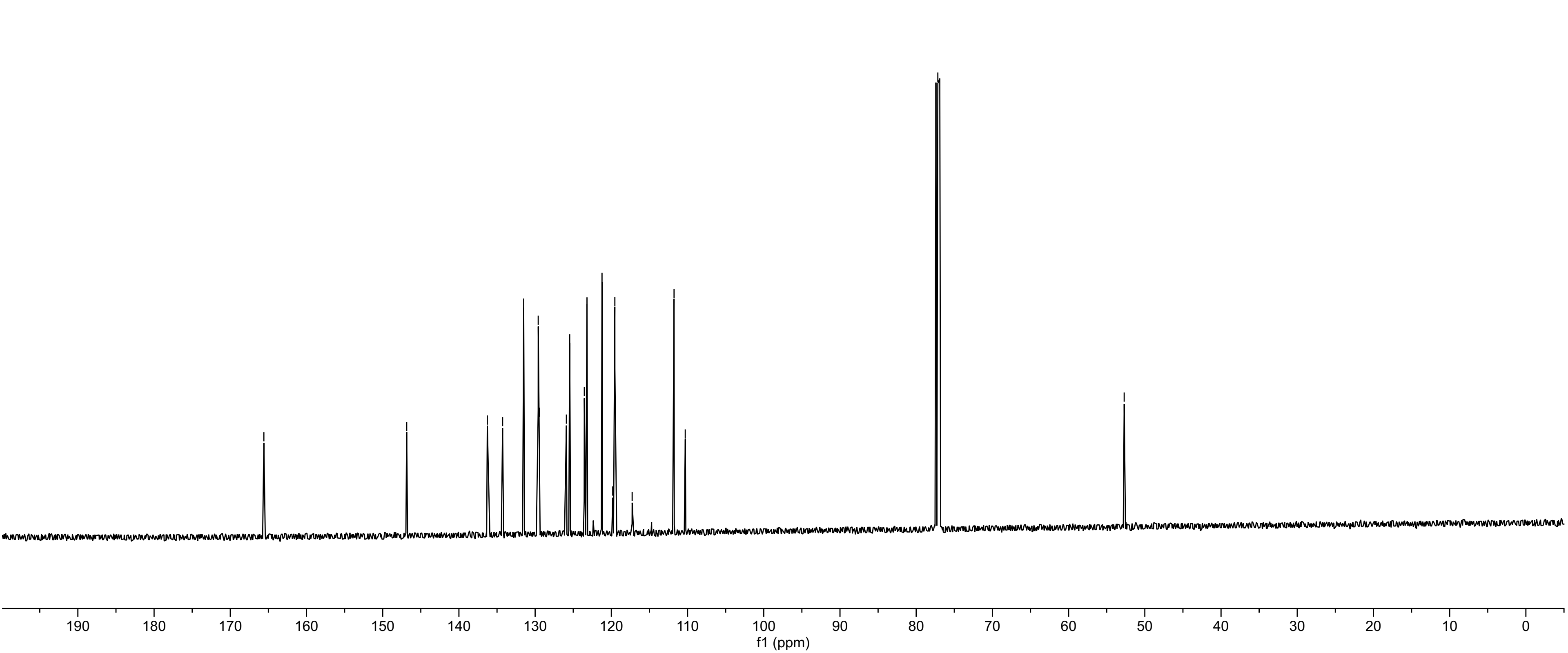

^{13}C NMR of **1x** (126 MHz, $CDCl_3$)

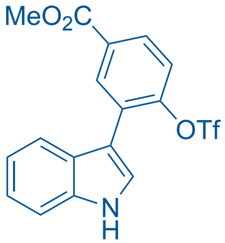

^{19}**F NMR** of **1x** (471 MHz, $CDCl_3$)

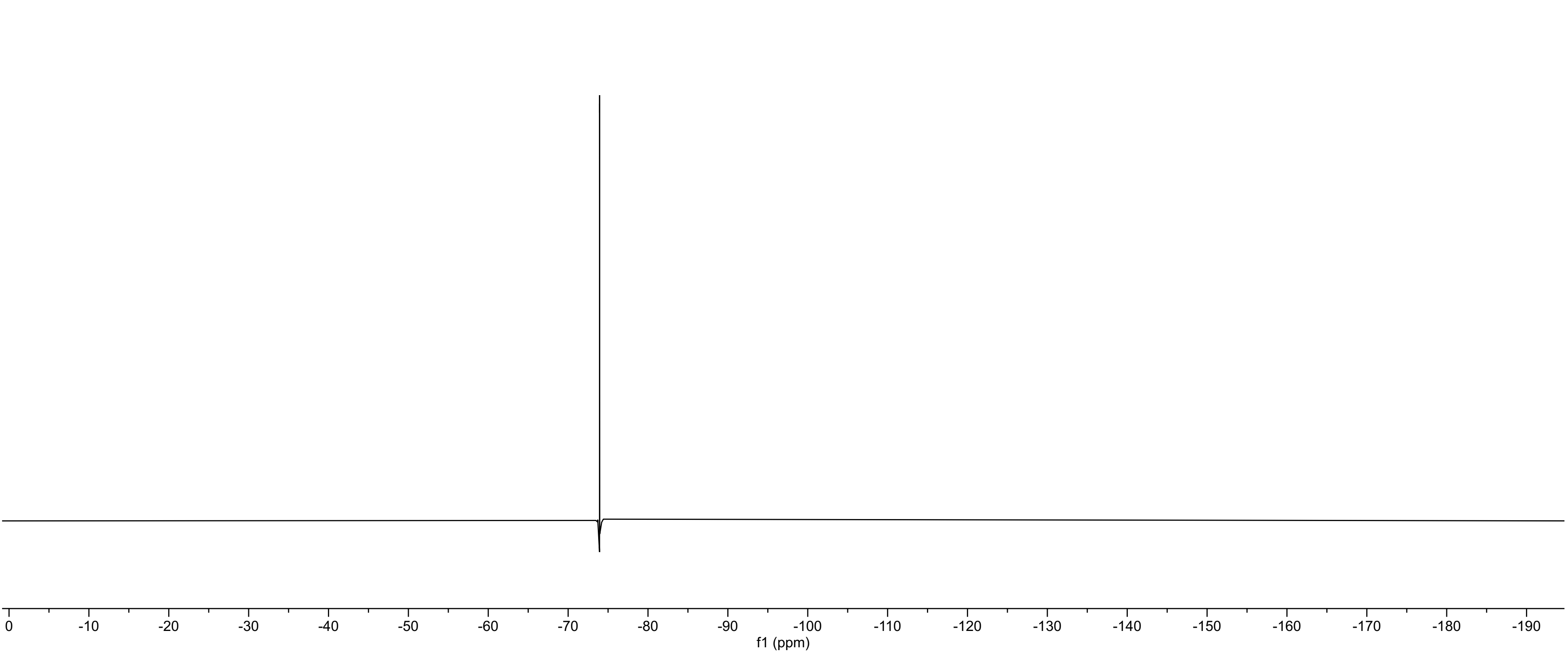

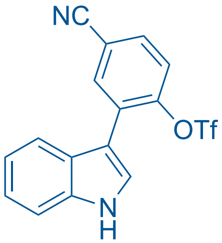

¹H NMR of **1y** (500 MHz, $CDCl_3$)

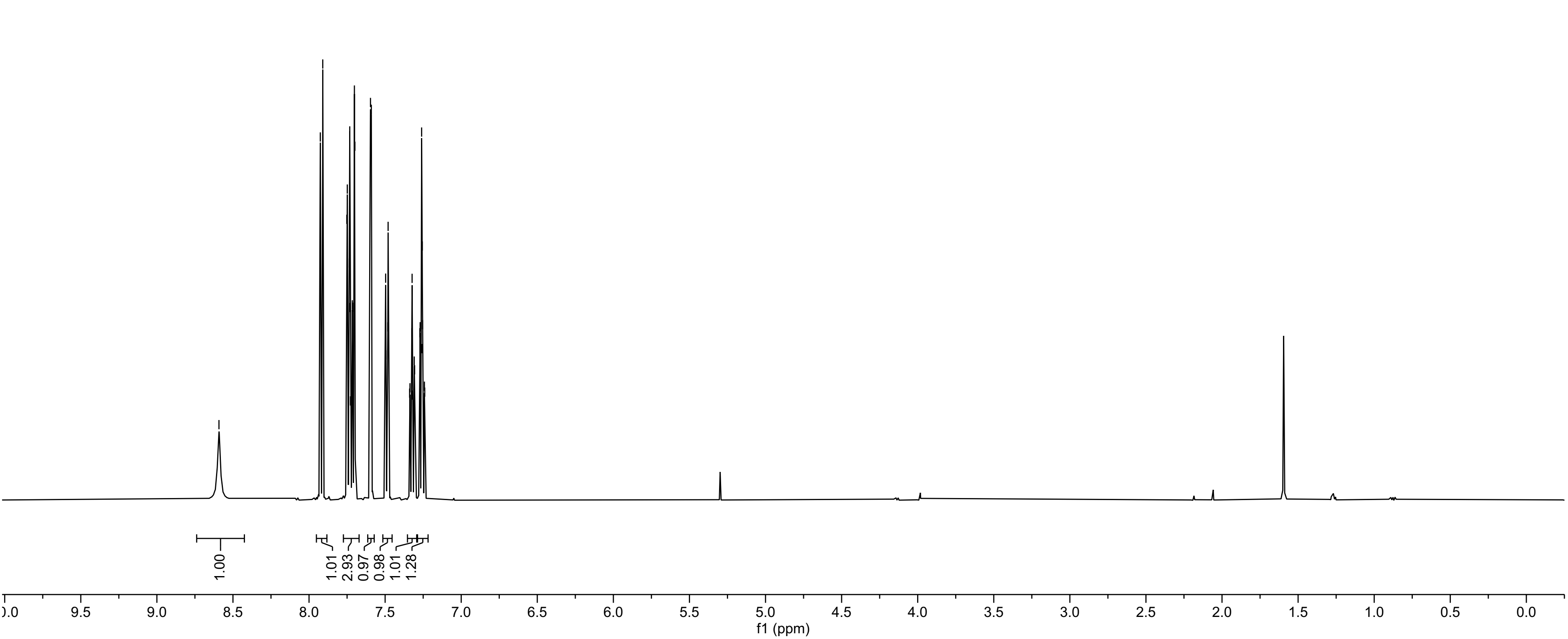

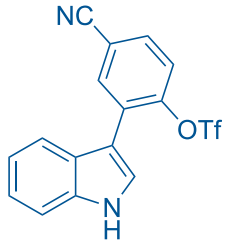

^{13}C NMR of **1y** (126 MHz, $CDCl_3$)

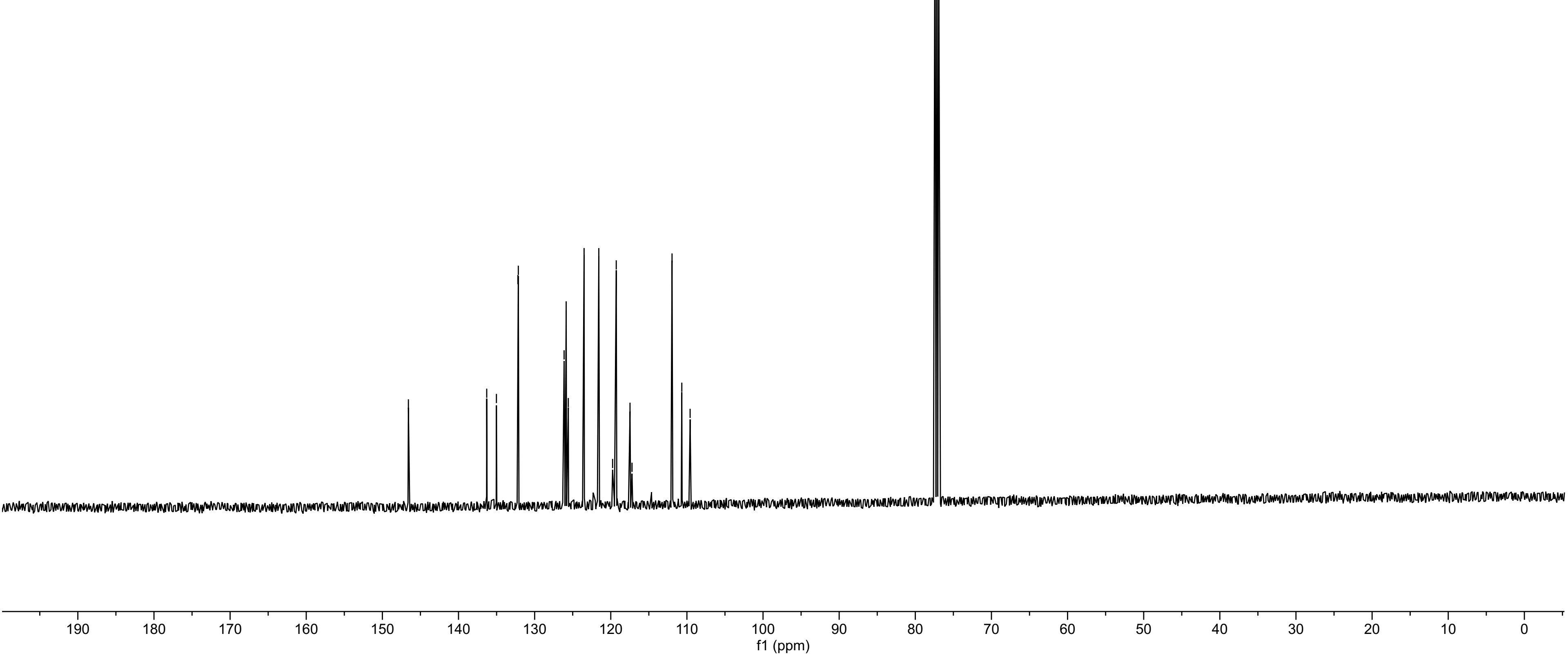

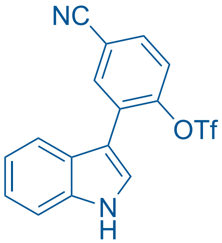

^{19}F NMR of **1y** (471 MHz, $CDCl_3$)

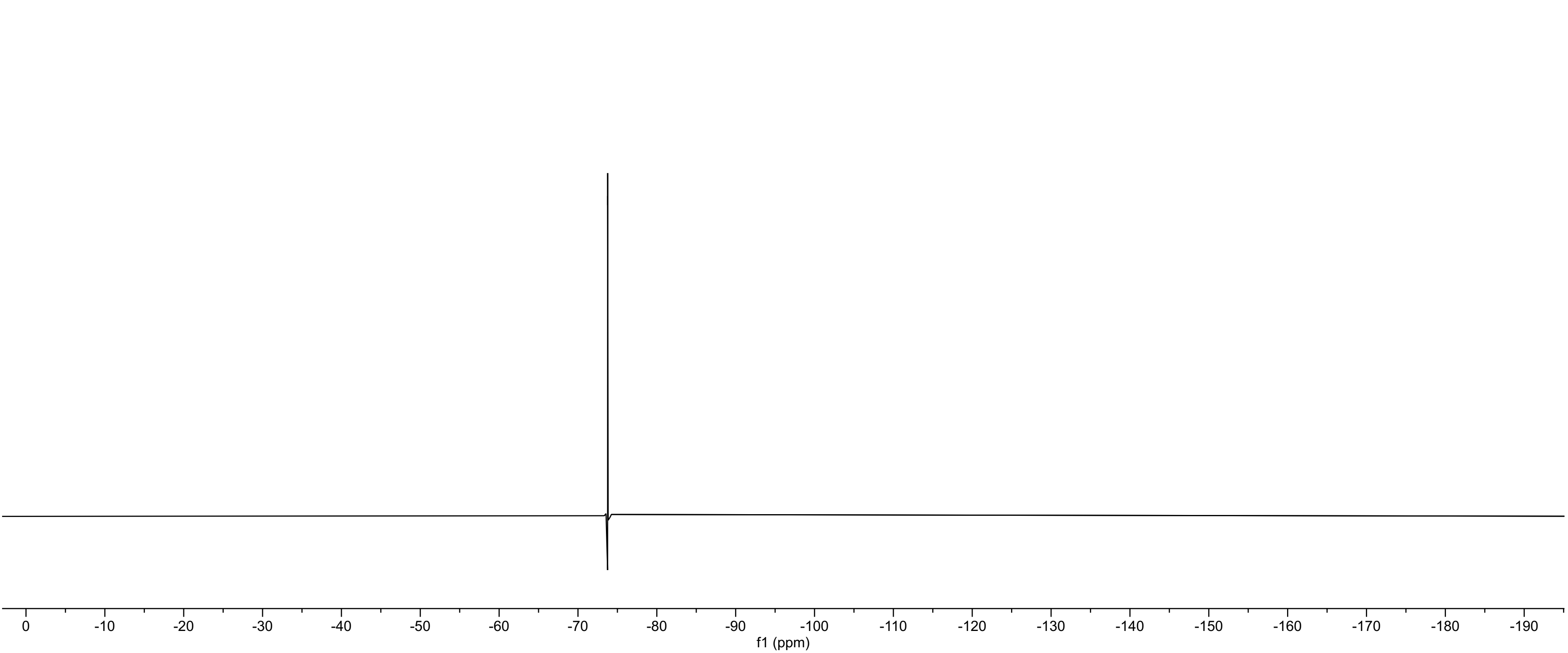

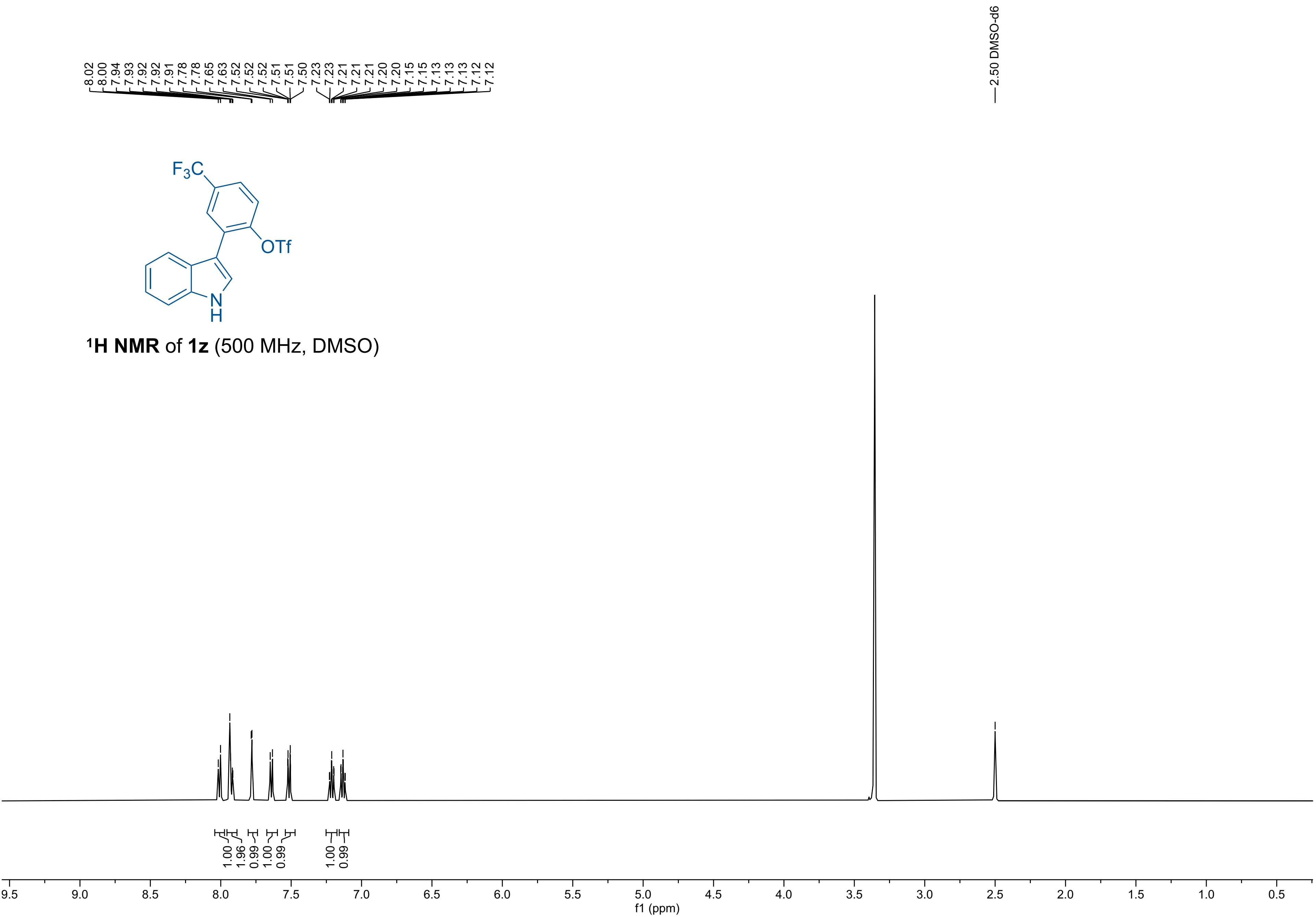

^{1}H NMR of **1z** (500 MHz, DMSO)

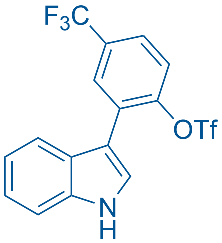

^{13}C NMR of 1z (101 MHz, DMSO)

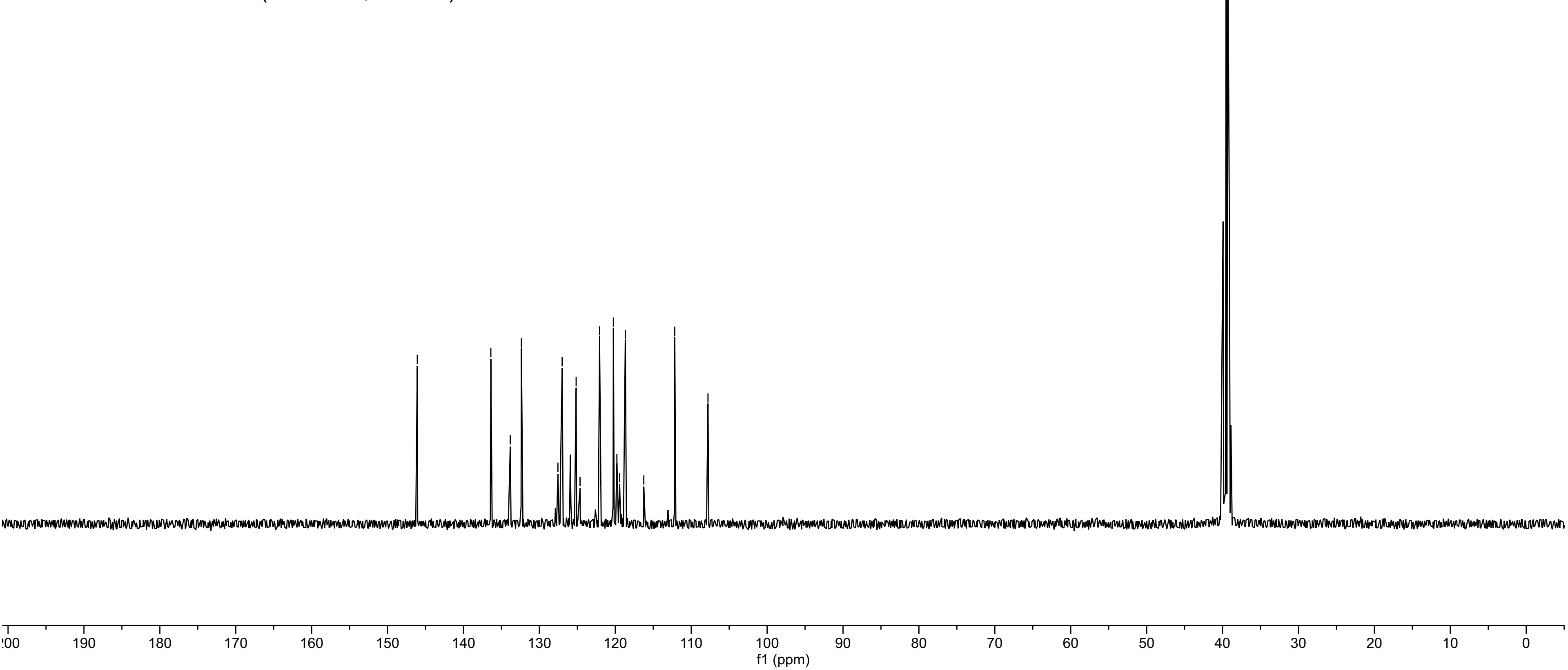

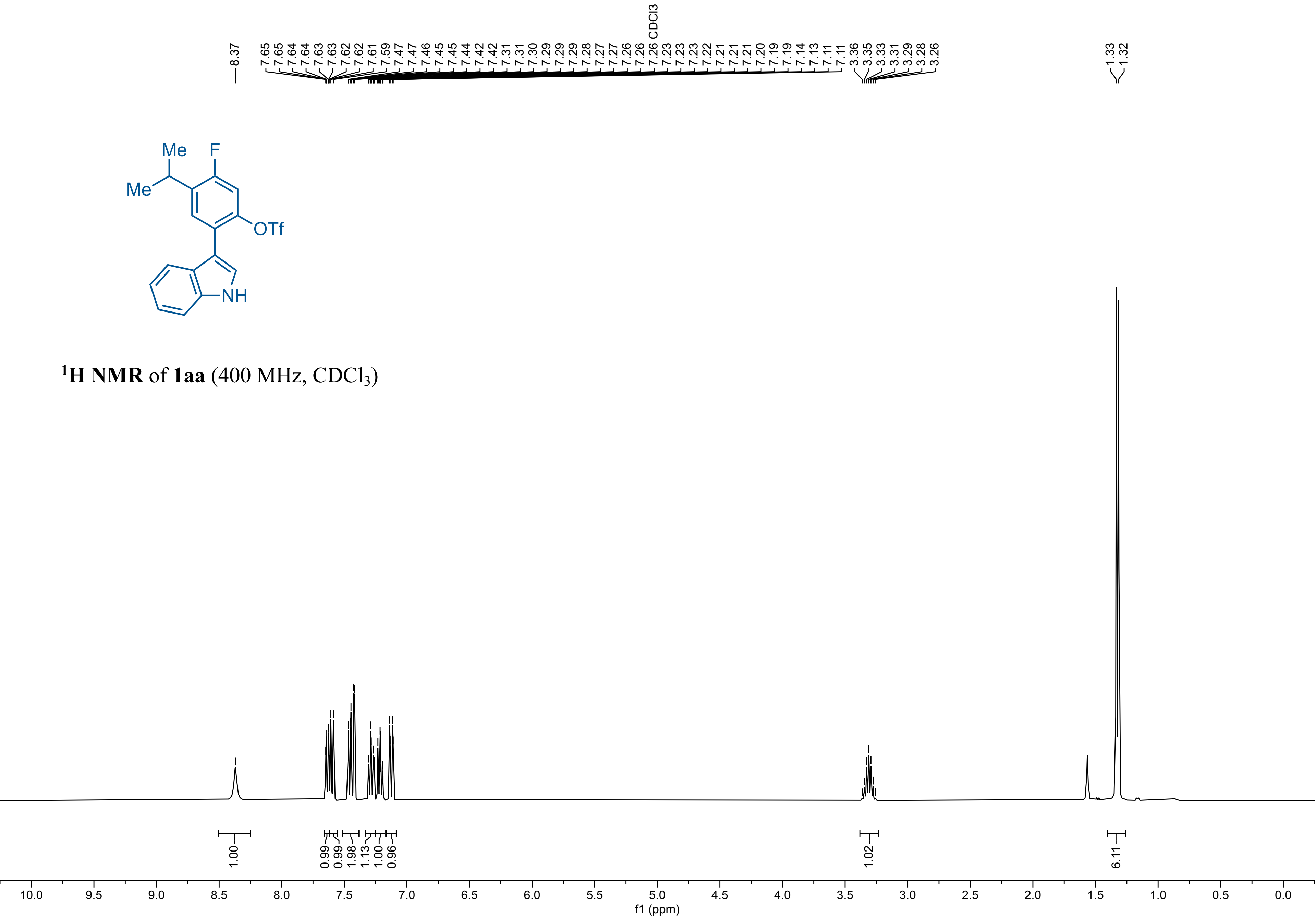

^{1}H NMR of **1aa** (400 MHz, $CDCl_3$)

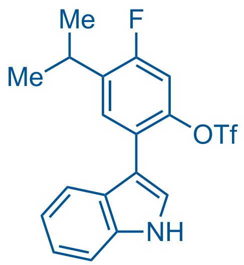

^{13}C NMR of **1aa** (101 MHz, $CDCl_3$)

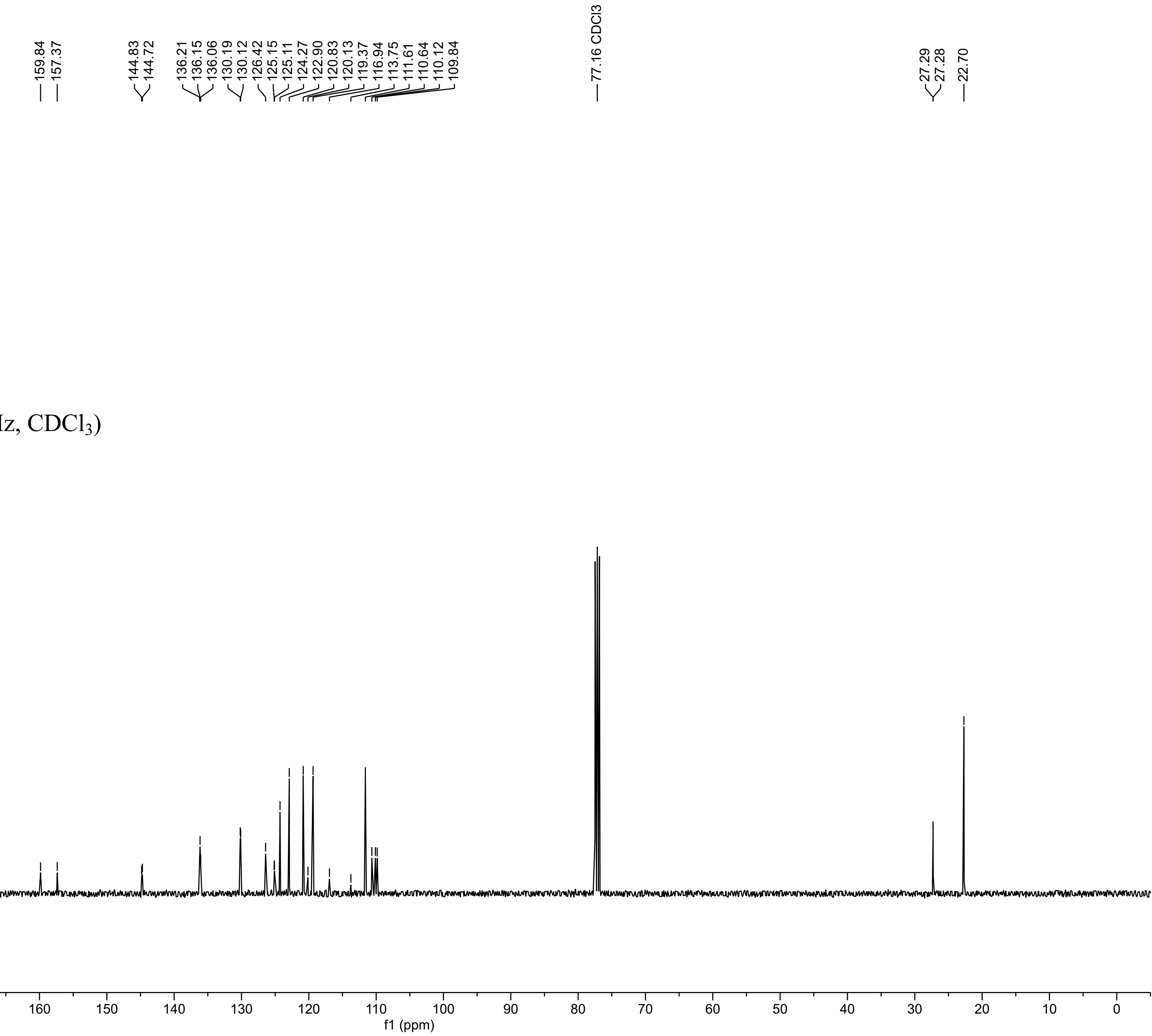

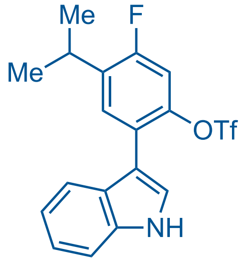

^{19}F NMR of **1aa** (376 MHz, $CDCl_3$)

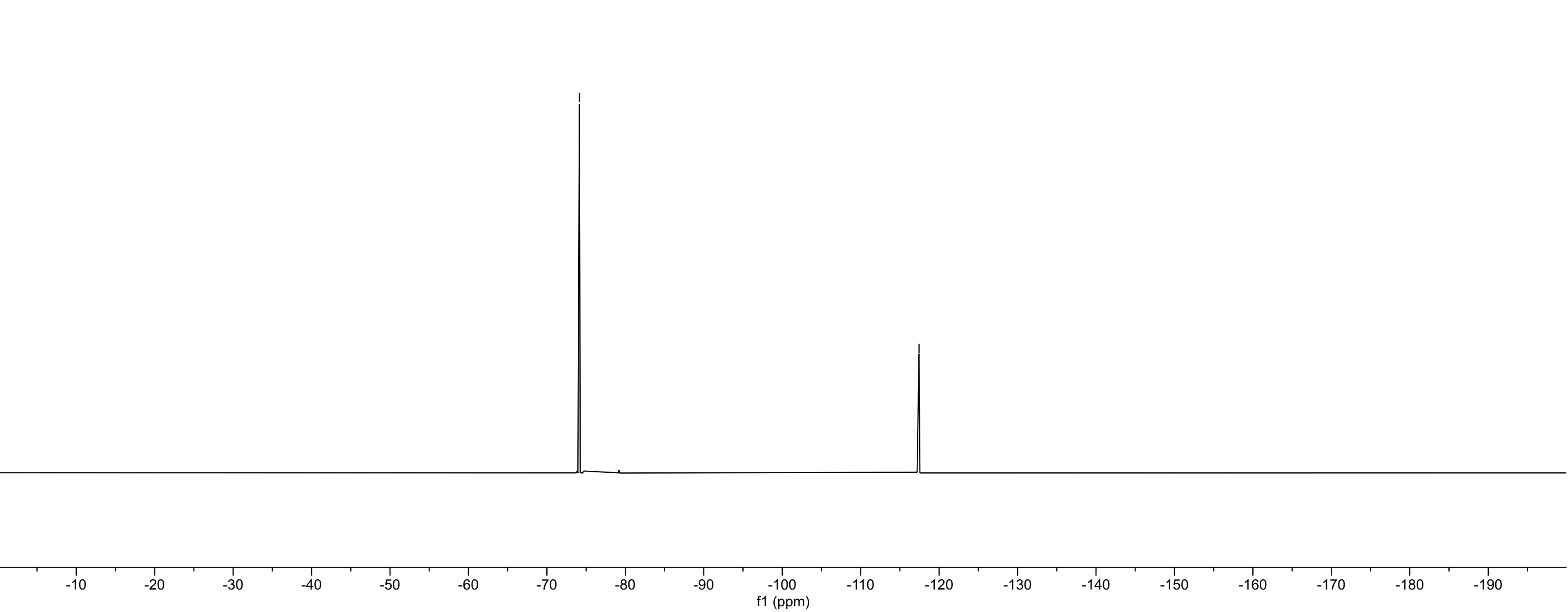

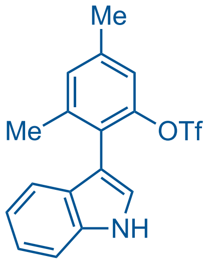

^{1}H NMR of **1ab** (400 MHz, $CDCl_3$)

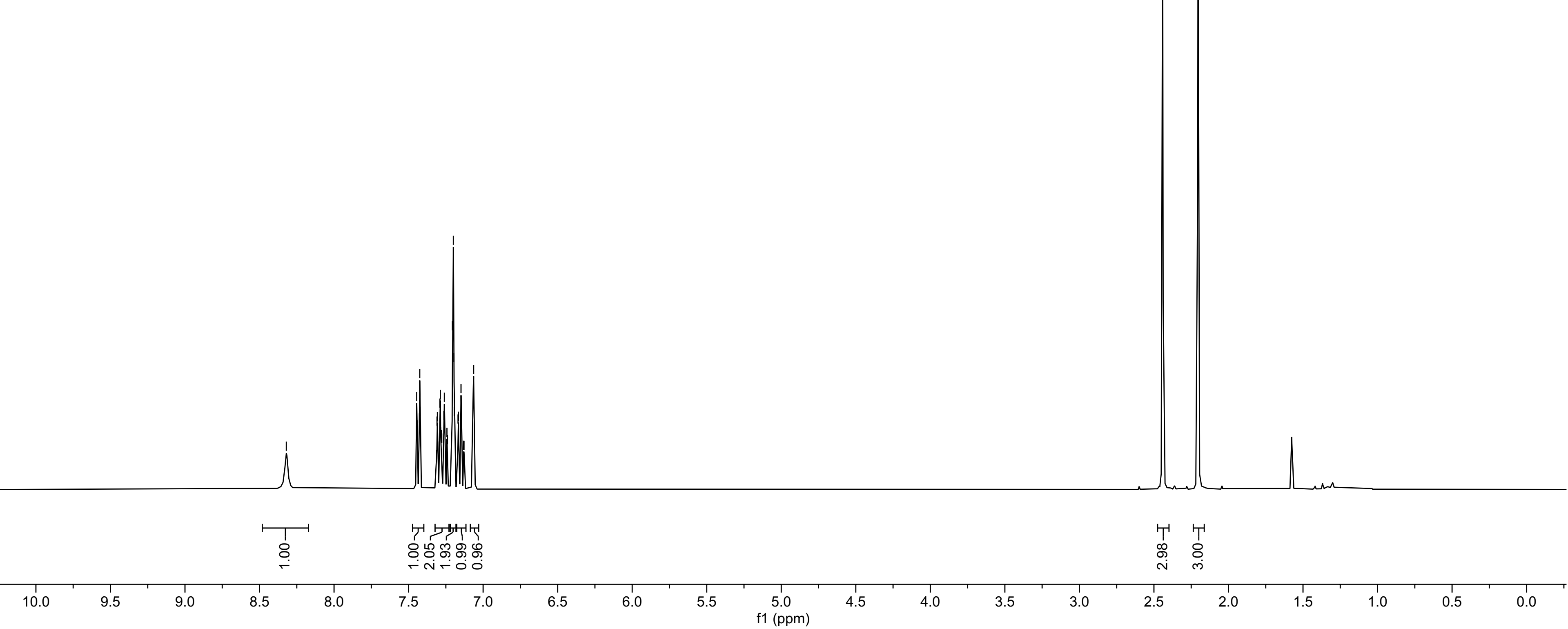

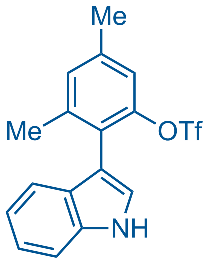

^{13}C NMR of **1ab** (101 MHz, $CDCl_3$)

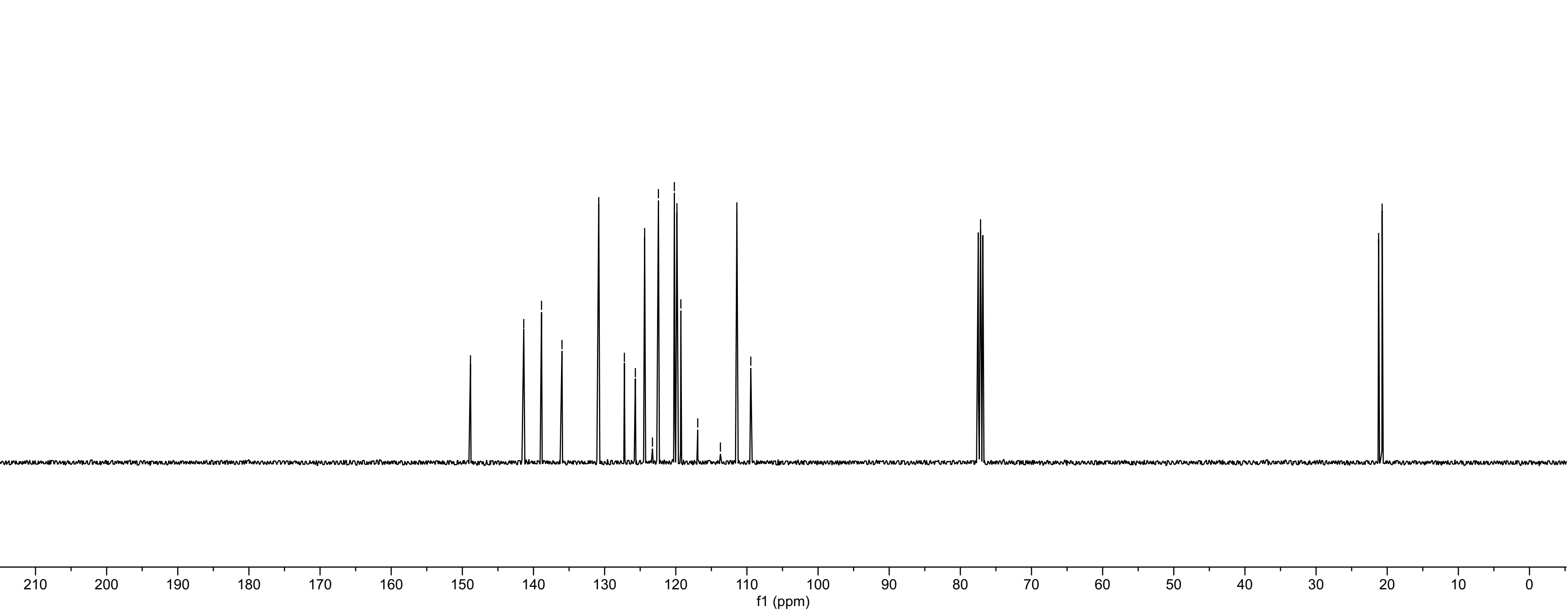

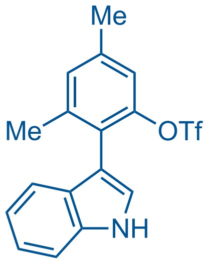

^{19}F NMR of **1ab** (376 MHz, $CDCl_3$)

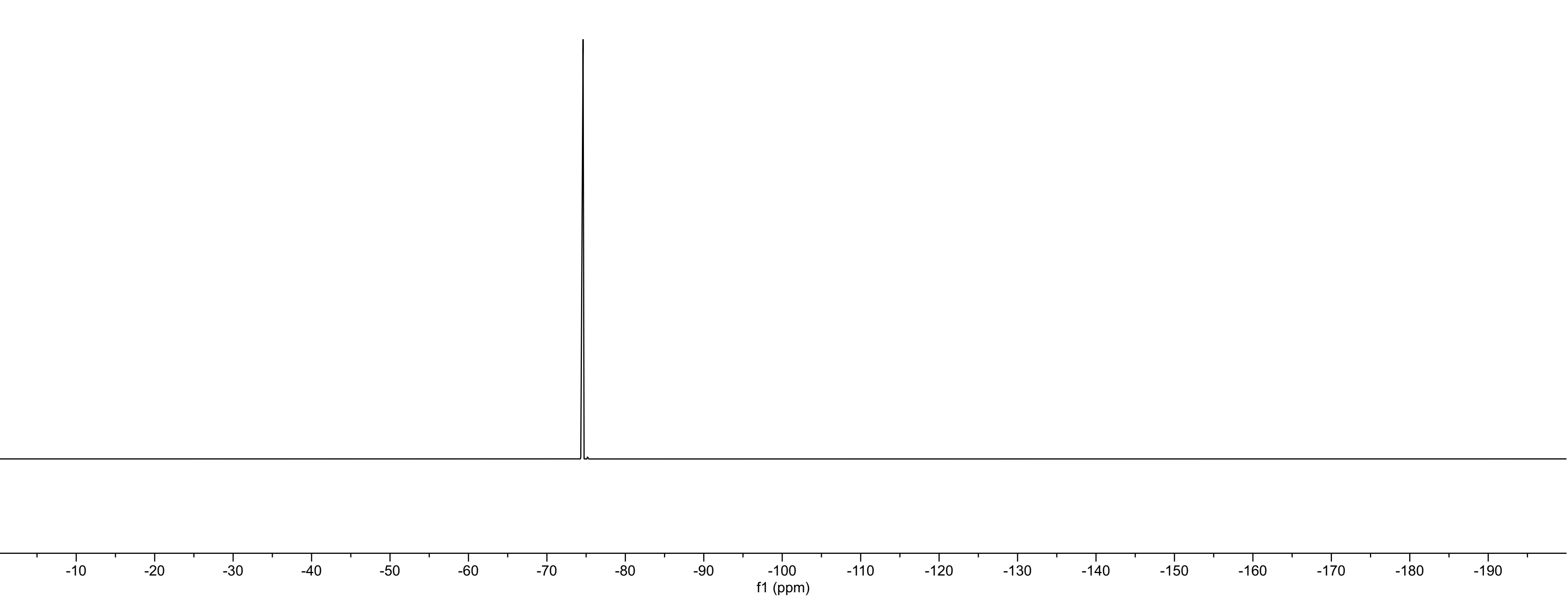

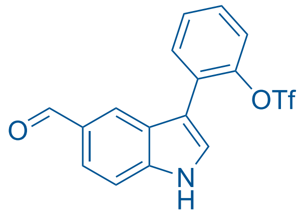

^{1}**H NMR** of (500 MHz, $CDCl_3$)

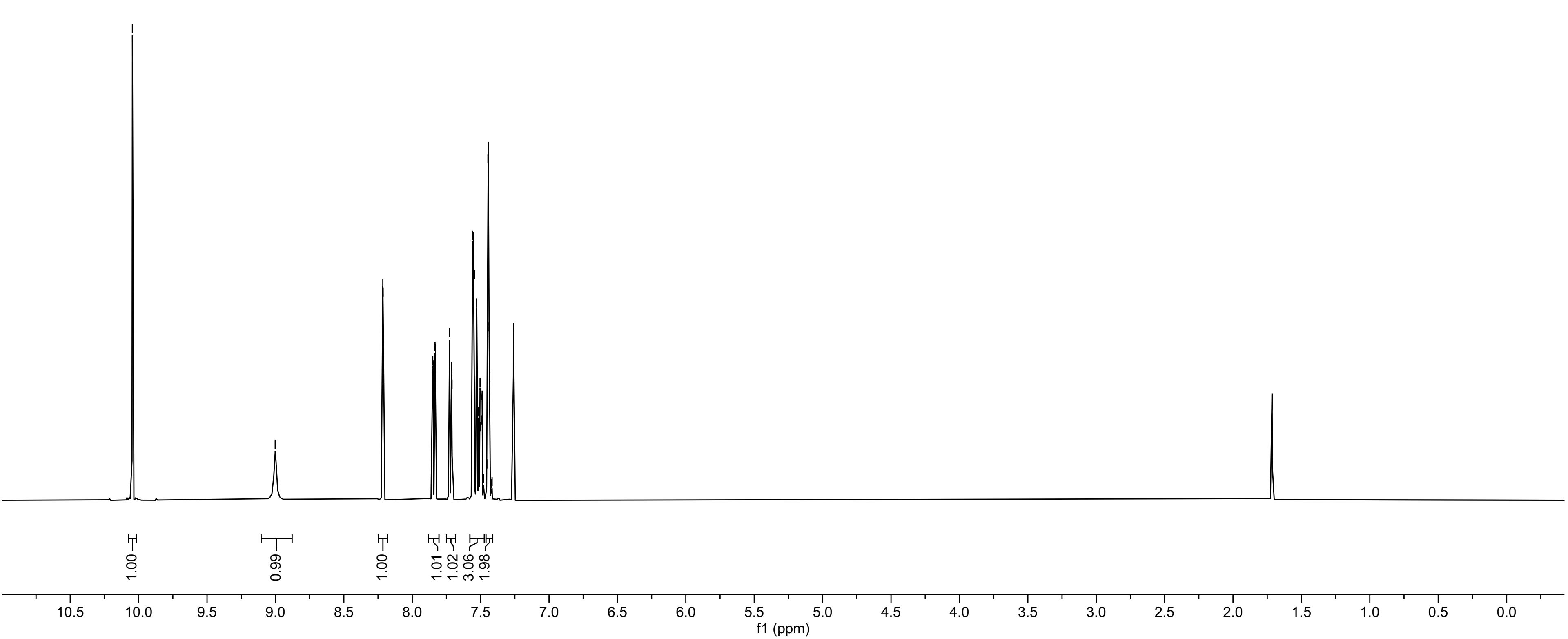

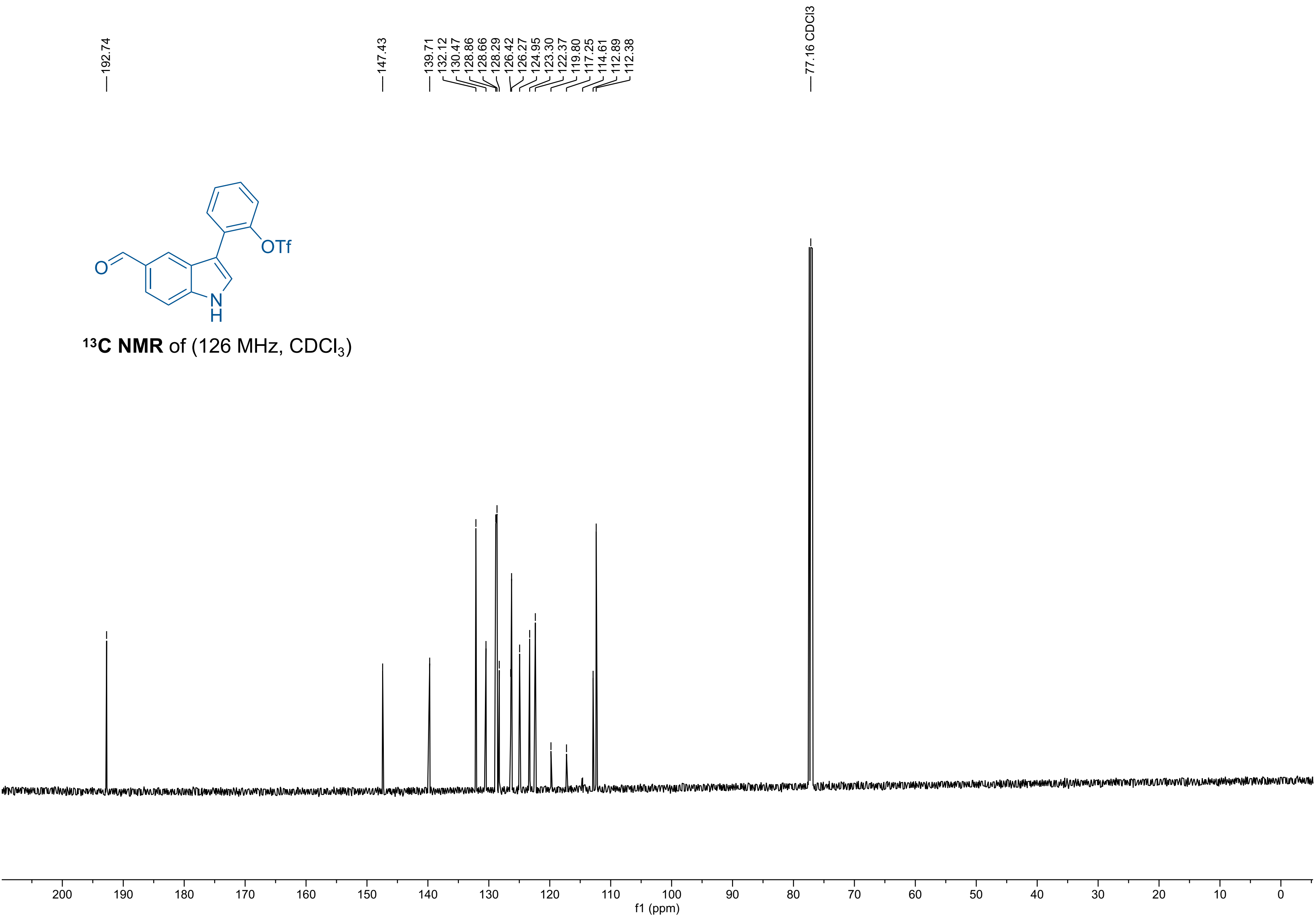

13C NMR of (126 MHz, $CDCl_3$)

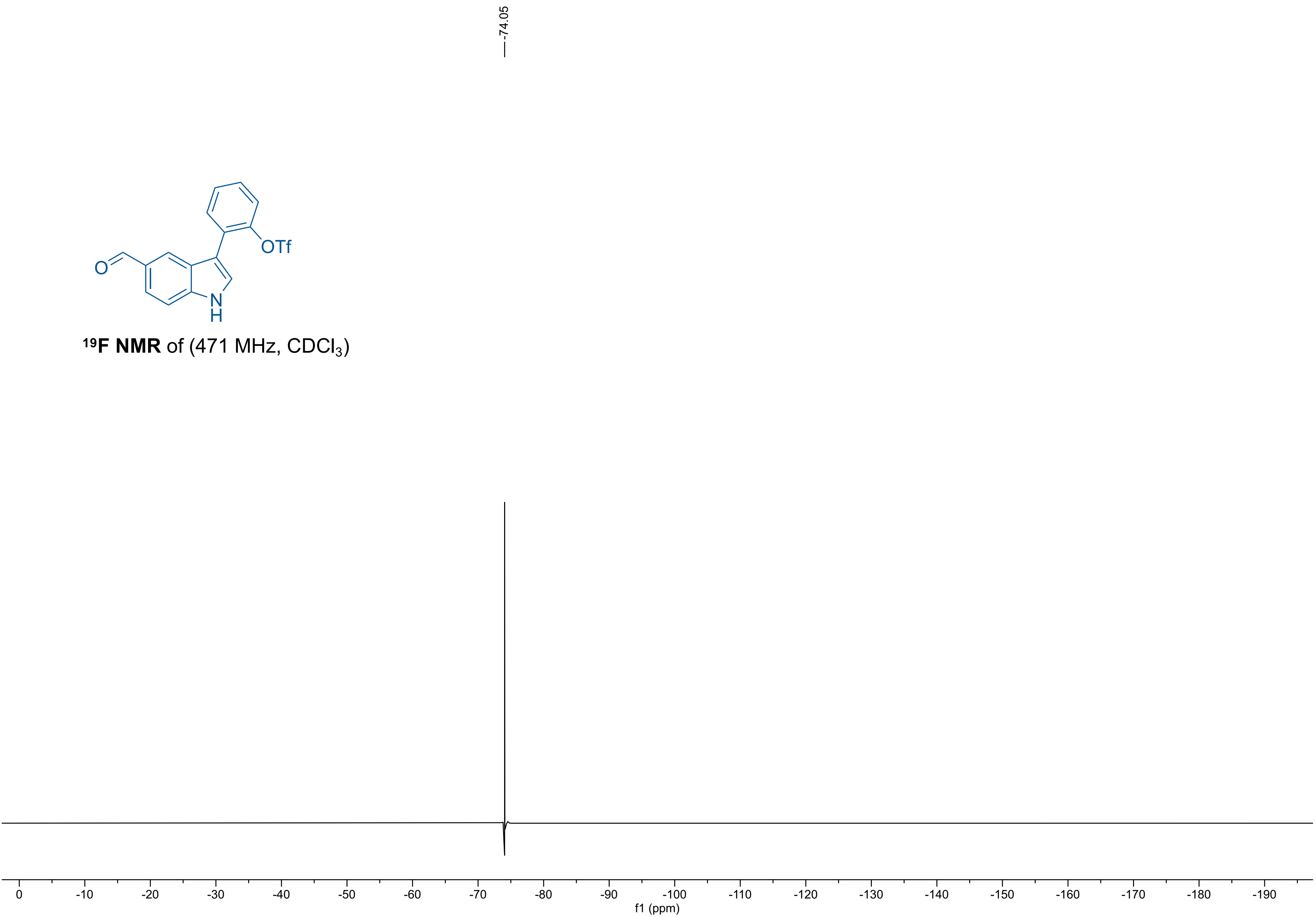

^{19}F NMR of (471 MHz, $CDCl_3$)

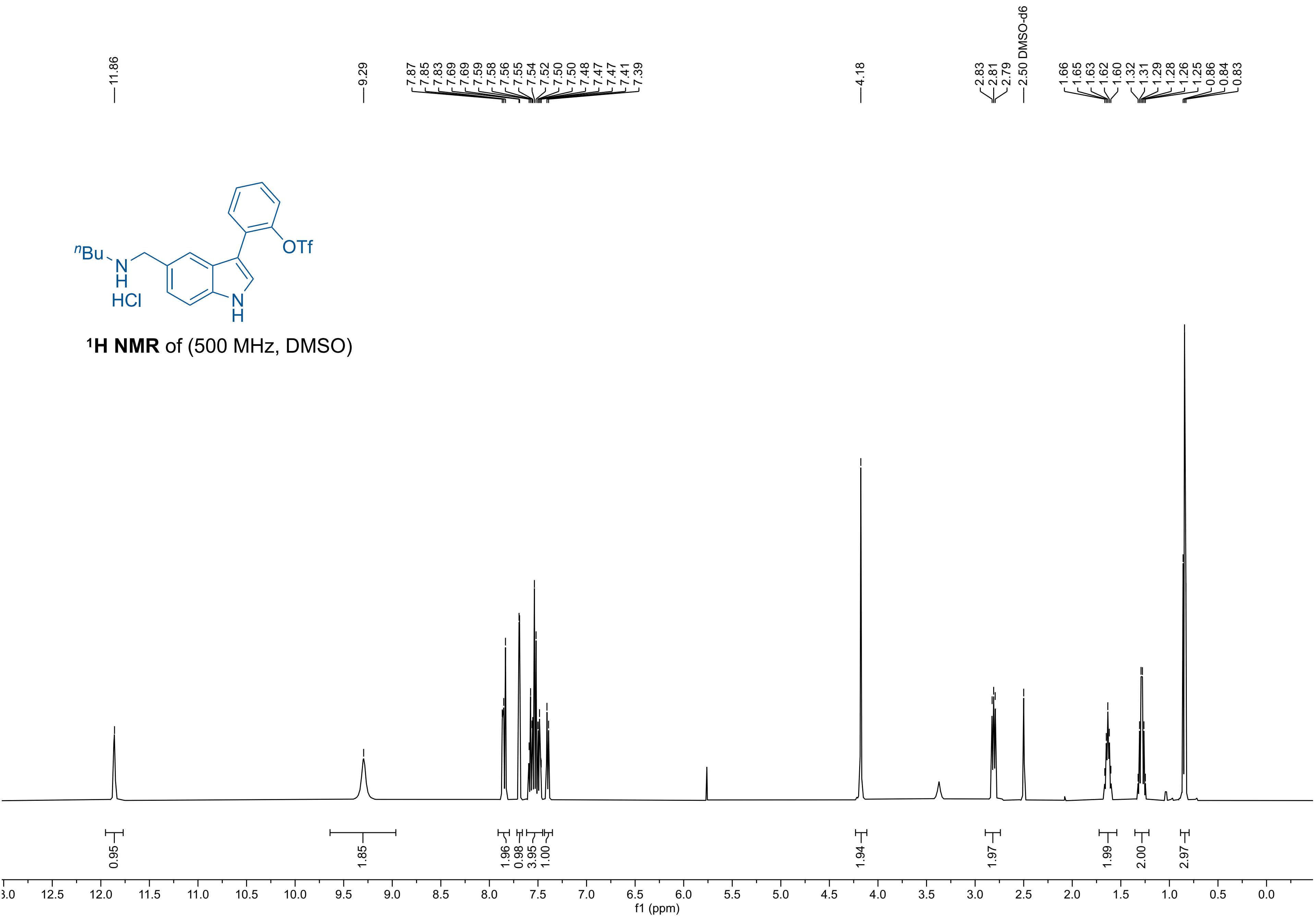

^{1}H NMR of (500 MHz, DMSO)

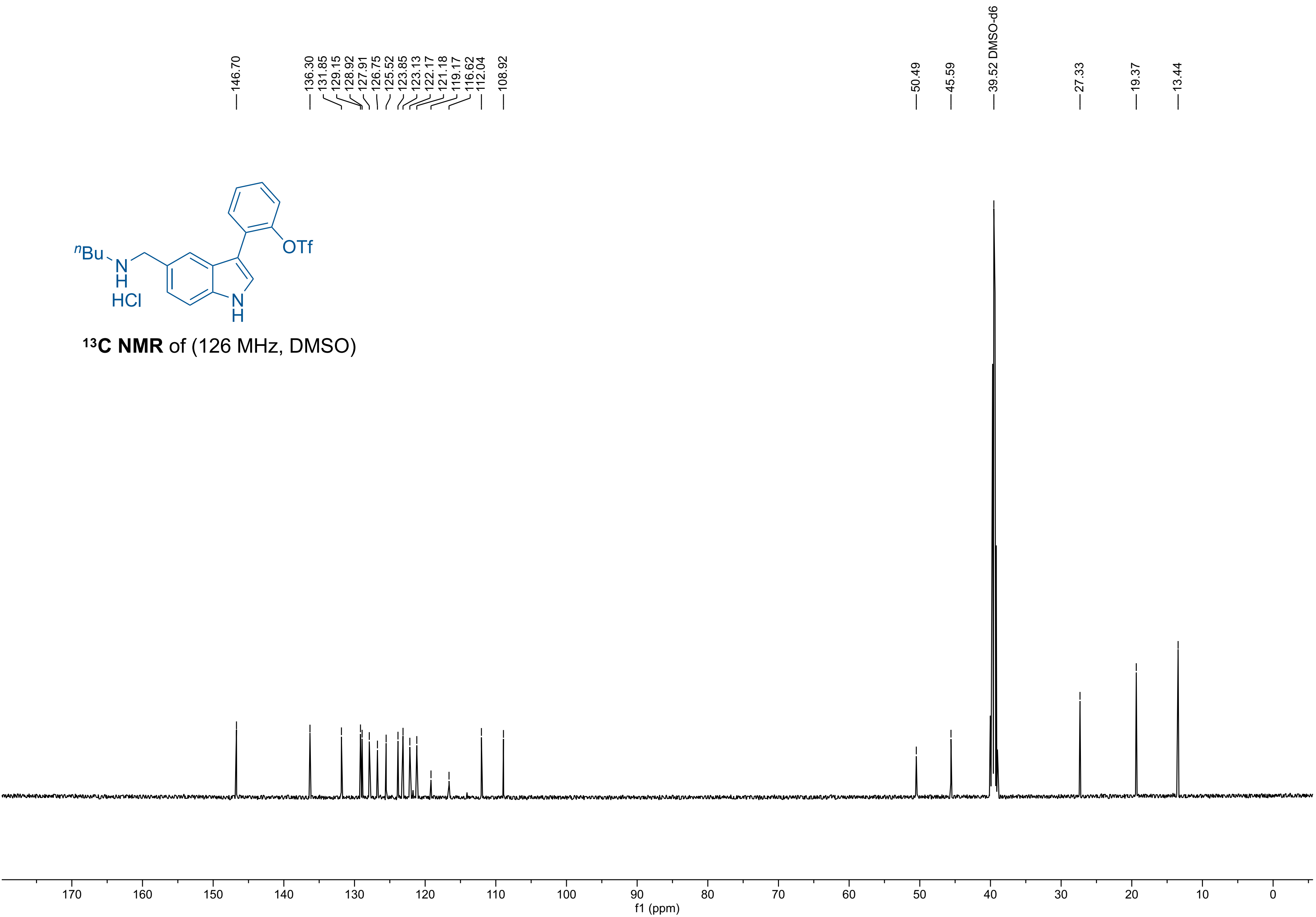
146.70
136.30
131.85
129.15
128.92
127.91
126.75
125.52
123.85
123.13
122.17
121.18
119.17
116.62
112.04
108.92
50.49
45.59
39.52 DMSO-d6
27.33
19.37
13.44
nBu
N
H
HCl
N
H
OTf
13C NMR of (126 MHz, DMSO)
170
160
150
140
130
120
110
100
90
80
70
60
50
40
30
20
10
0
f1 (ppm)

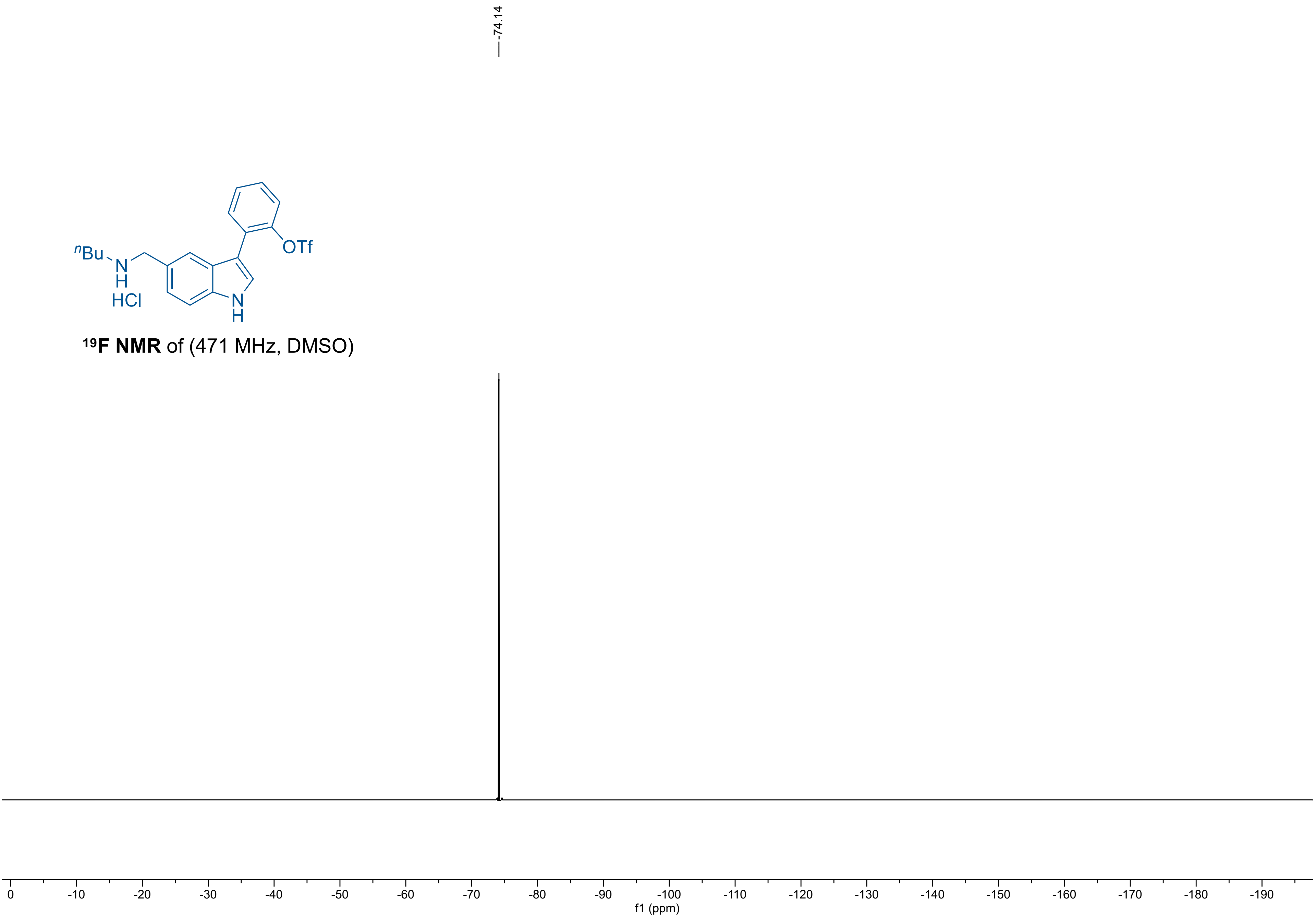

^{19}F NMR of (471 MHz, DMSO)

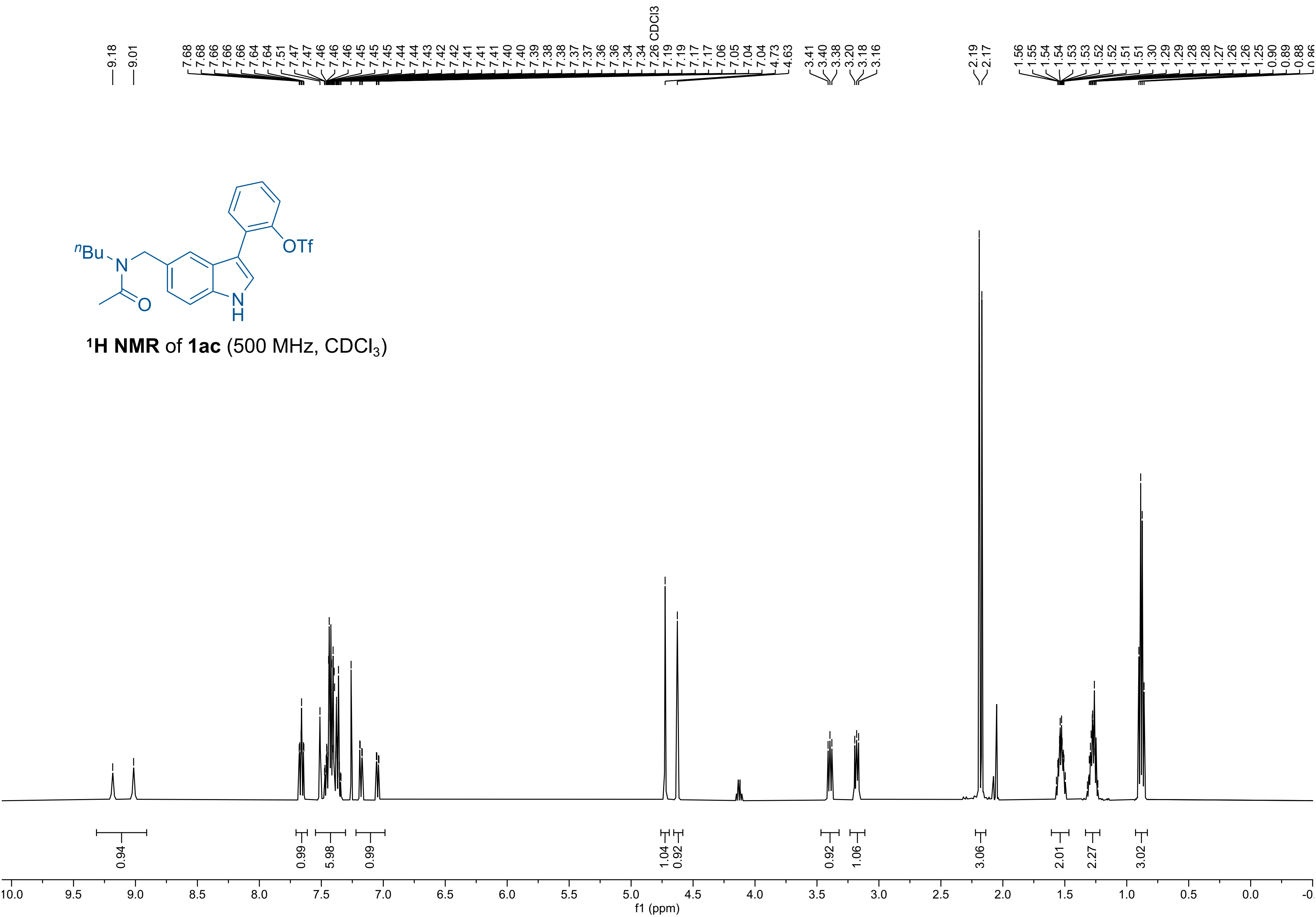

^{1}H NMR of **1ac** (500 MHz, $CDCl_3$)

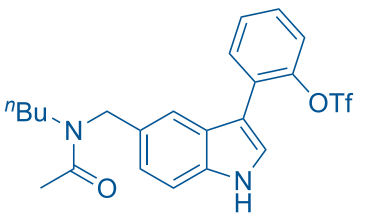

^{13}C NMR of **1ac**(126 MHz, $CDCl_3$)

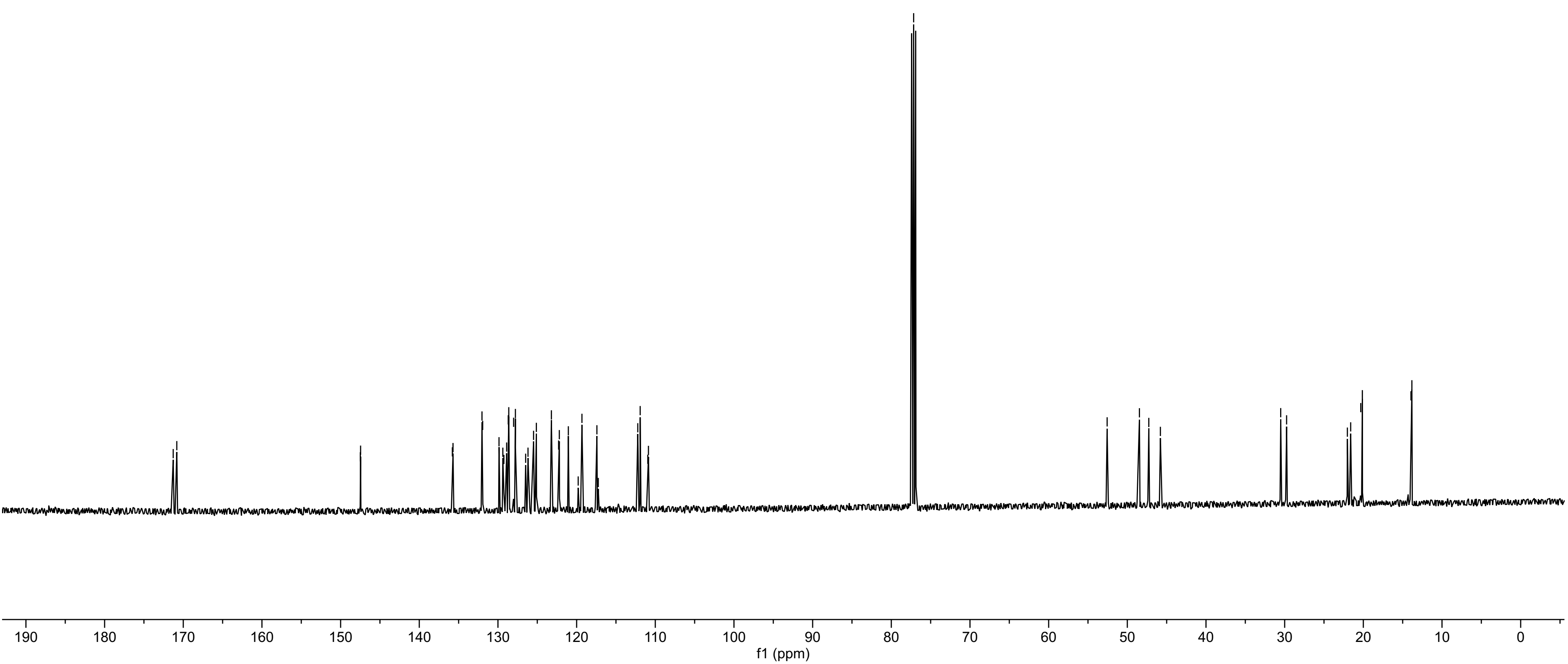

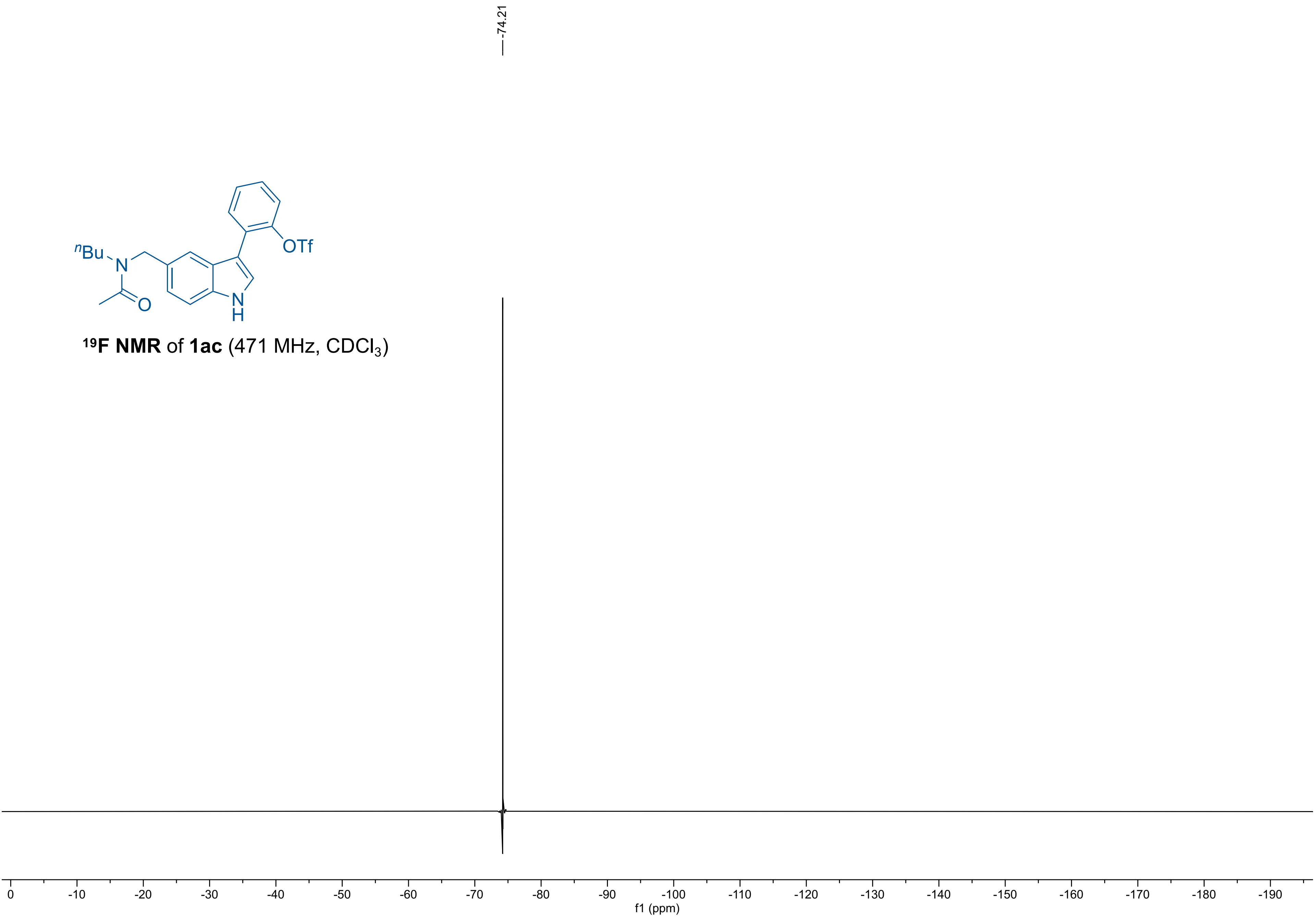

^{19}F NMR of **1ac** (471 MHz, $CDCl_3$)

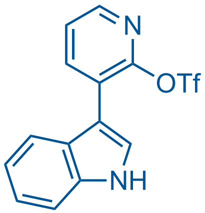

^{1}H NMR of **1ad** (400 MHz, $CDCl_3$)

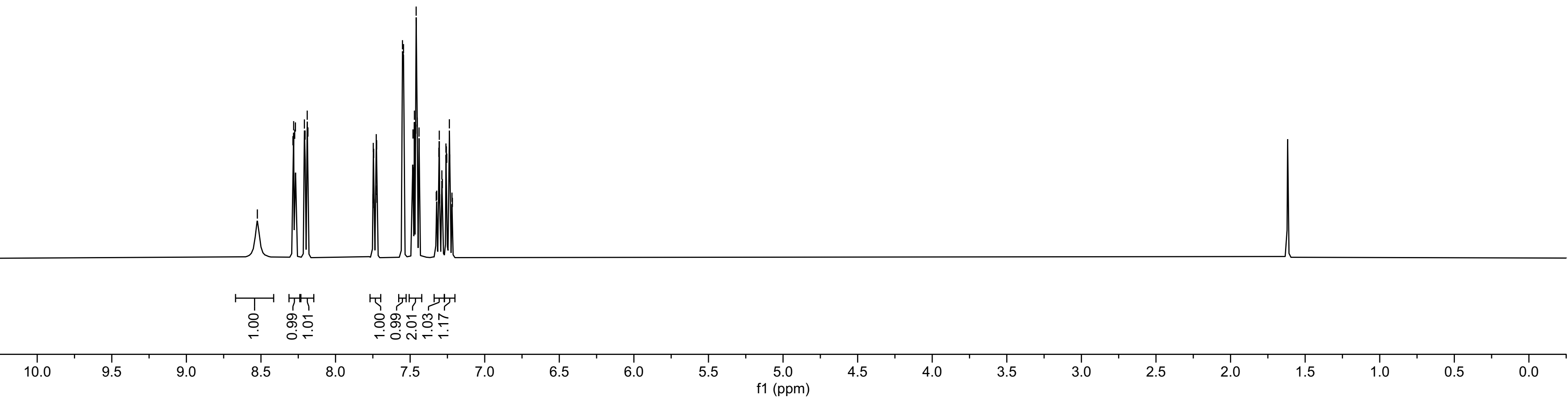

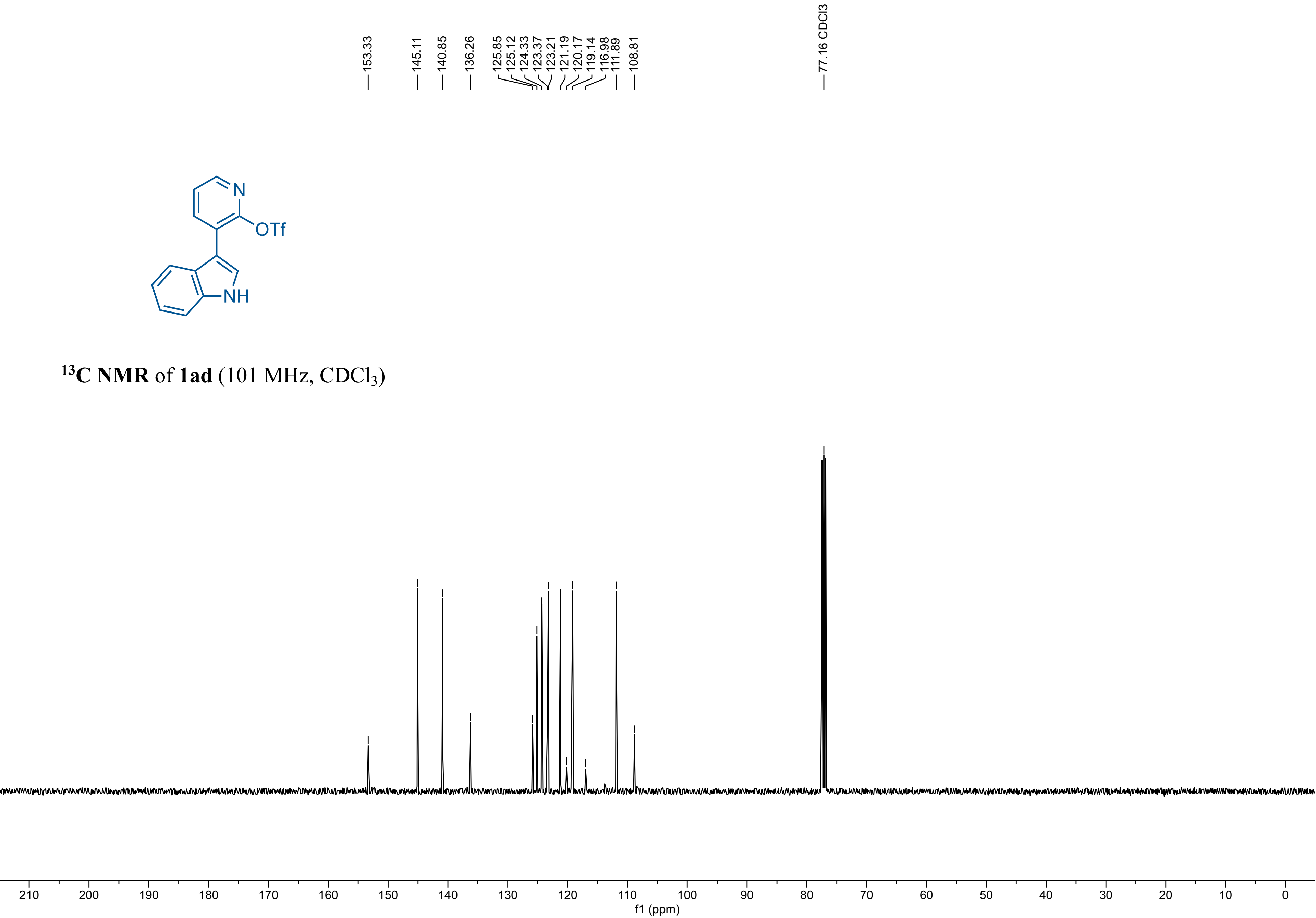

^{13}C NMR of **1ad** (101 MHz, $CDCl_3$)

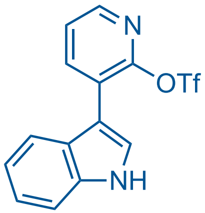

^{19}F NMR of **1ad** (376 MHz, $CDCl_3$)

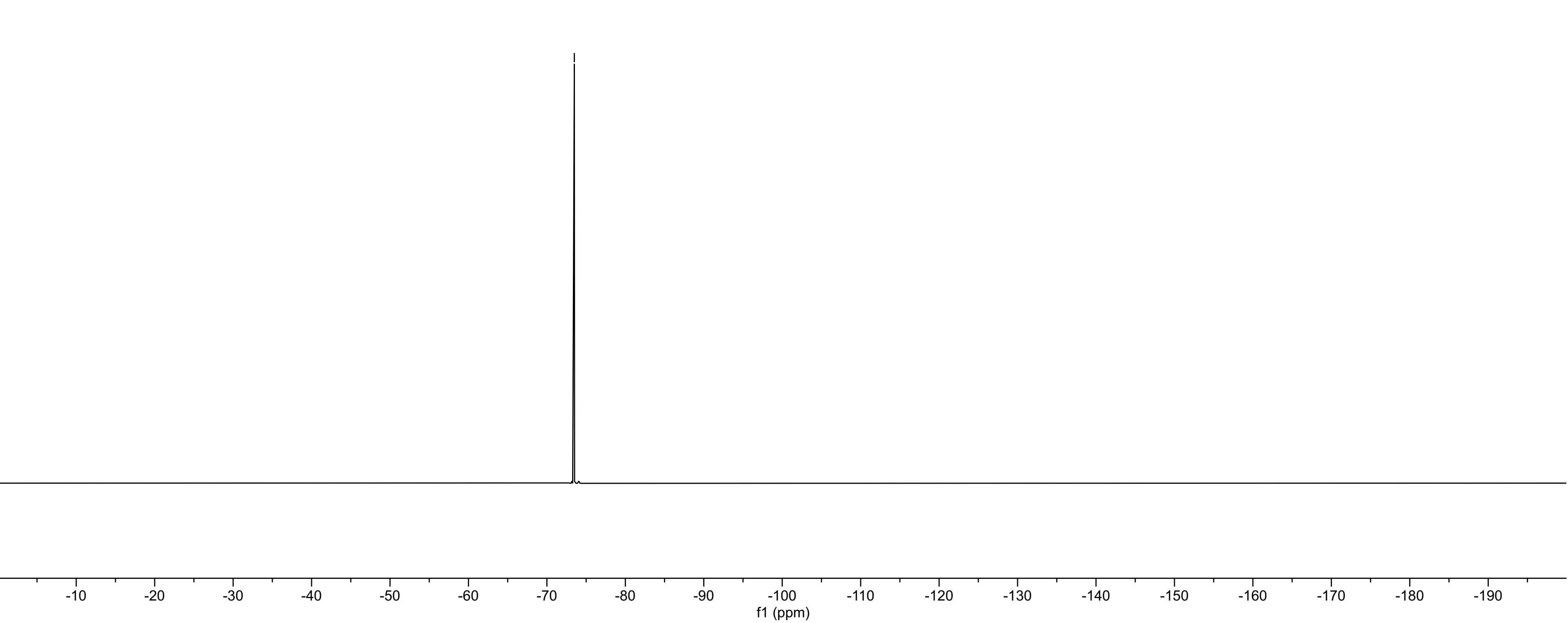

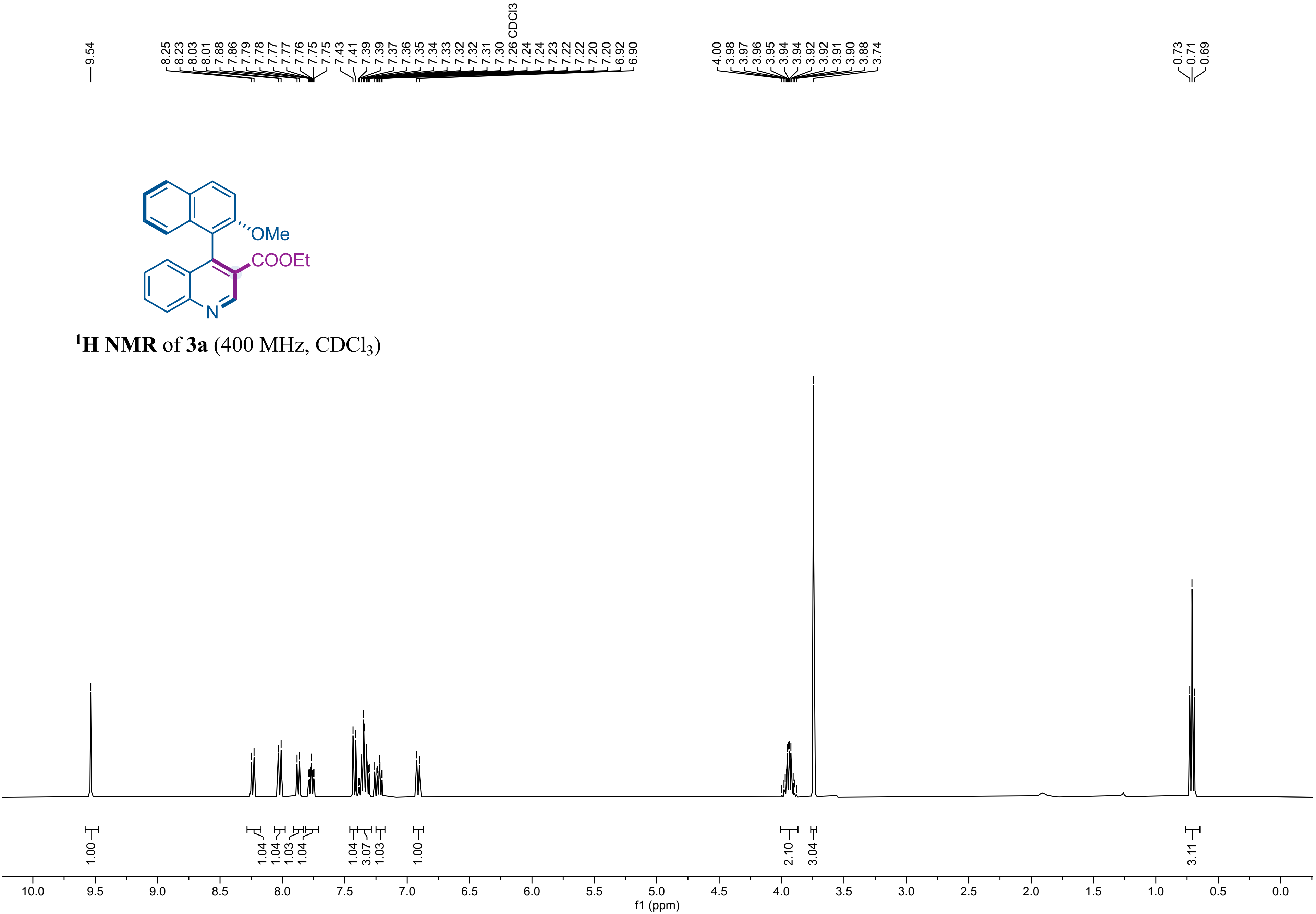

^{1}H NMR of **3a** (400 MHz, $CDCl_3$)

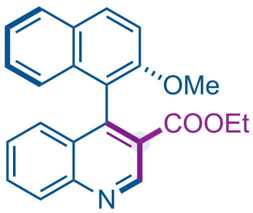

^{13}C NMR of 3a (101 MHz, $CDCl_3$)

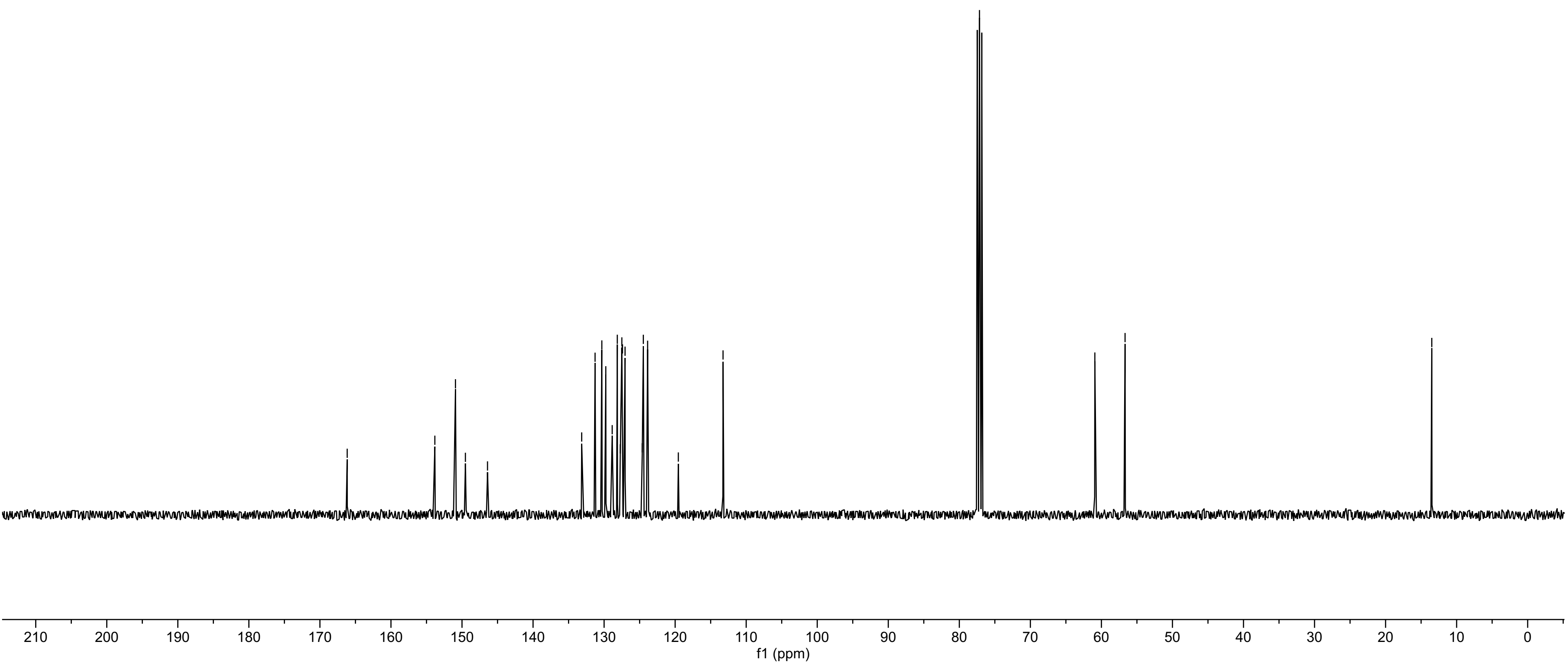

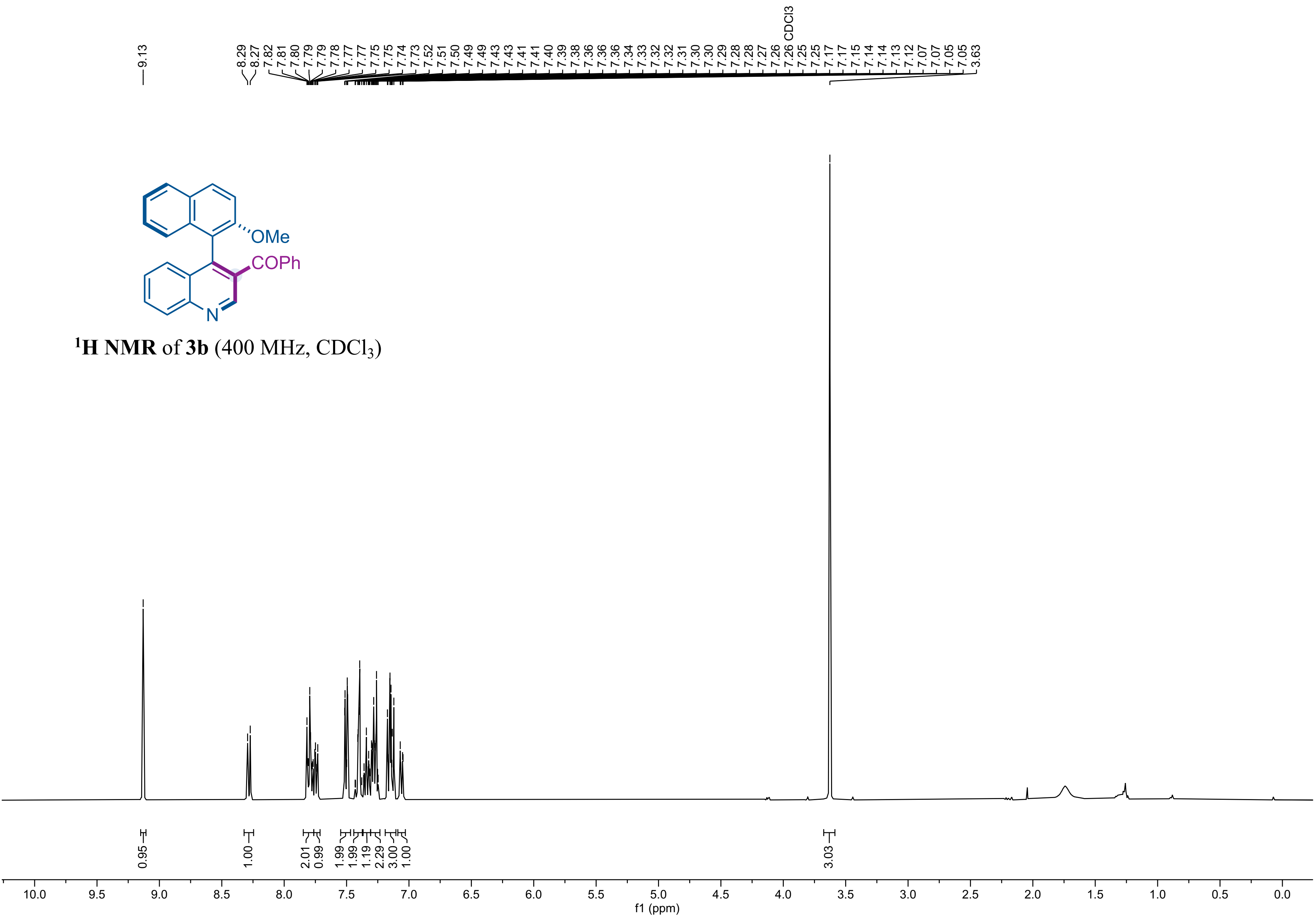

^{1}**H NMR** of **3b** (400 MHz, $CDCl_3$)

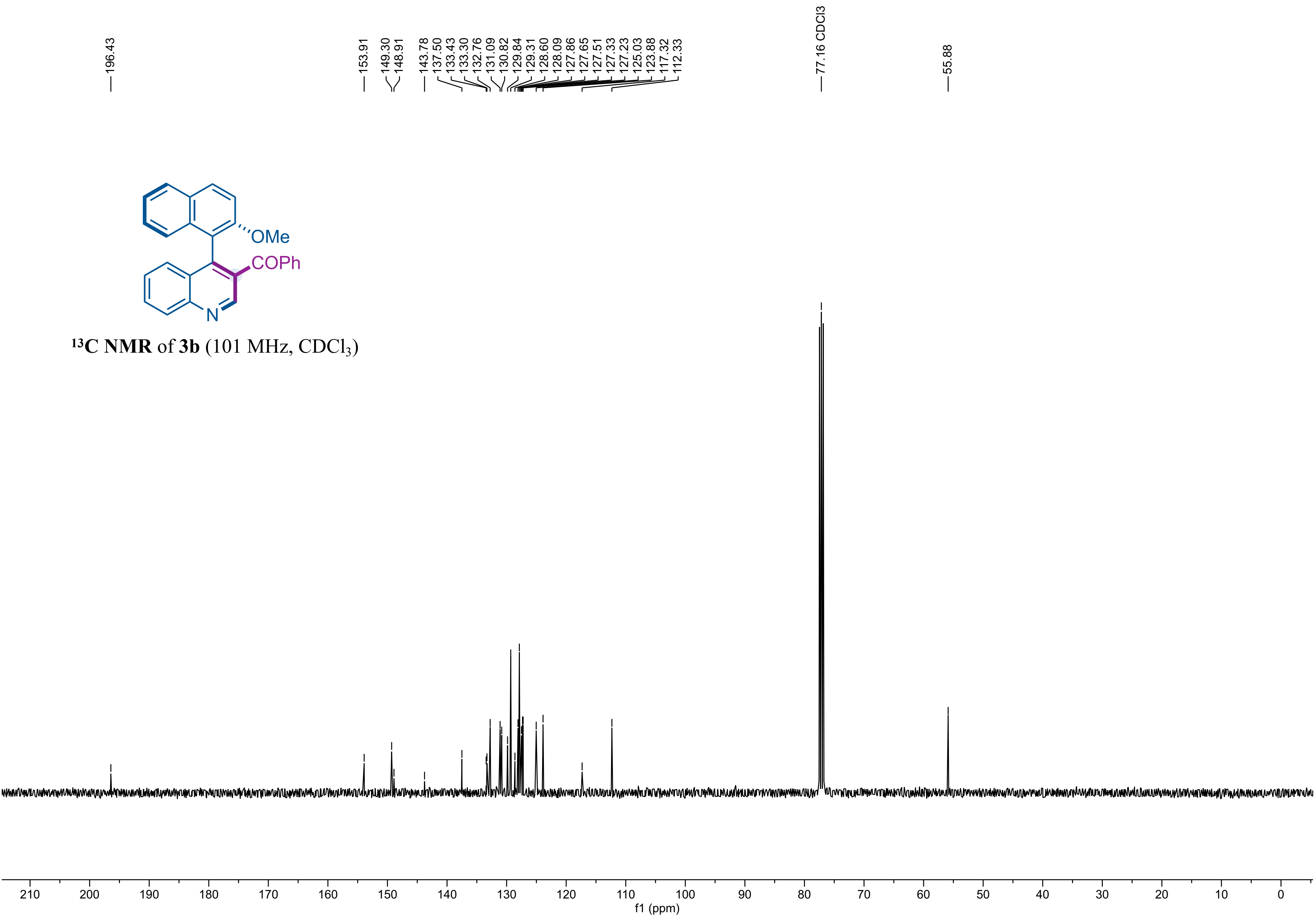

^{13}C NMR of **3b** (101 MHz, $CDCl_3$)

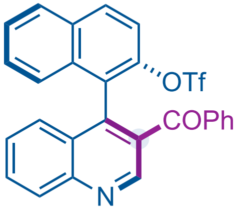

¹H NMR of **3c** (400 MHz, $CDCl_3$)

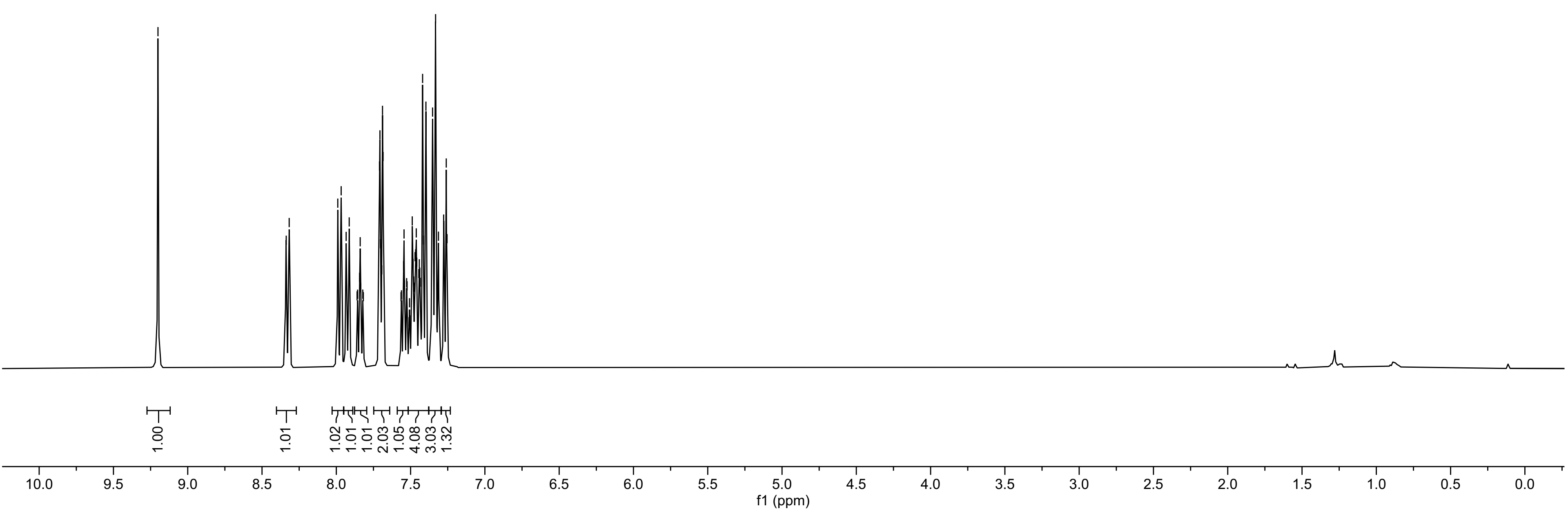

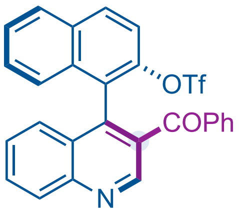

^{13}C NMR of **3c** (101 MHz, $CDCl_3$)

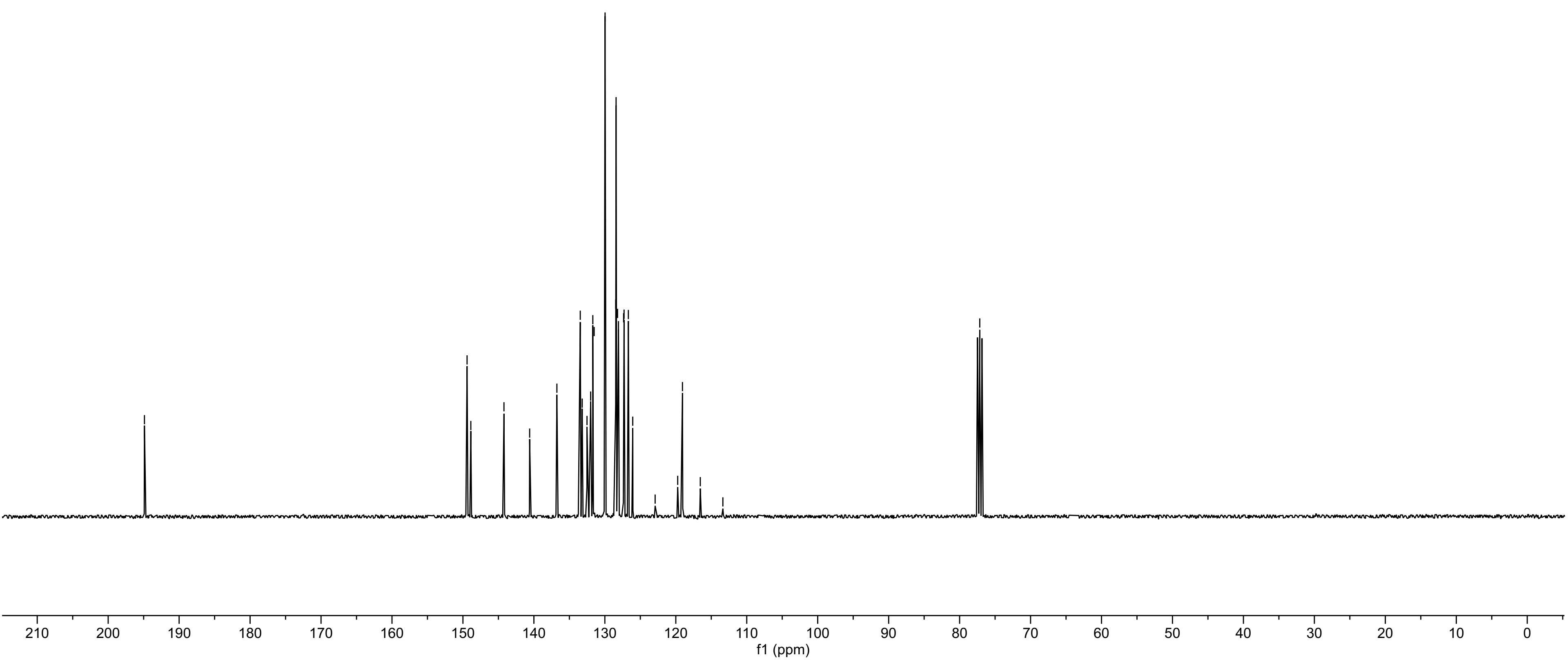

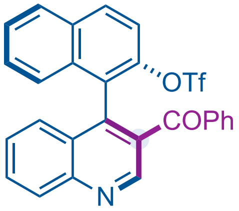

^{19}F NMR of **3c** (376 MHz, $CDCl_3$)

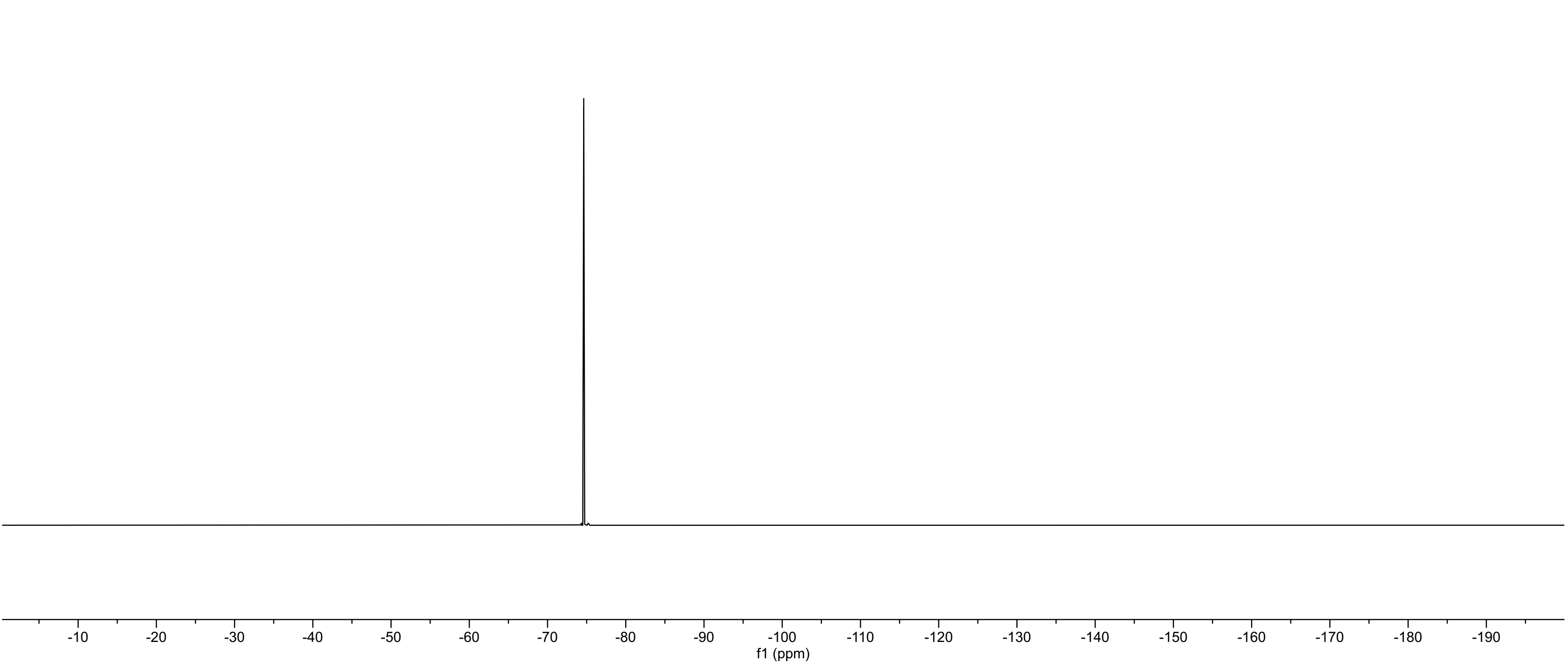

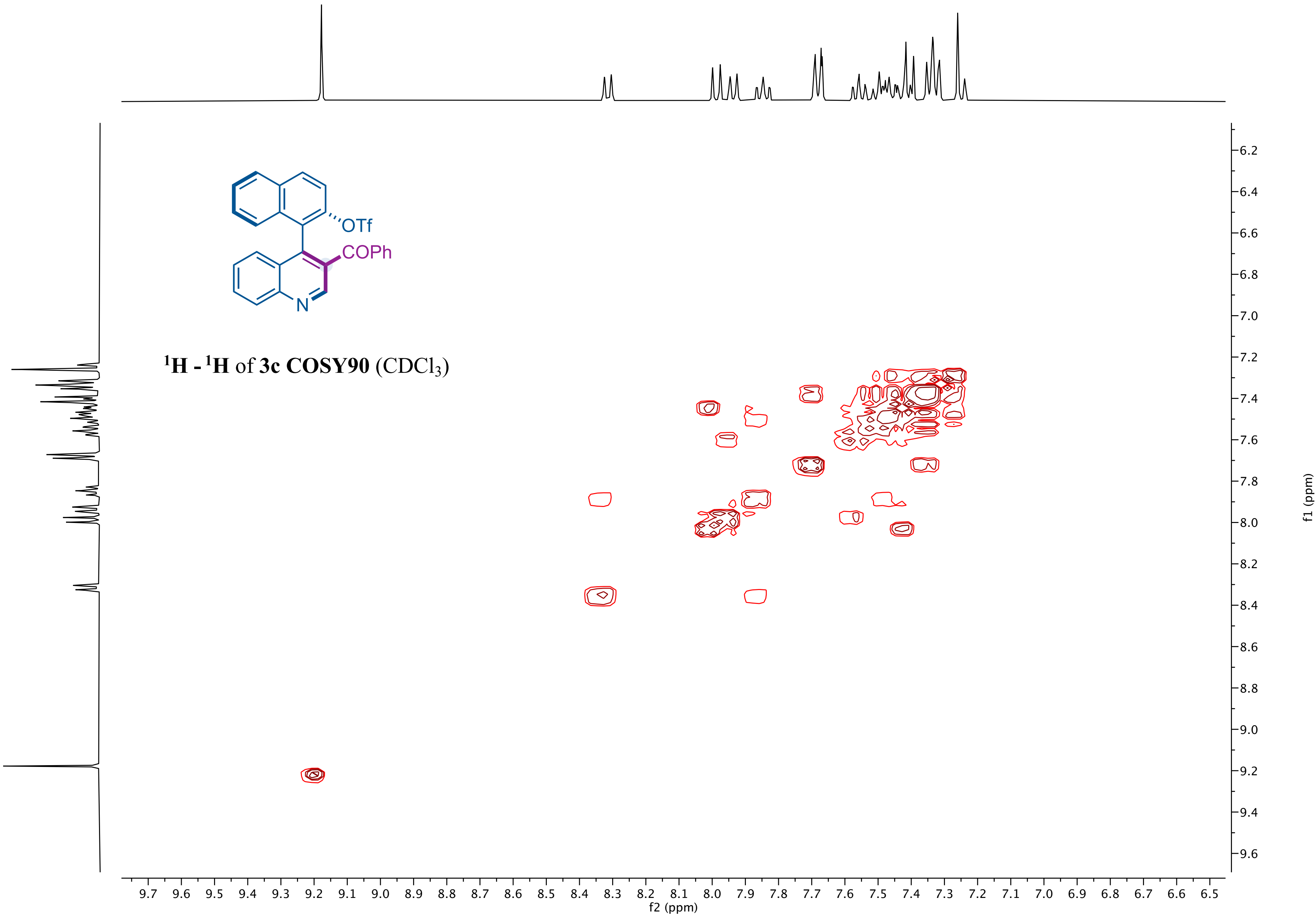
OTf
COPh
N
¹H - ¹H of 3c COSY90 (CDCl₃)
f2 (ppm)
f1 (ppm)

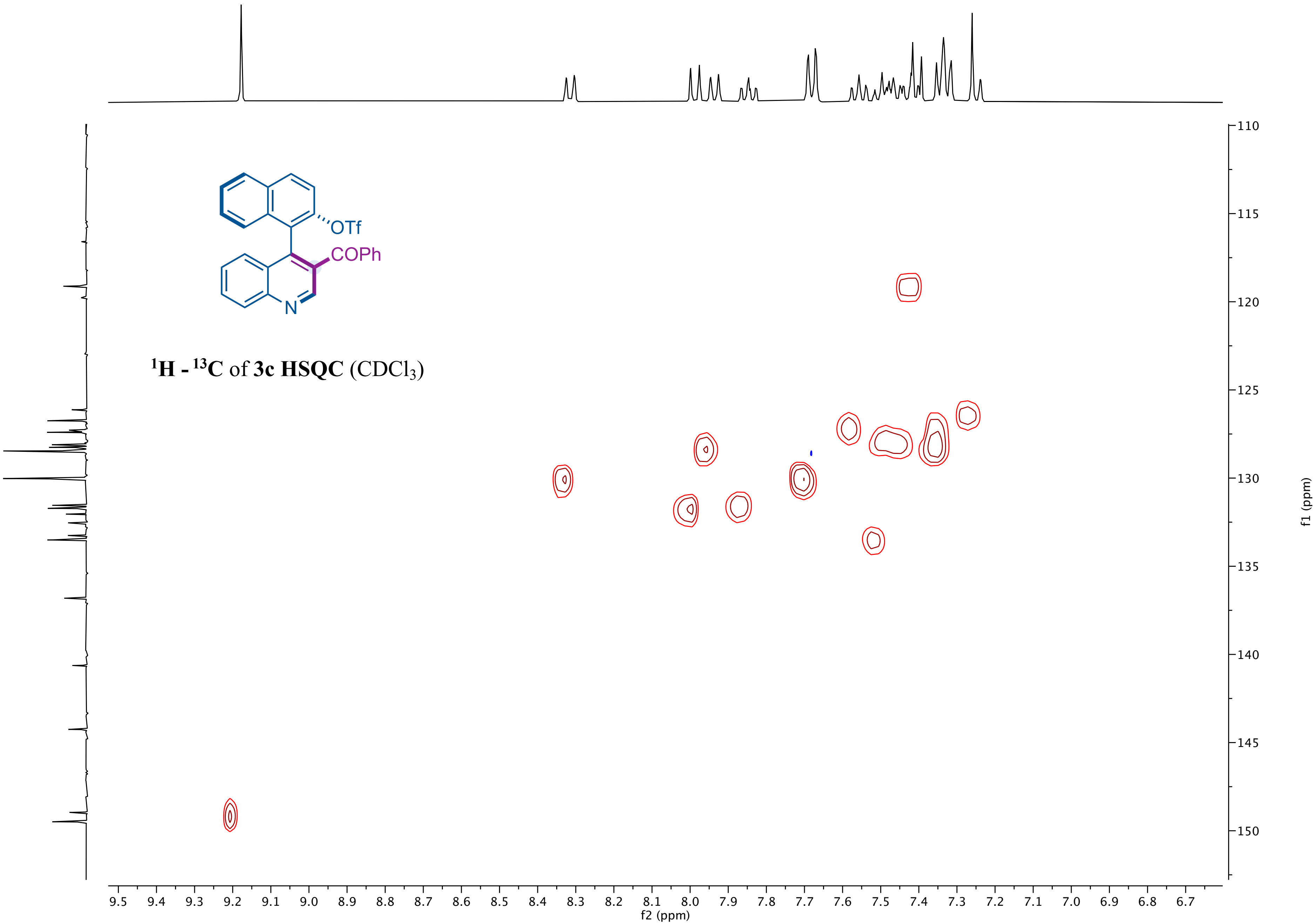

OTf
COPh
N
^{1}H - ^{13}C of 3c HSQC (CDCl$_3$)
f2 (ppm)
f1 (ppm)

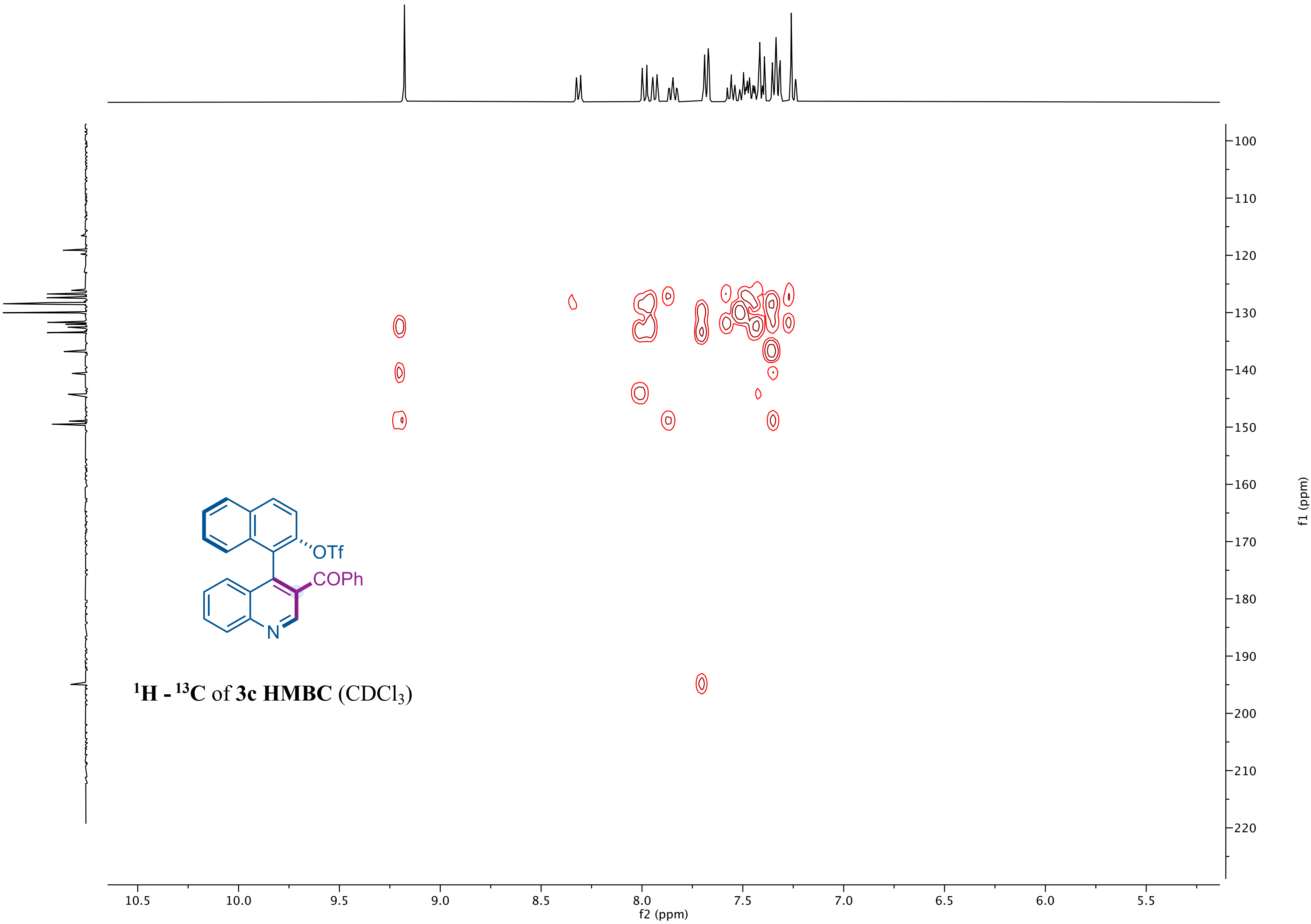

^{1}H - ^{13}C of **3c HMBC** ($CDCl_3$)

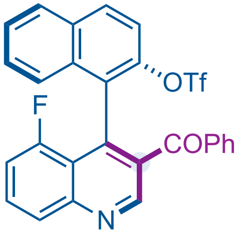

^{1}H NMR of **3d** (400 MHz, $CDCl_3$)

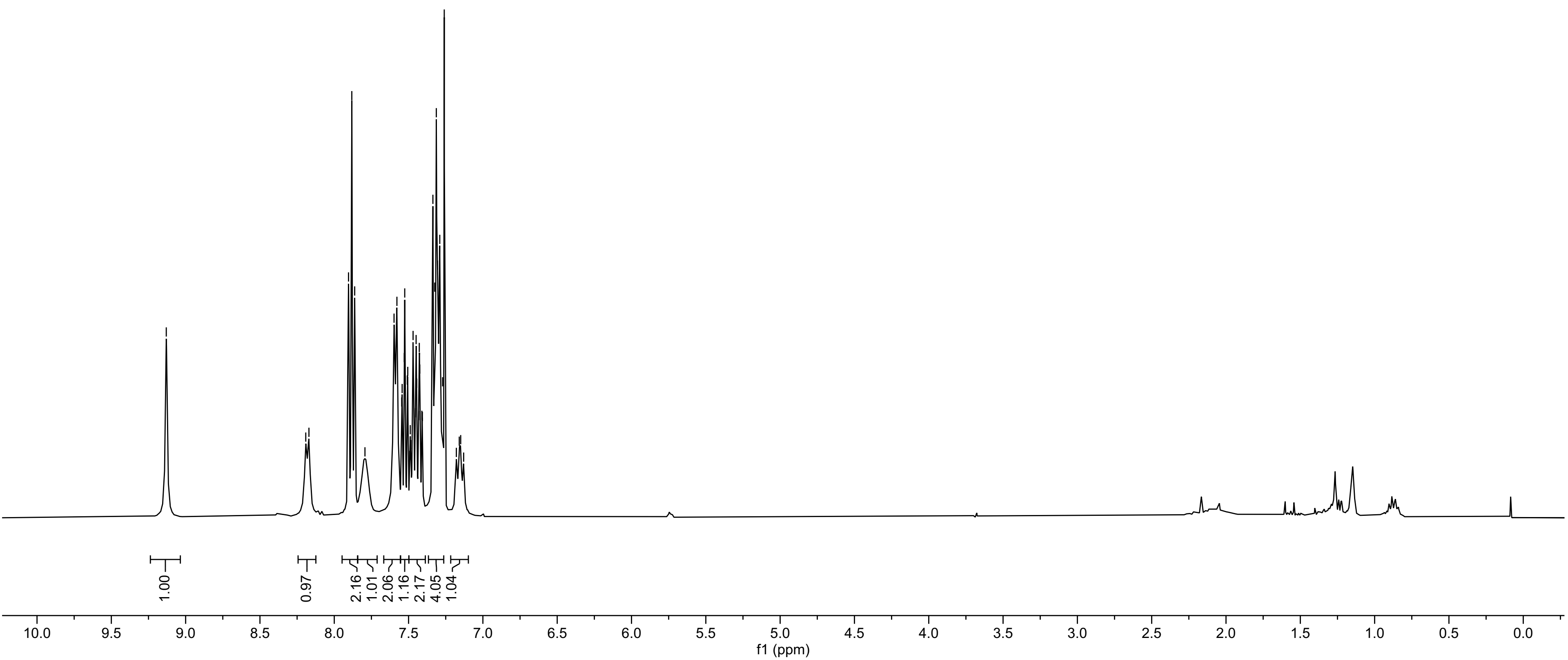

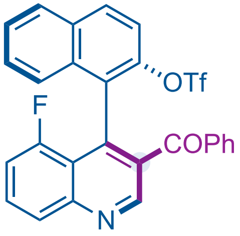

^{13}**C NMR** of **3d** (101 MHz, $CDCl_3$)

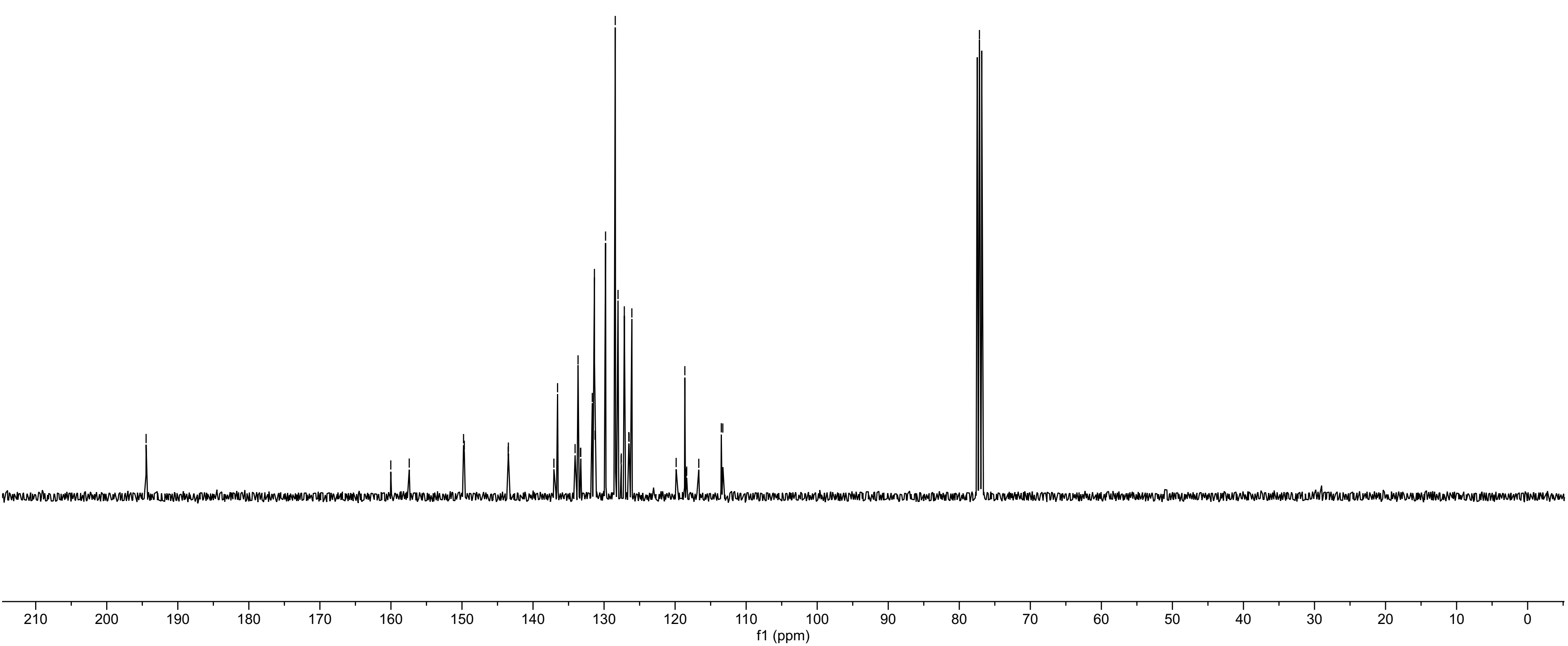

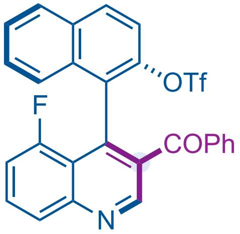

^{19}F NMR of **3d** (376 MHz, $CDCl_3$)

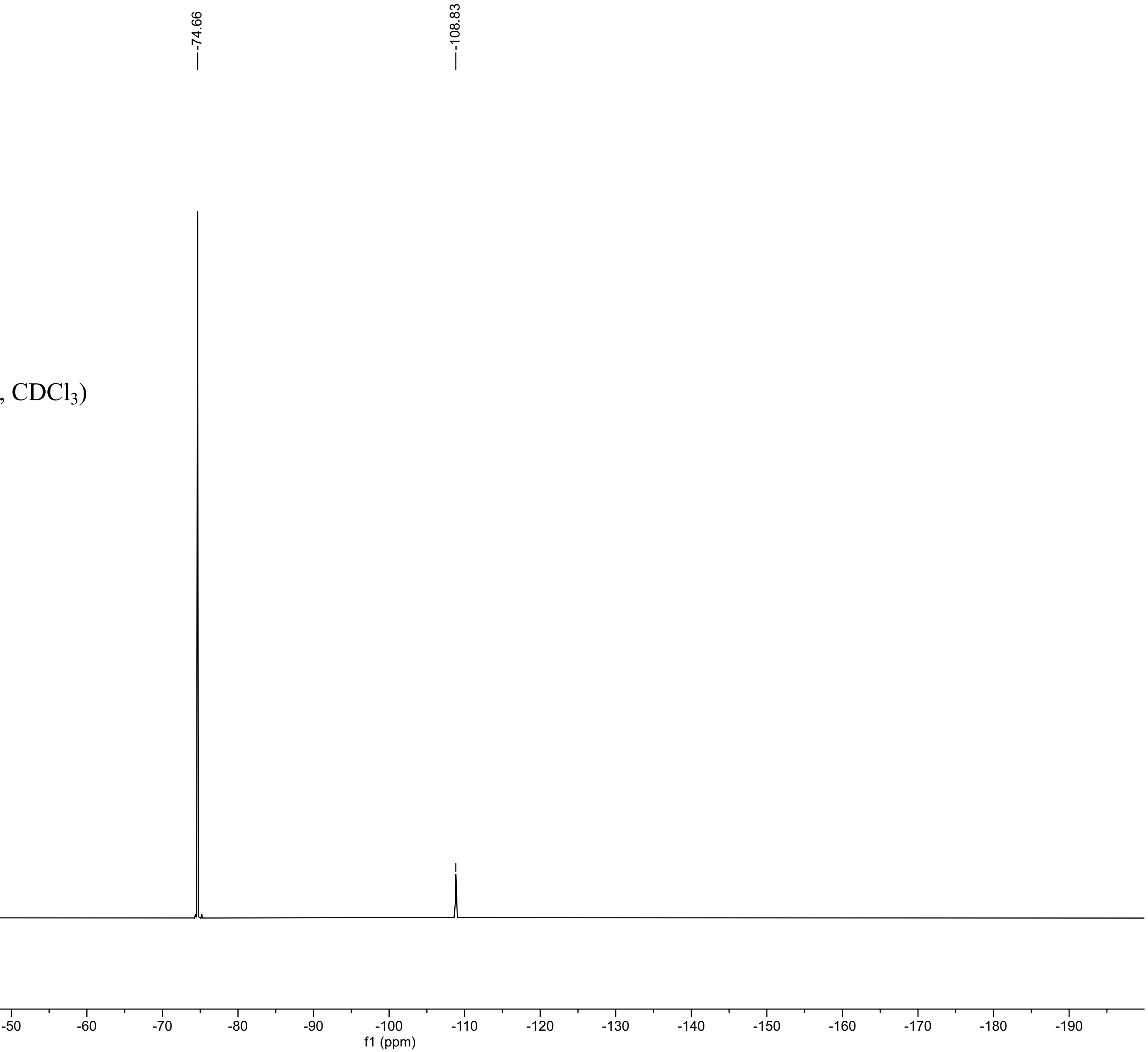

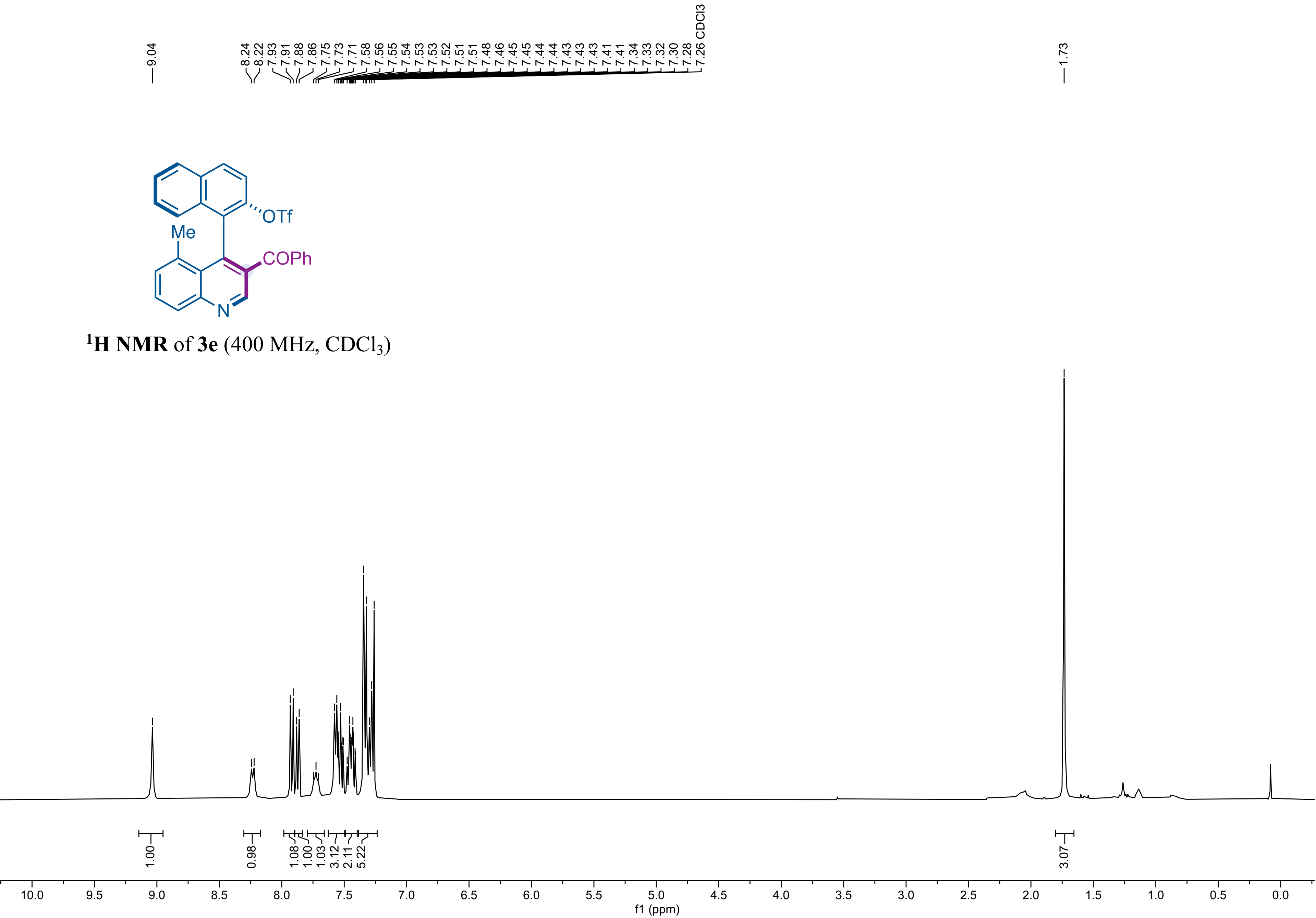

^{1}H NMR of **3e** (400 MHz, $CDCl_3$)

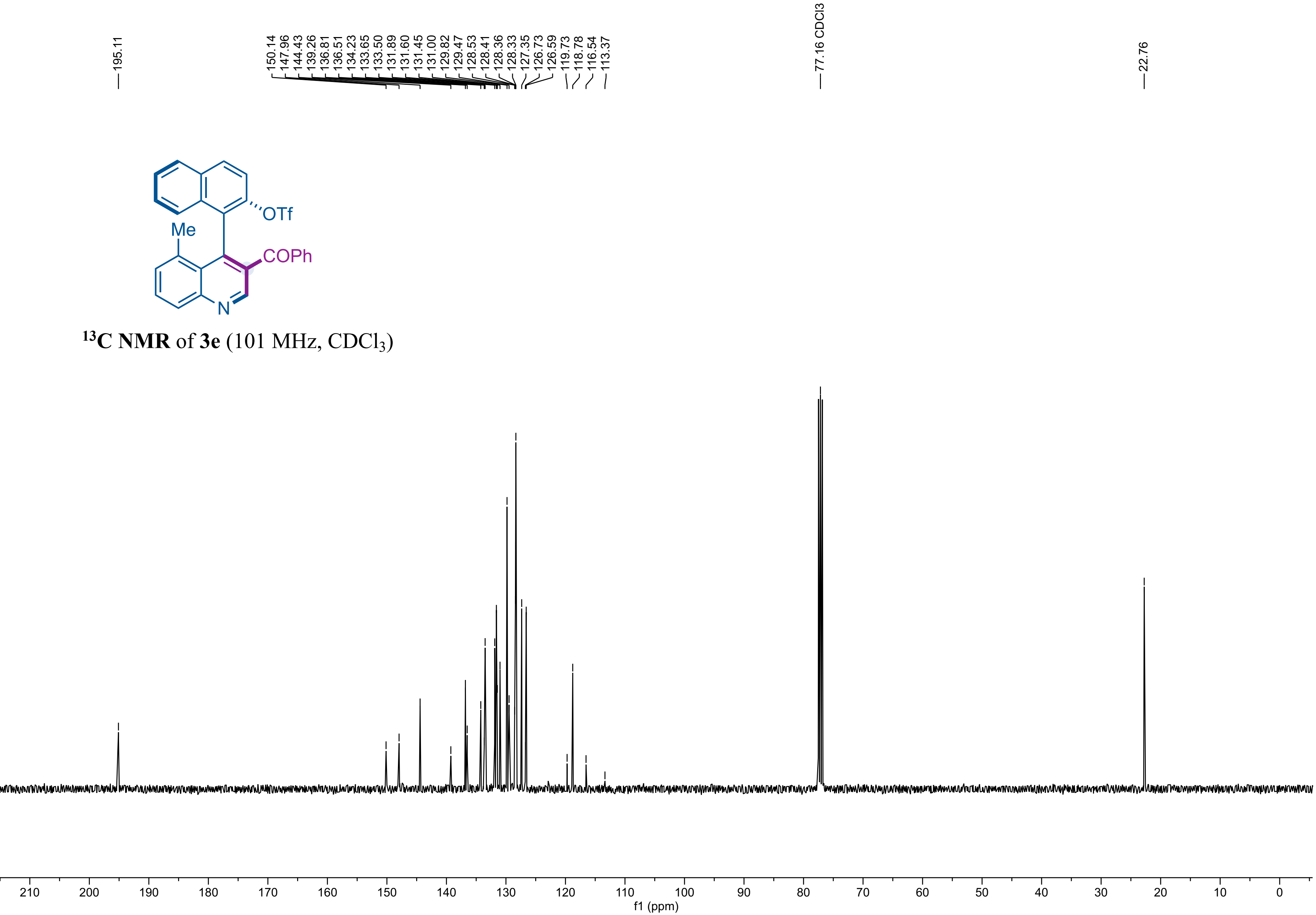

^{13}**C NMR** of **3e** (101 MHz, $CDCl_3$)

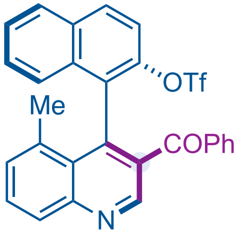

19F NMR of **3e** (376 MHz, $CDCl_3$)

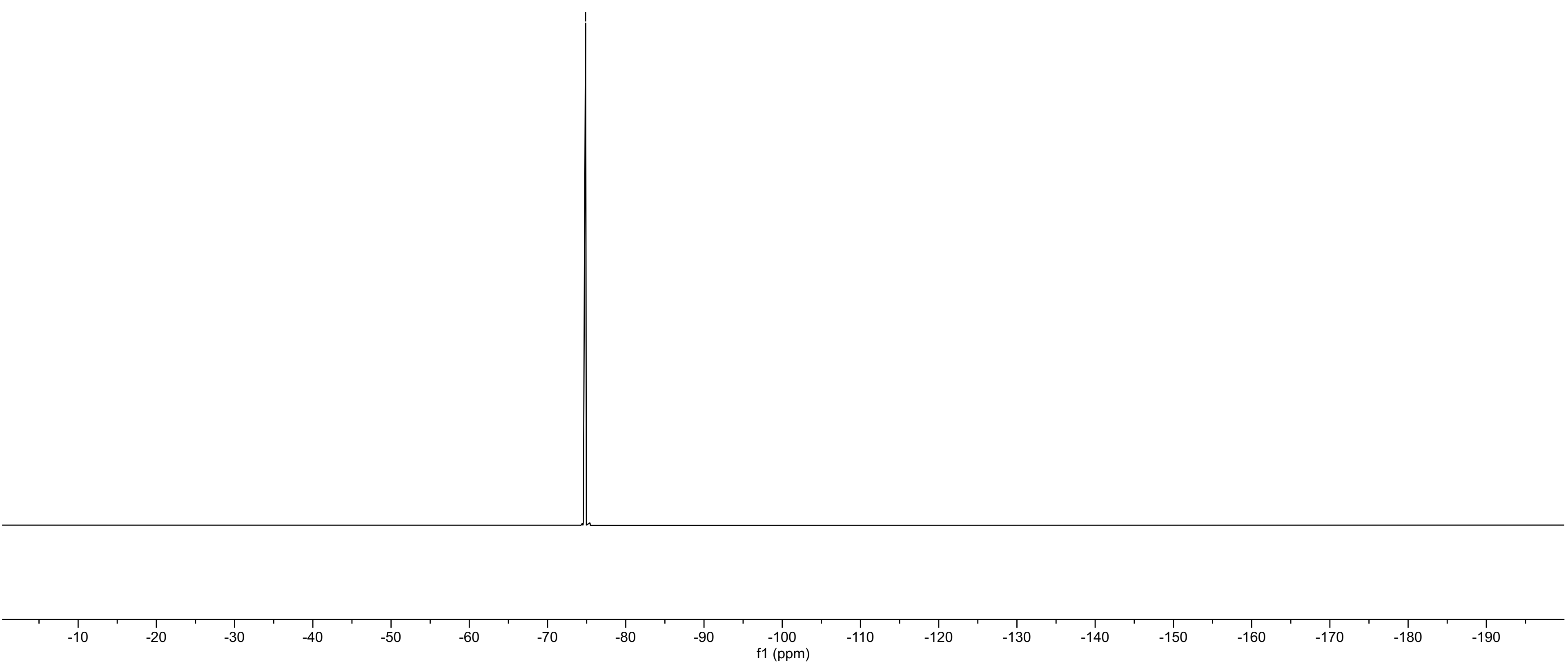

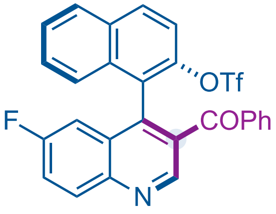

^{1}H NMR of **3f** (400 MHz, $CDCl_3$)

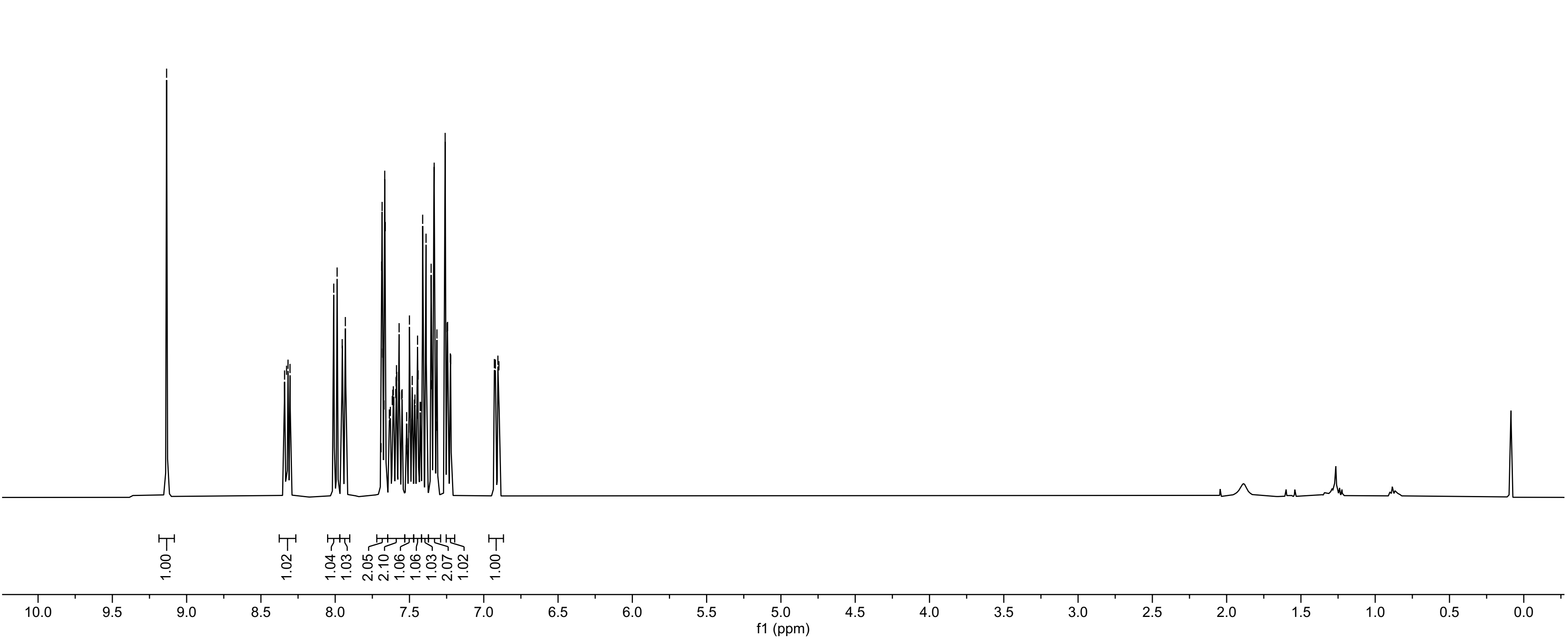

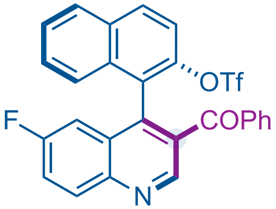

^{13}C NMR of **3f** (101 MHz, CDCl$_3$)

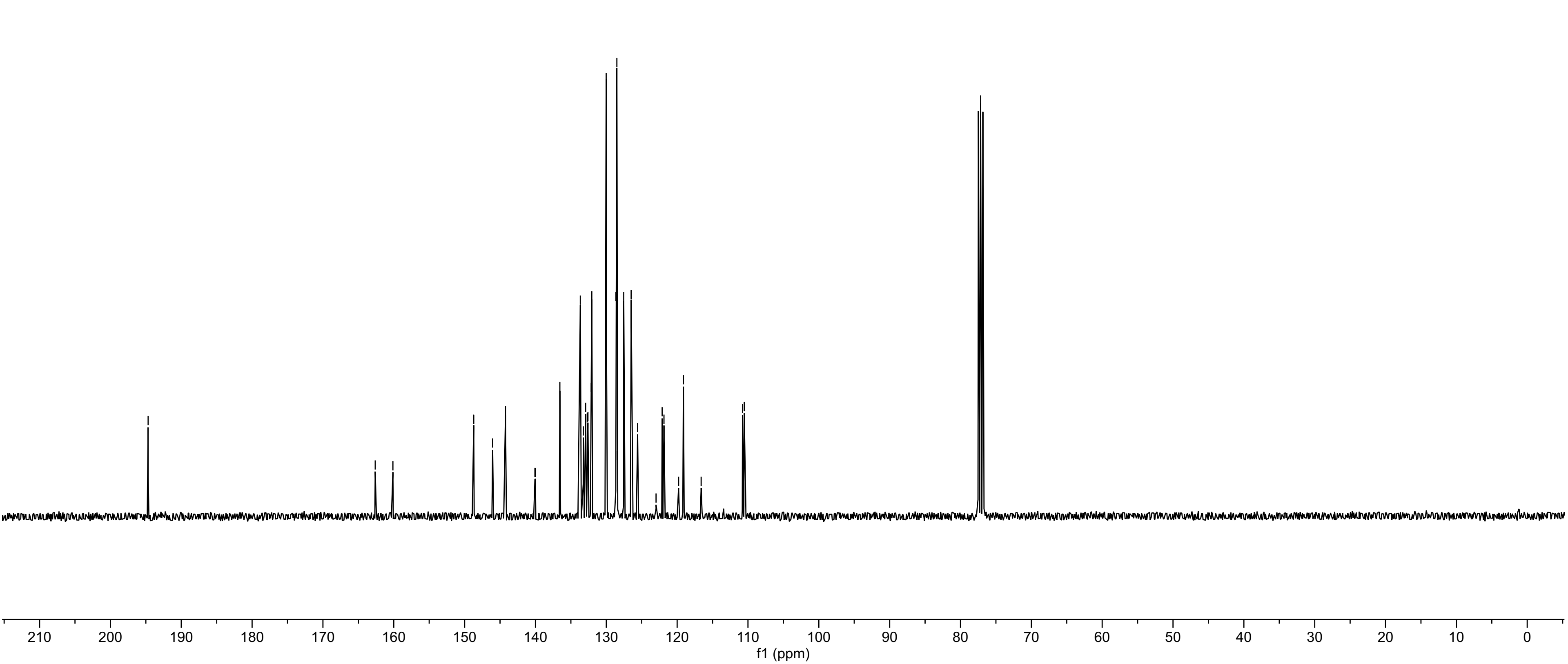

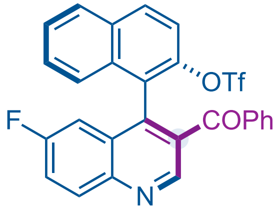

^{19}F NMR of **3f** (376 MHz, $CDCl_3$)

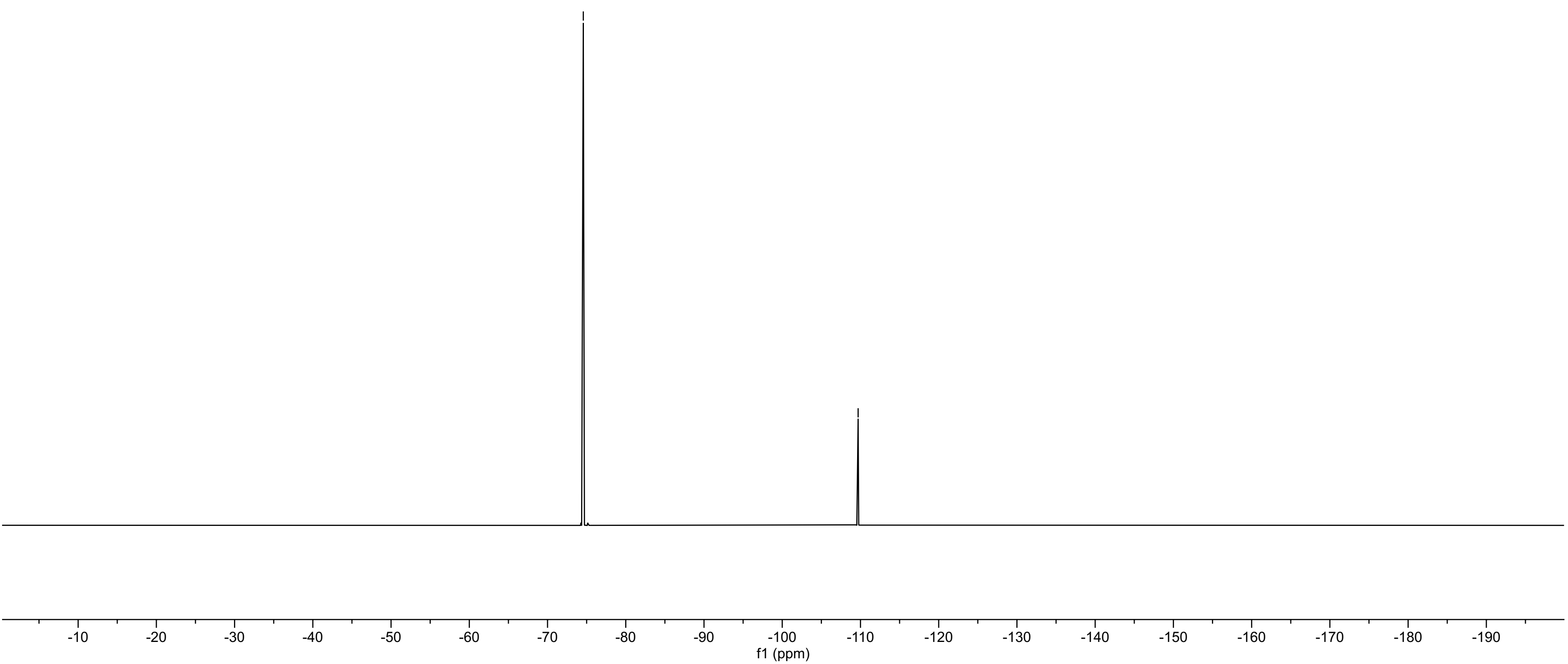

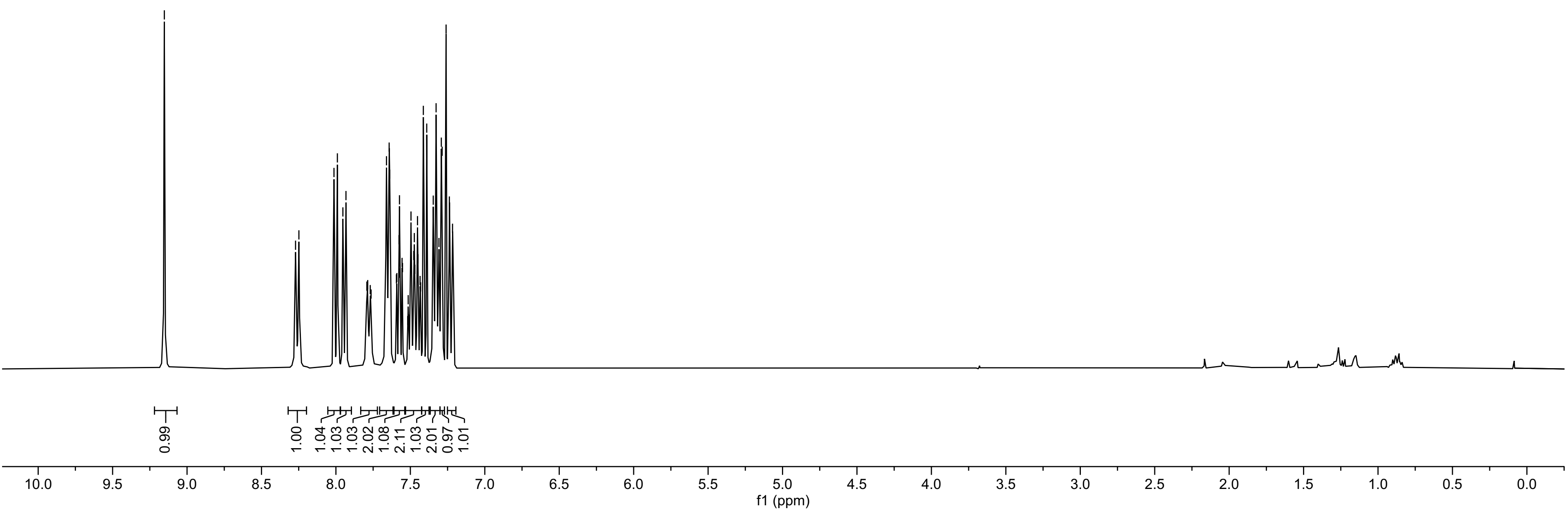

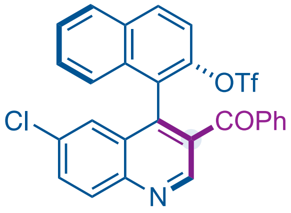

^{1}H NMR of **3g** (400 MHz, $CDCl_3$)

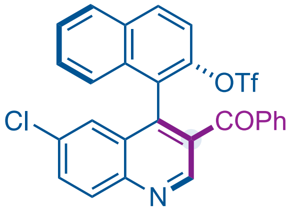

^{13}**C NMR** of **3g** (101 MHz, $CDCl_3$)

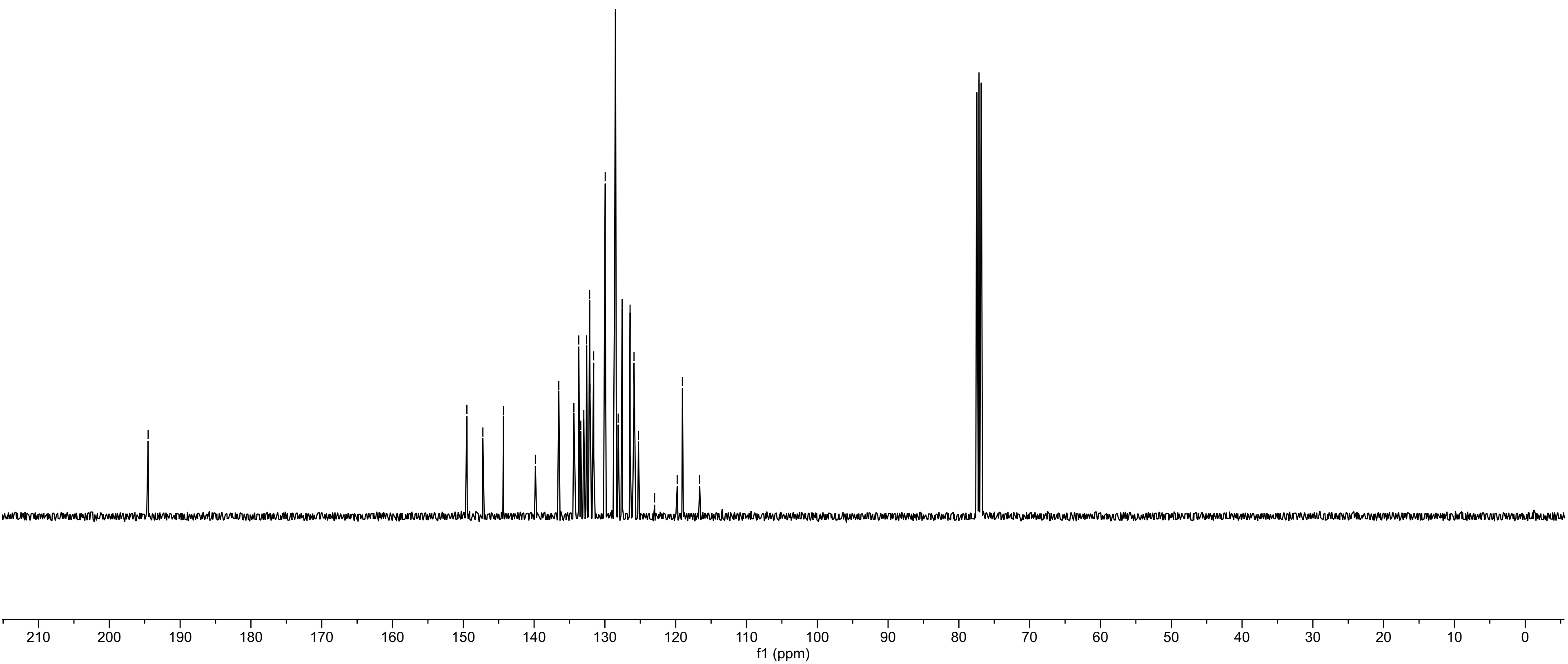

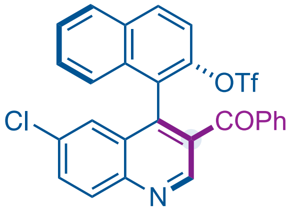

^{19}F NMR of **3g** (376 MHz, $CDCl_3$)

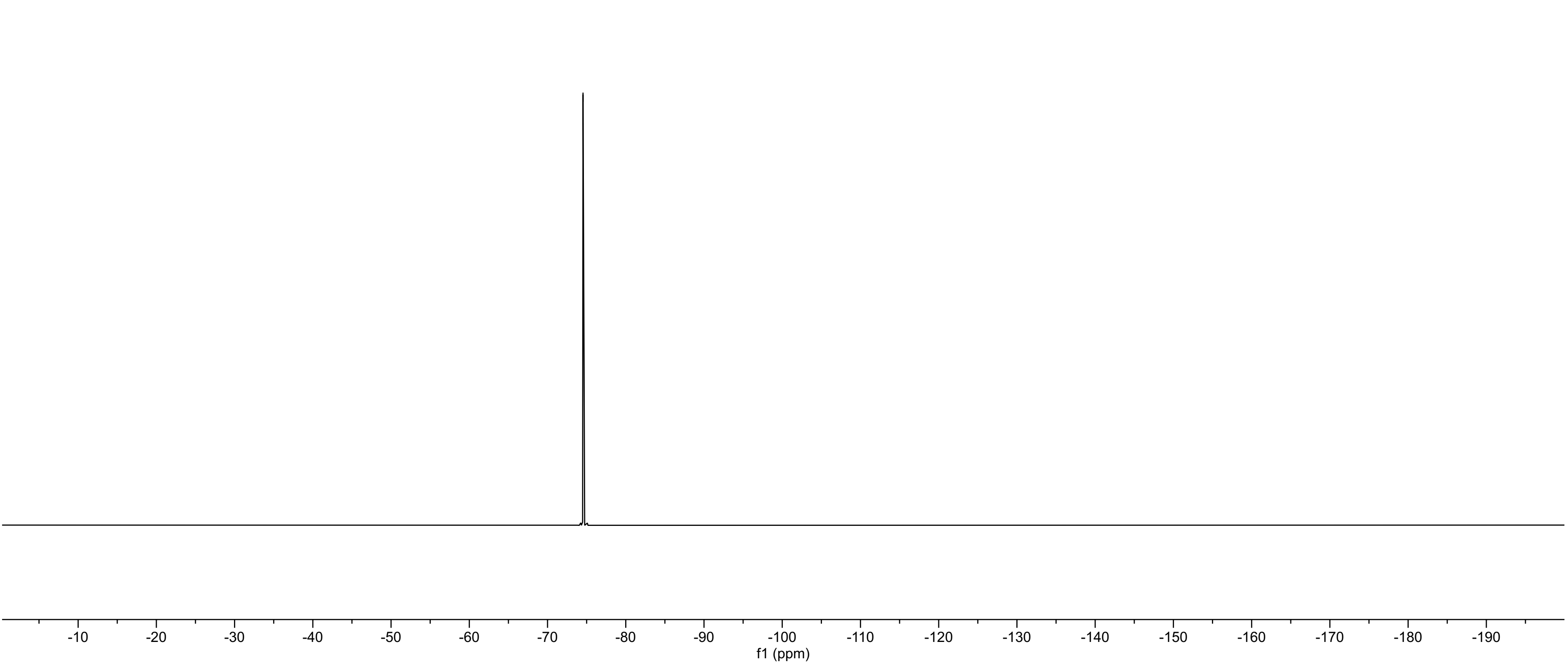

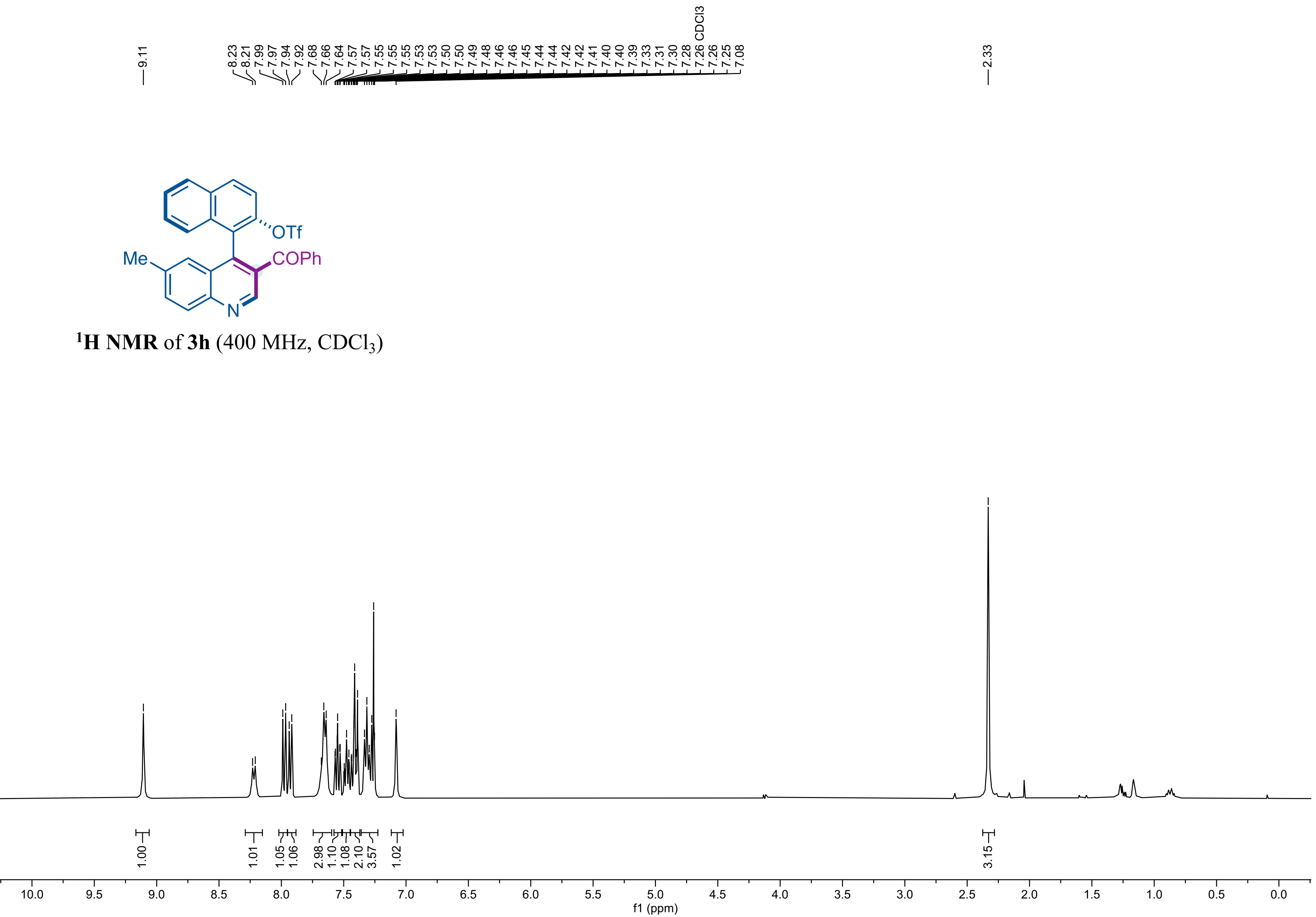

^{1}H NMR of **3h** (400 MHz, $CDCl_3$)

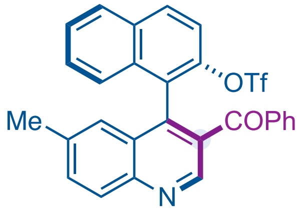

^{13}C NMR of **3h** (101 MHz, $CDCl_3$)

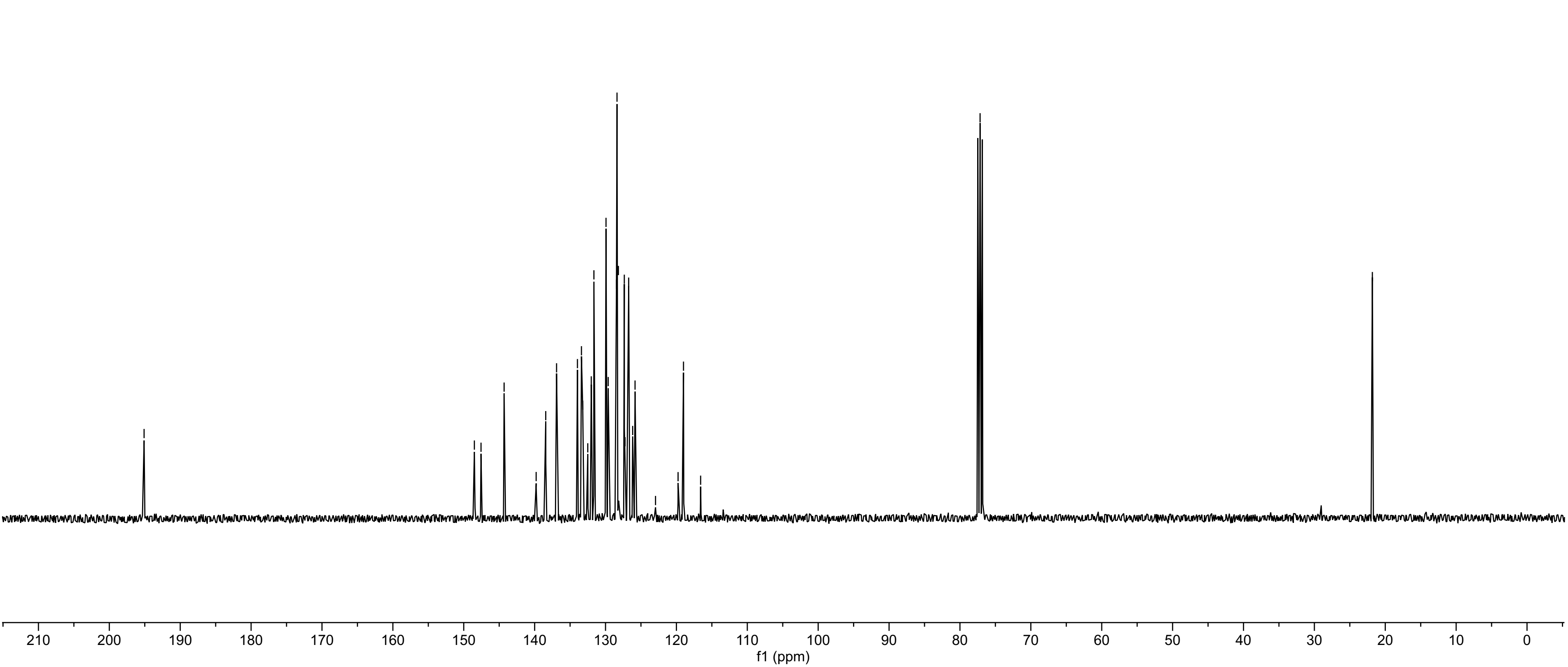

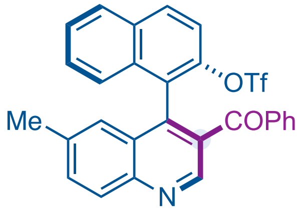

^{19}F **NMR** of **3h** (376 MHz, $CDCl_3$)

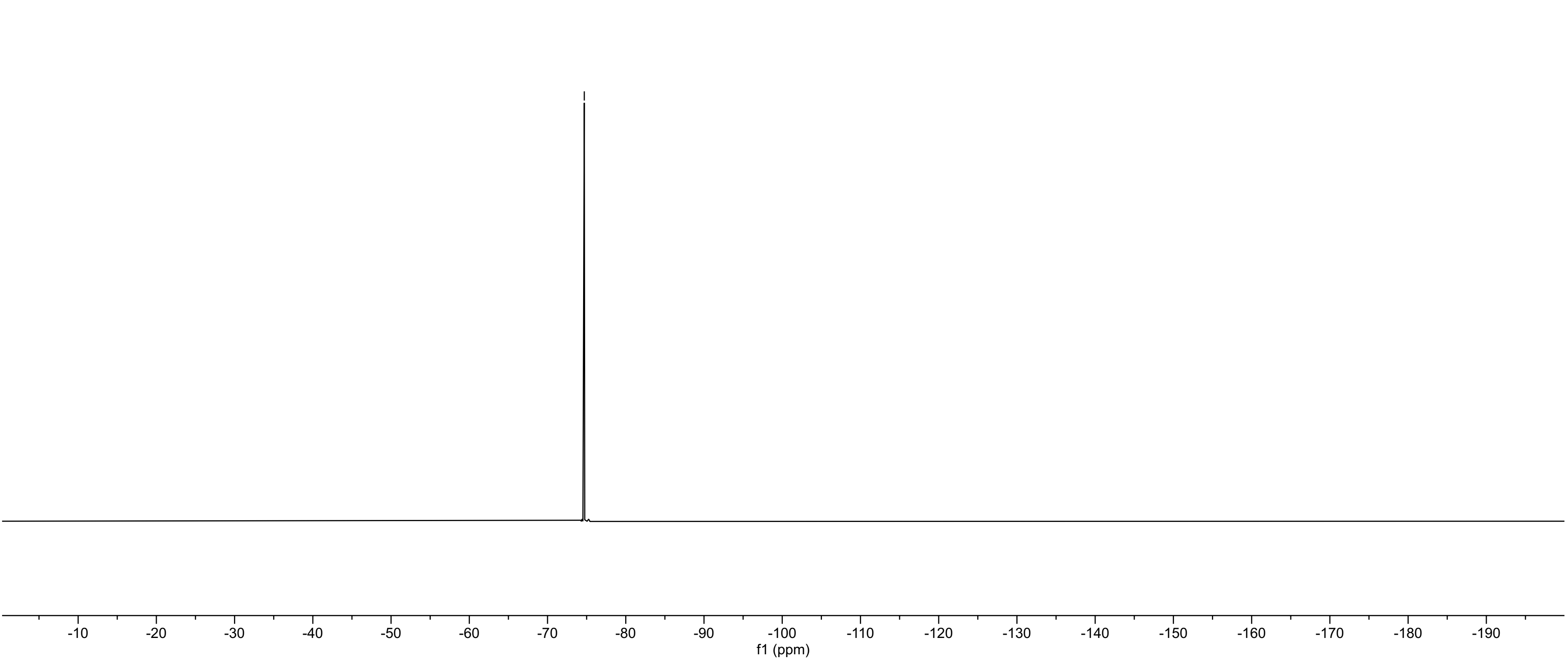

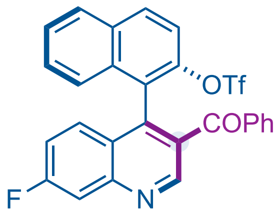

^{1}H NMR of **3i** (400 MHz, $CDCl_3$)

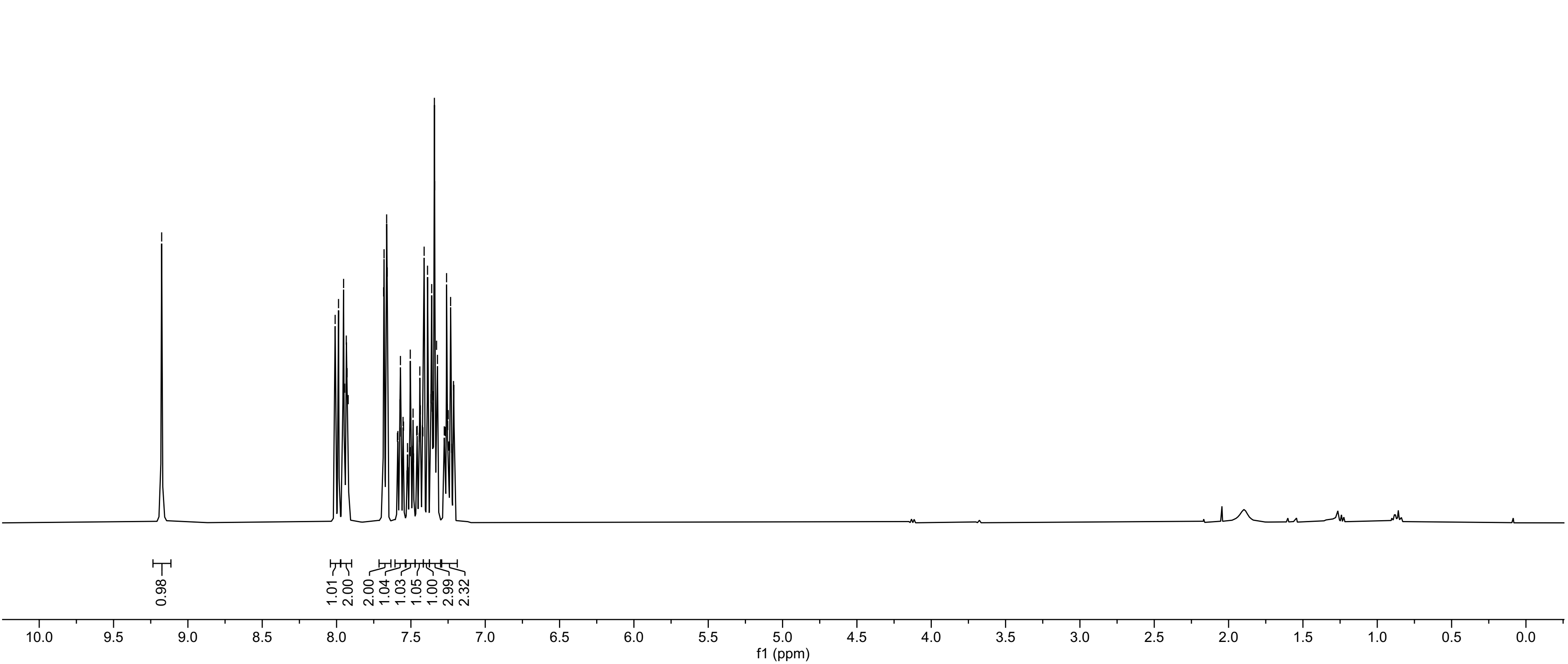

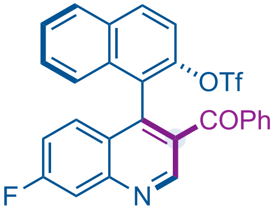

^{13}C **NMR** of **3i** (101 MHz, $CDCl_3$)

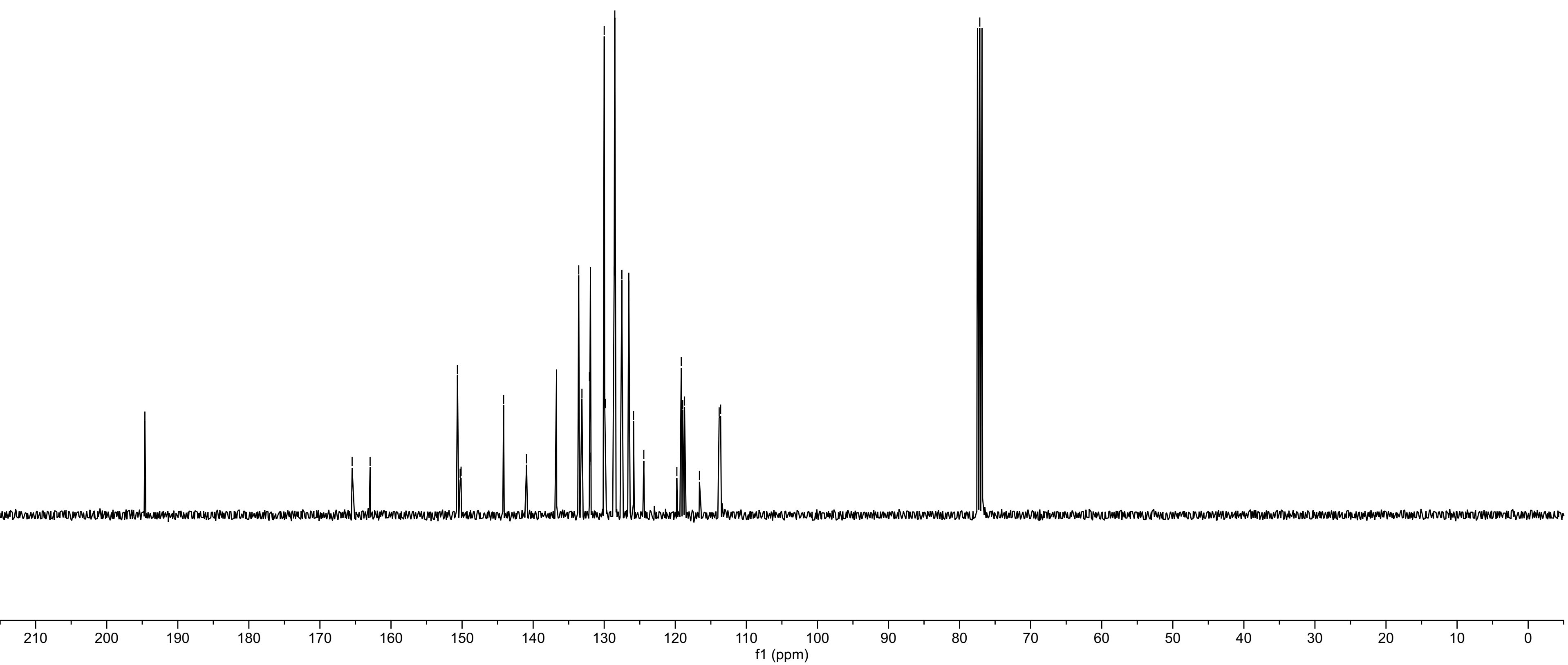

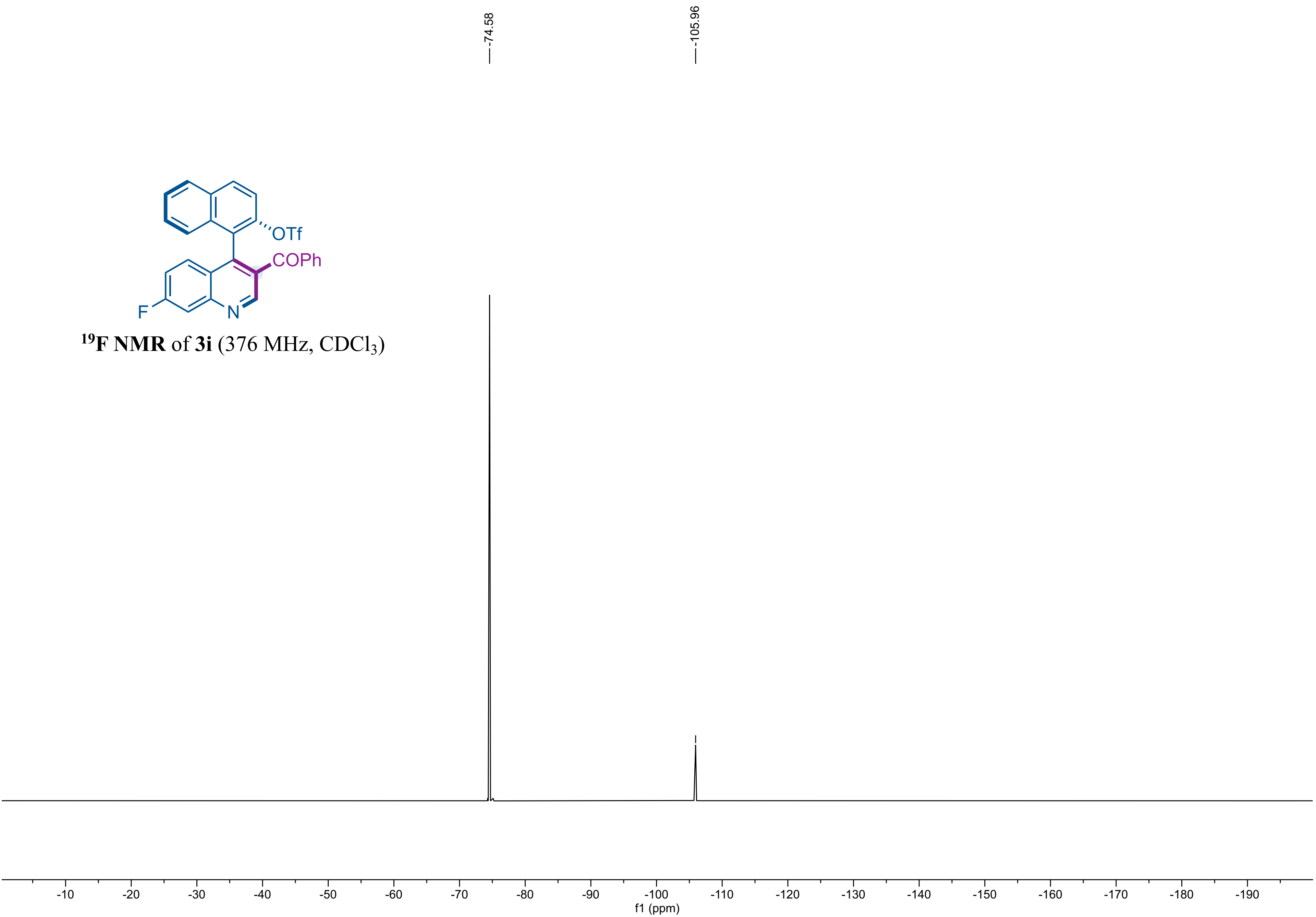

^{19}F NMR of **3i** (376 MHz, $CDCl_3$)

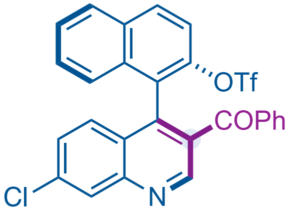

^{1}H NMR of 3j (400 MHz, $CDCl_3$)

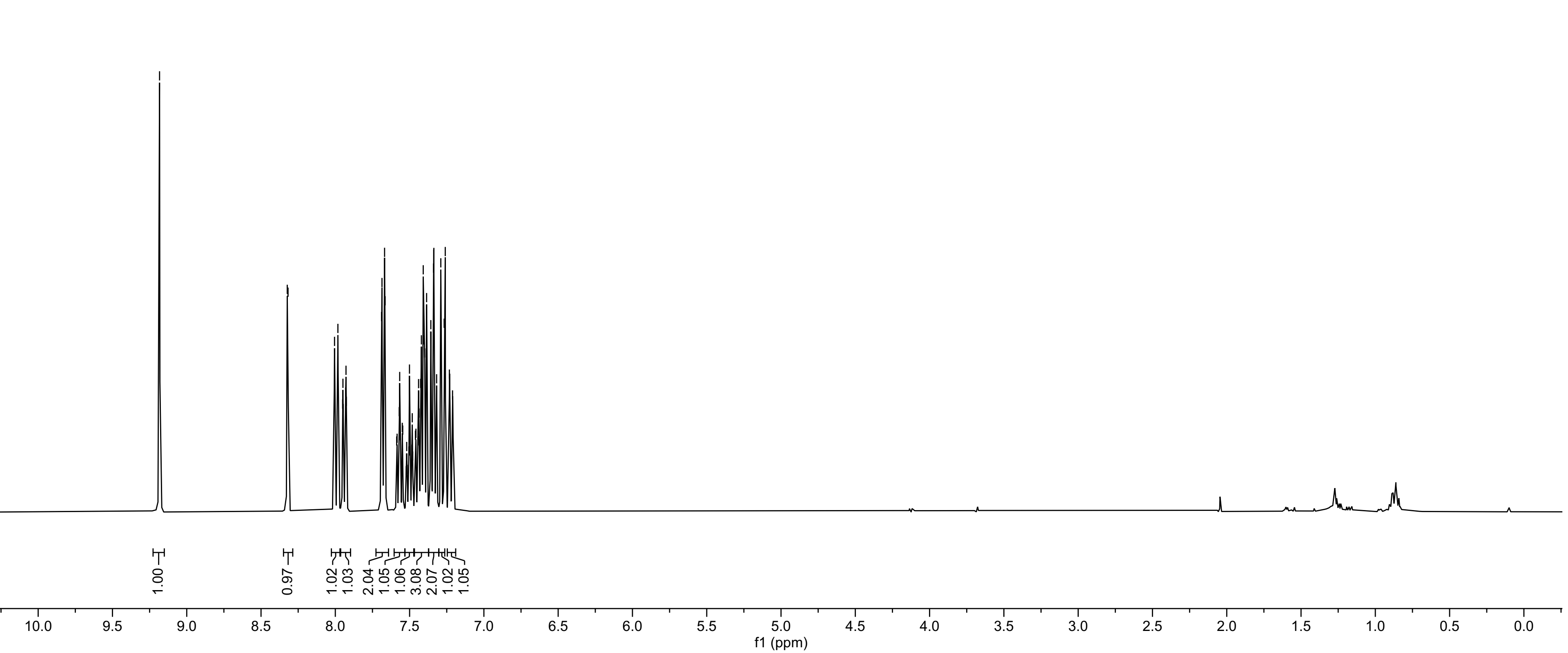

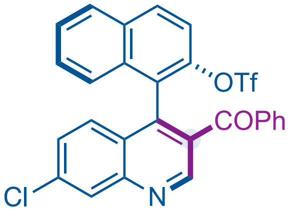

^{13}**C NMR** of **3j** (101 MHz, $CDCl_3$)

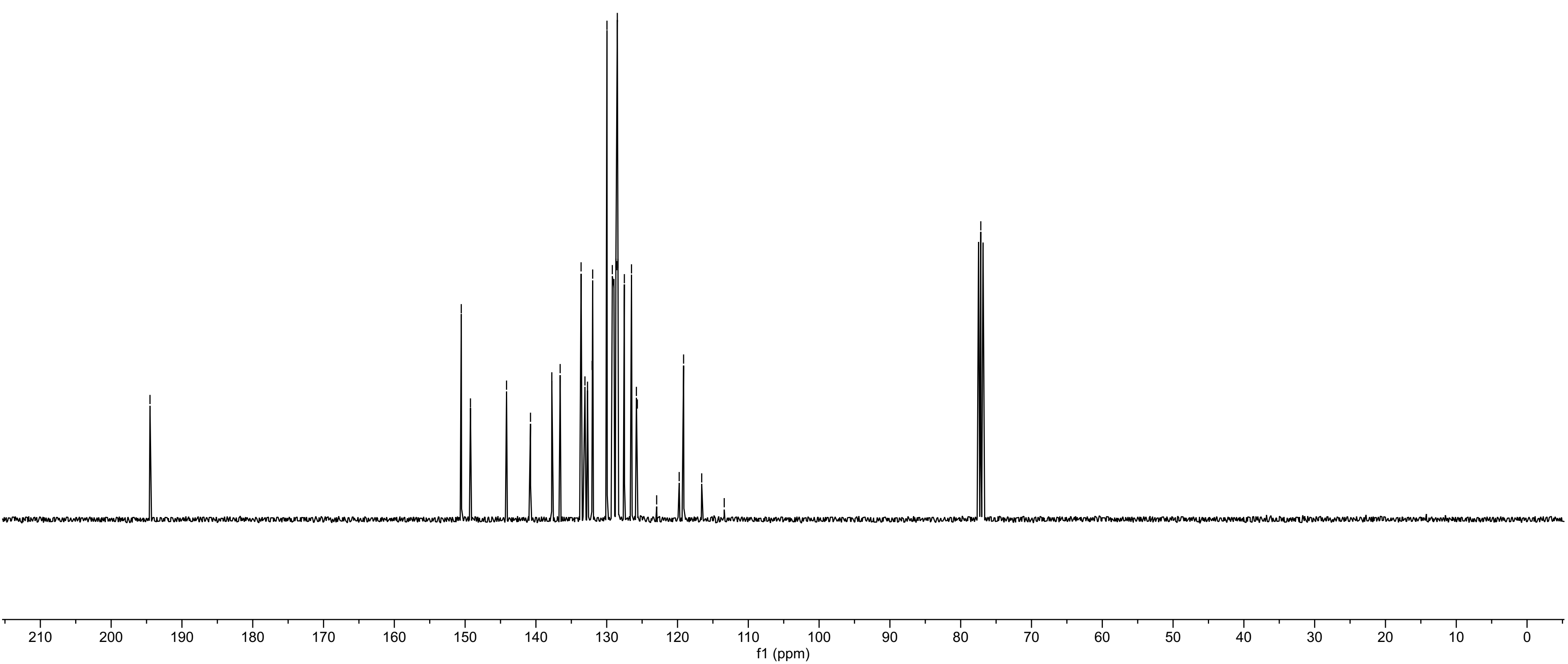

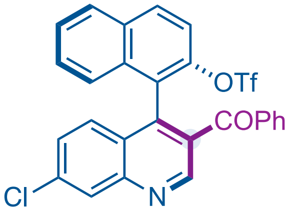

^{19}**F NMR** of **3j** (376 MHz, $CDCl_3$)

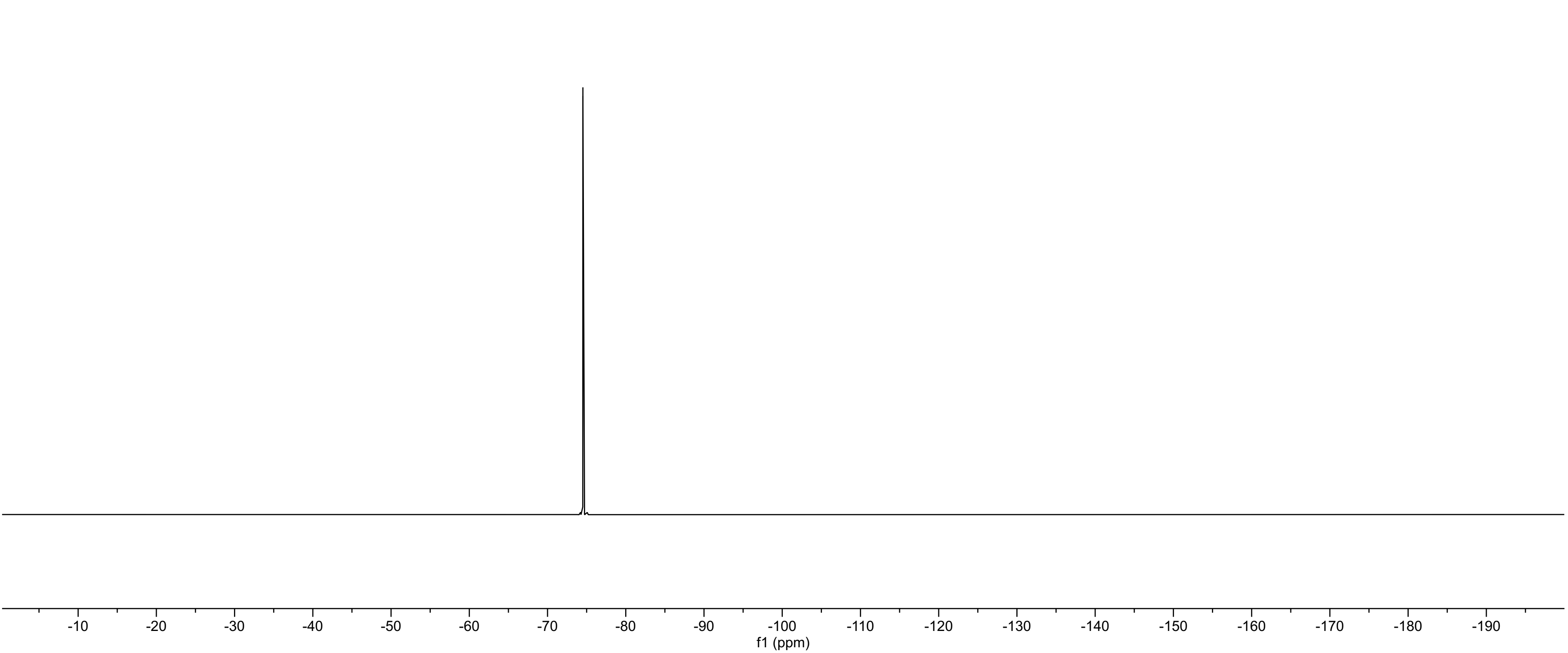

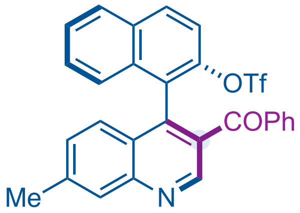

^{1}H NMR of **3k** (400 MHz, $CDCl_3$)

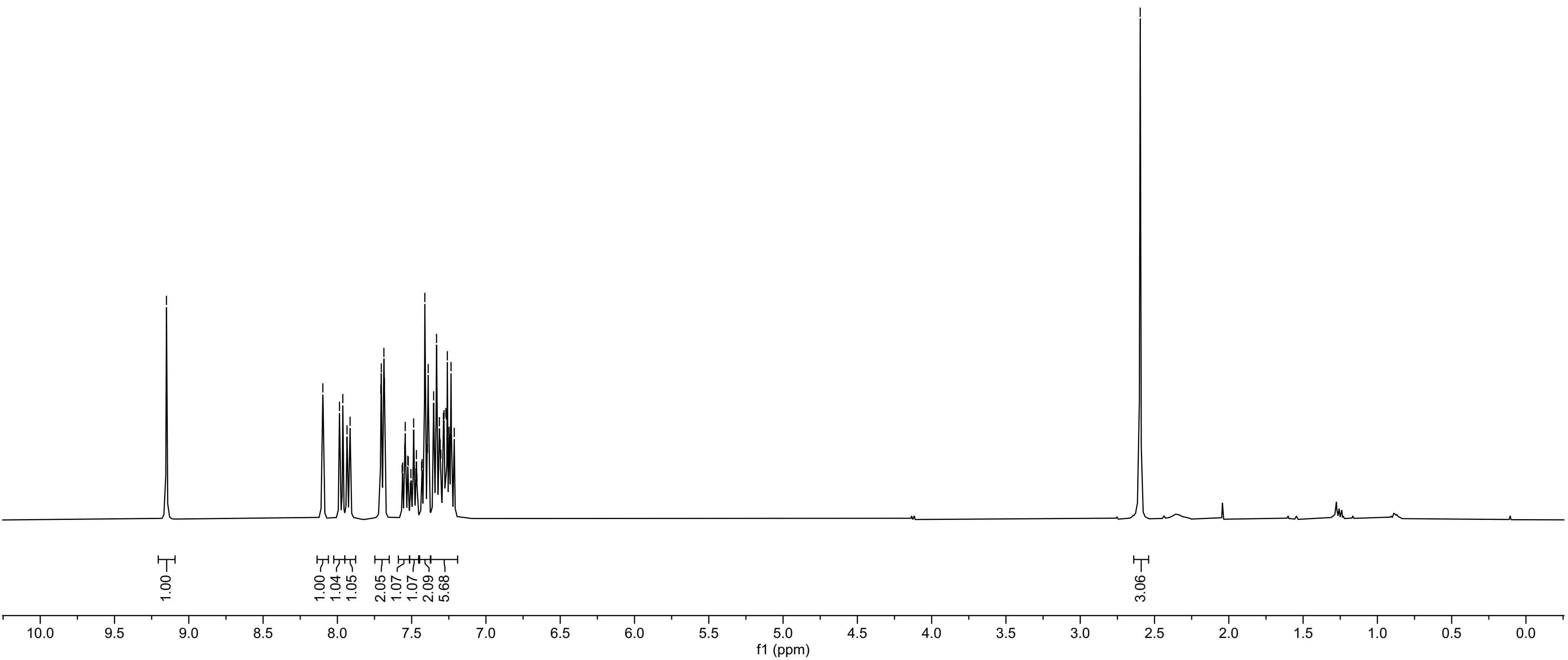

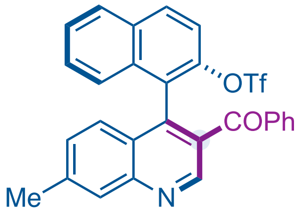

13C NMR of **3k** (101 MHz, $CDCl_3$)

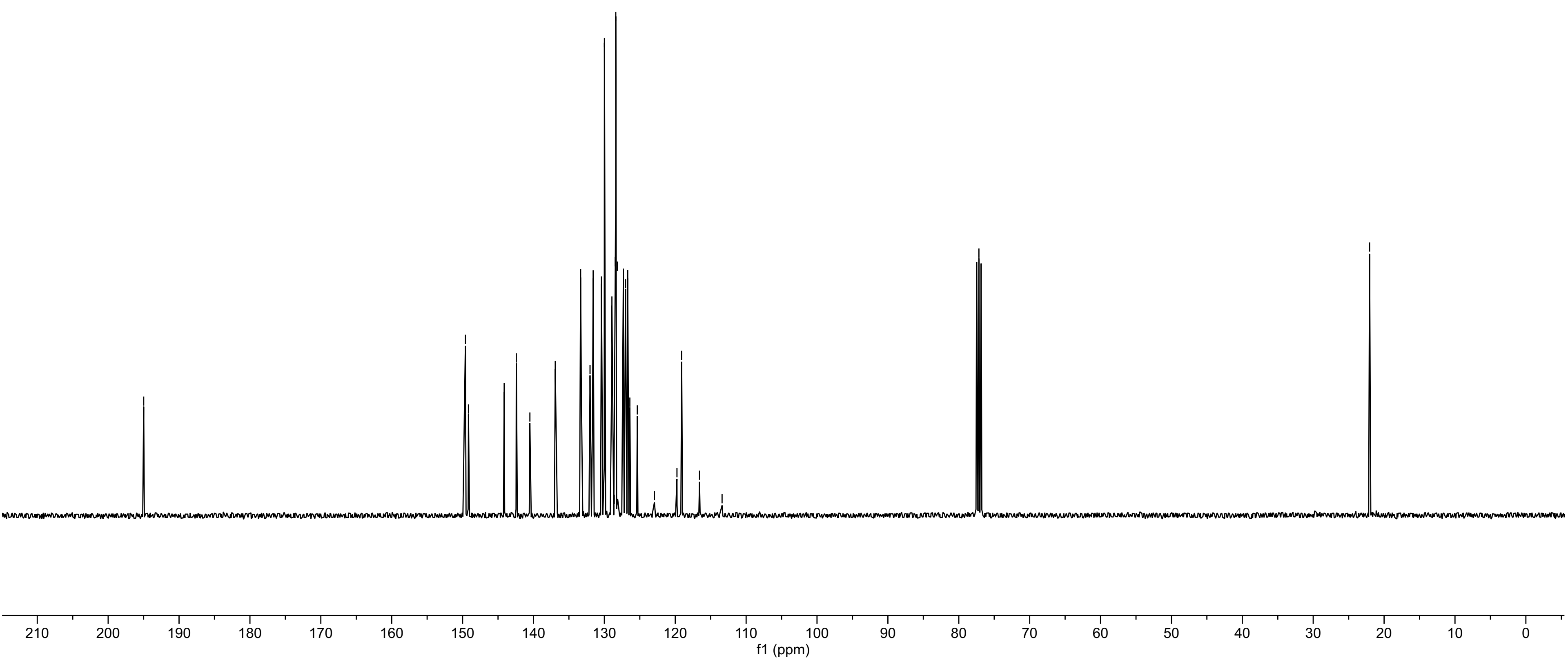

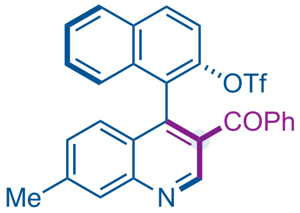

^{19}F NMR of **3k** (376 MHz, $CDCl_3$)

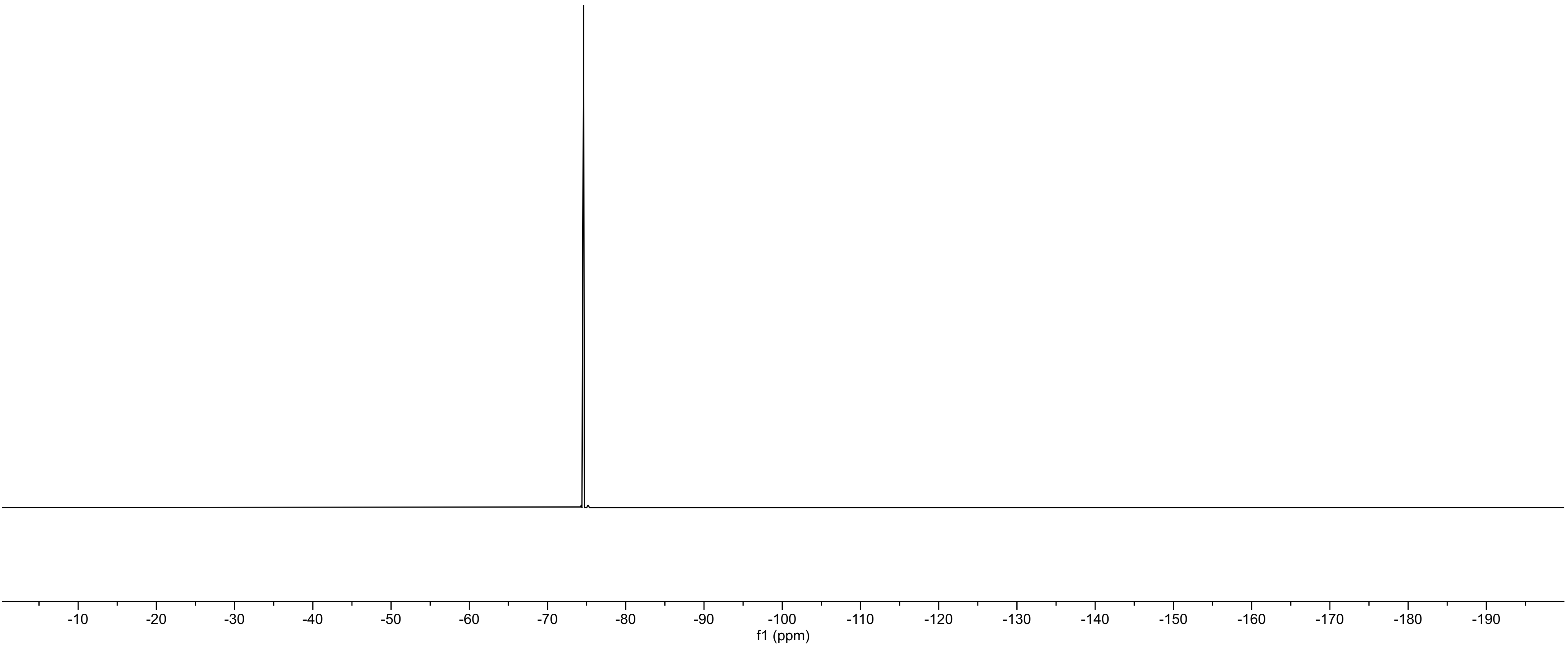

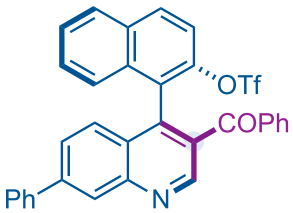

^{1}H NMR of **3l** (400 MHz, $CDCl_3$)

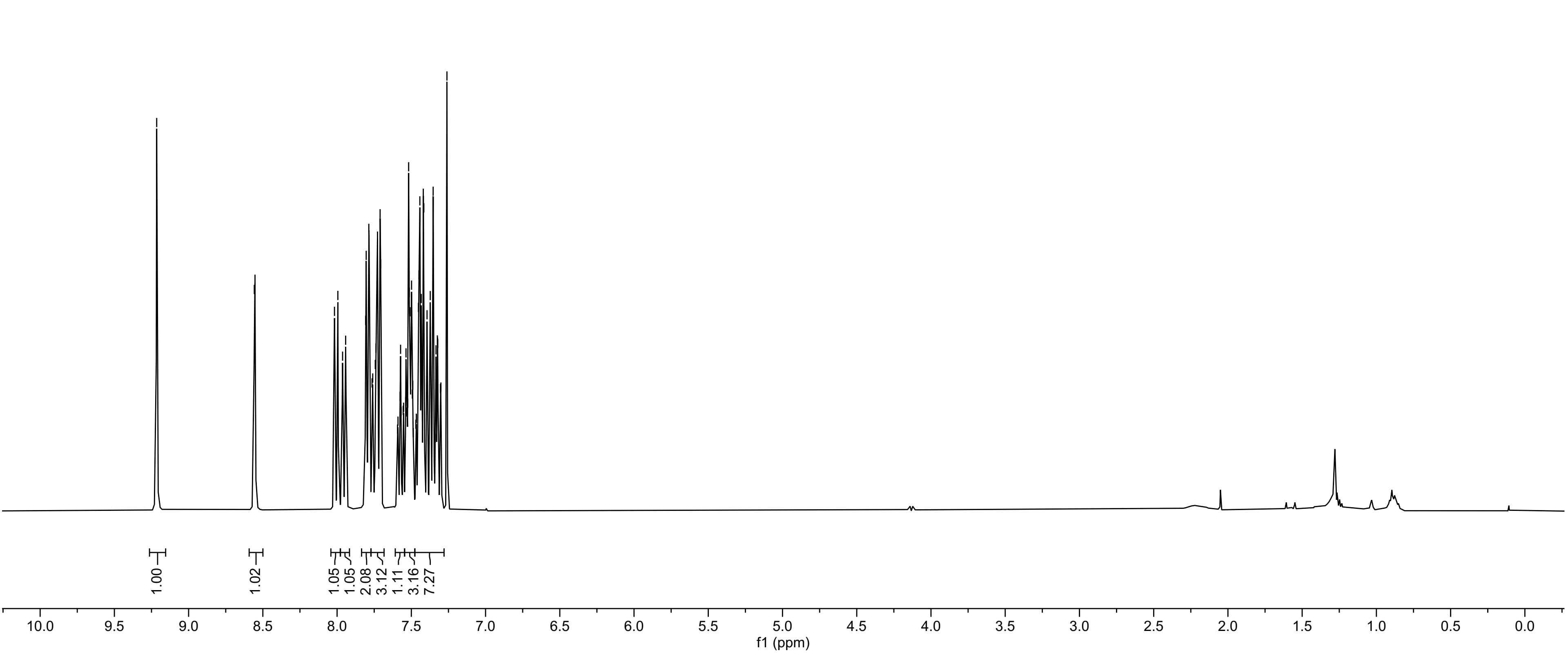

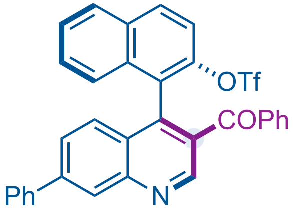

^{13}C NMR of 3l (101 MHz, $CDCl_3$)

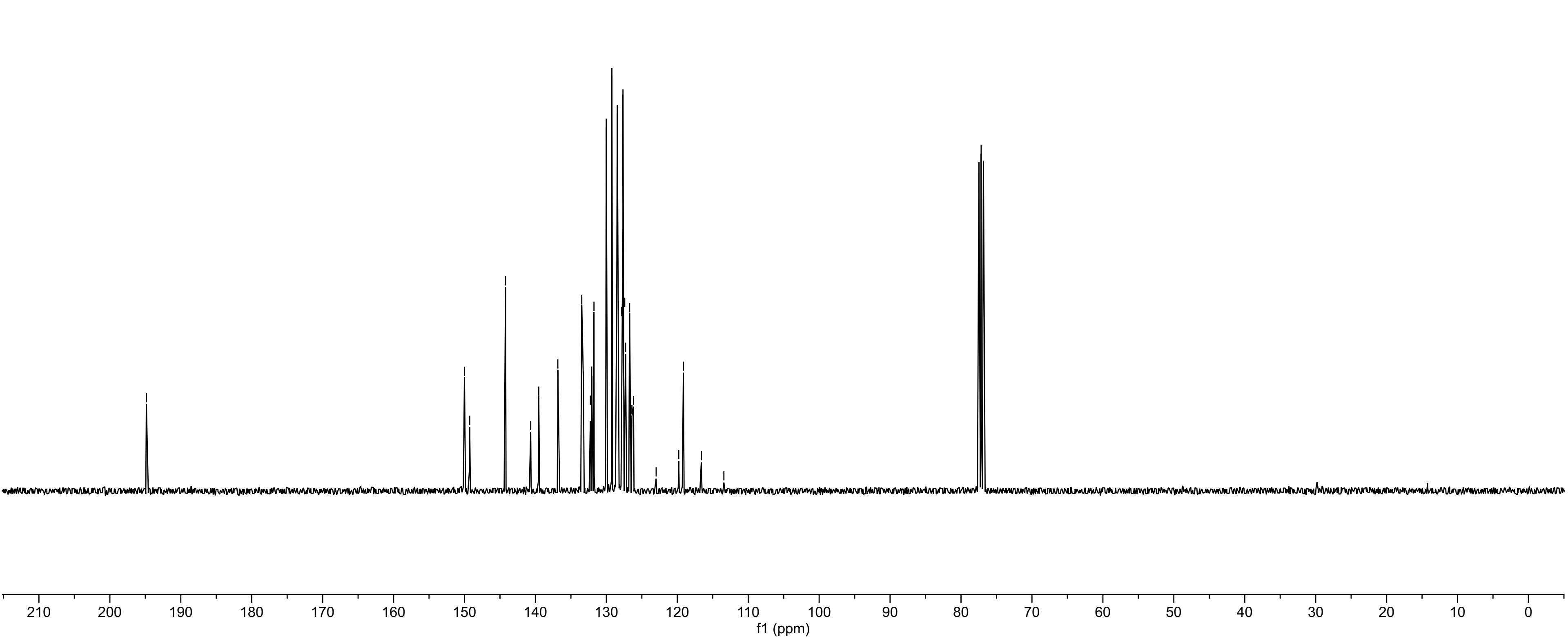

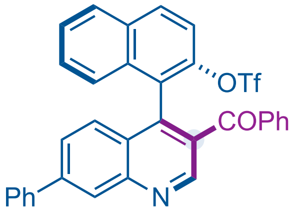

^{19}**F NMR** of **3l** (376 MHz, $CDCl_3$)

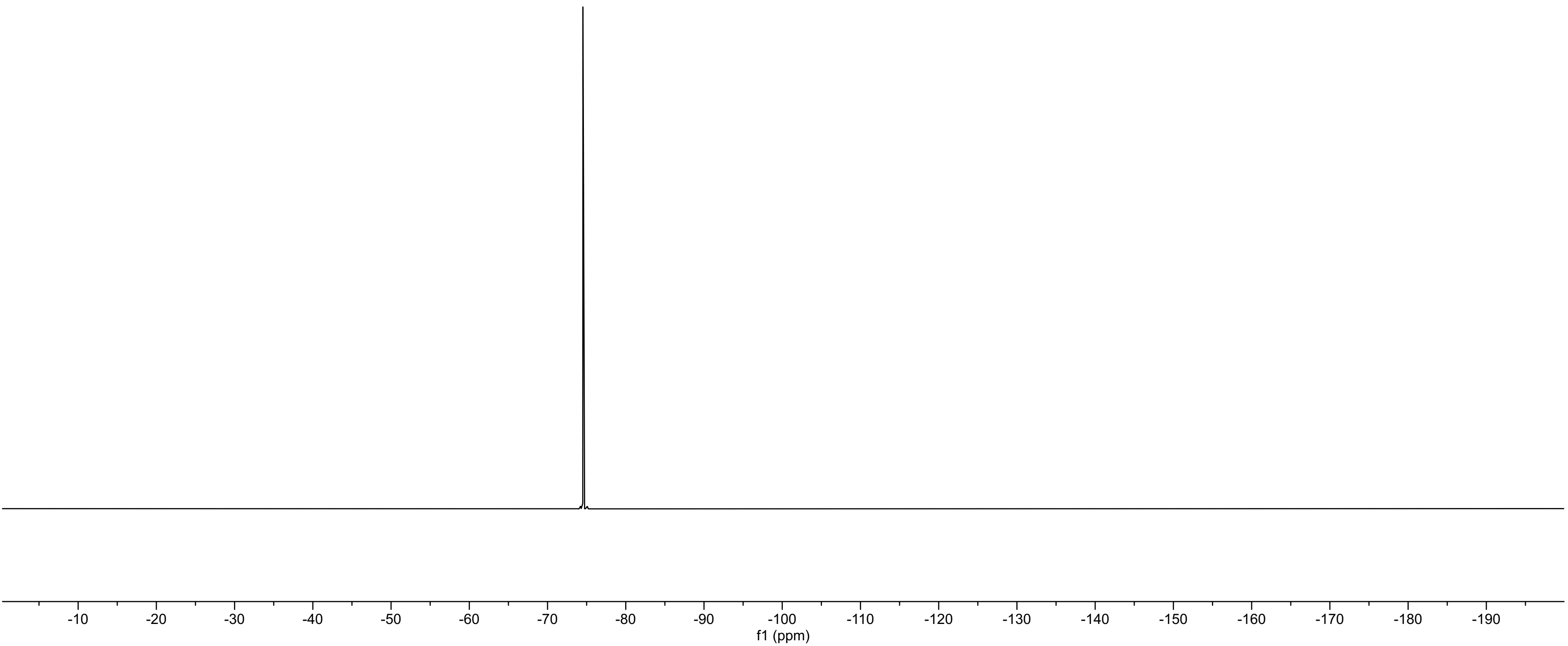

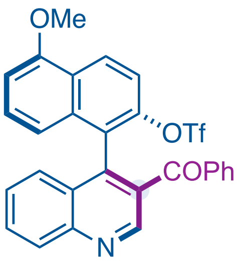

^{1}H NMR of **3o** (400 MHz, $CDCl_3$)

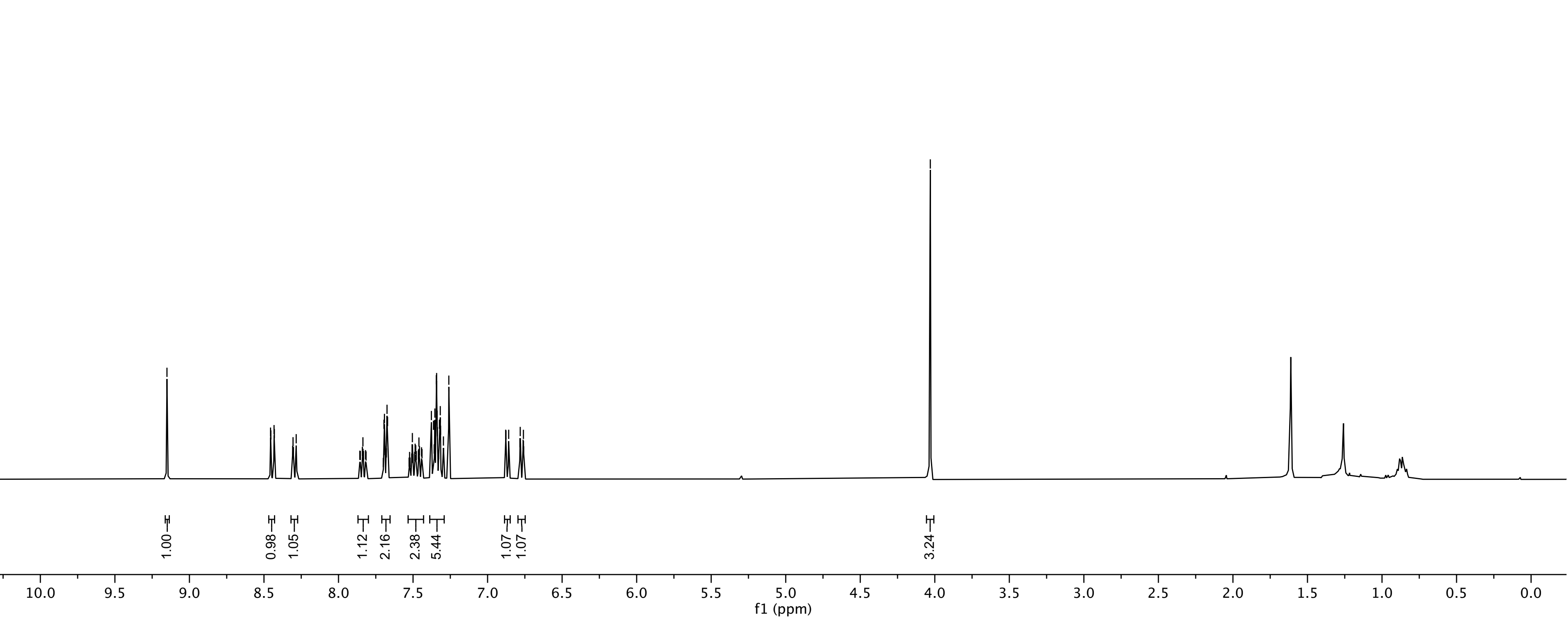

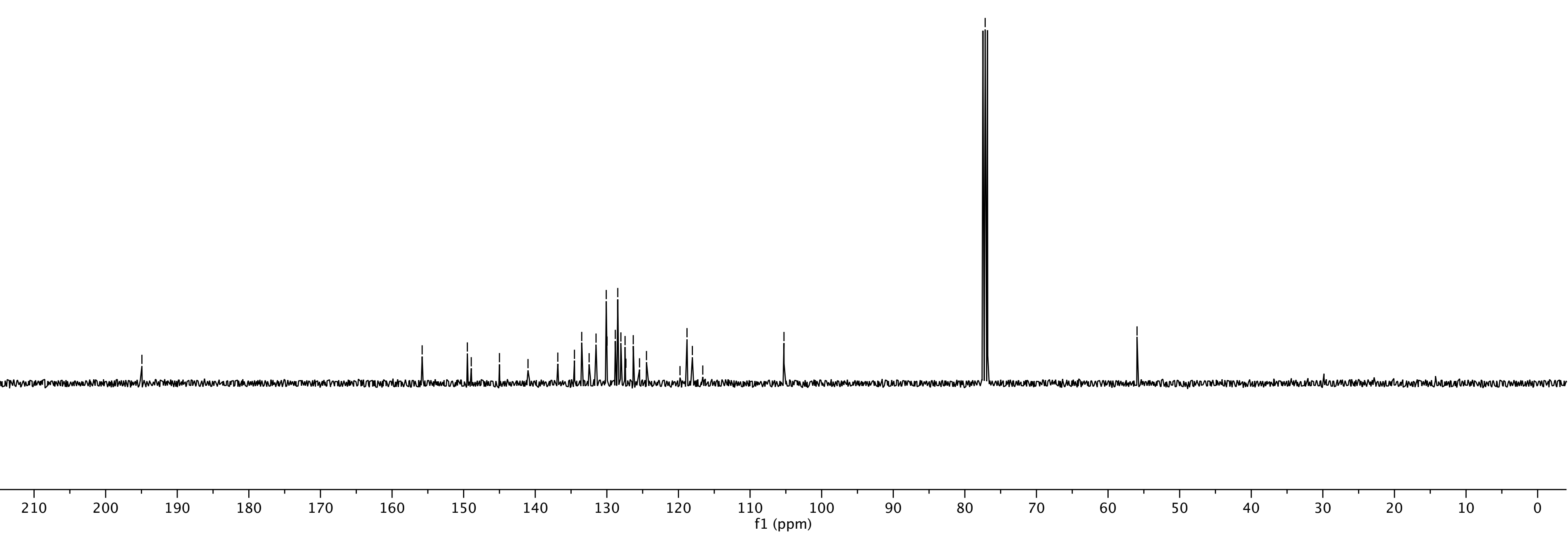

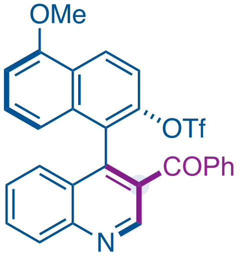

^{13}C NMR of **3o** (101 MHz, $CDCl_3$)

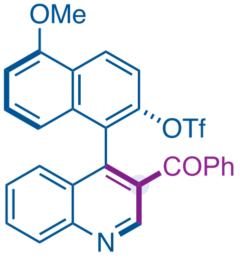

^{19}F NMR of **3o** (376 MHz, $CDCl_3$)

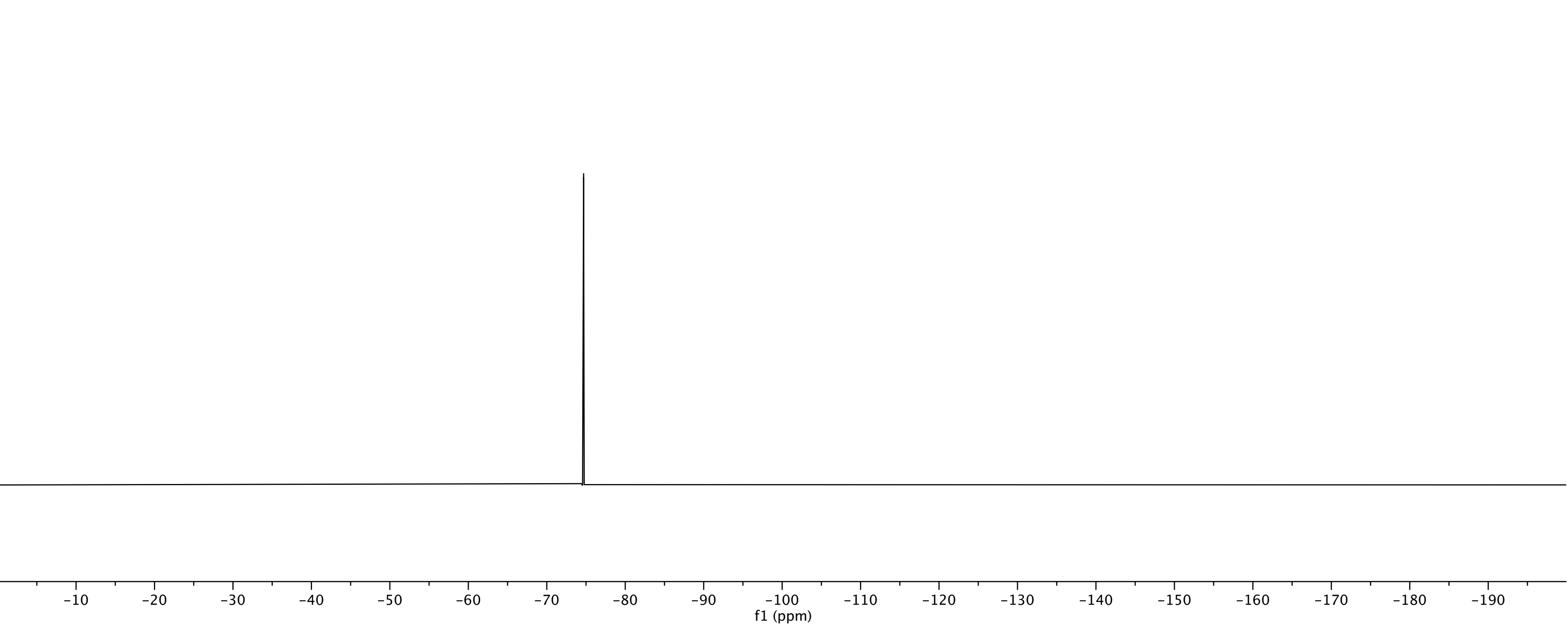

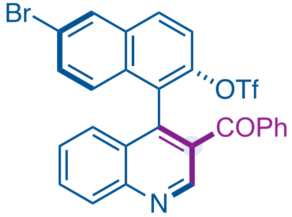

^{1}H NMR of **3p** (400 MHz, $CDCl_3$)

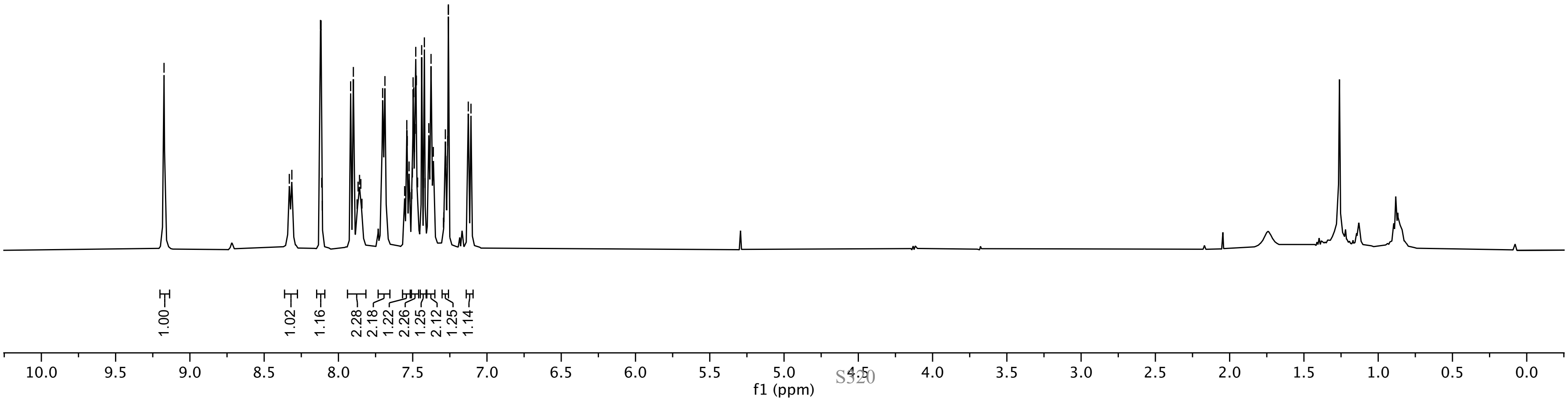

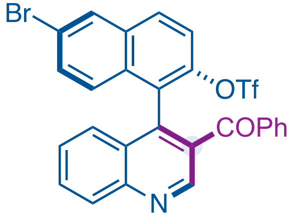

^{13}C NMR of **3p** (101 MHz, $CDCl_3$)

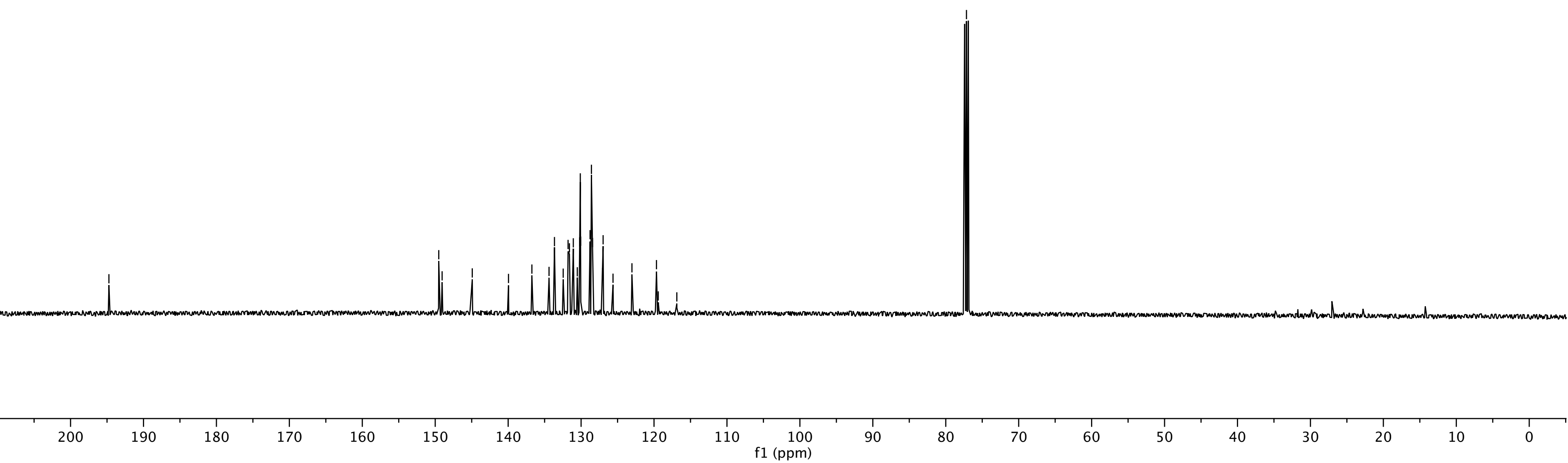

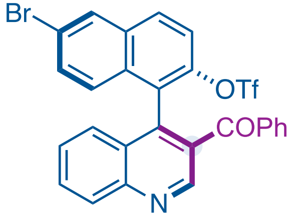

^{19}F NMR of **3p** (376 MHz, $CDCl_3$)

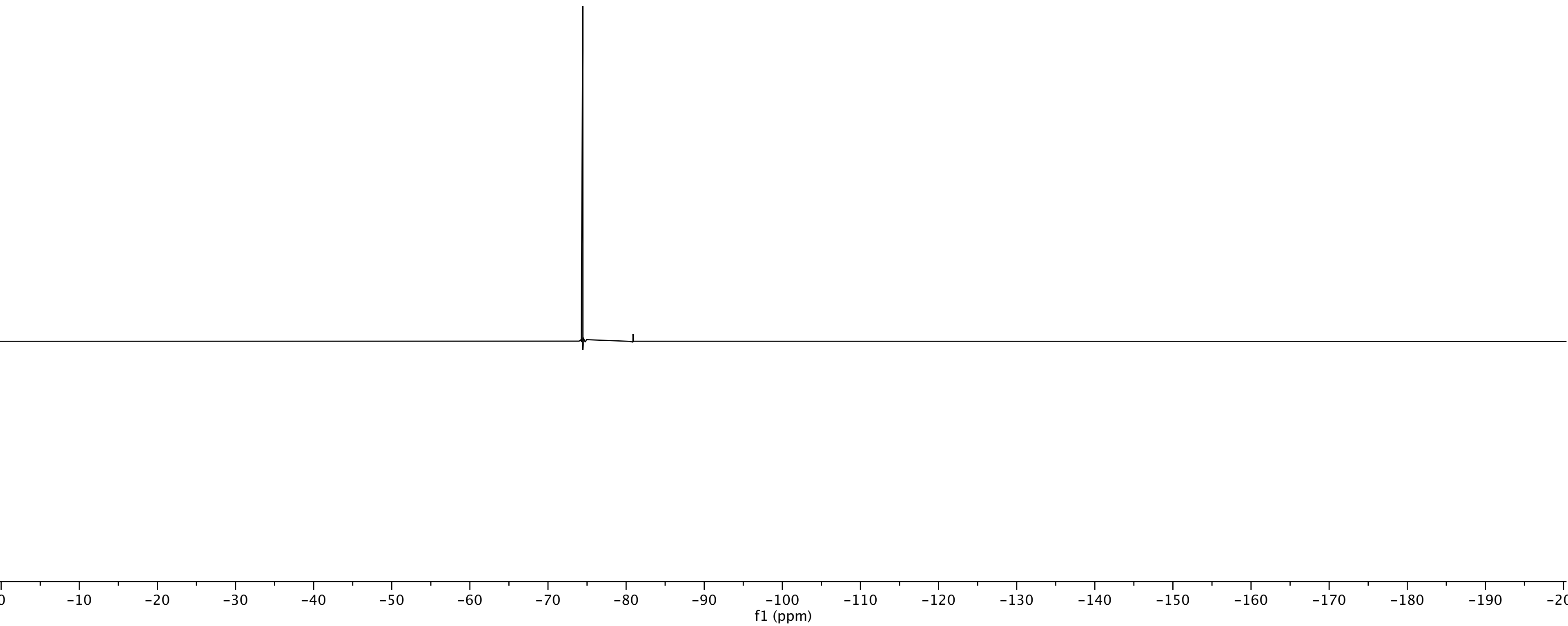

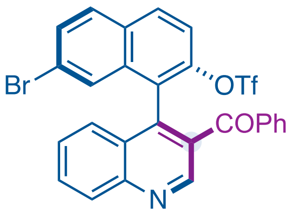

^{1}H NMR of **3q** (400 MHz, $CDCl_3$)

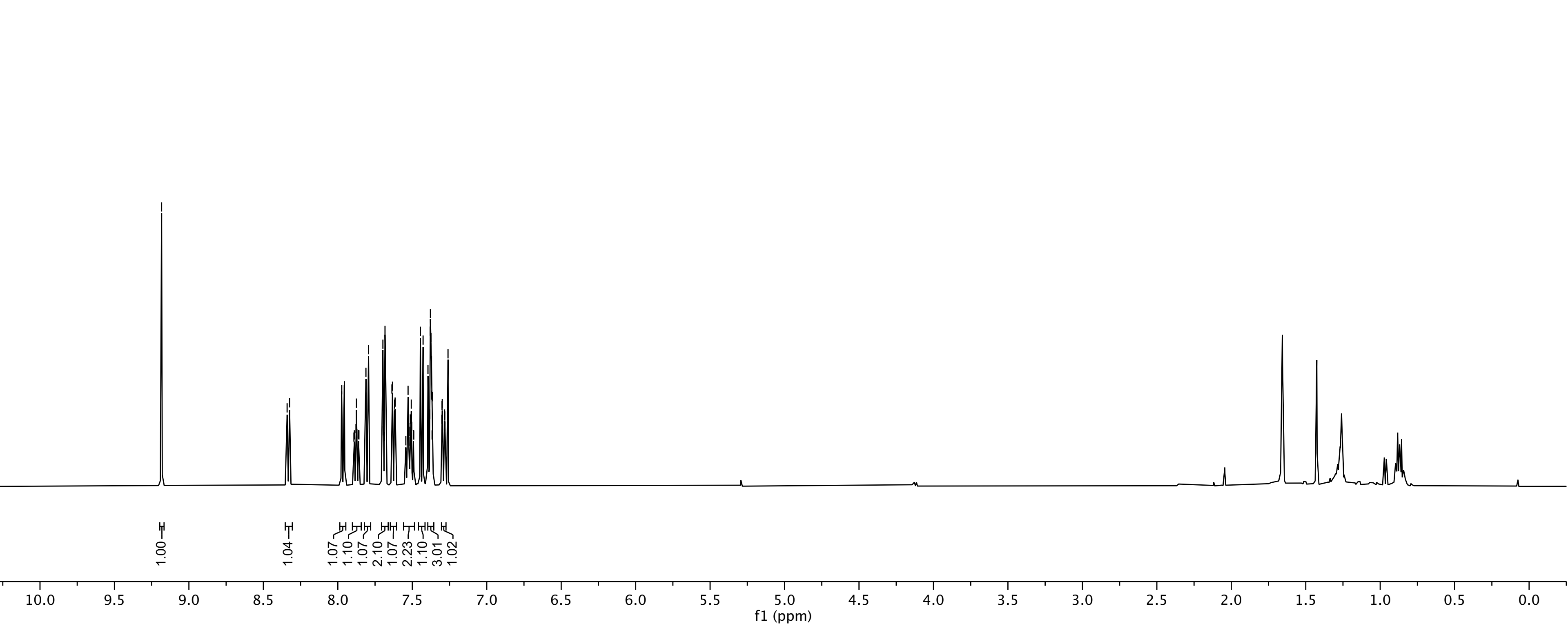

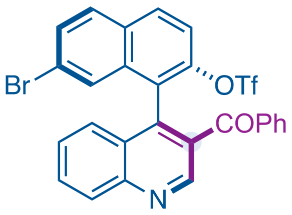

^{13}C NMR of **3q** (101 MHz, $CDCl_3$)

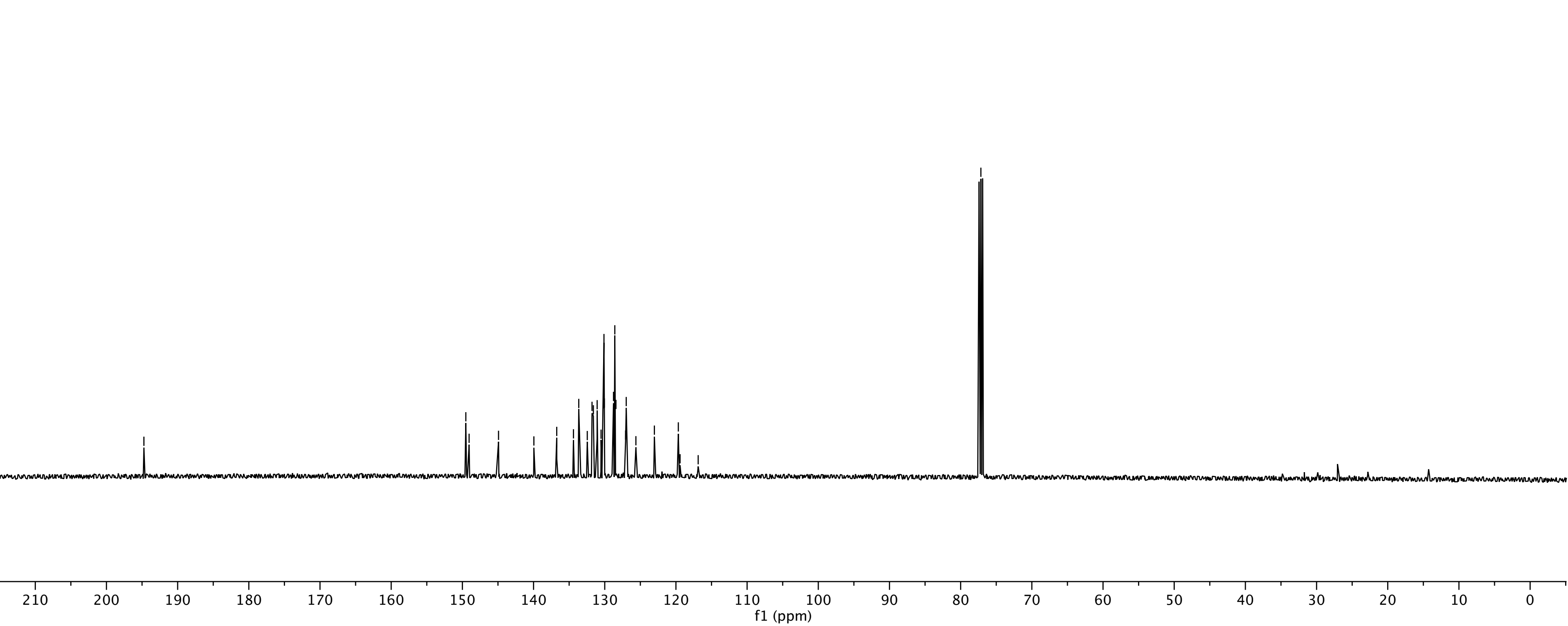

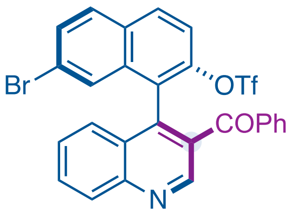

^{19}F NMR of **3q** (376 MHz, $CDCl_3$)

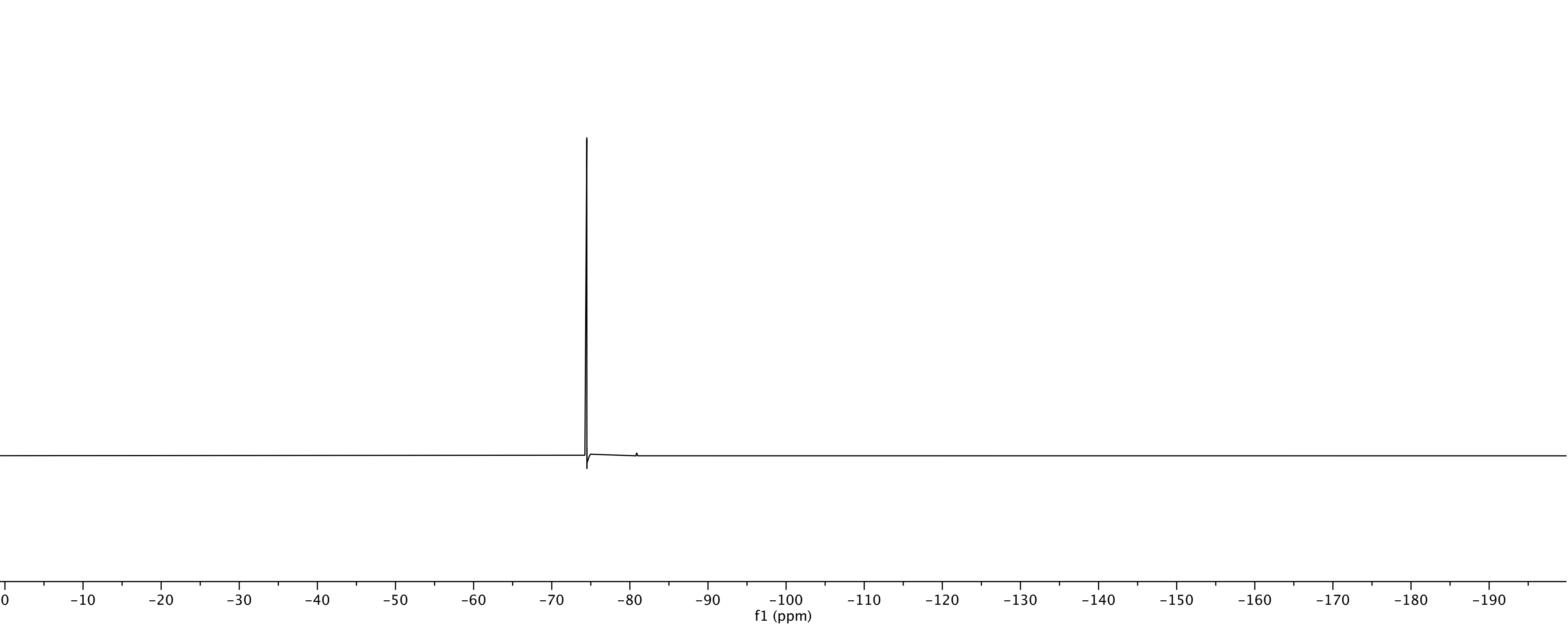

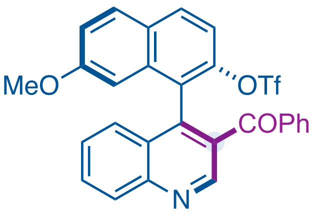

^{1}H NMR of **3r** (500 MHz, $CDCl_3$)

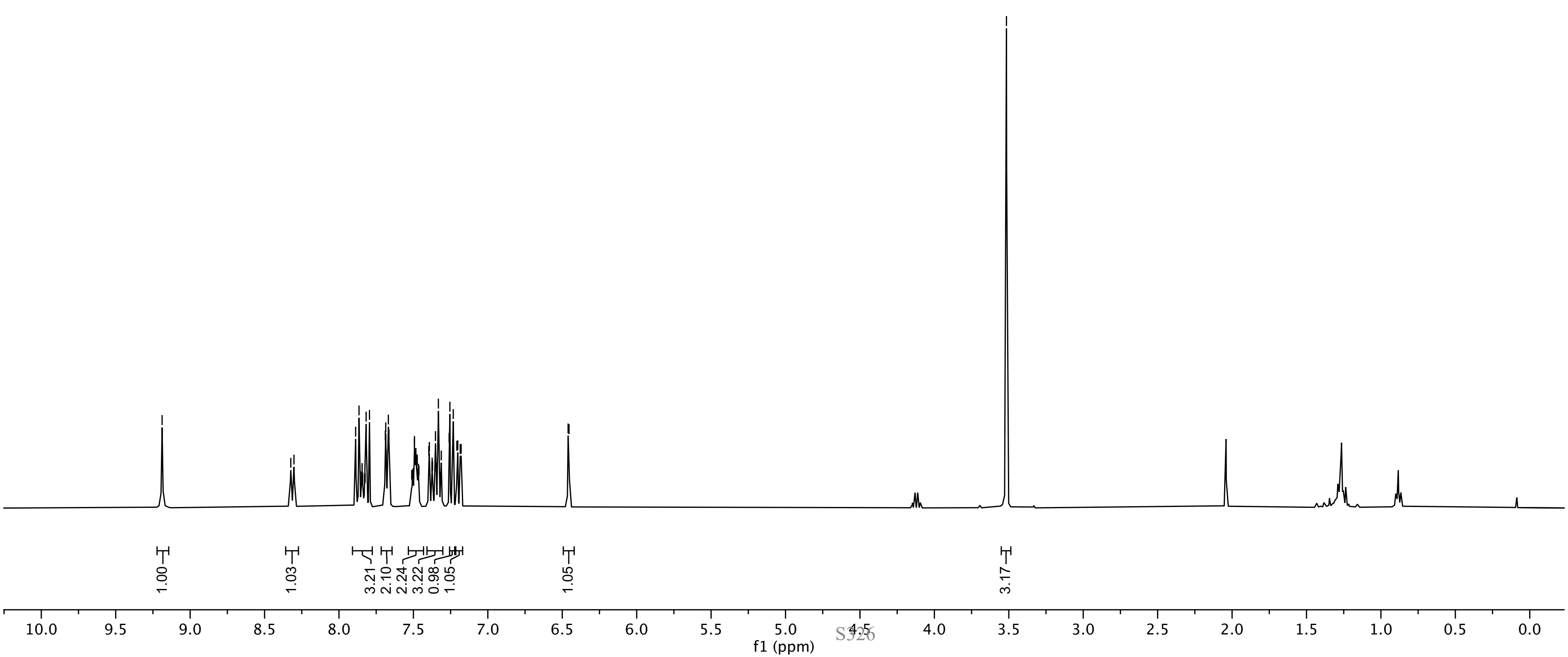

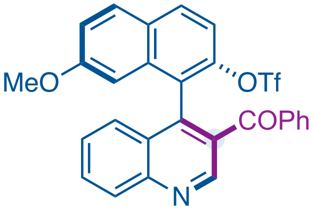

^{13}C NMR of **3r** (126 MHz, $CDCl_3$)

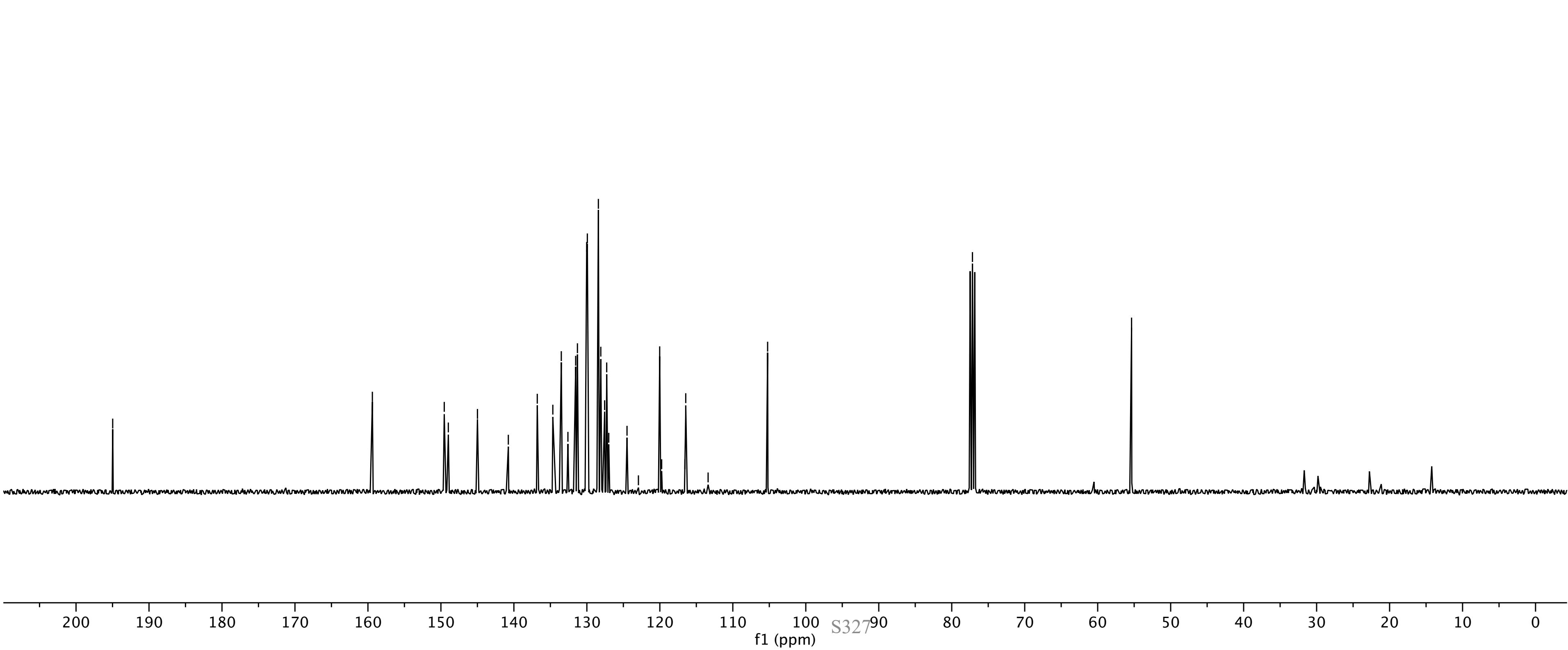

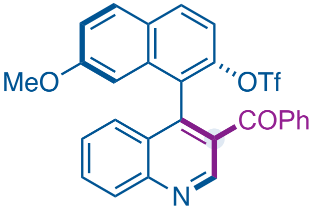

^{19}F NMR of **3r** (471 MHz, $CDCl_3$)

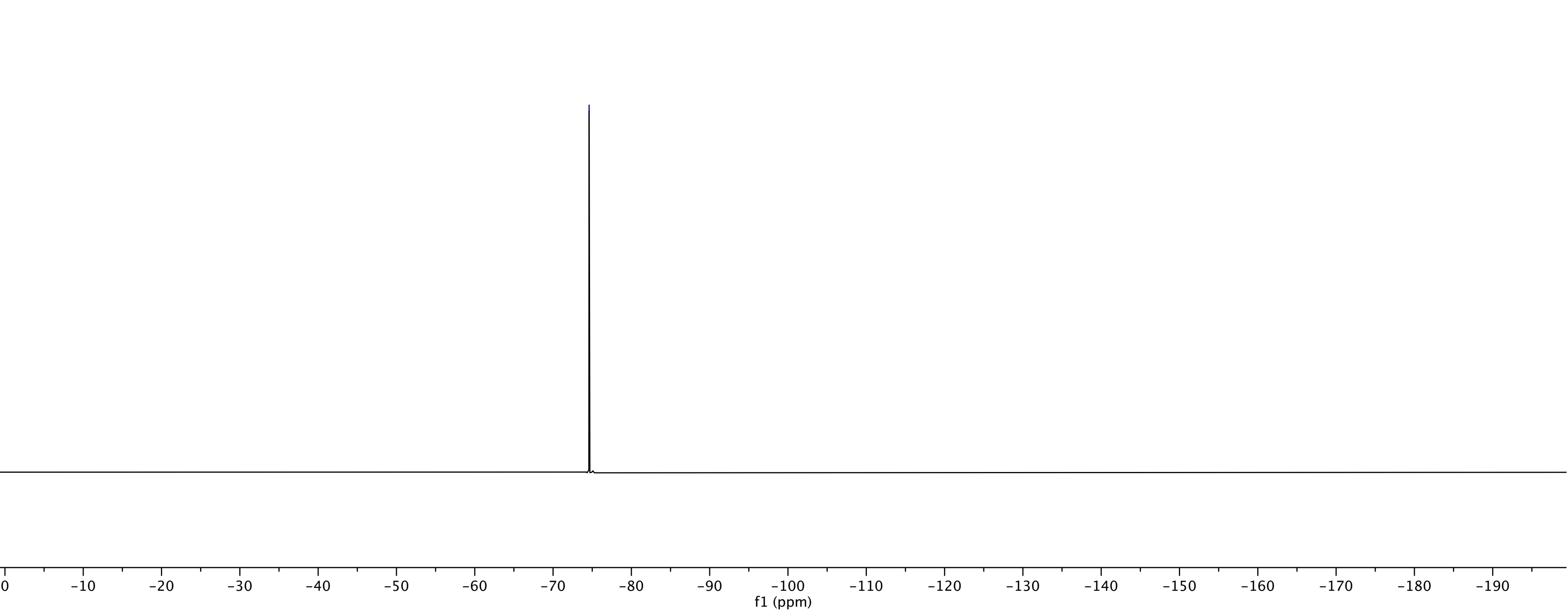

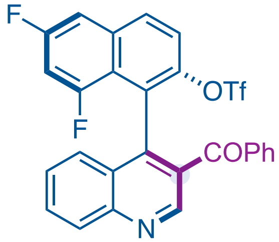

^{1}H NMR of 3s (400 MHz, $CDCl_3$)

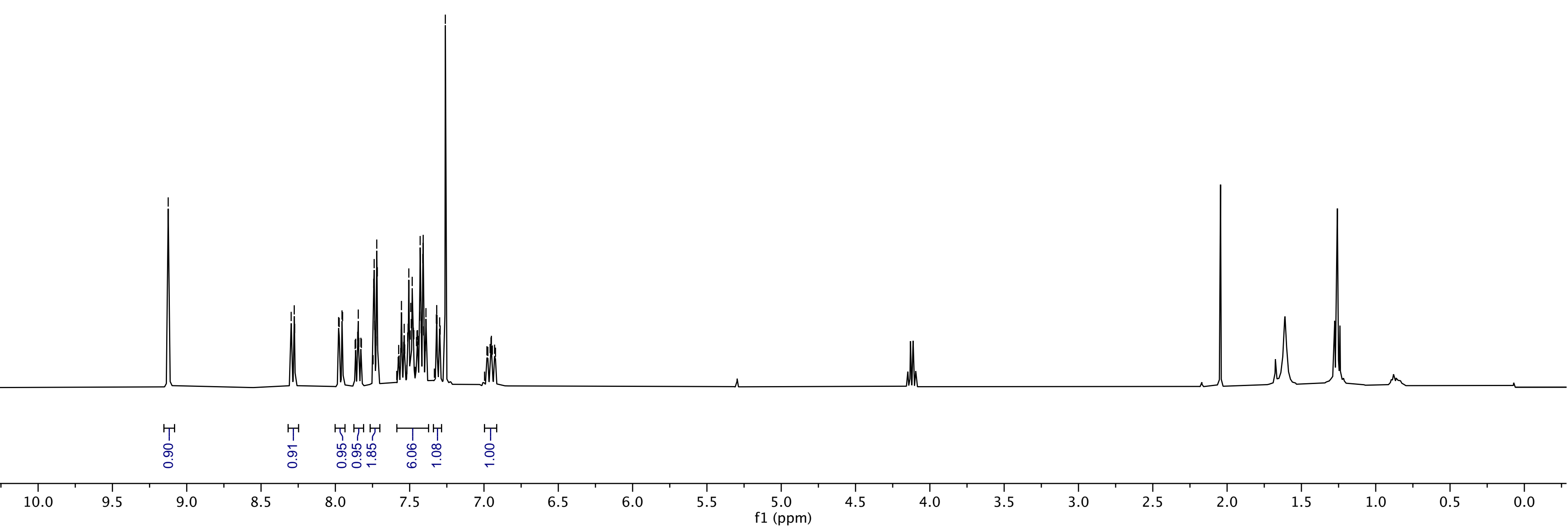

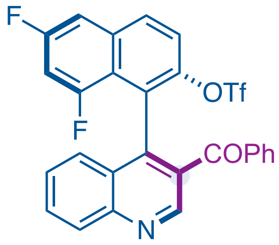

^{13}C NMR of **3s** (101 MHz, $CDCl_3$)

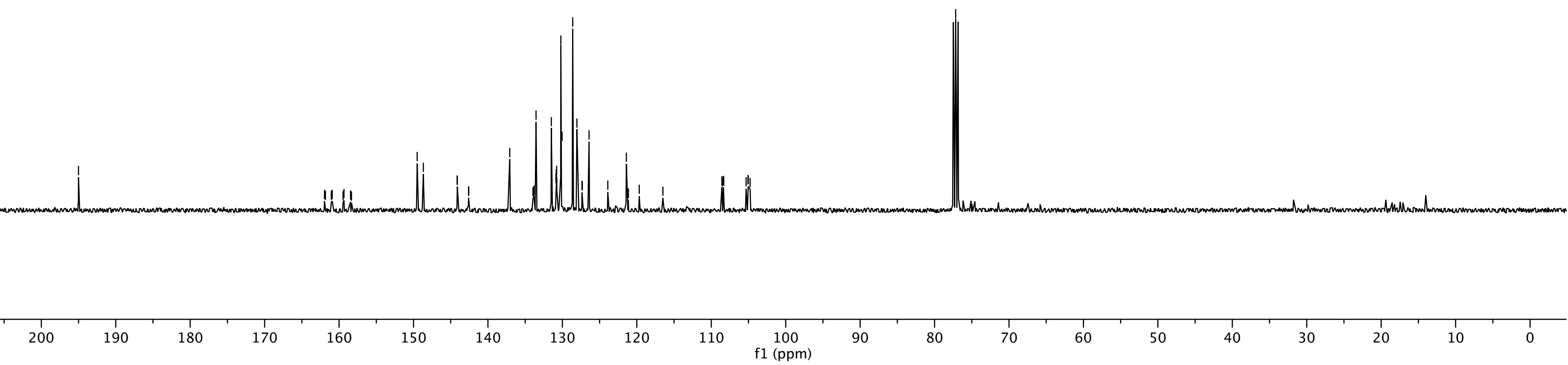

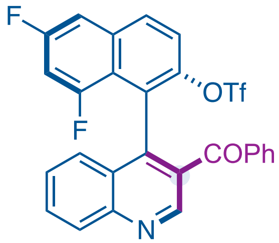

^{19}F NMR of **3s** (376 MHz, $CDCl_3$)

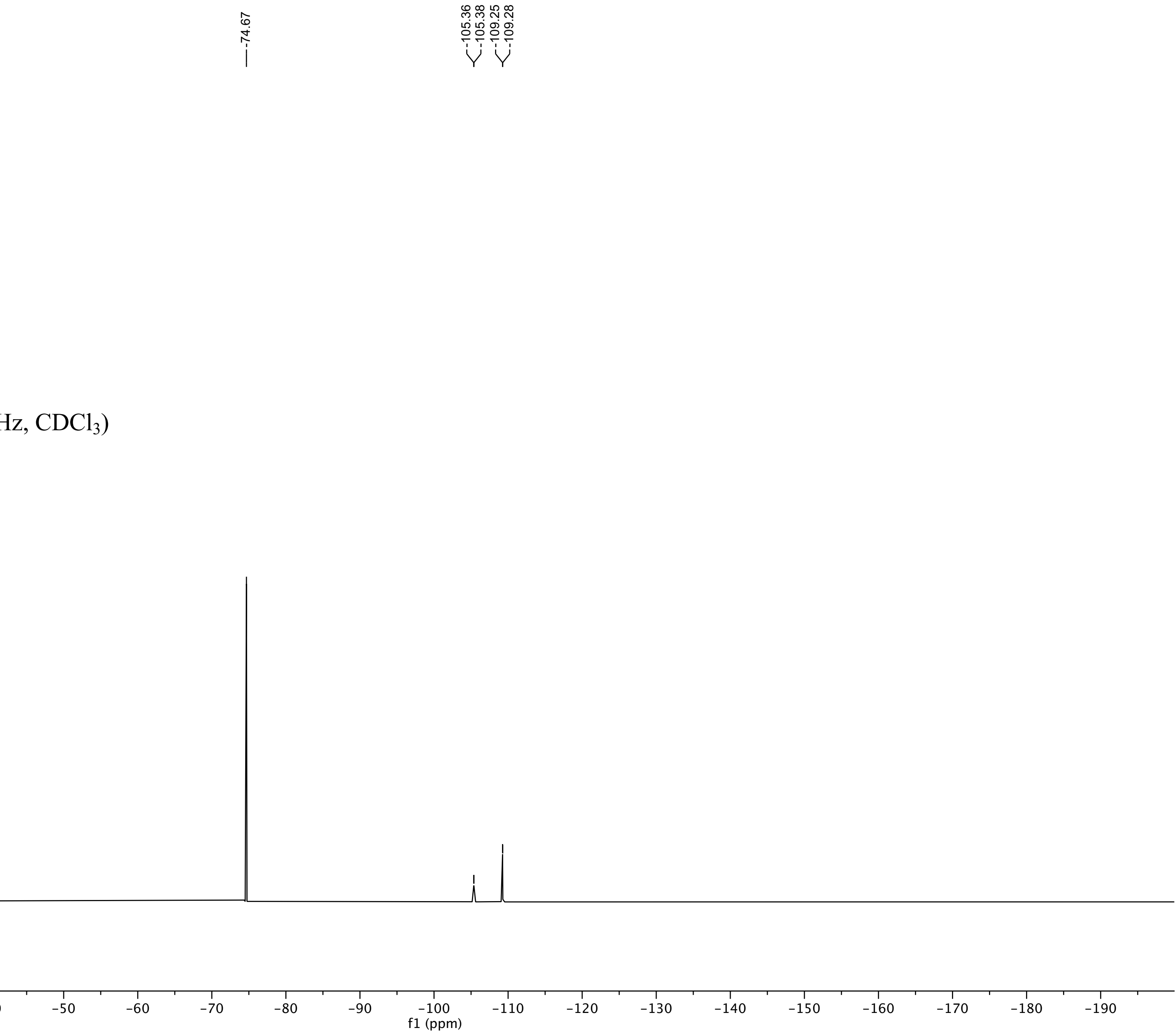

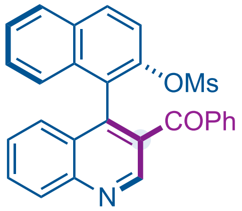

^{1}H NMR of **3t** (400 MHz, $CDCl_3$)

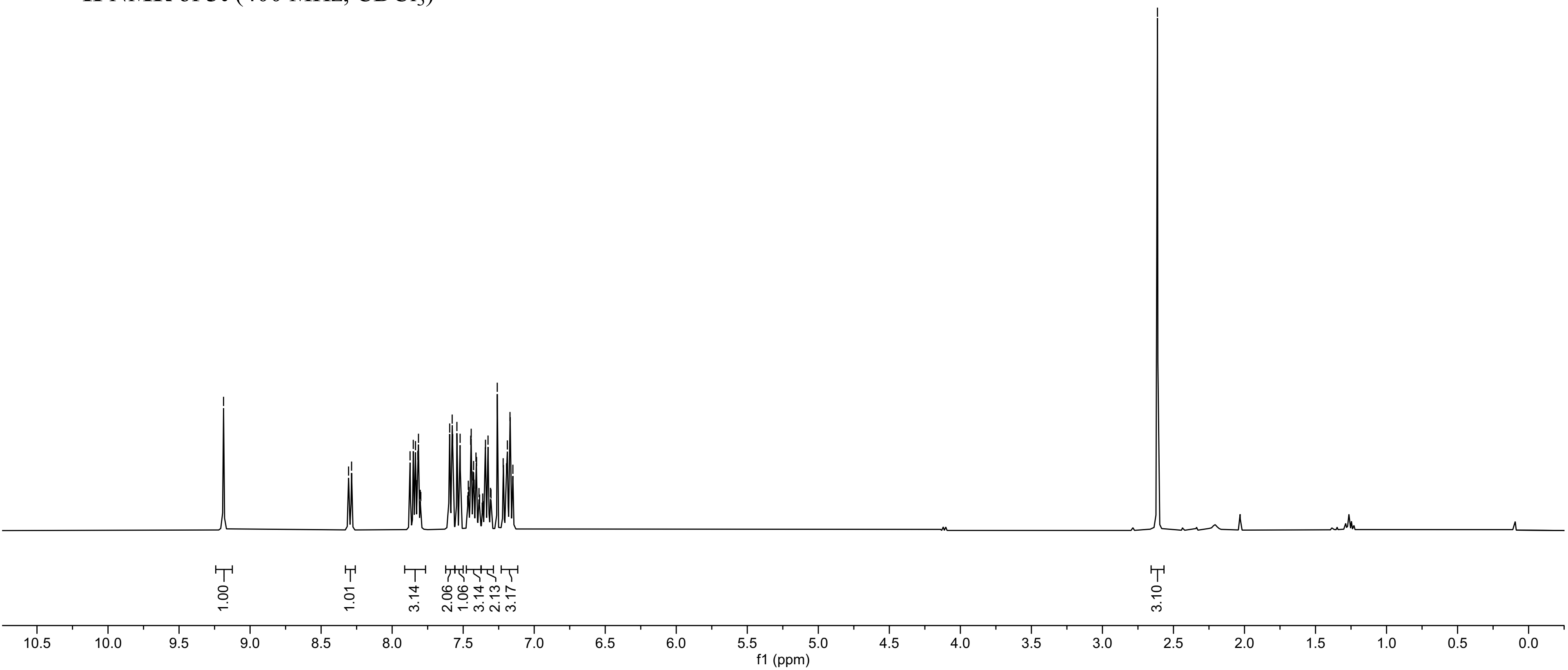

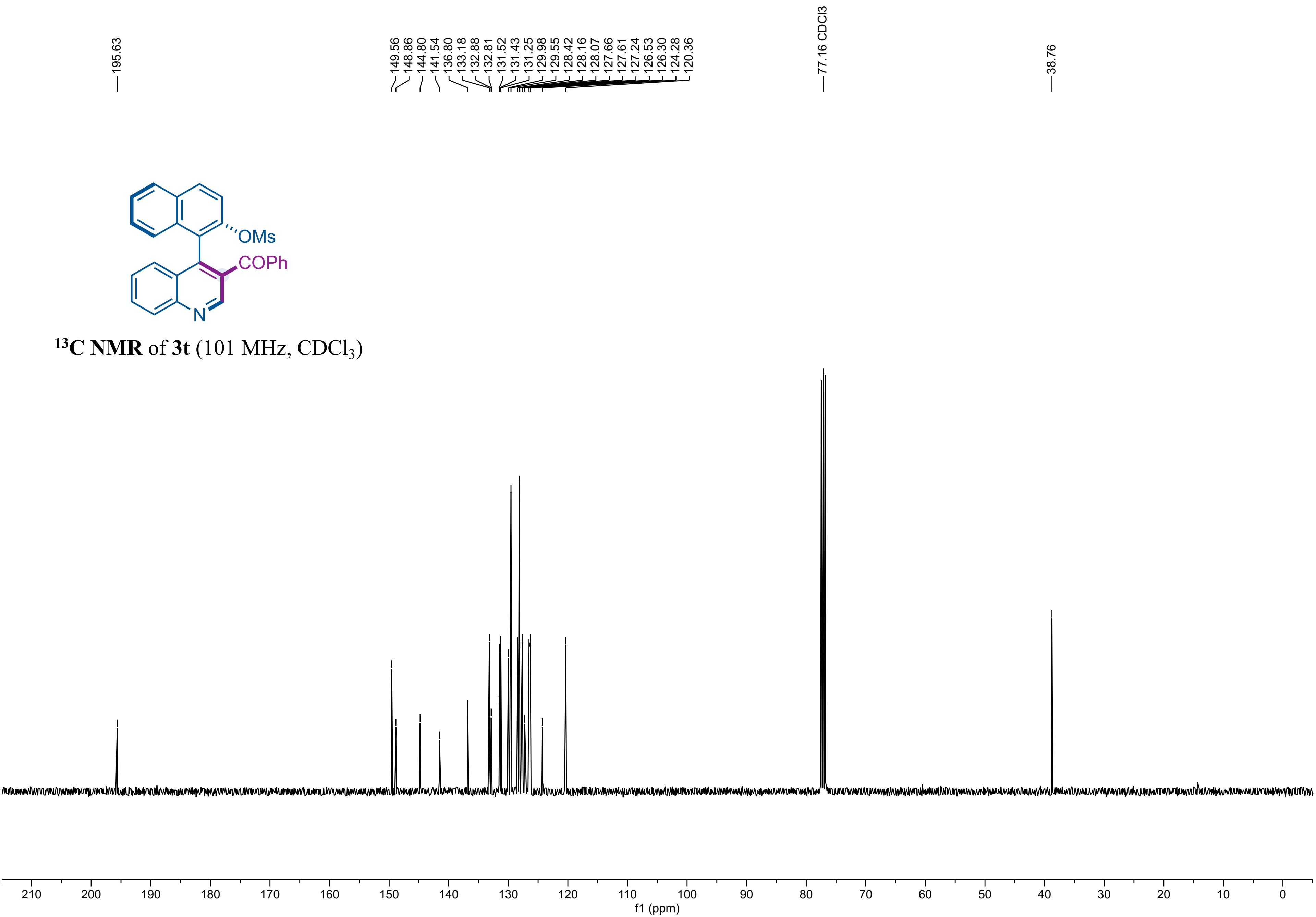

^{13}C NMR of **3t** (101 MHz, $CDCl_3$)

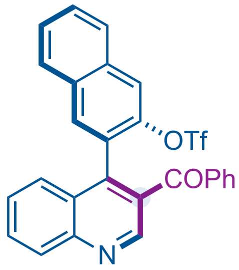

^{1}**H NMR** of **3u** (400 MHz, $CDCl_3$)

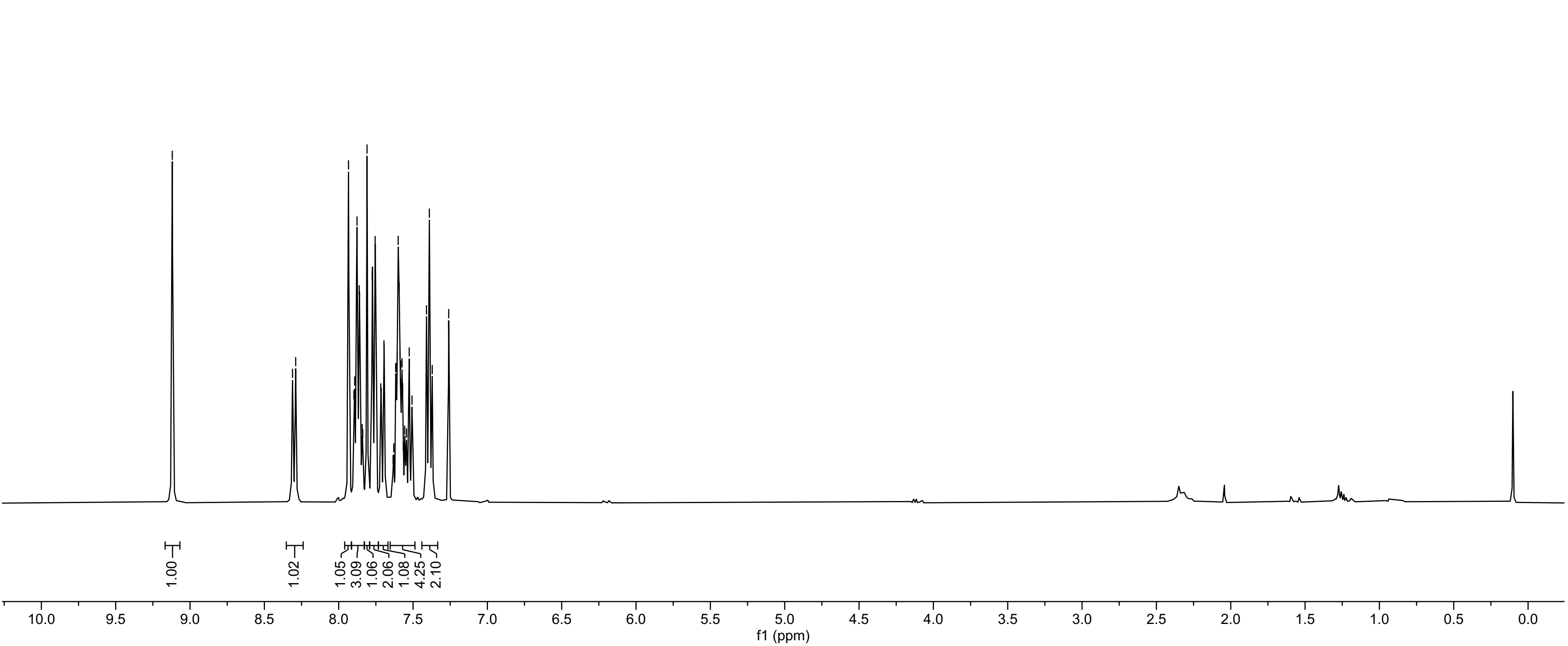

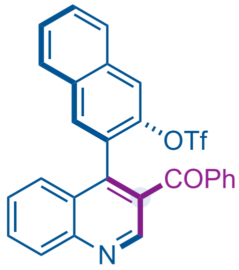

^{13}C NMR of **3u** (101 MHz, $CDCl_3$)

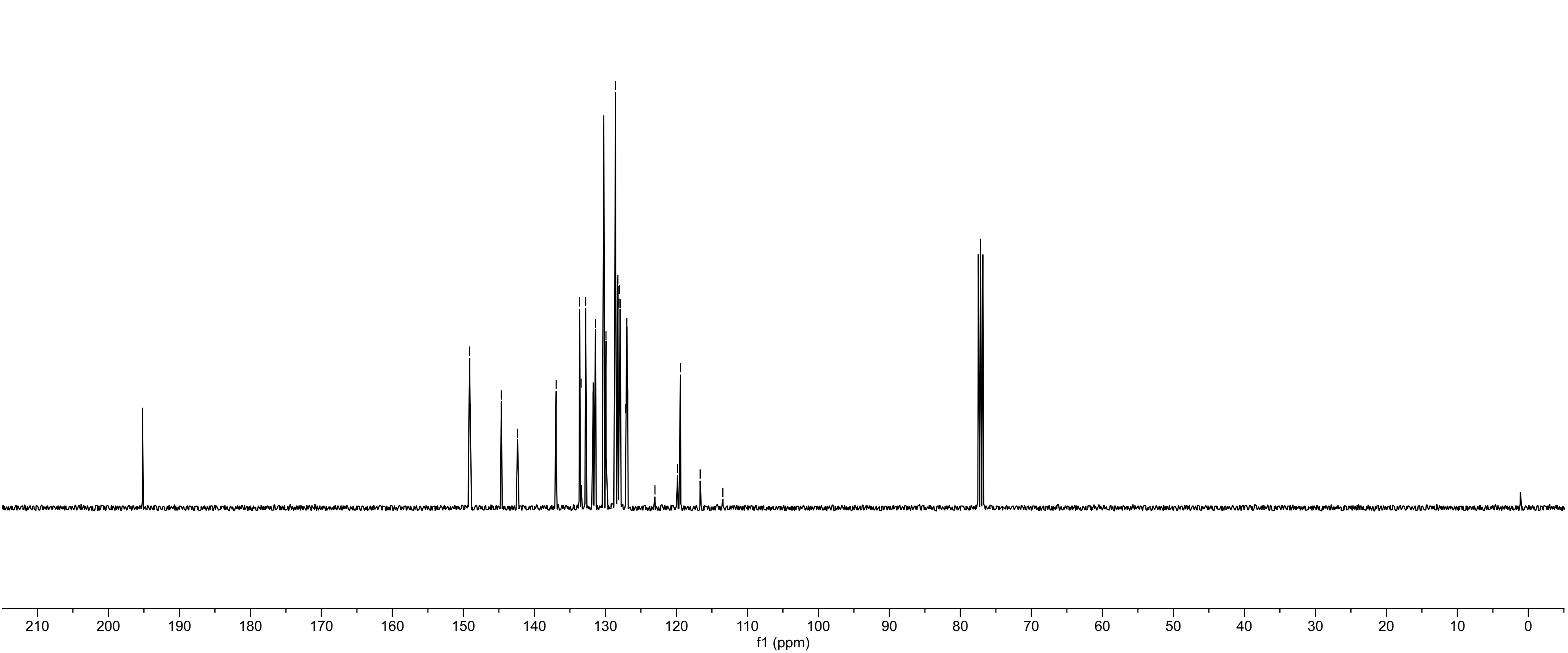

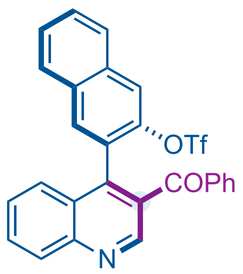

^{19}**F NMR** of **3u** (376 MHz, $CDCl_3$)

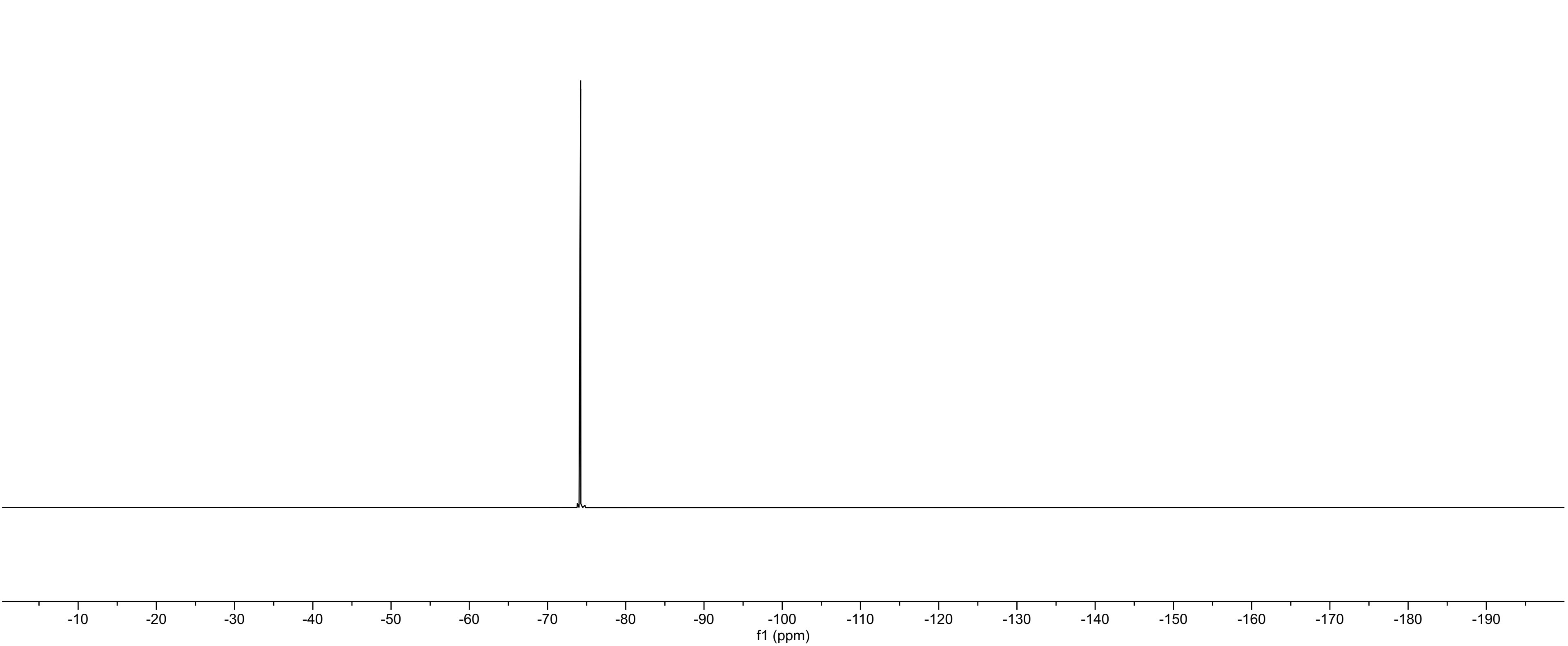

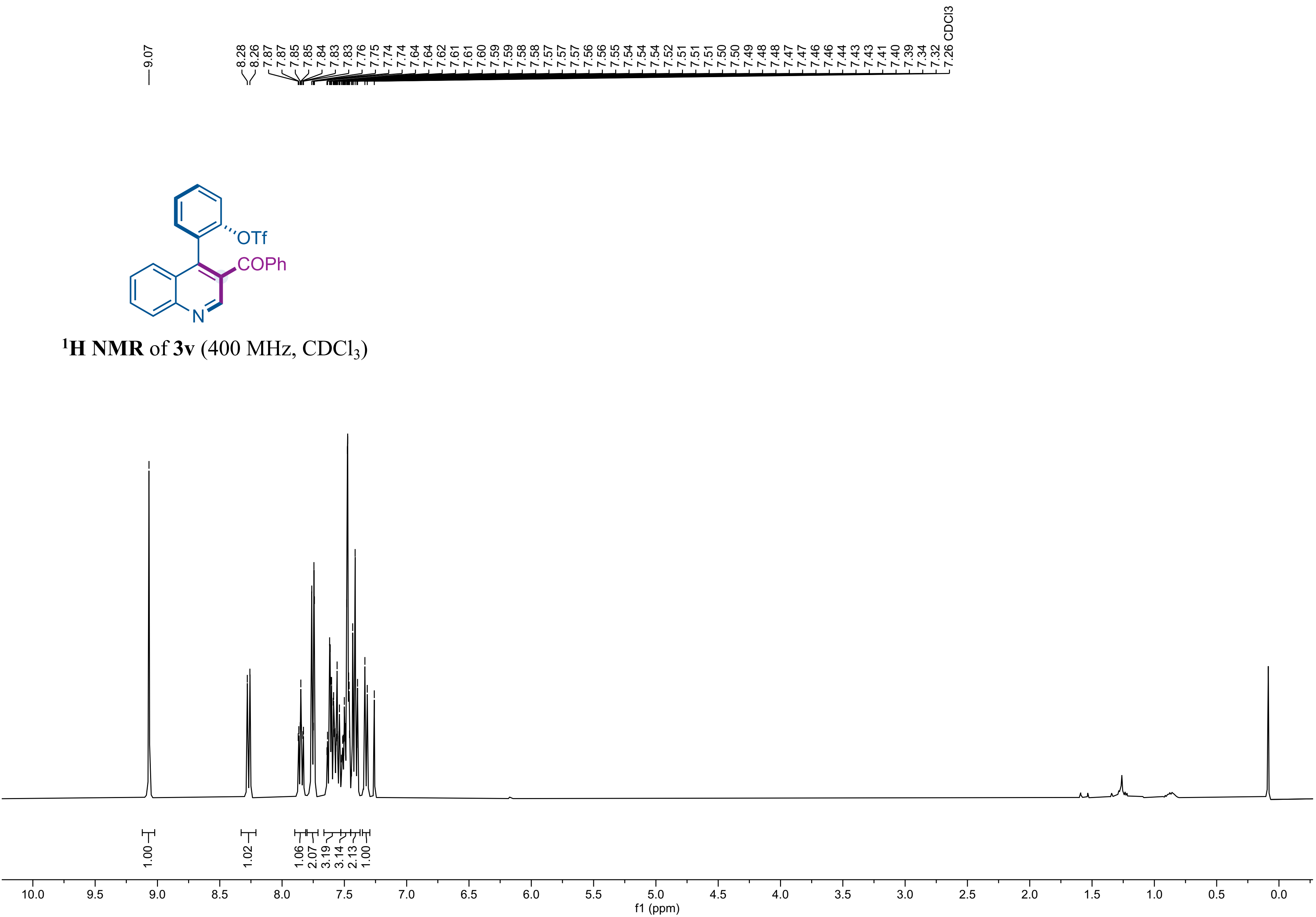

[1]**H NMR** of **3v** (400 MHz, $CDCl_3$)

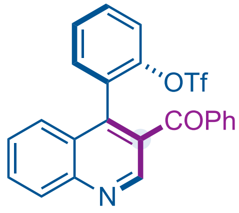

^{13}C NMR of **3v** (101 MHz, $CDCl_3$)

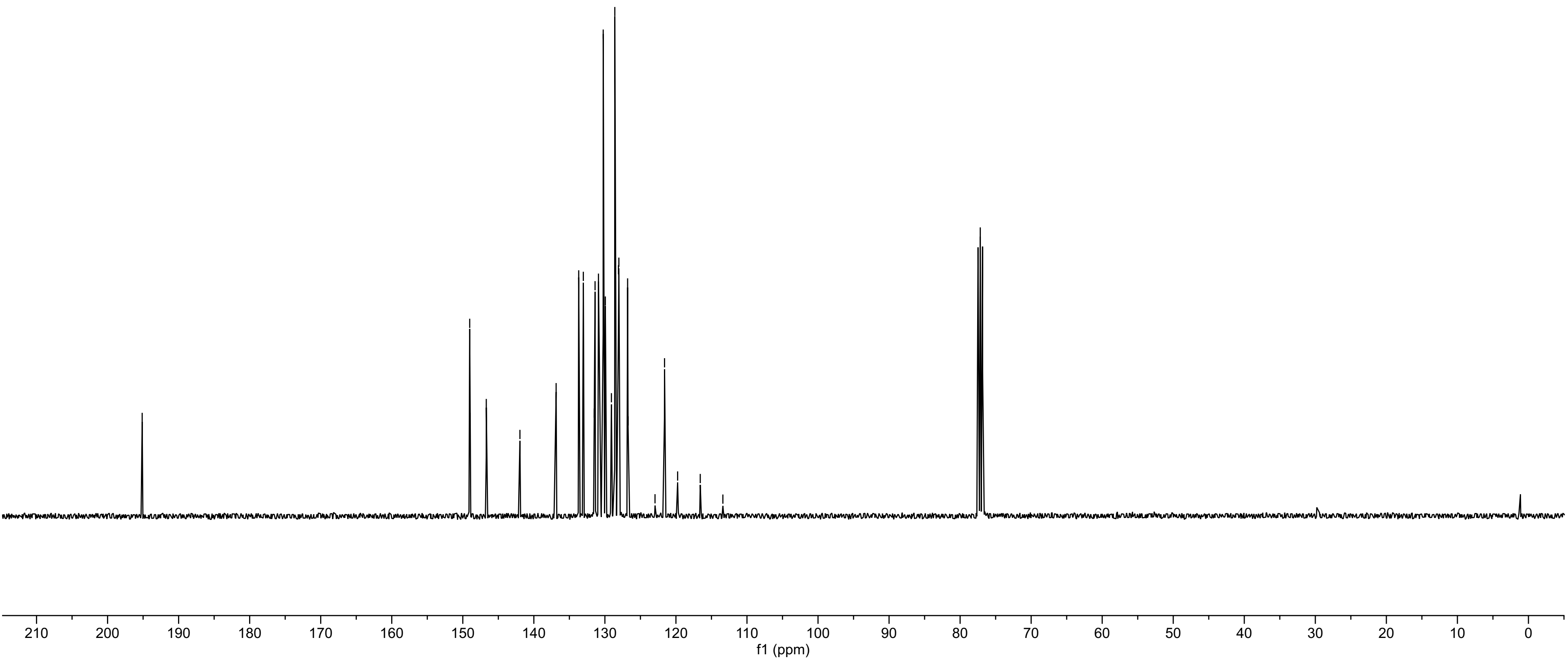

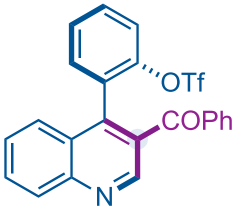

^{19}**F NMR** of **3v** (376 MHz, $CDCl_3$)

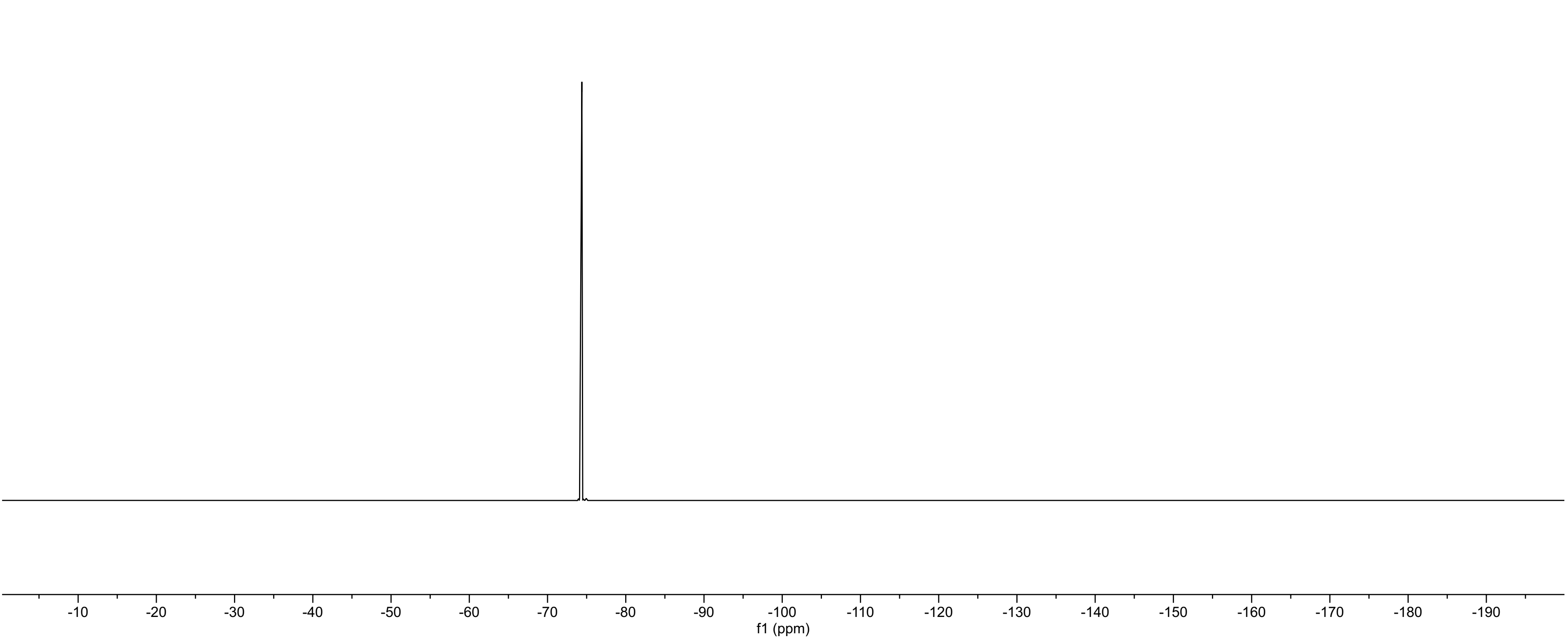

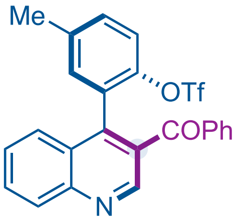

^{1}H NMR of **3w** (400 MHz, $CDCl_3$)

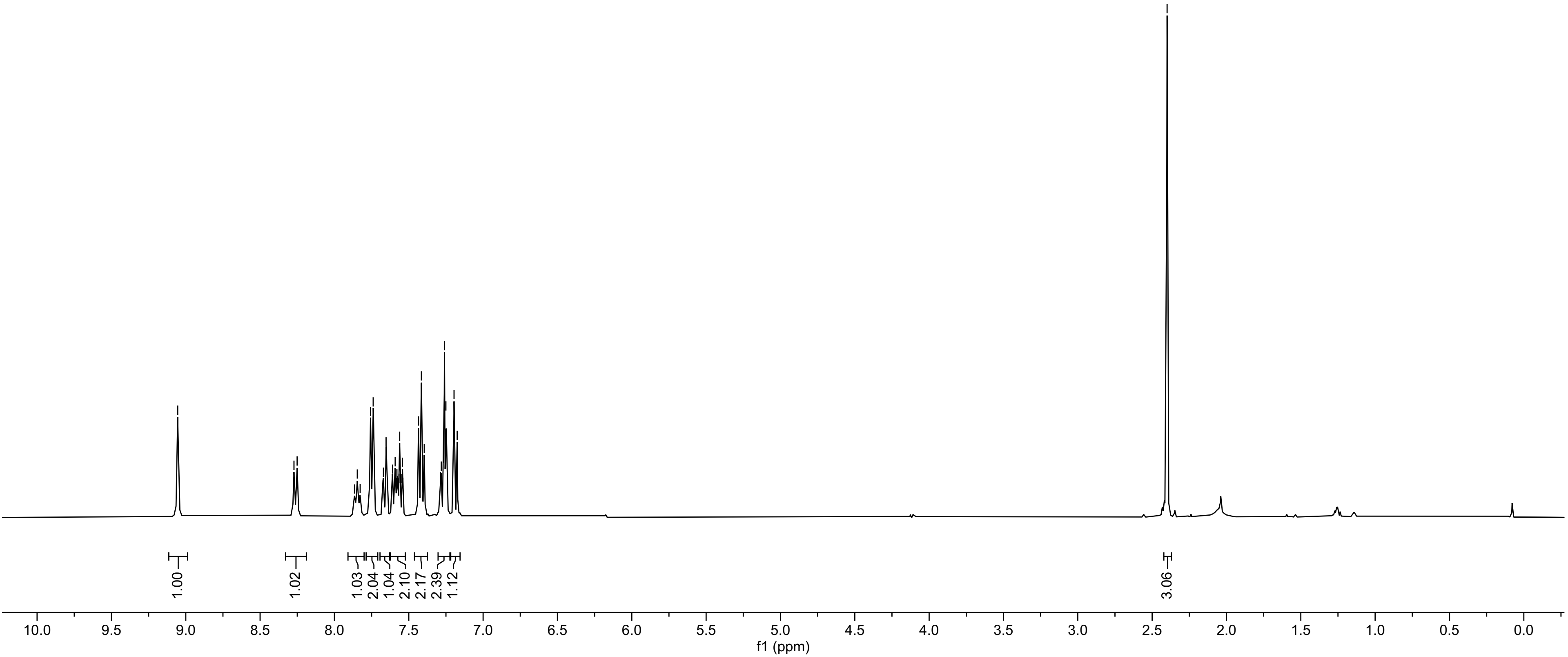

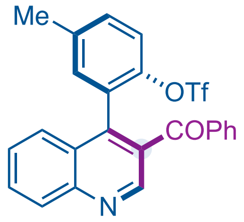

^{13}C NMR of **3w** (101 MHz, $CDCl_3$)

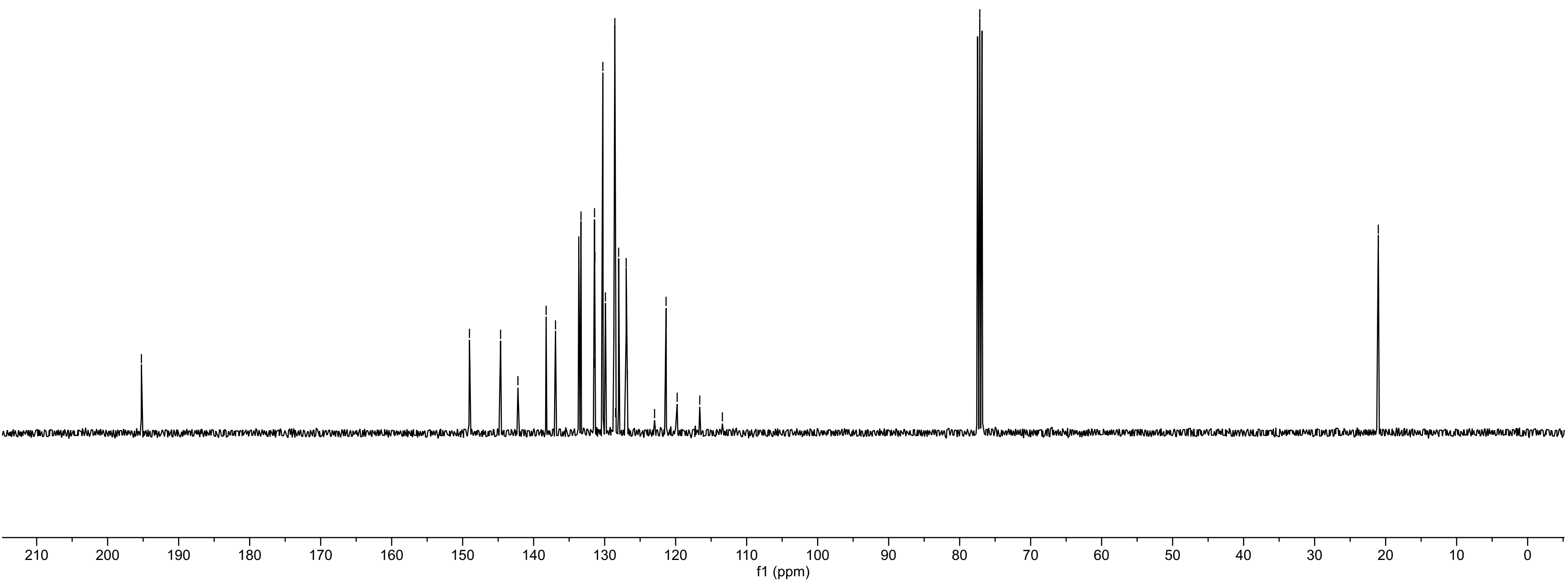

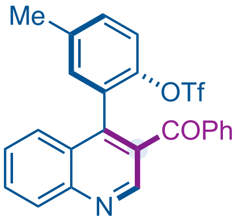

^{19}F NMR of **3w** (376 MHz, $CDCl_3$)

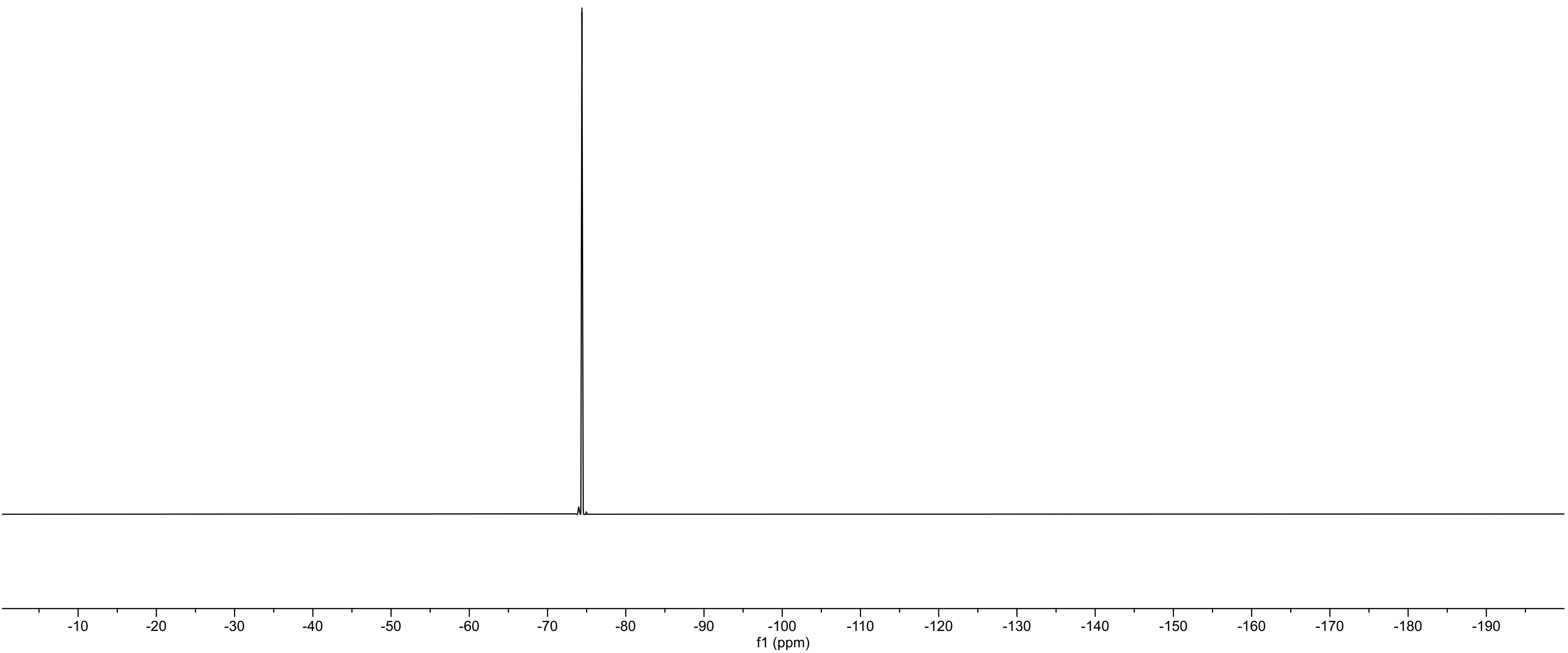

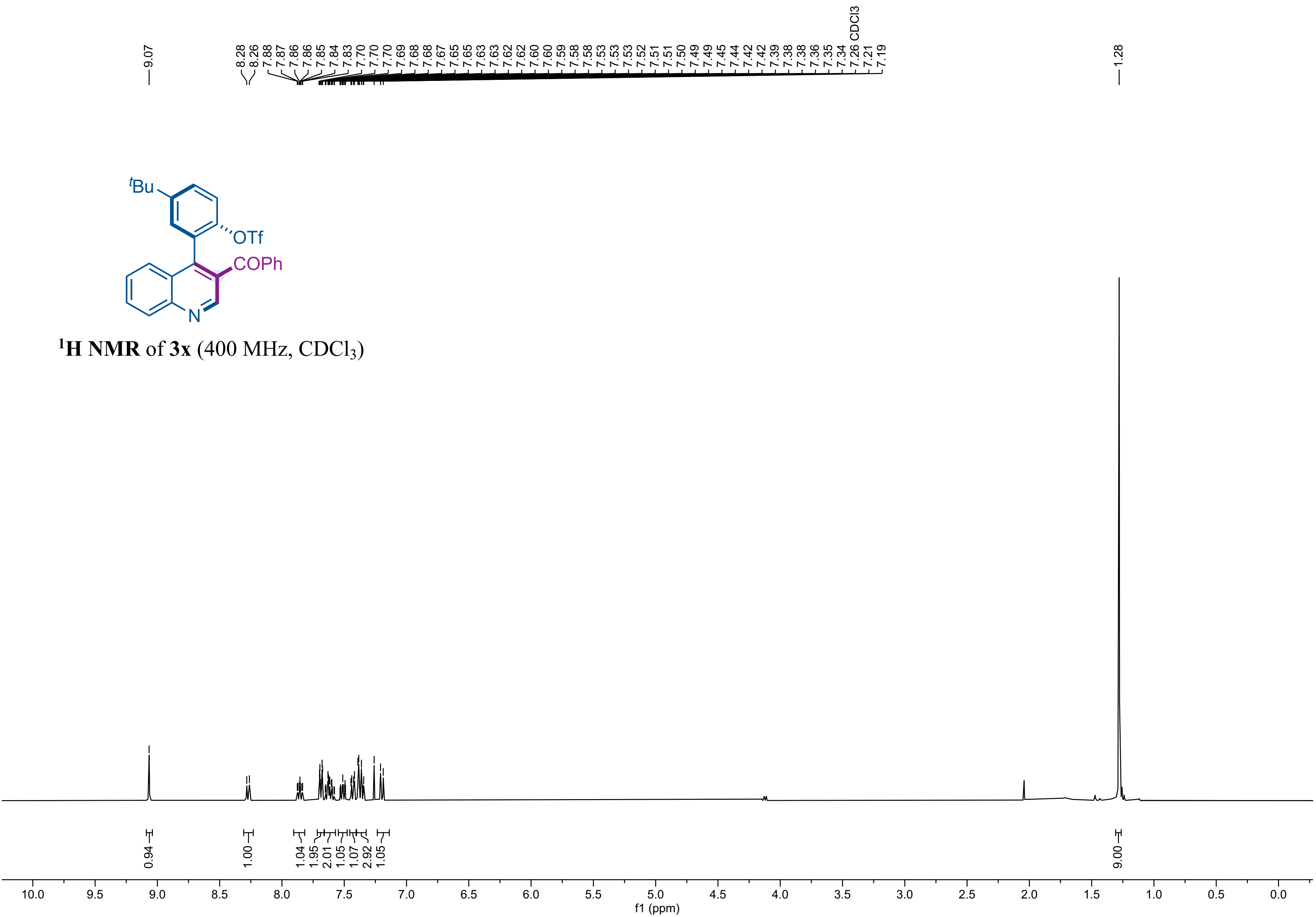

¹H NMR of **3x** (400 MHz, $CDCl_3$)

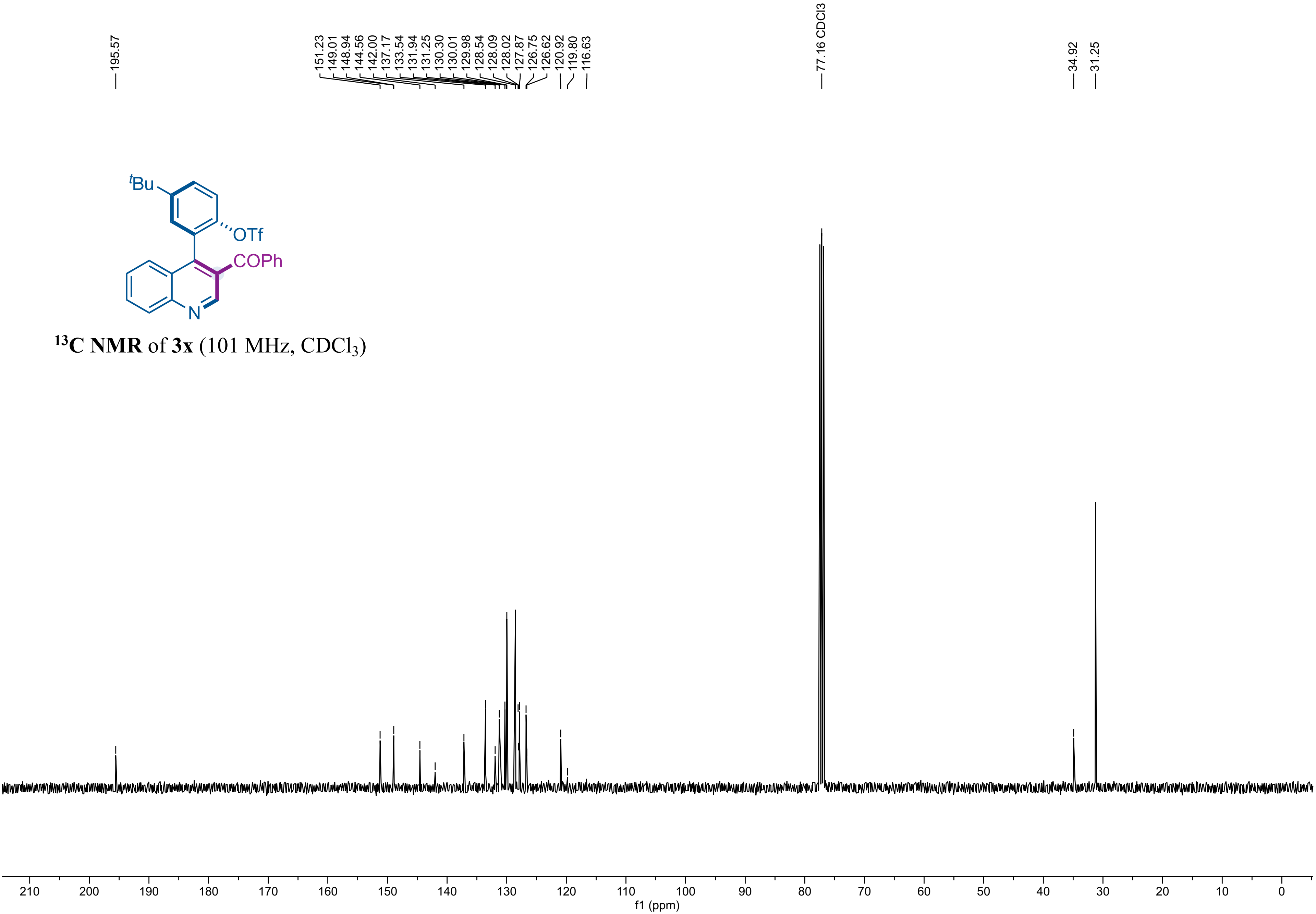

^{13}C NMR of **3x** (101 MHz, $CDCl_3$)

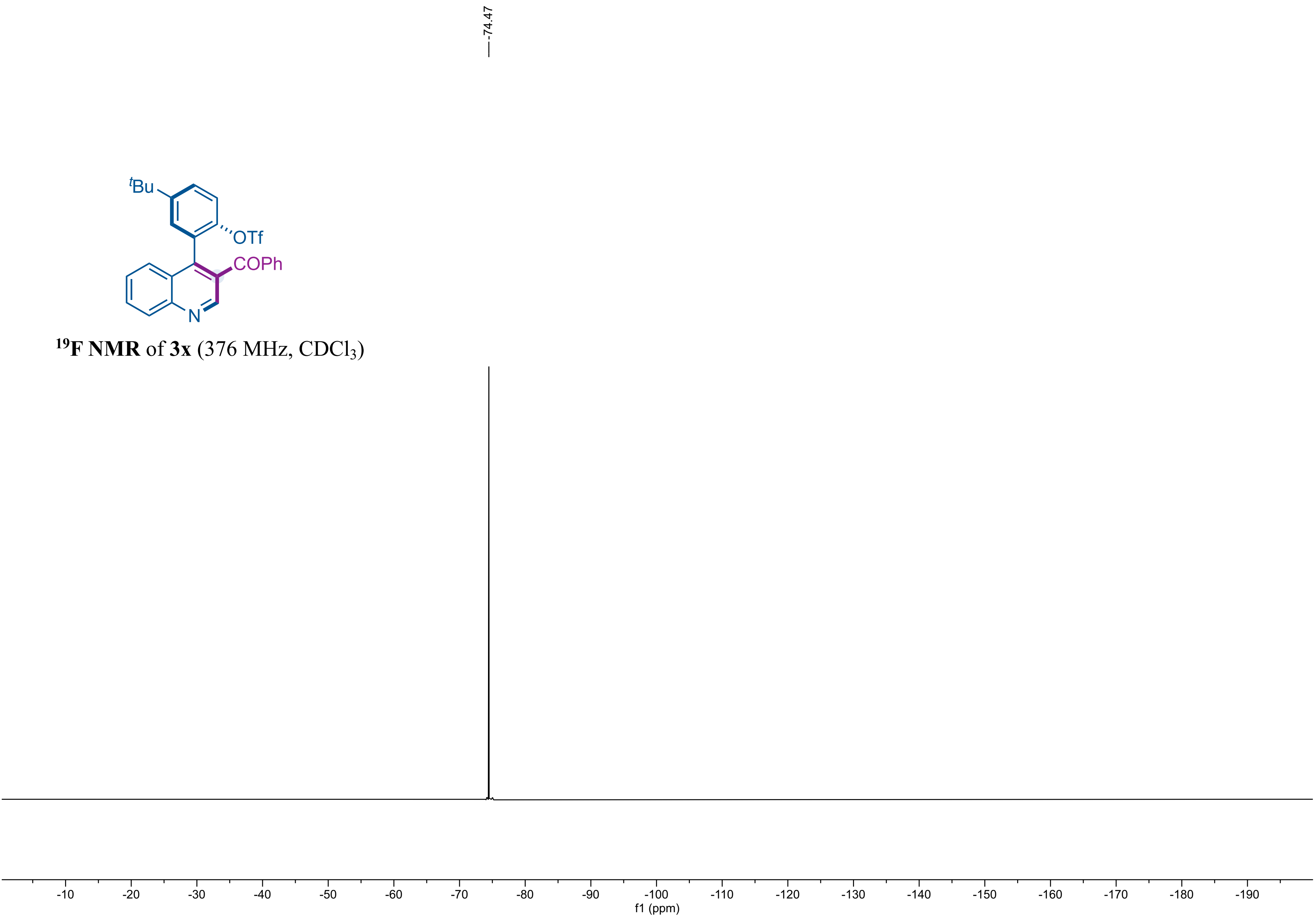

^{19}F NMR of **3x** (376 MHz, $CDCl_3$)

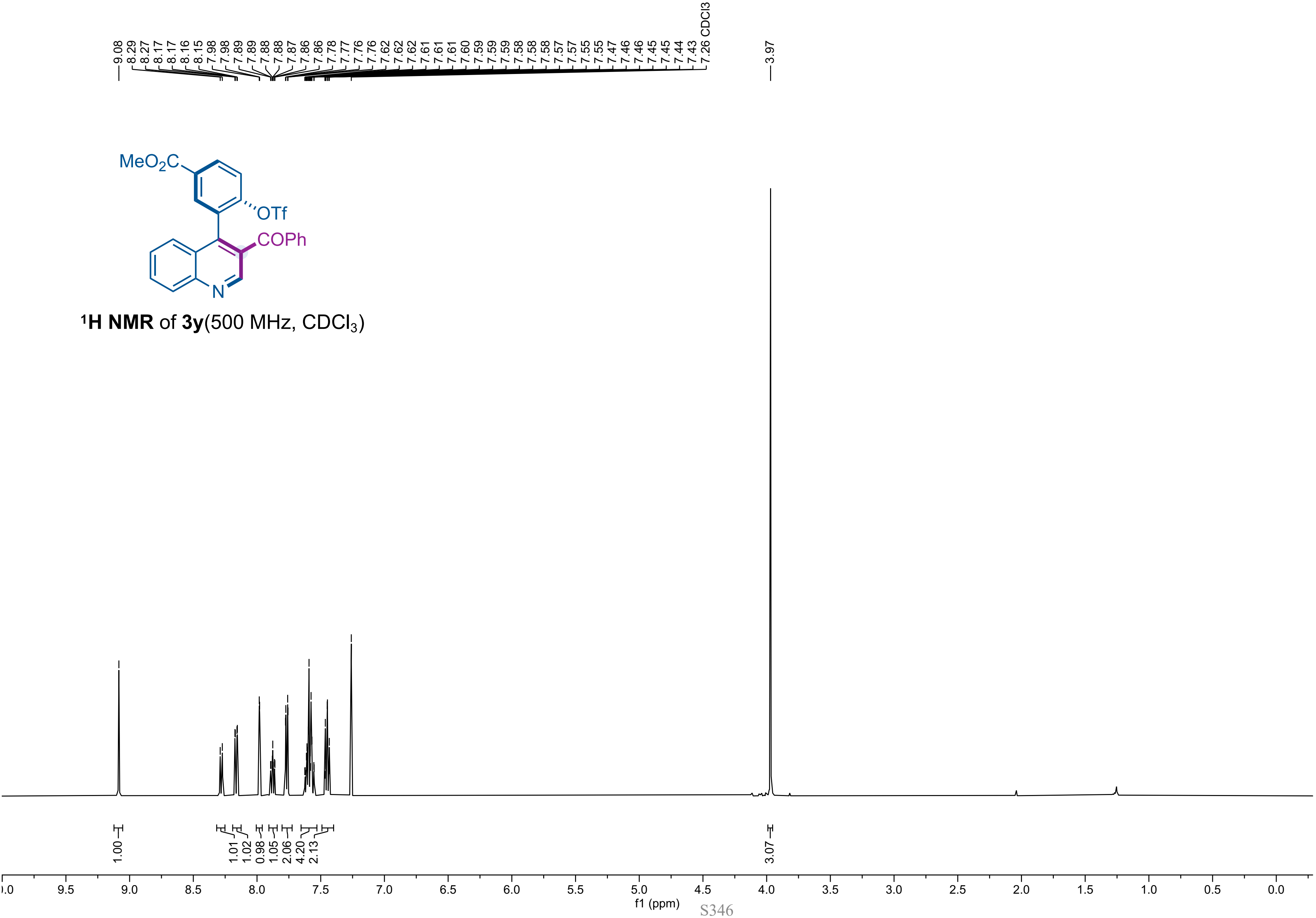
9.08
8.29
8.27
8.17
8.17
8.16
8.15
7.98
7.98
7.89
7.89
7.88
7.88
7.87
7.86
7.86
7.78
7.77
7.76
7.76
7.62
7.62
7.61
7.61
7.61
7.60
7.59
7.59
7.59
7.58
7.58
7.58
7.57
7.57
7.55
7.55
7.47
7.46
7.46
7.45
7.45
7.44
7.43
7.26 CDCl3
3.97
MeO2C
OTf
COPh
N
1H NMR of 3y(500 MHz, CDCl3)
1.00
1.01
1.02
0.98
1.05
2.06
4.20
2.13
3.07
9.5
9.0
8.5
8.0
7.5
7.0
6.5
6.0
5.5
5.0
4.5
4.0
3.5
3.0
2.5
2.0
1.5
1.0
0.5
0.0
f1 (ppm)

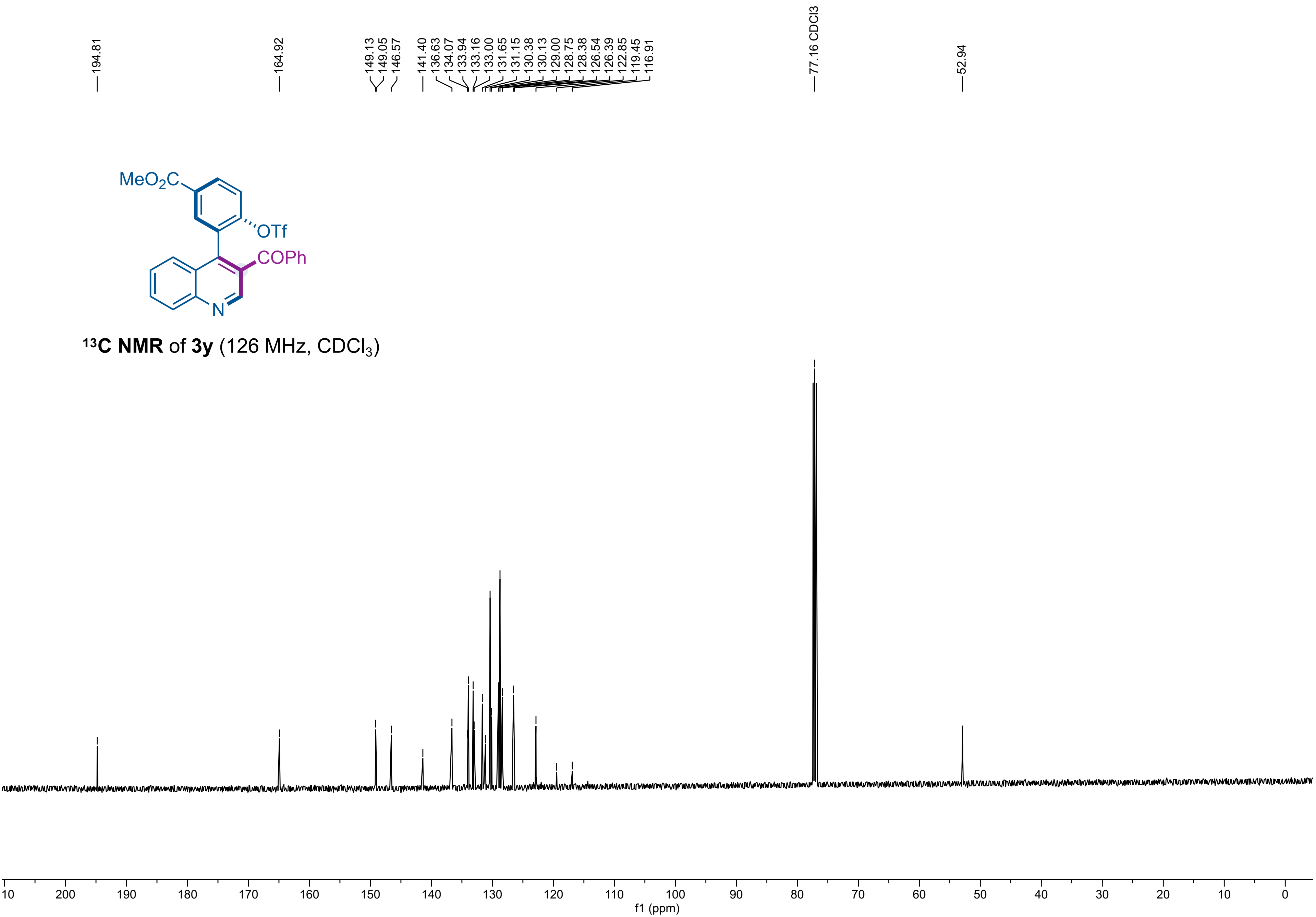

^{13}C NMR of 3y (126 MHz, $CDCl_3$)

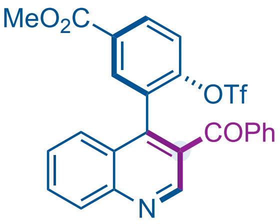

^{19}F NMR of **3y** (471 MHz, $CDCl_3$)

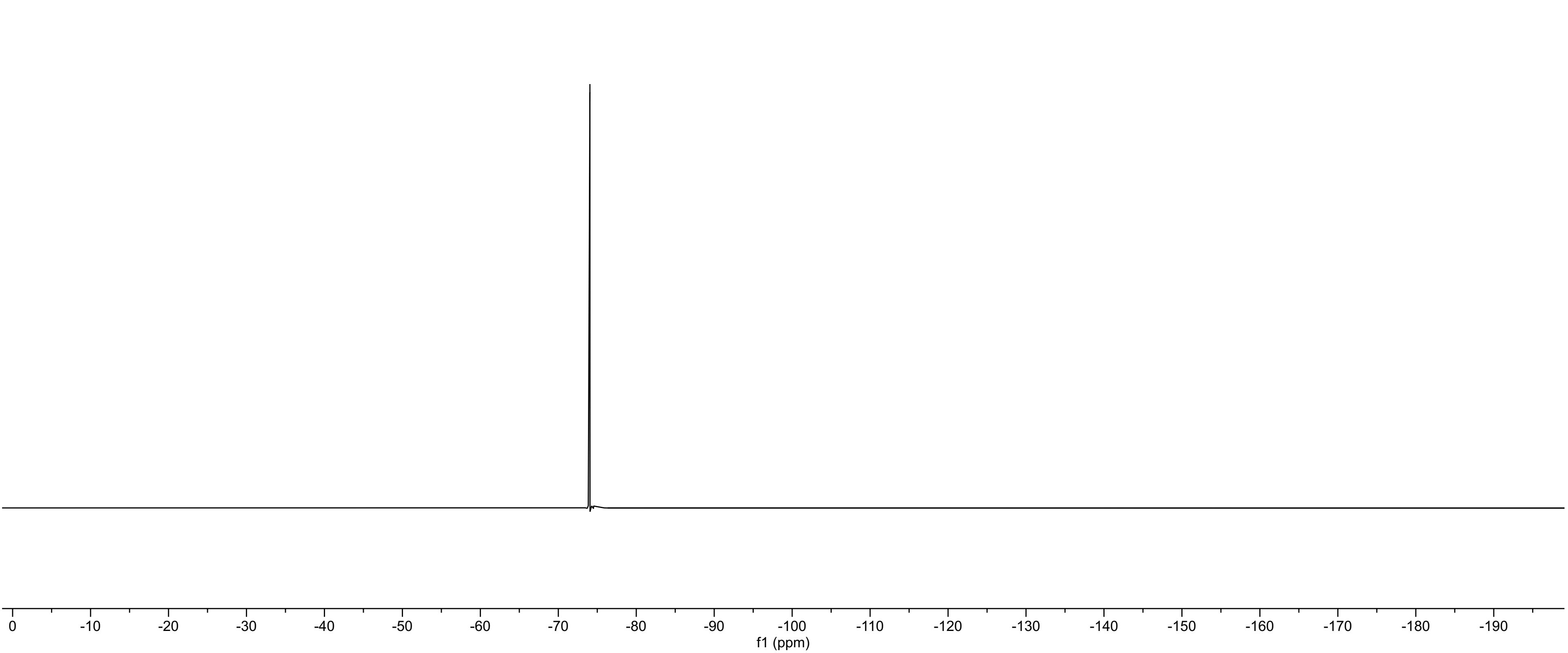

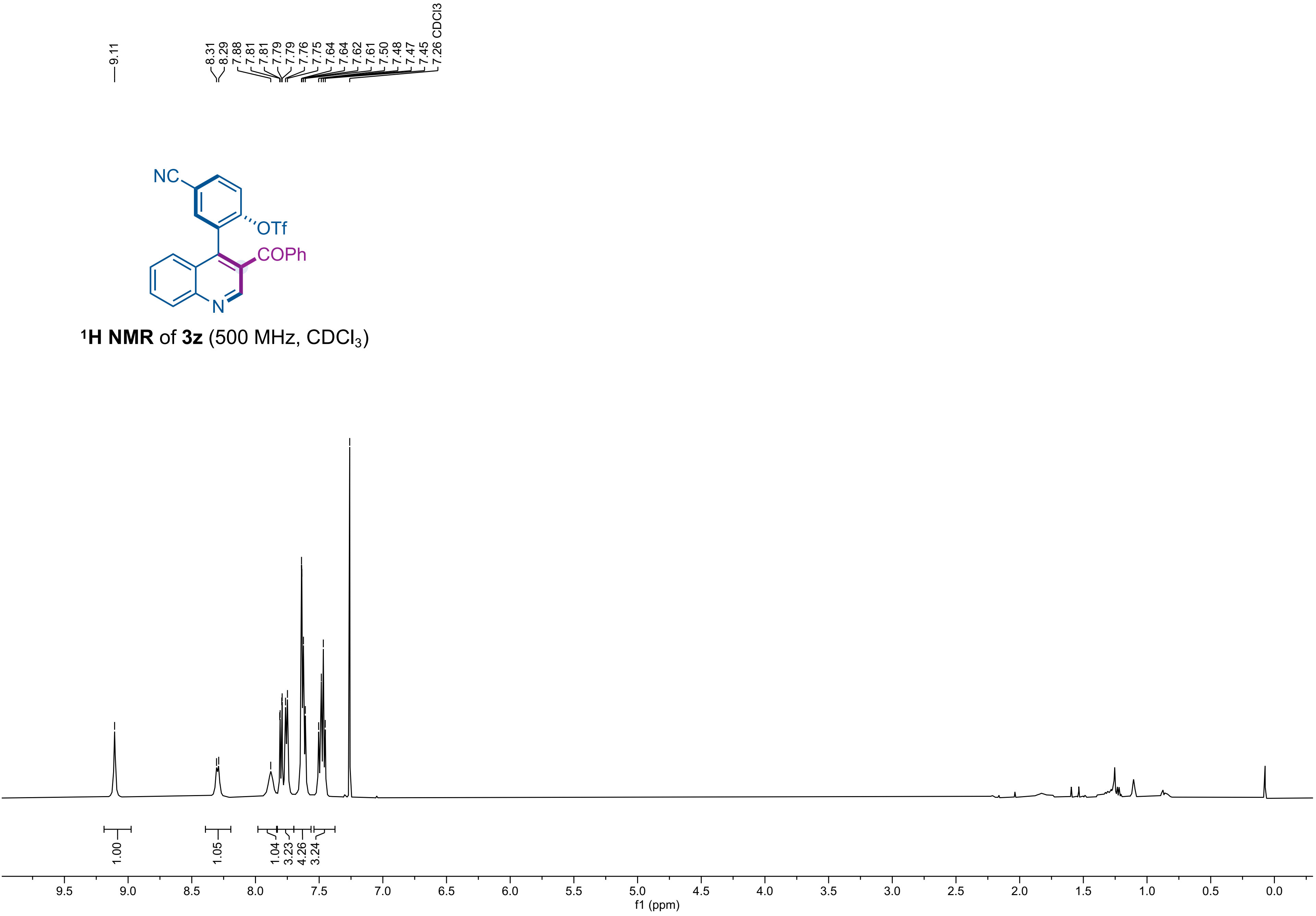

^{1}H NMR of **3z** (500 MHz, $CDCl_3$)

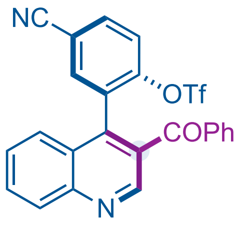

^{13}C NMR of **3z** (126 MHz, $CDCl_3$)

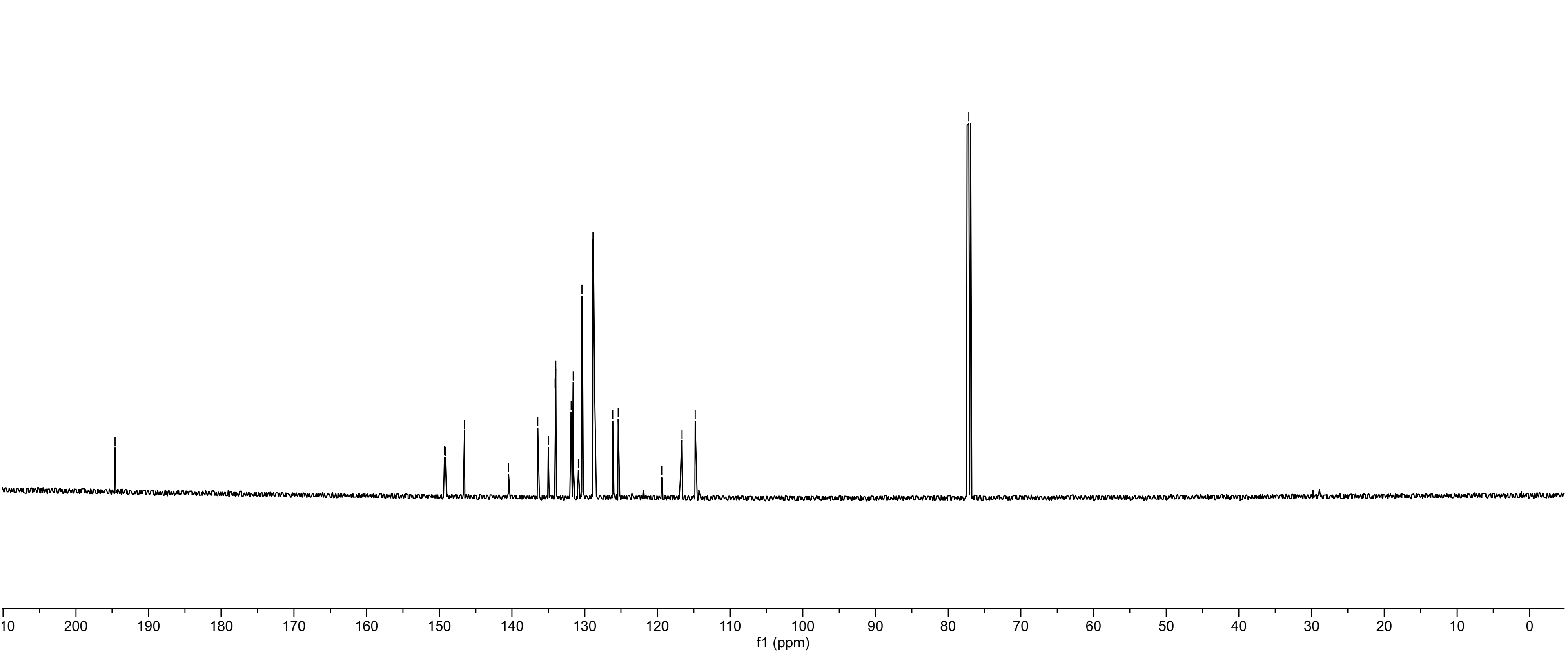

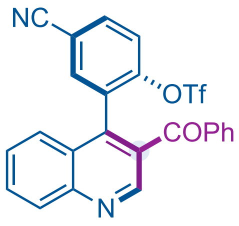

^{19}F NMR of **3z** (471 MHz, $CDCl_3$)

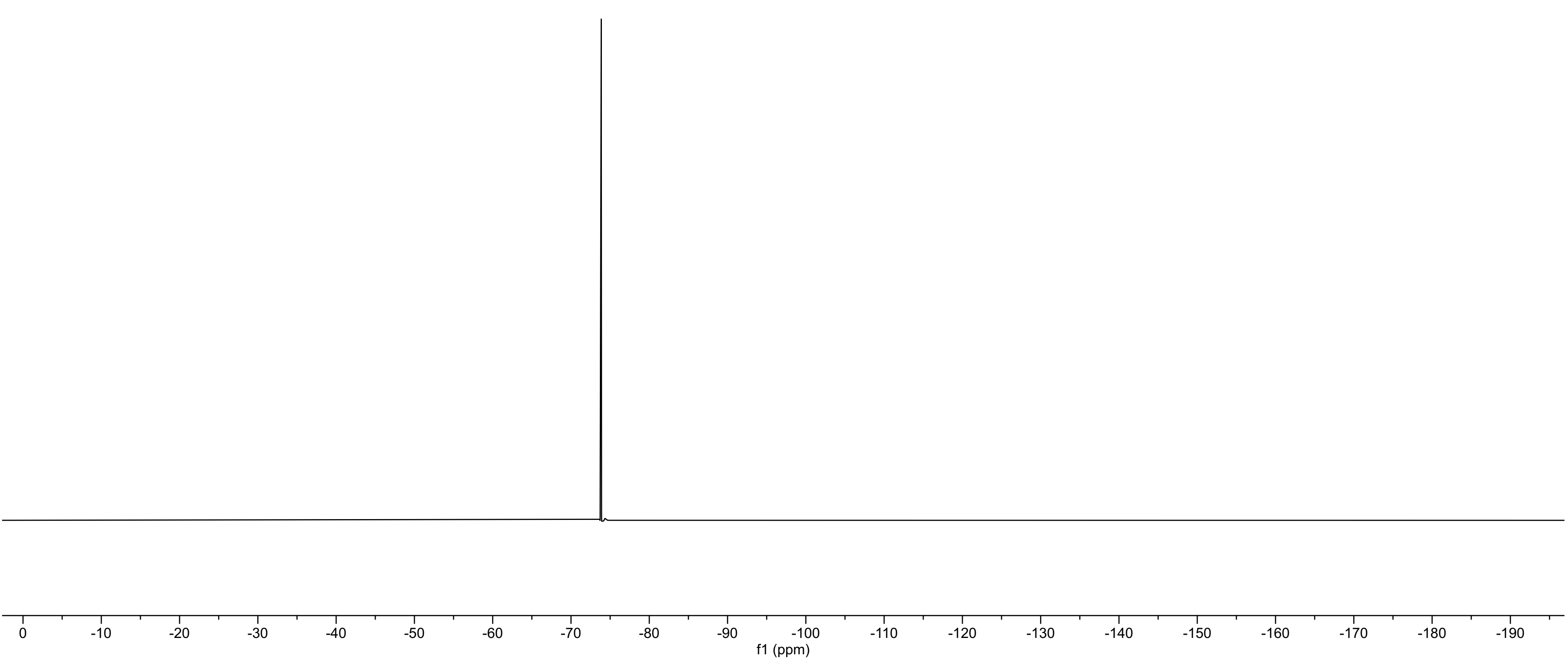

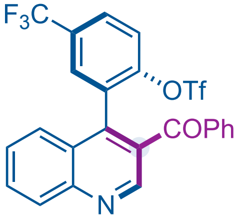

¹H NMR of **3aa** (300 MHz, $CDCl_3$)

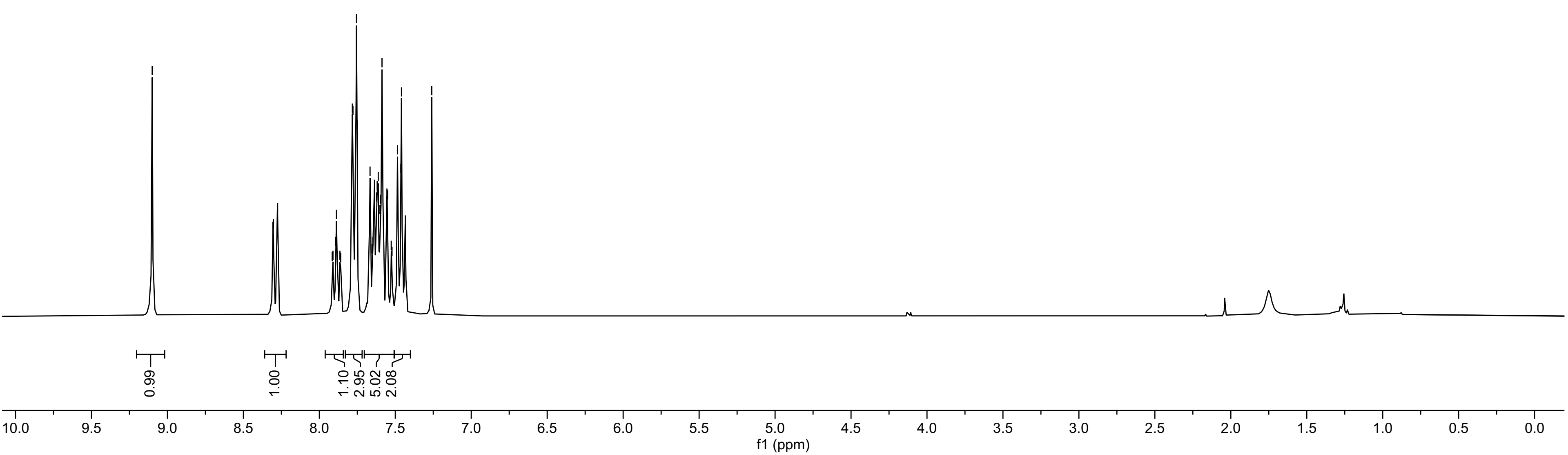

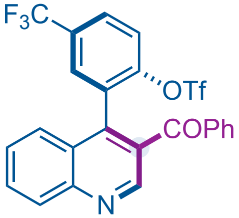

¹H NMR of **3aa** (300 MHz, $CDCl_3$)

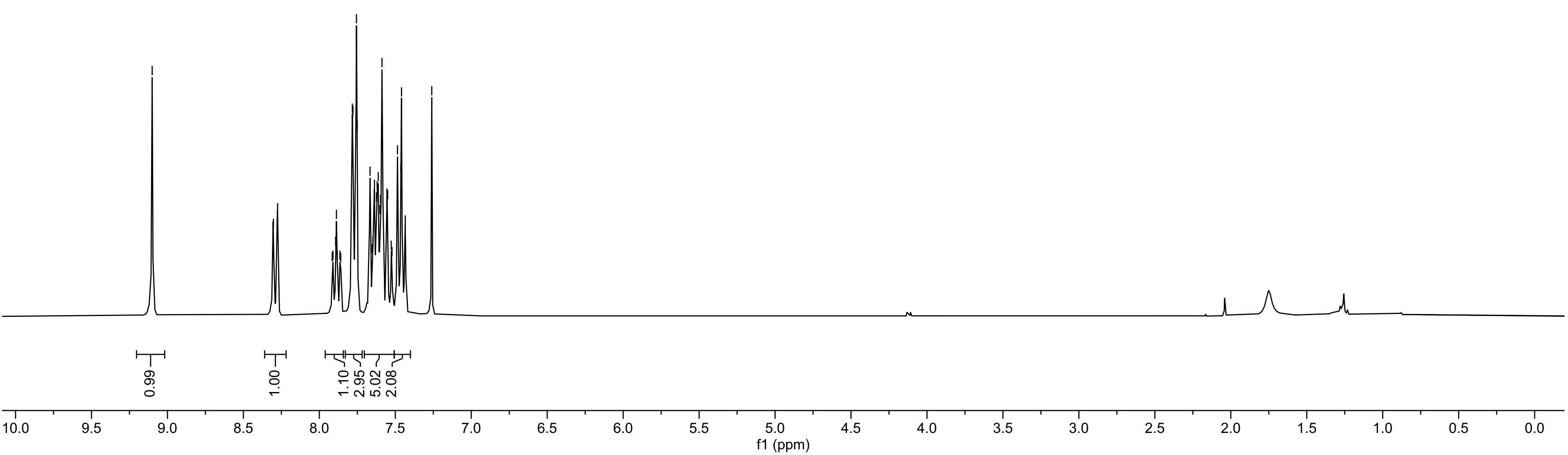

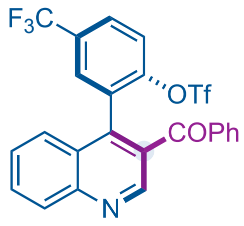

^{19}F NMR of **3aa** (282 MHz, $CDCl_3$)

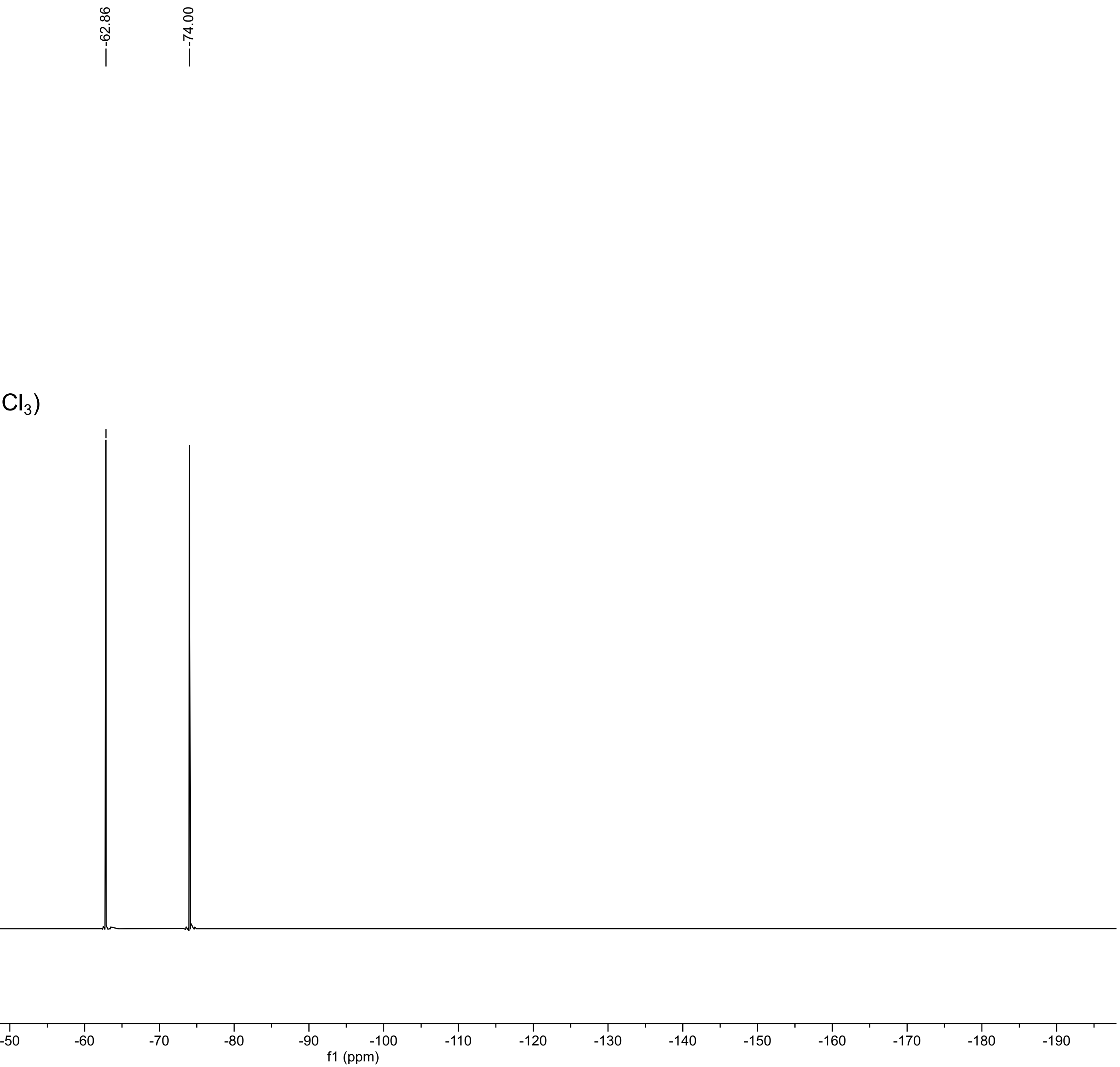

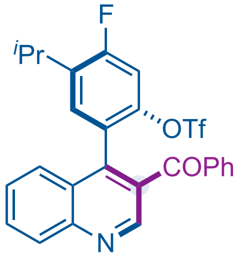

^{1}H NMR of **3ab** (400 MHz, $CDCl_3$)

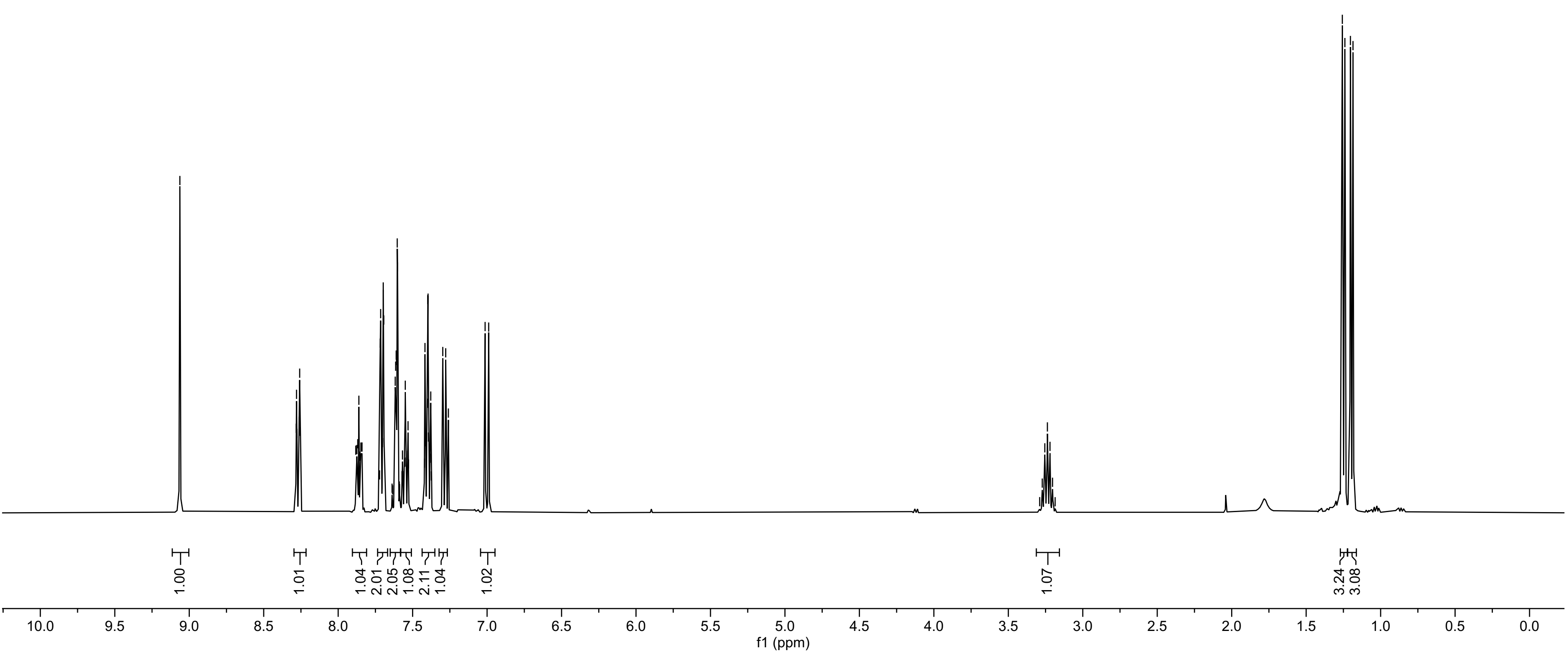

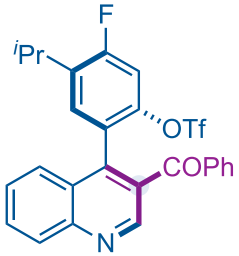

^{13}C NMR of **3ab** (101 MHz, $CDCl_3$)

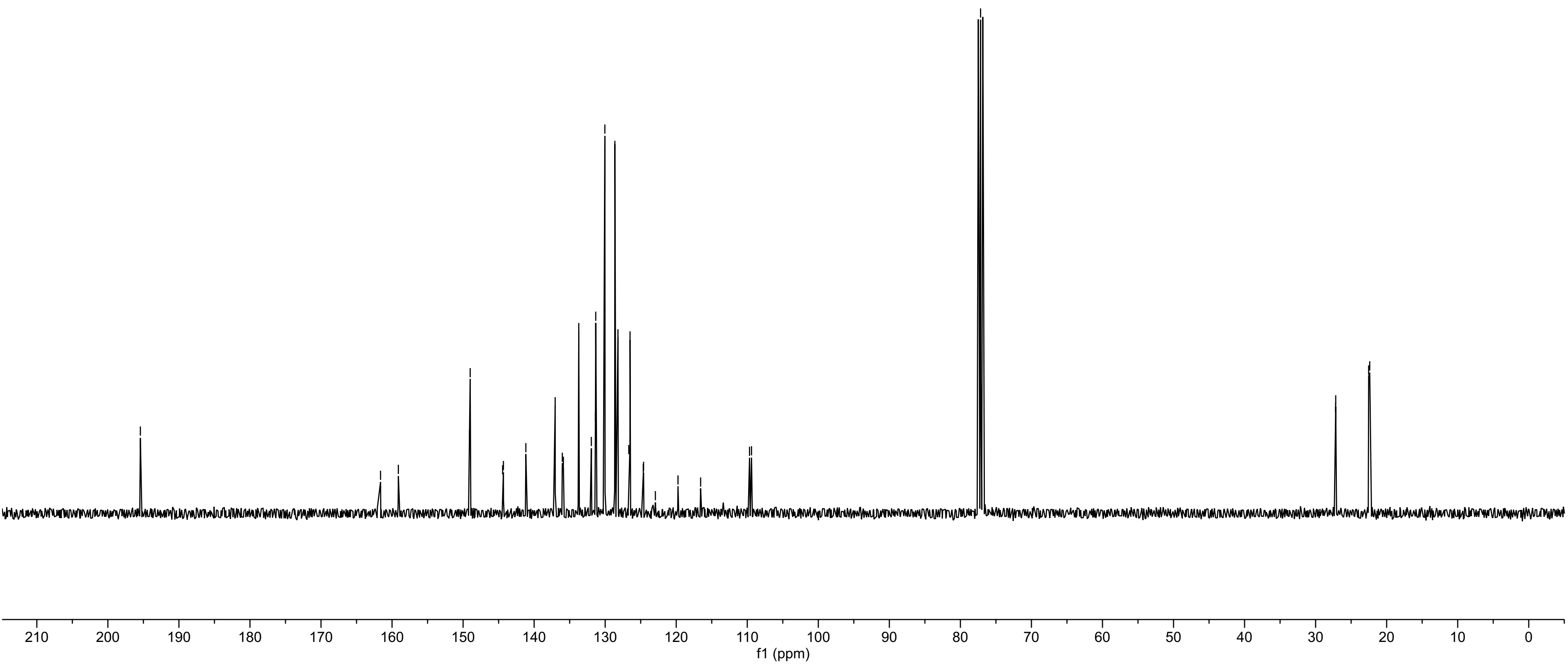

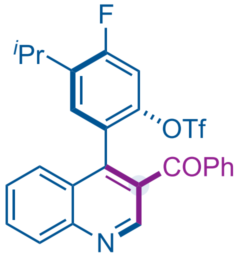

^{19}F NMR of **3ab** (376 MHz, $CDCl_3$)

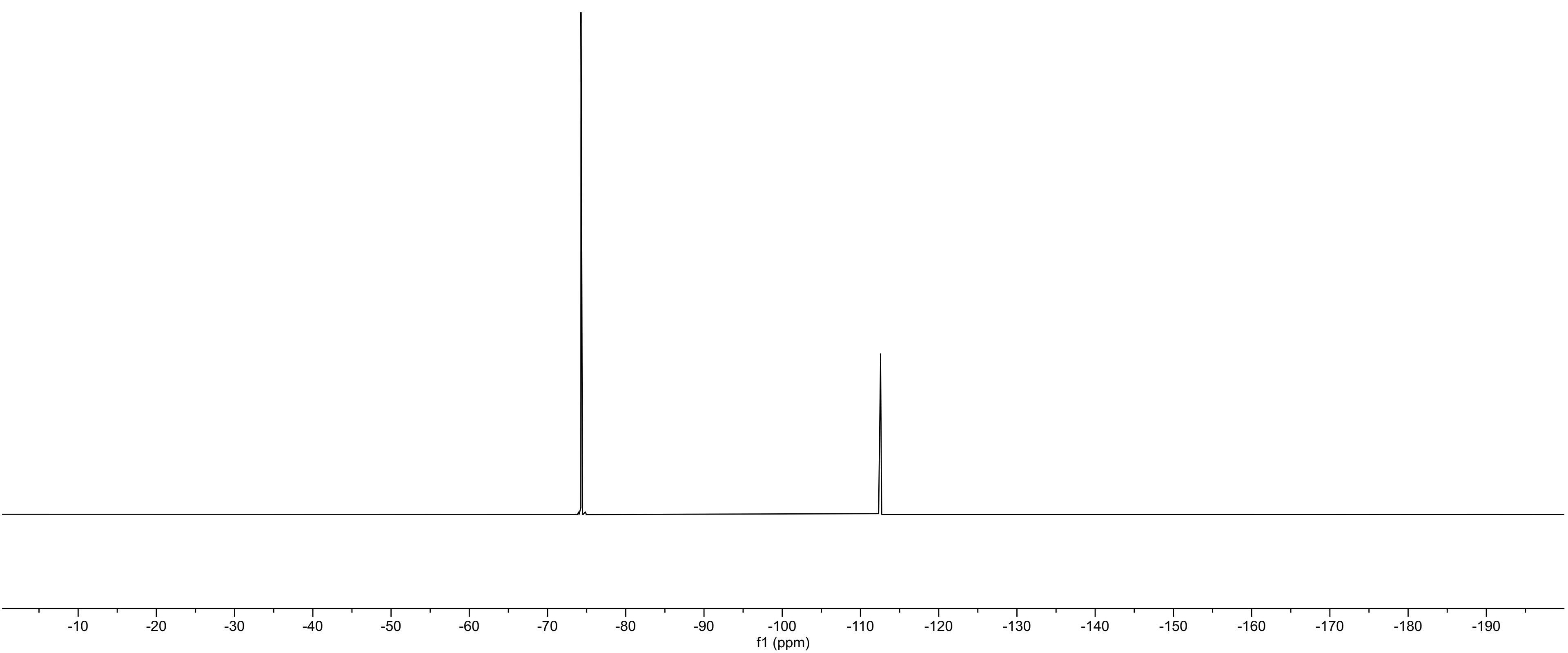

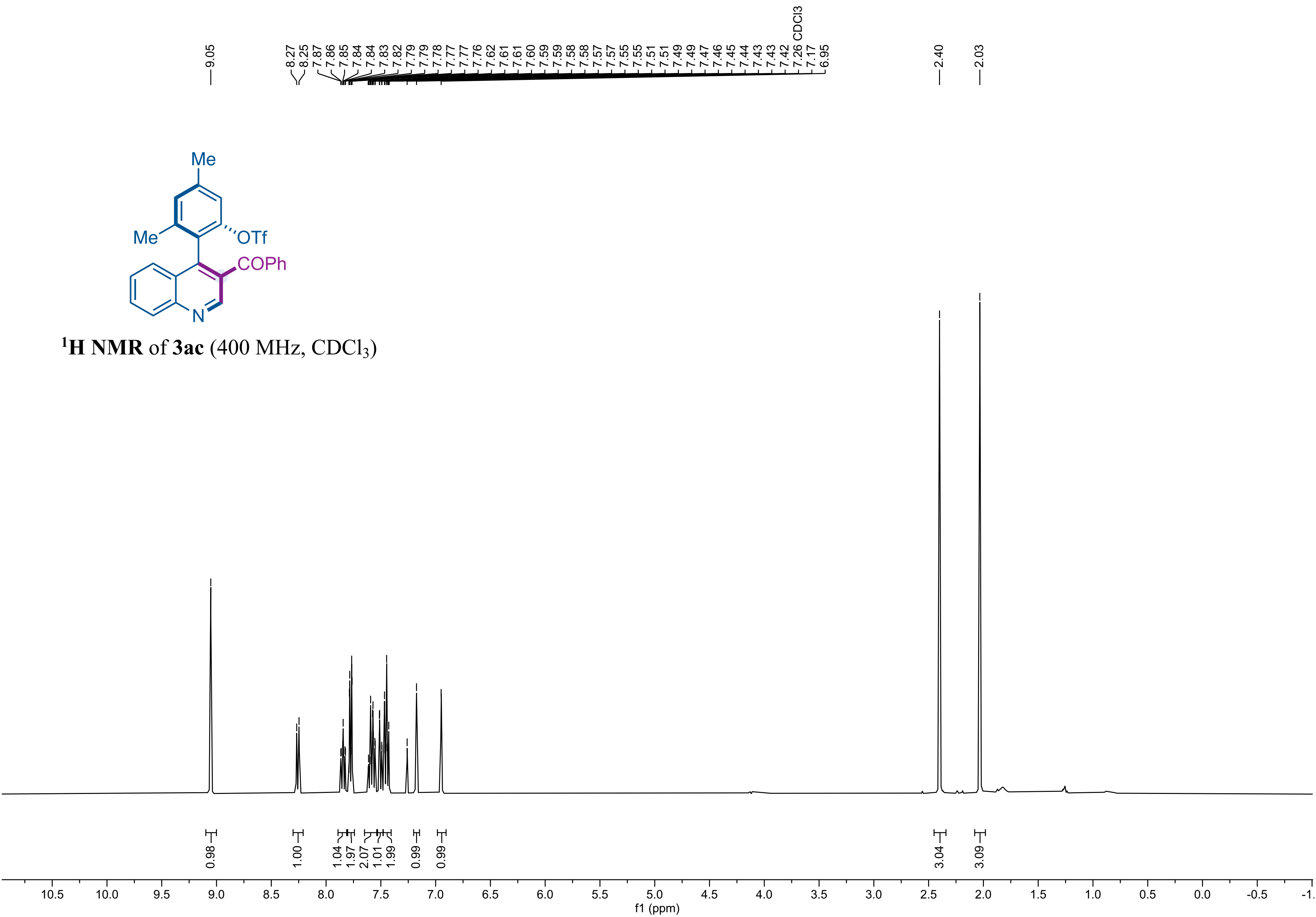

^{1}H NMR of **3ac** (400 MHz, $CDCl_3$)

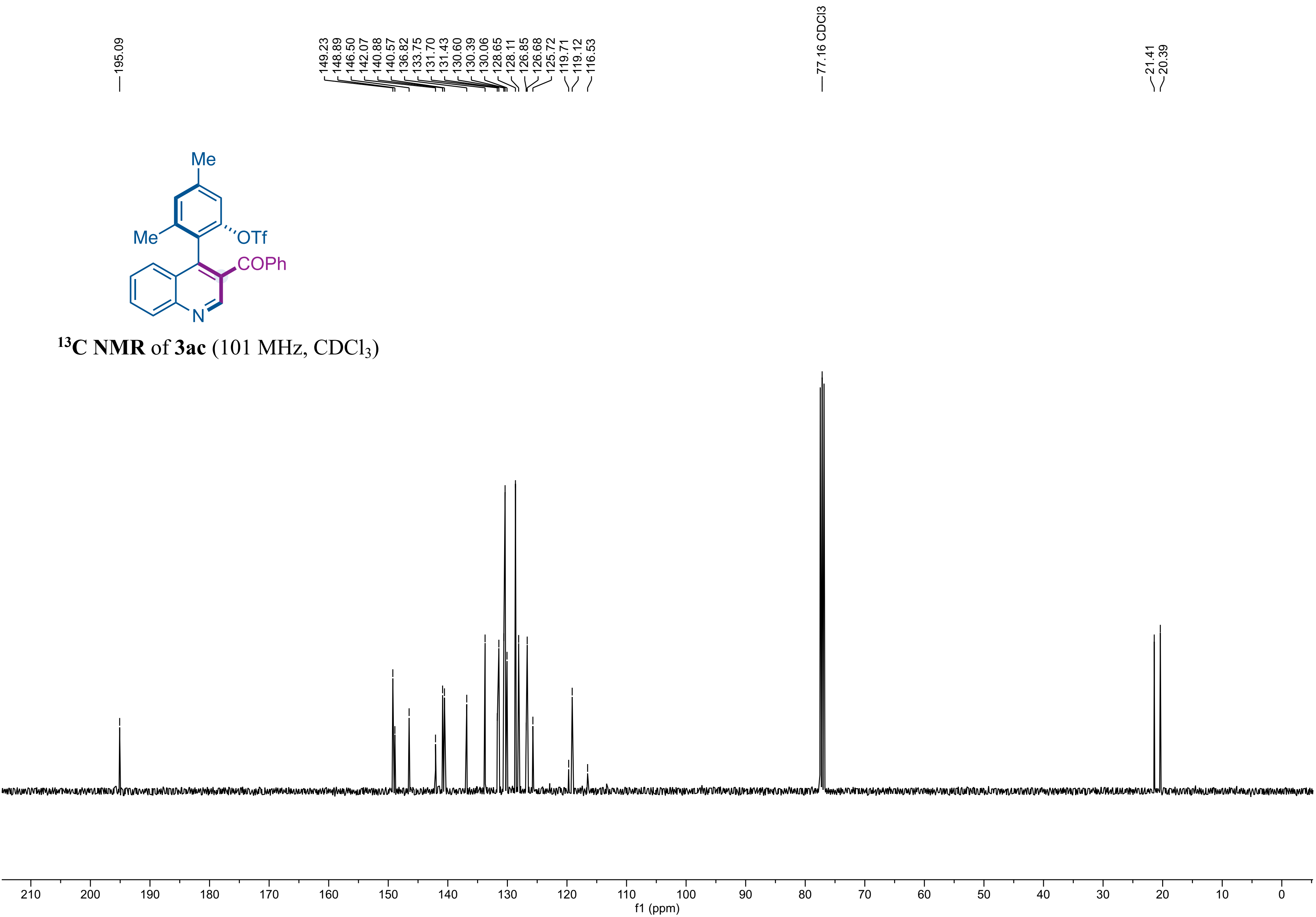

^{13}C NMR of **3ac** (101 MHz, $CDCl_3$)

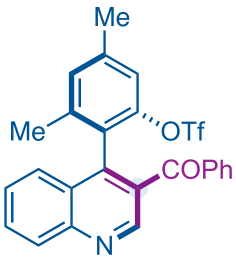

^{19}F NMR of **3ac** (376 MHz, $CDCl_3$)

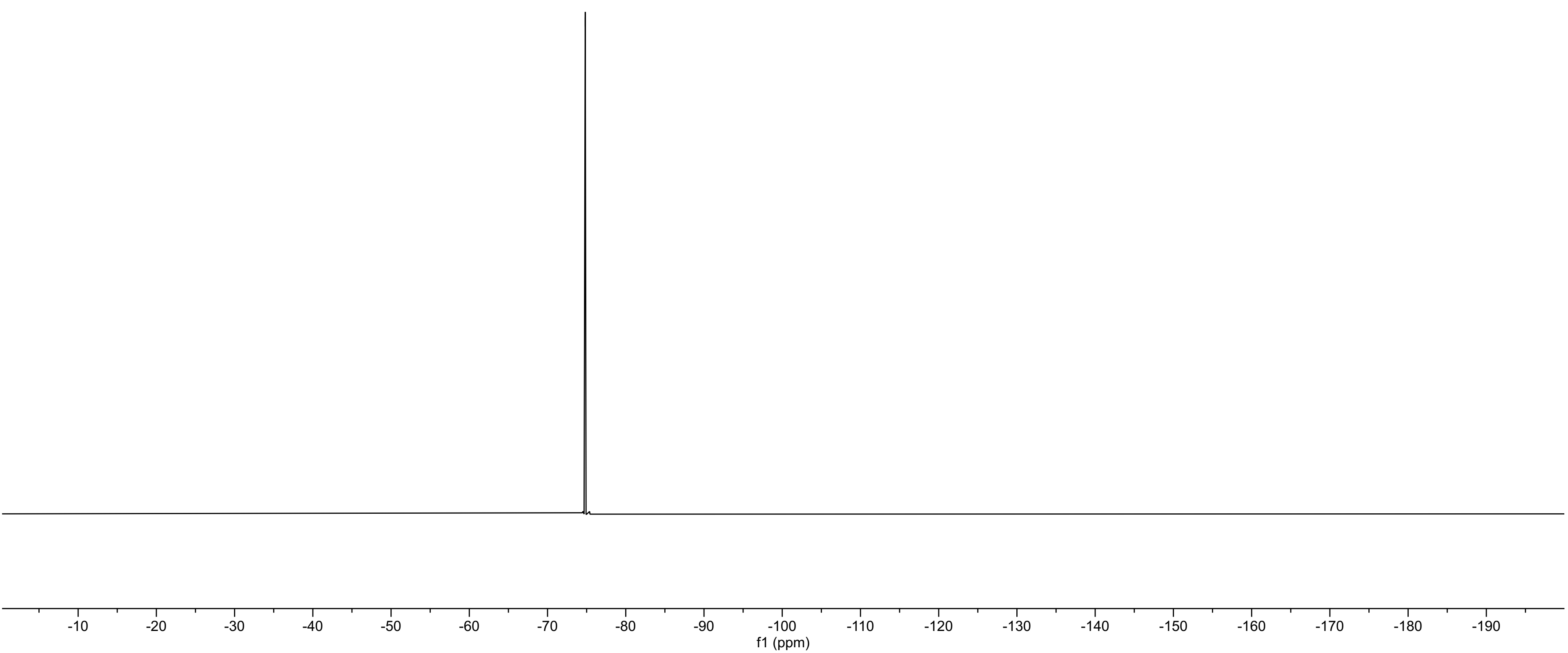

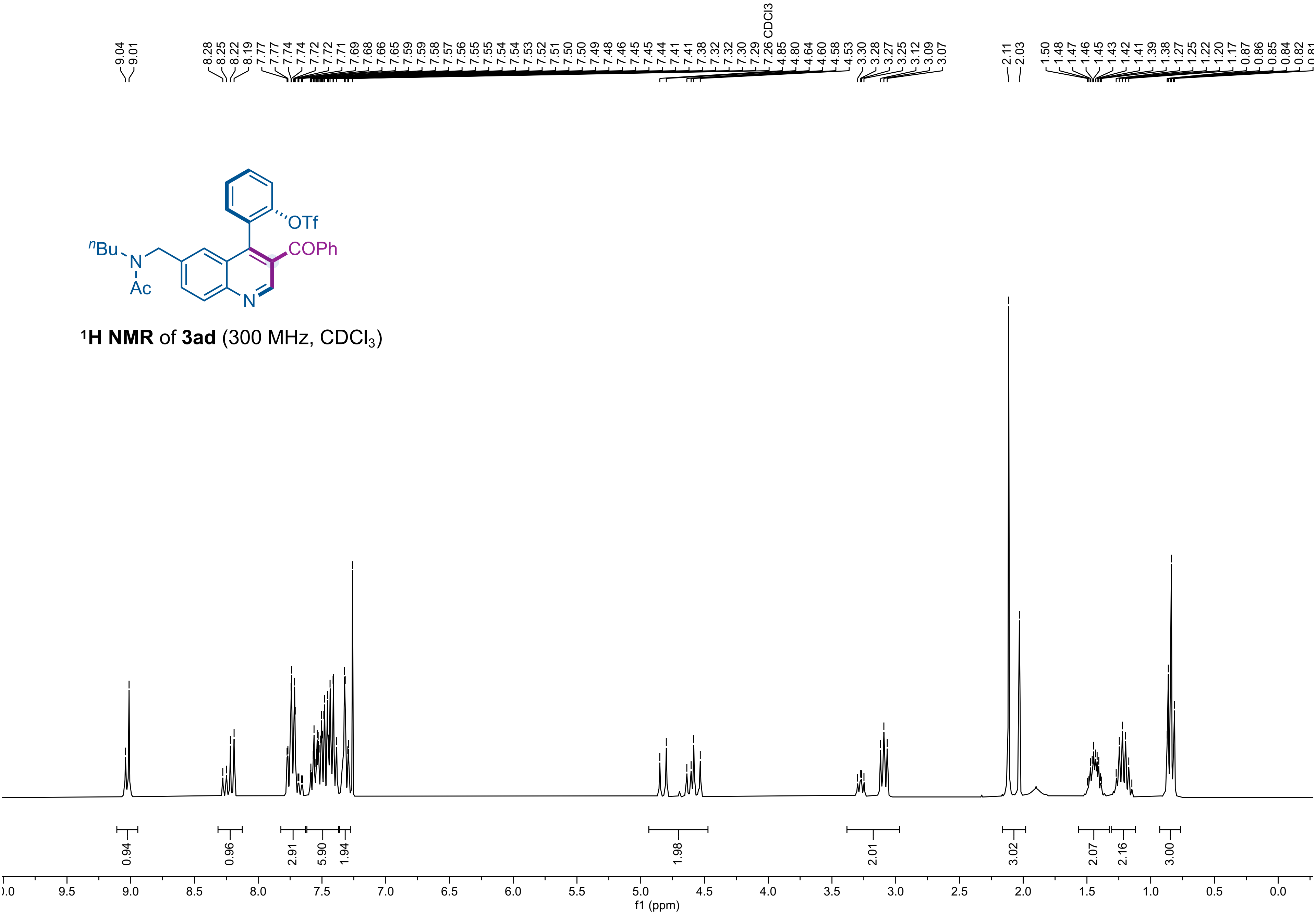

^{1}H NMR of **3ad** (300 MHz, $CDCl_3$)

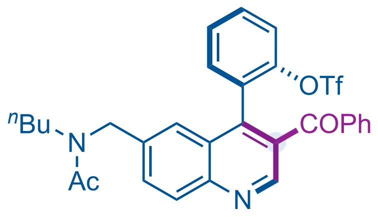

^{13}C NMR of **3ad** (75 MHz, $CDCl_3$)

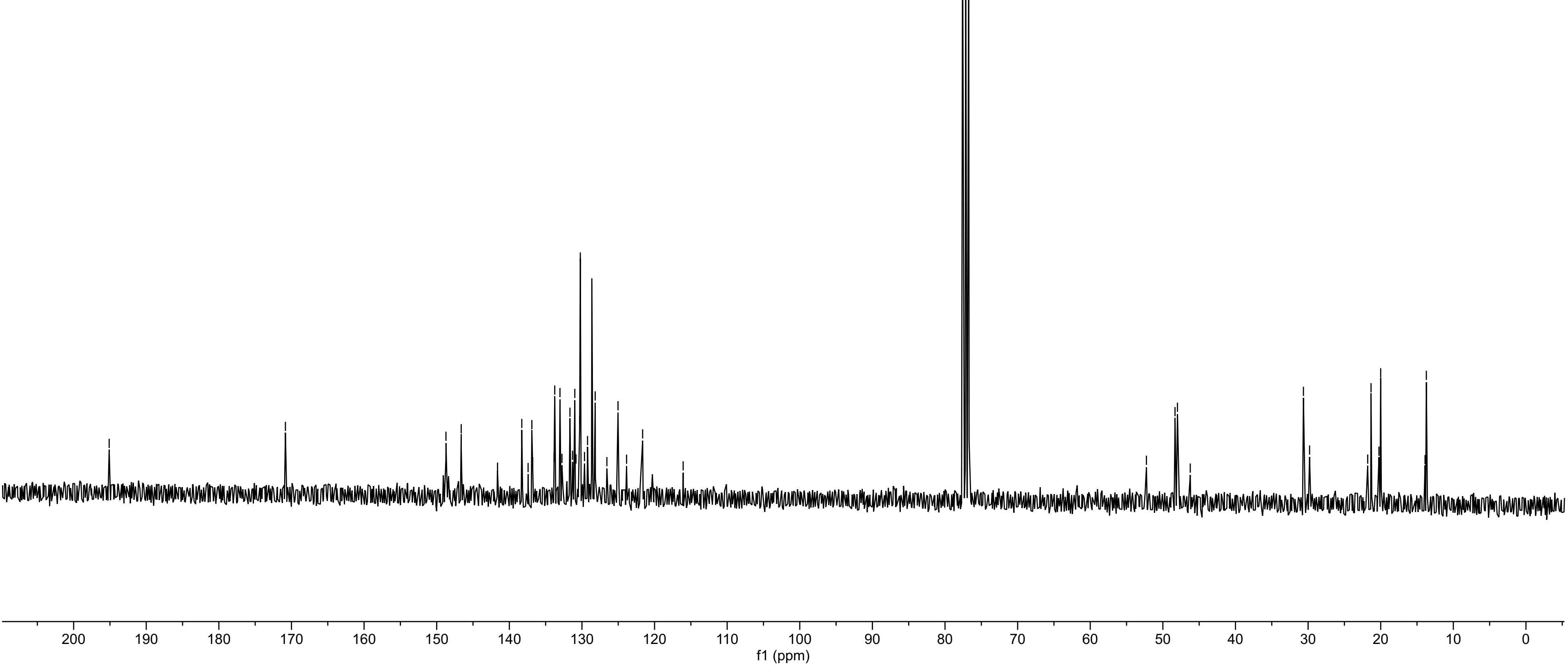

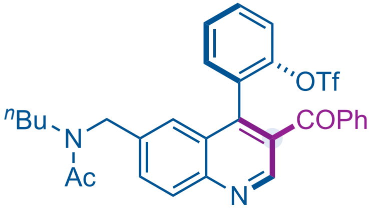

^{19}F NMR of **3ad** (282 MHz, $CDCl_3$)

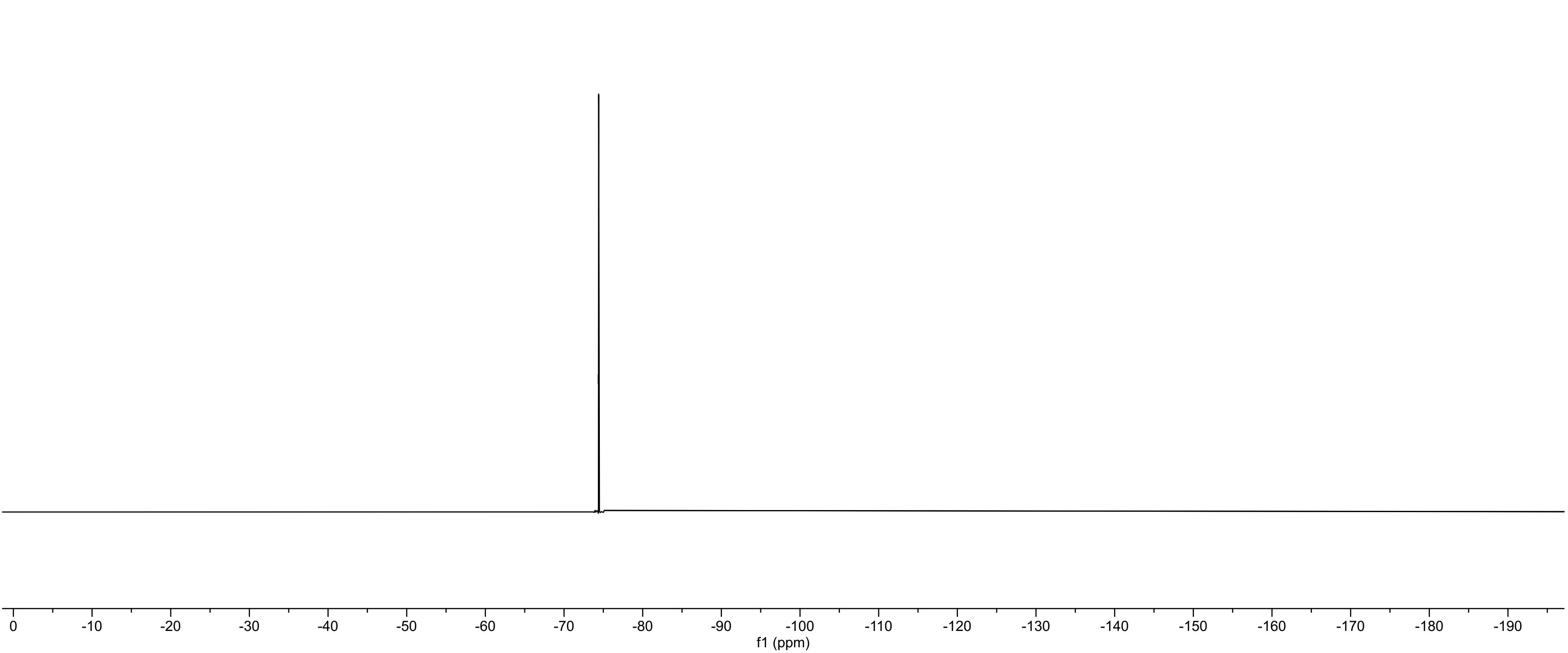

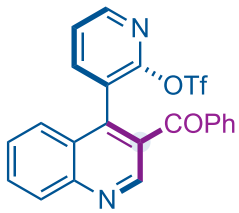

^{1}H NMR of **3ae** (400 MHz, $CDCl_3$)

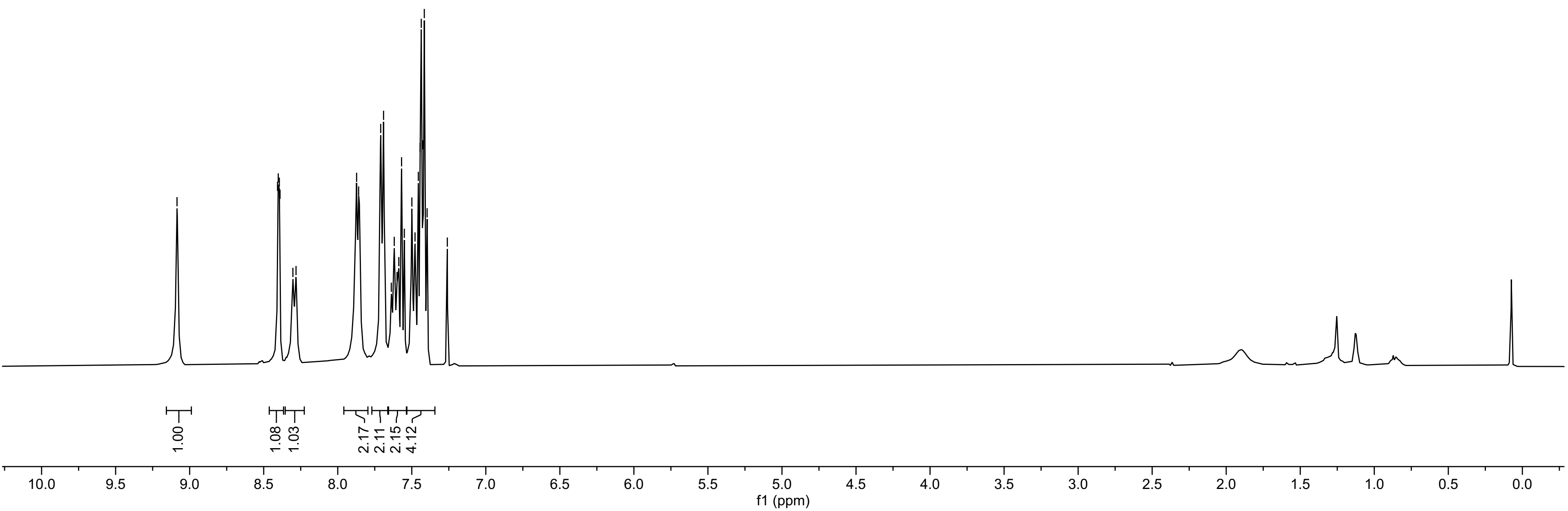

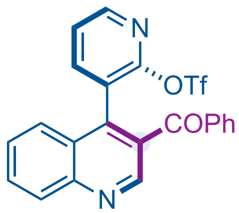

^{13}C NMR of **3ae** (101 MHz, $CDCl_3$)

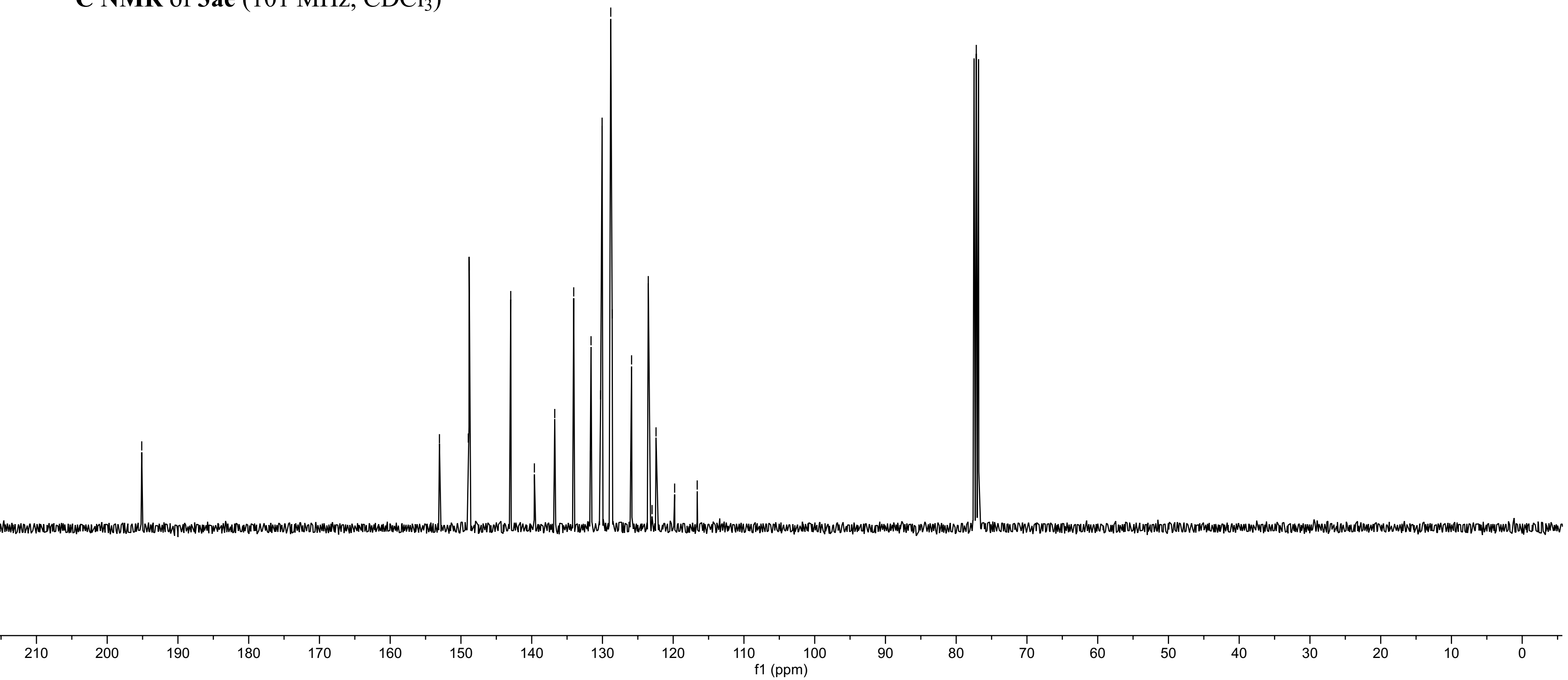

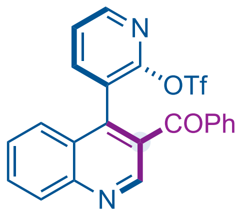

^{19}F NMR of **3ae** (376 MHz, $CDCl_3$)

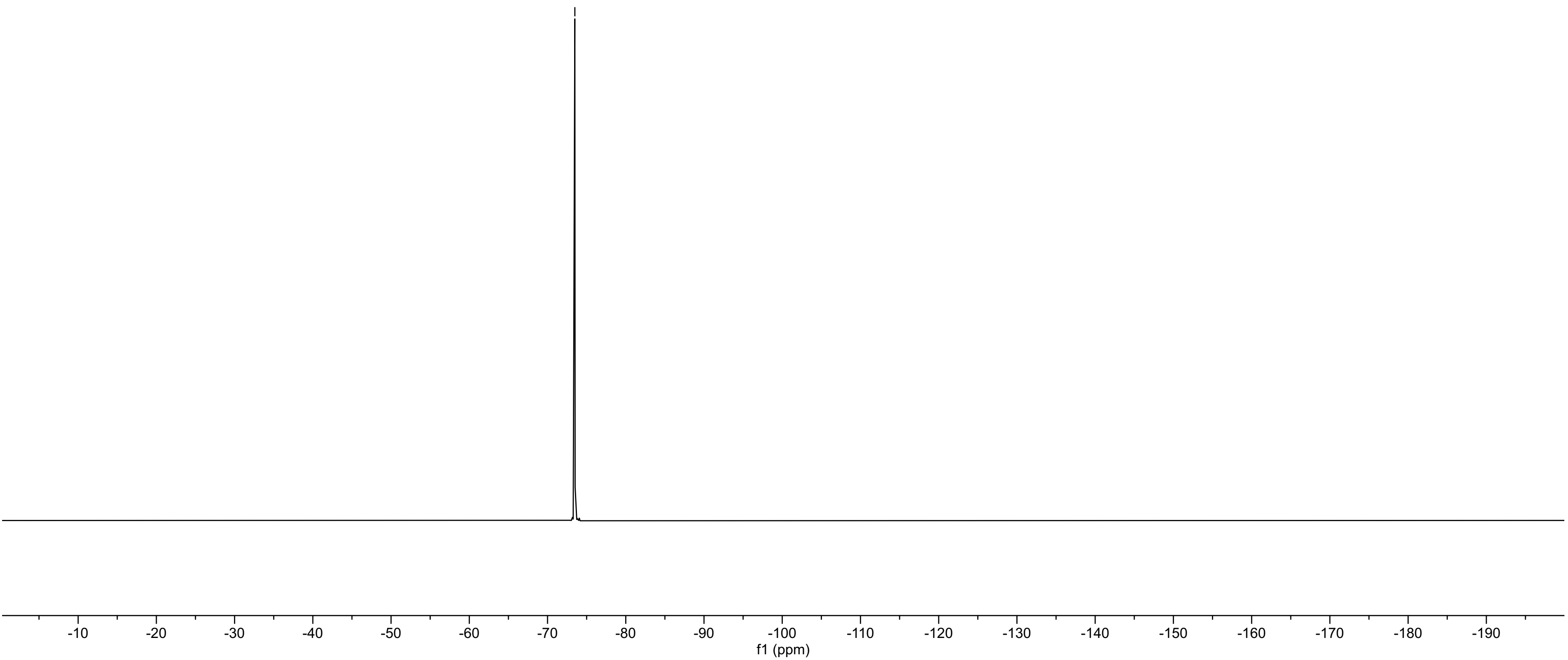

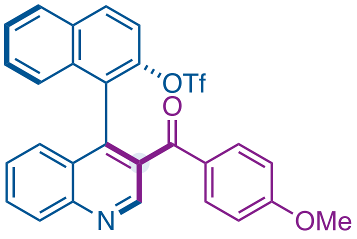

^{1}H NMR of **3af** (400 MHz, $CDCl_3$)

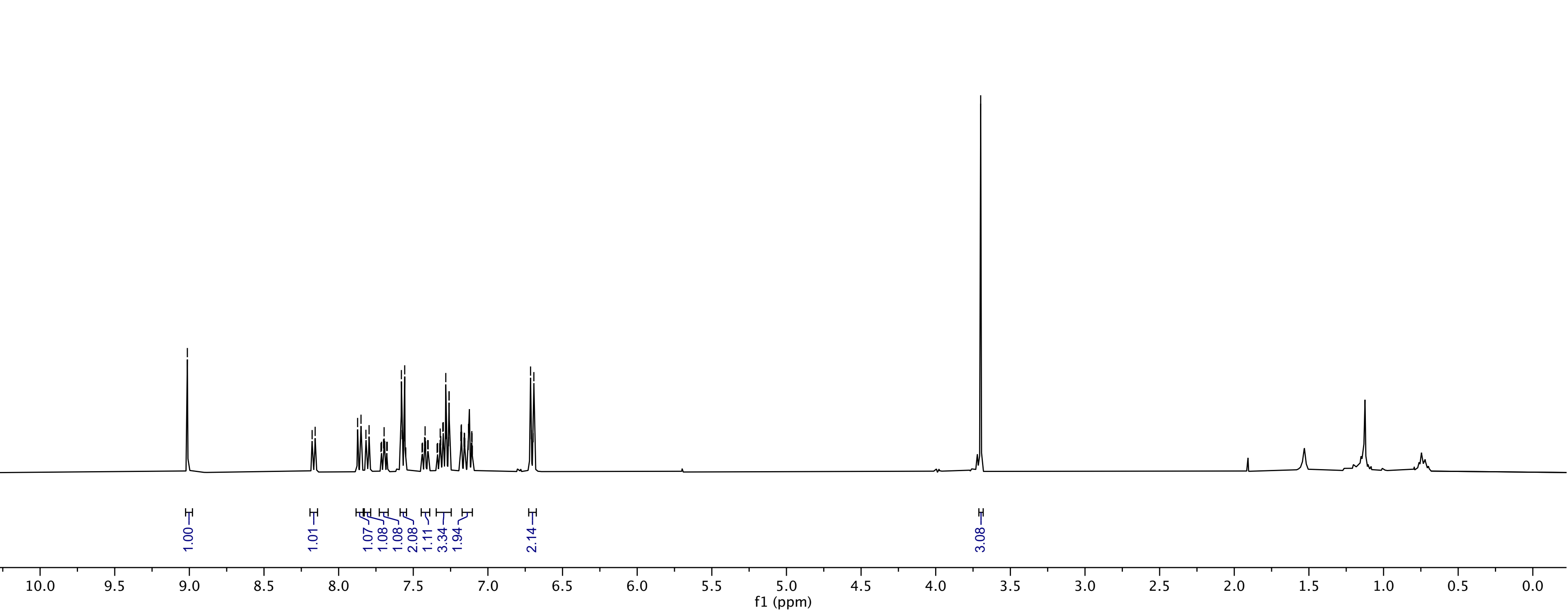

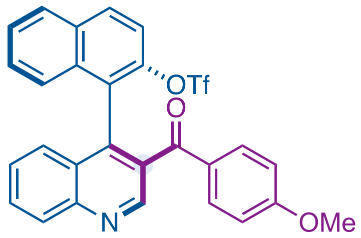

^{13}C NMR of **3af** (101 MHz, $CDCl_3$)

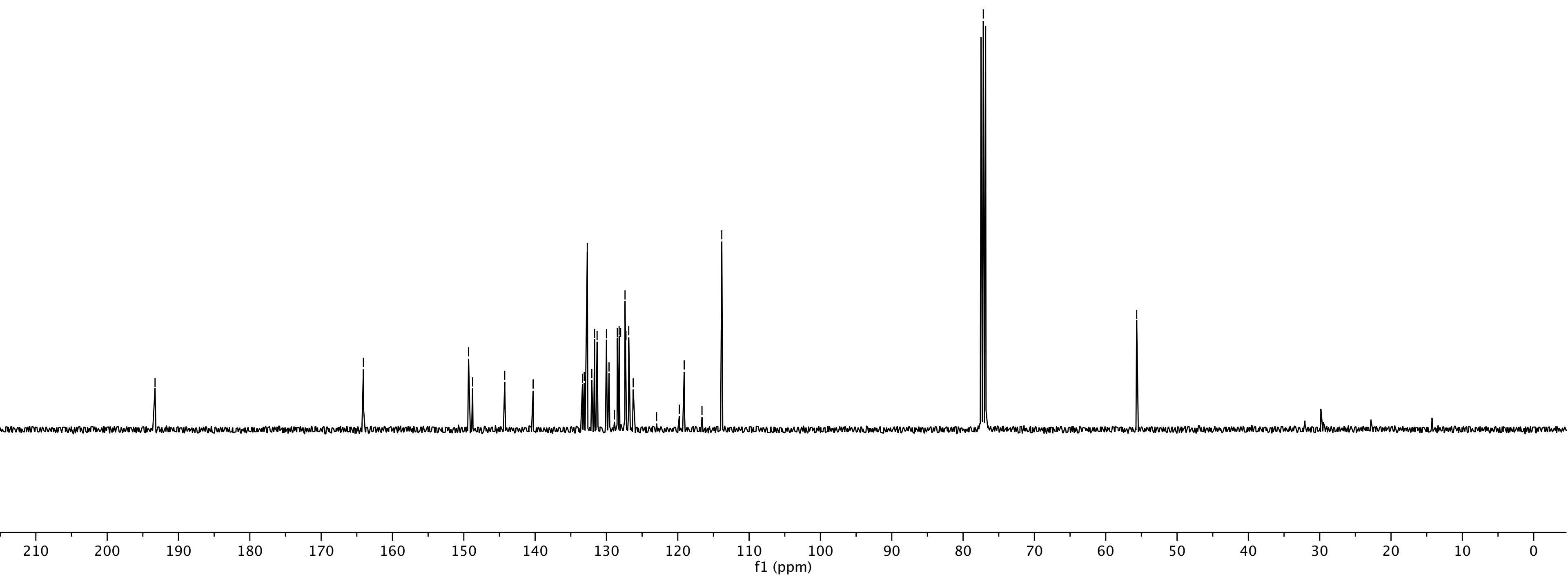

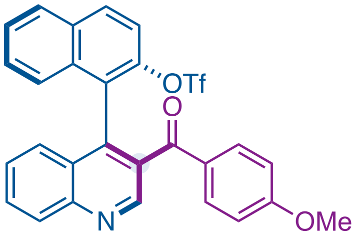

^{19}F NMR of **3af** (376 MHz, $CDCl_3$)

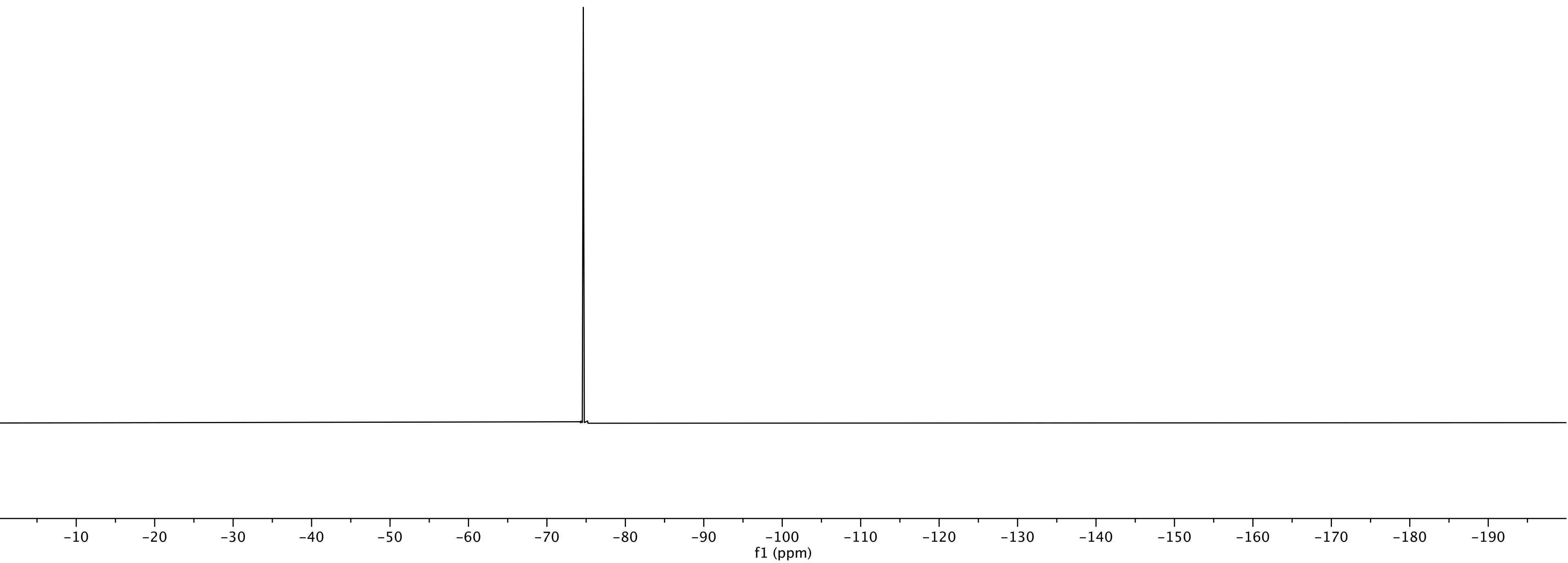

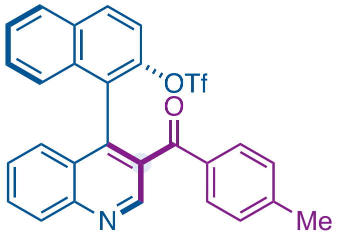

^{1}H NMR of **3ag** (400 MHz, $CDCl_3$)

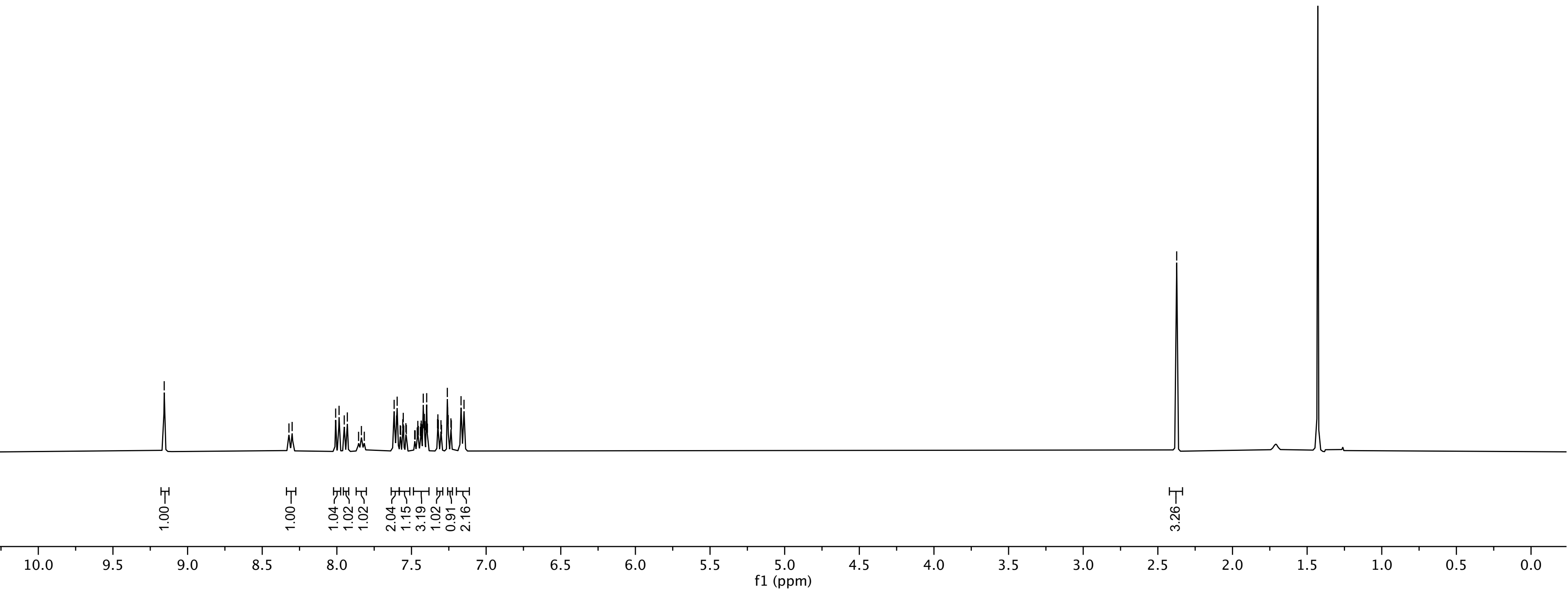

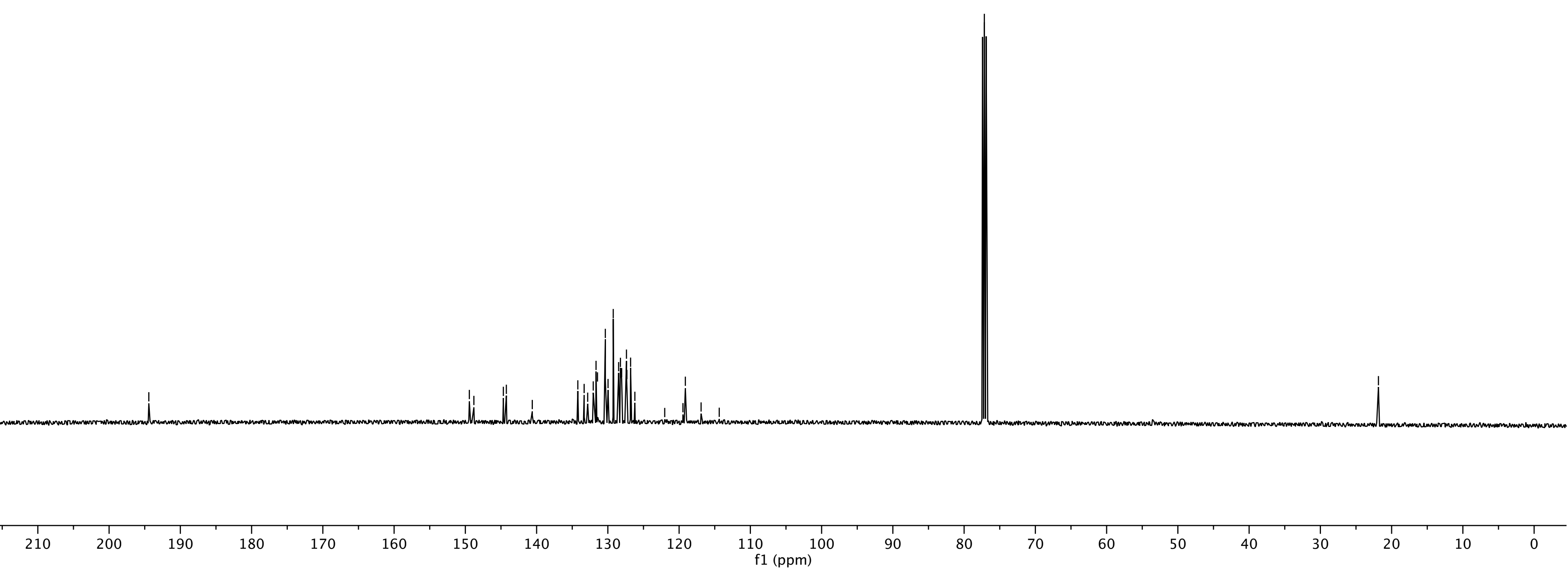

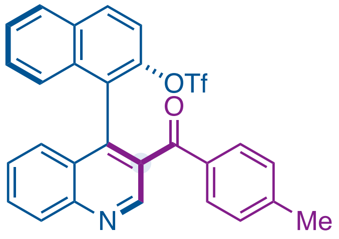

^{13}C NMR of **3ag** (101 MHz, $CDCl_3$)

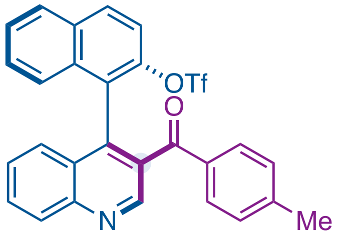

^{19}F NMR of **3ag** (375 MHz, $CDCl_3$)

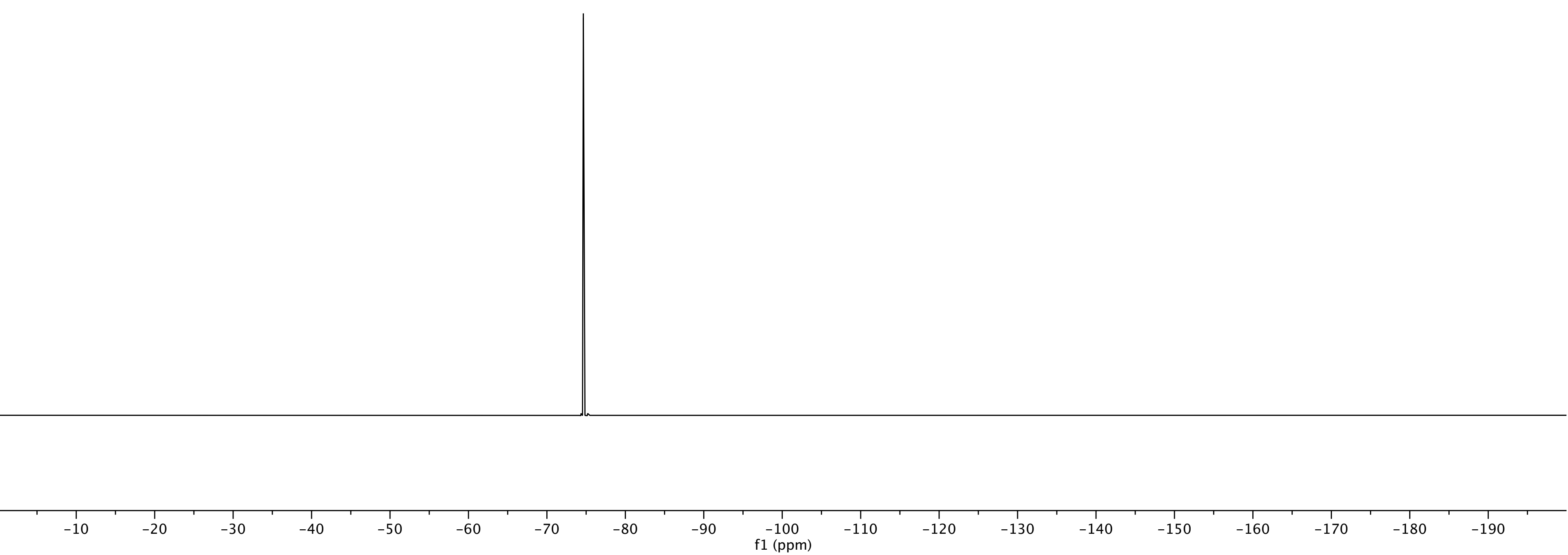

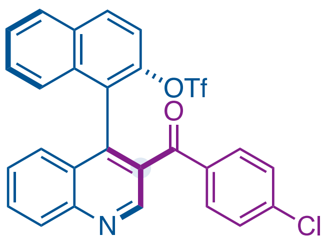

^{1}H NMR of **3ah** (400 MHz, $CDCl_3$)

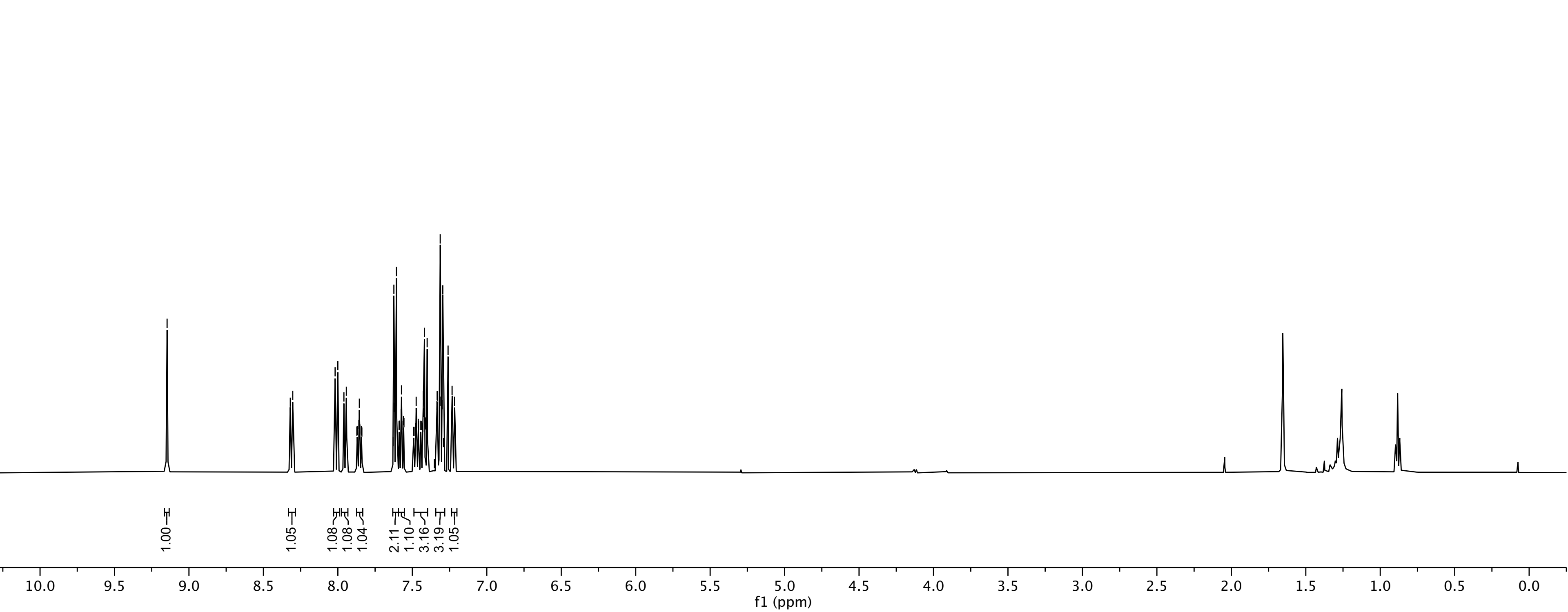

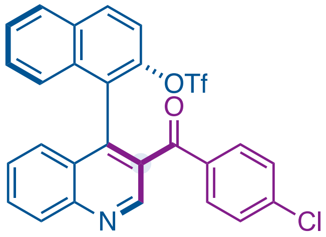

^{13}**C NMR** of **3ah** (101 MHz, $CDCl_3$)

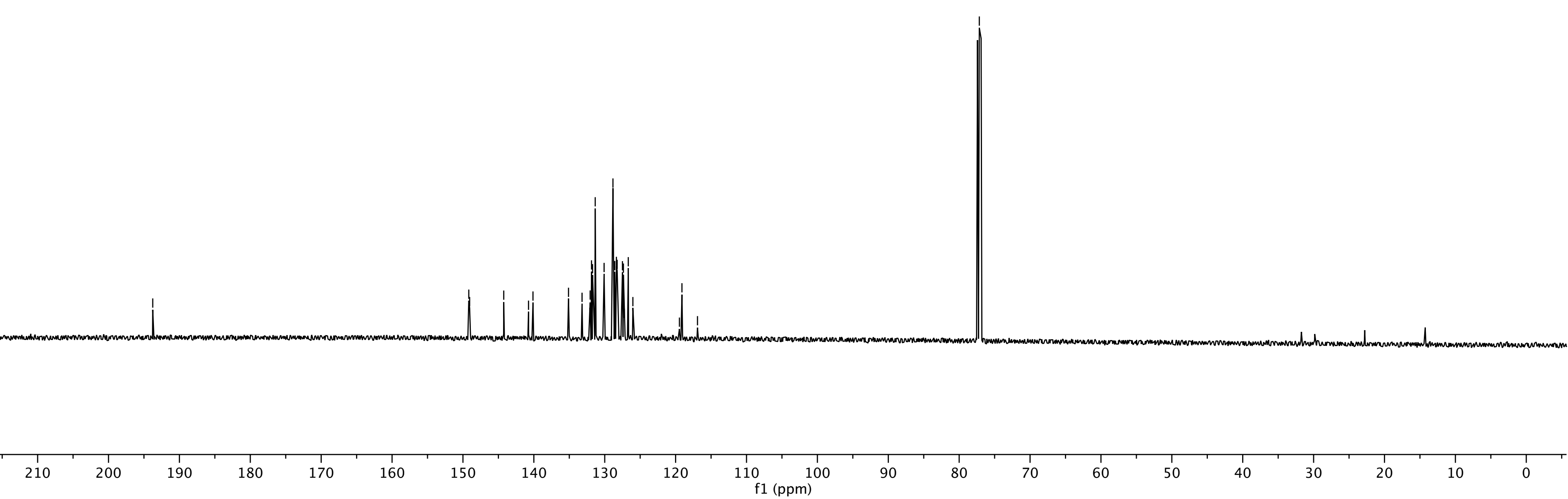

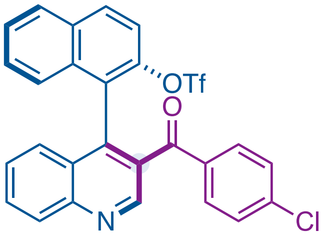

^{19}F NMR of **3ah** (376 MHz, $CDCl_3$)

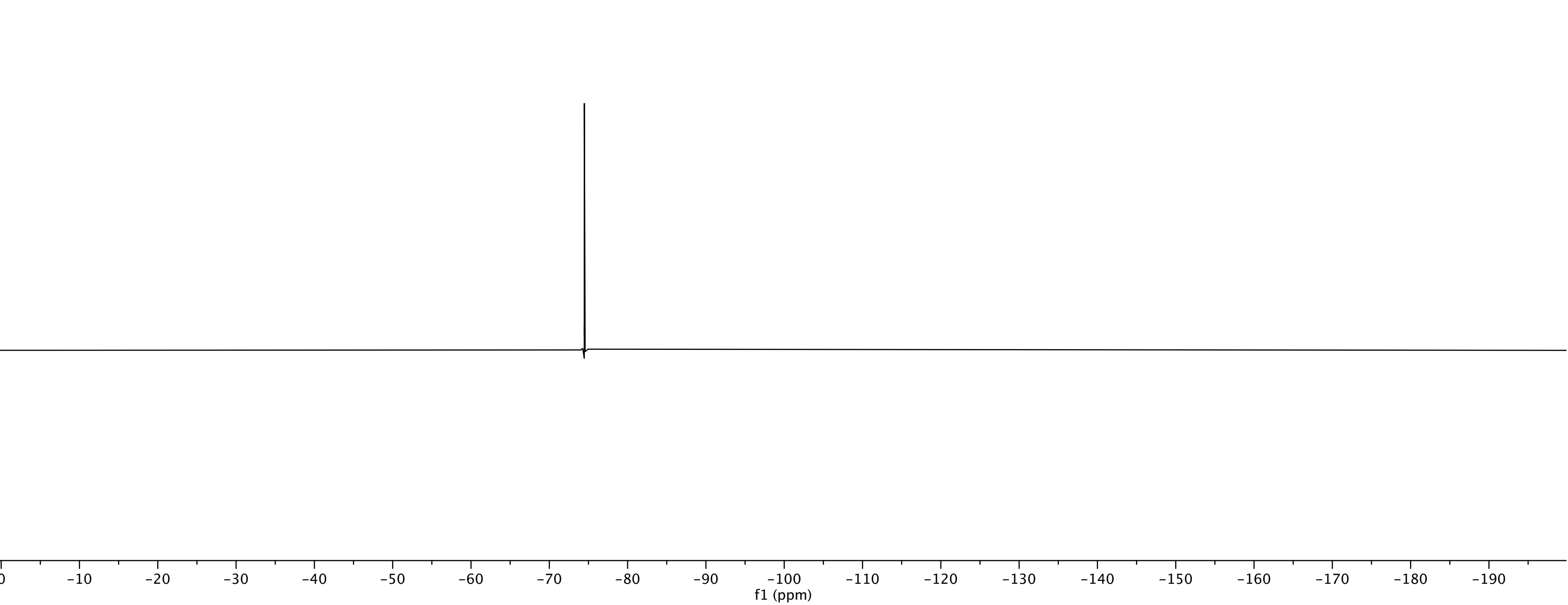

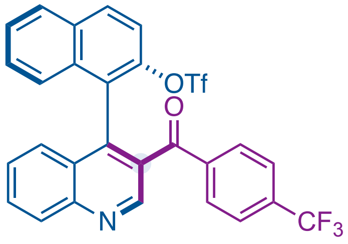

^{1}H NMR of **3ai** (400 MHz, $CDCl_3$)

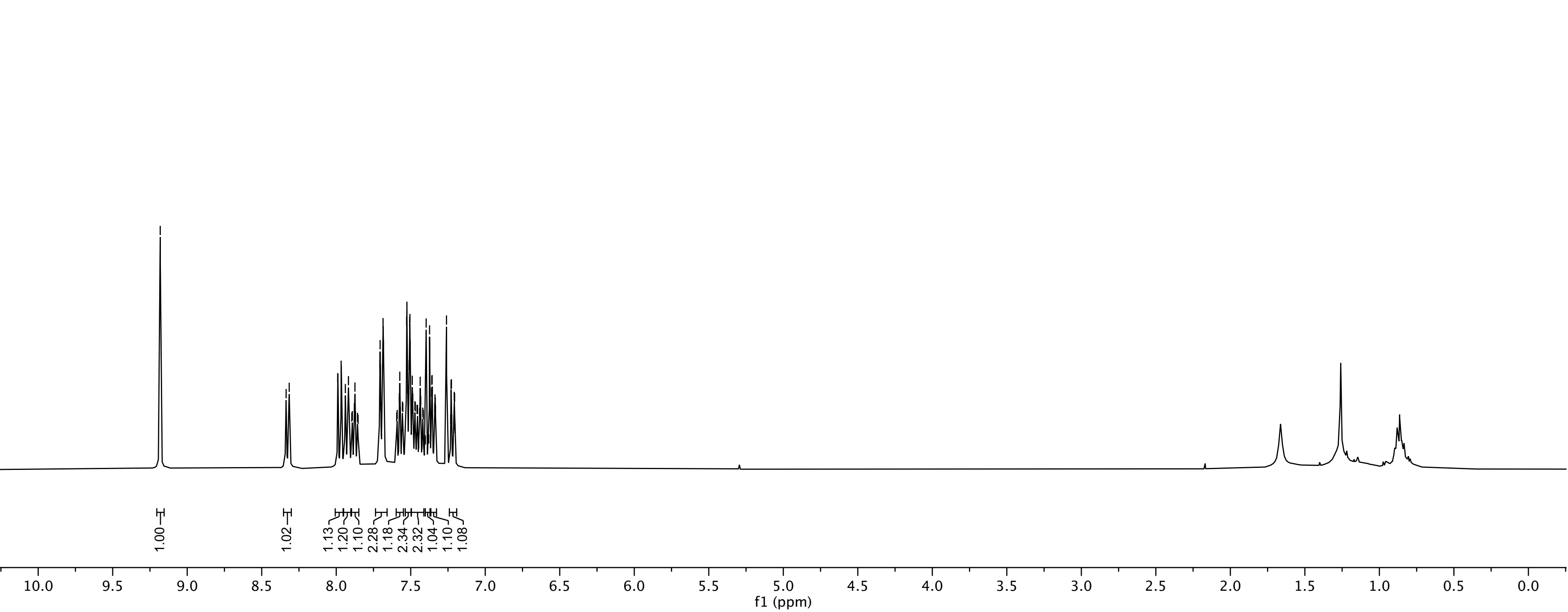

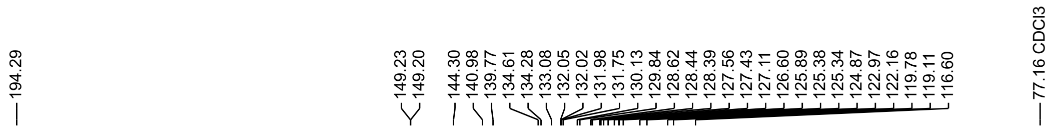

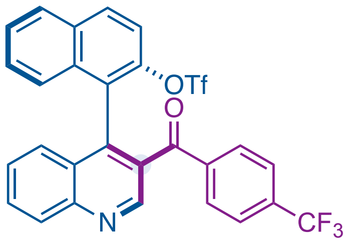

^{13}C NMR of **3ai** (101 MHz, $CDCl_3$)

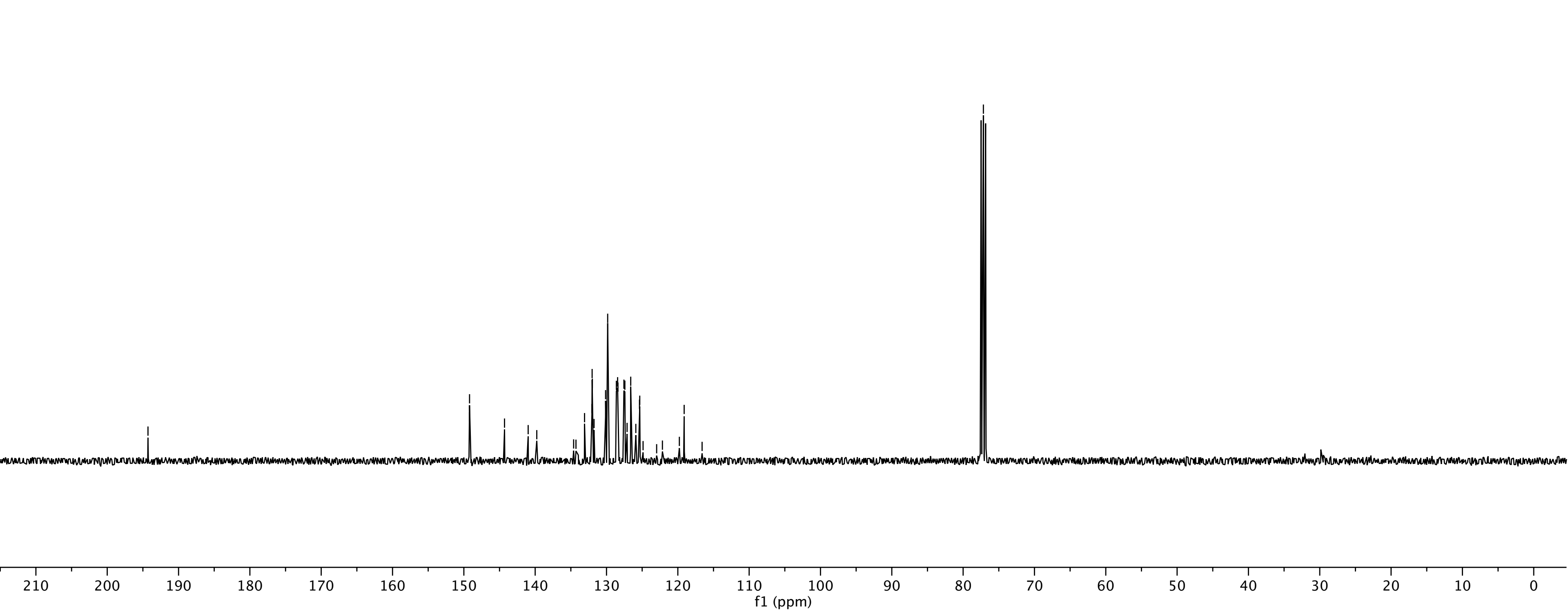

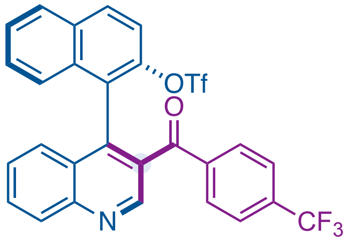

^{19}F NMR of **3ai** (376 MHz, $CDCl_3$)

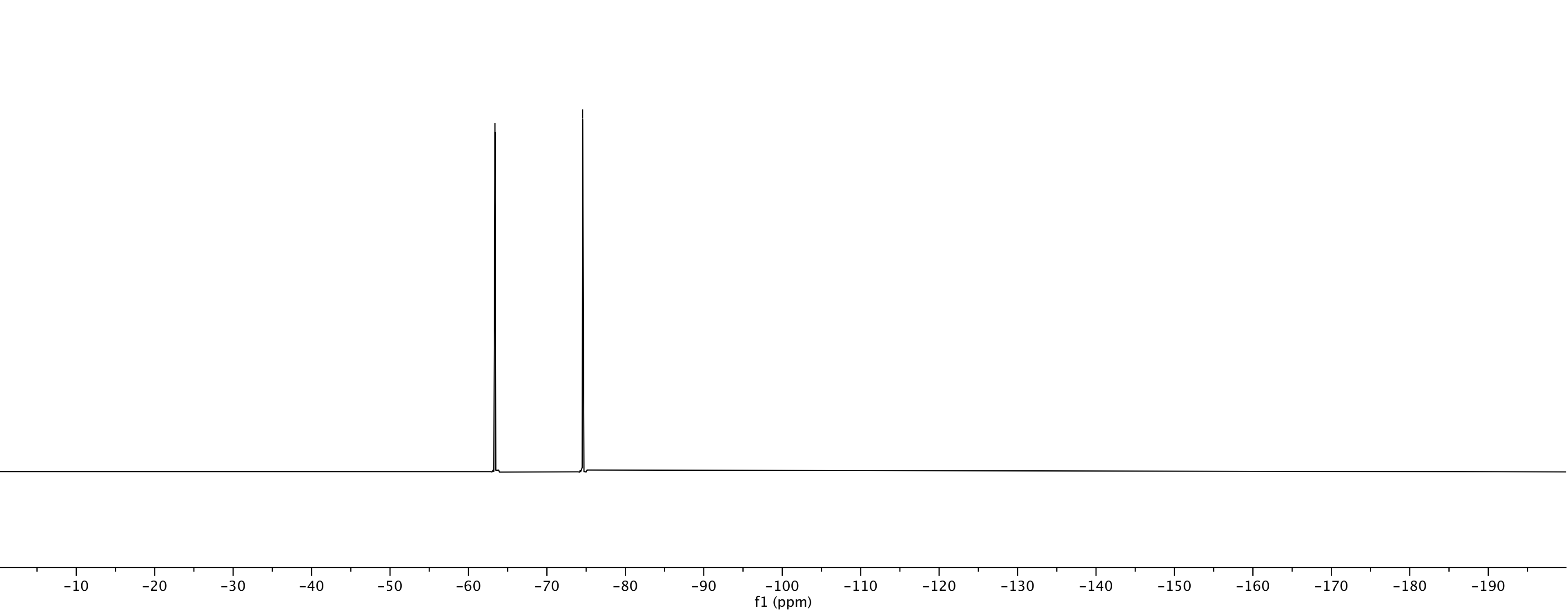

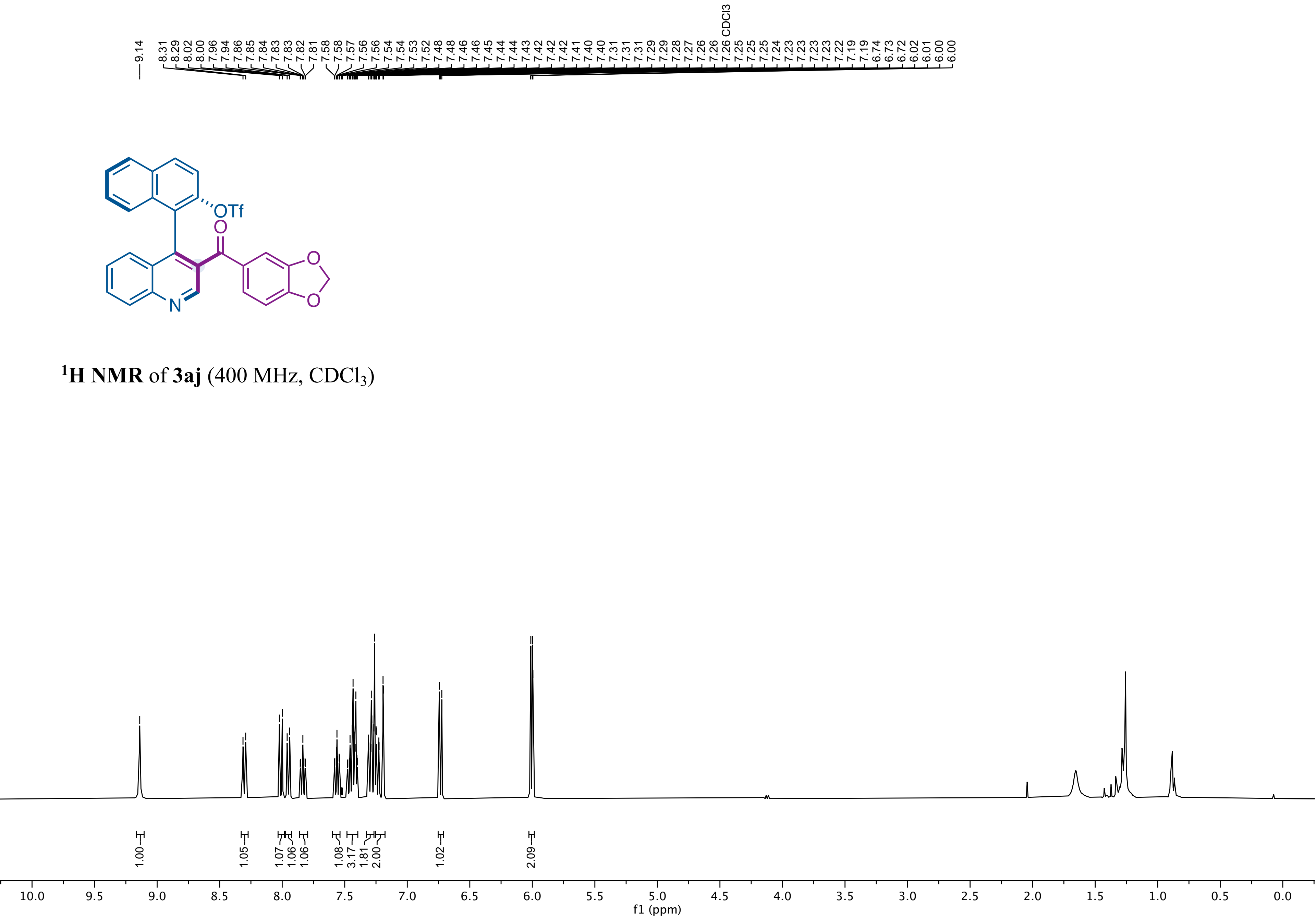

^{1}H NMR of **3aj** (400 MHz, $CDCl_3$)

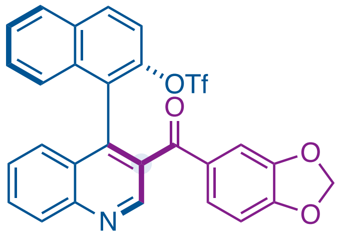

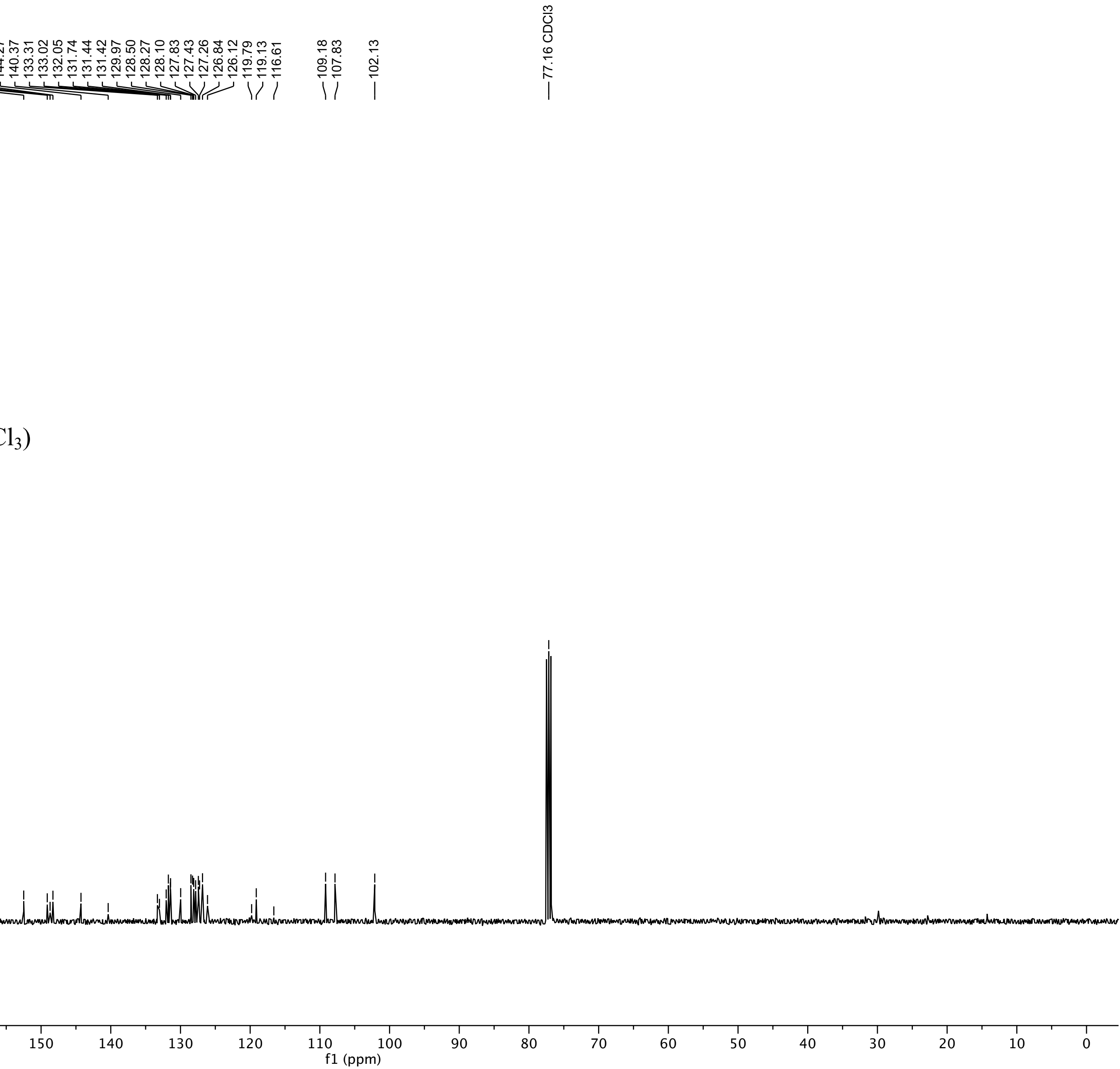

^{13}C NMR of **3aj** (101 MHz, $CDCl_3$)

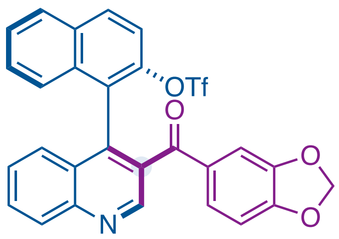

^{19}F NMR of **3aj** (376 MHz, $CDCl_3$)

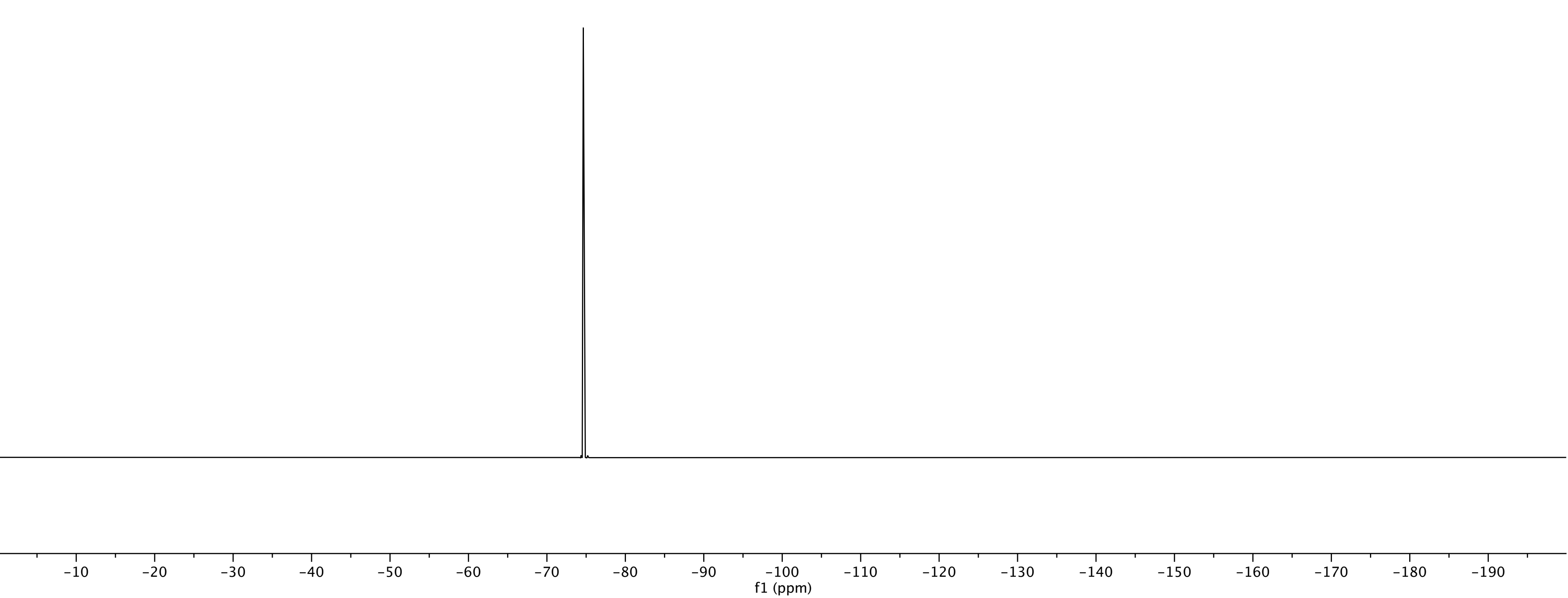

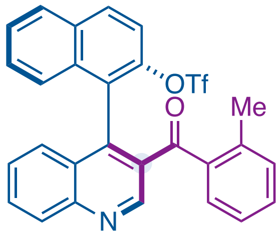

^{1}H NMR of **3ak** (400 MHz, $CDCl_3$)

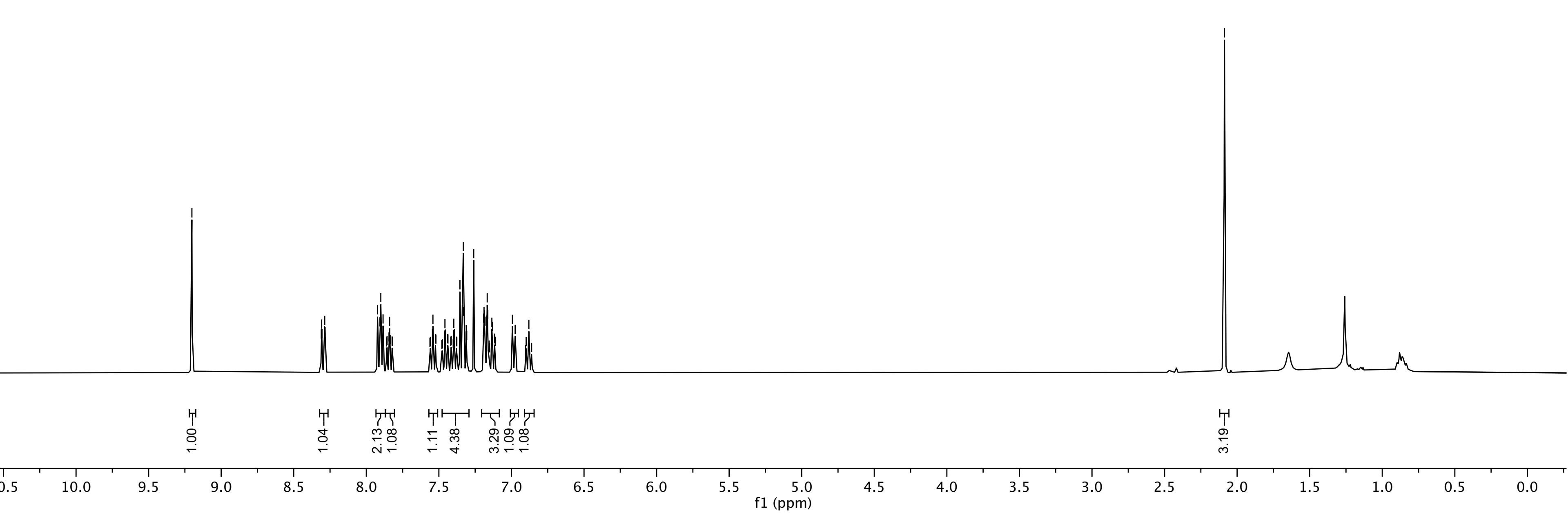

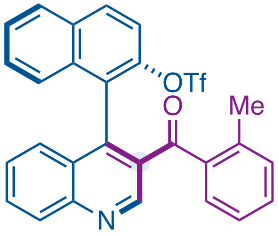

^{13}C NMR of **3ak** (101 MHz, $CDCl_3$)

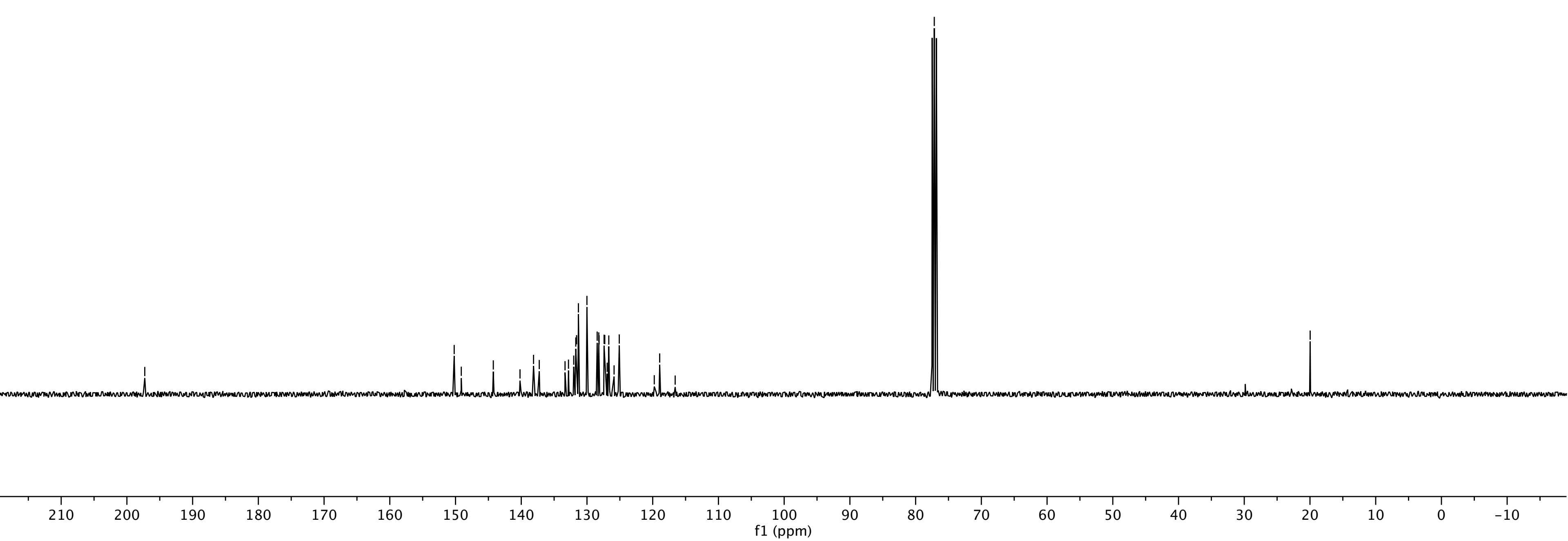

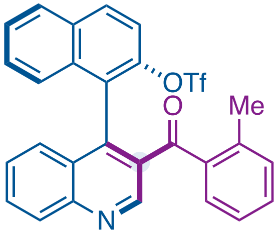

^{19}F NMR of **3ak** (376 MHz, $CDCl_3$)

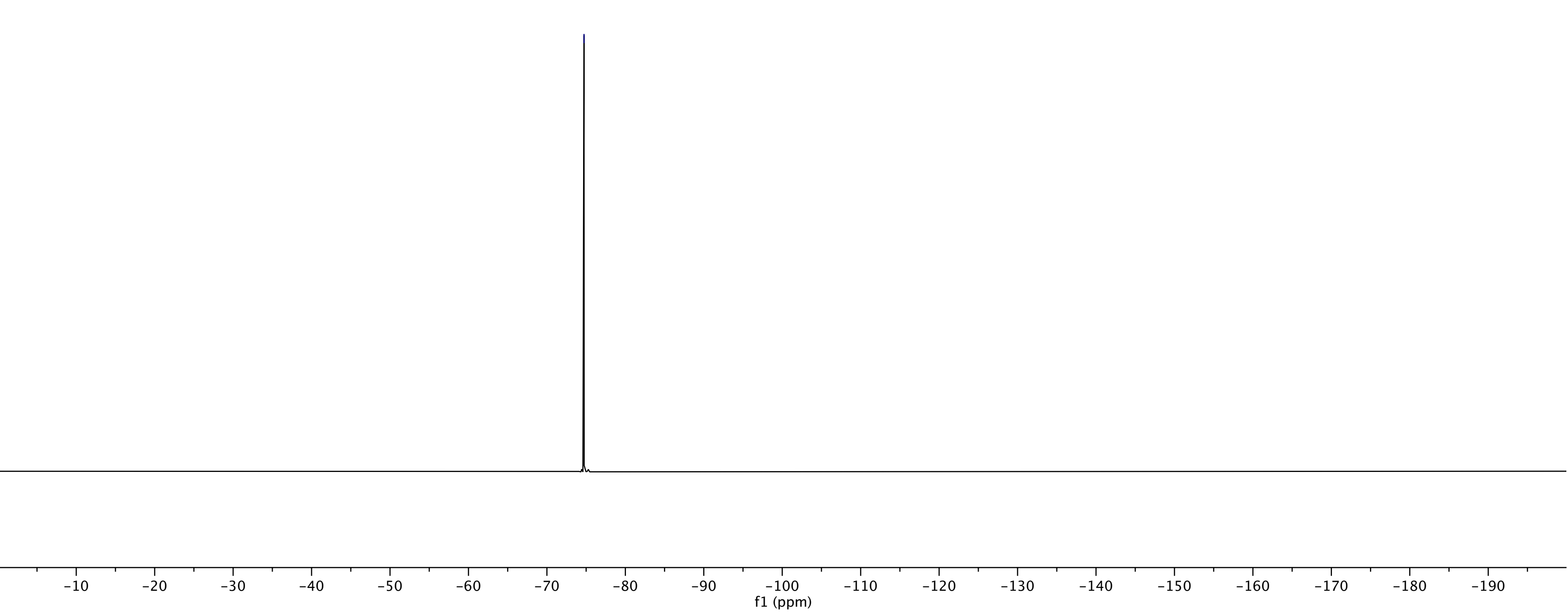

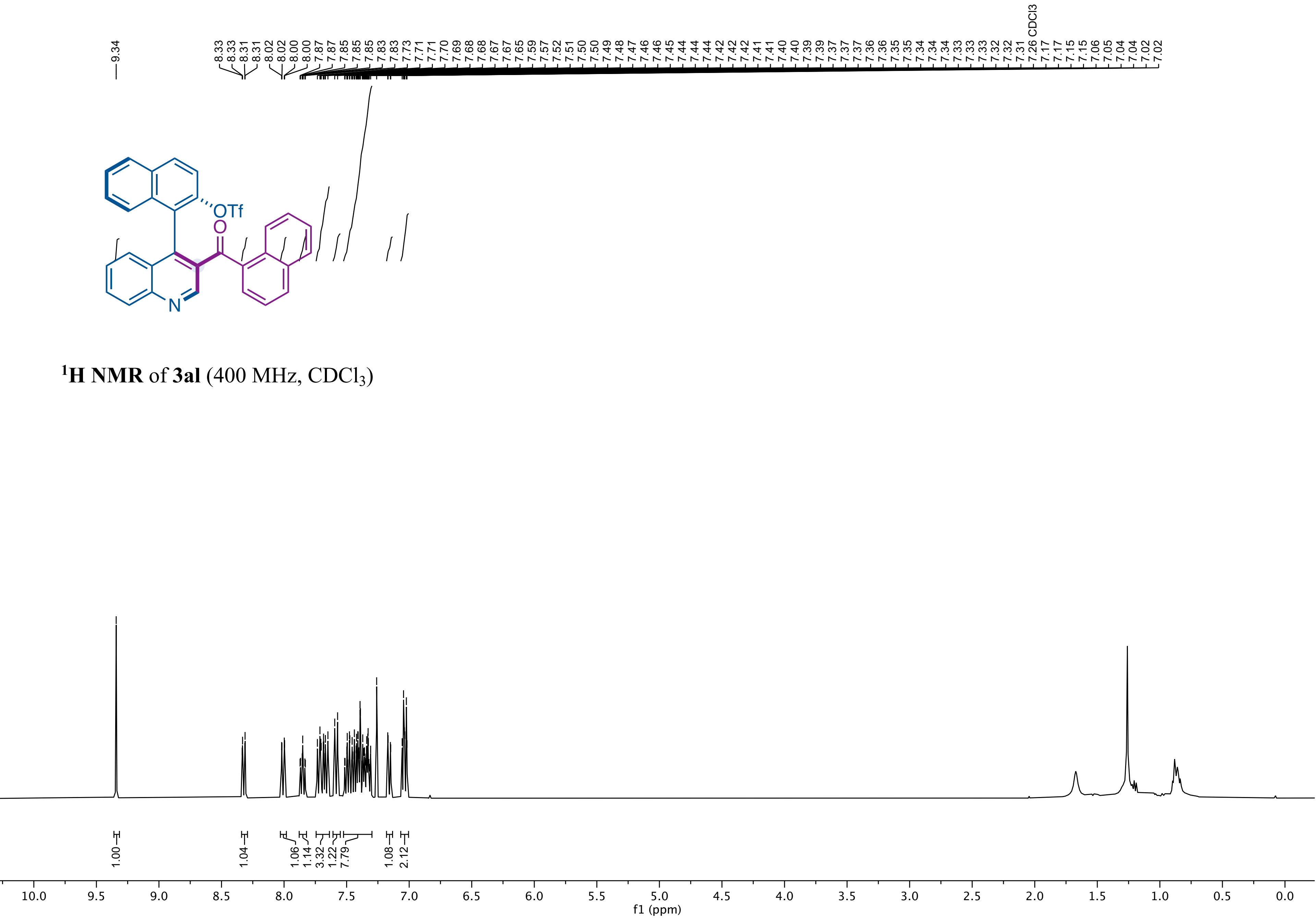

^{1}H NMR of **3al** (400 MHz, $CDCl_3$)

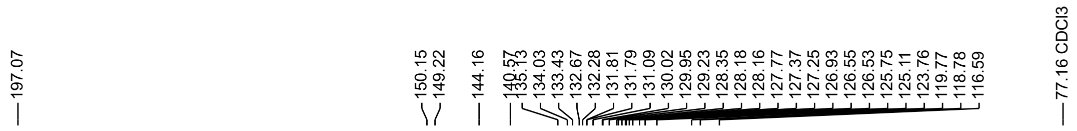

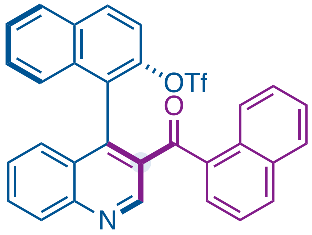

^{13}C NMR of **3al** (101 MHz, $CDCl_3$)

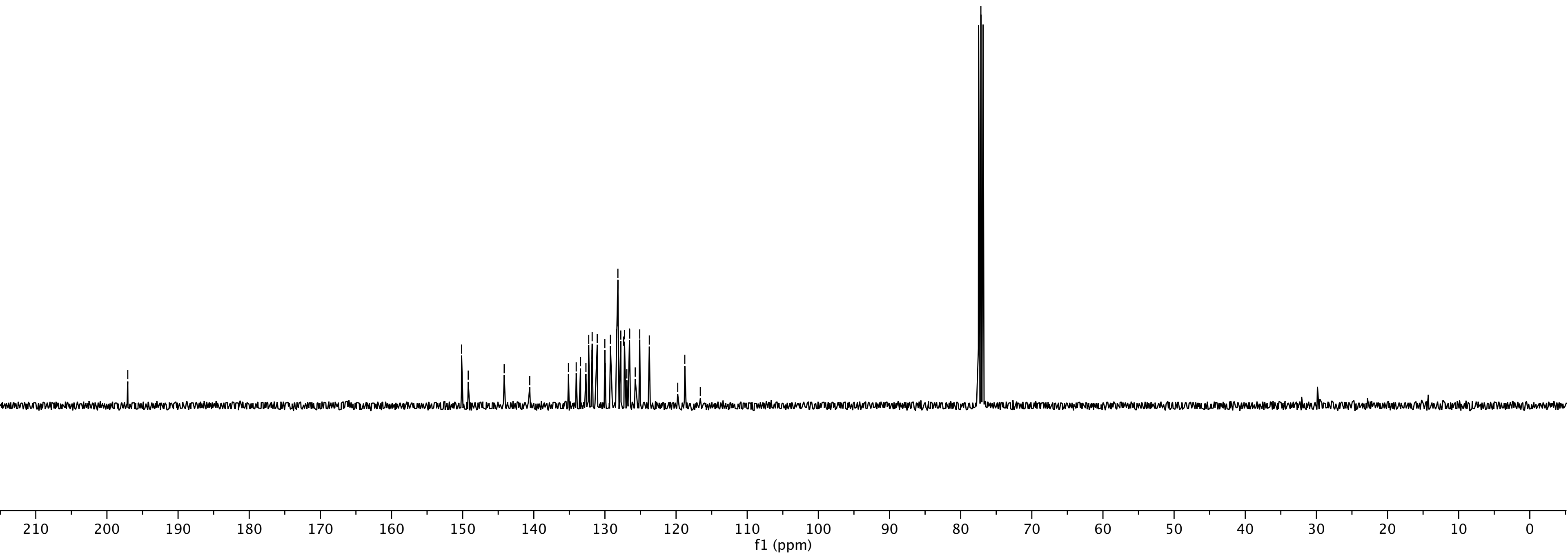

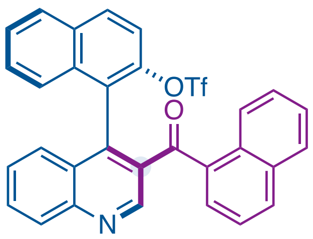

^{19}F NMR of **3al** (376 MHz, $CDCl_3$)

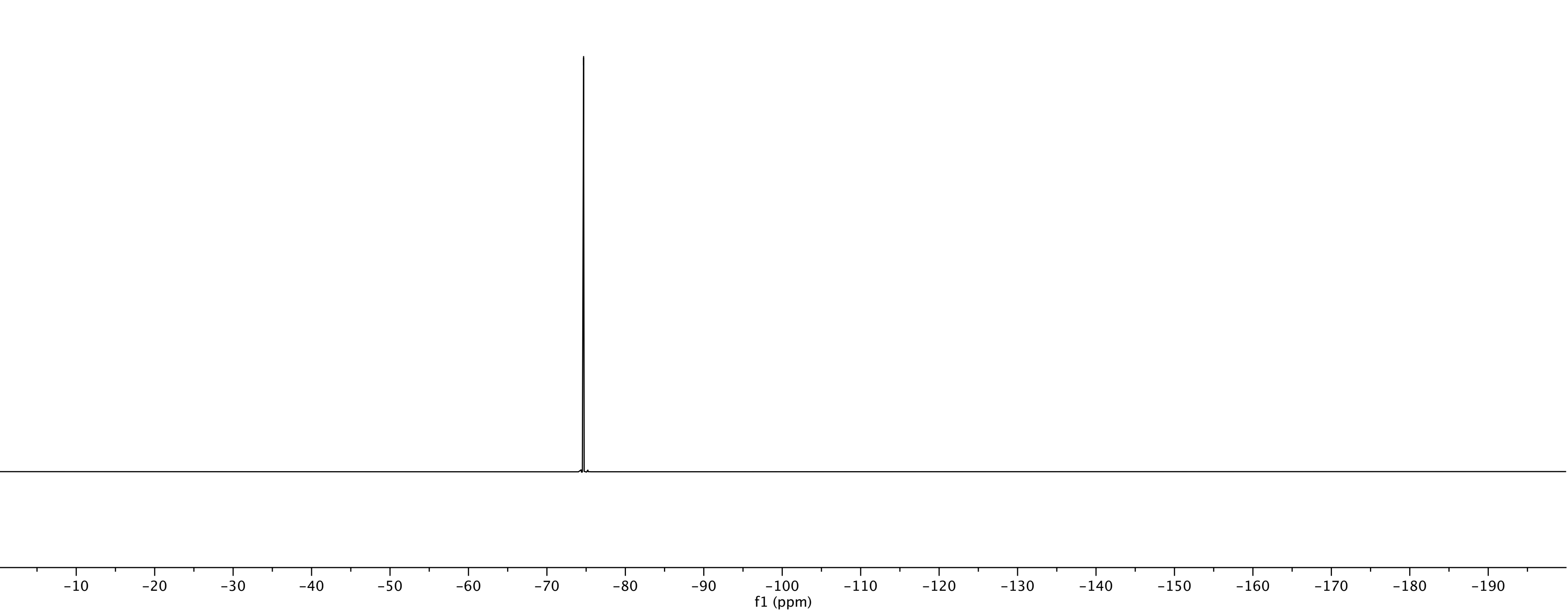

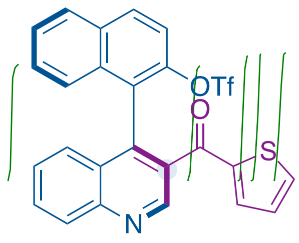

¹H NMR of **3am** (400 MHz, $CDCl_3$)

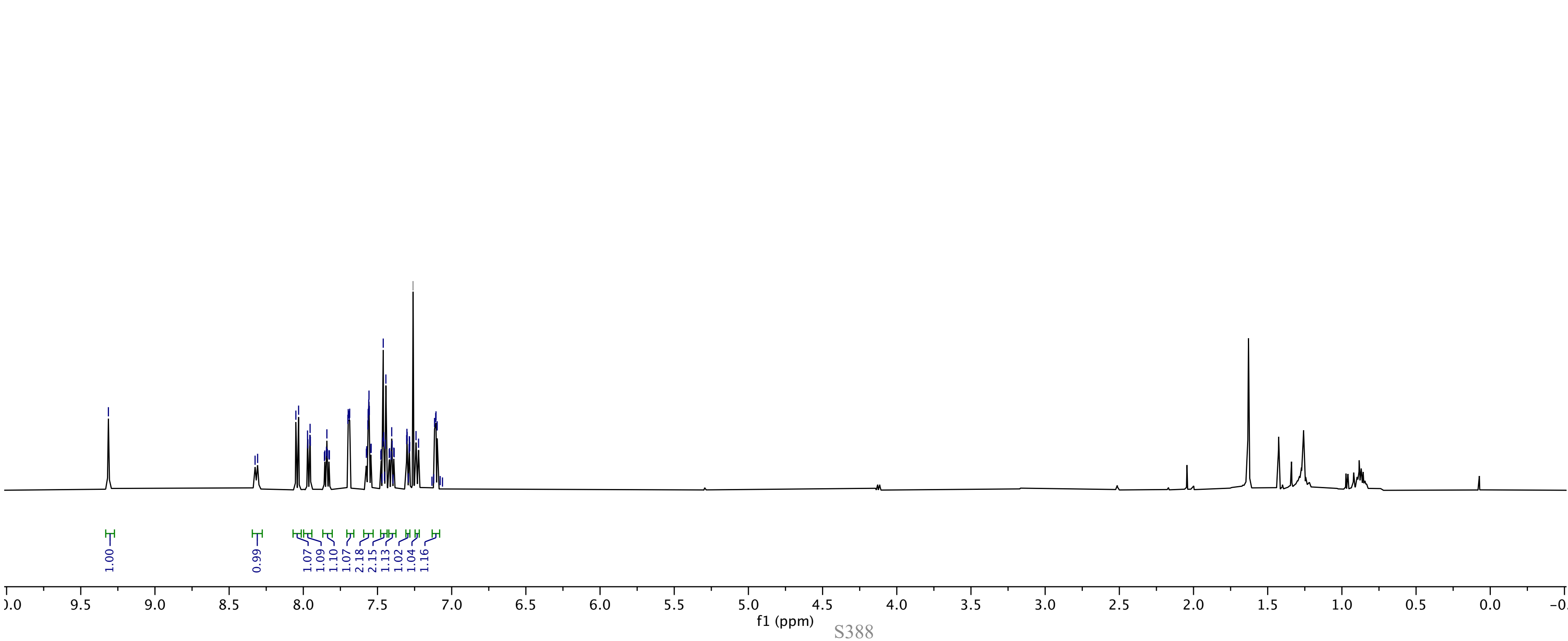

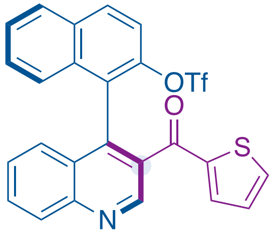

^{13}C NMR of **3am** (101 MHz, $CDCl_3$)

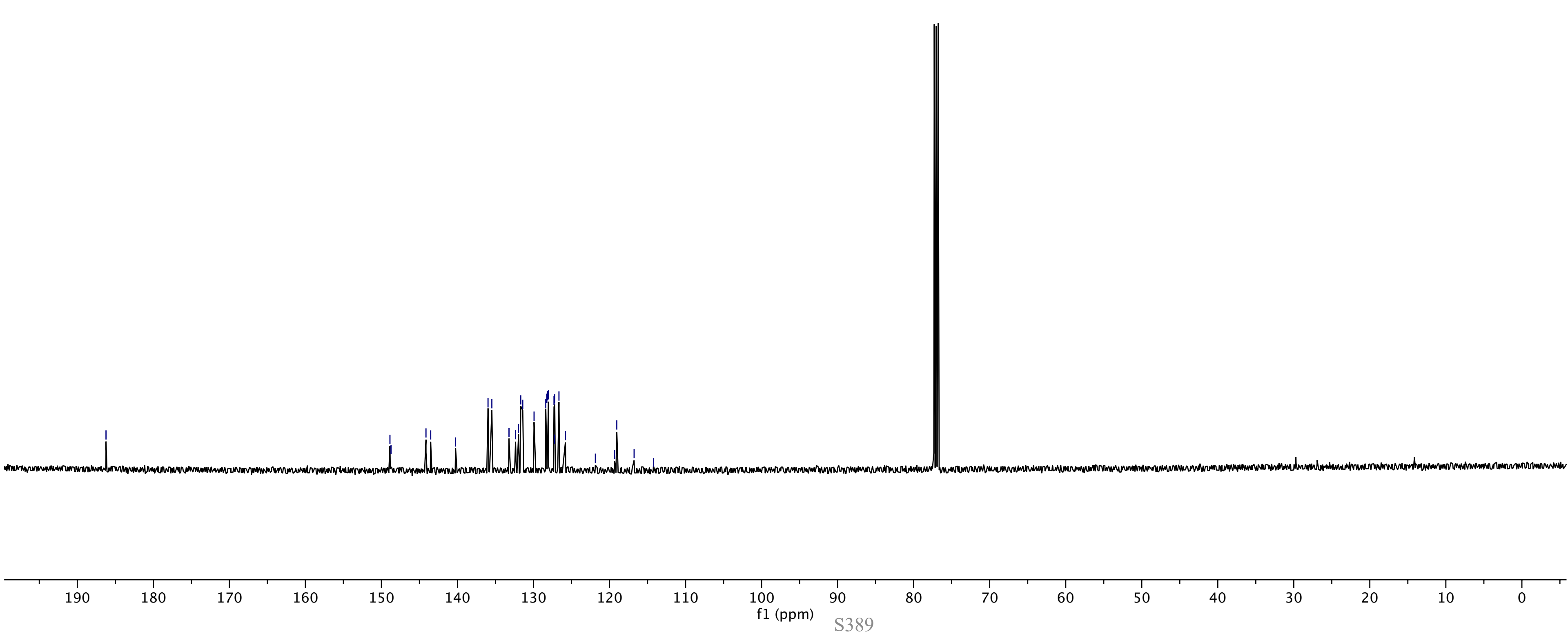

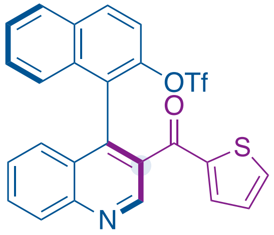

^{19}F NMR of **3am** (376 MHz, $CDCl_3$)

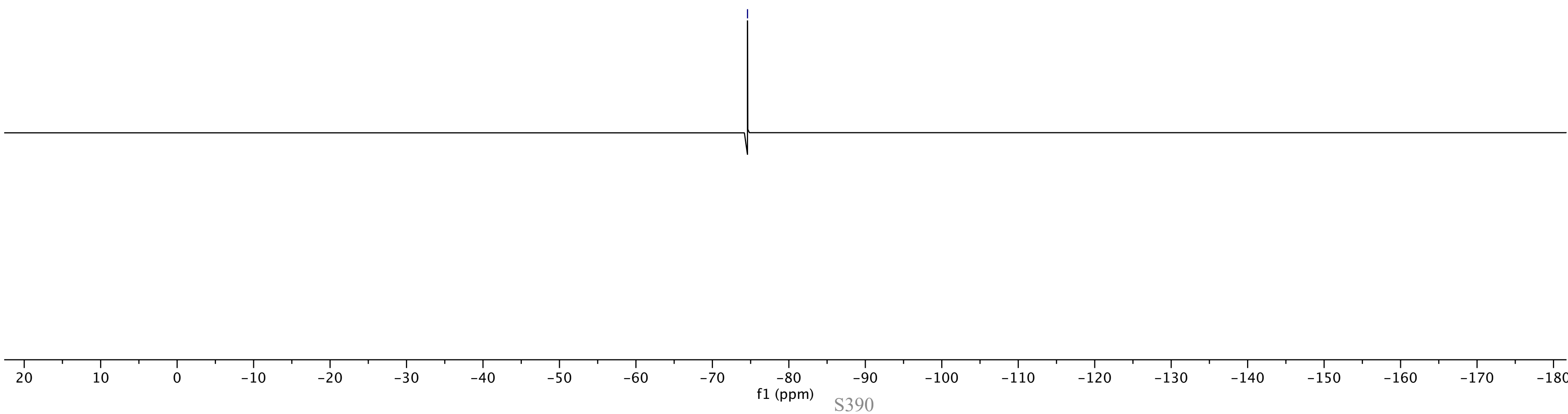

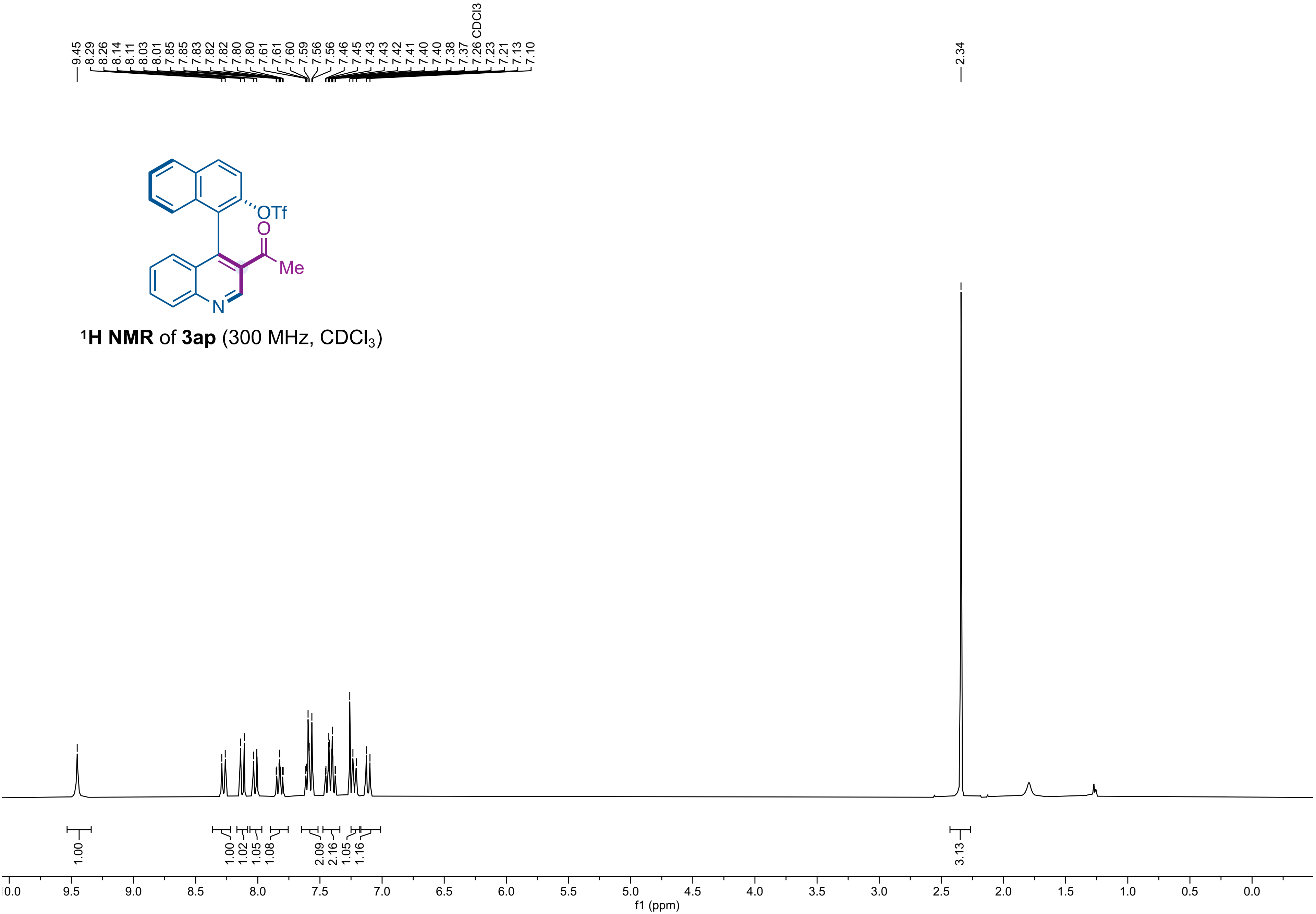

^{1}**H NMR** of **3ap** (300 MHz, $CDCl_3$)

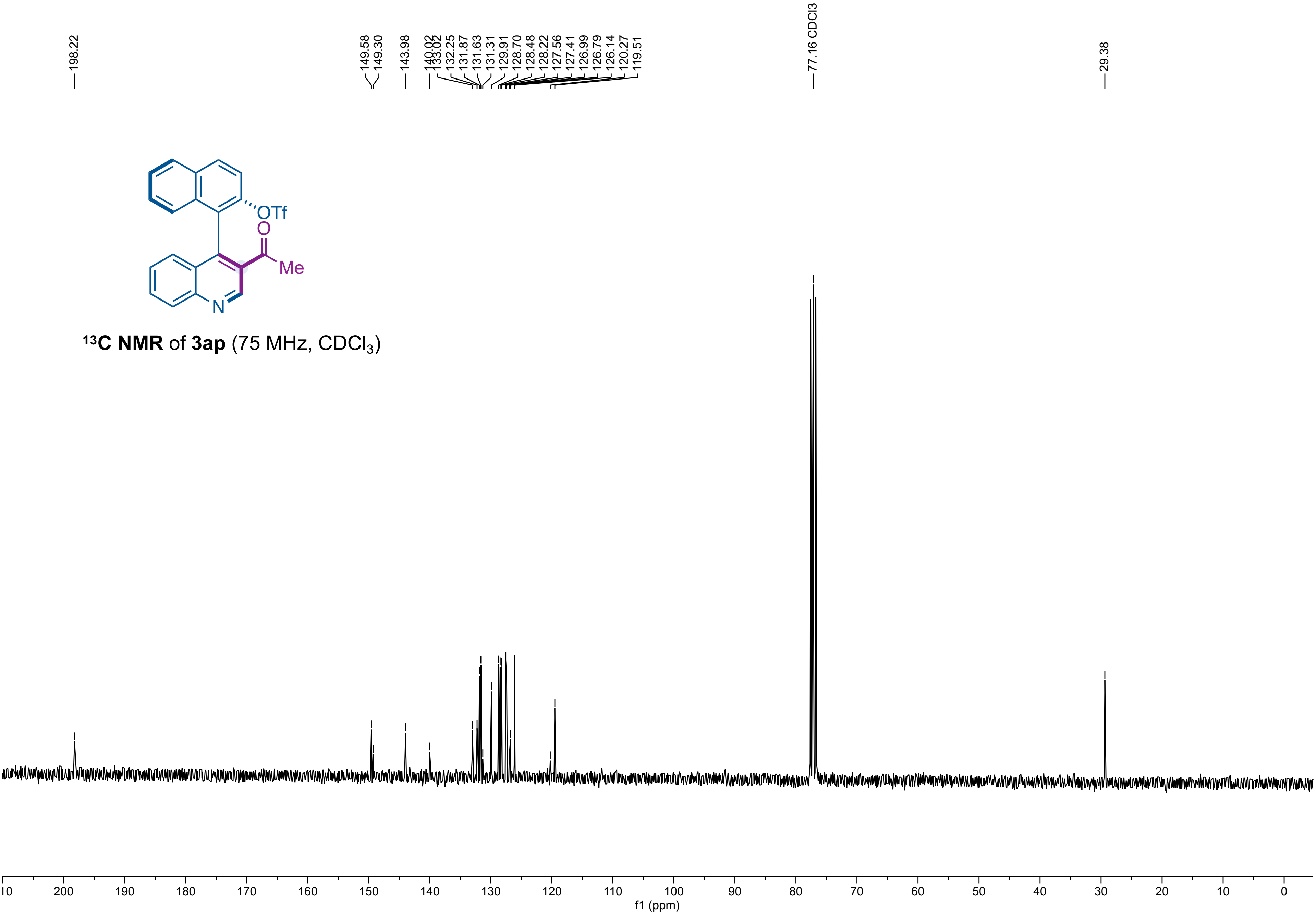

^{13}C NMR of **3ap** (75 MHz, $CDCl_3$)

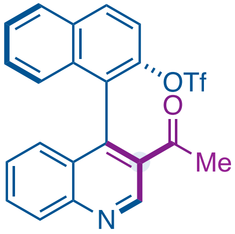

^{19}F NMR of **3ap** (282 MHz, $CDCl_3$)

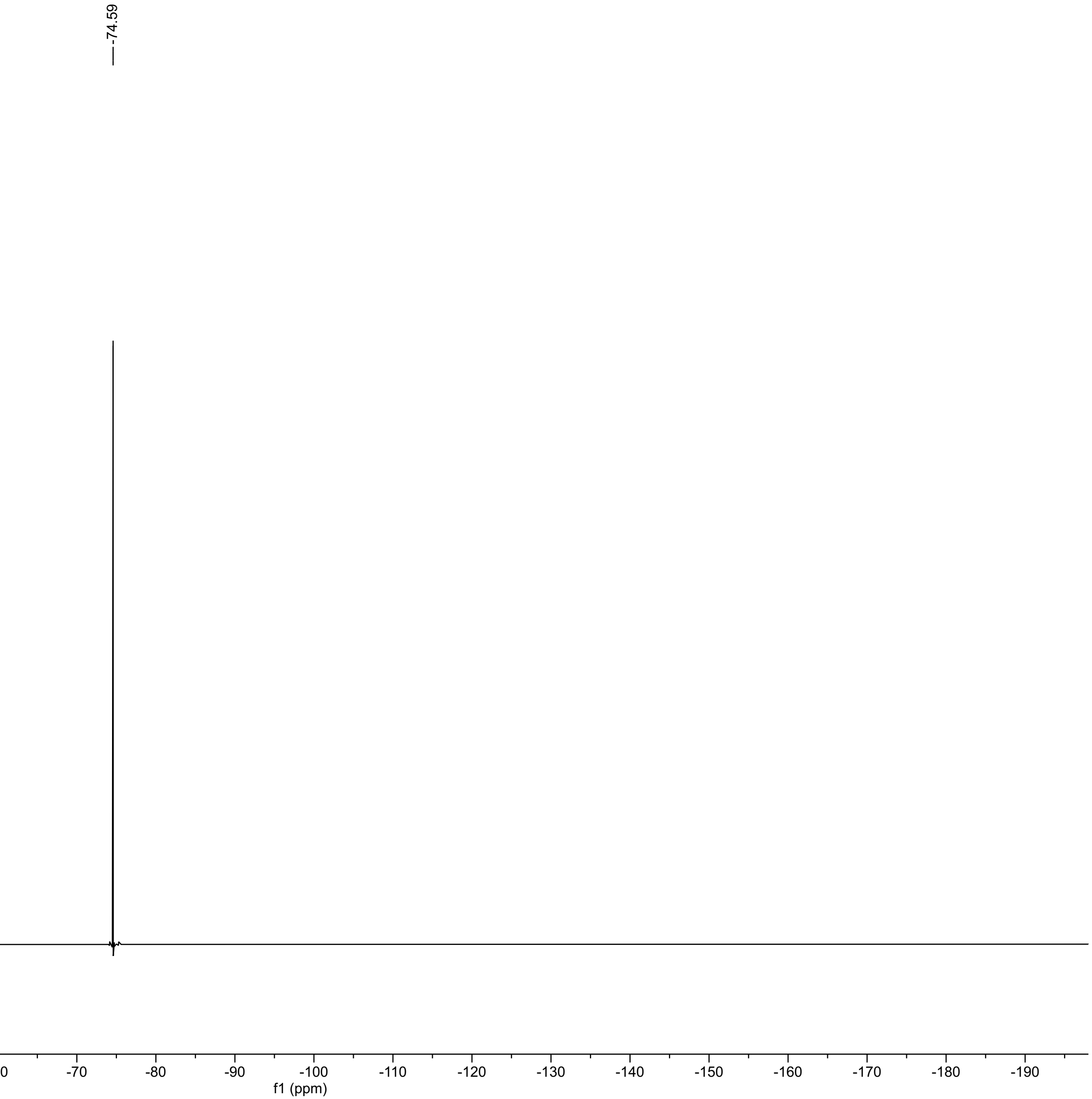

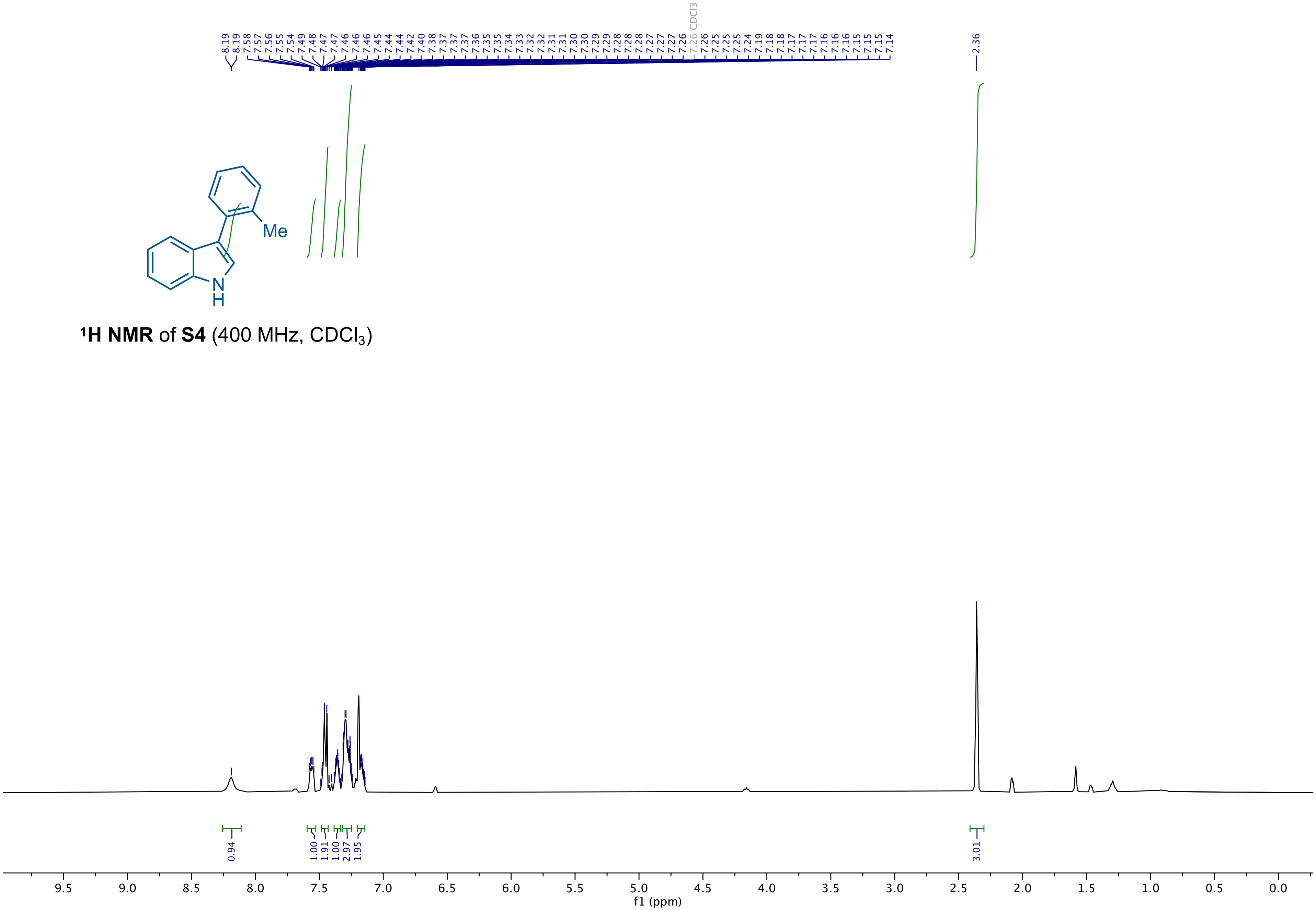

¹H NMR of **S4** (400 MHz, $CDCl_3$)

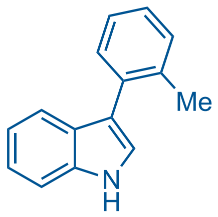

^{13}C NMR of **S4** (101 MHz, $CDCl_3$)

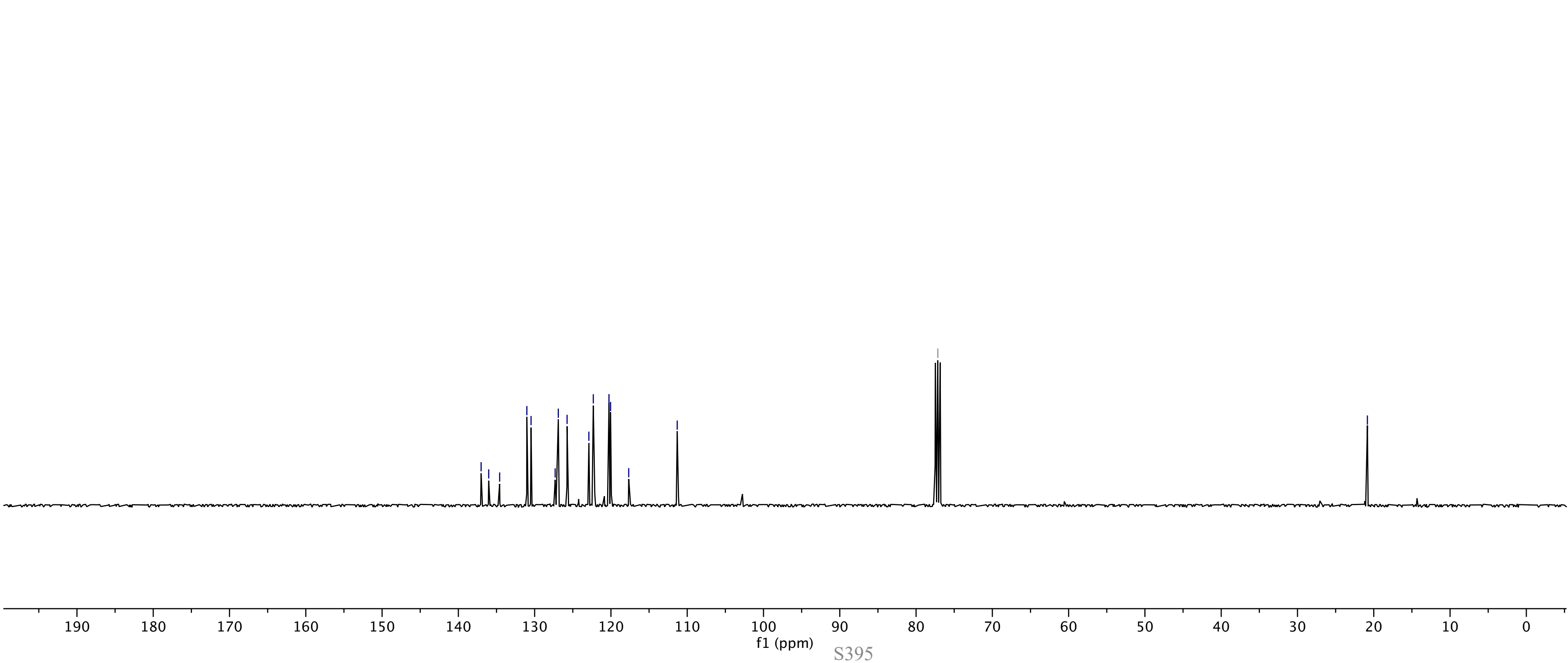

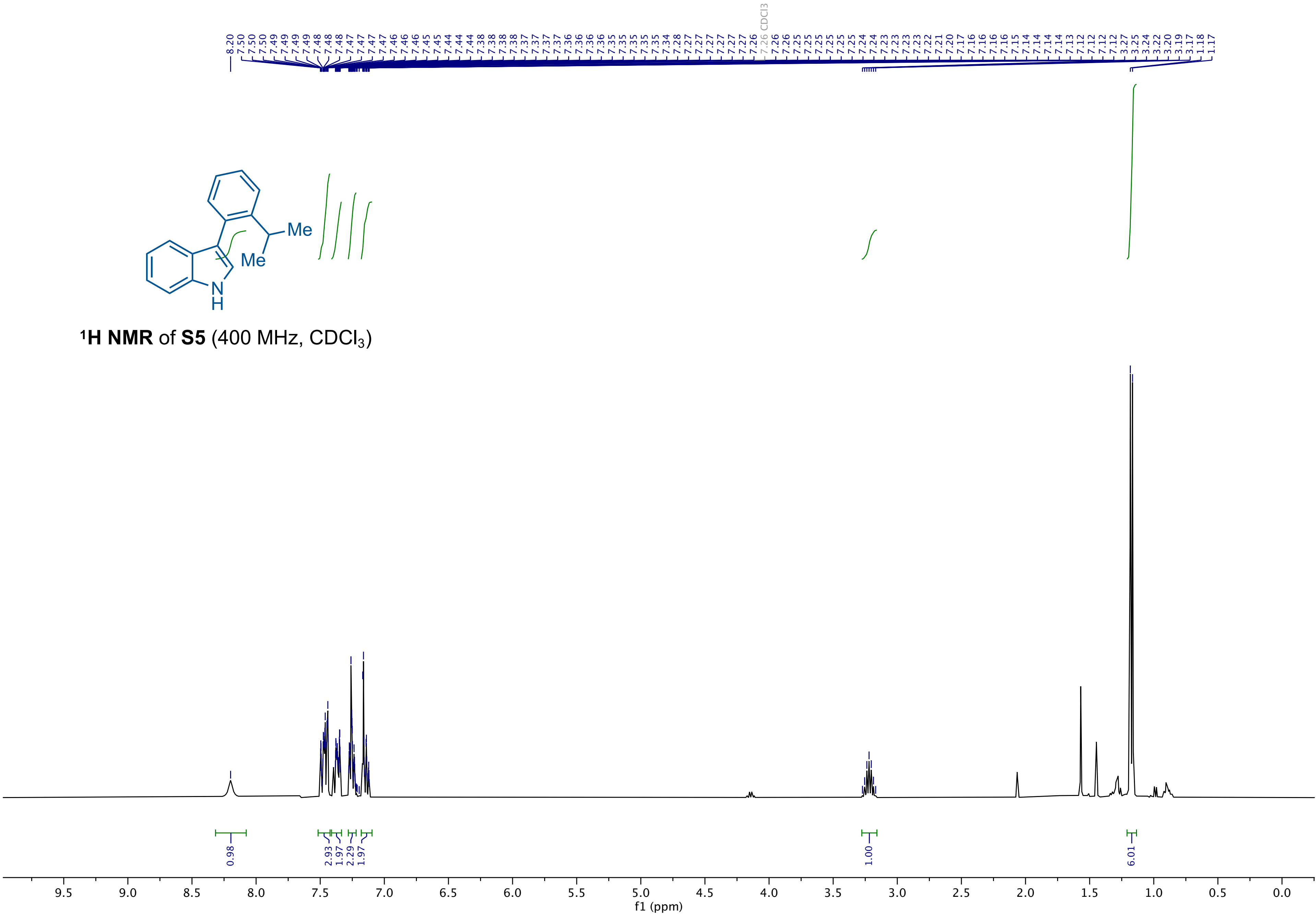

¹H NMR of S5 (400 MHz, CDCl₃)
Me
Me
N
H
8.20
7.26 CDCl3
3.27
3.25
3.24
3.22
3.20
3.19
3.17
1.18
1.17
0.98
2.93
1.97
2.29
1.97
1.00
6.01
f1 (ppm)

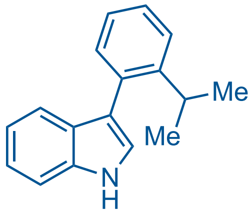

^{13}C NMR of **S5** (101 MHz, $CDCl_3$)

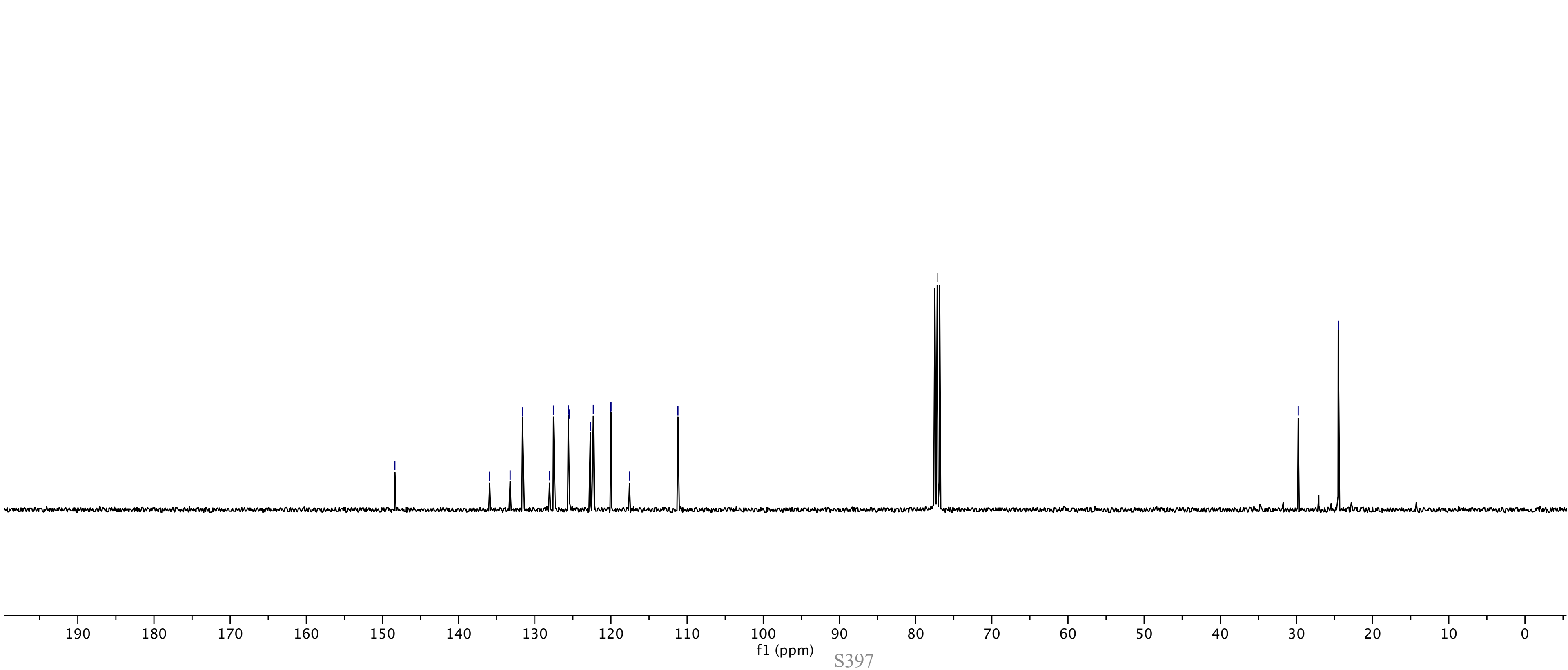

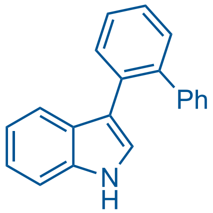

¹H NMR of **S6** (400 MHz, $CDCl_3$)

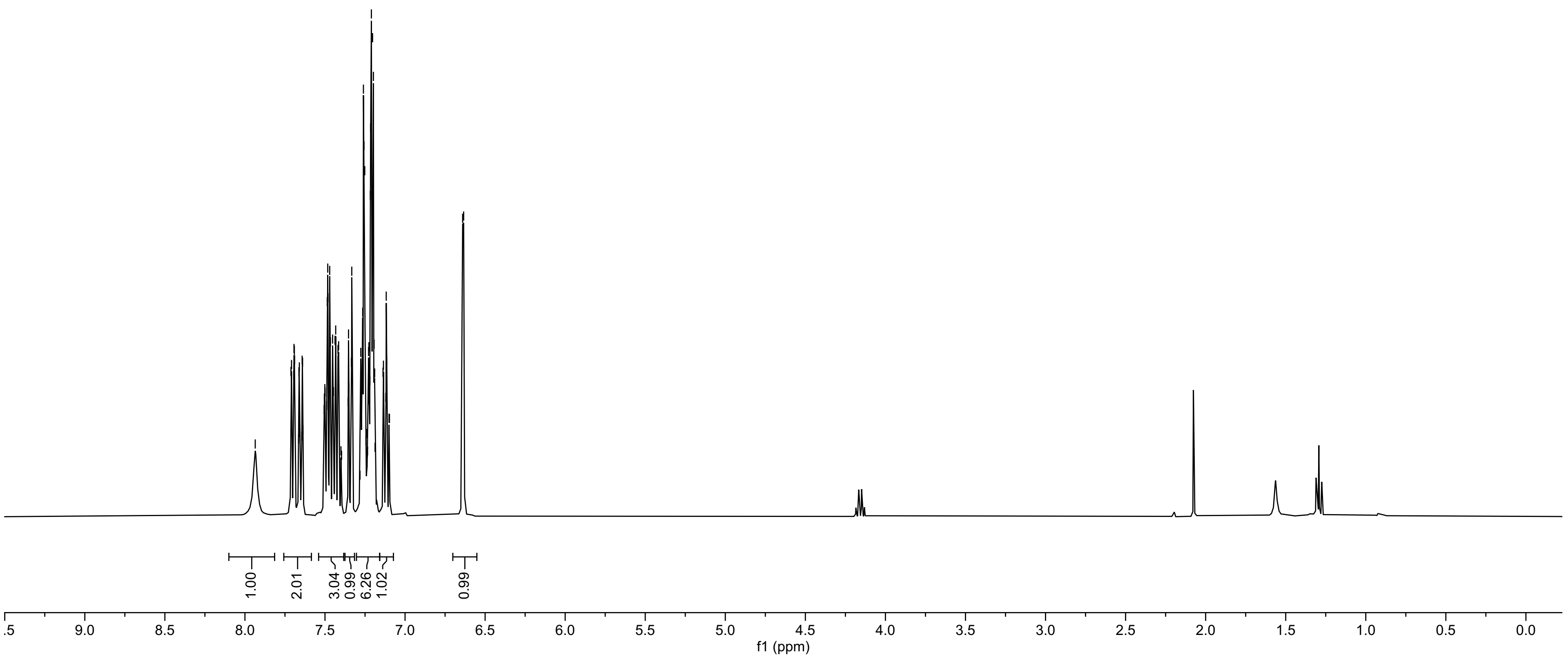

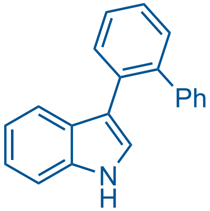

^{13}C NMR of **S6** (101 MHz, $CDCl_3$)

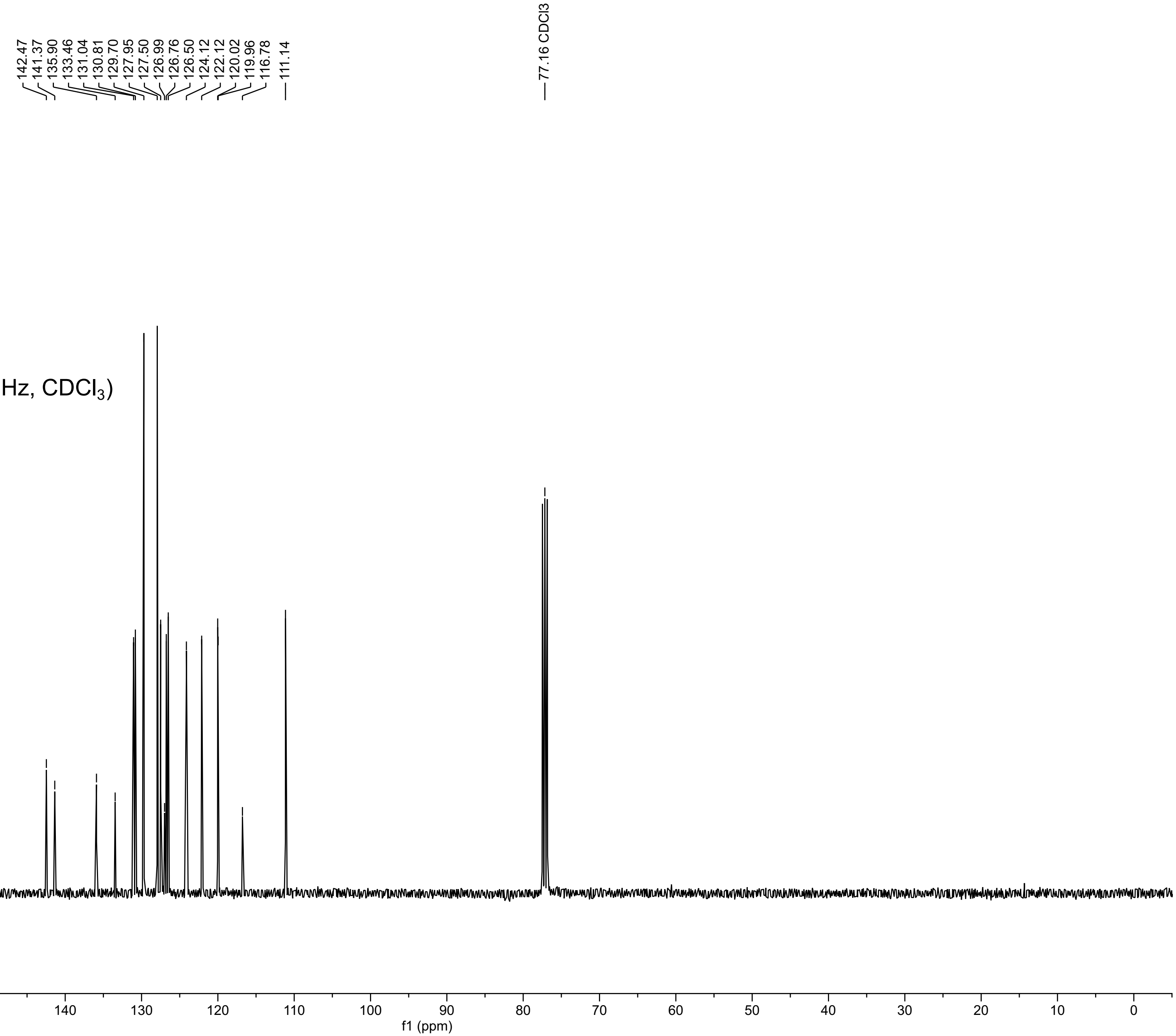

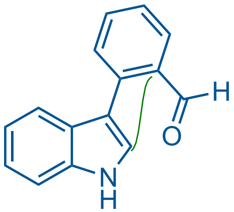

¹H NMR of **S7** (400 MHz, $CDCl_3$)

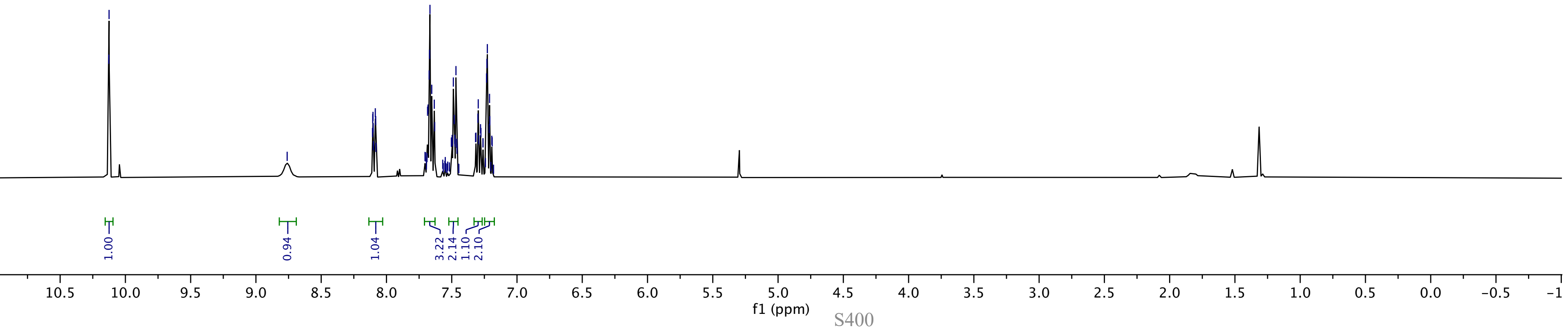

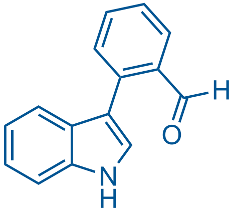

^{13}C NMR of **S7** (101 MHz, $CDCl_3$)

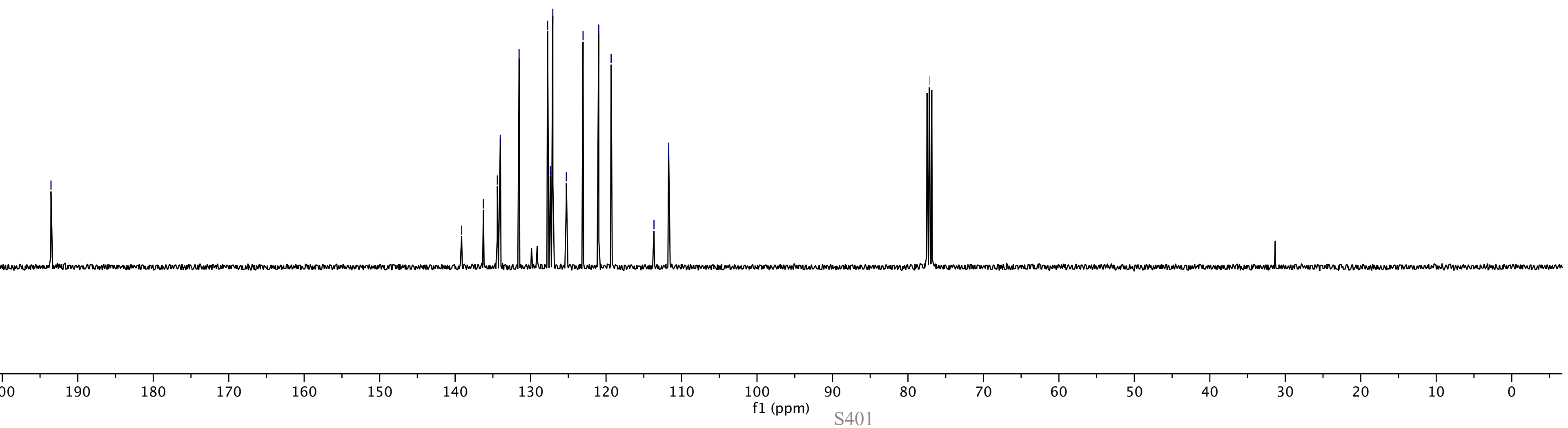

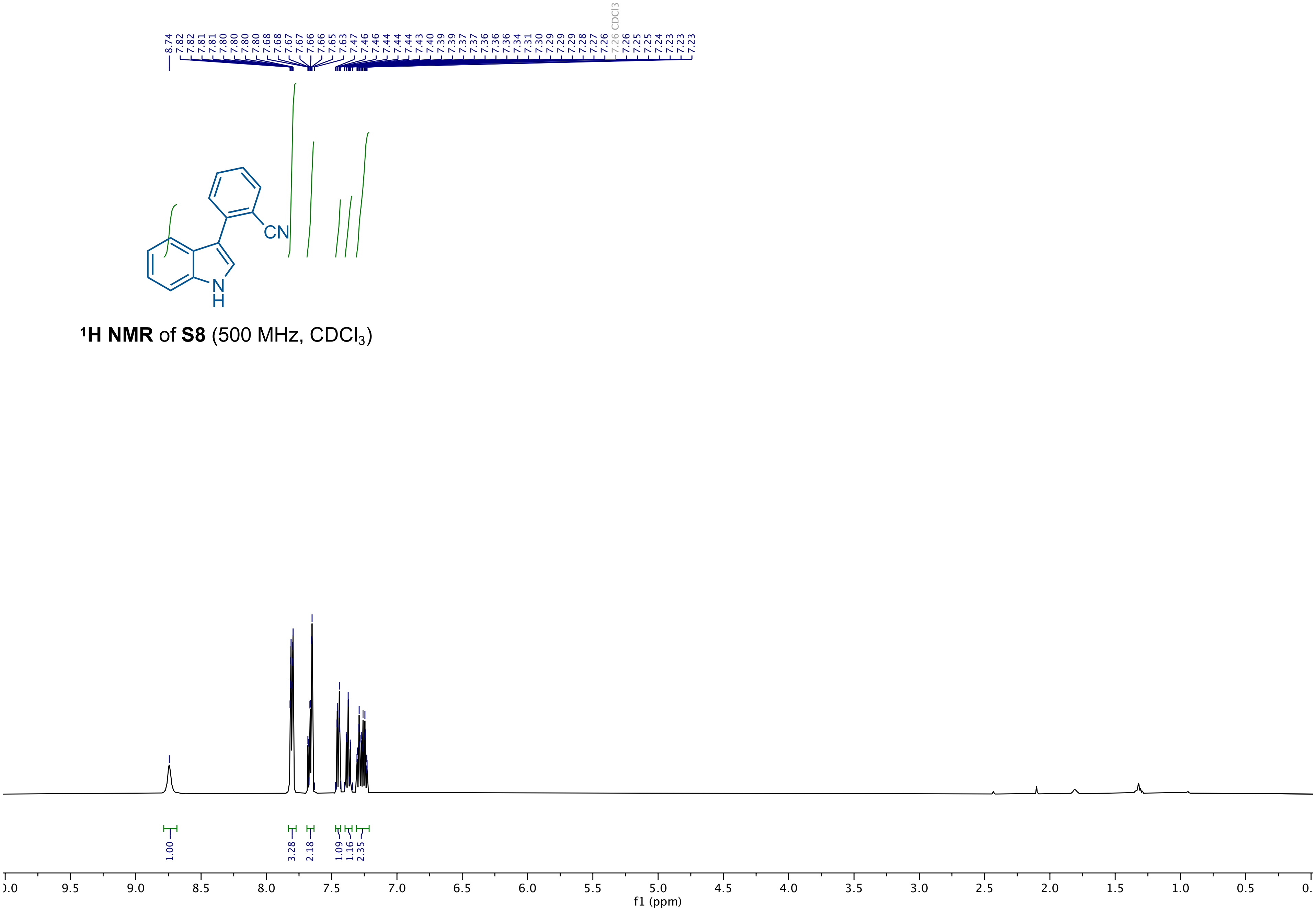

^{1}H NMR of **S8** (500 MHz, $CDCl_3$)

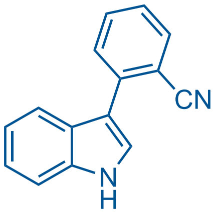

^{13}C NMR of **S8** (126 MHz, $CDCl_3$)

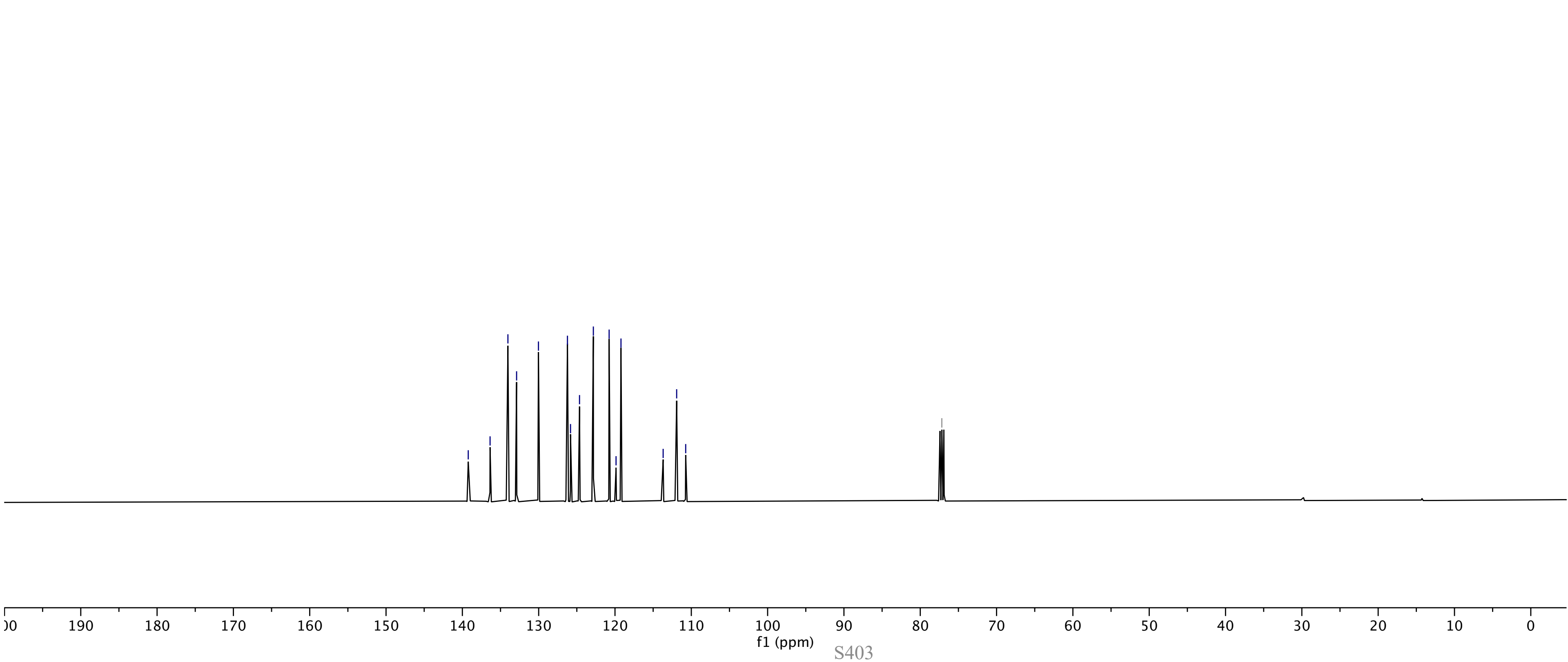

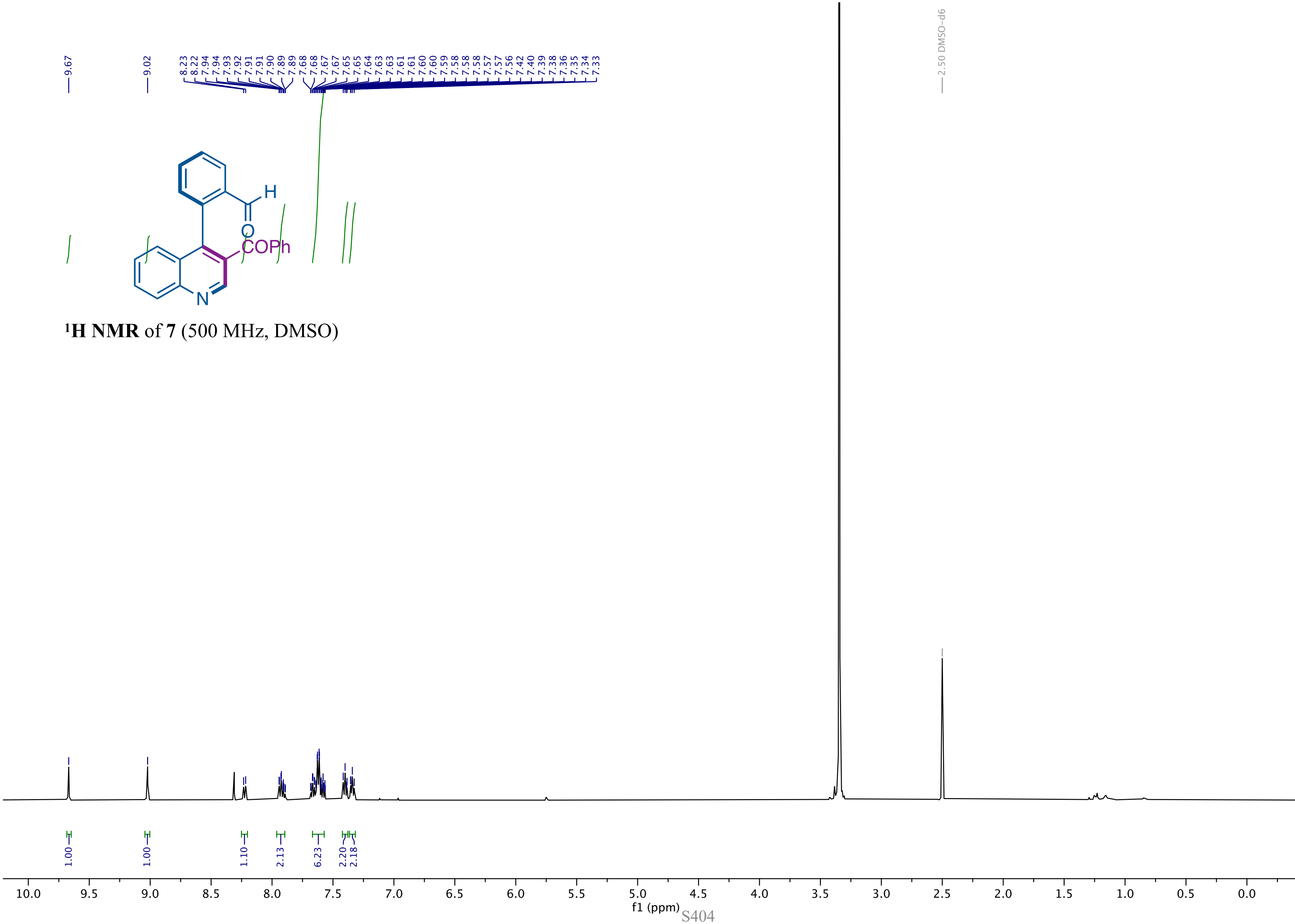

^{1}H NMR of **7** (500 MHz, DMSO)

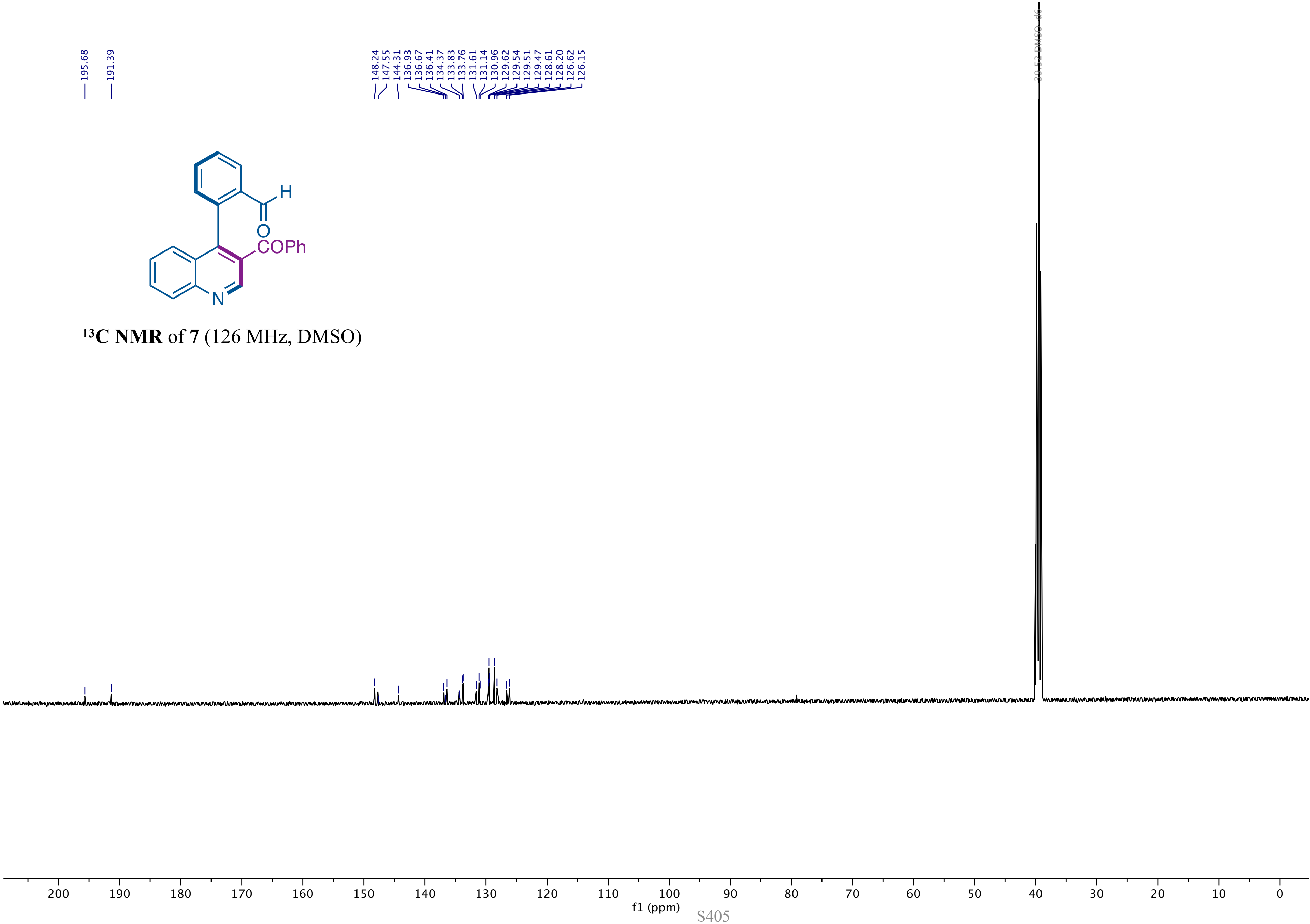

^{13}C NMR of **7** (126 MHz, DMSO)

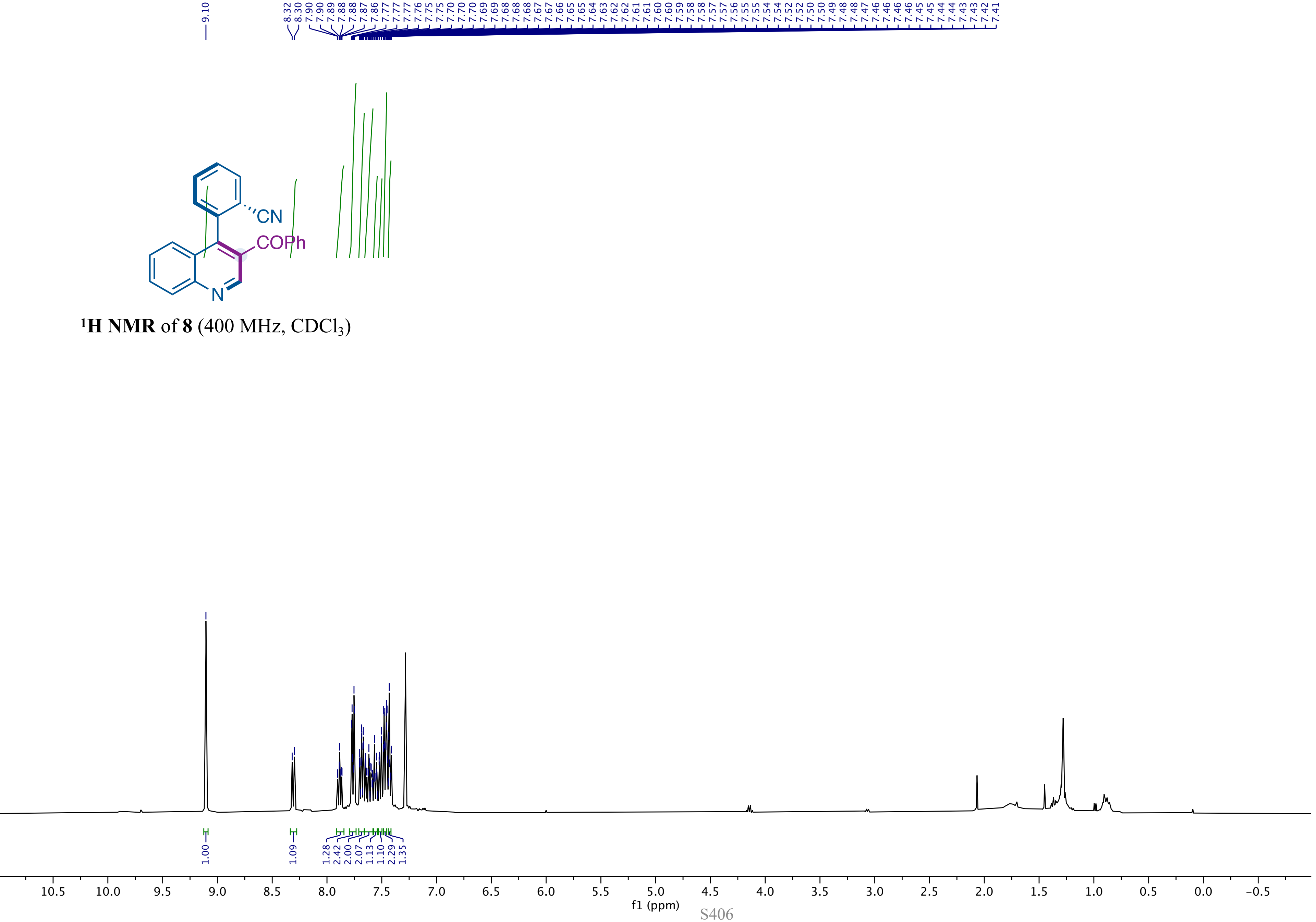

^{1}**H NMR** of **8** (400 MHz, $CDCl_3$)

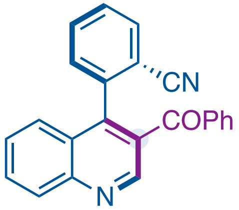

^{13}C **NMR** of **8** (101 MHz, $CDCl_3$)

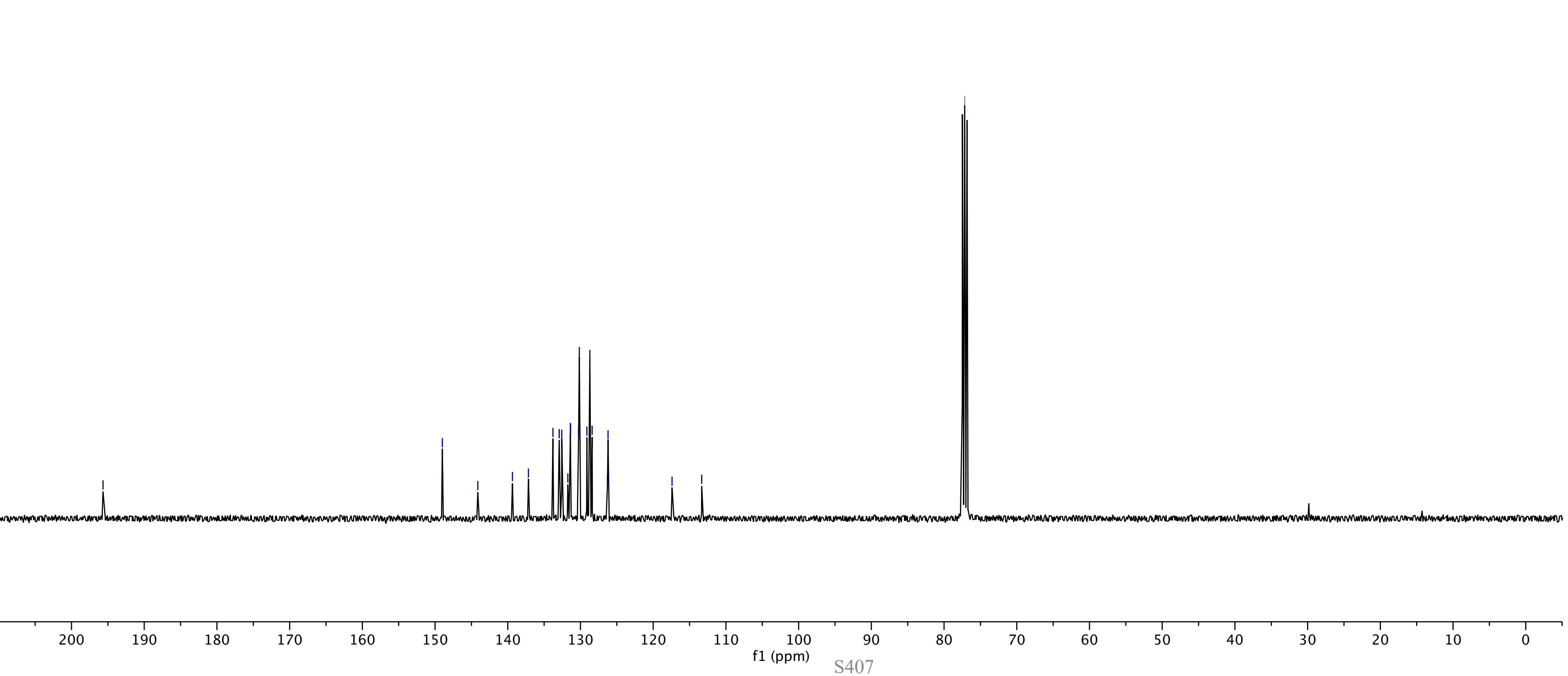

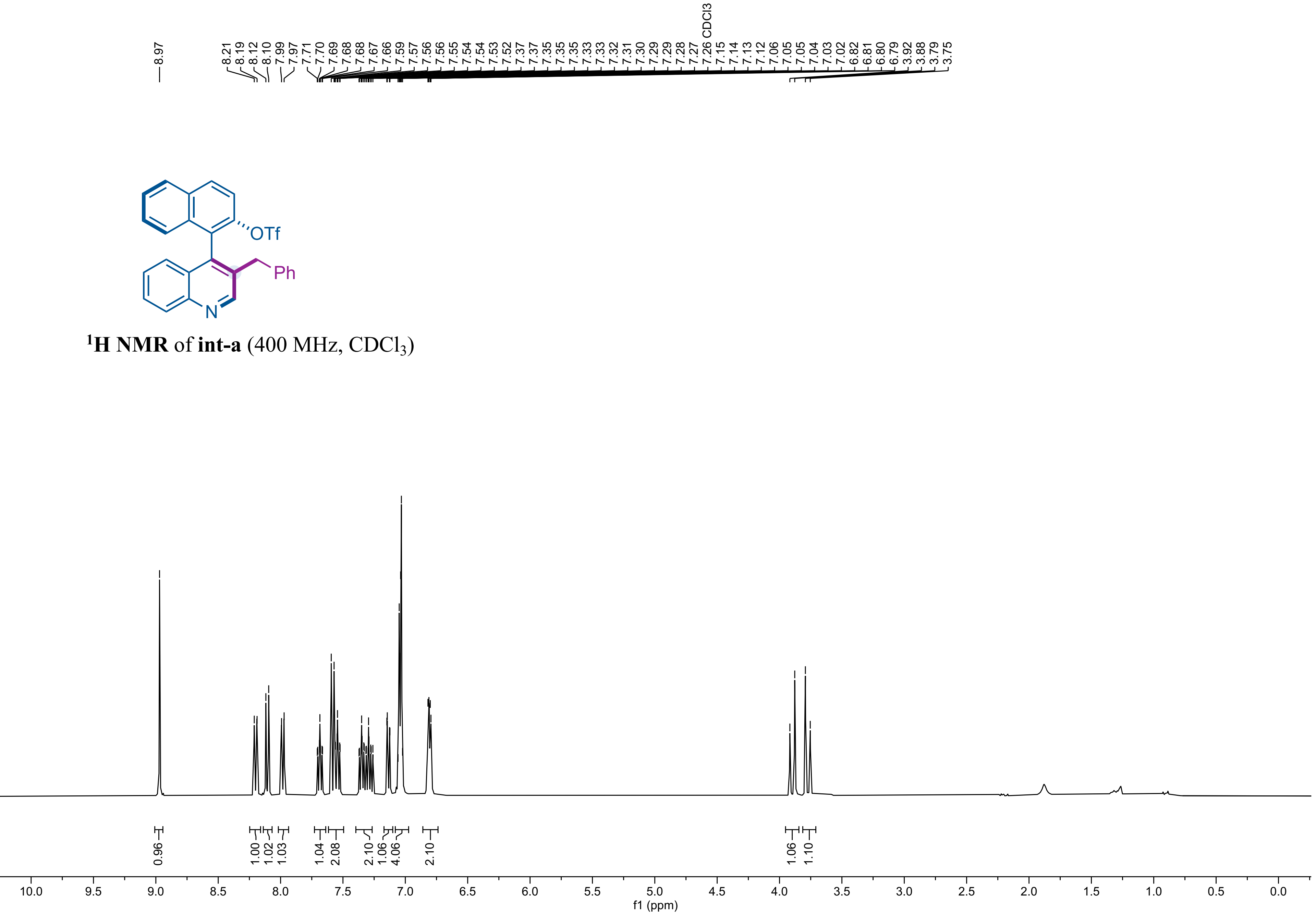

^{1}H NMR of **int-a** (400 MHz, $CDCl_3$)

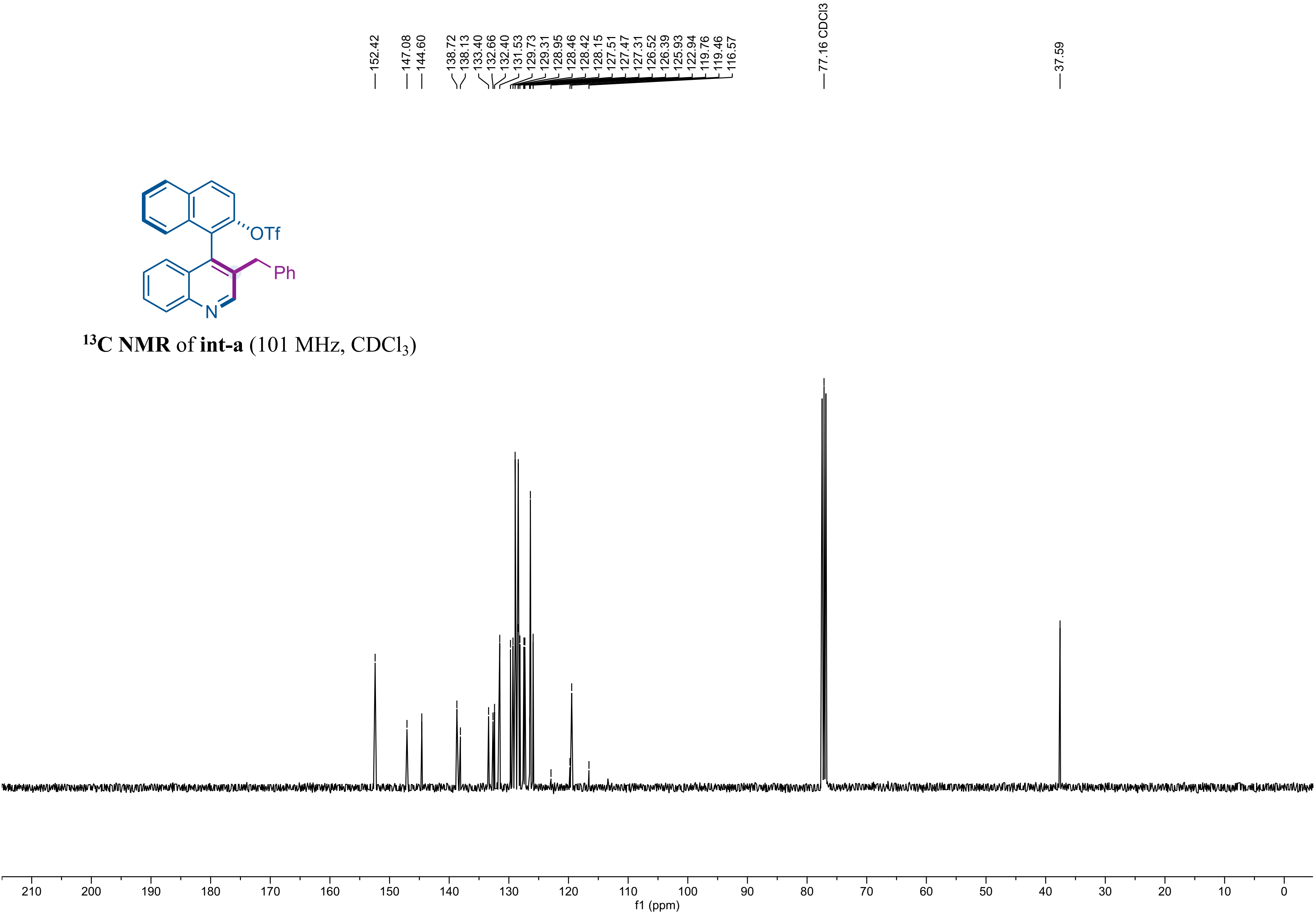

^{13}C NMR of **int-a** (101 MHz, $CDCl_3$)

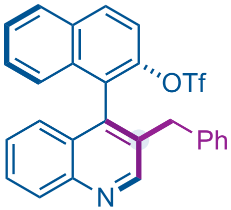

^{19}F NMR of **int-a** (376 MHz, $CDCl_3$)

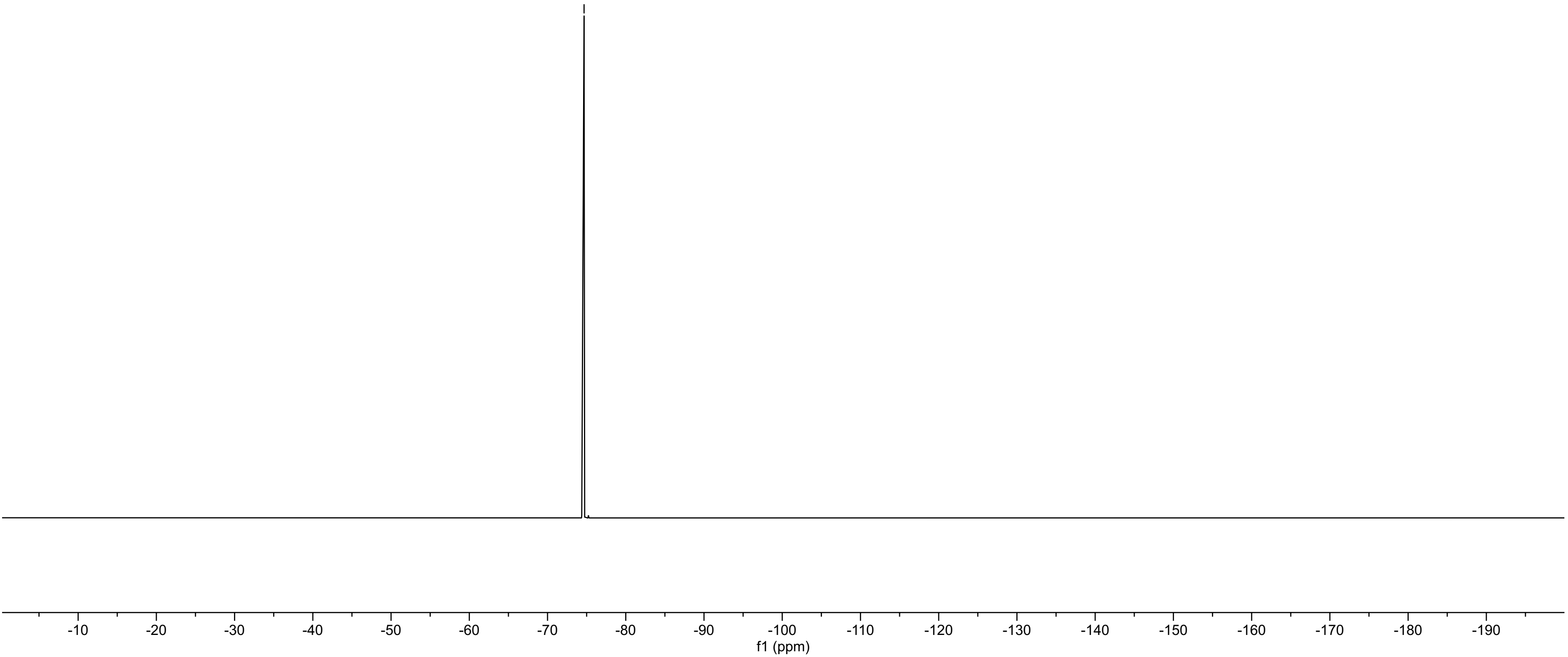

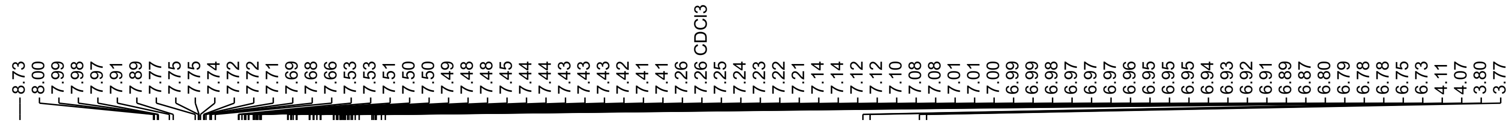

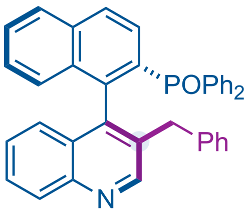

^{1}H NMR of **int-b** (400 MHz, $CDCl_3$)

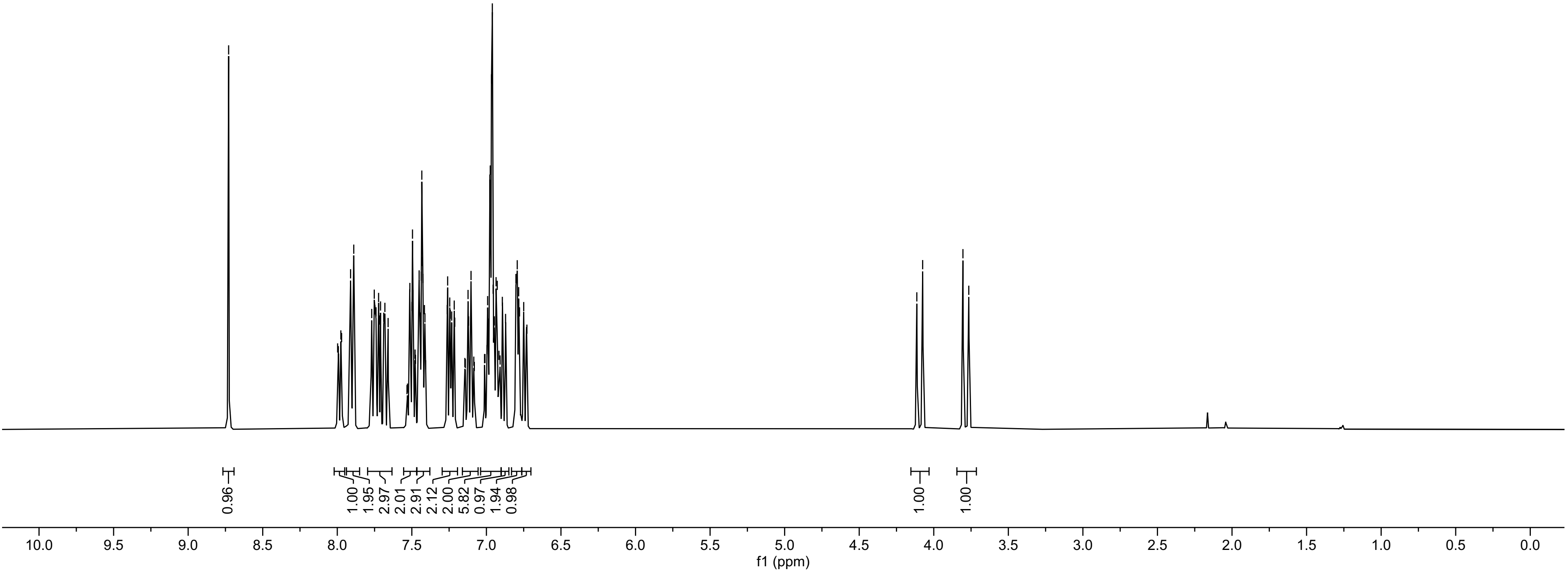

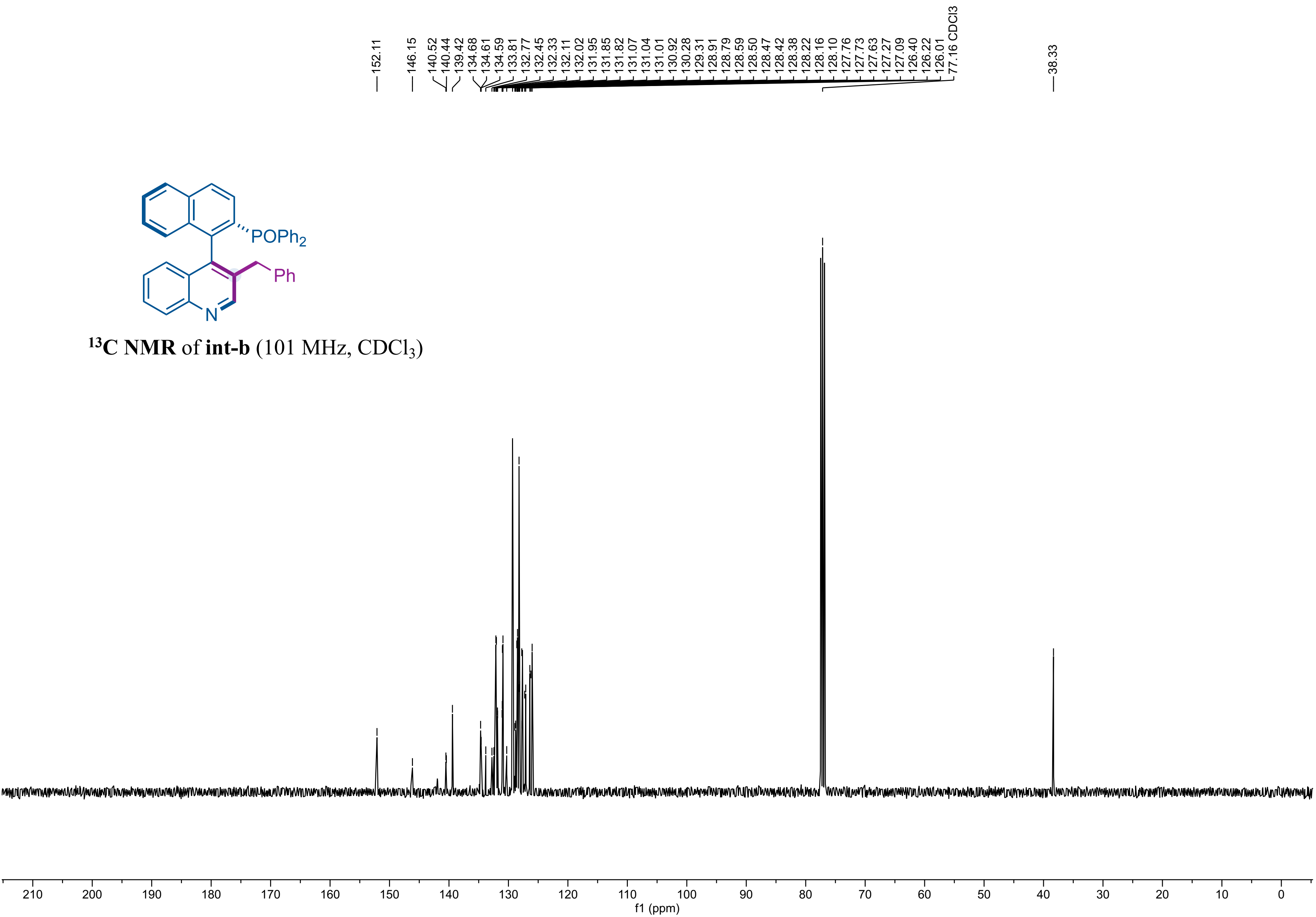

^{13}C NMR of **int-b** (101 MHz, $CDCl_3$)

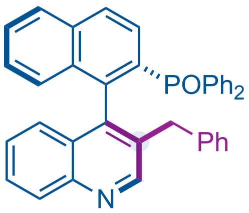

^{31}P NMR of **int-b** (162 MHz, $CDCl_3$)

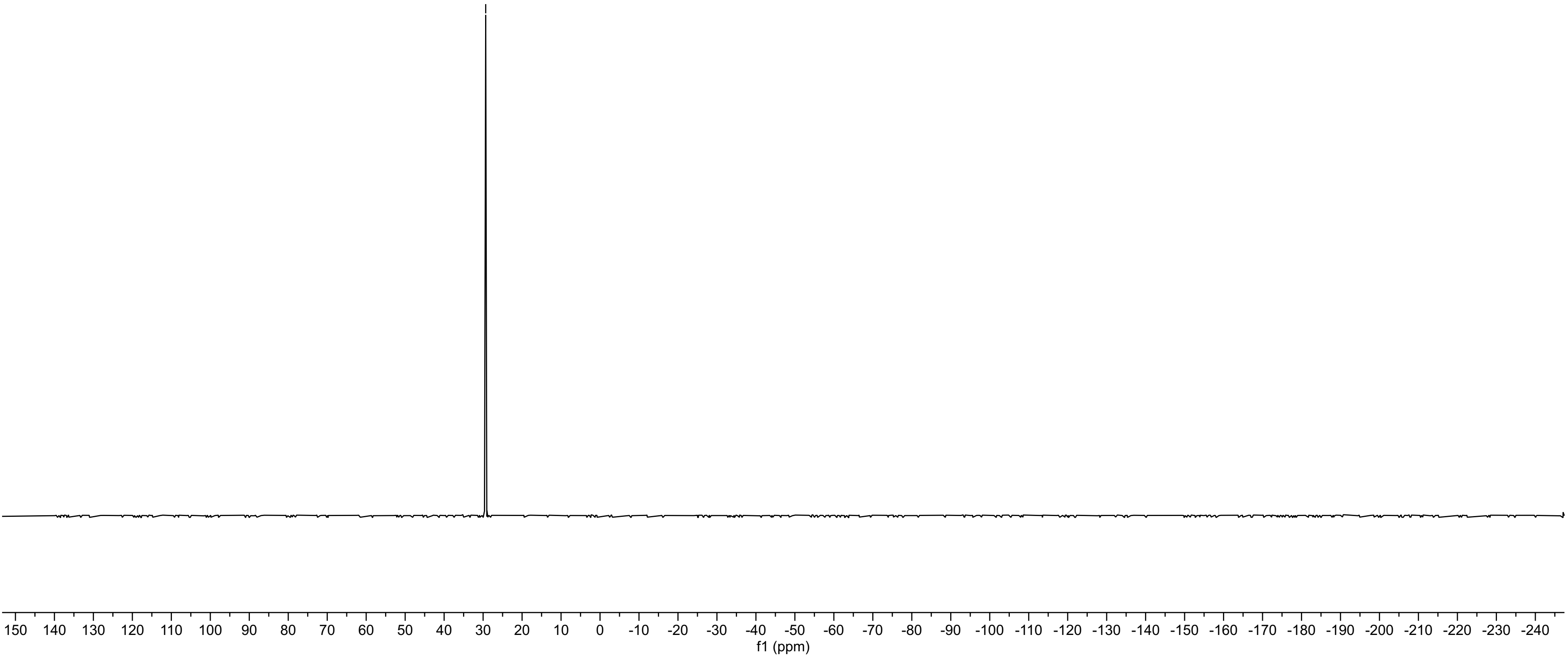

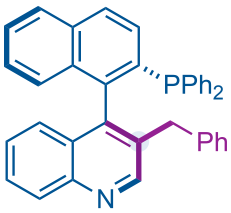

^{1}H NMR of **10** (400 MHz, $CDCl_3$)

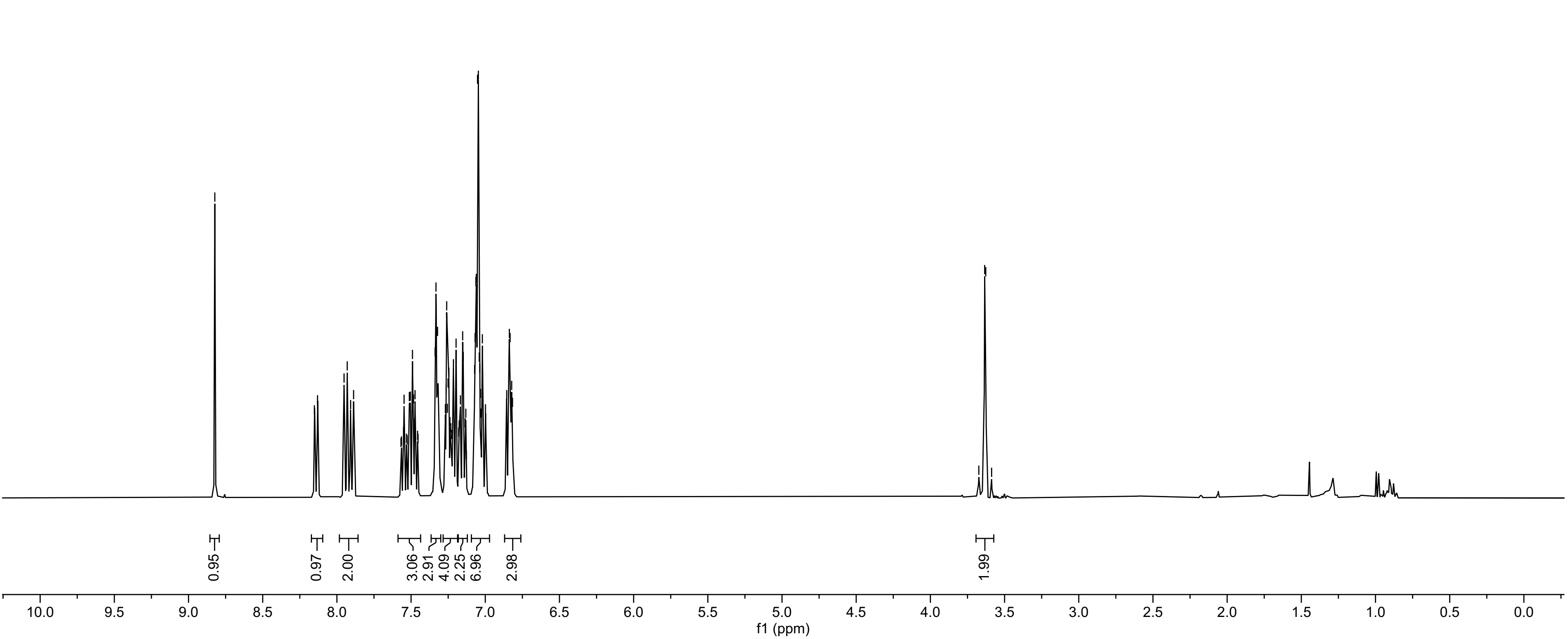

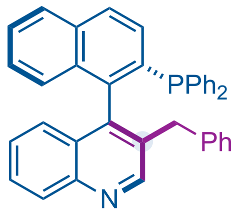

^{13}C NMR of **10** (101 MHz, $CDCl_3$)

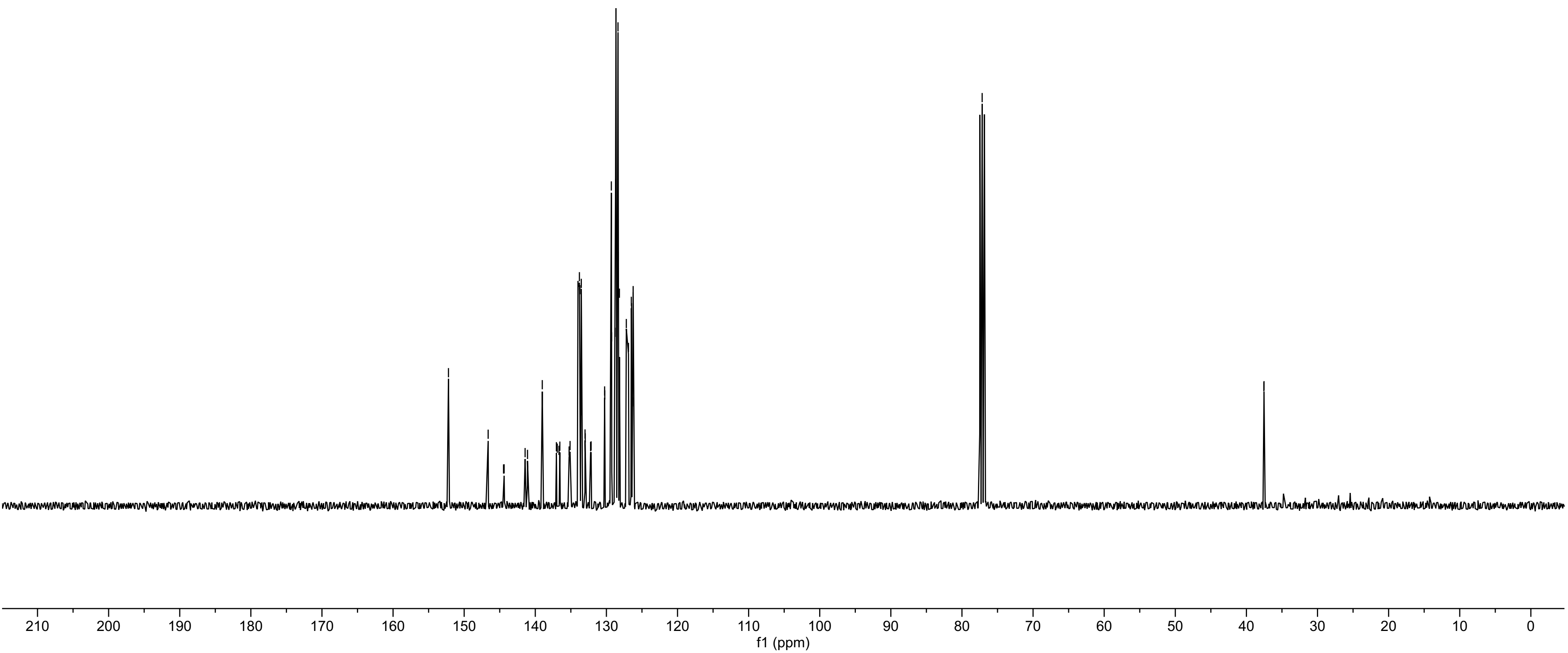

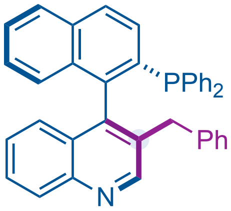

^{31}**P NMR** of **10** (162 MHz, $CDCl_3$)

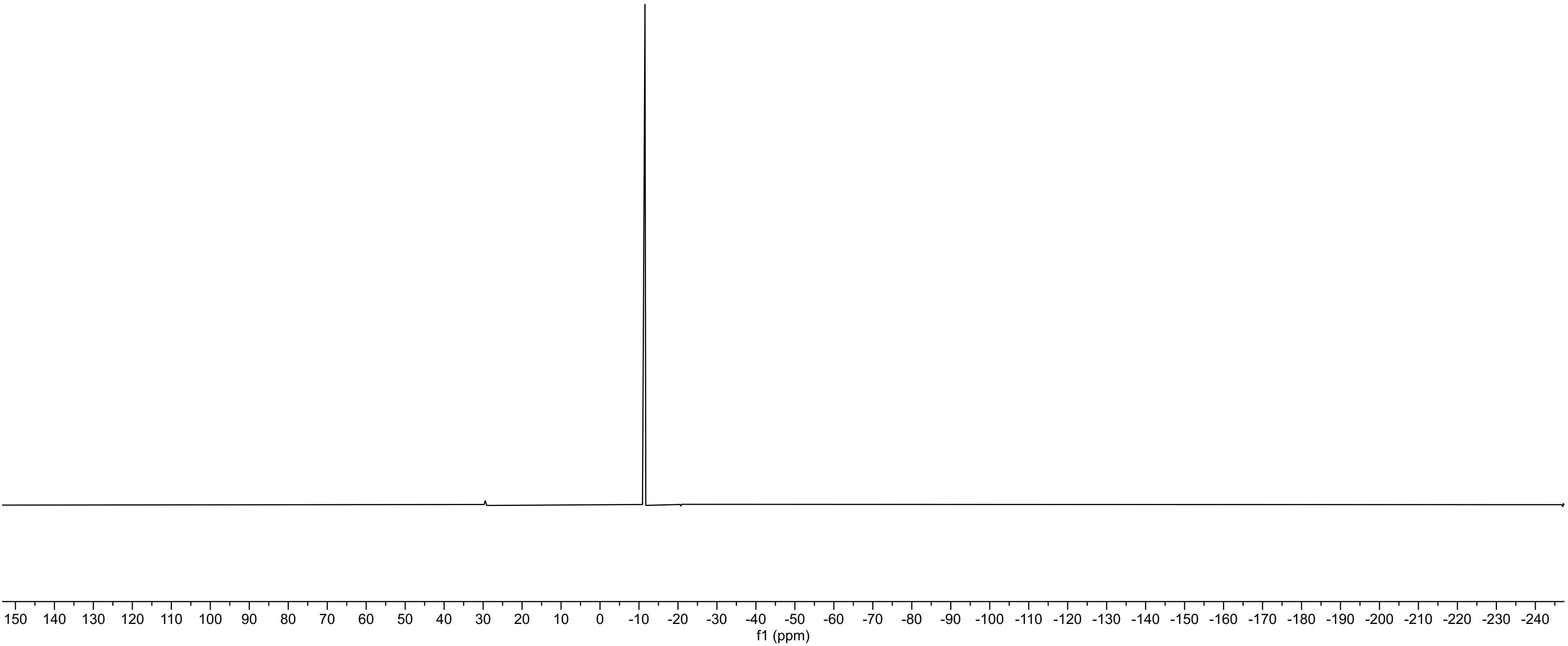

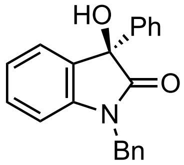

^{1}H NMR of **12** (400 MHz, $CDCl_3$)

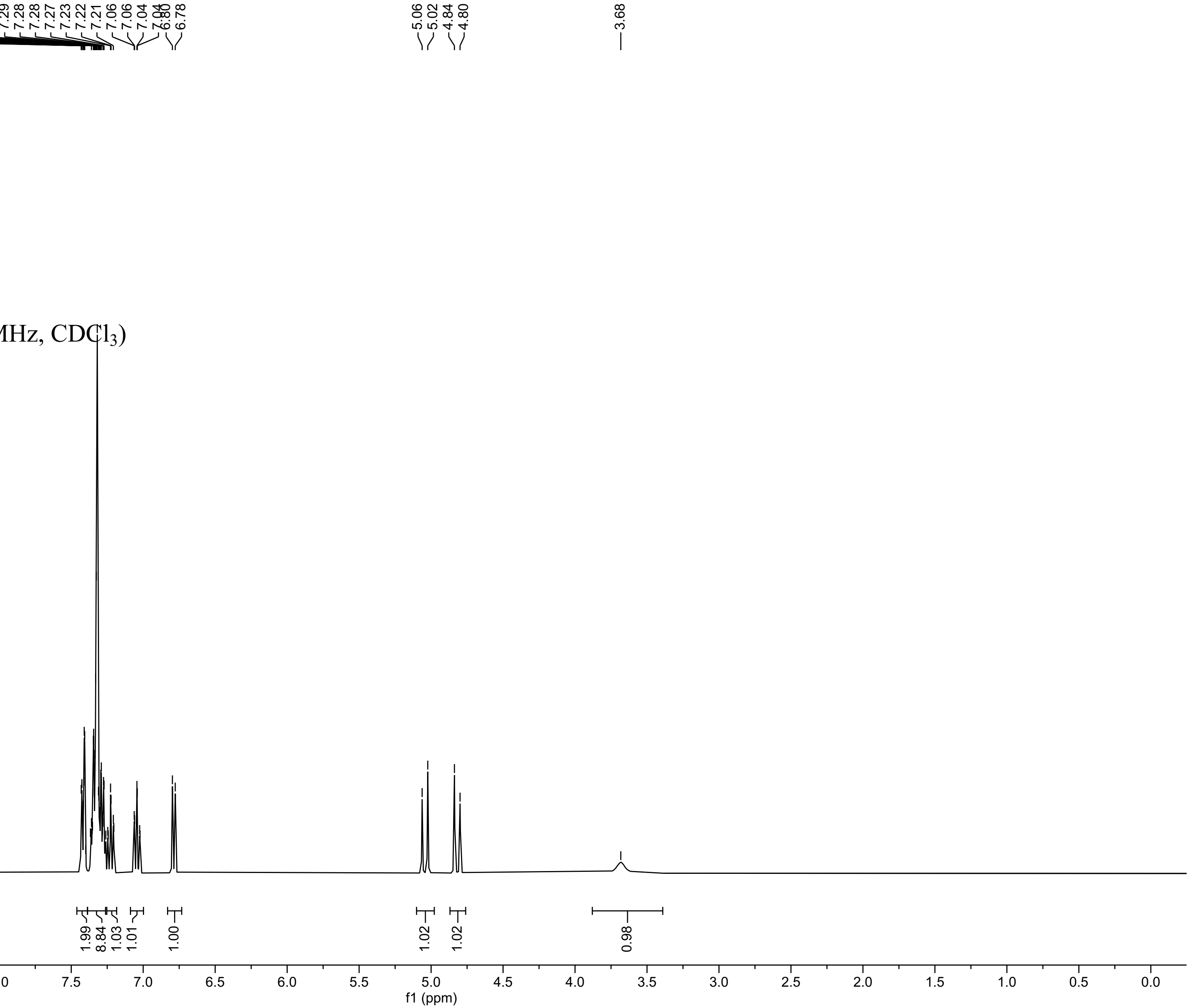

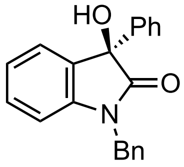

^{13}C NMR of **12** (101 MHz, $CDCl_3$)

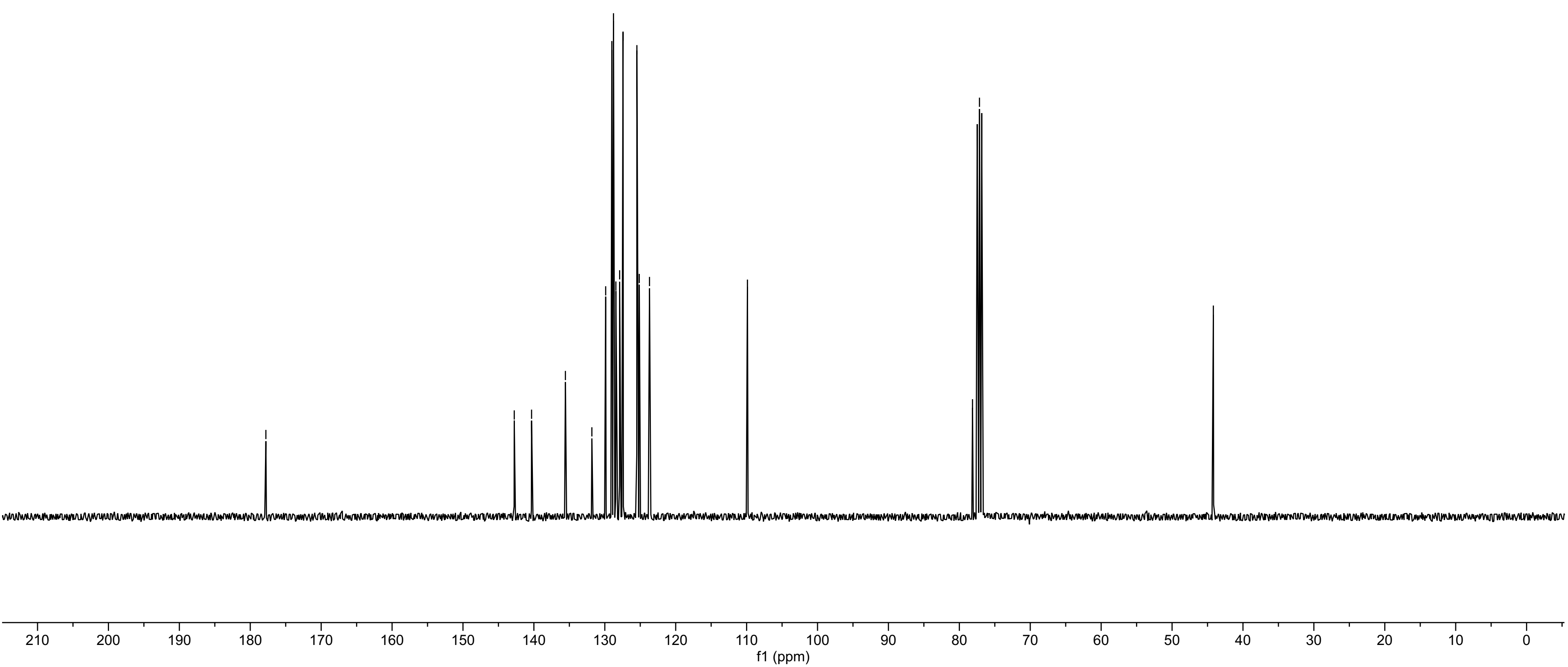

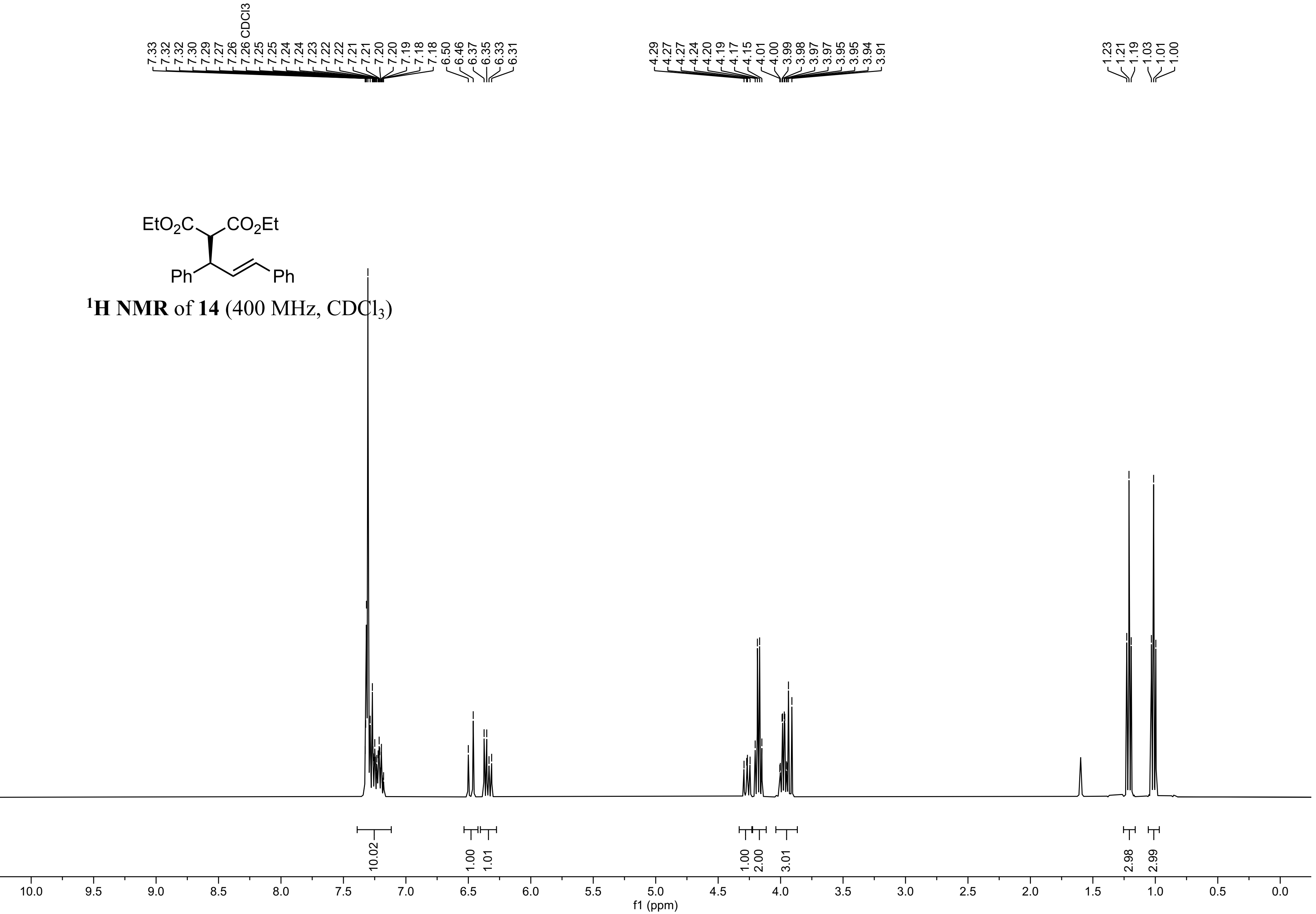

^{1}H NMR of **14** (400 MHz, $CDCl_3$)

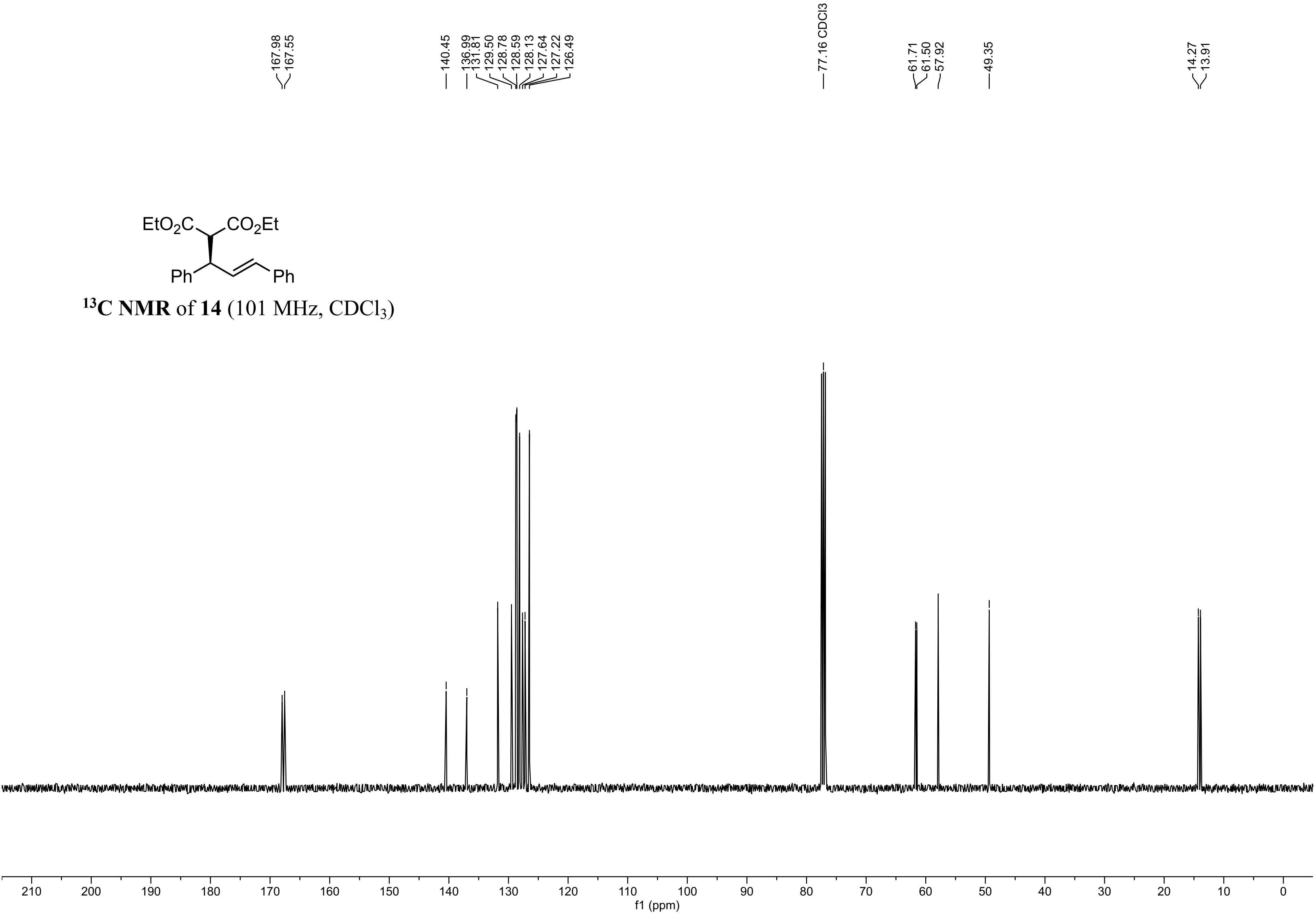

^{13}C NMR of **14** (101 MHz, $CDCl_3$)
